# A general synthesis of dendralenes†
†Electronic supplementary information (ESI) available. CCDC 1902661–1902664 and 1922943–1922945. For ESI and crystallographic data in CIF or other electronic format see DOI: 10.1039/c9sc03976g


**DOI:** 10.1039/c9sc03976g

**Published:** 2019-09-12

**Authors:** Josemon George, Jas S. Ward, Michael S. Sherburn

**Affiliations:** a Research School of Chemistry , Australian National University , Canberra , ACT 2601 , Australia . Email: michael.sherburn@anu.edu.au

## Abstract

The first general synthetic approach to substituted [3]- and higher dendralenes is reported. Fifty-one mono- through to penta-substituted dendralenes carrying alkyl-, cycloalkyl-, alkenyl-, alkynyl-, aryl- and heteroaryl-substitutents are accessed, and the first (*E*)/(*Z*)-stereoselective syntheses of dendralenes are reported (twenty-eight examples). The approach involves twofold Pd(0)-catalyzed Negishi couplings of 1,1-dibromoalkenes with alkenylzinc reagents, and exploits both substrate- and catalyst-controlled aspects of chemo-, regio- and stereoselectivity in the two C(sp^2^)–C(sp^2^) bond forming steps. The value of the new hydrocarbons in rapid structural complexity generation is demonstrated through their deployment in unprecedented diene- and triene-transmissive pericyclic reaction sequences.

## Abstract 2

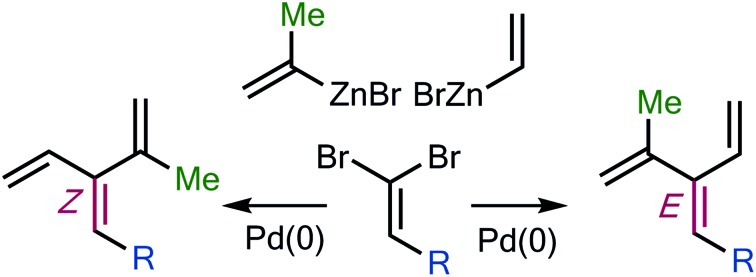
The first broad spectrum dendralene synthesis permits the widest structural and substituent variation and promotes applications in step economic synthesis.

## Introduction

**Fig. 1:**
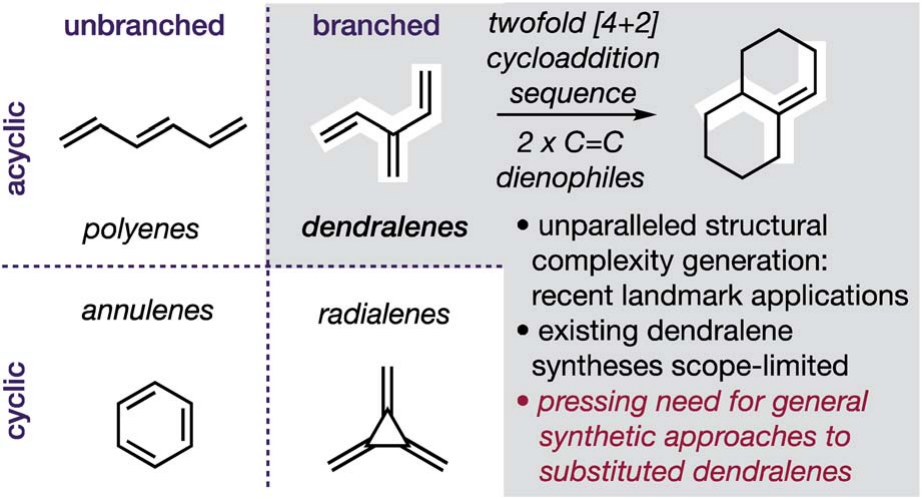
Hydrocarbon design with sp^2^ carbons, the unique synthetic value of dendralenes, and the urgent need for better syntheses.

In hydrocarbons comprising carbon atoms that are sp^2^ hybridized, the absence or presence of chain bifurcations and rings permit four fundamental structural families to be designed (Fig. 1).^1^ Dendralenes are the acyclic, branched class of structures that, until the turn of this century, were widely perceived to be unmanageable.^2^,^3^ [3]Dendralene through [12]dendralene have now been synthesized on scales of hundreds of milligrams to tens of grams^4^ and, building upon ground-breaking investigations primarily from the Tsuge^5^ and Fallis^6^ groups, the first applications of dendralenic building blocks in the most step economic total syntheses have recently been published.^7^ Dendralenes are useful building blocks for the swift generation of complex structures due to their unique ability to undergo sequential additions in an interconnected (*i.e.* diene-transmissive) manner.^3^,^8^,^9^

As the potential of dendralenes has been more widely recognized, numerous publications focusing on dendralene synthesis have recently appeared.^10^ The rapid expansion of interest in dendralenes, combined with the paucity of synthetic methods to access them, renders the recent contributions significant. Nonetheless, these existing methods are limited, in that they permit the synthesis of heavily restricted subsets of structures.^11^ Furthermore, none of these existing approaches permit the stereoselective preparation of dendralenes. Herein, we introduce a direct method for acyclic branched oligo-alkene synthesis that (a) tolerates a wider variety of substituent types; (b) permits greater diversity in the number of substituents; (c) represents the first stereoselective synthesis of dendralenes, whilst also being shorter in step count than existing methods.

**Fig. 2:**
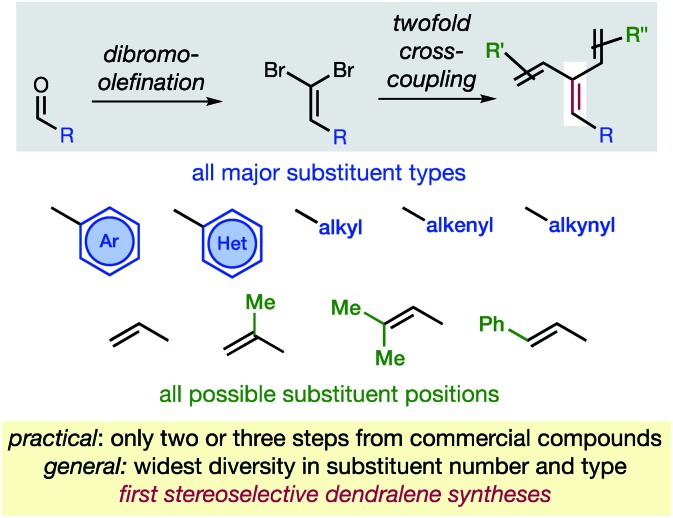
The first practical and general synthesis of [3]dendralenes.

The new approach permits the synthesis of dendralenes bearing the most common substituents (alkyl, cycloalkyl, alkenyl, alkynyl, aryl and heteroaryl groups) in only two or three steps from commercially-available aldehydes through a robust sequence involving Ramirez dibromomethylenation^12^ and Negishi cross-coupling^13^ reactions (Fig. 2).

Building upon the foundations of previous, narrow-scope cross-coupling methods for dendralene synthesis,^4^,10b,10d,^14^ and ground-breaking work from the Negishi laboratory,^15^,^16^ we establish a method that is unparalleled in its ability to generate dendralenic structural variety. Until now, no published dendralene preparation has addressed the diastereoselective synthesis of internally-substituted systems. This is a challenging and unsolved problem since it requires the stereoselective preparation of a tri-substituted C=C bond, whereupon two non-equivalent (but very similar) alkenyl-substituents are attached to the same carbon. We provide solutions that are of broad scope, allowing selective access to both *E*- and *Z*-diastereomers of an internally-substituted dendralene from the same 1,1-dibromoalkene precursor.

## Results and discussion

The first group of [3]dendralenes reported here lack stereogenicity about the central C=C bond. Thus, 1,1-dibromoalkenes **1** undergo twofold Negishi C(sp^2^)–C(sp^2^) coupling^17^ with unsubstituted and substituted alkenylzinc bromides to furnish dendralenic products **2**, where the two newly introduced alkenyl substituents are the same. Table 1 depicts 23 examples of twofold cross-couplings between 13 different 1,1-dibromoalkenes **1** and four different alkenyl nucleophiles, to demonstrate the broad scope of this lynchpin^4^ strategy.

**Table 1:** Synthesis of [3]dendralenes **2a–w**, lacking stereogenicity about the central C=C bond, by twofold C(sp^2^)–C(sp^2^) cross-coupling of 1,1-dibromoalkenes **1**^*a*^

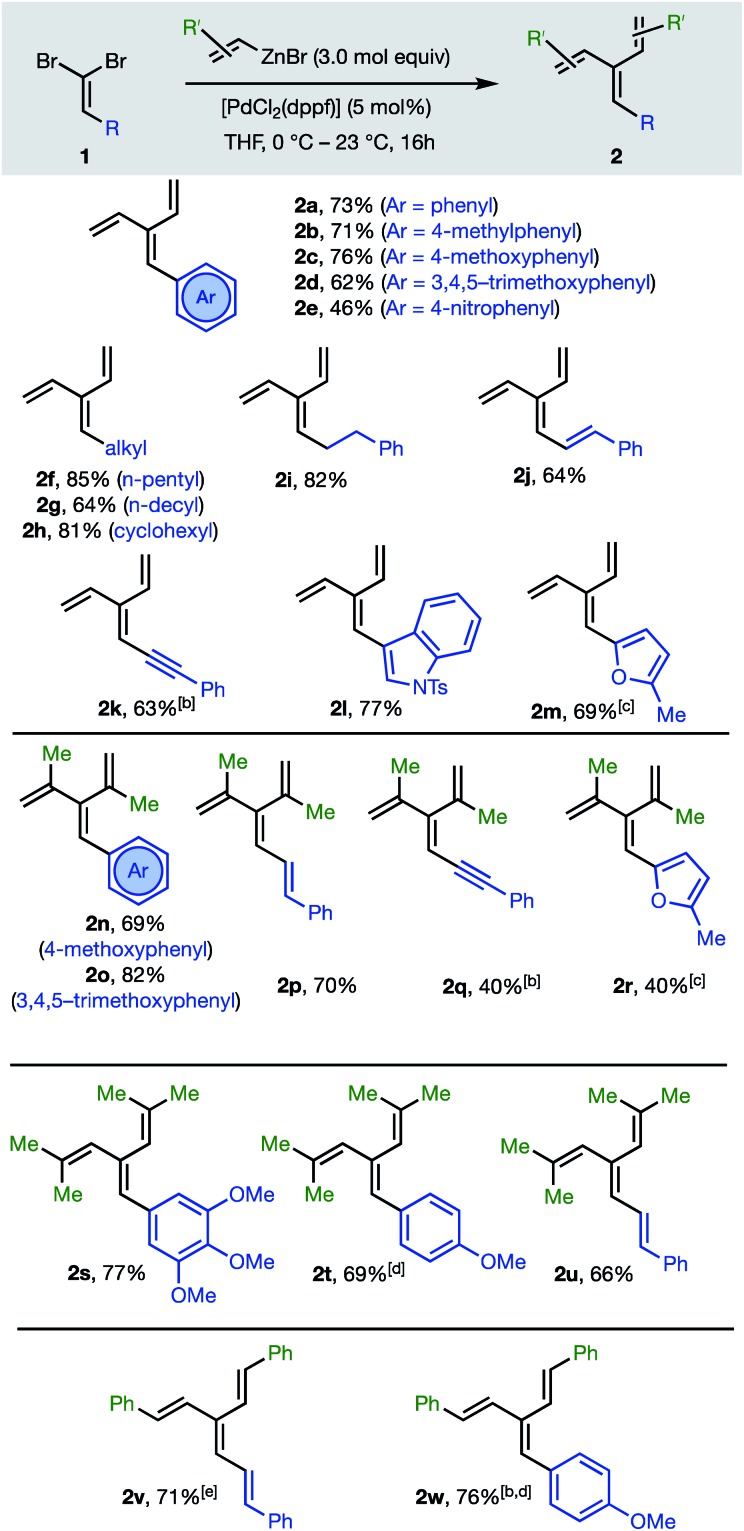

^*a*^All reactions were performed according to the conditions in the scheme with the following exceptions.

^*b*^Reflux, 4 h.

^*c*^Reflux, 16 h.

^*d*^ZnBr_2_ (5 mol equiv.) and Grignard reagent (4 mol equiv.) used.

^*e*^Reflux, 14 h.

The twofold Negishi cross-coupling protocol works well with vinylzinc bromide and its alkyl and aryl-substituted congeners, to access mono- to penta-substituted [3]dendralenes **2a–w** with substitution at all possible sites on the dendralene framework. Substituents incorporated at the central methylene position of the [3]dendralene (substituents colored blue in Table 1) include acyclic and cyclic primary and secondary alkyl-groups, carbocyclic and heterocyclic aromatic groups of diverse electronic characteristics, and alkenyl- and alkynyl-substituents. While several (pre)catalysts were effective, [PdCl_2_(dppf)] was superior for twofold cross-coupling.

To achieve the first diastereoselective synthesis of dendralenes, we envisioned two successive cross-couplings of a 1,1-dibromoalkene **1** with different alkenylzinc reagents. It is well established that aldehyde-derived 1,1-dibromoalkenes undergo chemo- and regio-selective single cross-coupling with an alkenyl nucleophile to replace the bromine *trans*- to the carbon-based substituent (Table 2, **1** → **3**).^18^

**Table 2:** Stereoselective synthesis of [3]dendralenes **4a–l** involving sequential C(sp^2^)–C(sp^2^) cross-couplings of 1,1-dibromoalkenes **1** with C(sp^2^) stereo-retention

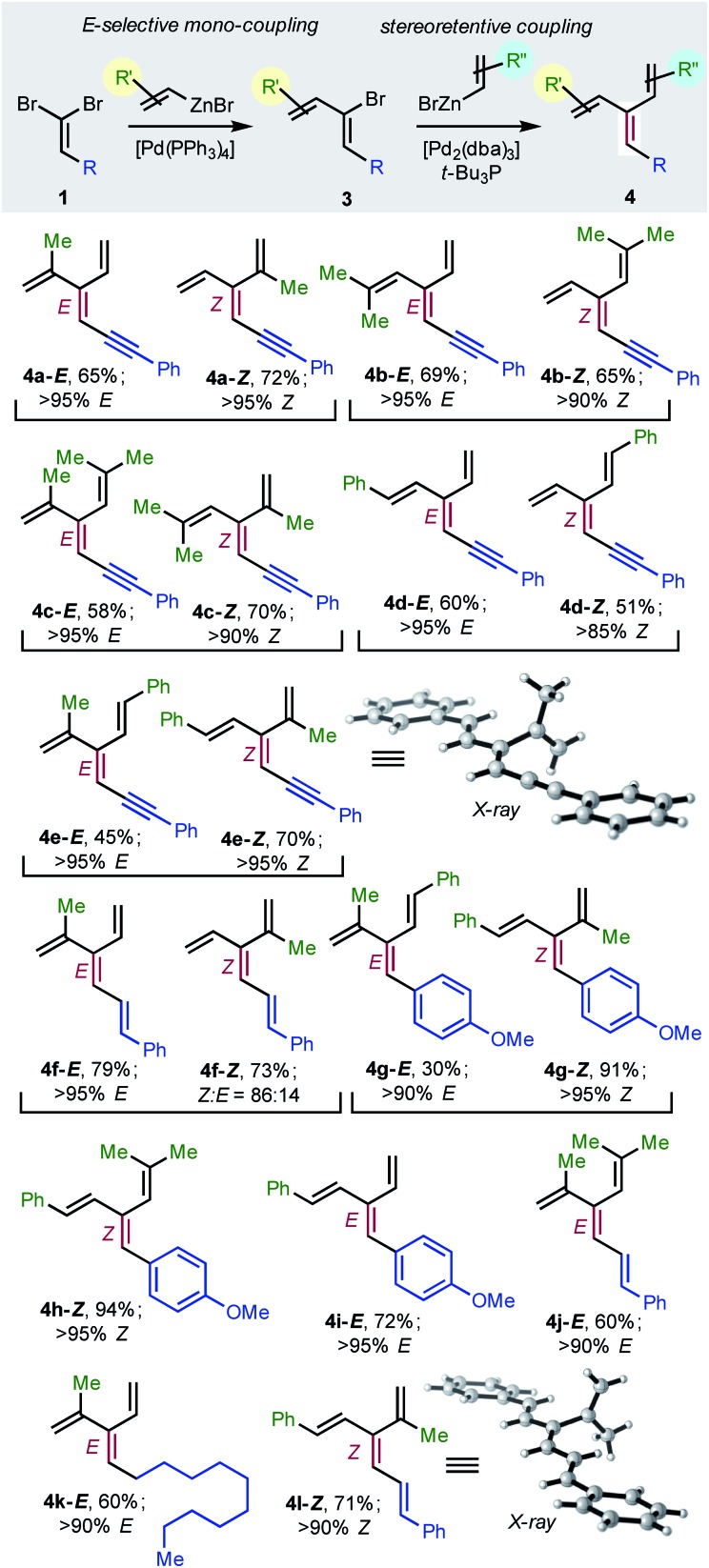

In our hands, [Pd(PPh_3_)_4_] was the most consistent performer in the *trans*-selective mono-coupling of 1,1-dibromoalkenes (**1** → **3**). In all but one case examined, a clear preference for the mono-coupled product was observed and, in every case, only the (1*Z*)-2-bromo-1,3-butadiene diastereomer was detected.^19^ A little fine-tuning of the reaction was needed for each substrate (*e.g.* stoichiometry of coupling partners, temperature) for optimal results. (The ESI† contains 18 examples involving seven different 1,1-dibromoalkenes **1** and four different alkenylzinc cross-coupling partners.)

Negishi reported that cross-couplings of the resulting (1*Z*)-2-bromo-1,3-dienes **3** with methyl-, ethyl-, *n*-butyl- or phenyl-zinc bromide proceed with retention^15^ of configuration at the sp^2^-C initially carrying the bromine with [(*t*-Bu_3_P)_2_Pd] as catalyst. We are delighted to report that the same ligand also brings about Pd(0)-catalyzed stereo-retentive cross-couplings of (1*Z*)-2-bromo-1,3-dienes with alkenyl nucleophiles to permit the first stereoselective dendralene synthesis (Table 2, **3** → **4**).

Consistent with the results of the Negishi group with non-olefinic nucleophiles,^15^ we find that alkenyl-zinc bromides exhibit wide scope in Pd(0)/*t*-Bu_3_P-catalyzed stereo-retentive couplings involving (1*Z*)-2-bromo-1,3-butadienes. Inconsistent with Negishi's findings, however, are couplings of alkyl-substituted systems (Table 2, **3** → **4**, **R** = alkyl), which generally give mixtures of *E* and *Z*-diastereomers in the second cross-coupling (see ESI† for details). Fortuitously, these substrates work well in cross-couplings with [PdCl_2_(dppf)] as pre-catalyst, which proceed with stereochemical inversion^16^,^20^ (Table 3, **3** → **4**).

**Table 3:** Stereoselective synthesis of [3]dendralenes **4k**, **4m**, and **4n** involving sequential C(sp^2^)–C(sp^2^) cross-couplings of 1,1-dibromoalkenes **1** with C(sp^2^) stereo-inversion

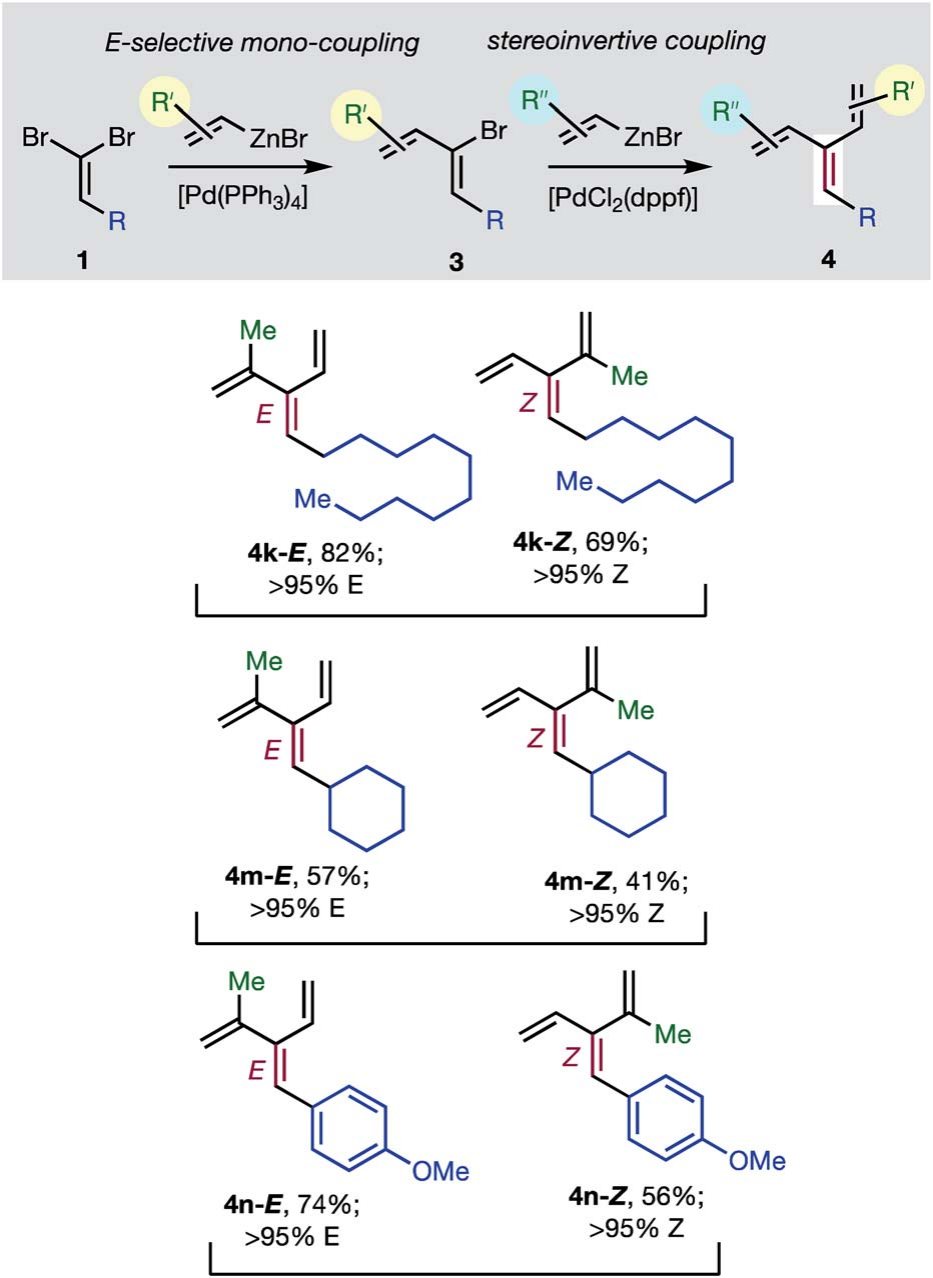

**Scheme 1:**
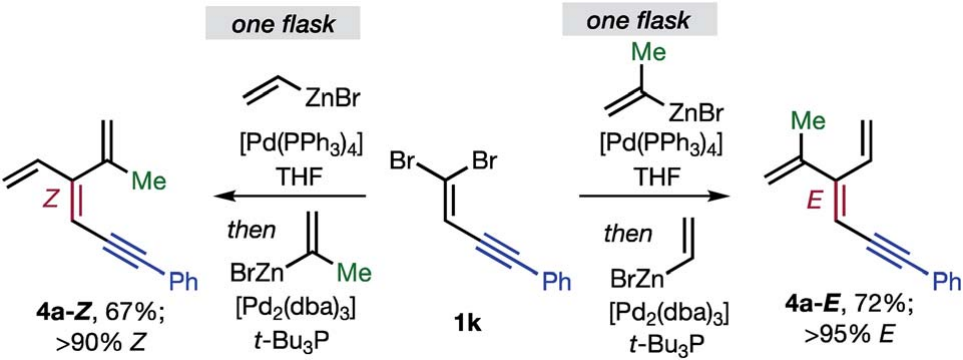
One flask stereoselective syntheses of *E*- and *Z*-diastereomers of dendralene **4a** from 1,1-dibromide **1k**.

Overall, the sequence involving Pd(0)/*t*-Bu_3_P-catalyzed stereo-retentive cross-coupling (Table 2) has broader scope than the alternative stereo-invertive pathway with [PdCl_2_(dppf)] pre-catalyst (Table 3). Nineteen [3]dendralenes, prepared through stereo-retentive couplings, are shown in Table 2 and six from stereo-invertive couplings are depicted in Table 3. Collectively, Tables 2 and 3 describe the stereoselective synthesis of ten pairs of *E* and *Z* diastereomeric dendralenes. Operationally, each pair of diastereomers is synthesized through a complementary pair of sequences (*i.e.***1** → **3** → **4**) in which the order of addition of the two non-equivalent alkenyl-zinc bromides is reversed. As shown in Scheme 1, the two couplings can be performed in the same flask, through successive additions of two pairs of catalysts and reagents. Thus, the previously unsolved problem of stereoselective dendralene synthesis is reduced to the simple task of (a) selecting whether a stereo-retentive or -invertive pathway is required, and (b) performing two cross-couplings with two different alkenyl-zinc reagents.

The geometry of every stereogenic dendralene (Tables 2 and 3) was assigned by NOE experiments, with two X-ray crystal structures supporting these assignments. The molecular structures of **4e-*Z*** and **4l-*Z*** (Table 2) exhibit essentially in plane conformations of the longest through-conjugated segment of each structure, namely 1,6-diphenyl-1,3-hexadien-5-yne and 1,6-diphenyl-1,3,5-butatriene, respectively. Both structures carry isopropenyl substituents, which are skewed at angles of 77° and 83° out of plane.

**Scheme 2:**
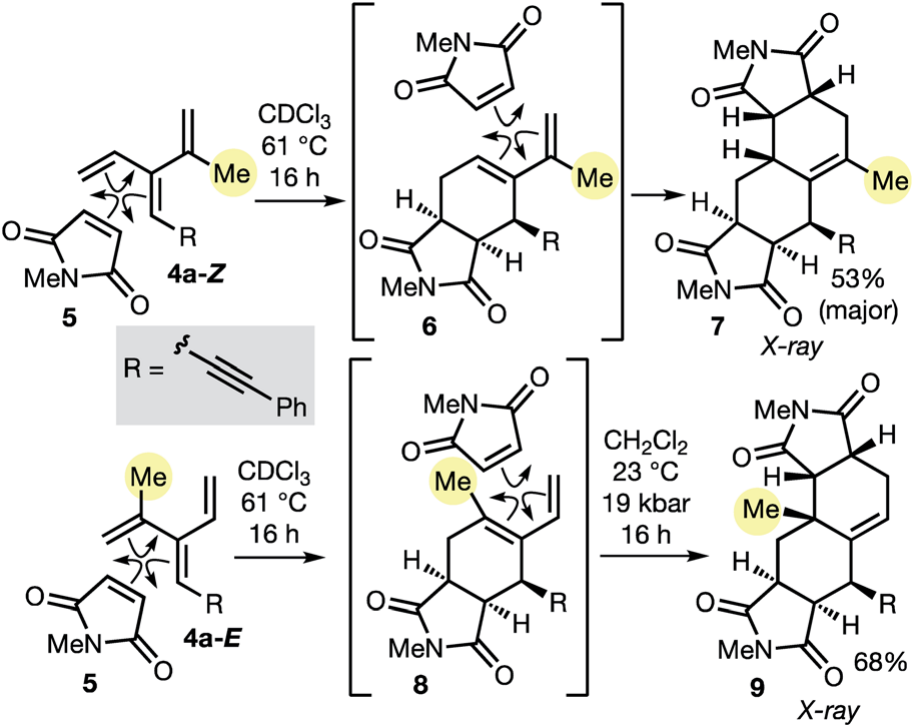
DTDA sequences on each of the two diastereomers of [3]dendralene **4a** grants access to constitutional isomers of a Δ^1(9)^-octalin **7** and **9**. All four cycloadditions are endo-selective and the second of each sequence exhibits π-diastereofacial selectivity.

As described in the introduction, the most important feature of dendralenes is their participation in diene-transmissive Diels–Alder (DTDA) sequences to form octalins. The present work significantly extends the scope of the DTDA process since, as we demonstrate here for the first time in Scheme 2, geometrical isomers of [3]dendralenes give different constitutional isomers of twofold cycloadducts.

Thus, each diastereomeric [3]dendralene **4a-*Z*** and **4a-*E*** reacts with the dienophile *N*-methylmaleimide (NMM, **5**) with complete selectivity for the 1,3-butadiene site that lacks the inside-1,3-butadiene R substituent.^21^ Substrate **4a-*Z*** gives semicyclic diene **6**, which reacts on, *in situ*, with a second NMM molecule to furnish 1-methyl-Δ^1(9)^-octalin **7** as the major product. The semicyclic diene **8** derived from diastereomeric dendralene **4a-*E*** undergoes a second NMM cycloaddition under high pressure conditions to form 10-methyl-Δ^1(9)^-octalin **9**, possessing an angular methyl substituent. Octalin and decalin ring systems are extremely common structural motifs in natural products and medicinal agents.^22^

**Scheme 3:**
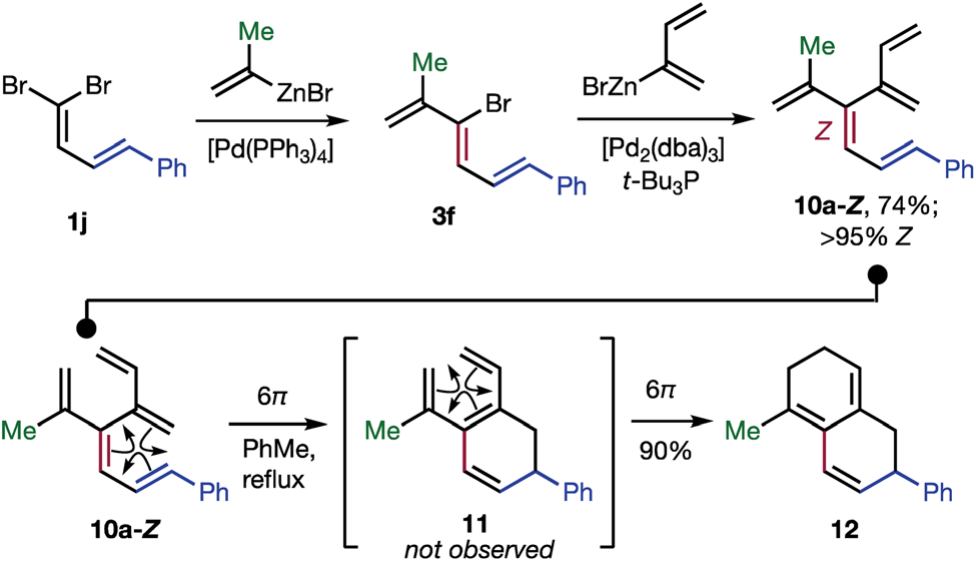
The first stereoselective synthesis of a [4]dendralene and an unprecedented triene-transmissive twofold 6π-electrocyclization sequence.

The 48 dendralenes depicted in Tables 1–3 conclusively demonstrate the broad scope of this method for substituted [3]dendralene synthesis. Scheme 3 shows that the same approach permits the first stereocontrolled synthesis of a substituted [4]dendralene^10^**10a-*Z***, by simply deploying 2-(1,3-butadienyl)zinc bromide as a coupling partner (see the ESI† for two more examples).

The freshly minted *Z*-configuration of the trisubstituted C=C unit of [4]dendralene **10a-*Z*** is essential for the first twofold, triene transmissive 6π–6π electrocyclization sequence (**10a-*Z*** → **11** → **12**). The execution of a pair of electrocyclizations in this interconnected manner is without precedent. Several variations upon this original theme can be envisaged, which has potential for development into a broad scope, new method for step economic polycycle synthesis.

## Conclusions

In conclusion, the first broad-spectrum synthesis of substituted dendralenes has been demonstrated, and unprecedented domino sequences for polycycle construction proven. [3]Dendralenes bearing from one to five alkyl, cycloalkyl, alkenyl, alkynyl, aryl and heteroaryl substituents have been prepared. Substitution at every conceivable position on the [3]dendralene framework has been realized. The previously unsolved problem of diastereoselective synthesis of internally-substituted systems has been solved. The method has been shown to work also with [4]dendralenes. Importantly, the approach represents the first general synthesis of dendralenic structures with extended C=C and C≡C through-conjugation, as evidenced by the preparation of 23 new compounds containing this feature. We venture that the findings described herein, when combined with the strategies recently introduced for the preparation of the unsubstituted higher [*n*]dendralenes (*n* = 5–12),^4^ will permit the chemical synthesis of any conceivable dendralenic structure in short order, and lead to new applications in step economic total syntheses.

## Conflicts of interest

There are no conflicts to declare.

## Supplementary Material

Supplementary information

Crystal structure data

Crystal structure data
