# Supplementary material for: A general synthesis of dendralenes
Source: Chem Sci. 2019 Sep 12;10(43):9969–73. doi: 10.1039/c9sc03976g (PMC7003925; doi:10.1039/c9sc03976g)

Electronic Supplementary Material (ESI) for Chemical Science.
This journal is © The Royal Society of Chemistry 2019

Supporting Information

A General Synthesis of Dendralenes

(Josemon George, Jas S. Ward, and Michael S. Sherburn)

Contents

Analyses of the Widest Scope Substituted [3]Dendralene Syntheses **S3**

Additional Findings on Retention/Inversion Experiments **S5**

General Methods **S7**

Synthesis of 1,1-Dibromoalkenes **S8**

Synthesis of [3]Dendralenes by Twofold Cross-Coupling **S16**

Synthesis of Monobromodienes by *E*-Selective Single Cross-Coupling **S32**

Stereoselective Synthesis of Dendralenes **S44**

Triene-transmissive Twofold 6π-Electrocyclisation of [4]Dendralene **S66**

Diene-transmissive Twofold Diels–Alder Reactions of [3]Dendralene **S67**

X-Ray Crystallography **S69**

References **S72**

^{1}H and ^{13}C NMR Spectra **S73**

Analyses of the Widest Scope Substituted [3]Dendralene Syntheses

To place this work in context, an analysis of the best existing methods are provided here. These cutting-edge methods for substituted [3]dendralene synthesis serve to calibrate the value of the present contribution.

H. Wang, B. Beiring, D.-G. Yu, K. D. Collins, F. Glorius, *Angew. Chem. Int. Ed.* **2013**, *52*, 12430-12434: "[3]Dendralene Synthesis: Rhodium(III)-Catalyzed Alkenyl C-H Activation and Coupling Reaction with Allenyl Carbinol Carbonate":

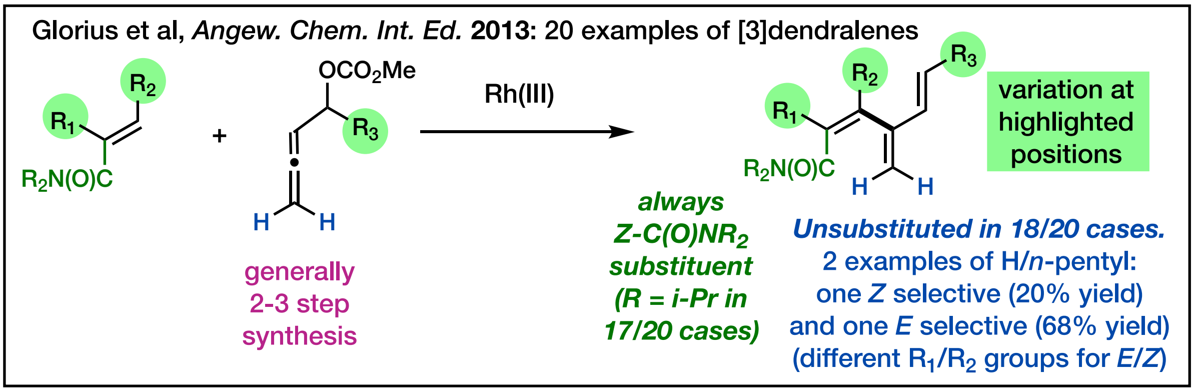

K. Sakashita, Y. Shibata, K. Tanaka, *Angew. Chem. Int. Ed.* **2016**, *55*, 6753-6757: "Rhodium-Catalyzed Cross-Cyclotrimerization and Dimerization of Allenes with Alkynes":

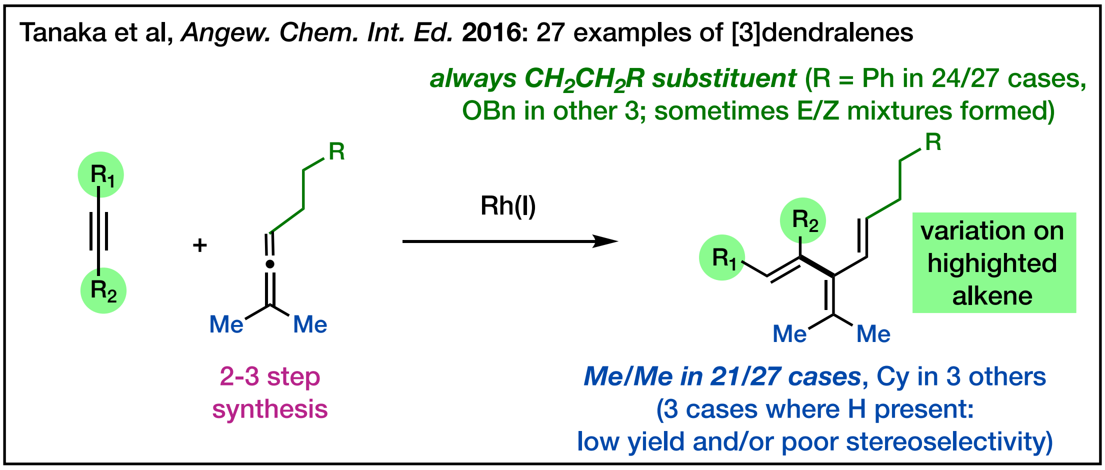

D. J. Lippincott, R. T. H. Linstadt, M. R. Maser, B. H. Lipshutz, *Angew. Chem. Int. Ed.* **2017**, *56*, 847-850: "Synthesis of Functionalized [3], [4], [5] and [6]Dendralenes through Palladium-Catalyzed Cross-Couplings of Substituted Allenoates":

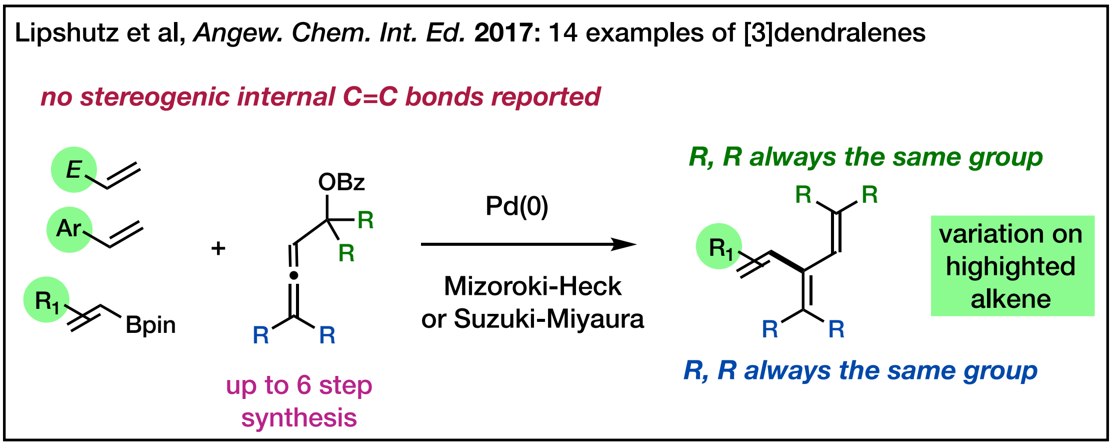

Y. Qiu, D. Posevins, J.-E. Baeckvall, *Angew. Chem. Int. Ed.* **2017**, *56*, 13112-13116: "Selective Palladium-Catalyzed Allenic C–H Bond Oxidation for the Synthesis of [3]Dendralenes":

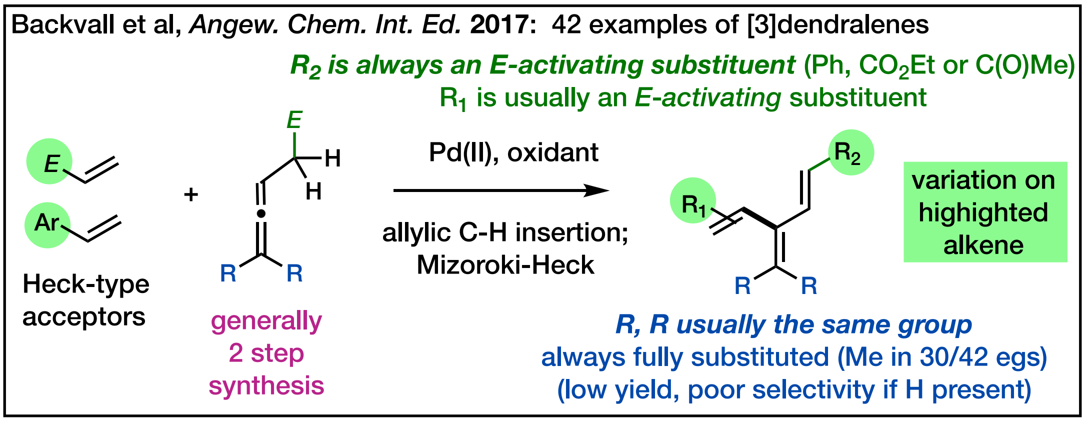

E. Rivera-Chao, M. Fananas-Mastral, *Angew. Chem. Int. Ed.* **2018**, *57*, 9945-9949: "Synthesis of Stereodefined Borylated Dendralenes through Copper-Catalyzed Allylboration of Alkynes":

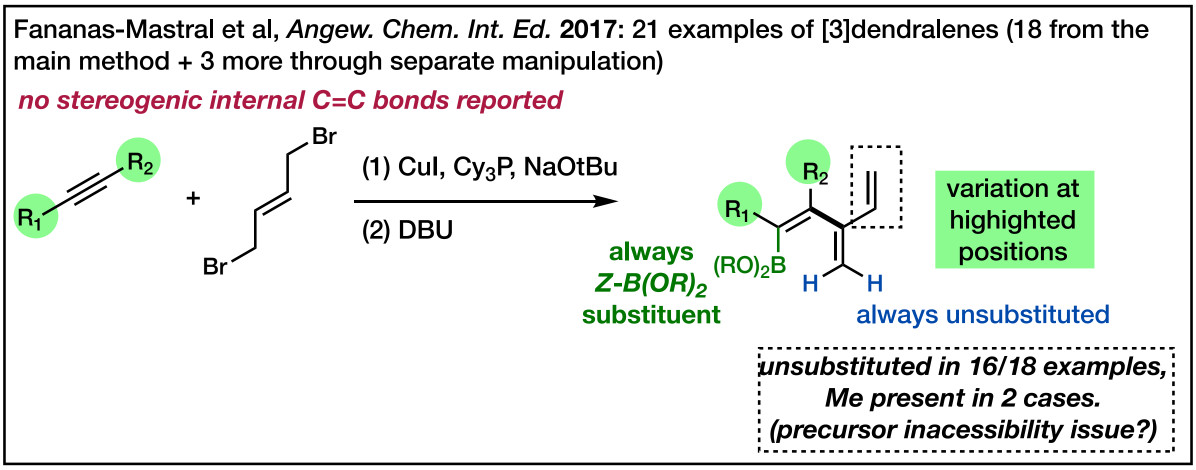

This work:

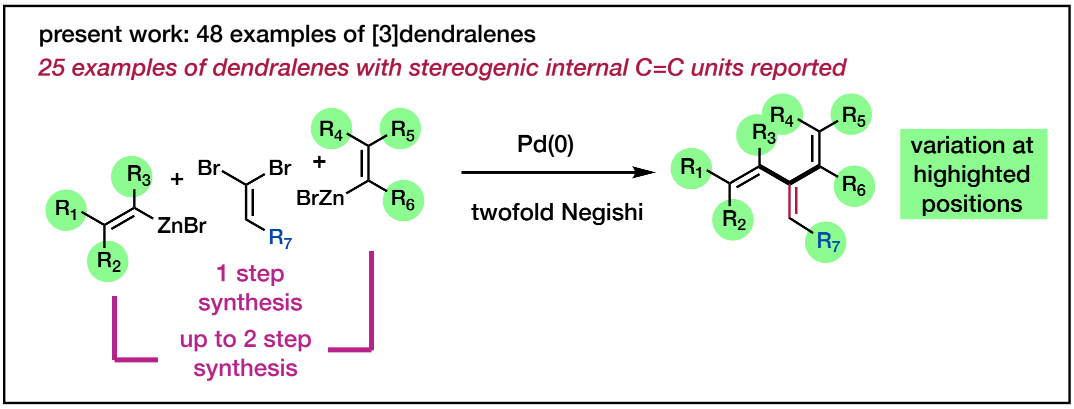

Additional Findings on Retention/Inversion Experiments

A stepwise inversion pathway was proposed (**Scheme S1**), driven by the minimization of steric strain in the initial oxidative insertion product **S1**. Thus, rearrangement through putative *cis*-alkenylidene-substituted π-allylpalladium complex **S2** to the σ-alkylpalladium complex **S3**, which after σ-bond rotation would generate the *trans*-alkenylidene-substituted π-allylpalladium complex **S4** (and/or σ-alkylpalladium complex **S5**). Evidently, for clean inversion, this process would have to be faster than transmetalation-reductive elimination of the initial oxidative insertion product **S1/S2**.

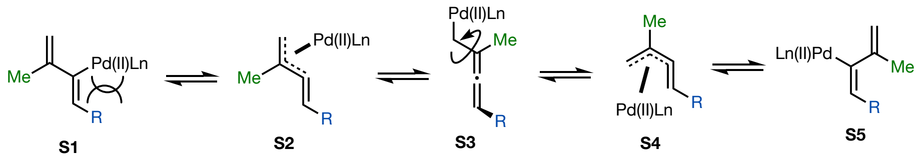

Scheme S1:

The Negishi group came to the following general conclusions regarding the inversion pathway: (a) it only works for alkyl aldehyde precursors, and (b) the reaction is successful irrespective of the nature of the nucleophile. Our results with alkenyl-zinc bromide nucleophiles (the Negishi work includes one example with vinyl-zinc bromide) are not fully consistent with these findings, in that the *para*-methoxybenzaldehyde-derived dibromoalkene works well (see main manuscript, Table 3, **4o-E/Z**). Additionally, in our hands several reactions of alkyl-substituted aldehydes gave mixtures of diastereomers with Pd(0)/*t*-Bu$_3$P (**Scheme S2**, these are referred to in the main manuscript in the paragraph above Table 3).

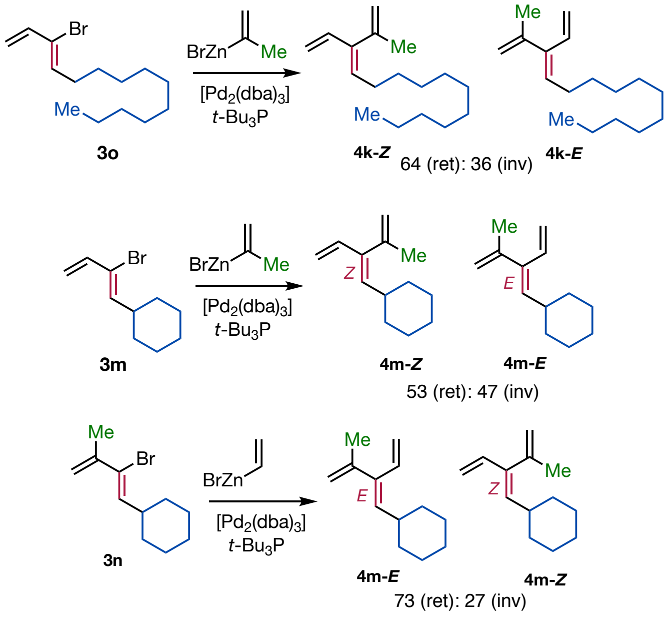

Scheme S2

Four other substrates gave clean retention of stereochemistry with [Pd(dppf)Cl_2] (**Scheme S3**)

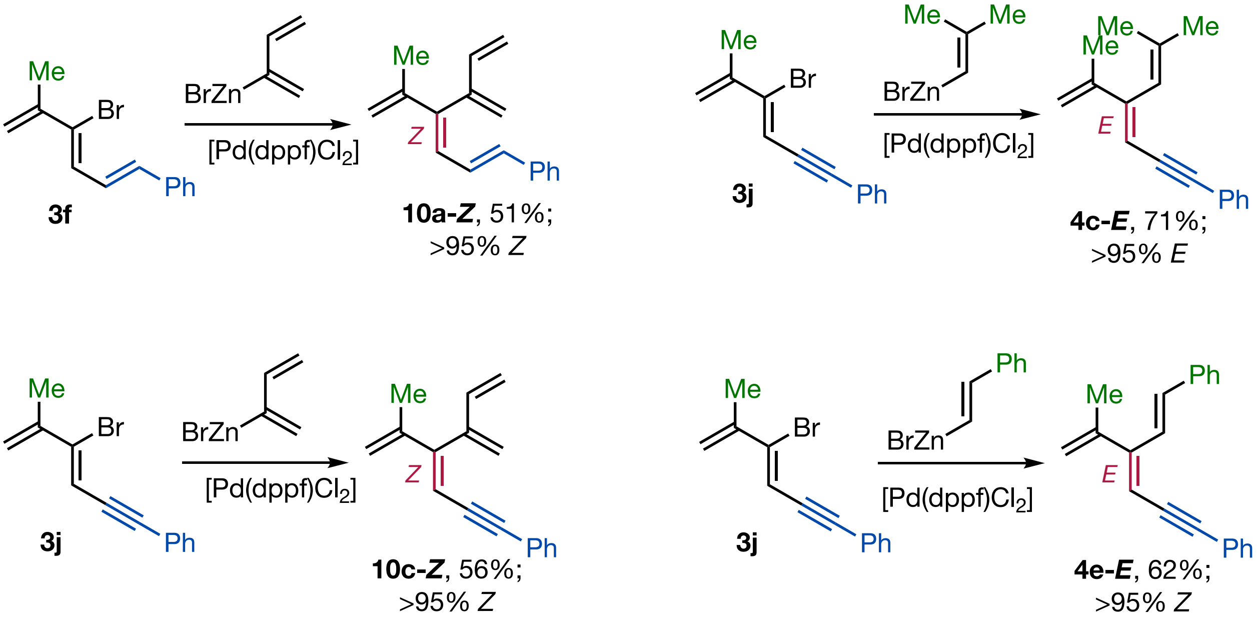

Scheme S3

General Methods

NMR Spectroscopy

^{1}H NMR spectra were recorded at 700, 600 and 400 MHz using a Bruker AVANCE 700, AVANCE 600, AVANCE 400 and Varian 400-MR spectrometer, as indicated. Residual solvent peaks were used as an internal reference for ^{1}H NMR spectra ($CDCl_3$ δ 7.26 ppm, CD_3OD δ 3.31 ppm, $(CD_3)_2SO$ δ 2.50 ppm, CD_3CN δ 1.94 ppm, CD_2Cl_2 δ 5.32 ppm, C_6D_6, δ 7.16 ppm). Coupling constants (*J*) are quoted to the nearest 0.1 Hz. The assignment of proton signals was assisted by COSY, HSQC and HMBC experiments. ^{13}C NMR spectra were recorded at 100 MHz and 175 MHz using a Bruker AVANCE 400 or Bruker AVANCE 700 spectrometer respectively. Solvent peaks were used as an internal reference for ^{13}C NMR spectra ($CDCl_3$ δ 77.16 ppm, CD_3OD δ 49.0 ppm, $(CD_3)_2SO$ δ 39.52 ppm, CD_3CN δ 1.32 ppm). Assignment of carbon signals was assisted by HSQC and HMBC experiments. NOE spectra were recorded at 400 MHz using Varian 400-MR spectrometer. The following abbreviations (or combinations thereof) are used to denote ^{1}H NMR multiplicities: s = singlet, d = doublet, dd = doublet of doublets, dt = doublet of triplet, t = triplet, m = multiplet.

Infrared Spectroscopy

IR spectra were recorded on a Perkin–Elmer UATR Two spectrometer as a thin film or solid.

Mass Spectrometry

Low-resolution EI mass spectra were recorded on a Finnigan Polaris Q ion trap mass spectrometer using electron impact (EI+) ionization mode at 70 eV. High-resolution EI mass spectra were recorded on a VG Autospec mass spectrometer operating at 70 eV. Low-resolution ESI mass spectra were recorded on a ZMD Micromass spectrometer with Waters Alliance 2690 HPLC. High-resolution ESI mass spectra were recorded on a Waters LCT Premier time-of-flight (TOF) mass spectrometer.

Melting Points

Melting points were measured on a Stanford Research Systems Optimelt Automated Melting Point System and are uncorrected.

X-ray Crystallography

Single crystal X-ray data was collected on a SuperNova (Dual Source) diffractometer using a SuperNova (Cu) X-ray radiation source. Crystallographic structures were solved using CryaAlis PRO.

Experimental Procedures, Reagents, Chromatography and Glassware

Reactions were conducted under a positive pressure of dry nitrogen in oven dried glassware and at room temperature, unless specified otherwise. Anhydrous solvents were either obtained from commercial sources or dried according to the procedure outlined by Grubbs and co-workers.[1] Commercially available chemicals were used as purchased, or where specified, purified by standard techniques. Analytical thin-layer chromatography was conducted with aluminum-backed silica gel 60 F_{254} (0.2 mm) plates supplied by Merck, and visualized using UV fluorescence (λmax = 254 nm), or developed using $KMnO_4$ or p-anisaldehyde or phosphomolybdic acid followed by heating. Flash chromatography employed Merck Kiesegel 60 silica gel (230–400 mesh). $AgNO_3$ coated silica TLC plates were prepared according to the procedure by Caspi and co-workers.[2] $AgNO_3$ impregnated silica gel was prepared as per the reported procedure by Mizaikoff and co-workers.[3] Solvent compositions are given in (v/v). PS 40–60 °C refers to petroleum spirits, boiling point fraction 40–60 °C. Grignard regents were prepared from the corresponding halides as per the general procedure by Cook and co-workers.[4]. Grignard reagent concentration was determined by the method detailed by Love and Jones.[5] $Pd_2(dba)_3{\cdot}CHCl_3$ was prepared according the procedure reported by Ananikov and co-workers.[6] $PdCl_2(dppf){\cdot}$toluene was prepared according to a procedure reported by Brandsma and co-workers.[7]

Synthesis of 1,1-Dibromoalkenes

(2,2-Dibromovinyl)benzene (1a)

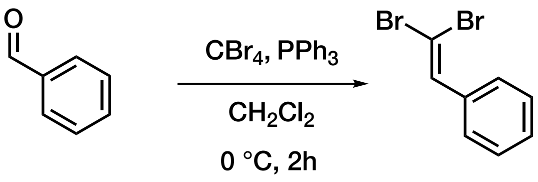

1a

(2,2-Dibromovinyl)benzene **1a** was prepared according to the reported procedure by Kim and co-workers from benzaldehyde (4.00 g, 37.7 mmol) as a colourless oil (7.12 g, 27.2 mmol, 72%) and the characterization data is consistent with the literature.[8] R_f = 0.63 (5% EtOAc in PS 40–60);

^{1}H NMR (400 MHz, $CDCl_3$) δ 7.58 – 7.51 (m, 2H), 7.49 (s, 1H), 7.41 – 7.31 (m, 3H) ppm; ^{13}C NMR (100 MHz, $CDCl_3$) δ 137.0 (CH), 135.5 (Cq), 128.7 (CH), 128.6 (2×CH), 128.5 (2×CH), 89.8 (Cq) ppm; IR (thin film): ν_{max} = 3056, 3023, 1948, 1885, 1595, 1493, 1444 cm^{-1}; LRMS: (EI+): m/z (%): 264 ([M(^{81}Br$_2$)]$^{+•}$, 59), 262 ([M(^{81}Br^{79}Br)]$^{+•}$, 100), 260 ([M(^{79}Br$_2$)]$^{+•}$, 53), 102 ([M–Br$_2$)]$^{+•}$, 100); HRMS (EI+): calculated for $C_8H_6{}^{81}Br_2$: 263.8795; found: 263.8796; calculated for $C_8H_6{}^{79}Br^{81}Br$: 261.8816; found: 261.8817; calculated for $C_8H_6{}^{79}Br_2$: 259.8836; found: 259.8839.

1-(2,2-Dibromovinyl)-4-methylbenzene (1b)

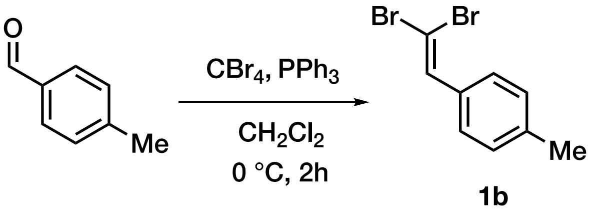

1b

1-(2,2-Dibromovinyl)-4-methylbenzene **1b** was prepared according to the reported procedure by Bolm and co-workers from 4-methylbenzaldehyde (5.00 g, 41.6 mmol) as a colourless oil (9.85 g, 35.7 mmol, 86%) and the characterization data is consistent with the literature.[8-9] R$_f$ = 0.63 (5% EtOAc in PS 40–60); ^{1}H NMR (400 MHz, $CDCl_3$) δ 7.47 – 7.42 (m, 3H), 7.18 (d, J = 8.0 Hz, 2H), 2.35 (s, 3H) ppm; ^{13}C NMR (100 MHz, $CDCl_3$) δ 138.8 (Cq), 136.9 (CH), 132.5 (Cq), 129.0 (2×CH), 128.5 (2×CH), 88.73 (Cq), 21.5 (CH_3) ppm; IR (thin film): ν_{max} = 3023, 2918, 1905, 1610, 1598, 1508 cm^{-1}; LRMS: (EI+): m/z (%): 278 ([M(^{81}Br$_2$)]$^{+•}$, 52), 276 ([M(^{81}Br^{79}Br)]$^{+•}$, 100), 274 ([M(^{79}Br$_2$)]$^{+•}$, 52), 197 ([M(^{81}Br$_2$)–^{81}Br]$^{+•}$, 15), 195 ([M(^{79}Br$_2$)–^{79}Br]$^{+•}$, 14), 116 ([M–Br$_2$]$^{+•}$, 76); HRMS (EI+): calculated for $C_9H_8{}^{81}Br_2$: 277.8952; found: 277.8959; calculated for $C_9H_8{}^{81}Br^{79}Br$: 275.8972; found: 275.8976; calculated for $C_9H_8{}^{79}Br_2$: 273.8993; found: 273.8995.

1-(2,2-Dibromovinyl)-4-methoxybenzene (1c)

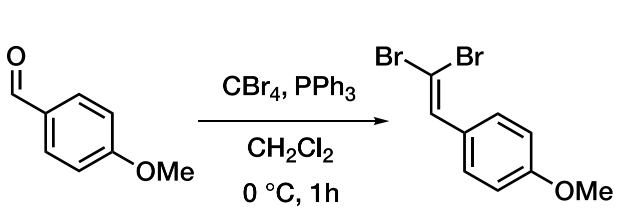

1c

1-(2,2-Dibromovinyl)-4-methoxybenzene **1c** was prepared according to the reported procedure by Bach and co-workers from 4-methoxybenzaldehyde (5.00 g, 36.7 mmol) as an off white solid (9.35 g, 32.0 mmol, 87%) and the characterization data is consistent with the literature.[10] R$_f$ = 0.51

(10% EtOAc in PS 40–60); m.p. 36 – 38 °C (CH_2Cl_2); ^{1}H NMR (400 MHz, $CDCl_3$) δ 7.51 (d, J = 8.9 Hz, 2H), 7.41 (s, 1H), 6.89 (d, J = 8.9 Hz, 2H), 3.82 (s, 3H) ppm; ^{13}C NMR (100 MHz, $CDCl_3$) δ 159.8 (Cq), 136.4 (CH), 130.0 (2×CH), 128.0 (Cq), 113.9 (2×CH), 87.44 (Cq), 55.5 (CH_3) ppm; IR (thin film): ν_{max} = 3005, 2956, 2835, 1605, 1508 cm^{-1}; LRMS: (EI+): m/z (%): 294 ([M($^{81}Br_2$)]$^{+\bullet}$, 29), 292 ([M($^{81}Br^{79}Br$)]$^{+\bullet}$, 100), 290 ([M($^{79}Br_2$)]$^{+\bullet}$, 50), 277 ([M($^{81}Br^{79}Br$)–CH_3)]$^{+\bullet}$, 29), 132 ([M–Br_2)]$^{+\bullet}$, 45); HRMS (EI+): calculated for $C_9H_8O^{81}Br_2$: 293.8901; found: 293.8902; calculated for $C_9H_8O^{79}Br^{81}Br$: 291.8921; found: 291.8916; calculated for $C_9H_8O^{79}Br_2$: 289.8942; found: 289.8934.

5-(2,2-Dibromovinyl)-1,2,3-trimethoxybenzene (1d)

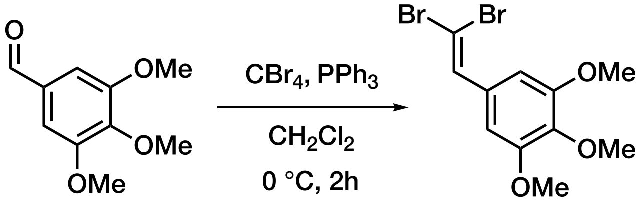

1d

5-(2,2-Dibromovinyl)-1,2,3-trimethoxybenzene **1d** was prepared according to the reported procedure by Doddi and co-workers from 3,4,5-trimethoxybenzaldehyde (5.00 g, 25.5 mmol) as a pale brown solid (7.80 g, 22.2 mmol, 87%) and the characterization data is consistent with the literature.[11] R_f = 0.45 (20% EtOAc in PS 40–60); m.p. 48 – 51 °C (CH_2Cl_2); ^{1}H NMR (400 MHz, $CDCl_3$) δ 7.41 (s, 1H), 6.80 (s, 2H), 3.87 (s, 3H), 3.86 (s, 6H) ppm; ^{13}C NMR (100 MHz, $CDCl_3$) δ 153.0 (2×Cq), 138.4 (Cq), 136.6 (CH), 130.5 (Cq), 105.8 (2×CH), 88.7 (Cq), 60.8 (CH_3), 56.2 (2×CH_3) ppm; IR (thin film): ν_{max} = 2997, 2936, 2830, 1576, 1506 cm^{-1}; LRMS: (EI+): m/z (%): 354 ([M($^{81}Br_2$)]$^{+\bullet}$, 53), 352 ([M($^{81}Br^{79}Br$)]$^{+\bullet}$, 100), 350 ([M($^{79}Br_2$)]$^{+\bullet}$, 50), 339 ([M($^{81}Br_2$)–CH_3]$^{+\bullet}$, 43), 337 ([M($^{81}Br^{79}Br$)–CH_3]$^{+\bullet}$, 43), 335 ([M($^{79}Br_2$)–CH_3]$^{+\bullet}$, 43), 177 ([M–CH_3Br_2]$^{+\bullet}$, 28); HRMS (EI+): calculated for $C_{11}H_{12}O_3{}^{81}Br_2$: 353.9112; found: 353.9120; calculated for $C_{11}H_{12}O_3{}^{79}Br^{81}Br$: 351.9133; found: 351.9132; calculated for $C_{11}H_{12}O_3{}^{79}Br_2$: 349.9153; found: 349.9153.

1-(2,2-Dibromovinyl)-4-nitrobenzene (1e)

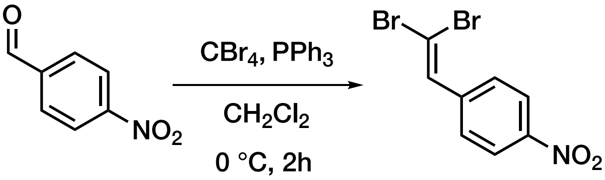

1e

1-(2,2-Dibromovinyl)-4-nitrobenzene **1e** was prepared according to the reported procedure by Doddi and co-workers from 4-nitrobenzaldehyde (3.00 g, 19.9 mmol) as a yellow solid (5.12 g, 16.7 mmol, 84%) and the characterization data is consistent with the literature.[8-9, 11a, 12] R_f = 0.45 (10% EtOAc in PS 40–60); m.p. 104 – 106 °C (CH_2Cl_2); ^{1}H NMR (400 MHz, $CDCl_3$) δ 8.23 (d, J = 8.8 Hz, 2H), 7.70 (d, J = 8.8 Hz, 2H), 7.56 (s, 1H) ppm; ^{13}C NMR (100 MHz, $CDCl_3$) δ 147.4 (Cq), 141.6 (Cq), 135.1 (CH), 129.3 (2×CH), 123.9 (2×CH), 94.3 (Cq) ppm; IR (thin film): ν_{max} = 3102, 3077, 2443, 1929, 1589, 1506 cm^{-1}; LRMS: (EI+): m/z (%): 309 ([M(2×^{81}Br)]$^{+\bullet}$, 50), 307 ([M(^{81}Br^{79}Br)]$^{+\bullet}$, 100), 305 ([M(2×^{79}Br)]$^{+\bullet}$, 50); HRMS (EI+): calculated for $C_8H_5NO_2{}^{81}Br_2$: 308.8646; found: 308.8639; calculated for $C_8H_5NO_2{}^{81}Br^{79}Br$: 306.8667; found: 306.8657; calculated for $C_8H_5NO_2{}^{79}Br_2$: 304.8687; found: 304.8688.

1,1-Dibromohept-1-ene (1f)

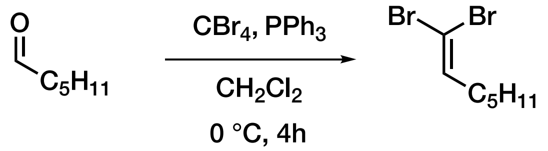

1f

Adapted from the literature procedure by Ramirez and co-workers.[13] To a stirred solution of Hexanal (1.00 g, 9.98 mmol) and PPh_3 (6.28 g, 24.0 mmol) in CH_2Cl_2 (40 mL) at 0 °C was added a solution of CBr_4 (3.97 g, 12.0 mmol) in CH_2Cl_2 (10 mL) over 10 minutes. After stirring at 0 °C for 4h, TLC analysis showed under reduced pressure and the resulting slurry was stirred with 50 mL petroleum ether for 30 minutes and then filtered through a pad of Celite. The filtrate was concentrated under reduced pressure to give the dibromide **1f** as a colourless oil (1.26 g, 4.92 mmol, 49%). R_f = 0.61 (100% PS 40–60); ^{1}H NMR (400 MHz, $CDCl_3$) δ 6.39 (t, J = 7.2 Hz, 1H), 2.09 (q, J = 7.2 Hz, 2H), 1.49 – 1.20 (m, 6H), 0.90 (t, J = 6.6 Hz, 3H) ppm; ^{13}C NMR (100 MHz, $CDCl_3$) δ 139.1 (CH), 88.6 (Cq), 33.1 (CH_2), 31.4 (CH_2), 27.6 (CH_2), 22.6 (CH_2), 14.1 (CH_3) ppm;
IR (thin film): ν_{max} = 2956, 2926, 2857, 1621 cm^{-1}; LRMS: (EI+): m/z (%): 258 ([M(^{81}Br$_2$)]$^{+\bullet}$, 40), 256 ([M(^{81}Br^{79}Br)]$^{+\bullet}$, 35), 254 ([M(^{79}Br$_2$)]$^{+\bullet}$, 30), 198 ([M(^{81}Br^{79}Br)–C_4H_{10}]$^{+\bullet}$, 50), 95 (100); HRMS (EI+): calculated for $C_7H_{12}{}^{81}Br_2$: 257.9265; found: 257.9268; calculated for $C_7H_{12}{}^{81}Br^{79}Br$: 255.9285; found: 255.9285; calculated for $C_7H_{12}{}^{79}Br_2$: 253.9306; found: 253.9302.

1,1-Dibromododec-1-ene (1g)

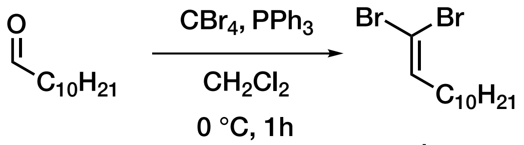

1g

Adapted from the literature procedure by Ramirez and co-workers.[13] To a stirred solution of undecanal (5.00 g, 29.4 mmol) and PPh_3 (18.5 g, 70.5 mmol) in CH_2Cl_2 (200 mL) at 0 °C was added a solution of CBr_4 (11.7 g, 35.2 mmol) in CH_2Cl_2 (50 mL) over 10 minutes. After stirring at 0 °C for 1h, TLC analysis showed reaction completion. CH_2Cl_2 was removed under reduced pressure and the resulting slurry was stirred with 250 mL PS 40–60 for 30 minutes and filtered through a pad of Celite. The filtrate was concentrated under reduced pressure to give the dibromide **1g** as a colourless oil (4.02 g, 12.3 mmol, 42%). R_f = 0.65 (100% PS 40–60); ^{1}H NMR (400 MHz, $CDCl_3$) δ 6.39 (t, J = 7.3 Hz, 1H), 2.09 (q, J = 7.3 Hz, 2H), 1.51 – 1.17 (m, 16H), 0.88 (t, J = 6.8 Hz, 3H) ppm; ^{13}C NMR (100 MHz, $CDCl_3$) δ 139.1 (CH), 88.6 (Cq), 33.2 (CH_2), 32.1 (CH_2), 29.7 (CH_2), 29.7 (CH_2), 29.5 (CH_2), 29.5 (CH_2), 29.2 (CH_2), 28.0 (CH_2), 22.9 (CH_2), 14.3 (CH_3) ppm; IR (thin film): ν_{max} = 2922, 2853, 1623, 1464 cm^{-1}; LRMS: (EI+): m/z (%): 328 ([M($^{81}Br_2$)]$^{+\bullet}$, 3), 326 ([M($^{81}Br^{79}Br$)]$^{+\bullet}$, 8), 324 ([M($^{79}Br_2$)]$^{+\bullet}$, 3), 201 ([M($^{81}Br_2$)–C_9H_{19}]$^{+\bullet}$, 26), 199 ([M($^{81}Br^{79}Br$)–C_9H_{19}]$^{+\bullet}$, 63), 197 ([M($^{79}Br_2$)–C_9H_{19}]$^{+\bullet}$, 26), 95 (100); HRMS (EI+): calculated for $C_{12}H_{22}{}^{81}Br_2$: 328.0047; found: 328.0049; calculated for $C_{12}H_{22}{}^{81}Br^{79}Br$: 326.0068; found: 326.0071; calculated for $C_{12}H_{22}{}^{79}Br_2$: 324.0088; found: 324.0092.

(2,2-Dibromovinyl)cyclohexane (1h)

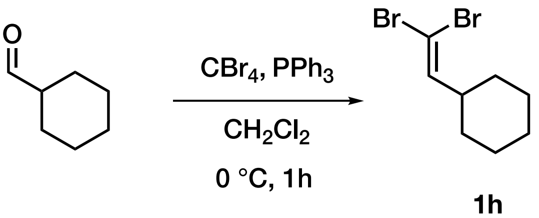

1h

(2,2-Dibromovinyl)cyclohexane **1h** was prepared according to the reported procedure by Hosomi and co-workers from cyclohexane carbaldehyde (3.00 g, 26.7 mmol) as a colourless liquid (6.15 g, 22.9 mmol, 86%) and the characterization data is consistent with the literature.[14] R_f = 0.56 (100% PS 40–60); ^{1}H NMR (400 MHz, $CDCl_3$) δ 6.23 (d, J = 9.2 Hz, 1H), 2.44 – 2.10 (m, 1H), 1.89 – 1.57 (m, 5H), 1.44 – 0.86 (m, 5H) ppm; ^{13}C NMR (100 MHz, $CDCl_3$) δ 143.9 (CH), 87.1 (Cq), 42.6 (CH), 31.4 (2×CH_2), 25.9 (CH_2), 25.6 (2×CH_2) ppm; IR (thin film): ν_{max} = 2923, 2850, 1603, 1447 cm^{-1}; LRMS: (EI+): m/z (%): 270 ([M($^{81}Br_2$)]$^{+\bullet}$, 42), 268 ([M($^{81}Br^{79}Br$)]$^{+\bullet}$, 100),

266 ([M($^{79}Br_2$)]$^{+•}$, 58), 82 ([M–$C_2H_2Br_2$]$^{+•}$, 100),; HRMS (EI+): calculated for $C_8H_{12}{}^{81}Br_2$: 269.9265; found: 269.9272; calculated for $C_8H_{12}{}^{79}Br^{81}Br$: 267.9285; found: 267.9297; calculated for $C_8H_{12}{}^{79}Br_2$: 265.9306; found: 265.9313.

(4,4-Dibromobut-3-en-1-yl)benzene (1i)

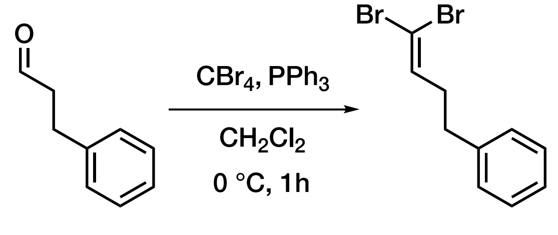

1i

(4,4-Dibromobut-3-en-1-yl)benzene **1i** was prepared according to the reported procedure by Doi and co-workers from 3-phenylpropanal (5.00 g, 37.3 mmol) as a pale brown oil (7.62 g, 26.3 mmol, 70%).[11a, 15] R_f = 0.63 (5% EtOAc in PS 40–60); ^{1}H NMR (400 MHz, $CDCl_3$) δ 7.34 – 7.27 (m, 2H), 7.25 – 7.16 (m, 3H), 6.42 (t, J = 7.2 Hz, 1H), 2.74 (t, J = 7.7 Hz, 2H), 2.42 (q, J = 7.2 Hz, 2H) ppm; ^{13}C NMR (100 MHz, $CDCl_3$) δ 140.7 (Cq), 137.8 (CH), 128.7 (2×CH), 128.5 (2×CH), 126.4 (CH), 89.6 (Cq), 34.8 (CH_2), 34.0 (CH_2) ppm; IR (thin film): ν_{max} = 3026, 2924, 2857, 1782, 1603, 1495 cm^{-1}; LRMS: (EI+): m/z (%): 292 ([M($^{81}Br_2$)]$^{+•}$, 1), 290 ([M($^{81}Br^{79}Br$)]$^{+•}$, 2), 288 ([M($^{79}Br_2$)]$^{+•}$, 1), 211 ([M($^{81}Br^{79}Br$)–^{79}Br]$^{+•}$, 33), 209 ([M($^{81}Br^{79}Br$)–^{81}Br]$^{+•}$, 33), 91 ([M–$C_3H_3Br_2$]$^{+•}$, 100); HRMS (EI+): calculated for $C_{10}H_{10}{}^{81}Br_2$: 291.9108; found: 291.9104; calculated for $C_{10}H_{10}{}^{81}Br^{79}Br$: 289.9129; found: 289.9120; calculated for $C_{10}H_{10}{}^{79}Br_2$: 287.9149; found: 287.9148.

(*E*)-(4,4-Dibromobuta-1,3-dien-1-yl)benzene (1j)

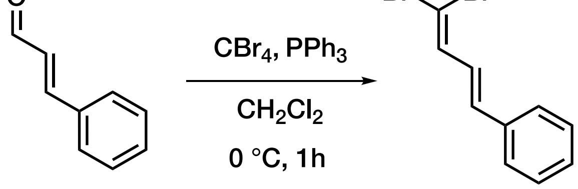

1j

(*E*)-(4,4-Dibromobuta-1,3-dien-1-yl)benzene **1j** was prepared according to the reported procedure by Doddi and co-workers from *trans*-cinnamaldehyde (5.00 g, 37.8 mmol) as a white solid (8.94 g, 31.0 mmol, 82%) and the characterization data is consistent with the literature.[9, 11a, 16] R_f= 0.60 (5% EtOAc in PS 40–60); m.p. 50 – 52 °C (CH_2Cl_2); ^{1}H NMR (400 MHz, $CDCl_3$) δ 7.52 – 7.42 (m, 2H), 7.38 – 7.27 (m, 3H), 7.10 (d, J = 9.6 Hz, 1H), 6.86 – 6.65 (m, 2H) ppm;

^{13}C NMR (100 MHz, $CDCl_3$) δ 137.3 (CH), 136.5 (Cq), 135.8 (CH), 128.9 (2×CH), 128.8 (CH), 127.0 (2×CH), 125.4 (CH), 91.4 (Cq) ppm; IR (thin film): ν_{max} = 3030, 3015, 1704, 1557, 1484 cm^{-1}; LRMS: (EI+): m/z (%): 290 ($[M(^{81}Br_2)]^{+\bullet}$, 35), 288 ($[M(^{81}Br^{79}Br)]^{+\bullet}$, 68), 286 ($[M(^{79}Br_2)]^{+\bullet}$, 37), 128 ($[M–Br_2)]^{+\bullet}$, 100); HRMS (EI+): calculated for $C_{10}H_8{}^{81}Br_2$: 289.8952; found: 289.8965; calculated for $C_{10}H_8{}^{81}Br^{79}Br$: 287.8972; found: 287.8979; calculated for $C_{10}H_8{}^{79}Br_2$: 285.8993; found: 285.8991.

(4,4-Dibromobut-3-en-1-yn-1-yl)benzene (1k)

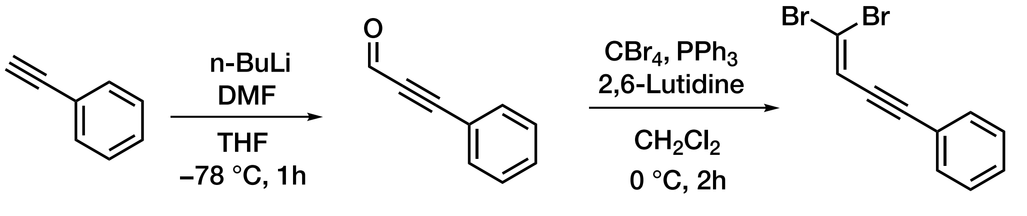

1k

(4,4-Dibromobut-3-en-1-yn-1-yl)benzene **1k** was prepared according to the reported procedure by Negishi and co-workers from phenylacetylene (2.00 g, 19.6 mmol) as a colourless oil (3.55 g, 12.4 mmol, 63% from phenylacetylene) and the characterization data is consistent with the literature.[17] R_f = 0.60 (5% EtOAc in PS 40–60); ^1H NMR (400 MHz, $CDCl_3$) δ 7.53 – 7.47 (m, 2H), 7.39 – 7.30 (m, 3H), 6.78 (s, 1H) ppm; ^{13}C NMR (100 MHz, $CDCl_3$) δ 131.7 (2×CH), 129.2 (CH), 128.6 (2×CH), 122.6 (Cq), 119.8 (CH), 101.9 (Cq), 97.3 (Cq), 86.3 (Cq) ppm; IR (thin film): ν_{max} = 3054, 3017, 2200, 1570, 1487 cm^{-1}; LRMS: (EI+): m/z (%): 288 ($[M(^{81}Br_2)]^{+\bullet}$, 39), 286 ($[M(^{81}Br^{79}Br)]^{+\bullet}$, 71), 284 ($[M(^{79}Br_2)]^{+\bullet}$, 39), 126 ($[M–Br_2)]^{+\bullet}$, 100); HRMS (EI+): calculated for $C_{10}H_6{}^{81}Br_2$: 287.8795; found: 287.8809; calculated for $C_{10}H_6{}^{81}Br^{79}Br$: 285.8816; found: 285.8822; calculated for $C_{10}H_6{}^{79}Br_2$: 283.8836; found: 283.8832.

3-(2,2-Dibromovinyl)-1-tosyl-1H-indole (1l)

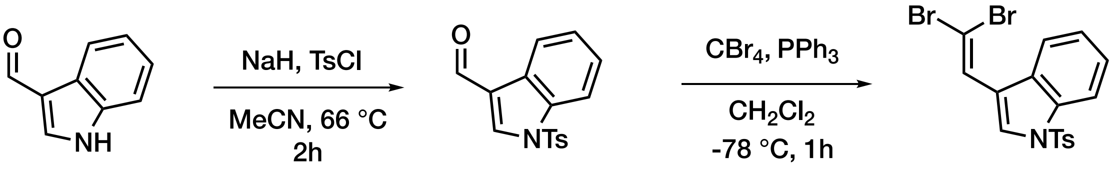

1l

3-(2,2-Dibromovinyl)-1-tosyl-1H-indole **1l** was prepared over two steps from indole-3-carboxaldehyde.according to the reported procedure by Katsumura and co-workers from indole-3-carboxaldehyde (2.00 g, 13.8 mmol) as a white solid (4.52 g, 9.93 mmol, 72% over two steps) and the data is consistent with the literature.[18] [19]R_f = 0.56 (30% EtOAc in PS 40–60);

m.p. 129 – 132 °C (CH_2Cl_2); ^{1}H NMR (400 MHz, $CDCl_3$) δ 8.29 (s, 1H), 7.99 (d, J = 8.3 Hz, 1H), 7.79 (d, J = 8.4 Hz, 2H), 7.59 – 7.47 (m, 2H), 7.36 (t, J = 7.6 Hz, 1H), 7.29 (d, J = 8.0 Hz, 1H), 7.24 (d, J = 8.4 Hz, 2H), 2.35 (s, 3H) ppm; ^{13}C NMR (100 MHz, $CDCl_3$) δ 145.5 (Cq), 135.1 (Cq), 134.2 (Cq), 130.2 (2×CH), 129.6 (Cq), 127.1 (2×CH), 127.0 (CH), 125.6 (CH), 125.1 (CH), 123.8 (CH), 119.0 (CH), 117.5 (Cq), 113.8 (CH), 90.5 (Cq), 21.8 (CH_3) ppm; IR (thin film): ν_{max} = 3157, 2923, 1592, 1537, 1446 cm^{-1}; LRMS: (EI+): m/z (%): 457 ([M($^{81}Br_2$)]$^{+\bullet}$, 43), 455 ([M($^{81}Br^{79}Br$)]$^{+\bullet}$, 70), 453 ([M($^{79}Br_2$)]$^{+\bullet}$, 43), 140 ([M–$C_7H_7O_2SBr_2$)]$^{+\bullet}$, 70); HRMS (EI+): calculated for $C_{17}H_{13}NO_2S^{81}Br_2$: 456.8993; found: 456.8992; calculated for $C_{17}H_{13}NO_2S^{79}Br^{81}Br$: 454.9013; found: 454.9016; calculated for $C_{17}H_{13}NO_2S\ ^{79}Br_2$: 452.9034; found: 452.9039.

2-(2,2-Dibromovinyl)-5-methylfuran (1m)

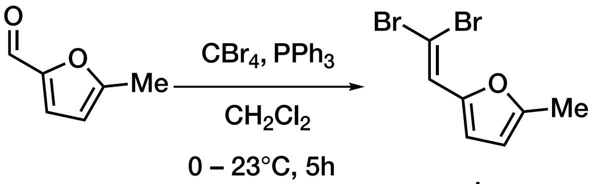

1m

2-(2,2-Dibromovinyl)-5-methylfuran **1m** was prepared according to the reported procedure by Mikami and co-workers from 5-methylfuraldehyde (2.60 g, 23.6 mmol) as a colourless liquid (4.53 g, 17.0 mmol, 72%) and the characterization data is consistent with the literature.[20] R_f = 0.42 (100% PS 40–60); ^{1}H NMR (400 MHz, $CDCl_3$) δ 7.32 (s, 1H), 6.83 (d, J = 2.8 Hz, 1H), 6.05 (d, J = 2.8 Hz, 1H), 2.28 (s, 3H) ppm; ^{13}C NMR (100 MHz, $CDCl_3$) δ 152.9 (Cq), 148.6 (Cq), 126.6 (CH), 112.9 (CH), 108.1 (CH), 85.4 (Cq), 13.8 (CH_3) ppm; IR (thin film): ν_{max} = 3028, 2921, 1586, 1520 cm^{-1}; LRMS: (EI+): m/z (%): 268 ([M($^{81}Br_2$)]$^{+\bullet}$, 50), 266 ([M($^{81}Br^{79}Br$)]$^{+\bullet}$, 100), 264 ([M($^{79}Br_2$)]$^{+\bullet}$, 50), 106 ([M–Br_2]$^{+\bullet}$, 50); HRMS (EI+): calculated for $C_7H_6O^{81}Br_2$: 267.8744; found: 267.8741; calculated for $C_7H_6O^{81}Br^{79}Br$: 265.8765; found: 265.8759; calculated for $C_7H_6O^{79}Br_2$: 263.8785; found: 263.8785.

Synthesis of [3]Dendralenes by Twofold Cross-Coupling

Standard Procedure A

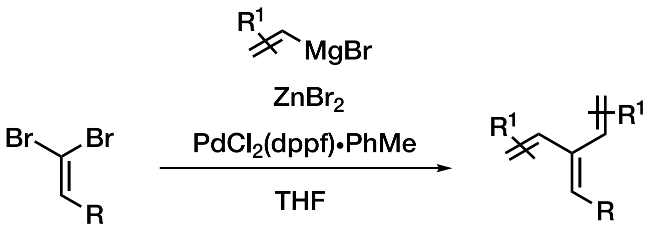

A solution of 1,1'-bis(diphenylphosphino)ferrocene palladium(II) chloride•toluene (5.0 mol %) and the dibromo olefin (1.0 mol equiv) in THF (50 mL/g dibromide precursor) was purged with N_2 for 5 minutes. After cooling to 0 °C, $ZnBr_2$ (4.0 mol equiv, 1.10 – 1.25 M solution in THF) was added, followed by dropwise addition of the Grignard reagent (3.0 mol equiv, 0.40 – 0.85 M solution in THF) over 15 minutes. The resulting heterogeneous reaction mixture was brought to room temperature and stirred for 16h, until the reaction was complete by ^{1}H NMR spectroscopic analysis. Saturated aqueous NH_4Cl (5 mL/g dibromide precursor) was added, the reaction mixture was filtered through Celite then the filtrate was diluted with CH_2Cl_2 (50 mL/g dibromide precursor) and water (100 mL/g dibromide precursor). The aqueous and organic layers were separated and the aqueous layer was extracted with CH_2Cl_2 (10 mL/g dibromide precursor × 3). The combined organic layers were washed with saturated brine (20 mL/g dibromide precursor), dried over Na_2SO_4 and concentrated under reduced pressure.

(2-Vinylbuta-1,3-dien-1-yl)benzene (2a)

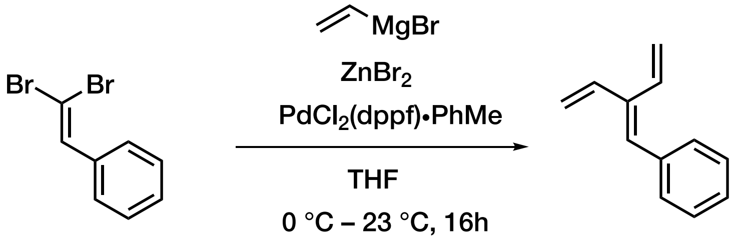

1a **2a**

Following **Standard Procedure A**, the reaction mixture containing dibromide **1a** (3.40 g, 13.0 mmol), 1,1'-bis(diphenylphosphino)ferrocene palladium(II) chloride•toluene (0.535 g, 0.649 mmol), $ZnBr_2$ solution in THF (47.2 mL, 1.10 M, 51.9 mmol) and vinyl magnesium bromide (32.5 mL, 1.20 M, 38.9 mmol) in THF (170 mL) was stirred at 23 °C for 16h. After work up, purification by flash column chromatography (120 g SiO_2, PS 40–60) gave dendralene **2a** as a colourless liquid (1.48 g, 9.47 mmol, 73%). R_f =0.48 (100% PS 40–60); ^{1}H NMR (400 MHz, $CDCl_3$) δ 7.42 – 7.30

(m, 4H), 7.28 – 7.20 (m, 1H), 6.71 (dd, J = 17.8, 11.1 Hz, 1H), 6.65 (s, 1H), 6.56 (dd, J = 17.2, 10.7 Hz, 1H), 5.55 (d, J = 17.2 Hz, 1H), 5.46 (d, J = 17.7 Hz, 1H), 5.36 (d, J = 11.1 Hz, 1H), 5.22 (d, J = 10.9 Hz, 1H) ppm; ^{13}C NMR (100 MHz, $CDCl_3$) δ 138.1 (Cq), 138.0 (CH), 137.2 (Cq), 133.7 (CH), 129.8 (2×CH), 129.7 (CH), 128.2 (2×CH), 127.3 (CH), 118.5 (CH_2), 116.2 (CH_2) ppm; IR (thin film): ν_{max} = 3085, 3007, 2955, 1830, 1605, 1491 cm^{-1}; LRMS: (EI+): m/z (%): 156 ($[M]^{+\bullet}$, 66), 141 ($[M–CH_3]^{+\bullet}$, 86), 128 ($[M–2×CH_2]^{+\bullet}$, 100), 115 ($[M–C_3H_5]^{+\bullet}$, 66); HRMS (EI+): calculated for $C_{12}H_{12}$: 156.0939; found: 156.0939.

1-Methyl-4-(2-vinylbuta-1,3-dien-1-yl)benzene (2b)

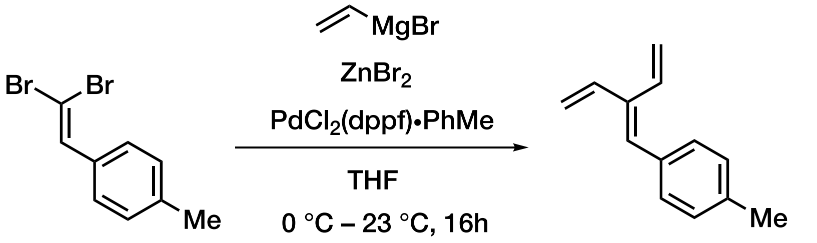

1b **2b**

Following **Standard Procedure A**, the reaction mixture containing dibromide **1b** (1.04 g, 3.77 mmol), 1,1'-bis(diphenylphosphino)ferrocene palladium(II) chloride•toluene (132 mg, 0.181 mmol), $ZnBr_2$ solution in THF (11.2 mL, 1.29 M, 14.5 mmol) and vinyl magnesium bromide (16.9 mL, 0.64 M, 10.9 mmol) in THF (50 mL) was stirred at 23 °C for 16h. After work up, purification by flash column chromatography (40 g SiO_2, PS 40–60) gave dendralene **2b** as a colourless liquid (0.46 g, 2.68 mmol, 71%). R_f = 0.44 (100% PS 40–60); ^1H NMR (400 MHz, $CDCl_3$) δ 7.27 (d, J = 8.0 Hz, 2H), 7.14 (d, J = 8.0 Hz, 2H), 6.71 (dd, J = 17.8, 11.1 Hz, 1H), 6.62 (s, 1H), 6.55 (dd, J = 17.3, 10.7 Hz, 1H), 5.52 (dd, J = 17.2, 1.5 Hz, 1H), 5.44 (dd, J = 17.7, 1.2 Hz, 1H), 5.34 (d, J = 11.1 Hz, 1H), 5.19 (dd, J = 10.7, 1.6 Hz, 1H), 2.36 (s, 3H) ppm; ^{13}C NMR (100 MHz, $CDCl_3$) δ 138.1 (CH), 137.4 (Cq), 137.1 (Cq), 134.4 (Cq), 133.8 (CH), 129.8 (2×CH), 129.7 (CH), 129.0 (2×CH), 118.2 (CH_2) 115.9 (CH_2), 21.4 (CH_3) ppm; IR (thin film): ν_{max} = 3085, 3005, 1604, 1507 cm^{-1}; LRMS: (EI+): m/z (%): 170 ($[M]^{+\bullet}$, 27), 155 ($[M–CH_3]^{+\bullet}$, 100), 142 ($[M–2×CH_2]^{+\bullet}$, 23), 128 ($[M–C_3H_6]^{+\bullet}$, 30), 115 ($[M–C_4H_7]^{+\bullet}$, 27); HRMS (EI+): calculated for $C_{13}H_{14}$: 170.1096; found: 170.1089.

1-Methoxy-4-(2-vinylbuta-1,3-dien-1-yl)benzene (2c)

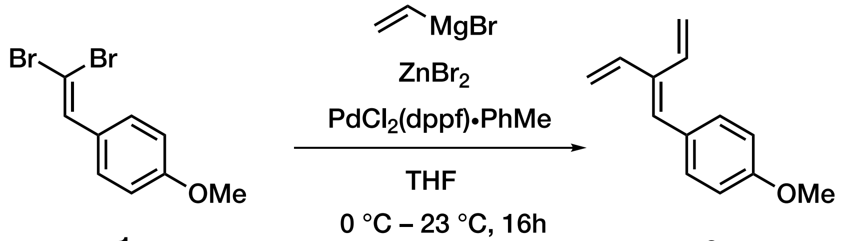

1c 2c

Following **Standard Procedure A**, the reaction mixture containing dibromide **1c** (3.00 g, 10.3 mmol), 1,1'-bis(diphenylphosphino)ferrocene palladium(II) chloride•toluene (0.423 g, 0.514 mmol), $ZnBr_2$ solution in THF (36.1 mL, 1.14 M, 41.1 mmol) and vinyl magnesium bromide (35.8 mL, 0.86 M, 30.8 mmol) in THF (150 mL) was stirred at 23 °C for 16h. After work up, purification by flash column chromatography (90 g SiO_2, 3% EtOAc in PS 40–60) gave dendralene **2c** as a colourless liquid (1.46 g, 7.84 mmol, 76%). R_f = 0.5 (5% EtOAc in PS 40–60); ^{1}H NMR (400 MHz, $CDCl_3$) δ 7.33 (d, J = 8.3 Hz, 2H), 6.88 (d, J = 8.3 Hz, 2H), 6.70 (dd, J = 17.8, 11.1 Hz, 1H), 6.60 (s, 1H), 6.54 (dd, J = 17.2, 10.7 Hz, 1H), 5.51 (d, J = 17.2 Hz, 1H), 5.44 (d, J = 17.8 Hz, 1H), 5.35 (d, J = 11.1 Hz, 1H), 5.18 (d, J = 10.7 Hz, 1H), 3.83 (s, 3H) ppm; ^{13}C NMR (100 MHz, $CDCl_3$) δ 158.9 (Cq), 138.2 (CH), 136.6 (Cq), 133.8 (CH), 131.2 (2×CH), 129.9 (Cq), 129.4 (CH), 118.0 (CH_2), 115.5 (CH_2), 113.7 (2×CH), 55.4 (CH_3) ppm; IR (thin film): ν_{max} = 3086, 3003, 2835, 1601, 1506 cm^{-1}; LRMS: (EI+): m/z (%): 186 ($[M]^{+\bullet}$, 100), 155 ($[M–CH_3O]^{+\bullet}$, 48), 128 ($[M–C_3H_6O]^{+\bullet}$, 62); HRMS (EI+): calculated for $C_{13}H_{14}O$: 186.1045; found: 186.1044.

1,2,3-Trimethoxy-5-(2-vinylbuta-1,3-dien-1-yl)benzene (2d)

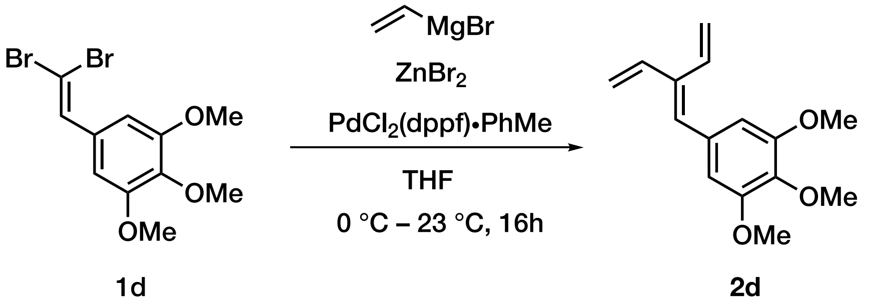

1d 2d

Following **Standard Procedure A**, the reaction mixture containing dibromide **1d** (3.00 g, 8.52 mmol), 1,1'-bis(diphenylphosphino)ferrocene palladium(II) chloride•toluene (0.351 g, 0.426 mmol), $ZnBr_2$ solution in THF (30.0 mL, 1.14 M, 34.1 mmol) and vinyl magnesium bromide (31.2 mL, 0.82 M, 25.6 mmol) in THF (150 mL) was stirred at 23 °C for 16h. After work up, purification by flash column chromatography (90 g SiO_2, 12% EtOAc in PS 40–60) gave dendralene **2d** as a colourless liquid (1.30 g, 5.28 mmol, 62%). R_f = 0.42 (20% EtOAc in PS 40–60); ^{1}H NMR (400 MHz, $CDCl_3$) δ 6.72 (dd, J = 17.6, 10.8 Hz, 1H), 6.61 (s, 2H), 6.59 – 6.47 (m, 2H), 5.52

(dd, J = 16.8, 1.2 Hz, 1H), 5.45 (dd, J = 17.6, 1.6 Hz, 1H), 5.37 (dt, J = 10.8, 1.6 Hz, 1H), 5.20 (dd, J = 10.4, 1.2 Hz, 1H), 3.86 (s, 3H), 3.85 (s, 6H) ppm; ^{13}C NMR (100 MHz, $CDCl_3$) δ 153.0 (2×Cq), 138.0 (CH), 137.8 (Cq), 137.6 (Cq), 133.8 (CH), 132.8 (Cq), 129.6 (CH), 118.5 (CH_2), 116.2 (CH_2), 107.1 (2×CH), 61.1 (CH_3), 56.2 (2×CH_3) ppm; IR (thin film): ν_{max} = 2937, 2835,1584,1503 cm^{-1}; LRMS: (EI+): m/z (%): 246 ($[M]^{+\bullet}$, 49), 215 ($[M–CH_3O]^{+\bullet}$, 100), 184 ($[M–2×CH_3O]^{+\bullet}$, 22); HRMS (EI+): calculated for $C_{15}H_{18}O_3$: 246.1256; found: 246.1259.

1-Nitro-4-(2-vinylbuta-1,3-dien-1-yl)benzene (2e)

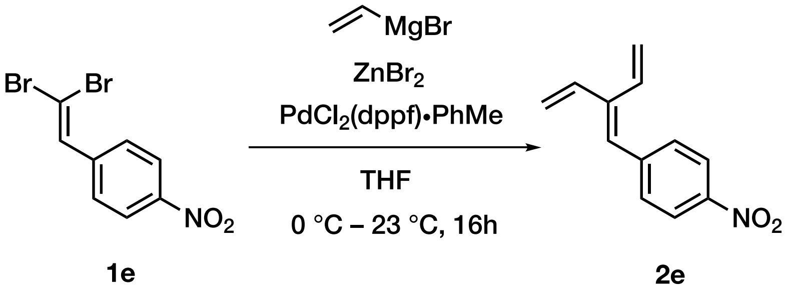

Following **Standard Procedure A**, the reaction mixture containing dibromide **1e** (3.00 g, 9.77 mmol), 1,1'-bis(diphenylphosphino)ferrocene palladium(II) chloride•toluene (0.403 g, 0.489 mmol), $ZnBr_2$ solution in THF (43.9 mL, 0.89 M, 39.1 mmol) and vinyl magnesium bromide (39.0 mL, 0.75 M, 29.3 mmol) in THF (150 mL) was stirred at 23 °C for 16h. After work up, purification by flash column chromatography (90 g SiO_2, 1% EtOAc in PS 40–60) gave dendralene **2e** as a yellow liquid (0.913 g, 4.54 mmol, 46%). R_f = 0.58 (5% EtOAc in PS 40–60); ^{1}H NMR (400 MHz, C_6D_6) δ 7.79 (d, J = 8.8 Hz, 2H), 6.89 (d, J = 8.8 Hz, 2H), 6.36 (dd, J = 17.4, 11.1 Hz, 2H), 6.22 (s, 1H), 5.44 (dd, J = 17.2, 1.4 Hz, 1H), 5.31 (dd, J = 17.7, 1.3 Hz, 1H), 5.15 (d, J = 11.2, Hz, 1H), 5.11 (dd, J = 10.7, 1.3 Hz, 1H) ppm; ^{13}C NMR (100 MHz, C_6D_6) δ 146.7 (Cq), 143.3 (Cq), 141.2 (Cq), 137.7 (CH), 132.8 (CH), 130.1 (2×CH), 127.6 (CH), 123.5 (2×CH), 120.4 (CH_2), 117.9 (CH_2) ppm; IR (thin film): ν_{max} = 3088, 3009, 2447, 1591, 1511 cm^{-1}; LRMS: (EI+): m/z (%): 201 ($[M]^{+\bullet}$, 70), 155 ($[M–NO_2]^{+\bullet}$, 61), 153 (100); HRMS (EI+): calculated for $C_{12}H_{11}NO_2$: 201.0790; found: 201.0787.

3-Vinylnona-1,3-diene (2f)

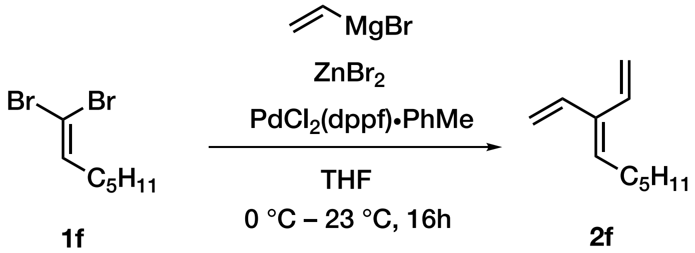

Following **Standard Procedure A**, the reaction mixture containing dibromide **1f** (1.02 g, 3.98 mmol), 1,1'-bis(diphenylphosphino)ferrocene palladium(II) chloride•toluene (142 mg, 0.195 mmol), $ZnBr_2$ solution in THF (12.1 mL, 1.29 M, 15.6 mmol) and vinyl magnesium bromide (18.7 mL, 0.64 M, 11.9 mmol) in THF (50 mL) was stirred at 23 °C for 16h. After work up, purification by flash column chromatography (80 g SiO_2, PS 40–60) gave dendralene **2f** as a colourless liquid (0.51 g, 3.33 mmol, 85%). R_f = 0.81 (100% PS 40–60); ^{1}H NMR (400 MHz, $CDCl_3$) δ 6.49 (dd, J = 18.3, 10.6 Hz, 1H), 6.40 (dd, J = 17.3, 10.7 Hz, 1H), 5.63 (t, J = 7.6 Hz, 1H), 5.26 (d, J = 16.5 Hz, 3H), 5.02 (dd, J = 10.7, 1.4 Hz, 1H), 2.20 (q, J = 7.5 Hz, 2H), 1.48 – 1.18 (m, 6H), 0.89 (t, J = 6.9 Hz, 3H) ppm; ^{13}C NMR (100 MHz, $CDCl_3$) δ 138.2 (CH), 137.0 (Cq), 133.2 (CH), 132.3 (CH), 117.1 (CH_2), 113.7 (CH_2), 31.7 (CH_2), 29.5 (CH_2), 28.4 (CH_2), 22.7 (CH_2), 14.2 (CH_3) ppm; IR (thin film): ν_{max} = 3088, 2957, 2924, 2856, 1627 cm^{-1}; LRMS: (EI+): m/z (%): 150 ($[M]^{+\bullet}$, 60), 121 ($[M–CH_3CH_2]^{+\bullet}$, 70), 79 (100); HRMS (EI+): calculated for $C_{11}H_{18}$: 150.1409; found: 150.1413.

3-Vinyltetradeca-1,3-diene (2g)

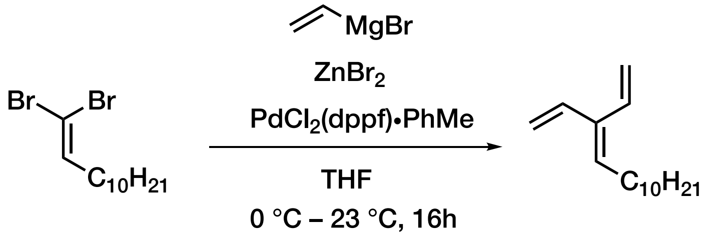

1g **2g**

Following **Standard Procedure A**, the reaction mixture containing dibromide **1g** (1.50 g, 4.60 mmol), 1,1'-bis(diphenylphosphino)ferrocene palladium(II) chloride•toluene (0.19 g, 0.23 mmol), $ZnBr_2$ solution in THF (16.3 mL, 1.13 M, 18.4 mmol) and vinyl magnesium bromide (18.4 mL, 0.75 M, 13.8 mmol) in THF (75 mL) was stirred at 23 °C for 16h. After work up, purification by flash column chromatography (45 g SiO_2, PS 40–60) gave dendralene **2g** as a colourless liquid (0.653 g, 2.96 mmol, 64%). R_f = 0.61 (100% PS 40–60); ^{1}H NMR (400 MHz, $CDCl_3$) δ 6.49 (dd, J = 18.3, 10.6 Hz, 1H), 6.40 (dd, J = 17.4, 10.7 Hz, 1H), 5.63 (t, J = 7.6 Hz, 1H), 5.31 – 5.23 (m, 3H), 5.02 (dd, J = 10.7, 1.2 Hz, 1H), 2.20 (q, J = 7.4 Hz, 2H), 1.43 – 1.19 (m, 16H), 0.88 (t, J = 6.8 Hz, 3H) ppm; ^{13}C NMR (100 MHz, $CDCl_3$) δ 138.1 (CH), 136.9 (Cq), 133.2 (CH), 132.3 (CH), 117.1 (CH_2), 113.7 (CH_2), 32.1 (CH_2), 29.8 (CH_2), 29.7 (2×CH_2), 29.6 (CH_2), 29.5 (CH_2), 29.5 (CH_2), 28.5 (CH_2), 22.9 (CH_2), 14.3 (CH_3) ppm; IR (thin film): ν_{max} = 2922, 2853, 1679 cm^{-1}; LRMS: (EI+): m/z (%): 220 ($[M]^{+\bullet}$, 10), 149 ($[M–C_5H_{11}]^{+\bullet}$, 5), 135 ($[M–C_6H_{13}]^{+\bullet}$, 11),

121 ($[M–C_7H_{15}]^{+\bullet}$, 28), 107 ($[M–C_8H_{17}]^{+\bullet}$, 38), 93 ($[M–C_9H_{19}]^{+\bullet}$, 76), 79 ($[M–C_{10}H_{21}]^{+\bullet}$, 100); HRMS (EI+): calculated for $C_{16}H_{28}$: 220.2191; found: 220.2188.

(2-Vinylbuta-1,3-dien-1-yl)cyclohexane (2h)

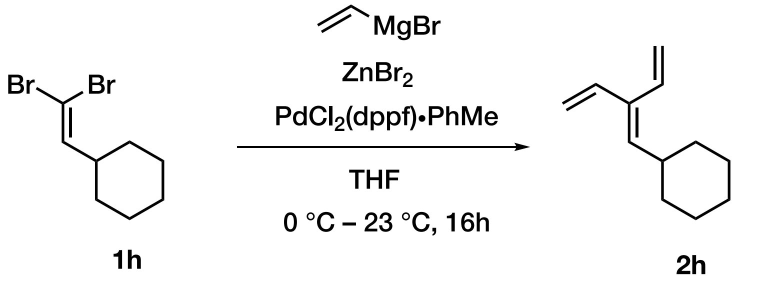

Following **Standard Procedure A**, the reaction mixture containing dibromide **1h** (3.00 g, 11.2 mmol), 1,1'-bis(diphenylphosphino)ferrocene palladium(II) chloride•toluene (0.461 g, 0.560 mmol), $ZnBr_2$ solution in THF (39.3 mL, 1.14 M, 44.8 mmol) and vinyl magnesium bromide (41 mL, 0.82 M, 33.6 mmol) in THF (150 mL) was stirred at 23 °C for 16h. Purification by flash column chromatography (90 g SiO_2, PS 40–60) gave dendralene **2h** as a colourless liquid (1.48 g, 9.12 mmol, 81%). R_f = 0.72 (100% PS 40–60); ^{1}H NMR (400 MHz, $CDCl_3$) δ 6.51 (dd, J = 17.6, 11.2 Hz, 1H), 6.38 (dd, J = 17.3, 10.7 Hz, 1H), 5.46 (d, J = 9.6 Hz, 1H), 5.33 – 5.17 (m, 3H), 5.02 (d, J = 10.7 Hz, 1H), 2.51 – 2.32 (m, 1H), 1.81 – 1.57 (m, 5H), 1.41 – 0.98 (m, 5H) ppm; ^{13}C NMR (100 MHz, $CDCl_3$) δ 138.7 (CH), 138.2 (CH), 135.3 (Cq), 132.5 (CH), 116.8 (CH_2), 113.9 (CH_2), 37.2 (CH), 33.2 (2×CH_2), 26.2 (CH_2), 26.0 (2×CH_2) ppm; IR (thin film): ν_{max} = 3087, 2923, 2850, 1628 cm^{-1}; LRMS: (EI+): m/z (%): 162 ($[M]^{+\bullet}$, 31), 147 ($[M–CH_3]^{+\bullet}$, 12), 133 ($[M–C_2H_5]^{+\bullet}$, 26), 79 (100); HRMS (EI+): calculated for $C_{12}H_{18}$: 162.1409; found: 162.1410.

(4-Vinylhexa-3,5-dien-1-yl)benzene (2i)

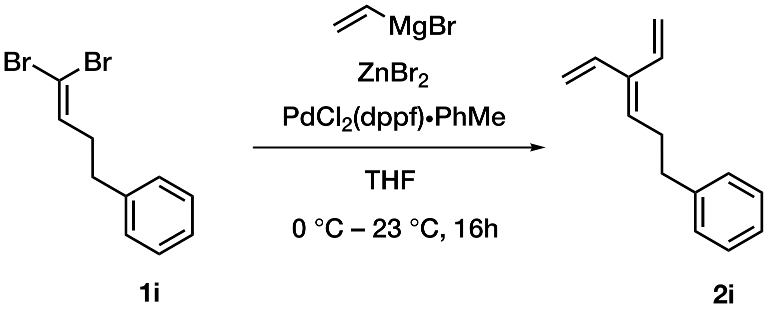

Following **Standard Procedure A**, the reaction mixture containing dibromide **1i** (3.00 g, 10.3 mmol), 1,1'-bis(diphenylphosphino)ferrocene palladium(II) chloride•toluene (0.426 g, 0.517 mmol), $ZnBr_2$ solution in THF (34.5 mL, 1.20 M, 41.4 mmol) and vinyl magnesium bromide (36.1 mL, 0.86 M, 31.0 mmol) in THF (150 mL) was stirred at 23 °C for 16h. After work up, purification by

flash column chromatography (90 g SiO_2, 100% PS 40–60) gave dendralene **2i** as a pale yellow liquid (1.56 g, 8.47 mmol, 82%). R_f = 0.44 (100% PS 40–60); ^{1}H NMR (400 MHz, $CDCl_3$) δ 7.33 – 7.27 (m, 2H), 7.23 – 7.18 (m, 3H), 6.57 – 6.32 (m, 2H), 5.68 (t, J = 7.5 Hz, 1H), 5.32 – 5.21 (m, 3H), 5.05 (dd, J = 10.4, 1.6 Hz, 1H), 2.77 – 2.70 (m, 2H), 2.55 (q, J = 7.6 Hz, 2H) ppm; ^{13}C NMR (100 MHz, $CDCl_3$) δ 141.9 (Cq), 138.0 (CH), 137.7 (Cq), 132.1 (CH), 131.6 (CH), 128.6 (2×CH), 128.5 (2×CH), 126.0 (CH), 117.6 (CH_2), 114.2 (CH_2), 36.0 (CH_2), 30.4 (CH_2) ppm; IR (thin film): ν_{max} = 3086, 3026, 2924, 2856, 1603 cm^{-1}; LRMS: (EI+): m/z (%): 184 ($[M]^{+\bullet}$, 10), 169 ($[M–CH_3]^{+\bullet}$, 12), 155 ($[M–C_2H_5]^{+\bullet}$, 24), 91 ($[M–C_7H_9]^{+\bullet}$, 100), 77 ($[M–C_8H_{11}]^{+\bullet}$, 35); HRMS (EI+): calculated for $C_{14}H_{16}$: 184.1252; found: 184.1251.

(*E*)-(4-Vinylhexa-1,3,5-trien-1-yl)benzene (2j)

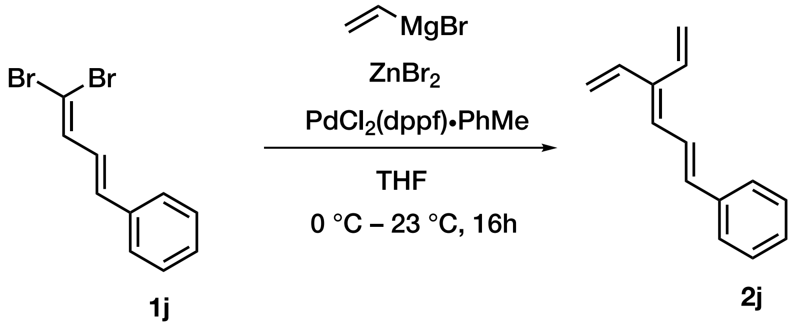

1j **2j**

Following **Standard Procedure A**, the reaction mixture containing dibromide **1j** (3.00 g, 10.4 mmol), 1,1'-bis(diphenylphosphino)ferrocene palladium(II) chloride•toluene (0.429 g, 0.521 mmol), $ZnBr_2$ solution in THF (36.6 mL, 1.14 M, 41.7 mmol) and vinyl magnesium bromide (36.3 mL, 0.86 M, 31.3 mmol) in THF (150 mL) was stirred at 23 °C for 16h. After work up, purification by flash column chromatography (90 g SiO_2, 100% PS 40–60) gave dendralene **2j** as a colourless liquid (1.22 g, 6.69 mmol, 64%). R_f = 0.32 (100% PS 40–60); ^{1}H NMR (400 MHz, $CDCl_3$) δ 7.43 (d, J = 7.8 Hz, 2H), 7.32 (t, J = 7.6 Hz, 2H), 7.29 – 7.18 (m, 2H), 6.78 – 6.58 (m, 2H), 6.50 (dd, J = 17.4, 10.8 Hz, 1H), 6.37 (d, J = 11.4 Hz, 1H), 5.50 – 5.34 (m, 3H), 5.16 (d, J = 10.8 Hz, 1H) ppm; ^{13}C NMR (100 MHz, $CDCl_3$) δ 138.2 (Cq), 137.9 (CH), 137.6 (Cq), 134.2 (CH), 132.0 (CH), 130.7 (CH), 128.8 (2×CH), 127.8 (CH), 126.7 (2×CH), 125.5 (CH), 119.1 (CH_2), 115.4 (CH_2) ppm; IR (thin film): ν_{max} = 3083, 3031, 3002, 1812, 1602 cm^{-1}; LRMS: (EI+): m/z (%): 182 ($[M]^{+\bullet}$, 62), 167 ($[M–CH_3]^{+\bullet}$, 100), 141 ($[M–C_3H_5]^{+\bullet}$, 29); HRMS (EI+): calculated for $C_{14}H_{14}$: 182.1096; found: 182.1095.

(4-Vinylhexa-3,5-dien-1-yn-1-yl)benzene (2k)

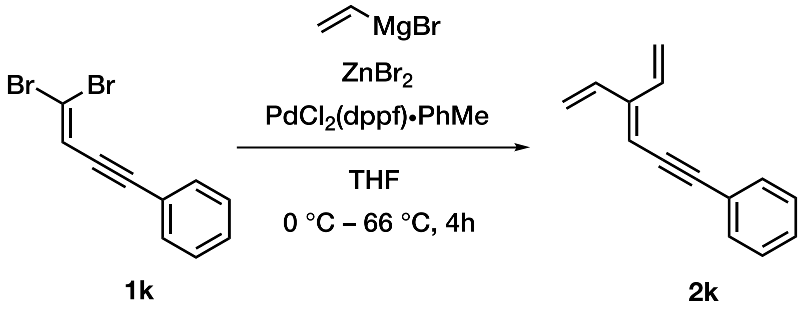

Following **Standard Procedure A**, the reaction mixture containing dibromide **1k** (1.00 g, 3.49 mmol), 1,1'-bis(diphenylphosphino)ferrocene palladium(II) chloride•toluene (0.140 g, 0.175 mmol), $ZnBr_2$ solution in THF (12.7 mL, 1.10 M, 14.0 mmol) and vinyl magnesium bromide (19.1 mL, 0.55 M, 10.5 mmol) in THF (50 mL) was heated under reflux for 4h. After work up, purification by flash column chromatography (30 g SiO_2, 100% PS 40–60) gave dendralene **2k** as a pale yellow liquid (0.398 g, 2.21 mmol, 63%). R_f = 0.50 (100% PS 40–60); ^{1}H NMR (400 MHz, $CDCl_3$) δ 7.50 – 7.42 (m, 2H), 7.37 – 7.28 (m, 3H), 6.96 (dd, J = 17.6, 11.2 Hz, 1H), 6.52 (dd, J = 17.2, 10.8 Hz, 1H), 5.88 (s, 1H), 5.65 (d, J = 17.6 Hz, 1H), 5.56 (d, J = 17.2 Hz, 1H), 5.43 (d, J = 11.2 Hz, 1H), 5.25 (d, J = 10.8 Hz, 1H) ppm; ^{13}C NMR (100 MHz, $CDCl_3$) δ 146.8 (Cq), 135.0 (CH), 133.2 (CH), 131.6 (2×CH), 128.5 (2×CH), 128.4 (CH), 123.7 (Cq), 118.7 (CH_2), 117.2 (CH_2), 108.6 (CH), 97.6 (Cq), 87.8 (Cq) ppm; IR (thin film): ν_{max} = 3086, 3010, 2198, 1830, 1612, 1597 cm^{-1}; LRMS: (EI+): m/z (%): 180 ($[M]^{+•}$, 100), 165 ($[M–CH_3]^{+•}$, 60), 152 ($[M–C_2H_4]^{+•}$, 38); HRMS (EI+): calculated for $C_{14}H_{12}$: 180.0939; found: 180.0939.

1-Tosyl-3-(2-vinylbuta-1,3-dien-1-yl)-1H-indole (2l)

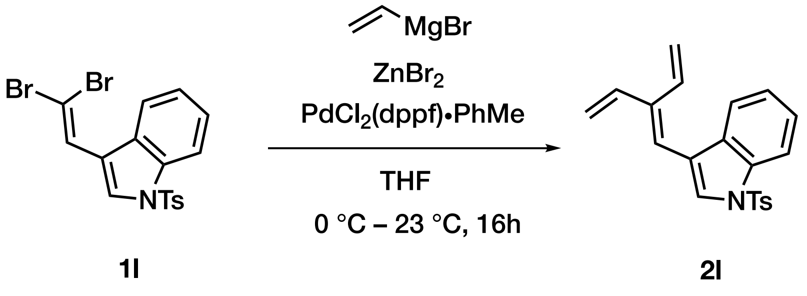

Following **Standard Procedure A**, the reaction mixture containing dibromide **1l** (650 mg, 1.43 mmol), 1,1'-bis(diphenylphosphino)ferrocene palladium(II) chloride•toluene (60 mg, 0.0714 mmol), $ZnBr_2$ solution in THF (5.0 mL, 1.14 M, 5.71 mmol) and vinyl magnesium bromide (5.2 mL, 0.82 M, 4.28 mmol) in THF (35 mL) was stirred at 23 °C for 16h. After work up, purification by flash column chromatography (20 g SiO_2, 3% EtOAc in PS 40–60) gave dendralene **2l** as a pale yellow liquid (383 mg, 1.10 mmol, 77%). R_f = 0.33 (10% EtOAc in PS 40–60); ^{1}H NMR (400 MHz, $CDCl_3$) δ 7.99 (d, J = 8.0 Hz, 1H), 7.77 (d, J = 8.4 Hz, 2H), 7.63 (s, 1H), 7.57 (d, J = 7.6 Hz, 1H),

7.38 – 7.31 (m, 1H), 7.29 – 7.20 (m, 3H), 6.72 (dd, *J* = 18.0, 11.2 Hz, 1H), 6.63 – 6.53 (m, 2H), 5.59 – 5.49 (m, 2H), 5.46 (d, *J* = 11.2 Hz, 1H), 5.24 (dd, *J* = 10.8, 1.2 Hz, 1H), 2.34 (s, 3H) ppm; ^{13}C NMR (100 MHz, $CDCl_3$) δ 145.2 (Cq), 139.3 (Cq), 137.5 (CH), 135.2 (Cq), 134.8 (Cq), 133.6 (CH), 130.8 (Cq), 130.0 (2×CH), 127.0 (2×CH), 125.2 (CH), 125.2 (CH), 123.5 (CH), 119.8 (CH), 119.2 (Cq), 119.1 (CH_2), 118.2 (CH), 116.5 (CH_2), 113.8 (CH), 21.7 (CH_3); IR (thin film): ν_{max} = 3005, 2923, 1740, 1596, 1446 cm^{-1}; LRMS: (EI+): m/z (%): 349 ($[M]^{+\bullet}$, 100), 295 ($[M–C_4H_6]^{+\bullet}$, 23); HRMS (EI+): calculated for $C_{21}H_{19}NO_2S$: 349.1137; found: 349.1136.

2-Methyl-5-(2-vinylbuta-1,3-dien-1-yl)furan (2m)

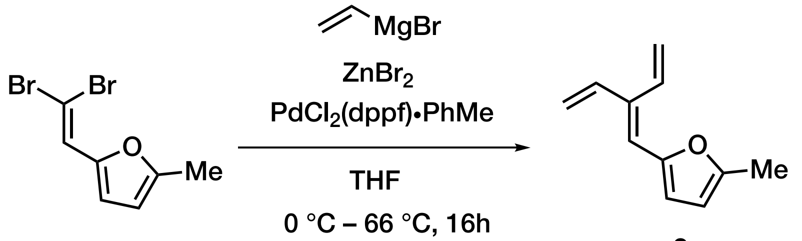

1m **2m**

Following **Standard Procedure A**, the reaction mixture containing dibromide **1m** (3.00 g, 11.3 mmol), 1,1'-bis(diphenylphosphino)ferrocene palladium(II) chloride•toluene (0.465 g, 0.564 mmol), $ZnBr_2$ solution in THF (37.6 mL, 1.2 M, 45.1 mmol) and vinyl magnesium bromide (39.4 mL, 0.86 M, 33.8 mmol) in THF (150 mL) was heated under reflux for 16h. After work up, purification by flash column chromatography (90 g SiO_2, 100% PS 40–60) gave dendralene **2m** as a yellow liquid (1.25 g, 7.80 mmol, 69%). R_f = 0.40 (100% PS 40–60); ^{1}H NMR (400 MHz, $CDCl_3$) δ 7.07 (dd, *J* = 17.7, 11.1 Hz, 1H), 6.52 (dd, *J* = 17.2, 10.8 Hz, 1H), 6.34 – 6.27 (m, 2H), 6.02 (d, *J* = 2.7 Hz, 1H), 5.54 – 5.39 (m, 2H), 5.35 (d, *J* = 11.1 Hz, 1H), 5.15 (dd, *J* = 10.7, 1.3 Hz, 1H), 2.33 (s, 3H) ppm; ^{13}C NMR (100 MHz, $CDCl_3$) δ 152.9 (Cq), 151.7 (Cq), 137.2 (CH), 134.0 (Cq), 133.8 (CH), 117.6 (CH_2), 116.9 (CH), 115.4 (CH_2), 113.0 (CH), 108.1 (CH), 14.0 (CH_3) ppm; IR (thin film): ν_{max} = 3088, 2921, 1577, 1521 cm^{-1}; LRMS: (EI+): m/z (%): 160 ($[M]^{+\bullet}$, 88), 145 ($[M–CH_3]^{+\bullet}$, 23), 117 ($[M–C_3H_7]^{+\bullet}$, 100); 91 ($[M–C_5H_9]^{+\bullet}$, 36) HRMS (EI+): calculated for $C_{11}H_{12}O$: 160.0888; found: 160.0888.

1-Methoxy-4-(3-methyl-2-(prop-1-en-2-yl)buta-1,3-dien-1-yl)benzene (2n)

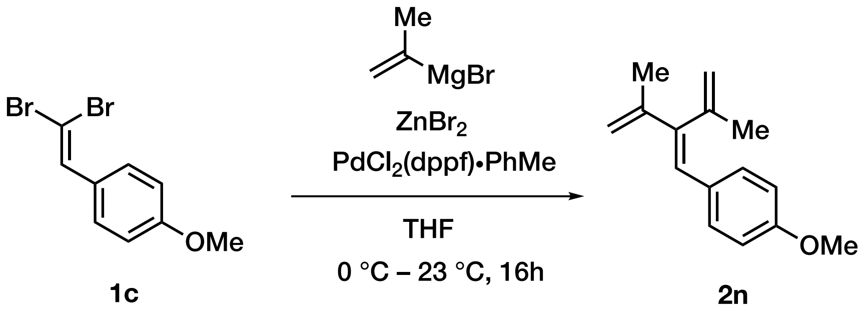

Following **Standard Procedure A**, the reaction mixture containing dibromide **1c** (500 mg, 1.7 mmol), 1,1'-bis(diphenylphosphino)ferrocene palladium(II) chloride•toluene (71 mg, 0.086 mmol), $ZnBr_2$ solution in THF (6.0 mL, 1.14 M, 6.85 mmol) and isopropenyl magnesium bromide (9.2 mL, 0.56 M, 5.14 mmol) in THF (25 mL) was stirred at 23 °C for 16h. After work up, purification by flash column chromatography (15 g SiO_2, 1.5% EtOAc in PS 40–60) gave dendralene **2n** as a colourless liquid (251 mg, 1.18 mmol, 69%). R_f = 0.73 (5% EtOAc in PS 40–60); ^{1}H NMR (400 MHz, $CDCl_3$) δ 7.48 (d, J = 8.8 Hz, 2H), 6.83 (d, J = 8.8 Hz, 2H), 6.38 (s, 1H), 5.23 (s, 1H), 5.17 (s, 1H), 5.04 (s, 1H), 4.86 (s, 1H), 3.81 (s, 3H), 2.02 (s, 3H), 1.89 (s, 3H) ppm; ^{13}C NMR (100 MHz, $CDCl_3$) δ 158.6 (Cq) , 143.7 (Cq), 143.3 (Cq), 143.2 (Cq), 130.5 (2×CH), 130.2 (Cq), 124.1 (CH), 116.6 (CH_2), 114.9 (CH_2), 113.7 (2×CH), 55.4 (CH_3), 23.2 (CH_3), 21.0 (CH_3) ppm; IR (thin film): ν_{max} = 3079, 2950, 2836, 1599, 1509 cm^{-1}; LRMS: (EI+): m/z (%): 214 ([M]$^{+\bullet}$, 27), 199 ([M–CH_3]$^{+\bullet}$, 100), 184 ([M–2×CH_3]$^{+\bullet}$,17), 158 ([M–C_4H_8]$^{+\bullet}$,17); HRMS (EI+): calculated for $C_{15}H_{18}O$: 214.1358; found: 214.1358.

1,2,3-Trimethoxy-5-(3-methyl-2-(prop-1-en-2-yl)buta-1,3-dien-1-yl)benzene (2o)

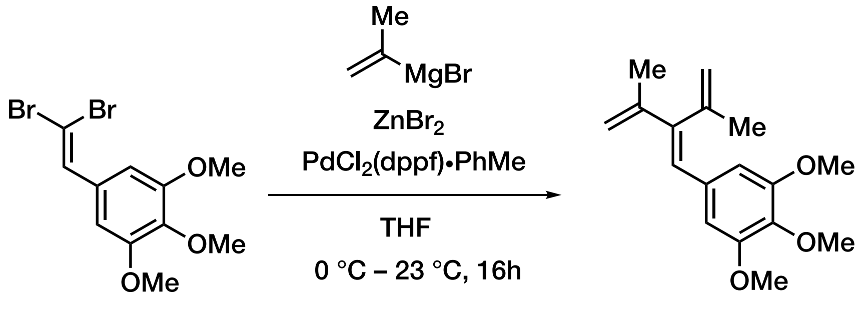

Following **Standard Procedure A**, the reaction mixture containing dibromide **1d** (1.00 g, 2.84 mmol), 1,1'-bis(diphenylphosphino)ferrocene palladium(II) chloride•toluene (117 mg, 0.142 mmol), $ZnBr_2$ solution in THF (9.2 mL, 1.23 M, 11.4 mmol) and isopropenyl magnesium bromide (16.7 mL, 0.51 M, 8.52 mmol) in THF (10 mL) was stirred at 23 °C for 16h. After work up, purification by flash column chromatography (40 g SiO_2, 10% EtOAc in PS 40–60) gave dendralene **2o** as a

pale yellow solid (0.64 g, 2.33 mmol, 82%). R_f = 0.34 (10% EtOAc in PS 40–60); m.p. 50 – 52 °C (CH_2Cl_2); ^{1}H NMR (400 MHz, $CDCl_3$) δ 6.85 (s, 2H), 6.35 (s, 1H), 5.29 (s, 1H), 5.22 (s, 1H), 5.08 (s, 1H), 4.91 (s, 1H), 3.85 (s, 3H), 3.84 (s, 6H), 2.03 (s, 3H), 1.92 (s, 3H) ppm; ^{13}C NMR (100 MHz, $CDCl_3$) δ 152.9 (2×Cq), 144.6 (Cq), 144.1 (Cq), 143.0 (Cq), 137.3 (Cq), 132.9 (Cq), 124.5 (CH), 116.6 (CH_2), 115.7 (CH_2), 106.5 (2×CH), 61.1 (CH_3), 56.1 (2×CH_3), 23.3 (CH_3), 21.0 (CH_3) ppm; IR (thin film): ν_{max} = 2997, 2939, 2834, 1573, 1504 cm^{-1}; LRMS: (EI+): m/z (%): 274 ($[M]^{+\bullet}$, 60), 259 ($[M–CH_3]^{+\bullet}$, 42), 243 ($[M–CH_3O]^{+\bullet}$,100), 212 ($[M–2×CH_3O]^{+\bullet}$, 18); HRMS (EI+): calculated for $C_{17}H_{22}O_3$: 274.1569; found: 274.1567.

(*E*)-(5-Methyl-4-(prop-1-en-2-yl)hexa-1,3,5-trien-1-yl)benzene (2p)

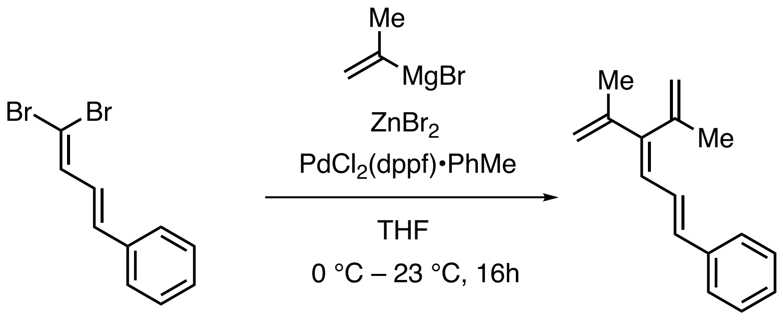

1j **2p**

Following **Standard Procedure A**, the reaction mixture containing dibromide **1j** (1.00 g, 3.47 mmol), 1,1'-bis(diphenylphosphino)ferrocene palladium(II) chloride•toluene (143 mg, 0.174 mmol), $ZnBr_2$ solution in THF (11.3 mL, 1.23 M, 13.9 mmol) and isopropenyl magnesium bromide (18.6 mL, 0.56 M, 10.4 mmol) in THF (50 mL) was stirred at 23 °C for 16h. After work up, purification by flash column chromatography (40 g SiO_2, 100% PS 40–60) gave dendralene **2p** as a colourless liquid (0.51 g, 2.42 mmol, 70%). R_f = 0.38(100% PS 40–60); ^{1}H NMR (400 MHz, $CDCl_3$) δ 7.40 (d, J = 7.6 Hz, 2H), 7.31 (t, J = 7.4 Hz, 2H), 7.21 (t, J = 7.2 Hz, 1H), 7.04 (dd, J = 15.6, 11.2 Hz, 1H), 6.63 (d, J = 15.6 Hz, 1H), 6.29 (d, J = 11.2 Hz, 1H), 5.27 (s, 1H), 5.13 (s, 1H), 5.05 (s, 1H), 4.81 (s, 1H), 1.98 (s, 3H), 1.91 (s, 3H) ppm; ^{13}C NMR (100 MHz, $CDCl_3$) δ 146.9 (Cq), 143.2 (Cq), 141.8 (Cq), 137.9 (Cq), 133.0 (CH), 128.7 (2×CH), 127.5 (CH), 127.4 (CH), 126.6 (2×CH), 125.4 (CH), 116.2 (CH_2), 115.8 (CH_2), 24.0 (CH_3), 20.5 (CH_3) ppm; IR (thin film): ν_{max} = 3081, 3032, 2969, 1799, 1637, 1600 cm^{-1}; LRMS: (EI+): m/z (%): 210 ($[M]^{+\bullet}$, 83), 195 ($[M–CH_3]^{+\bullet}$, 100), 180 ($[M–2×CH_3]^{+\bullet}$,23); HRMS (EI+): calculated for $C_{16}H_{18}$: 210.1409; found: 210.1408.

(5-Methyl-4-(prop-1-en-2-yl)hexa-3,5-dien-1-yn-1-yl)benzene (2q)

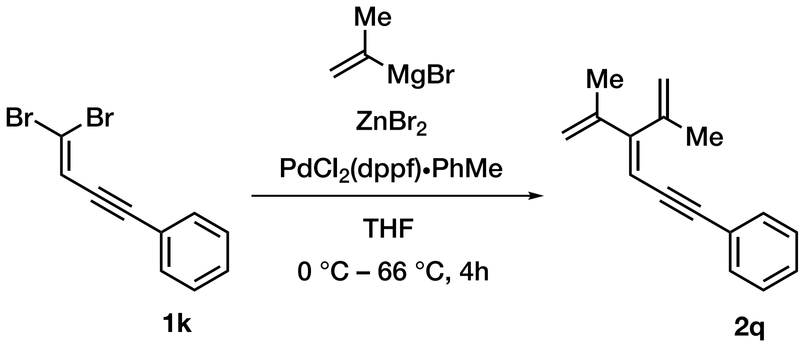

Following **Standard Procedure A**, the reaction mixture containing dibromide **1k** (200 mg, 0.70 mmol), 1,1'-bis(diphenylphosphino)ferrocene palladium(II) chloride•toluene (29 mg, 0.035 mmol), $ZnBr_2$ solution in THF (1.9 mL, 1.48 M, 2.8 mmol) and isopropenyl magnesium bromide (3.5 mL, 0.60 M, 2.1 mmol) in THF (10 mL) was stirred at 66 °C for 4h. After work up, purification by flash column chromatography (8 g $AgNO_3$ impregnated silica, 100% PS 40–60) gave dendralene **2q** as a colourless liquid (58 mg, 0.28 mmol, 40%). R_f = 0.30 (100% PS 40–60); ^{1}H NMR (400 MHz, $CDCl_3$) δ 7.41 – 7.38 (m, 2H), 7.34 – 7.27 (m, 3H), 5.76 (s, 1H), 5.24 (s, 1H), 5.21 (s, 1H), 5.11 (s, 1H), 4.90 (s, 1H), 1.98 (s, 3H), 1.95 (s, 3H) ppm; ^{13}C NMR (100 MHz, $CDCl_3$) δ 156.4 (Cq), 143.1 (Cq), 141.3 (Cq), 131.5 (2×CH), 128.4 (2×CH), 128.1 (CH), 124.1 (Cq), 117.7 (CH_2), 115.9 (CH_2), 105.1 (CH), 95.4 (Cq), 88.6 (Cq), 23.0 (CH_3), 20.3 (CH_3) ppm; IR (thin film): ν_{max} = 3079, 2970, 2196, 1801, 1598 cm^{-1}; LRMS: (EI+): m/z (%): 208 ([M]$^{+\bullet}$, 46), 193 ([M–CH_3]$^{+\bullet}$, 40), 178 ([M–2×CH_3]$^{+\bullet}$,100); HRMS (EI+): calculated for $C_{16}H_{16}$: 208.1252; found: 208.1255.

2-Methyl-5-(3-methyl-2-(prop-1-en-2-yl)buta-1,3-dien-1-yl)furan (2r)

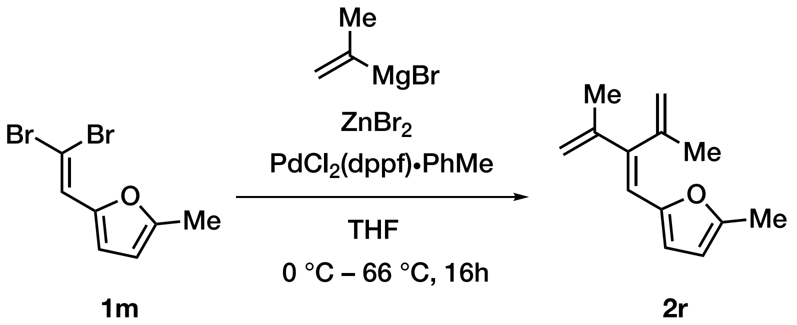

Following **Standard Procedure A**, the reaction mixture containing dibromide **1m** (2.30 g, 8.65 mmol), 1,1'-bis(diphenylphosphino)ferrocene palladium(II) chloride•toluene (0.356 g, 0.432 mmol), $ZnBr_2$ solution in THF (28.1 mL, 1.23 M, 34.6 mmol) and isopropenyl magnesium bromide (46.3 mL, 0.56 M, 26.0 mmol) in THF (115 mL) was stirred at 66 °C for 16h. After work up, purification by flash column chromatography (70 g SiO_2, 100% PS 40–60) and further by $AgNO_3$ impregnated silica gave dendralene **2r** as a colourless liquid (0.653 g, 3.47 mmol, 40%).

R$_f$ = 0.38 (100% PS 40–60); ^{1}H NMR (400 MHz, $CDCl_3$) δ 6.48 (s, 1H), 6.31 (s, 1H), 5.99 (s, 1H), 5.23 (s, 1H), 5.16 (s, 1H), 5.03 (s, 1H), 4.83 (s, 1H), 2.30 (s, 3H), 2.00 (s, 3H), 1.91 (s, 3H) ppm; ^{13}C NMR (100 MHz, $CDCl_3$) δ 151.5 (Cq), 151.4 (Cq), 143.9 (Cq), 142.1 (Cq), 141.9 (Cq), 115.6 (CH_2), 115.4 (CH_2), 113.5 (CH), 110.2 (CH), 108.2 (CH), 22.2 (CH_3), 20.5 (CH_3), 13.8 (CH_3) ppm; IR (thin film): ν_{max} = 3078, 2967, 2947, 1642, 1603, 1526 cm^{-1}; LRMS: (EI+): m/z (%): 188 ([M]$^{+\bullet}$, 100), 173 ([M–CH_3]$^{+\bullet}$, 46), 158 ([M–2×CH_3]$^{+\bullet}$, 14); HRMS (EI+): calculated for $C_{13}H_{16}O$: 188.1201; found: 188.1202.

1,2,3-Trimethoxy-5-(4-methyl-2-(2-methylprop-1-en-1-yl)penta-1,3-dien-1-yl)benzene (2s)

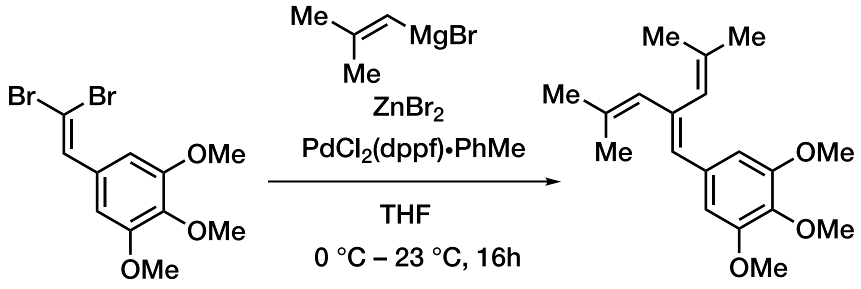

1d **2s**

Following **Standard Procedure A**, the reaction mixture containing dibromide **1d** (200 mg, 0.568 mmol), 1,1'-bis(diphenylphosphino)ferrocene palladium(II) chloride•toluene (23 mg, 0.028 mmol), $ZnBr_2$ solution in THF (1.9 mL, 1.23 M, 2.27 mmol) and isobutenyl magnesium bromide (3.9 mL, 0.44 M, 1.70 mmol) in THF (10 mL) was stirred at 23 °C for 16h. After work up, purification by flash column chromatography (5 g SiO_2, 5% EtOAc in PS 40–60) gave dendralene **2s** as a colourless liquid (133 mg, 0.440 mmol, 77%). R$_f$ = 0.40 (20% EtOAc in PS 40–60);

^{1}H NMR (400 MHz, $CDCl_3$) δ 6.70 (s, 2H), 6.18 (s, 1H), 6.00 (s, 1H), 5.82 (s, 1H), 3.85 (s, 3H), 3.83 (s, 6H), 1.84 (s, 3H), 1.83 (s, 6H), 1.62 (s, 3H) ppm; ^{13}C NMR (100 MHz, $CDCl_3$) δ 152.8 (2×Cq), 136.8 (Cq), 136.8 (Cq), 136.1 (Cq), 134.6 (Cq), 134.0 (Cq), 129.2 (CH), 128.6 (CH), 124.7 (CH), 106.2 (2×CH), 61.0 (CH_3), 56.0 (2×CH_3), 27.1 (CH_3), 26.0 (CH_3), 19.8 (CH_3), 19.7 (CH_3) ppm; IR (thin film): ν_{max} = 2962, 2928, 2908, 1570, 1504 cm^{-1}; LRMS: (EI+): m/z (%): 302 ([M]$^{+\bullet}$, 100), 287 ([M–CH_3]$^{+\bullet}$, 51), 272 ([M–2×CH_3]$^{+\bullet}$,11); HRMS (EI+): calculated for $C_{19}H_{26}O_3$: 302.1882; found: 302.1891.

1-Methoxy-4-(4-methyl-2-(2-methylprop-1-en-1-yl)penta-1,3-dien-1-yl)benzene (2t)

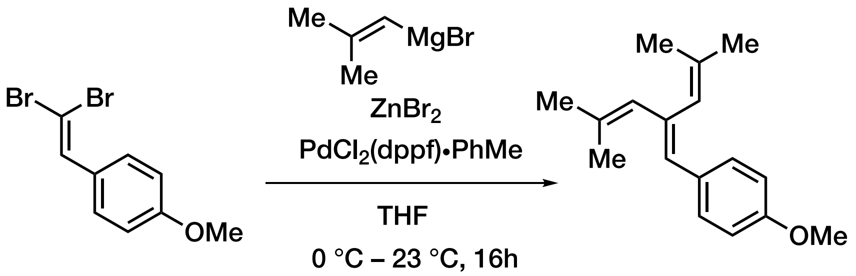

1c **2t**

Following **Standard Procedure A**, the reaction mixture containing dibromide **1c** (200 mg, 0.685 mmol), 1,1'-bis(diphenylphosphino)ferrocene palladium(II) chloride•toluene (28 mg, 0.034 mmol), $ZnBr_2$ solution in THF (2.8 mL, 1.22 M, 3.43 mmol) and isobutenyl magnesium bromide (6.2 mL, 0.44 M, 2.74 mmol) in THF (10 mL) was stirred at 23 °C for 16h. After work up, purification by flash column chromatography (5 g SiO_2, 1% EtOAc in PS 40–60) gave dendralene **2t** as a colourless liquid (115 mg, 0.475 mmol, 69%). R_f = 0.51 (5% EtOAc in PS 40–60); ^{1}H NMR (400 MHz, $CDCl_3$) δ 7.33 (d, J = 8.8 Hz, 2H), 6.83 (d, J = 8.8 Hz, 2H), 6.20 (s, 1H), 5.95 (s, 1H), 5.83 (s, 1H), 3.81 (s, 3H), 1.83 (s, 3H), 1.83 (s, 3H), 1.81 (s, 3H), 1.59 (s, 3H) ppm;
^{13}C NMR (100 MHz, $CDCl_3$) δ 158.2 (Cq), 135.8 (Cq), 135.4 (Cq), 133.9 (Cq), 131.3 (Cq), 130.3 (2×CH), 129.1 (CH), 128.9 (CH), 124.6 (CH), 113.6 (2×CH), 55.4 (CH_3), 27.1 (CH_3), 26.1 (CH_3), 19.8 (CH_3), 19.7 (CH_3) ppm; IR (thin film): ν_{max} = 2964, 2907, 2852, 1605, 1506 cm^{-1}; LRMS: (EI+): m/z (%): 242 ([M]$^{+\bullet}$, 100), 227 ([M–CH_3]$^{+\bullet}$, 91), 212 ([M–2×CH_3]$^{+\bullet}$, 20); HRMS (EI+): calculated for $C_{17}H_{22}O$: 242.1671; found: 242.1671.

(*E*)-(6-Methyl-4-(2-methylprop-1-en-1-yl)hepta-1,3,5-trien-1-yl)benzene (2u)

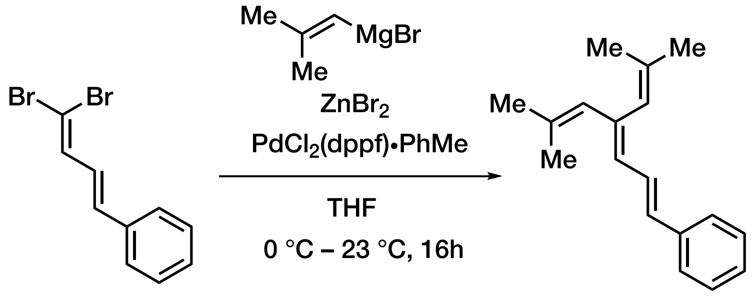

1j **2u**

Following **Standard Procedure A**, the reaction mixture containing dibromide **1j** (200 mg, 0.694 mmol), 1,1'-bis(diphenylphosphino)ferrocene palladium(II) chloride•toluene (29 mg, 0.035 mmol), $ZnBr_2$ solution in THF (2.3 mL, 1.23 M, 2.78 mmol) and isobutenyl magnesium bromide (4.7 mL, 0.44 M, 2.08 mmol) in THF (10 mL) was stirred at 23 °C for 16h. After work up, purification by flash column chromatography (5 g SiO_2, 100% PS 40–60) gave dendralene **2u** as a colourless liquid

(110 mg, 0.461 mmol, 66%). R_f = 0.23 (100% PS 40–60); ^{1}H NMR (400 MHz, $CDCl_3$) δ 7.39 (d, J = 7.6 Hz, 2H), 7.30 (t, J = 7.6 Hz, 2H), 7.19 (t, J = 7.4 Hz, 1H), 6.93 (dd, J = 15.6, 11.2 Hz, 1H), 6.52 (d, J = 15.6 Hz, 1H), 6.11 (d, J = 11.2 Hz, 1H), 5.93 (s, 1H), 5.84 (s, 1H), 1.90 (s, 3H), 1.85 (s, 3H), 1.82 (s, 3H), 1.63 (s, 3H) ppm; ^{13}C NMR (100 MHz, $CDCl_3$) δ 138.8 (Cq), 138.3 (Cq), 135.9 (Cq), 135.4 (Cq), 131.2 (CH), 130.0 (CH), 128.7 (2×CH), 128.0 (CH), 127.4 (CH), 127.2 (CH), 126.3 (2×CH), 123.6 (CH), 27.7 (CH_3), 26.0 (CH_3), 20.0 (CH_3), 19.9 (CH_3) ppm; IR (thin film): ν_{max} = 3027, 2966, 2907, 2729, 1637, 1596 cm^{-1}; LRMS: (EI+): m/z (%): 238 ($[M]^{+\bullet}$, 100), 223 ($[M–CH_3]^{+\bullet}$, 59), 208 ($[M–2×CH_3]^{+\bullet}$, 5), 181 ($[M–C_4H_9]^{+\bullet}$, 76); HRMS (EI+): calculated for $C_{18}H_{22}$: 238.1722; found: 238.1721.

((1*E*,4*E*)-3-((*E*)-3-Phenylallylidene)penta-1,4-diene-1,5-diyl)dibenzene (2v)

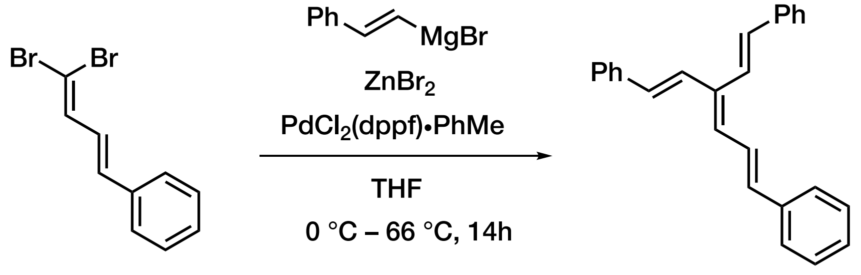

1j **2v**

Following **Standard Procedure A**, the reaction mixture containing dibromide **1j** (400 mg, 1.4 mmol), 1,1'-bis(diphenylphosphino)ferrocene palladium(II) chloride•toluene (57 mg, 0.07 mmol), $ZnBr_2$ solution in THF (4.6 mL, 1.22 M, 5.56 mmol) and styrenyl magnesium bromide (7.2 mL, 0.58 M, 4.17 mmol) in THF (20 mL) was heated under reflux for 14h. After work up, purification by flash column chromatography (15 g SiO_2, 0.5% EtOAc in PS 40–60) gave **2v** as a white solid (330 mg, 0.99 mmol, 71%). R_f = 0.38 (5% EtOAc in PS 40–60); m.p 93 – 95 °C (hexane: EtOAc, 8:2); ^{1}H NMR (700 MHz, CD_3CN) δ 7.64 (d, J = 7.4 Hz, 2H), 7.58 – 7.46 (m, 5H), 7.44 – 7.20 (m, 10H), 7.14 (d, J = 16.1 Hz, 1H), 6.95 (d, J = 8.4 Hz, 1H), 6.91 (d, J = 9.1 Hz, 1H), 6.78 (d, J = 15.4 Hz, 1H), 6.67 (d, J = 11.9 Hz, 1H) ppm; ^{13}C NMR (175 MHz, CD_3CN) δ 138.6 (Cq), 138.6 (Cq), 138.4 (Cq), 138.0 (Cq), 135.2 (CH), 133.7 (CH), 131.8 (CH), 131.1 (CH), 130.3 (CH), 129.7 (2×CH), 129.7 (2×CH), 129.6 (2×CH), 128.8 (CH), 128.7 (CH), 128.6 (CH), 127.7 (2×CH), 127.6 (2×CH), 127.5 (2×CH), 126.5 (CH), 124.9 (CH) ppm; IR (thin film): ν_{max} = 3057, 3026, 1595 cm^{-1}; LRMS: (EI+): m/z (%): 334 ($[M]^{+\bullet}$, 100), 243 ($[M–C_7H_7]^{+\bullet}$, 35); HRMS (EI+): calculated for $C_{26}H_{22}$: 334.1722; found: 334.1724.

((1*E*,4*E*)-3-(4-Methoxybenzylidene)penta-1,4-diene-1,5-diyl)dibenzene (2w)

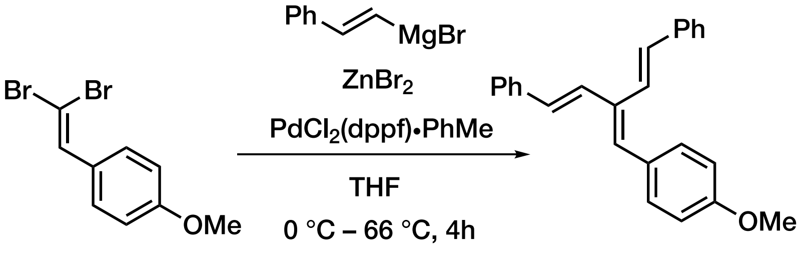

1c **2w**

Following **Standard Procedure A**, the reaction mixture containing dibromide **1c** (1.00 g, 3.43 mmol), 1,1'-bis(diphenylphosphino)ferrocene palladium(II) chloride•toluene (0.14 g, 0.17 mmol), $ZnBr_2$ solution in THF (13.9 mL, 1.23 M, 17.1 mmol) and styrenyl magnesium bromide (34.3 mL, 0.40 M, 13.7 mmol) in THF (50 mL) was heated under reflux for 4h. After work up, purification by flash column chromatography (30 g SiO_2, 1% EtOAc in PS 40–60) and recrystallization from hexane:CH_2Cl_2 (9:1) gave **2w** as a white solid (0.88 g, 2.60 mmol, 76%).

R_f = 0.38 (5% EtOAc in PS 40–60); m.p. 105 – 107 °C (hexane:CH_2Cl_2, 9:1); ^{1}H NMR (400 MHz, $CDCl_3$) δ 7.50 (d, J = 7.6 Hz, 2H), 7.46 (d, J = 7.6 Hz, 2H), 7.41 – 7.31 (m, 6H), 7.30 – 7.22 (m, 2H), 7.18 (d, J = 16.4 Hz, 1H), 7.05 (d, J = 15.6 Hz, 1H), 6.95 – 6.89 (m, 3H), 6.86 (d, J = 16.4 Hz, 1H), 6.80 (s, 1H), 3.84 (s, 3H) ppm; ^{13}C NMR (100 MHz, $CDCl_3$) δ 159.3 (Cq), 137.8 (Cq), 137.7 (Cq), 135.9 (Cq), 132.6 (CH), 131.4 (2×CH), 130.8 (CH), 130.6 (CH), 130.4 (Cq), 129.9 (CH), 128.9 (2×CH), 128.8 (2×CH), 127.8 (CH), 127.7 (CH), 126.7 (2×CH), 126.7 (2×CH), 126.6 (CH), 114.0 (2×CH), 55.5 (CH_3) ppm; IR (thin film): ν_{max} = 3057, 3024, 2989, 2837, 1597 cm^{-1}; LRMS: (EI+): m/z (%): 338 ([M]$^{+\bullet}$, 100), 247 ([M–C_7H_7]$^{+\bullet}$, 38); HRMS (EI+): calculated for $C_{25}H_{22}O$: 338.1671; found: 338.1668.

Synthesis of Monobromodienes by *E*-Selective Single Cross-Coupling

Standard Procedure B:

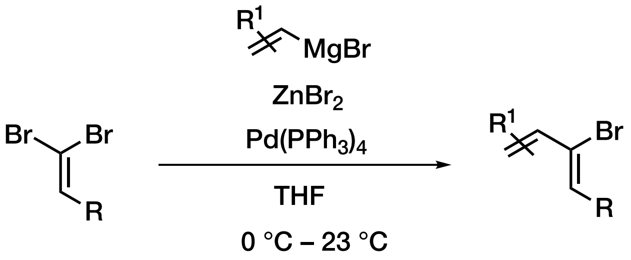

A solution of $Pd(PPh_3)_4$ (3.0 - 4.0 mol%) and dibromo olefin (1.0 mol equiv) in THF (20 mL/g dibromo olefin) was purged with N_2 for 5 minutes. The reaction mixture was cooled to 0 °C and $ZnBr_2$ (0.90 - 3.0 mol equiv, 1.10 - 1.48 M solution in THF) was added, followed by dropwise addition of Grignard reagent (0.75 - 2.0 mol equiv, 0.31 - 0.86 M solution in THF) over 5 minutes. The resulting heterogeneous reaction mixture was warmed to room temperature and stirred until complete by ^{1}H NMR/TLC analysis. Saturated aqueous NH_4Cl (5 mL/g dibromo olefin) was added, the reaction mixture was filtered through a pad of Celite and the filtrate was diluted with CH_2Cl_2 (25 mL/g dibromo olefin) and water (50 mL/g dibromo olefin). The aqueous and organic layers were separated and the aqueous layer was extracted with CH_2Cl_2 (10 mL/g dibromo olefin × 3). The combined organic layers were washed with saturated brine (20 mL/g dibromo olefin), dried over Na_2SO_4 and concentrated under reduced pressure.

(*Z*)-1-(2-Bromobuta-1,3-dien-1-yl)-4-methoxybenzene (3a)

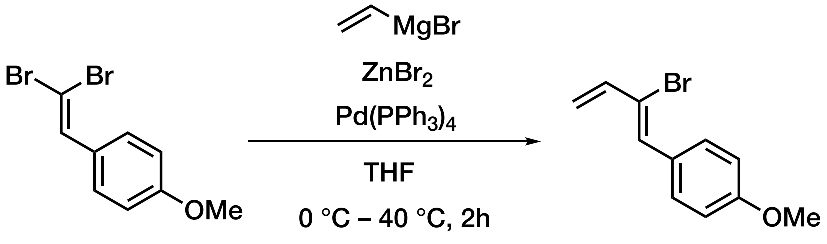

1c 3a

Following **Standard Procedure B**, the reaction mixture containing dibromide **1c** (1.00 g, 3.43 mmol), $Pd(PPh_3)_4$ (0.12 g, 0.103 mmol) $ZnBr_2$ solution in THF (4.5 mL, 1.14 M in THF, 5.14 mmol) and vinyl magnesium bromide (5.0 mL, 0.82 M in THF, 4.11 mmol) in THF (20 mL) was stirred at 40 °C for 2h. After work up, purification by flash column chromatography (25 g SiO_2, 1% EtOAc in PS 40–60) gave monobromodiene **3a** as a white solid (0.736 g, 3.07 mmol, 90%). R_f = 0.33 (5% EtOAc in PS 40–60); m.p. 51 – 52 °C (hexane:EtOAc, 9:1); ^{1}H NMR (400 MHz, $CDCl_3$) δ 7.70 (d, J = 8.8 Hz, 2H), 6.99 – 6.82 (m, 3H), 6.49 (dd, J = 16.0, 10.4 Hz, 1H), 5.68

(d, J = 16.0 Hz, 1H), 5.28 (d, J = 10.4 Hz, 1H), 3.84 (s, 3H) ppm; ^{13}C NMR (100 MHz, $CDCl_3$) δ 159.7 (Cq), 137.4 (CH), 132.0 (CH), 131.3 (2×CH), 128.2 (Cq), 122.1 (Cq), 118.1 (CH_2), 113.8 (2×CH), 55.4 (CH_3) ppm; IR (thin film): ν_{max} = 3003, 2965, 2839, 1591, 1505 cm^{-1}; LRMS: (EI+): m/z (%): 240 ([M(^{81}Br)]$^{+\bullet}$, 26), 238 ([M(^{79}Br)]$^{+\bullet}$, 26), 159 ([M–Br]$^{+\bullet}$, 60), 144 ([M–CH_3Br]$^{+\bullet}$, 86), 128 ([M–OCH_3Br]$^{+\bullet}$, 38), 115 (100); HRMS (EI+): calculated for $C_{11}H_{11}{}^{81}BrO$: 239.9973; found: 239.9961;calculated for $C_{11}H_{11}{}^{79}BrO$: 237.9993; found: 237.9997.

(*Z*)-1-(2-Bromo-3-methylbuta-1,3-dien-1-yl)-4-methoxybenzene (3b)

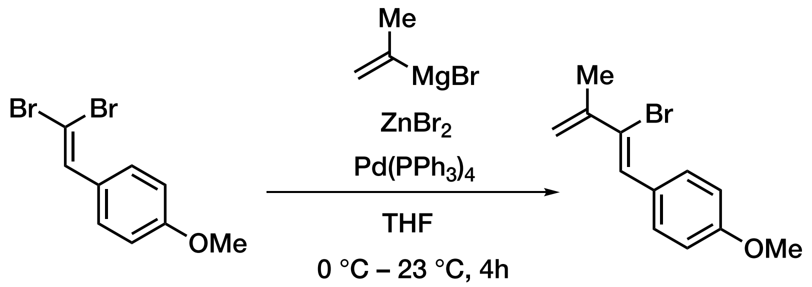

1c **3b**

Following **Standard Procedure B**, the reaction mixture containing dibromide **1c** (1.50 g, 5.14 mmol), $Pd(PPh_3)_4$ (0.180 g, 0.154 mmol) $ZnBr_2$ (4.1 mL, 1.14 M in THF, 4.62 mmol) and isopropenyl magnesium bromide (6.9 mL, 0.56 M in THF, 3.85 mmol) in THF (30 mL) was stirred at 23 °C for 4h. After work up, purification by flash column chromatography (45 g SiO_2, 1% EtOAc in PS 40–60) gave monobromodiene **3b** as a pale yellow liquid (0.650 g, 2.57 mmol, 50%). R_f = 0.41 (5% EtOAc in PS 40–60); ^{1}H NMR (400 MHz, $CDCl_3$) δ 7.63 (d, J = 8.8 Hz, 2H), 7.00 (s, 1H), 6.91 (d, J = 8.8 Hz, 2H), 5.63 (s, 1H), 5.22 (s, 1H), 3.84 (s, 3H), 2.13 (s, 3H) ppm; ^{13}C NMR (100 MHz, $CDCl_3$) δ 159.4 (Cq), 142.0 (Cq), 131.2 (2×CH), 128.9 (Cq), 128.5 (CH), 124.7 (Cq), 118.8 (CH_2), 113.6 (2×CH), 55.4 (CH_3), 21.5 (CH_3) ppm; IR (thin film): ν_{max} = 3000, 2953, 2835, 1601, 1507 cm^{-1}; LRMS: (EI+): m/z (%): 254 ([M(^{81}Br)]$^{+\bullet}$, 16), 252 ([M(^{79}Br)]$^{+\bullet}$, 16), 239 ([M(^{81}Br)–CH_3]$^{+\bullet}$, 14), 237 ([M(^{79}Br)–CH_3]$^{+\bullet}$, 14), 173 ([M–Br]$^{+\bullet}$, 46), 158 ([M–CH_3Br]$^{+\bullet}$, 100); HRMS (EI+): calculated for $C_{12}H_{13}O^{81}Br$: 254.0129; found: 254.0124; calculated for $C_{12}H_{13}O^{79}Br$: 252.0150; found: 252.0151.

1-((1*Z*,3*E*)-2-Bromo-4-phenylbuta-1,3-dien-1-yl)-4-methoxybenzene (3c)

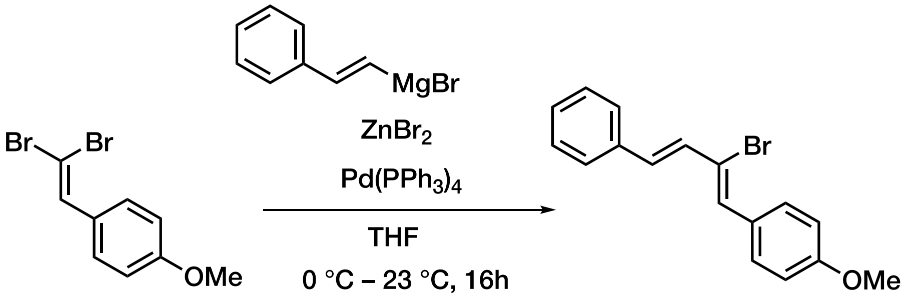

1c **3c**

Following **Standard Procedure B**, the reaction mixture containing dibromide **1c** (1.00 g, 3.43mmol), $Pd(PPh_3)_4$ (0.119 g, 0.103 mmol) $ZnBr_2$ (8.4 mL, 1.23 M in THF, 10.3 mmol) and styrenyl magnesium bromide (17.1 mL, 0.40 M in THF, 6.85 mmol) in THF (20 mL) was stirred at 23 °C for 16h. After work up, purification by flash column chromatography (25 g SiO_2, 1% EtOAc in PS 40–60) gave monobromodiene **3c** as a pale yellow solid (0.92 g, 2.93 mmol, 85%). $R_f = 0.26$ (1% EtOAc in PS 40–60); m.p. 124 –126 °C (hexane:EtOAc, 9:1); ^{1}H NMR (400 MHz, $(CD_3)_2SO$) δ 7.76 (d, J = 9.2 Hz, 2H), 7.58 (d, J = 7.6 Hz, 2H), 7.42 – 7.35 (m, 3H), 7.33 – 7.25 (m, 2H), 7.01 (d, J = 9.2 Hz, 2H), 6.93 (d, J = 15.2 Hz, 1H), 3.80 (s, 3H) ppm; ^{13}C NMR (100 MHz, $(CD_3)_2SO$) δ 159.4 (Cq), 136.2 (Cq), 132.4 (CH), 132.0 (CH), 131.0 (2×CH), 129.8 (CH), 128.9 (2×CH), 128.1 (CH), 127.8 (Cq), 126.9 (2×CH), 120.6 (Cq), 113.9 (2×CH), 55.3 (CH_3) ppm; IR (thin film): ν_{max} = 3008, 2837, 1599, 1578, 1505 cm^{-1}; LRMS: (EI+): m/z (%): 316 ([M(^{81}Br)]$^{+\bullet}$, 46), 314 ([M(^{79}Br)]$^{+\bullet}$, 46), 235 ([M–Br]$^{+\bullet}$, 100), 220 ([M–CH_3Br]$^{+\bullet}$, 81), 204 ([M–OCH_3Br]$^{+\bullet}$, 36); HRMS (EI+): calculated for $C_{17}H_{15}O^{81}Br$: 316.0286; found: 316.0276; calculated for $C_{17}H_{15}O^{79}Br$: 314.0306; found: 314.0310.

(*Z*)-1-(2-Bromo-4-methylpenta-1,3-dien-1-yl)-4-methoxybenzene (3d)

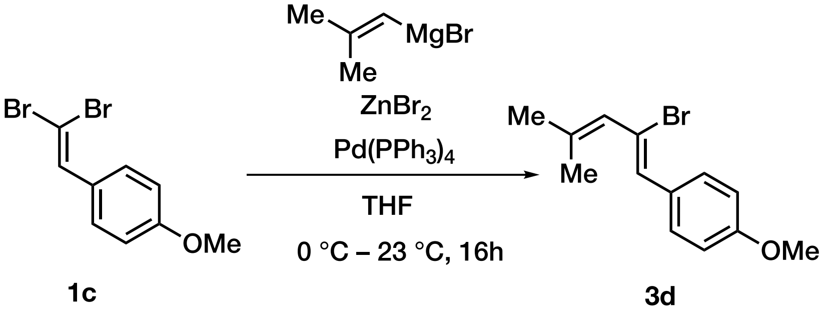

1c **3d**

Following **Standard Procedure B**, the reaction mixture containing dibromide **1c** (1.00 g, 3.43 mmol), $Pd(PPh_3)_4$ (0.119 g, 0.103 mmol) $ZnBr_2$ (7.0 mL, 1.22 M in THF, 8.56 mmol) and isobutenyl magnesium bromide (13.6 mL, 0.44 M in THF, 6.00 mmol) in THF (20 mL) was stirred at 23 °C for 16h. After work up, purification by flash column chromatography (30 g SiO_2, 1%

EtOAc in PS 40–60) gave monobromodiene **3d** as a colourless liquid (0.654 g, 2.45 mmol, 71%). R_f = 0.42 (5% EtOAc in PS 40–60); ^{1}H NMR (400 MHz, $CDCl_3$) δ 7.62 (d, J = 8.8 Hz, 2H), 6.91 (d, J = 8.8 Hz, 2H), 6.63 (s, 1H), 5.95 (s, 1H), 3.83 (s, 3H), 1.89 (d, J = 0.8 Hz, 3H), 1.86 (d, J = 1.2 Hz, 3H) ppm; ^{13}C NMR (100 MHz, $CDCl_3$) δ 159.3 (Cq), 137.9 (Cq), 130.5 (2×CH), 129.5 (CH), 128.6 (Cq), 127.6 (CH), 119.1 (Cq), 113.7 (2×CH), 55.4 (CH_3), 25.9 (CH_3), 19.7 (CH_3) ppm; IR (thin film): ν_{max} = 2966, 2932, 2835, 1606, 1508 cm^{-1}; LRMS: (EI+): m/z (%): 268 ([M(^{81}Br)]$^{+\bullet}$, 29), 266 ([M(^{79}Br)]$^{+\bullet}$, 29), 253 ([M(^{81}Br)–CH_3]$^{+\bullet}$, 13), 251 ([M(^{79}Br)–CH_3]$^{+\bullet}$, 13), 187 ([M–Br]$^{+\bullet}$, 22), 172 ([M–CH_3Br]$^{+\bullet}$, 100); HRMS (EI+): calculated for $C_{13}H_{15}O^{81}Br$: 268.0286; found: 268.0287; calculated for $C_{13}H_{15}O^{79}Br$: 266.0306; found: 266.0305.

((1*E*,3*Z*)-4-Bromohexa-1,3,5-trien-1-yl)benzene (3e)

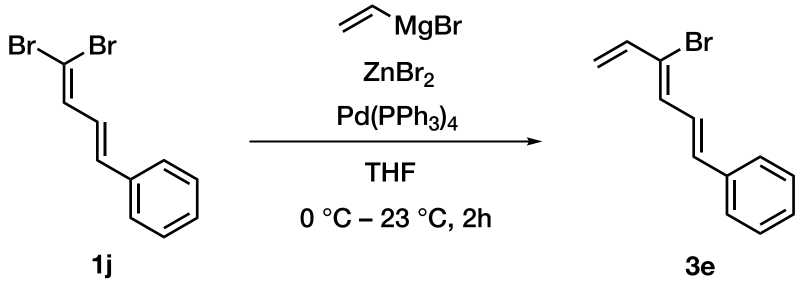

Following **Standard Procedure B**, the reaction mixture containing dibromide **1j** (500 mg, 1.74 mmol), $Pd(PPh_3)_4$ (80 mg, 0.069 mmol) $ZnBr_2$ (3.5 mL, 1.48 M in THF, 5.21 mmol) and vinyl magnesium bromide (4.4 mL, 0.86 M in THF, 3.82 mmol) in THF (10 mL) was stirred at 23 °C for 2h. After work up, purification by flash column chromatography (15 g SiO_2, 100% PS 40–60) gave monobromodiene **3e** as a white solid (325 mg, 1.38 mmol, 79%). R_f = 0.37 (100% PS 40–60); m.p. 64 – 68 °C (CH_2Cl_2); ^{1}H NMR (400 MHz, $CDCl_3$) δ 7.51 (d, J = 8.0 Hz, 2H), 7.37 (t, J = 7.4 Hz, 2H), 7.33 – 7.22 (m, 2H), 6.80 (d, J = 15.6 Hz, 1H), 6.70 (d, J = 10.4 Hz, 1H), 6.46 (dd, J = 16.2, 10.4 Hz, 1H), 5.70 (d, J = 16.2 Hz, 1H), 5.31 (d, J = 10.4 Hz, 1H) ppm; ^{13}C NMR (100 MHz, $CDCl_3$) δ 137.0 (Cq), 136.8 (CH), 136.1 (CH), 132.7 (CH), 128.9 (2×CH), 128.5 (CH), 127.0 (2×CH), 126.7 (CH), 126.1 (Cq), 119.0 (CH_2) ppm; IR (thin film): ν_{max} = 3032, 2917, 1824, 1599 cm^{-1}; LRMS: (EI+): m/z (%): 236 ([M(^{81}Br)]$^{+\bullet}$, 26), 234 ([M(^{79}Br)]$^{+\bullet}$, 26), 155 ([M–Br]$^{+\bullet}$, 100), 154 ([M–HBr]$^{+\bullet}$, 30); HRMS (EI+): calculated for $C_{12}H_{11}{}^{81}Br$: 236.0024; found: 236.0020; calculated for $C_{12}H_{11}{}^{79}Br$: 234.0044; found: 234.0050.

((1*E*,3*Z*)-4-Bromo-5-methylhexa-1,3,5-trien-1-yl)benzene (3f)

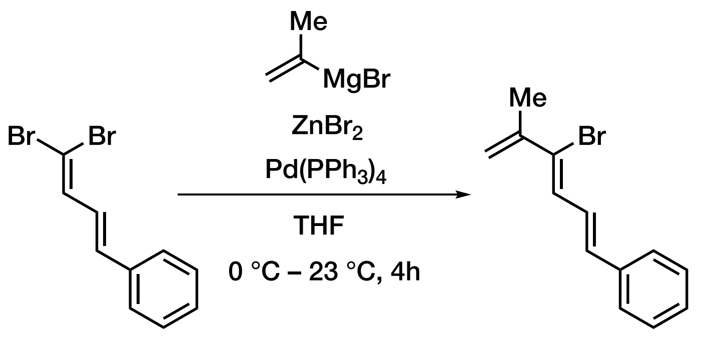

Following **Standard Procedure B**, the reaction mixture containing dibromide **1j** (2.00 g, 6.94 mmol), $Pd(PPh_3)_4$ (241 mg, 0.208 mmol) $ZnBr_2$ (9.0 mL, 1.23 M in THF, 11.1 mmol) and isopropenyl magnesium bromide (14.9 mL, 0.56 M in THF, 8.33 mmol) in THF (40 mL) was stirred at 23 °C for 4h. After work up, purification by flash column chromatography (50 g SiO_2, 100% PS 40–60) gave monobromodiene **3f** as a white solid (1.41 g, 5.66 mmol, 82%). R_f = 0.38 (100% PS 40–60); m.p. 68 – 69 °C (hexane); ^{1}H NMR (400 MHz, $CDCl_3$) δ 7.49 (d, J = 7.2 Hz, 2H), 7.35 (t, J = 7.4 Hz, 2H), 7.30 – 7.24 (m, 2H), 6.80 (d, J = 16.0 Hz, 1H), 6.74 (d, J = 10.4 Hz, 1H), 5.64 (s, 1H), 5.22 (s, 1H), 2.09 (s, 3H) ppm; ^{13}C NMR (100 MHz, $CDCl_3$) δ 140.8 (Cq), 137.1 (Cq), 136.6 (CH), 129.2 (CH), 128.9 (2×CH), 128.4 (CH), 127.8 (Cq), 127.8 (CH), 127.0 (2×CH), 119.5 (CH_2), 21.1 (CH_3) ppm; IR (thin film): ν_{max} = 3037, 2956, 1792, 1598 cm^{-1}; LRMS: (EI+): m/z (%): 250 ([M(^{81}Br)]$^{+\bullet}$, 31), 248 ([M(^{79}Br)]$^{+\bullet}$, 31), 169 ([M–Br]$^{+\bullet}$, 100), 154 ([M– CH_3Br]$^{+\bullet}$, 73); HRMS (EI+): calculated for $C_{13}H_{13}{}^{81}$Br: 250.0180; found: 250.0188; calculated for $C_{13}H_{13}{}^{79}$Br: 248.0201; found: 248.0203.

((1*E*,3*Z*,5*E*)-3-Bromohexa-1,3,5-triene-1,6-diyl)dibenzene (3g)

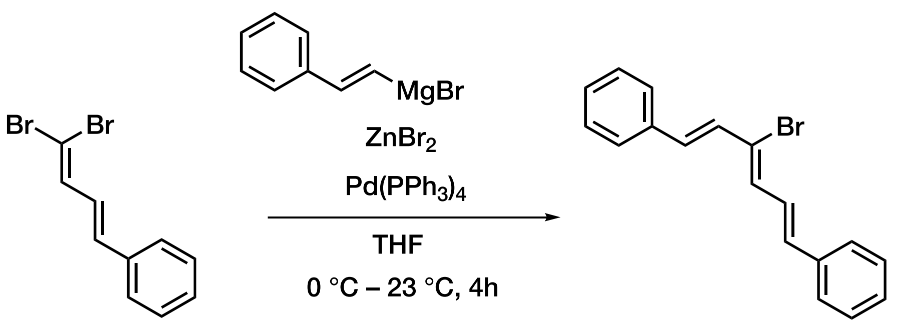

Following **Standard Procedure B**, the reaction mixture containing dibromide **1j** (500 mg, 1.74 mmol), $Pd(PPh_3)_4$ (60 mg, 0.052 mmol) $ZnBr_2$ (3.3 mL, 1.22 M in THF, 4.0 mmol) and styrenyl magnesium bromide (4.8 mL, 0.58 M in THF, 2.78 mmol) in THF (10 mL) was stirred at 23 °C for 4h. After work up, purification by flash column chromatography (15 g SiO_2, 100% PS 40–60) gave

monobromodiene **3g** as a pale yellow solid (398 mg, 1.28 mmol, 74%). R_f = 0.23 (100% PS 40–60); m.p. 142 –144 °C (hexane:CH_2Cl_2, 9:1); ^1H NMR (400 MHz, $CDCl_3$) δ 7.56 – 7.48 (m, 4H), 7.42 – 7.36 (m, 4H), 7.36 – 7.27 (m, 3H), 7.07 (d, J = 15.1 Hz, 1H), 6.90 (d, J = 15.1 Hz, 1H), 6.83 (d, J = 11.2 Hz, 1H), 6.80 (d, J = 6.8 Hz, 1H) ppm; ^{13}C NMR (100 MHz, $CDCl_3$) δ 137.1 (Cq), 136.7 (Cq), 136.3 (CH), 133.9 (CH), 132.7 (CH), 128.9 (4×CH), 128.5 (CH), 128.3 (CH), 128.1 (CH), 127.1 (2×CH), 127.0 (CH), 127.0 (2×CH), 125.7 (Cq) ppm; IR (thin film): ν_{max} = 3027, 1621, 1593 cm^{-1}; LRMS: (EI+): m/z (%): 312 ($[M(^{81}Br)]^{+\bullet}$, 60), 310 ($[M(^{79}Br)]^{+\bullet}$, 60), 231 ($[M–Br]^{+\bullet}$, 100); HRMS (EI+): calculated for $C_{18}H_{15}{}^{81}Br$: 312.0337; found: 312.0333; calculated for $C_{18}H_{15}{}^{79}Br$: 310.0357; found: 310.0357.

((1*E*,3*Z*)-4-Bromo-6-methylhepta-1,3,5-trien-1-yl)benzene (3h)

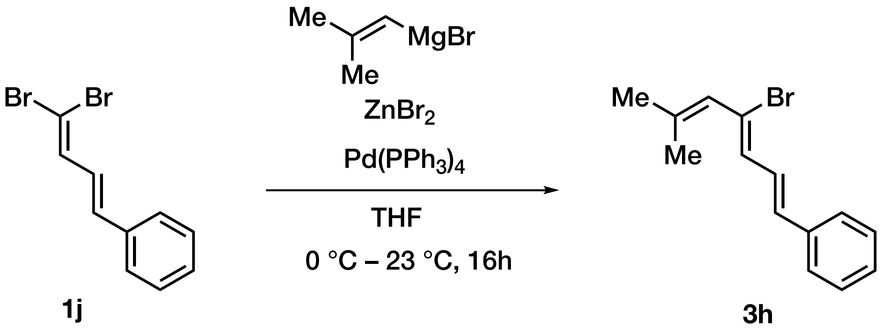

Following **Standard Procedure B**, the reaction mixture containing dibromide **1j** (500 mg, 1.74 mmol), $Pd(PPh_3)_4$ (60 mg, 0.052 mmol) $ZnBr_2$ (3.6 mL, 1.22 M in THF, 4.34 mmol) and isobutenyl magnesium bromide (7.5 mL, 0.44 M in THF, 3.30 mmol) in THF (10 mL) was stirred at 23 °C for 16h. After work up, purification by flash column chromatography (15 g SiO_2, 100% PS 40–60) gave monobromodiene **3h** as a colourless liquid (195 mg, 0.741 mmol, 43%).

R_f = 0.29 (100% PS 40–60); ^1H NMR (400 MHz, $CDCl_3$) δ 7.49 (d, J = 7.4 Hz, 2H), 7.36 (t, J = 7.5 Hz, 2H), 7.31 – 7.24 (m, 1H), 7.14 (dd, J = 15.7, 10.2 Hz, 1H), 6.73 (d, J = 15.7 Hz, 1H), 6.43 (d, J = 10.2 Hz, 1H), 5.95 (s, 1H), 1.92 (s, 3H), 1.88 (s, 3H) ppm; ^{13}C NMR (100 MHz, $CDCl_3$) δ 138.9 (Cq), 137.3 (Cq), 134.7 (CH), 130.3 (CH), 128.8 (2×CH), 128.1 (CH), 127.3 (CH), 126.8 (2×CH), 126.6 (CH), 123.3 (Cq), 26.3 (CH_3), 20.0 (CH_3) ppm; IR (thin film): ν_{max} = 3035, 2970, 2909, 1633 cm^{-1}; LRMS: (EI+): m/z (%): 264 ($[M(^{81}Br)]^{+\bullet}$, 61), 262 ($[M(^{79}Br)]^{+\bullet}$, 61), 183 ($[M–Br]^{+\bullet}$, 100), 168 ($[M–CH_3Br]^{+\bullet}$, 95), 153 ($[M–CH_3CH_3Br]^{+\bullet}$, 47); HRMS (EI+): calculated for $C_{14}H_{15}{}^{81}Br$: 264.0337; found: 264.0349; calculated for $C_{14}H_{15}{}^{79}Br$: 262.0357; found: 262.0358.

(*Z*)-(4-Bromohexa-3,5-dien-1-yn-1-yl)benzene (3i)

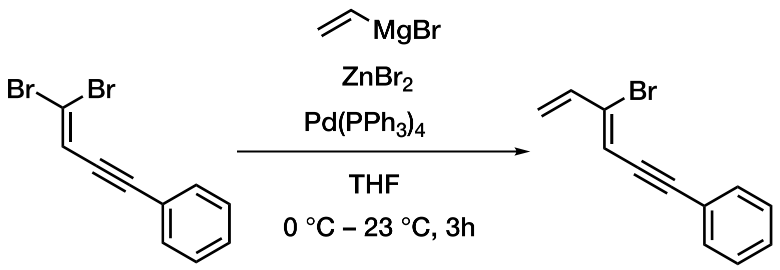

1k **3i**

Following **Standard Procedure B**, the reaction mixture containing dibromide **1k** (510 mg, 1.78 mmol), $Pd(PPh_3)_4$ (62 mg, 0.054 mmol) $ZnBr_2$ (2.2 mL, 1.23 M in THF, 2.68 mmol) and vinyl magnesium bromide (3.0 mL, 0.71 M in THF, 2.14 mmol) in THF (10 mL) was stirred at 23 °C for 3h. After work up, purification by flash column chromatography (15 g SiO_2, 100% PS 40–60) gave monobromodiene **3i** as a pale yellow liquid (320 mg, 1.37 mmol, 77%). R_f = 0.20 (100% PS 40–60); ^{1}H NMR (400 MHz, $CDCl_3$) δ 7.59 – 7.48 (m, 2H), 7.41 – 7.30 (m, 3H), 6.45 (dd, J = 16.4, 10.4 Hz, 1H), 6.33 (s, 1H), 5.74 (d, J = 16.4 Hz, 1H), 5.40 (d, J = 10.4 Hz, 1H) ppm; ^{13}C NMR (100 MHz, $CDCl_3$) δ 135.0 (CH), 134.8 (Cq), 131.8 (2×CH), 128.9 (CH), 128.5 (2×CH), 123.1 (Cq), 121.8 (CH_2), 114.1 (CH), 99.2 (Cq), 87.6 (Cq) ppm; IR (thin film): ν_{max} = 3054, 3001, 2193, 1613, 1597, 1568 cm^{-1}; LRMS: (EI+): m/z (%): 234 ([M(^{81}Br)]$^{+\bullet}$, 63), 232 ([M(^{79}Br)]$^{+\bullet}$, 63), 153 ([M–Br]$^{+\bullet}$, 76), 152 ([M–HBr]$^{+\bullet}$, 100), 151 ([M–HBrH]$^{+\bullet}$, 43); HRMS (EI+): calculated for $C_{12}H_9{}^{81}Br$: 233.9867; found: 233.9860; calculated for $C_{12}H_9{}^{79}Br$: 231.9888; found: 231.9888.

(*Z*)-(4-Bromo-5-methylhexa-3,5-dien-1-yn-1-yl)benzene (3j)

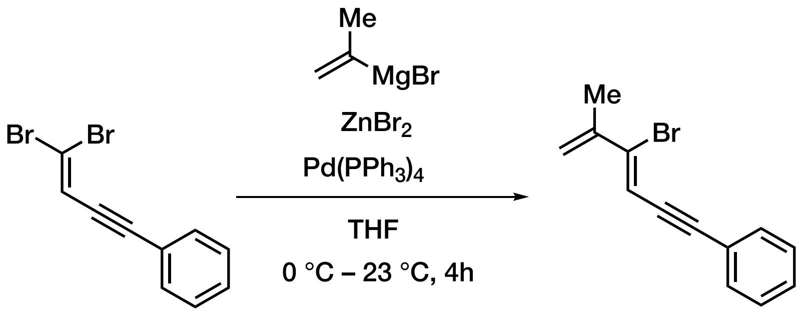

1k **3j**

Following **Standard Procedure B**, the reaction mixture containing dibromide **1k** (970 mg, 3.40 mmol), $Pd(PPh_3)_4$ (118 mg, 0.102 mmol) $ZnBr_2$ (4.1 mL, 1.23 M in THF, 5.09 mmol) and isopropenyl magnesium bromide (8.0 mL, 0.51 M in THF, 4.07 mmol) in THF (20 mL) was stirred at 23 °C for 4h. After work up, purification by flash column chromatography (25 g SiO_2, 100% PS 40–60) gave monobromodiene **3j** as a pale yellow liquid (675 mg, 2.73 mmol, 81%).

R_f = 0.28 (100% PS 40–60); ^{1}H NMR (400 MHz, $CDCl_3$) δ 7.58 – 7.48 (m, 2H), 7.36 – 7.31 (m, 3H), 6.39 (s, 1H), 5.68 (s, 1H), 5.29 (s, 1H), 2.06 (s, 3H) ppm; ^{13}C NMR (100 MHz, $CDCl_3$) δ 140.2 (Cq), 136.4 (Cq), 131.8 (2×CH), 128.8 (CH), 128.5 (2×CH), 123.2 (Cq), 121.5 (CH_2), 111.4 (CH), 98.5 (Cq), 88.1 (Cq), 20.5 (CH_3) ppm; IR (thin film): ν_{max} = 3025, 2923, 2197. 1599 cm^{-1}; LRMS: (EI+): m/z (%): 248 ($[M(^{81}Br)]^{+\bullet}$, 31), 246 ($[M(^{79}Br)]^{+\bullet}$, 31), 167 ($[M–Br]^{+\bullet}$, 90), 166 ($[M–HBr]^{+\bullet}$, 36), 165 ($[M–HBrH]^{+\bullet}$, 100), 152 ($[M–CH_3Br]^{+\bullet}$, 70), 126 ($[M–C_3H_5Br]^{+\bullet}$, 13); HRMS (EI+): calculated for $C_{13}H_{11}{}^{81}Br$: 248.0024; found: 248.0024; calculated for $C_{13}H_{11}{}^{79}Br$: 246.0044; found: 246.0043.

((1*E*,3*Z*)-3-Bromohexa-1,3-dien-5-yne-1,6-diyl)dibenzene (3k)

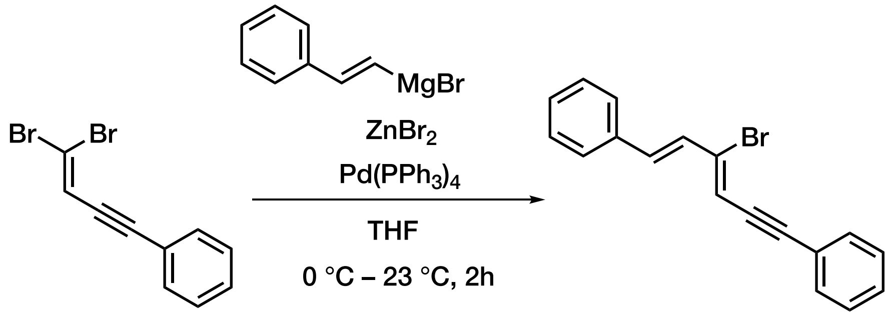

1k **3k**

Following **Standard Procedure B**, the reaction mixture containing dibromide **1k** (460 mg, 1.61 mmol), $Pd(PPh_3)_4$ (56 mg, 0.048 mmol) $ZnBr_2$ (3.7 mL, 1.22 M in THF, 4.50 mmol) and styrenyl magnesium bromide (6.4 mL, 0.58 M in THF, 3.70 mmol) in THF (10 mL) was stirred at 23 °C for 2h. After work up, purification by flash column chromatography (10 g SiO_2, 100% PS 40–60) gave monobromodiene **3k** as a white solid (438 mg, 1.42 mmol, 88%). R_f = 0.25 (100% PS 40–60); m.p. 139 – 141 °C (hexane:CH_2Cl_2, 9:1); ^{1}H NMR (400 MHz, $CDCl_3$) δ 7.60 – 7.52 (m, 2H), 7.49 (d, J = 7.3 Hz, 2H), 7.43 – 7.27 (m, 6H), 7.10 (d, J = 14.8 Hz, 1H), 6.87 (d, J = 14.8 Hz, 1H), 6.41 (s, 1H) ppm; ^{13}C NMR (100 MHz, $CDCl_3$) δ 136.7 (CH), 136.1 (Cq), 134.3 (Cq), 131.8 (2×CH), 129.0 (2×CH), 128.9 (CH), 128.8 (CH), 128.5 (2×CH), 127.4 (2×CH), 126.7 (CH), 123.2 (Cq), 113.5 (CH), 99.6 (Cq), 88.4 (Cq) ppm; IR (thin film): ν_{max} = 3050, 3024, 2190, 1595, 1573, 1554 cm^{-1}; LRMS: (EI+): m/z (%): 310 ($[M(^{81}Br)]^{+\bullet}$, 16), 308 ($[M(^{79}Br)]^{+\bullet}$, 16), 229 ($[M–Br]^{+\bullet}$, 100), 152 ($[M–C_6H_5Br]^{+\bullet}$, 86); HRMS (EI+): calculated for $C_{18}H_{13}{}^{81}Br$: 310.0180; found: 310.0180; calculated for $C_{18}H_{13}{}^{79}Br$: 308.0201; found: 308.0204.

(*Z*)-(4-Bromo-6-methylhepta-3,5-dien-1-yn-1-yl)benzene (3l)

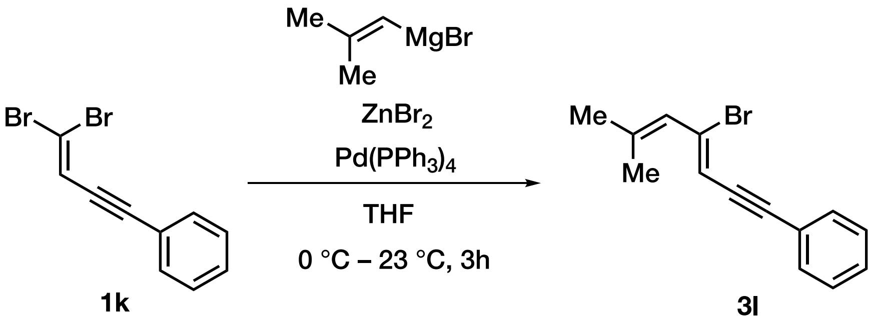

1k 3l

Following **Standard Procedure B**, the reaction mixture containing dibromide **1k** (410 mg, 1.43 mmol), $Pd(PPh_3)_4$ (50 mg, 0.0430 mmol) $ZnBr_2$ (2.9 mL, 1.22 M in THF, 3.58 mmol) and isobutenyl magnesium bromide (5.7 mL, 0.50 M in THF, 2.87 mmol) in THF (10 mL) was stirred at 23 °C for 3h. After work up, purification by flash column chromatography (20 g SiO_2, 100% PS 40–60) gave monobromodiene **3l** as a colourless liquid (230 mg, 0.881 mmol, 62%). R_f = 0.27 (100% PS 40–60); ^{1}H NMR (400 MHz, $CDCl_3$) δ 7.54 – 7.45 (m, 2H), 7.36 – 7.29 (m, 3H), 6.10 (s, 1H), 5.93 (brs, 1H), 1.91 (d, J = 1.2 Hz, 3H), 1.86 (d, J = 1.2 Hz, 3H) ppm; ^{13}C NMR (100 MHz, $CDCl_3$) δ 140.7 (Cq), 132.1 (Cq), 131.7 (2×CH), 128.6 (CH), 128.5 (2×CH), 125.7 (CH), 123.3 (Cq), 112.4 (CH), 96.3 (Cq), 87.6 (Cq), 26.5 (CH_3), 20.0 (CH_3) ppm; IR (thin film): ν_{max} = 2971, 2909, 2198, 1633 cm^{-1}; LRMS: (EI+): m/z (%): 262 ([M(^{81}Br)]$^{+\bullet}$, 21), 260 ([M(^{79}Br)]$^{+\bullet}$, 21), 181 ([M–Br]$^{+\bullet}$, 34), 166 ([M–CH_3Br]$^{+\bullet}$, 72), 165 ([M–CH_3HBr]$^{+\bullet}$, 100); HRMS (EI+): calculated for $C_{14}H_{13}{}^{81}Br$: 262.0180; found: 262.0177; calculated for $C_{14}H_{13}{}^{79}Br$: 260.0201; found: 260.0204.

(*Z*)-(2-Bromobuta-1,3-dien-1-yl)cyclohexane (3m)

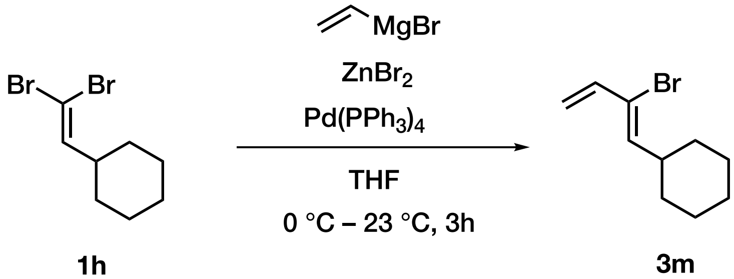

1h 3m

Following **Standard Procedure B**, the reaction mixture containing dibromide **1h** (960 mg, 3.58 mmol), $Pd(PPh_3)_4$ (124 mg, 0.107 mmol) $ZnBr_2$ (6.2 mL, 1.22 M in THF, 7.52 mmol) and vinyl magnesium bromide (7.2 mL, 0.75 M in THF, 5.37 mmol) in THF (20 mL) was stirred at 23 °C for 3h. After work up, purification by flash column chromatography (30 g $AgNO_3$ impregnated silica, 1% EtOAc in PS 40–60) gave monobromodiene **3m** as a colourless liquid (506 mg, 2.35 mmol, 66%). R_f = 0.56 (100% PS 40–60); ^{1}H NMR (400 MHz, $CDCl_3$) δ 6.28 (dd, J = 16.3, 10.5 Hz, 1H),

5.81 (d, J = 8.8 Hz, 1H), 5.53 (d, J = 16.3 Hz, 1H), 5.16 (d, J = 10.4 Hz, 1H), 2.66 – 2.49 (m, 1H), 1.82 – 1.59 (m, 5H), 1.42 – 1.01 (m, 5H) ppm; ^{13}C NMR (100 MHz, $CDCl_3$) δ 140.4 (CH), 136.2 (CH), 123.9 (Cq), 117.4 (CH_2), 40.8 (CH), 31.8 (2×CH_2), 26.1 (CH_2), 25.7 (2×CH_2) ppm; IR (thin film): ν_{max} = 2924, 2850, 1631 cm^{-1}; LRMS: (EI+): m/z (%): 216 ([M(^{81}Br)]$^{+\bullet}$, 26), 214 ([M(^{79}Br)]$^{+\bullet}$, 26), 135 ([M–Br]$^{+\bullet}$, 54), 67 (100); HRMS (EI+): calculated for $C_{10}H_{15}{}^{81}Br$: 216.0337; found: 216.0337; calculated for $C_{10}H_{15}{}^{79}Br$: 214.0357; found: 214.0362.

(*Z*)-(2-Bromo-3-methylbuta-1,3-dien-1-yl)cyclohexane (3n)

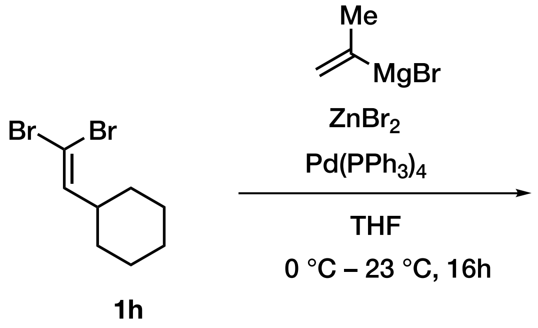

Following **Standard Procedure B**, the reaction mixture containing dibromide **1h** (920 mg, 3.43 mmol), $Pd(PPh_3)_4$ (119 mg, 0.103 mmol) $ZnBr_2$ (6.5 mL, 1.22 M in THF, 7.90 mmol) and isopropenyl magnesium bromide (11.9 mL, 0.46 M in THF, 5.50 mmol) in THF (20 mL) was stirred at 23 °C for 16h. After work up, purification by flash column chromatography (25 g $AgNO_3$ impregnated silica, 100% PS 40–60) gave monobromodiene **3n** as a colourless liquid (536 mg, 2.34 mmol, 68%). R_f = 0.63 (100% PS 40–60); ^{1}H NMR (400 MHz, $CDCl_3$) δ 5.82 (d, J = 8.8 Hz, 1H), 5.46 (s, 1H), 5.08 (s, 1H), 2.65 – 2.44 (m, 1H), 1.98 (s, 3H), 1.85 – 1.58 (m, 5H), 1.44 – 1.02 (m, 5H) ppm; ^{13}C NMR (100 MHz, $CDCl_3$) δ 141.1 (Cq), 136.5 (CH), 125.8 (Cq), 117.8 (CH_2), 41.6 (CH), 32.0 (2×CH_2), 26.1 (CH_2), 25.8 (2×CH_2), 21.2 (CH_3) ppm; IR (thin film): ν_{max} = 2924, 2850, 1624, 1605 cm^{-1}; LRMS: (EI+): m/z (%): 230 ([M(^{81}Br)]$^{+\bullet}$, 30), 228 ([M(^{79}Br)]$^{+\bullet}$, 30), 215 ([M(^{81}Br)–CH_3]$^{+\bullet}$, 8), 213 ([M(^{79}Br)–CH_3]$^{+\bullet}$, 8), 149 ([M–Br]$^{+\bullet}$, 74), 120 ([M–C_2H_5Br]$^{+\bullet}$, 11), 32 (100); HRMS (EI+): calculated for $C_{11}H_{17}{}^{81}Br$: 230.0493; found: 230.0496; calculated for $C_{11}H_{17}{}^{79}Br$: 228.0514; found: 228.0514.

(*Z*)-3-Bromotetradeca-1,3-diene (3o)

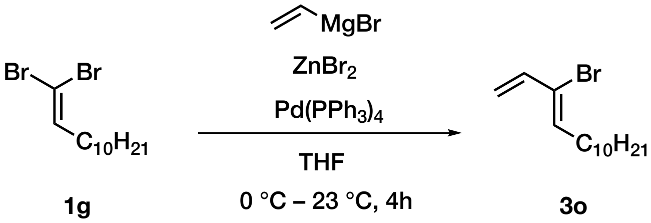

Following **Standard Procedure B**, the reaction mixture containing dibromide **1g** (677 mg, 2.08 mmol), $Pd(PPh_3)_4$ (72 mg, 0.062 mmol) $ZnBr_2$ (3.9 mL, 1.22 M in THF, 4.80 mmol) and vinyl magnesium bromide (4.7 mL, 0.75 M in THF, 3.53 mmol) in THF (15 mL) was stirred at 23 °C for 4h. After work up, purification by flash column chromatography (20 g $AgNO_3$ impregnated silica, 100% PS 40–60) gave monobromodiene **3o** as a colourless liquid (260 mg, 0.951 mmol, 46%). R_f = 0.60 (100% PS 40–60); ^{1}H NMR (400 MHz, $CDCl_3$) δ 6.31 (dd, J = 16.3, 10.4 Hz, 1H), 5.98 (t, J = 7.2 Hz, 1H), 5.53 (d, J = 16.4 Hz, 1H), 5.15 (d, J = 10.4 Hz, 1H), 2.31 (q, J = 7.3 Hz, 2H), 1.50 – 1.09 (m, 16H), 0.88 (t, J = 6.8 Hz, 3H) ppm; ^{13}C NMR (100 MHz, $CDCl_3$) δ 136.0 (CH), 135.4 (CH), 125.9 (Cq), 117.2 (CH_2), 32.1 (CH_2), 31.7 (CH_2), 29.8 (CH_2), 29.7 (CH_2), 29.6 (CH_2), 29.5 (CH_2), 29.4 (CH_2), 28.5 (CH_2), 22.9 (CH_2), 14.3 (CH_3) ppm; IR (thin film): ν_{max} = 2955, 2923, 2853, 1632 cm^{-1}; LRMS: (EI+): m/z (%): 274 ([M(^{81}Br)]$^{+\bullet}$, 12), 272 ([M(^{79}Br)]$^{+\bullet}$, 12), 193 ([M–Br]$^{+\bullet}$, 16), 81 (100); HRMS (EI+): calculated for $C_{14}H_{25}{}^{81}Br$: 274.1119; found: 274.1123; calculated for $C_{14}H_{25}{}^{79}Br$: 272.1140; found: 272.1149.

(*Z*)-3-Bromo-2-methyltetradeca-1,3-diene (3p)

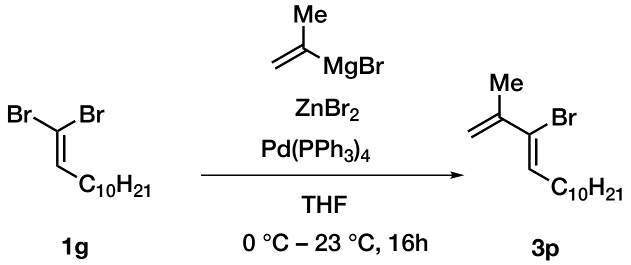

Following **Standard Procedure B**, the reaction mixture containing dibromide **1g** (780 mg, 2.39 mmol), $Pd(PPh_3)_4$ (83 mg, 0.072 mmol) $ZnBr_2$ (3.9 mL, 1.22 M in THF, 4.78 mmol) and isopropenyl magnesium bromide (6.8 mL, 0.49 M in THF, 3.35 mmol) in THF (15 mL) was stirred at 23 °C for 16h. After work up, purification by flash column chromatography (25 g $AgNO_3$ impregnated silica, 100% PS 40–60) gave monobromodiene **3p** as a colourless liquid (382 mg, 1.33 mmol, 55%). R_f = 0.60 (100% PS 40–60); ^{1}H NMR (400 MHz, $CDCl_3$) δ 6.01 (t, J = 6.8 Hz, 1H), 5.46 (s, 1H), 5.08 (s, 1H), 2.31 (q, J = 7.2 Hz, 2H), 1.99 (s, 3H), 1.51 – 1.17 (m, 16H), 0.87 (t, J = 6.8 Hz, 3H) ppm; ^{13}C NMR (100 MHz, $CDCl_3$) δ 141.0 (Cq), 131.6 (CH), 127.7 (Cq), 117.6 (CH_2), 32.6 (CH_2), 32.1 (CH_2), 29.8 (CH_2), 29.7 (CH_2), 29.6 (CH_2), 29.5 (2×CH_2), 28.6 (CH_2), 22.9 (CH_2), 21.2 (CH_3), 14.3(CH_3) ppm; IR (thin film): ν_{max} = 2922, 2853, 1625, 1606 cm^{-1}; LRMS: (EI+): m/z (%): 288 ([M(^{81}Br)]$^{+\bullet}$, 20), 286 ([M(^{79}Br)]$^{+\bullet}$, 20), 207 ([M–Br]$^{+\bullet}$, 10), 79 (100); HRMS (EI+): calculated for $C_{15}H_{27}{}^{81}Br$: 288.1276; found: 288.1267; calculated for $C_{15}H_{27}{}^{79}Br$: 286.1296; found: 286.1297.

(*Z*)-1-(2-Bromobuta-1,3-dien-1-yl)-4-nitrobenzene (3q)

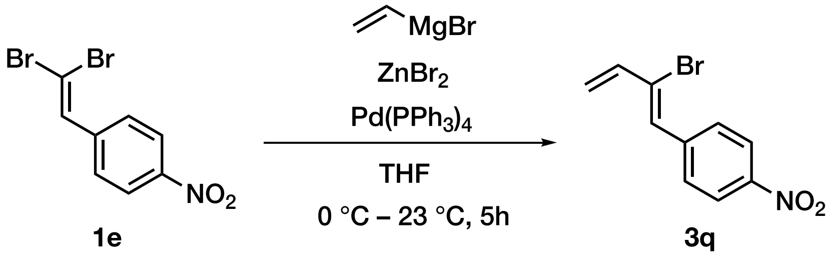

Following **Standard Procedure B**, the reaction mixture containing dibromide **1e** (850 mg, 2.77 mmol), $Pd(PPh_3)_4$ (96 mg, 0.083 mmol) $ZnBr_2$ (4.5 mL, 1.22 M in THF, 5.54 mmol) and vinyl magnesium bromide (5.2 mL, 0.75 M in THF, 3.88 mmol) in THF (20 mL) was stirred at 23 °C for 5h. After work up, purification by flash column chromatography (30 g SiO_2, 1% EtOAc in PS 40–60) gave monobromodiene **3q** as a pale yellow fluffy solid (449 mg, 1.77 mmol, 64%). R_f = 0.33 (5% EtOAc in PS 40–60); ^{1}H NMR (400 MHz, $CDCl_3$) δ 8.23 (d, J = 8.8 Hz, 2H), 7.83 (d, J = 8.8 Hz, 2H), 7.03 (s, 1H), 6.53 (dd, J = 16.2, 10.4 Hz, 1H), 5.85 (d, J = 16.2 Hz, 1H), 5.48 (d, J = 10.4 Hz, 1H) ppm; ^{13}C NMR (100 MHz, $CDCl_3$) δ 147.2 (Cq), 142.2 (Cq), 136.7 (CH), 130.3 (2×CH), 130.1 (CH), 127.7 (Cq), 123.6 (2×CH), 121.7 (CH_2) ppm; IR (thin film): ν_{max} = 3101, 3082, 2924, 1831, 1590, 1504 cm^{-1}; LRMS: (EI+): m/z (%): 255 ([M(^{81}Br)]$^{+\bullet}$, 4), 253 ([M(^{79}Br)]$^{+\bullet}$, 4), 174 ([M–Br]$^{+\bullet}$, 4), 128 ([M–NO_2Br]$^{+\bullet}$, 100); HRMS (EI+): calculated for $C_{10}H_8NO_2{}^{81}Br$: 254.9718; found: 254.9716; calculated for $C_{10}H_8NO_2{}^{79}Br$: 252.9738; found: 252.9738.

(*Z*)-2-(2-Bromo-3-methylbuta-1,3-dien-1-yl)-5-methylfuran (3r)

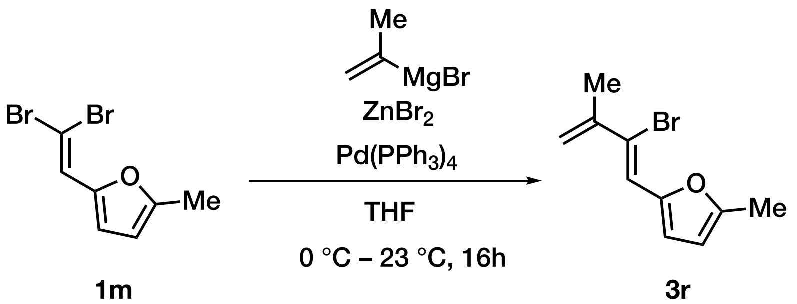

Following **Standard Procedure B**, the reaction mixture containing dibromide **1m** (230 mg, 0.865 mmol), $Pd(PPh_3)_4$ (30 mg, 0.026 mmol) $ZnBr_2$ (1.1 mL, 1.23 M in THF, 1.30 mmol) and isopropenyl magnesium bromide (1.85 mL, 0.56 M in THF, 1.04 mmol) in THF (5 mL) was stirred at 23 °C for 16h. After work up, purification by flash column chromatography (10 g SiO_2, 100% PS 40–60) gave monobromodiene **3r** as a colourless liquid (145 mg, 0.638 mmol, 74%).

R_f = 0.33 (100% PS 40–60); ^{1}H NMR (400 MHz, $CDCl_3$) δ 7.13 (d, J = 3.4 Hz, 1H), 6.98 (s, 1H), 6.11 (d, J = 3.4 Hz, 1H), 5.60 (s, 1H), 5.19 (s, 1H), 2.32 (s, 3H), 2.09 (s, 3H) ppm;
^{13}C NMR (100 MHz, $CDCl_3$) δ 152.7 (Cq), 150.2 (Cq), 141.2 (Cq), 121.8 (Cq), 118.8 (CH_2), 118.7 (CH), 113.8 (CH), 108.4 (CH), 21.1 (CH_3), 13.9 (CH_3) ppm; IR (thin film): ν_{max} = 2953, 2922, 1609, 1588, 1519 cm^{-1}; LRMS: (EI+): m/z (%): 228 ([M(^{81}Br)]$^{+\bullet}$, 50), 226 ([M(^{79}Br)]$^{+\bullet}$, 50), 213 ([M(^{81}Br)–CH_3]$^{+\bullet}$, 5), 211 ([M(^{79}Br)–CH_3]$^{+\bullet}$, 13), 147 ([M–Br]$^{+\bullet}$, 100), 132 ([M–CH_3Br]$^{+\bullet}$, 50); HRMS (EI+): calculated for $C_{10}H_{11}O^{81}Br$: 227.9973; found: 227.9980; calculated for $C_{10}H_{11}O^{79}Br$: 225.9993; found: 225.9995.

Stereoselective Synthesis of Dendralenes

Standard Procedure C: Second Cross-Coupling with Pd(0)/*t*-Bu$_3$P (Retention)

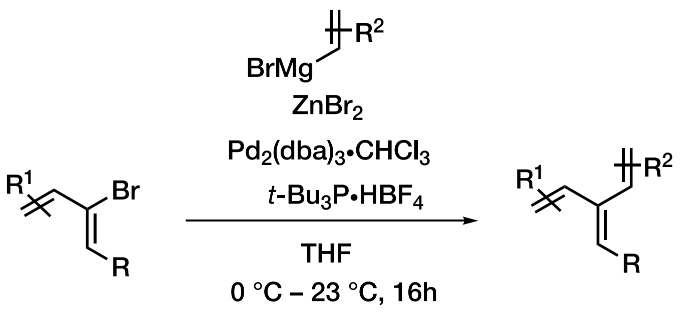

A solution of $Pd_2(dba)_3$•$CHCl_3$ (3.0 – 4.0 mol%), *t*-Bu$_3$P•HBF$_4$ (6.0 – 8.0 mol%) and monobromodiene (1.0 mol equiv) in THF (50 mL/g monobromodiene precursor) was purged with N_2 for 5 minutes. The reaction mixture was cooled to 0 °C and $ZnBr_2$ (4.0 mol equiv, 1.22 – 1.23 M solution in THF) was added, followed by dropwise addition of the Grignard reagent (3.0 mol equiv, 0.44 – 0.70 M solution in THF) dropwise over 5 minutes. The resulting heterogeneous reaction mixture was warmed to room temperature and stirred for 16h until complete by ^{1}H NMR analysis. Saturated aqueous NH_4Cl (5 mL/g monobromodiene precursor) was added, the reaction mixture was filtered through a pad of Celite, then the filtrate was diluted with CH_2Cl_2 (50 mL/g monobromodiene precursor) and water (100 mL/g monobromodiene precursor). The aqueous and organic layers were separated and the aqueous layer was extracted with CH_2Cl_2 (10 mL/g monobromodiene precursor × 3). The combined organic layers were washed with saturated brine (20 mL/g monobromodiene precursor), dried over Na_2SO_4 and concentrated under reduced pressure.

(*E*)-(5-Methyl-4-vinylhexa-3,5-dien-1-yn-1-yl)benzene (4a-*E*)

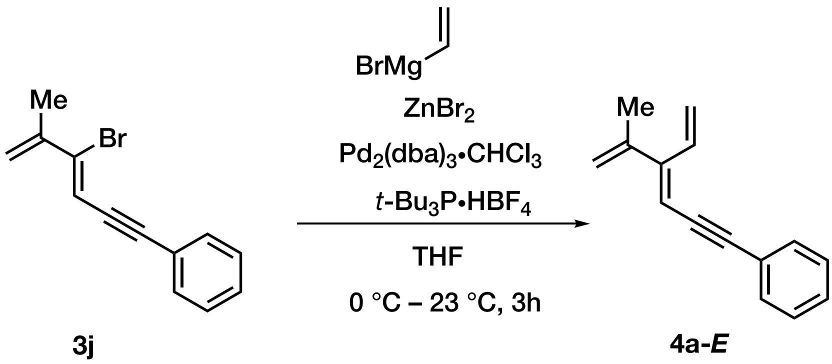

Following **Standard Procedure C**, the reaction mixture containing monobromodiene **3j** (75 mg, 0.303 mmol), $Pd_2(dba)_3$•$CHCl_3$ (11 mg, 0.011 mmol), *t*-Bu_3P•HBF_4 (6 mg, 0.021 mmol) $ZnBr_2$ (1.0 mL, 1.23 M in THF, 1.21 mmol) and vinyl magnesium bromide (1.3 mL, 0.68 M in THF, 0.910 mmol) in THF (4 mL) was stirred at 23 °C for 3h. After work up, purification by flash column chromatography (3 g SiO_2, 100% PS 40–60) gave dendralene **4a-*E*** as a colourless liquid (38 mg, 0.196 mmol, 65%, >95% retention). R_f = 0.49 (100% PS 40–60); ^{1}H NMR (400 MHz, $CDCl_3$) δ 7.49 – 7.42 (m, 2H), 7.38 – 7.27 (m, 3H), 6.91 (dd, J = 17.6, 10.8 Hz, 1H), 5.75 (s, 1H), 5.56 (d, J = 17.6 Hz, 1H), 5.45 (d, J = 10.8 Hz, 1H), 5.13 (s, 1H), 5.10 (s, 1H), 1.96 (s, 3H) ppm; ^{13}C NMR (100 MHz, $CDCl_3$) δ 152.0 (Cq), 142.9 (Cq), 133.5 (CH), 131.6 (2×CH), 128.5 (2×CH), 128.3 (CH), 123.7 (Cq), 120.0 (CH_2), 116.3 (CH_2), 107.4 (CH), 96.5 (Cq), 87.6 (Cq), 22.4 (CH_3) ppm; IR (thin film): ν_{max} = 3052, 2924, 2853, 1597, 1488 cm^{-1}; LRMS: (EI+): m/z (%): 194 ($[M]^{+\bullet}$, 48), 179 ($[M–CH_3]^{+\bullet}$, 49), 178 ($[M–CH_4]^{+\bullet}$, 100); HRMS (EI+): calculated for $C_{15}H_{14}$: 194.1096; found: 194.1091.

(*Z*)-(5-Methyl-4-vinylhexa-3,5-dien-1-yn-1-yl)benzene (4a-*Z*)

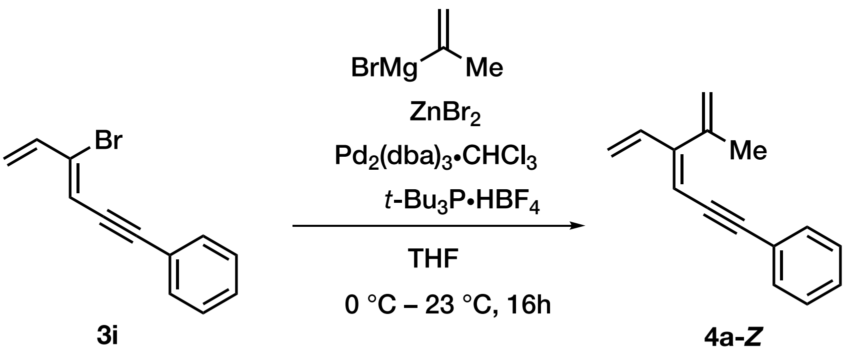

Following **Standard Procedure C**, the reaction mixture containing monobromodiene **3i** (75 mg, 0.322 mmol), $Pd_2(dba)_3$•$CHCl_3$ (13 mg, 0.013 mmol), *t*-Bu_3P•HBF_4 (8 mg, 0.026 mmol) $ZnBr_2$ (1.1 mL, 1.23 M in THF, 1.29 mmol) and isopropenyl magnesium bromide (1.9 mL, 0.51 M in THF, 0.965 mmol) in THF (4 mL) was stirred at 23 °C for 16h. After work up, purification by flash

column chromatography (3 g SiO_2, 100% PS 40–60) gave dendralene **4a-*Z*** as a colourless liquid (45 mg, 0.232 mmol, 72%, >95% retention). R_f = 0.44 (100% PS 40–60); ^{1}H NMR (400 MHz, $CDCl_3$) δ 7.45 – 7.36 (m, 2H), 7.33 – 7.27 (m, 3H), 6.41 (dd, J = 17.4, 10.7 Hz, 1H), 5.69 (s, 1H), 5.38 (d, J = 17.3 Hz, 1H), 5.28 (s, 1H), 5.21 (d, J = 10.6 Hz, 1H), 4.94 (s, 1H), 2.01 (s, 3H) ppm; ^{13}C NMR (100 MHz, $CDCl_3$) δ 154.3 (Cq), 141.2 (Cq), 137.2 (CH), 131.5 (2×CH), 128.4 (2×CH), 128.2 (CH), 123.9 (Cq), 117.8 (CH_2), 116.2 (CH_2), 108.7 (CH), 96.2 (Cq), 88.2 (Cq), 22.6 (CH_3) ppm; IR (thin film): ν_{max} = 3080, 2969, 2918, 2192, 1722, 1682, 1599 cm^{-1}; LRMS: (EI+): m/z (%): 194 ([M]$^{+\bullet}$, 44), 193 ([M–H]$^{+\bullet}$, 24), 179 ([M–CH_3]$^{+\bullet}$, 47), 178 ([M–CH_4]$^{+\bullet}$, 100); HRMS (EI+): calculated for $C_{15}H_{14}$: 194.1096; found: 194.1097.

(*E*)-(6-Methyl-4-vinylhepta-3,5-dien-1-yn-1-yl)benzene (4b-*E*)

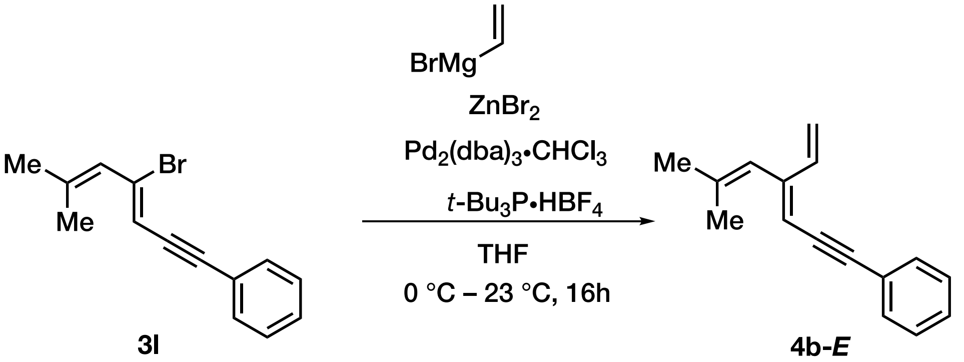

Following **Standard Procedure C**, the reaction mixture containing monobromodiene **3l** (65 mg, 0.250 mmol), $Pd_2(dba)_3$•$CHCl_3$ (10 mg, 0.010 mmol), *t*-Bu_3P•HBF_4 (6 mg, 0.020 mmol) $ZnBr_2$ (0.82 mL, 1.22 M in THF, 0.996 mmol) and vinyl magnesium bromide (1.1 mL, 0.70 M in THF, 0.747 mmol) in THF (3.5 mL) was stirred at 23 °C for 16h. After work up, purification by flash column chromatography (3 g SiO_2, 100% PS 40–60) gave dendralene **4b-*E*** as a colourless liquid (36 mg, 0.173 mmol, 69%, >95% retention). R_f = 0.33 (100% PS 40–60); ^{1}H NMR (400 MHz, $CDCl_3$) δ 7.49 – 7.44 (m, 2H), 7.36 – 7.28 (m, 3H), 7.12 (dd, J = 17.6, 10.8 Hz, 1H), 5.86 (d, J = 1.2 Hz, 1H), 5.62 (s, 1H), 5.41 (dd, J = 17.6, 1.6 Hz, 1H), 5.28 (dt, J = 10.8, 1.6 Hz, 1H), 1.87 (d, J = 1.2 Hz, 3H), 1.78 (d, J = 1.2 Hz, 3H) ppm; ^{13}C NMR (100 MHz, $CDCl_3$) δ 146.9 (Cq), 138.4 (Cq), 135.3 (CH), 131.5 (2×CH), 128.5 (2×CH), 128.2 (CH), 123.8 (Cq), 122.0 (CH), 117.8 (CH_2), 110.0 (CH), 96.8 (Cq), 87.4 (Cq), 26.5 (CH_3), 20.0 (CH_3) ppm; IR (thin film): ν_{max} = 2969, 2910, 2190, 1597 cm^{-1}; LRMS: (EI+): m/z (%): 208 ([M]$^{+\bullet}$, 33), 193 ([M–CH_3]$^{+\bullet}$, 50), 178 ([M–2×CH_3]$^{+\bullet}$, 100), 165 ([M–C_3H_7]$^{+\bullet}$, 57); HRMS (EI+): calculated for $C_{16}H_{16}$: 208.1252; found: 208.1251.

(*Z*)-(6-Methyl-4-vinylhepta-3,5-dien-1-yn-1-yl)benzene (4b-*Z*)

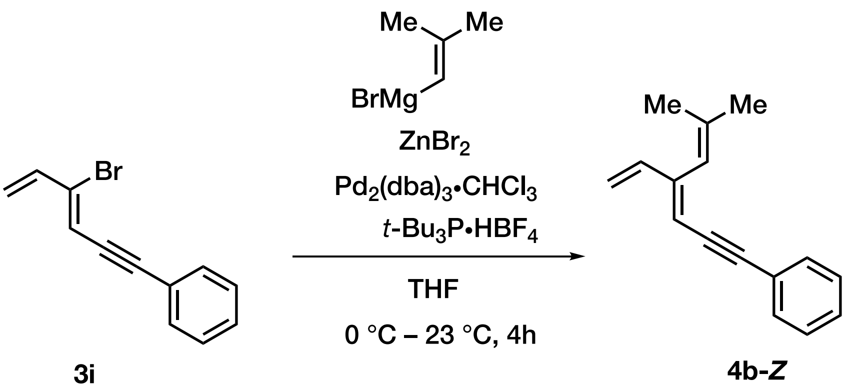

3i **4b-*Z***

Following **Standard Procedure C**, the reaction mixture containing monobromodiene **3i** (60 mg, 0.26 mmol), $Pd_2(dba)_3$•$CHCl_3$ (11 mg, 0.010 mmol), *t*-Bu_3P•HBF_4 (6 mg, 0.021 mmol) $ZnBr_2$ (0.84 mL, 1.23 M in THF, 1.03 mmol) and isobutenyl magnesium bromide (1.8 mL, 0.44 M in THF, 0.772 mmol) in THF (3 mL) was stirred at 23 °C for 4h. After work up, purification by flash column chromatography (3 g SiO_2, 100% PS 40–60) gave dendralene **4b-*Z*** as a pale yellow liquid (35 mg, 0.168 mmol, 65%, >90% retention). R_f = 0.30 (100% PS 40–60); ^{1}H NMR (400 MHz, $CDCl_3$) δ 7.42 – 7.37 (m, 2H), 7.35 – 7.27 (m, 3H), 6.45 (dd, J = 17.2, 10.4 Hz, 1H), 5.85 (s, 1H), 5.75 (s, 1H), 5.33 (d, J = 17.2 Hz, 1H), 5.18 (d, J = 10.4 Hz, 1H), 1.93 (s, 3H), 1.72 (s, 3H) ppm; ^{13}C NMR (100 MHz, $CDCl_3$) δ 148.7 (Cq), 139.4 (Cq), 138.5 (CH), 131.5 (2×CH), 128.5 (2×CH), 128.1 (CH), 124.0 (Cq), 119.7 (CH), 117.2 (CH_2), 110.5 (CH), 96.5 (Cq), 89.2 (Cq), 26.1 (CH_3), 20.8 (CH_3) ppm; ν_{max} = 3005, 2969, 2909, 2190, 1822, 1661, 1597 cm^{-1}; LRMS: (EI+): m/z (%): 208 ($[M]^{+\bullet}$, 39), 193 ($[M–CH_3]^{+\bullet}$, 49), 178 ($[M–2×CH_3]^{+\bullet}$, 100); HRMS (EI+): calculated for $C_{16}H_{16}$: 208.1252; found: 208.1249.

(*E*)-(6-Methyl-4-(prop-1-en-2-yl)hepta-3,5-dien-1-yn-1-yl)benzene (4c-*E*)

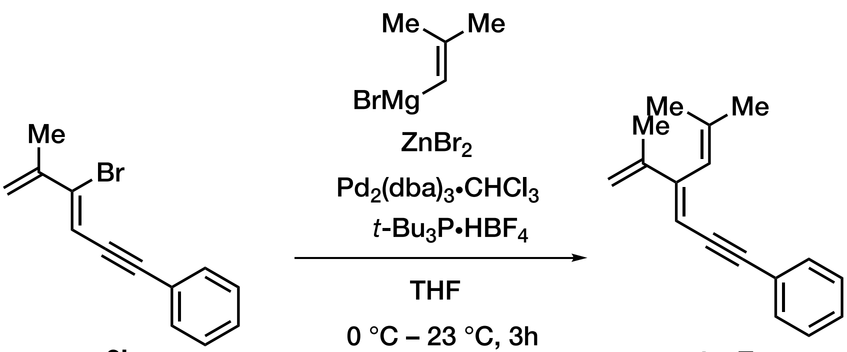

3j **4c-*E***

Following **Standard Procedure C**, the reaction mixture containing monobromodiene **3j** (52 mg, 0.21 mmol), $Pd_2(dba)_3$•$CHCl_3$ (9 mg, 0.0084 mmol), *t*-Bu_3P•HBF_4 (5 mg, 0.017 mmol), $ZnBr_2$ (0.68 mL, 1.23 M in THF, 0.842 mmol) and isobutenyl magnesium bromide (1.4 mL, 0.44 M in THF,

0.631 mmol) in THF (3 mL) was stirred at 23 °C for 3h. After work up, purification by flash column chromatography (3 g SiO_2, 100% PS 40–60) gave dendralene **4c-*E*** as a colourless liquid (27 mg, 0.121 mmol, 58%, >95% retention). R_f = 0.33 (100% PS 40–60); ^{1}H NMR (400 MHz, $CDCl_3$) δ 7.43 – 7.35 (m, 2H), 7.34 – 7.27 (m, 3H), 5.88 (s, 1H), 5.85 (s, 1H), 5.19 (s, 1H), 5.07 (s, 1H), 1.96 (s, 3H), 1.91 (d, J = 1.6 Hz, 3H), 1.68 (d, J = 1.2 Hz, 3H) ppm;
^{13}C NMR (100 MHz, $CDCl_3$) δ 150.6 (Cq), 142.8 (Cq), 138.1 (Cq), 131.4 (2×CH), 128.4 (2×CH), 128.0 (CH), 124.2 (Cq), 121.8 (CH), 117.0 (CH_2), 107.0 (CH), 95.7 (Cq), 89.5 (Cq), 25.9 (CH_3), 20.4 (CH_3), 20.4 (CH_3) ppm; IR (thin film): ν_{max} = 3076, 2970, 2928, 1598 cm^{-1}; LRMS: (EI+): m/z (%): 222 ($[M]^{+\bullet}$, 65), 207 ($[M–CH_3]^{+\bullet}$, 82), 192 ($[M–2×CH_3]^{+\bullet}$, 100); HRMS (EI+): calculated for $C_{17}H_{18}$: 222.1409; found: 222.1406.

(*Z*)-(6-Methyl-4-(prop-1-en-2-yl)hepta-3,5-dien-1-yn-1-yl)benzene (4c-*Z*)

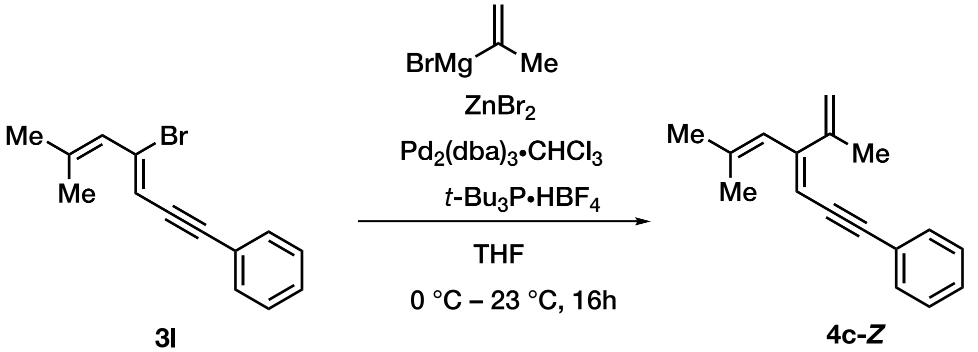

Following **Standard Procedure C**, the reaction mixture containing monobromodiene **3l** (70 mg, 0.268 mmol), $Pd_2(dba)_3$•$CHCl_3$ (11 mg, 0.0107 mmol), *t*-Bu_3P•HBF_4 (6 mg, 0.021 mmol) $ZnBr_2$ (0.88 mL, 1.22 M in THF, 1.07 mmol) and isopropenyl magnesium bromide (1.6 mL, 0.49 M in THF, 0.804 mmol) in THF (3.5 mL) was stirred at 23 °C for 16h. After work up, purification by flash column chromatography (3 g SiO_2, 100% PS 40–60) gave dendralene **4c-*Z*** as a colourless liquid (42 mg, 0.189 mmol, 70%, >90% retention). R_f = 0.29 (100% PS 40–60);
^{1}H NMR (400 MHz, $CDCl_3$) δ 7.48 – 7.39 (m, 2H), 7.38 – 7.24 (m, 3H), 5.83 (s, 1H), 5.61 (s, 1H), 5.20 (s, 1H), 5.18 (s, 1H), 2.11 (s, 3H), 1.86 (s, 3H), 1.84 (s, 3H) ppm; ^{13}C NMR (100 MHz, $CDCl_3$) δ 151.6 (Cq), 143.9 (Cq), 137.6 (Cq), 131.3 (2×CH), 128.4 (2×CH), 128.0 (CH), 125.9 (CH), 124.2 (Cq), 116.6 (CH_2), 107.5 (CH), 95.1 (Cq), 89.1 (Cq), 27.1 (CH_3), 22.7 (CH_3), 19.7 (CH_3) ppm; IR (thin film): ν_{max} = 3079, 2969, 2911, 2190, 1684, 1598 cm^{-1}; LRMS: (EI+): m/z (%): 222 ($[M]^{+\bullet}$, 63), 207 ($[M–CH_3]^{+\bullet}$, 85), 192 ($[M–2×CH_3]^{+\bullet}$, 100); HRMS (EI+): calculated for $C_{17}H_{18}$: 222.1409; found: 222.1410.

((1*E*,3*E*)-3-Vinylhexa-1,3-dien-5-yne-1,6-diyl)dibenzene (4d-*E*)

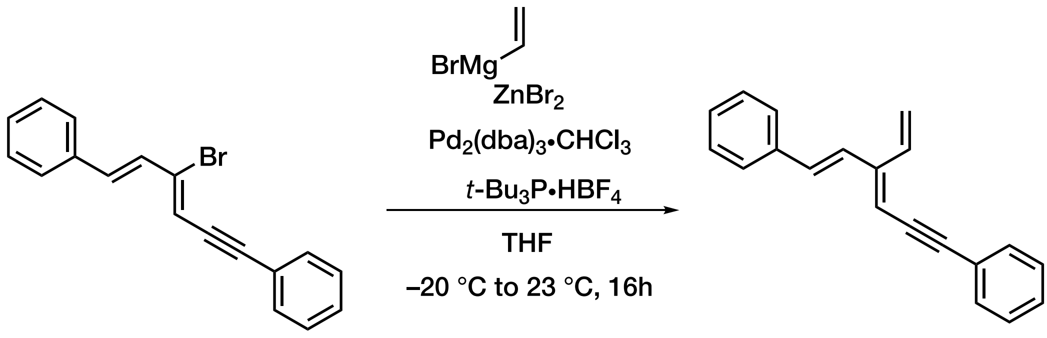

3K **4d-*E***

Following **Standard Procedure C**, vinyl magnesium bromide (0.97 mL, 0.70 M in THF, 0.68 mmol) was added to a THF (4 mL) solution of monobromodiene **3k** (70 mg, 0.23 mmol), $Pd_2(dba)_3 \cdot CHCl_3$ (9 mg, 0.009 mmol), *t*-$Bu_3P \cdot HBF_4$ (5 mg, 0.018 mmol) and $ZnBr_2$ (0.74 mL, 1.22 M in THF, 0.906 mmol) at −20 °C and brought the reaction mixture to 23 °C and stirred for 16h. After work up, purification by flash column chromatography (3 g SiO_2, 0.5% EtOAc in PS 40–60) gave dendralene **4d-*E*** as a pale yellow solid (35 mg, 0.137 mmol, 60%, >95% selective retention). R_f= 0.50 (5% EtOAc in PS 40–60); m.p. 64 – 68 °C (CH_2Cl_2); ^{1}H NMR (400 MHz, $CDCl_3$) δ 7.51 – 7.43 (m, 4H), 7.39 – 7.30 (m, 5H), 7.30 – 7.24 (m, 1H), 6.99 (dd, J = 17.7, 11.1 Hz, 1H), 6.92 (s, 2H), 5.99 (s, 1H), 5.75 (dd, J = 17.7, 0.8 Hz, 1H), 5.51 (d, J = 11.2 Hz, 1H) ppm;
^{13}C NMR (100 MHz, $CDCl_3$) δ 146.6 (Cq), 137.2 (Cq), 133.4 (CH), 131.8 (CH), 131.6 (2×CH), 128.9 (2×CH), 128.5 (2×CH), 128.4 (CH), 128.2 (CH), 127.0 (CH), 126.9 (2×CH), 123.7 (Cq), 119.2 (CH_2), 108.6 (CH), 98.2 (Cq), 88.3 (Cq) ppm; IR (thin film): ν_{max} = 3052, 3024, 2923, 2186, 1595 cm^{-1}; LRMS: (EI+): m/z (%): 256 ([M]$^{+\bullet}$, 100), 255 ([M–H]$^{+\bullet}$, 69), 242 ([M–CH_2]$^{+\bullet}$, 17), 229 ([M–CH_2CH]$^{+\bullet}$, 14), 179 ([M–C_6H_5]$^{+\bullet}$, 17); HRMS (EI+): calculated for $C_{20}H_{16}$: 256.1252; found: 256.1251.

((1*E*,3*Z*)-3-Vinylhexa-1,3-dien-5-yne-1,6-diyl)dibenzene (4d-*Z*)

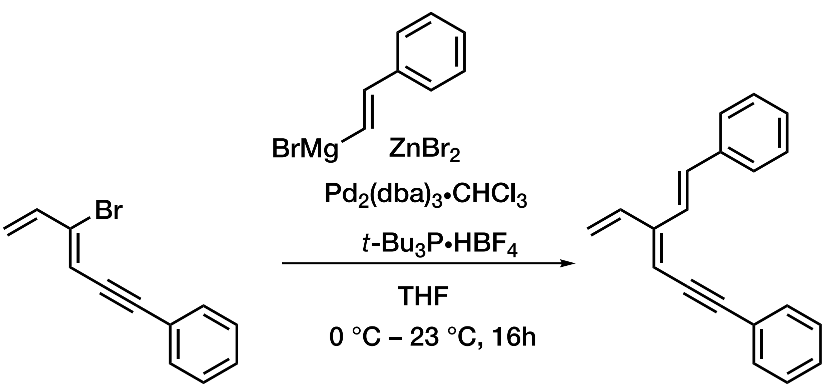

3i **4d-*Z***

Following **Standard Procedure C**, the reaction mixture containing monobromodiene **3i** (63 mg, 0.27 mmol), $Pd_2(dba)_3$•$CHCl_3$ (11 mg, 0.011 mmol), *t*-Bu_3P•HBF_4 (6 mg, 0.022 mmol) $ZnBr_2$ (0.88 mL, 1.23 M in THF, 1.08 mmol) and styrenyl magnesium bromide (1.4 mL, 0.58 M in THF, 0.811 mmol) in THF (4.0 mL) was stirred at 23 °C for 16h. After work up, purification by purification by reverse phase HPLC (Phenomenex Luna 5u C18(2), 100 A, 250×10 mm, eluting with 15% water/MeCN, t_R = 9.6) gave dendralene **4d-*Z*** as a colourless liquid (35 mg, 0.14 mmol, 51%, >85% selective retention). R_f = 0.50 (5% EtOAc in PS 40–60);

^{1}H NMR (400 MHz, $CDCl_3$) δ 7.57 – 7.49 (m, 4H), 7.45 (d, *J* = 16.4 Hz, 1H), 7.42 – 7.33 (m, 5H), 7.33 – 7.26 (m, 1H), 7.02 (d, *J* = 16.4 Hz, 1H), 6.66 (dd, *J* = 17.2, 10.8 Hz, 1H), 5.95 (s, 1H), 5.64 (dd, *J* = 17.2, 0.8 Hz, 1H), 5.33 (d, *J* = 10.8 Hz, 1H) ppm; ^{13}C NMR (100 MHz, $CDCl_3$) δ 146.7 (Cq), 137.3 (Cq), 135.3 (CH), 133.0 (CH), 131.5 (2×CH), 128.9 (2×CH), 128.6 (2×CH), 128.4 (CH), 128.3 (CH), 127.0 (2×CH), 125.4 (CH), 123.7 (Cq), 117.5 (CH_2), 108.4 (CH), 98.3 (Cq), 88.2 (Cq) ppm; IR (thin film): ν_{max} = 3079, 3058, 3027, 2923, 2852, 2185, 1597 cm^{-1}; LRMS: (EI+): m/z (%): 256 ($[M]^{+•}$, 77), 255 ($[M–H]^{+•}$, 84), 242 ($[M–CH_2]^{+•}$, 19), 241 ($[M–CH_3]^{+•}$, 100), 229 ($[M–CH_3CH_2]^{+•}$, 17), 179 ($[M–C_6H_5]^{+•}$, 42); HRMS (EI+): calculated for $C_{20}H_{16}$: 256.1252; found: 256.1254.

((1*E*,3*E*)-3-(Prop-1-en-2-yl)hexa-1,3-dien-5-yne-1,6-diyl)dibenzene (4e-*E*)

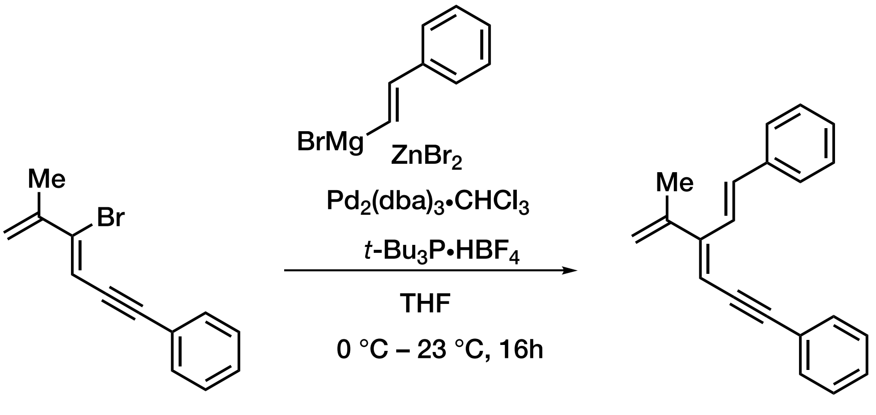

3j **4e-*E***

Following **Standard Procedure C**, the reaction mixture containing monobromodiene **3j** (71 mg, 0.287 mmol), $Pd_2(dba)_3$•$CHCl_3$ (12 mg, 0.0115 mmol), *t*-Bu_3P•HBF_4 (7 mg, 0.023 mmol) $ZnBr_2$ (0.94 mL, 1.22 M in THF, 1.15 mmol) and styrenyl magnesium bromide (1.5 mL, 0.58 M in THF, 0.862 mmol) in THF (4 mL) was stirred at 23 °C for 16h. After work up, purification by reverse phase HPLC (Phenomenex Luna 5u C18(2), 100A, 250×10 mm, eluting with 15% water/MeCN, t_R =19 min) gave dendralene **4e-*E*** as a colourless liquid (35 mg, 0.13 mmol, 45%, >95% retention). R_f = 0.34 (5% EtOAc in PS 40–60);

^{1}H NMR (400 MHz, $CDCl_3$) δ 7.39 – 7.28 (m, 4H), 7.24 – 7.05 (m, 7H), 6.71 (d, J = 16.4 Hz, 1H), 5.61 (s, 1H), 5.02 (s, 1H), 4.96 (s, 1H), 1.85 (s, 3H) ppm; ^{13}C NMR (100 MHz, $CDCl_3$) δ 152.3 (Cq), 143.6 (Cq), 137.4 (Cq), 134.3 (CH), 131.5 (2×CH), 128.9 (2×CH), 128.5 (2×CH), 128.3 (CH), 128.2 (CH), 127.0 (2×CH), 125.6 (CH), 123.8 (Cq), 116.3 (CH_2), 107.5 (CH), 97.3 (Cq), 87.9 (Cq), 23.0 (CH_3) ppm; IR (thin film): ν_{max} = 3078, 3026, 2187, 2919, 1596 cm^{-1}; LRMS: (EI+): m/z (%): 270 ([M]$^{+\bullet}$, 89), 269 ([M–H]$^{+\bullet}$, 54), 255 ([M–CH_3]$^{+\bullet}$, 100); HRMS (EI+): calculated for $C_{21}H_{18}$: 270.1409; found: 270.1411.

((1*E*,3*Z*)-3-(Prop-1-en-2-yl)hexa-1,3-dien-5-yne-1,6-diyl)dibenzene (4e-*Z*)

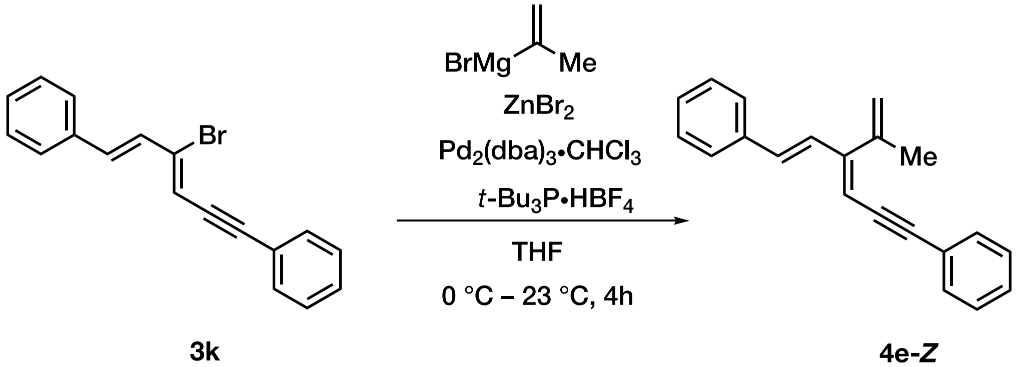

Following **Standard Procedure C**, the reaction mixture containing monobromodiene **3k** (70 mg, 0.226 mmol), $Pd_2(dba)_3$•$CHCl_3$ (9 mg, 0.0091 mmol), *t*-Bu_3P•HBF_4 (5 mg, 0.018 mmol) $ZnBr_2$ (0.74 mL, 1.22 M in THF, 0.906 mmol) and isopropenyl magnesium bromide (1.3 mL, 0.51 M in THF, 0.679 mmol) in THF (3.5 mL) was stirred at 23 °C for 4h. After work up, purification by flash column chromatography (3 g SiO_2, 0.5% EtOAc in PS 40–60) gave dendralene **4e-*Z*** as a pale yellow solid (43 mg, 0.16 mmol, 70%, >95% retention). R_f = 0.61 (5% EtOAc in PS 40–60); m.p. 77 – 79 °C (hexane:EtOAc, 8:2); ^{1}H NMR (400 MHz, $CDCl_3$) δ 7.62 – 7.42 (m, 4H), 7.42 – 7.21 (m, 6H), 6.88 (d, J = 16.0 Hz, 1H), 6.73 (d, J = 16.0 Hz, 1H), 5.83 (s, 1H), 5.39 (s, 1H), 5.03 (s, 1H), 2.12 (s, 3H) ppm; ^{13}C NMR (100 MHz, $CDCl_3$) δ 154.5 (Cq), 141.7 (Cq), 137.1 (Cq), 132.5 (CH), 131.5 (2×CH), 129.3 (CH), 128.8 (2×CH), 128.4 (2×CH), 128.2 (CH), 128.1 (CH), 126.9 (2×CH), 124.0 (Cq), 116.3 (CH_2), 108.8 (CH), 96.9 (Cq), 88.8 (Cq), 22.8 (CH_3) ppm; IR (thin film): ν_{max} = 3057, 3022, 2967, 1640, 1594 cm^{-1}; LRMS: (EI+): m/z (%): 270 ([M]$^{+\bullet}$, 91), 269 ([M–H]$^{+\bullet}$, 50), 255 ([M–CH_3]$^{+\bullet}$, 100); HRMS (EI+): calculated for $C_{21}H_{18}$: 270.1409; found: 270.1410.

((1*E*,3*E*)-5-Methyl-4-vinylhexa-1,3,5-trien-1-yl)benzene (4f-*E*)

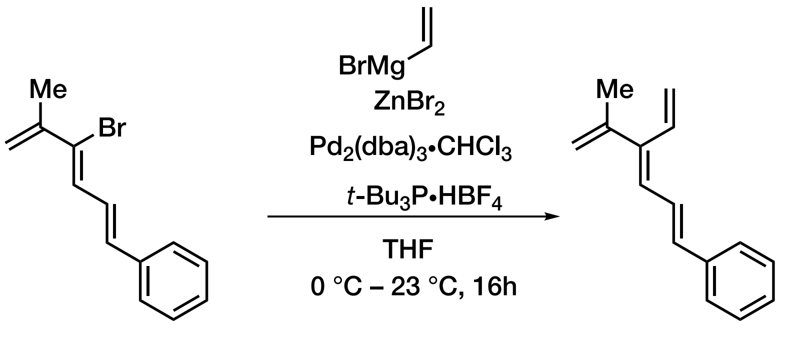

3f **4f-*E***

Following **Standard Procedure C**, the reaction mixture containing monobromodiene **3f** (105 mg, 0.421 mmol), $Pd_2(dba)_3$•$CHCl_3$ (15 mg, 0.015 mmol), *t*-Bu_3P•HBF_4 (9 mg, 0.030 mmol) $ZnBr_2$ (1.70 mL, 1.48 M in THF, 2.53 mmol) and vinyl magnesium bromide (2.50 mL, 0.85 M in THF, 2.11 mmol) in THF (6 mL) was stirred at 23 °C for 16h. After work up, purification by flash column chromatography (5 g SiO_2, 100% PS 40–60) gave dendralene **4f-*E*** as a colourless liquid (65 mg, 0.331 mmol, 79%, >95% retention). R_f = 0.38 (100% PS 40–60);

^{1}H NMR (400 MHz, $CDCl_3$) δ 7.42 (d, *J* = 7.6 Hz, 2H), 7.32 (t, *J* = 7.6 Hz, 2H), 7.28 – 7.18 (m, 2H), 6.69 – 6.56 (m, 2H), 6.35 (d, *J* = 11.2 Hz, 1H), 5.45 (dd, *J* = 10.8, 1.6 Hz, 1H), 5.30 (dd, *J* = 17.6, 1.6 Hz, 1H), 5.09 (s, 1H), 5.07 (s, 1H), 1.99 (s, 3H) ppm; ^{13}C NMR (100 MHz, $CDCl_3$) δ 143.8 (Cq), 141.9 (Cq), 137.8 (Cq), 133.7 (CH), 133.2 (CH), 128.8 (2×CH), 127.9 (CH), 127.7 (CH), 126.6 (2×CH), 126.3 (CH), 119.8 (CH_2), 115.4 (CH_2), 21.6 (CH_3) ppm; ν_{max} = 3079, 3033, 2997, 1796, 1601 cm^{-1}; LRMS: (EI+): m/z (%): 196 ($[M]^{+\bullet}$, 68), 181 ($[M–CH_3]^{+\bullet}$, 100), 167 ($[M–CH_3CH_2]^{+\bullet}$, 33), 155 ($[M–C_3H_5]^{+\bullet}$, 21); HRMS (EI+): calculated for $C_{15}H_{16}$: 196.1252; found: 196.1251.

((1*E*,3*Z*)-5-Methyl-4-vinylhexa-1,3,5-trien-1-yl)benzene (4f-*Z*)

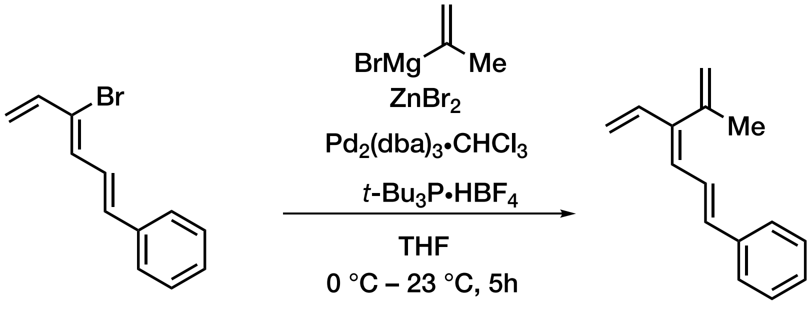

3e **4f-*Z***

Following **Standard Procedure C**, the reaction mixture containing monobromodiene **3e** (106 mg, 0.451 mmol), $Pd_2(dba)_3{\cdot}CHCl_3$ (14 mg, 0.0135 mmol), *t*-$Bu_3P{\cdot}HBF_4$ (8 mg, 0.03 mmol) $ZnBr_2$ (1.5 mL, 1.48 M in THF, 2.25 mmol) and isopropenyl magnesium bromide (3.1 mL, 0.59 M in THF, 1.80 mmol) in THF (6 mL) was stirred at 23 °C for 5h. After work up, purification by flash column chromatography (5 g SiO_2, 100% PS 40–60) gave dendralene **4f-*Z*** as a colourless liquid (65 mg, 0.331 mmol, 73%, >86% retention). R_f = 0.38 (100% PS 40–60);

^{1}H NMR (400 MHz, $CDCl_3$) δ 7.40 (d, J = 7.6 Hz, 2H), 7.31 (t, J = 7.6 Hz, 2H), 7.21 (t, J = 7.3 Hz, 1H), 7.02 (dd, J = 15.6, 11.1 Hz, 1H), 6.60 (d, J = 15.6 Hz, 1H), 6.39 (dd, J = 17.3, 10.5 Hz, 1H), 6.18 (d, J = 11.1 Hz, 1H), 5.29 (s, 1H), 5.25 (d, J = 17.3 Hz, 1H), 5.12 (d, J = 10.5 Hz, 1H), 4.84 (s, 1H), 1.93 (s, 3H) ppm; ^{13}C NMR (100 MHz, $CDCl_3$) δ 145.4 (Cq), 141.6 (Cq), 138.3 (CH), 137.7 (Cq), 133.3 (CH), 129.9 (CH), 128.7 (2×CH), 127.7 (CH), 126.5 (2×CH), 126.5 (CH), 116.4 (CH_2), 115.4 (CH_2), 23.6 (CH_3) ppm; ν_{max} = 3081, 3029, 3000, 2969, 1809, 1643, 1599 cm^{-1}; LRMS: (EI+): m/z (%): 196 ($[M]^{+\bullet}$, 90), 181 ($[M–CH_3]^{+\bullet}$, 100), 166 ($[M–CH_3CH_2H]^{+\bullet}$, 55), 165 ($[M–C_2H_7]^{+\bullet}$, 68) 155 ($[M–C_3H_5]^{+\bullet}$, 21); HRMS (EI+): calculated for $C_{15}H_{16}$: 196.1252; found: 196.1254.

1-Methoxy-4-((*E*)-3-methyl-2-((*E*)-styryl)buta-1,3-dien-1-yl)benzene (4g-*E*)

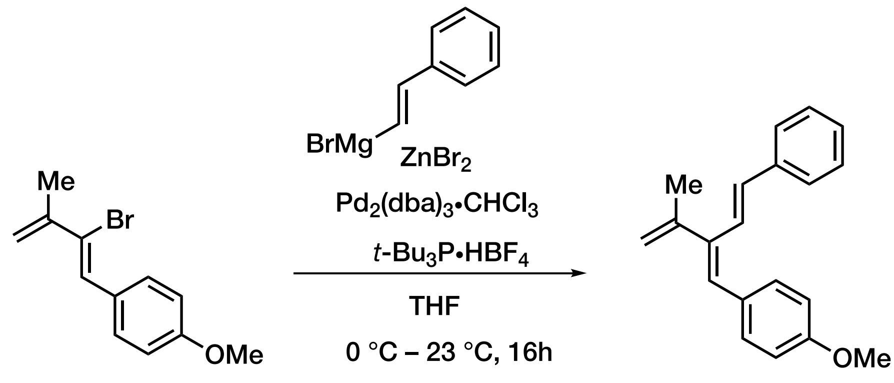

3b **4g-*E***

Following **Standard Procedure C**, the reaction mixture containing monobromodiene **3b** (90 mg, 0.36 mmol), $Pd_2(dba)_3{\cdot}CHCl_3$ (15 mg, 0.014 mmol), *t*-$Bu_3P{\cdot}HBF_4$ (8 mg, 0.03 mmol) $ZnBr_2$ (1.2 mL, 1.22 M in THF, 1.42 mmol) and styrenyl magnesium bromide (1.9 mL, 0.56 M in THF, 1.07 mmol) in THF (5 mL) was stirred at 23 °C for 16h. After work up, purification by reverse phase HPLC (Phenomenex Luna 5u C18(2) 100A 250×10 mm, eluting with 15% water/MeCN, t_R = 14.9 min) gave dendralene **4g-*E*** as a colourless liquid (30 mg, 0.109 mmol, 30%, >90% retention). R_f = 0.49 (5% EtOAc in PS 40–60); ^{1}H NMR (400 MHz, $CDCl_3$) δ 7.42 (d, J = 7.2 Hz, 2H), 7.36 – 7.29 (m, 4H), 7.27 – 7.21 (m, 1H), 7.11 (d, J = 16.4 Hz, 1H), 6.90 (d, J = 8.4 Hz, 2H), 6.66 (d, J = 16.4 Hz, 1H), 6.54 (s, 1H), 5.14 (s, 2H), 3.84 (s, 3H), 2.04 (s, 3H) ppm;

^{13}C NMR (100 MHz, $CDCl_3$) δ 158.8 (Cq), 146.0 (Cq), 141.3 (Cq), 137.8 (Cq), 133.0 (CH), 131.2 (2×CH), 130.4 (Cq), 128.8 (2×CH), 128.1 (CH), 127.6 (CH), 126.6 (2×CH), 125.8 (CH), 115.4 (CH_2), 113.8 (2×CH), 55.4 (CH_3), 22.8 (CH_3) ppm; IR (thin film): ν_{max} = 3026, 2922, 2835, 1603, 1507 cm^{-1}; LRMS: (EI+): m/z (%): 276 ($[M]^{+\bullet}$, 79), 261 ($[M–CH_3]^{+\bullet}$, 43), 246 ($[M–2×CH_3]^{+\bullet}$, 13), 121 (100); HRMS (EI+): calculated for $C_{20}H_{20}O$: 276.1514; found: 276.1512.

1-Methoxy-4-((*Z*)-3-methyl-2-((*E*)-styryl)buta-1,3-dien-1-yl)benzene (4g-*Z*)

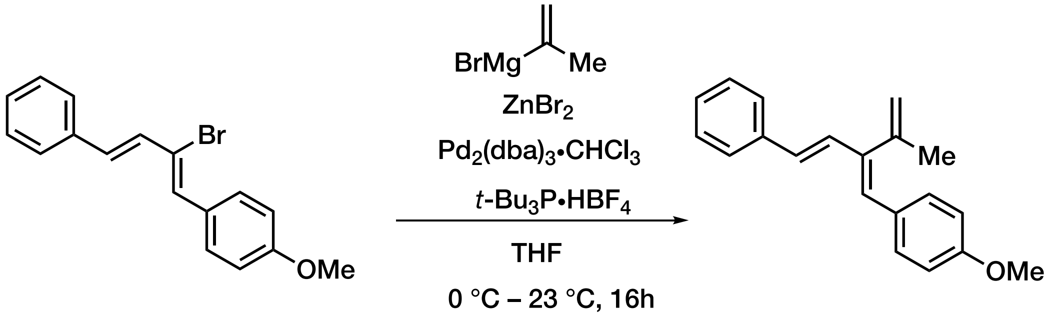

3c **4g-*Z***

Following **Standard Procedure C**, the reaction mixture containing monobromodiene **3c** (200 mg, 0.634 mmol), $Pd_2(dba)_3$•$CHCl_3$ (26 mg, 0.025 mmol), *t*-Bu_3P•HBF_4 (15 mg, 0.051 mmol) $ZnBr_2$ (2.1 mL, 1.23 M in THF, 2.54 mmol) and isopropenyl magnesium bromide (3.7 mL, 0.51 M in THF, 1.90 mmol) in THF (10 mL) was stirred at 23 °C for 16h. After work up, purification by flash column chromatography (8 g SiO_2, 1% EtOAc in PS 40–60) gave dendralene **4g-*Z*** as a colourless solid (160 mg, 0.579 mmol, 91%, >95% retention). R_f = 0.36 (5% EtOAc in PS 40–60); m.p. 79 – 82 °C (hexane:CH_2Cl_2, 9:1); ^1H NMR (400 MHz, $CDCl_3$) δ 7.52 (d, J = 8.5 Hz, 2H), 7.44 (d, J = 7.6 Hz, 2H), 7.32 (t, J = 7.6 Hz, 2H), 7.25 – 7.18 (m, 1H), 6.91 (d, J = 15.8 Hz, 1H), 6.86 (d, J = 8.5 Hz, 2H), 6.60 (d, J = 15.8 Hz, 1H), 6.40 (s, 1H), 5.38 (s, 1H), 5.00 (s, 1H), 3.82 (s, 3H), 1.97 (s, 3H) ppm; ^{13}C NMR (100 MHz, $CDCl_3$) δ 158.9 (Cq), 142.7 (Cq), 141.5 (Cq), 137.9 (Cq), 132.7 (CH), 130.5 (2×CH), 129.9 (Cq), 129.3 (CH), 128.8 (CH), 128.7 (2×CH), 127.3 (CH), 126.5 (2×CH), 116.8 (CH_2), 113.9 (2×CH), 55.4 (CH_3), 22.8 (CH_3) ppm; IR (thin film): ν_{max} = 3081, 3012, 2959, 2835, 1601, 1507 cm^{-1}; LRMS: (EI+): m/z (%): 276 ($[M]^{+\bullet}$, 75), 261 ($[M–CH_3]^{+\bullet}$, 45), 246 ($[M–2×CH_3]^{+\bullet}$, 15), 121 (100); HRMS (EI+): calculated for $C_{20}H_{20}O$: 276.1514; found: 276.1516.

1-Methoxy-4-((*Z*)-4-methyl-2-((*E*)-styryl)penta-1,3-dien-1-yl)benzene (4h-*Z*)

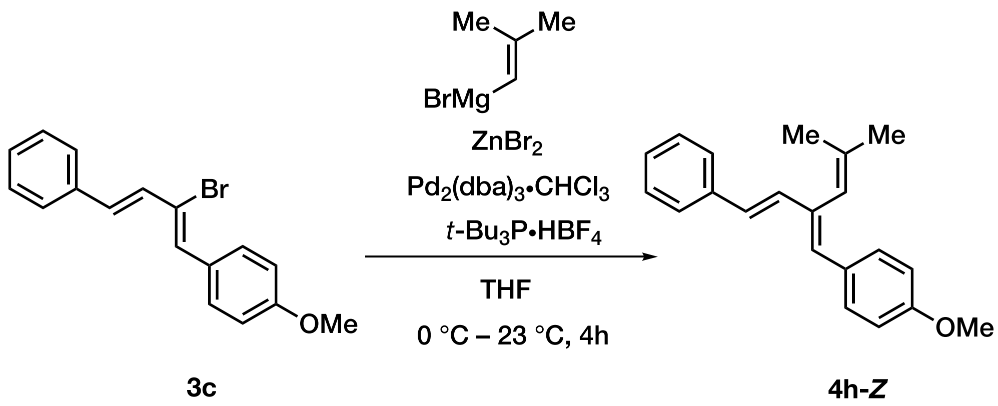

Following **Standard Procedure C**, the reaction mixture containing monobromodiene **3c** (60 mg, 0.19 mmol), $Pd_2(dba)_3$•$CHCl_3$ (6 mg, 0.0057 mmol), *t*-Bu_3P•HBF_4 (4 mg, 0.013 mmol) $ZnBr_2$ (0.62 mL, 1.23 M in THF, 0.761 mmol) and isobutenyl magnesium bromide (1.3 mL, 0.44 M in THF, 0.571 mmol) in THF (3 mL) was stirred at 23 °C for 4h. After work up, purification by flash column chromatography (3 g SiO_2, 2% EtOAc in PS 40–60) gave dendralene **4h-*Z*** as a white solid (52 mg, 0.179 mmol, 94%, >95% retention). R_f = 0.46 (5% EtOAc in PS 40–60); m.p. 74 – 78 °C (hexane); ^1H NMR (400 MHz, $(CD_3)_2SO$) δ 7.50 – 7.46 (m, 4H), 7.33 (t, J = 7.6 Hz, 2H), 7.24 – 7.20 (m, 1H), 7.05 (d, J = 16.0 Hz, 1H), 6.91 (d, J = 8.8 Hz, 2H), 6.64 (s, 1H), 6.46 (d, J = 16.0 Hz, 1H), 5.93 (s, 1H), 3.77 (s, 3H), 1.92 (d, J = 1.2 Hz, 3H), 1.44 (d, J = 0.8 Hz, 3H) ppm; ^{13}C NMR (100 MHz, $(CD_3)_2SO$) δ 158.3 (Cq), 137.4 (Cq), 136.5 (Cq), 135.2 (Cq), 133.1 (CH), 131.4 (CH), 130.2 (2×CH), 130.1 (Cq), 128.7 (2×CH), 128.1 (CH), 127.1 (CH), 126.2 (2×CH), 120.7 (CH), 113.8 (2×CH), 55.1 (CH_3), 25.1 (CH_3), 19.4 (CH_3) ppm; IR (thin film): ν_{max} = 3022, 2930, 1599, 1505 cm^{-1}; LRMS: (EI+): m/z (%): 290 ($[M]^{+\bullet}$, 100); HRMS (EI+): calculated for $C_{21}H_{22}O$: 290.1671; found: 290.1674.

1-Methoxy-4-((1*E*,3*E*)-4-phenyl-2-vinylbuta-1,3-dien-1-yl)benzene (4i-*E*)

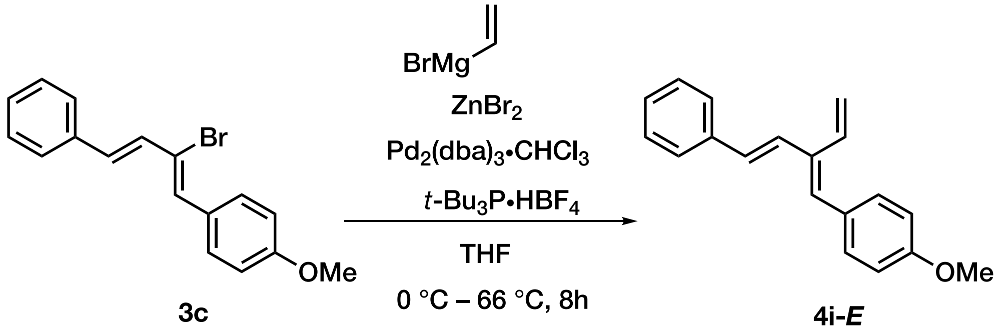

Following **Standard Procedure C**, the reaction mixture containing monobromodiene **3c** (50 mg, 0.16 mmol), $Pd_2(dba)_3$•$CHCl_3$ (5 mg, 0.005 mmol), *t*-Bu_3P•HBF_4 (3 mg, 0.0095 mmol) $ZnBr_2$ (0.77 mL, 1.23 M in THF, 0.952 mmol) and vinyl magnesium bromide (1.2 mL, 0.68 M in THF, 0.793

mmol) in THF (2.5 mL) was stirred at 66 °C for 8h. After work up, purification by flash column chromatography (3 g SiO2, 1.5% EtOAc in PS 40–60) gave dendralene **4i-*E*** as a colourless liquid (30 mg, 0.114 mmol, 72%, >95% retention). R_f = 0.43 (5% EtOAc in PS 40–60); ^{1}H NMR (400 MHz, $CDCl_3$) δ 7.46 (d, J = 7.5 Hz, 2H), 7.40 – 7.29 (m, 4H), 7.29 – 7.19 (m, 1H), 6.99 – 6.82 (m, 4H), 6.75 (dd, J = 17.9, 11.2 Hz, 1H), 6.70 (s, 1H), 5.51 (dd, J = 17.8, 1.5 Hz, 1H), 5.43 (d, J = 11.1 Hz, 1H), 3.83 (s, 3H) ppm; ^{13}C NMR (100 MHz, $CDCl_3$) δ 158.9 (Cq), 137.8 (Cq), 136.3 (Cq), 134.4 (CH), 131.2 (2×CH), 130.6 (CH), 130.2 (CH), 130.1 (Cq), 129.7 (CH), 128.8 (2×CH), 127.5 (CH), 126.6 (2×CH), 118.6 (CH_2), 113.8 (2×CH), 55.4 (CH_3) ppm; IR (thin film): ν_{max} = 3026, 2929, 2835, 1701, 1602, 1507 cm^{-1}; LRMS: (EI+): m/z (%): 262 ($[M]^{+\bullet}$, 100), 247 ($[M–CH_3]^{+\bullet}$, 13), 231 ($[M–OCH_3]^{+\bullet}$, 17); HRMS (EI+): calculated for $C_{19}H_{18}O$: 262.1358; found: 262.1360.

((1*E*,3*E*)-6-methyl-4-(prop-1-en-2-yl)hepta-1,3,5-trien-1-yl)benzene (4j-*E*)

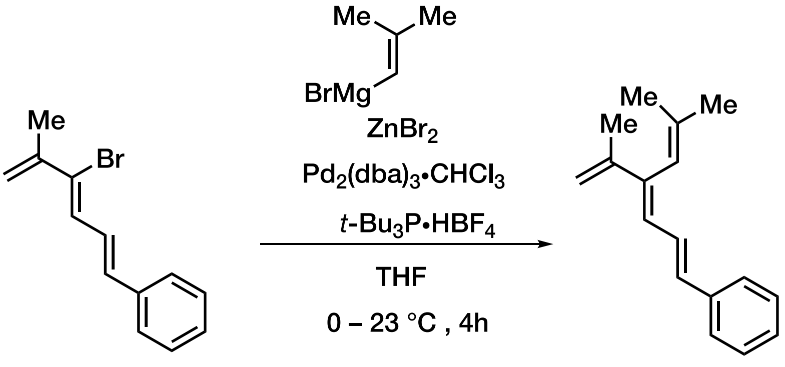

3f **4j-*E***

Following **Standard Procedure C**, the reaction mixture containing monobromodiene **3f** (92 mg, 0.37 mmol), $Pd_2(dba)_3$•$CHCl_3$ (12 mg, 0.011 mmol), *t*-Bu_3P•HBF_4 (78 mg, 0.026 mmol) $ZnBr_2$ (1.2 mL, 1.23 M in THF, 1.48 mmol) and isobutenyl magnesium bromide (2.5 mL, 0.44 M in THF, 1.11 mmol) in THF (5 mL) was stirred at 23 °C for 4h. After work up, purification by flash column chromatography (3 g SiO_2, 100% PS 40–60) gave dendralene **4j-*E*** as a colourless liquid (50 mg, 0.223 mmol, 60%, >90% retention). R_f = 0.44 (100% PS 40–60); ^{1}H NMR (400 MHz, $CDCl_3$) δ 7.44 – 7.36 (m, 2H), 7.36 – 7.27 (m, 2H), 7.25 – 7.17 (m, 1H), 6.92 (dd, J = 15.6, 10.8 Hz, 1H), 6.63 (d, J = 15.6 Hz, 1H), 6.39 (d, J = 10.8 Hz, 1H), 5.81 (s, 1H), 5.11 (d, J = 2.0 Hz, 1H), 5.00 (s, 1H), 2.00 (s, 3H), 1.93 (d, J = 1.4 Hz, 3H), 1.54 (d, J = 1.1 Hz, 3H) ppm; ^{13}C NMR (100 MHz, $CDCl_3$) δ 143.3 (Cq), 140.8 (Cq), 138.1 (Cq), 136.9 (Cq), 132.8 (CH), 128.7 (2×CH), 128.1 (CH), 127.5 (CH), 127.2 (CH), 126.4 (2×CH), 121.9 (CH), 115.3 (CH_2), 25.5 (CH_3), 20.6 (CH_3), 19.7 (CH_3) ppm; IR (thin film): ν_{max} = 3030, 2968, 2910, 2855, 1789, 1601 cm^{-1}; LRMS: (EI+): m/z (%):

224 ([M]$^{+\bullet}$, 100), 209 ([M–CH_3]$^{+\bullet}$, 60), 194 ([M–CH_3CH_3]$^{+\bullet}$, 11), 179 ([M–3×CH_3]$^{+\bullet}$, 37), 165 ([M–C_4H_{11}]$^{+\bullet}$, 54); HRMS (EI+): calculated for $C_{17}H_{20}$: 224.1565; found: 224.1565.

(*E*)-2-Methyl-3-vinyltetradeca-1,3-diene (4k-*E*)

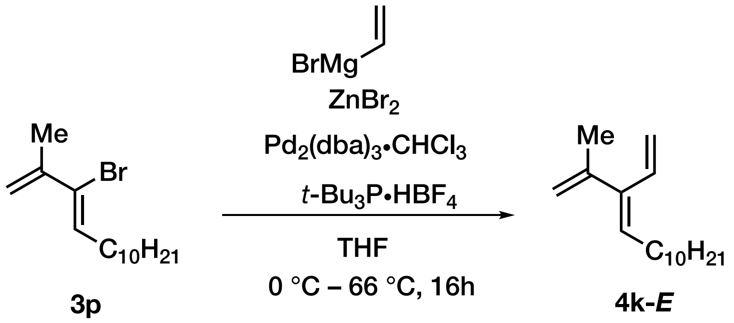

Following **Standard Procedure C**, the reaction mixture containing monobromodiene **3p** (70 mg, 0.244 mmol), $Pd_2(dba)_3$•$CHCl_3$ (10 mg, 0.010 mmol), *t*-Bu_3P•HBF_4 (6 mg, 0.019 mmol) $ZnBr_2$ (1.0 mL, 1.22 M in THF, 1.22 mmol) and vinyl magnesium bromide (1.4 mL, 0.70 M in THF, 0.975 mmol) in THF (4 mL) was stirred at 66 °C for 16h. After work up, purification by flash column chromatography (3 g SiO_2, 100% PS 40–60) gave dendralene **4k-*E*** as a colourless liquid (34 mg, 0.145 mmol, 60%, >90% retention). R_f = 0.67 (100% PS 40–60); ^{1}H NMR (400 MHz, $CDCl_3$) δ 6.46 (dd, J = 17.6, 11.2 Hz, 1H), 5.53 (t, J = 7.5 Hz, 1H), 5.25 (d, J = 11.2 Hz, 1H), 5.16 (dd, J = 17.6, 2.0 Hz, 1H), 4.94 (s, 1H), 4.91 (s, 1H), 2.18 (q, J = 7.4 Hz, 2H), 1.89 (s, 3H), 1.50 – 1.18 (m, 16H), 0.88 (t, J = 6.6 Hz, 3H) ppm; ^{13}C NMR (100 MHz, $CDCl_3$) δ 144.8 (Cq), 140.7 (Cq), 132.9 (CH), 130.2 (CH), 117.4 (CH_2), 113.7 (CH_2), 32.1 (CH_2), 30.0 (CH_2), 29.8 (2×CH_2), 29.7 (CH_2), 29.5 (CH_2), 29.5 (CH_2), 28.6 (CH_2), 22.9 (CH_2), 22.2 (CH_3), 14.3 (CH_3) ppm; IR (thin film): ν_{max} = 2955, 2923, 2853, 1631, 1456 cm^{-1}; LRMS: (EI+): m/z (%): 234 ([M]$^{+\bullet}$, 8), 219 ([M–CH_3]$^{+\bullet}$, 8), 79 (100); HRMS (EI+): calculated for $C_{17}H_{30}$: 234.2348; found: 234.2356.

((1*E*,3*Z*,5*E*)-3-(Prop-1-en-2-yl)hexa-1,3,5-triene-1,6-diyl)dibenzene (4l-*Z*)

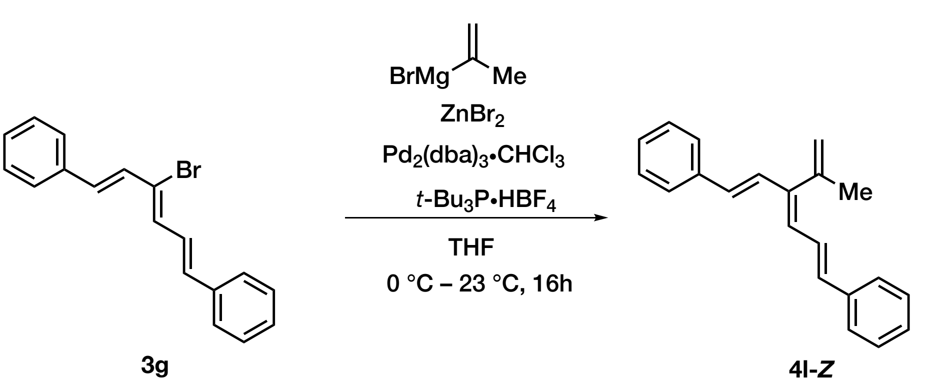

Following **Standard Procedure C**, the reaction mixture containing monobromodiene **3g** (200 mg, 0.643 mmol), $Pd_2(dba)_3$•$CHCl_3$ (27 mg, 0.026 mmol), *t*-Bu_3P•HBF_4 (15 mg, 0.051 mmol) $ZnBr_2$ (2.1 mL, 1.22 M in THF, 2.57 mmol) and isopropenyl magnesium bromide (3.8 mL, 0.51 M in THF, 1.93 mmol) in THF (10 mL) was stirred at 23 °C for 16h. After work up, purification by flash column chromatography (6 g SiO_2, 0.75% EtOAc in PS 40–60) gave dendralene **4l-*Z*** as a yellow solid (125 mg, 0.459 mmol, 71%, >90% retention). R_f = 0.53 (5% EtOAc in PS 40–60); m.p. 141 – 143 °C (hexane:CH_2Cl_2, 8:2); ^{1}H NMR (400 MHz, $CDCl_3$) δ 7.48 – 7.40 (m, 4H), 7.36 – 7.29 (m, 4H), 7.25 – 7.19 (m, 2H), 7.08 (dd, J = 15.6, 11.2 Hz, 1H), 6.84 (d, J = 16.0 Hz, 1H), 6.63 (d, J = 15.6 Hz, 1H), 6.58 (d, J = 16.0 Hz, 1H), 6.32 (d, J = 11.2 Hz, 1H), 5.37 (s, 1H), 4.92 (s, 1H), 2.01 (s, 3H) ppm; ^{13}C NMR (100 MHz, $CDCl_3$) δ 145.4 (Cq), 141.9 (Cq), 137.8 (Cq), 137.7 (Cq), 133.2 (CH), 130.6 (CH), 130.5 (CH), 130.2 (CH), 128.8 (4×CH), 127.7 (CH), 127.5 (CH), 126.7 (CH), 126.7 (2×CH), 126.6 (2×CH), 116.6 (CH_2), 23.9 (CH_3) ppm; IR (thin film): ν_{max} = 3058, 3022, 2961, 2936, 1880, 1813, 1593 cm^{-1}; LRMS: (EI+): m/z (%): 272 ([M]$^{+\bullet}$, 100), 257 ([M–CH_3]$^{+\bullet}$, 17); HRMS (EI+): calculated for $C_{21}H_{20}$: 272.1565; found: 272.1566.

Standard Procedure D: Second Cross-Coupling with $PdCl_2$(dppf)·Toluene (Inversion)

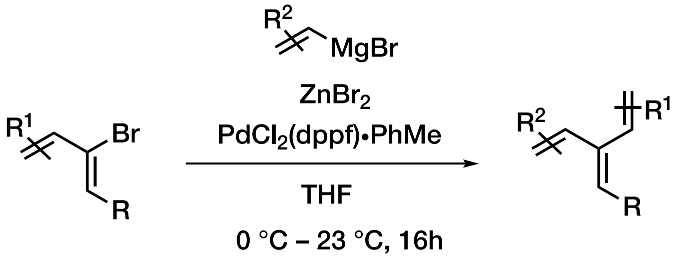

1,1'-Bis(diphenylphosphino)ferrocene palladium(II) chloride•toluene (3 – 5 mol%) and monobromodiene (1.0 mol equiv) in THF (50 mL/g monobromo diene precursor) was purged with N_2 for 5 minutes. The reaction mixture was cooled to 0 °C, $ZnBr_2$ (4.0 –10.0 mol equiv, 0.96 – 1.23 M solution in THF) was added, followed by dropwise addition of the Grignard reagent (3.0 – 8.0 mol equiv, 0.46 – 0.86 M solution in THF) over 5 minutes. The resulting heterogeneous reaction mixture was warmed to room temperature and stirred for 16h until complete by ^{1}H NMR analysis. Saturated aqueous NH_4Cl (5 mL/g monobromodiene precursor) was added, the reaction mixture was filtered through a pad of Celite, then the filtrate was diluted with CH_2Cl_2 (50 mL/g monobromodiene precursor) and water (100 mL/g monobromodiene precursor). The aqueous and organic layers were separated and the aqueous layer was extracted with CH_2Cl_2 (10 mL/g

monobromodiene precursor × 3). The combined organic layer was washed with saturated brine (20 mL/g monobromodiene precursor), dried over Na_2SO_4 and concentrated under reduced pressure.

(*E*)-(3-Methyl-2-vinylbuta-1,3-dien-1-yl)cyclohexane (4m-*E*)

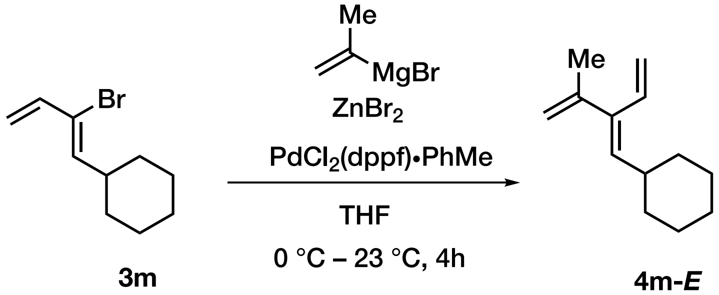

3m **4m-*E***

Following **Standard Procedure D**, the reaction mixture containing monobromodiene **3m** (67 mg, 0.31 mmol), 1,1'-bis(diphenylphosphino)ferrocene palladium(II) chloride•toluene (10 mg, 0.013 mmol) $ZnBr_2$ (1.1 mL, 1.09 M in THF, 1.25 mmol) and isopropenyl magnesium bromide (2.0 mL, 0.46 M in THF, 0.934 mmol) in THF (3.5 mL) was stirred at 23 °C for 4h. After work up, purification by flash column chromatography (3 g SiO_2, 100% PS 40–60) gave dendralene **4m-*E*** as a colourless liquid (31 mg, 0.18 mmol, 57%, >95% inversion). R_f = 0.63 (100% PS 40–60); ^{1}H NMR (400 MHz, $CDCl_3$) δ 6.48 (dd, J = 17.6, 10.8 Hz, 1H), 5.34 (d, J = 9.2 Hz, 1H), 5.23 (d, J = 10.8 Hz, 1H), 5.18 (dd, J = 17.6, 2.0 Hz, 1H), 4.94 (s, 1H), 4.90 (s, 1H), 2.57 – 2.26 (m, 1H), 1.88 (s, 3H), 1.79 – 1.58 (m, 5H), 1.40 – 1.00 (m, 5H) ppm; ^{13}C NMR (100 MHz, $CDCl_3$) δ 144.8 (Cq), 139.1 (Cq), 135.8 (CH), 133.1 (CH), 117.1 (CH_2), 113.9 (CH_2), 37.3 (CH), 33.4 (2×CH_2), 26.2 (CH_2), 26.0 (2×CH_2), 22.2 (CH_3) ppm; IR (thin film): ν_{max} = 3084, 2922, 2850,1630 cm^{-1}; LRMS: (EI+): m/z (%): 176 ($[M]^{+\bullet}$, 31), 161 ($[M–CH_3]^{+\bullet}$, 41), 147 ($[M–CH_3CH_2]^{+\bullet}$, 30), 91 (100); HRMS (EI+): calculated for $C_{13}H_{20}$: 176.1565; found: 176.1569.

(*Z*)-(3-Methyl-2-vinylbuta-1,3-dien-1-yl)cyclohexane (4m-*Z*)

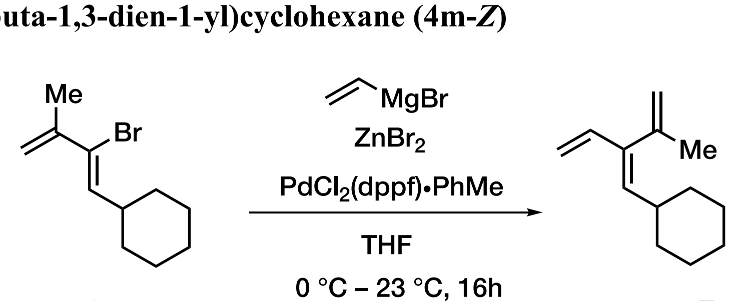

3n **4m-*Z***

Following **Standard Procedure D**, the reaction mixture containing monobromodiene **3n** (110 mg, 0.48 mmol), 1,1'-bis(diphenylphosphino)ferrocene palladium(II) chloride•toluene (20 mg, 0.024 mmol), $ZnBr_2$ (3.0 mL, 0.96 M in THF, 2.88 mmol) and vinyl magnesium bromide (3.2 mL, 0.75 M in THF, 2.40 mmol) in THF (5.5 mL) was stirred at 23 °C for 16h. After work up,

purification by flash column chromatography (4 g SiO_2, 100% PS 40–60) gave dendralene **4m-*Z*** as a colourless liquid (35 mg, 0.199 mmol, 41%, >95% inversion). R_f = 0.71 (100% PS 40–60); ^{1}H NMR (400 MHz, $CDCl_3$) δ 6.30 (dd, J = 17.6, 10.8 Hz, 1H), 5.24 (d, J = 10.0 Hz, 1H), 5.12 (s, 1H), 5.06 (d, J = 17.6 Hz, 1H), 4.95 (d, J = 10.8 Hz, 1H), 4.69 (s, 1H), 2.30 – 2.16 (m, 1H), 1.84 (s, 3H), 1.75 – 1.55 (m, 5H), 1.33 – 1.00 (m, 5H) ppm; ^{13}C NMR (100 MHz, $CDCl_3$) δ 142.1 (Cq), 141.5 (Cq), 139.1 (CH), 137.7 (CH), 114.8 (CH_2), 113.0 (CH_2), 38.1 (CH), 33.4 (2×CH_2), 26.2 (CH_2), 25.9 (2×CH_2), 23.7 (CH_3) ppm; IR (thin film): ν_{max} = 2922, 2850, 1726, 1448 cm^{-1}; LRMS: (EI+): m/z (%): 176 ($[M]^{+\bullet}$, 31), 161 ($[M–CH_3]^{+\bullet}$, 44), 147 ($[M–CH_3CH_2]^{+\bullet}$, 20), 91 (100); HRMS (EI+): calculated for $C_{13}H_{20}$: 176.1565; found: 176.1569.

(*E*)-2-Methyl-3-vinyltetradeca-1,3-diene (4k-*E*)

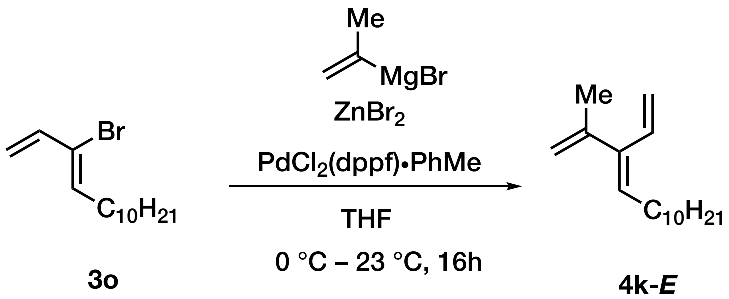

Following **Standard Procedure D**, the reaction mixture containing monobromodiene **3o** (100 mg, 0.366 mmol), 1,1'-bis(diphenylphosphino)ferrocene palladium(II) chloride•toluene (9 mg, 0.015 mmol) $ZnBr_2$ (1.5 mL, 1.22 M in THF, 1.83 mmol) and isopropenyl magnesium bromide (3.2 mL, 0.46 M in THF, 1.46 mmol) in THF (5.0 mL) was stirred at 23 °C for 16h. After work up, purification by flash column chromatography (5 g SiO_2, 100% PS 40–60) gave dendralene **4k-*E*** as a colourless liquid (70 mg, 0.30 mmol, 82%, >95% inversion). R_f = 0.67 (100% PS 40–60); ^{1}H NMR (400 MHz, $CDCl_3$) δ 6.46 (dd, J = 17.6, 11.2 Hz, 1H), 5.53 (t, J = 7.5 Hz, 1H), 5.25 (d, J = 11.2 Hz, 1H), 5.16 (dd, J = 17.6, 2.0 Hz, 1H), 4.94 (s, 1H), 4.91 (s, 1H), 2.18 (q, J = 7.4 Hz, 2H), 1.89 (s, 3H), 1.50 – 1.18 (m, 16H), 0.88 (t, J = 6.6 Hz, 3H) ppm; ^{13}C NMR (100 MHz, $CDCl_3$) δ 144.8 (Cq), 140.7 (Cq), 132.9 (CH), 130.2 (CH), 117.4 (CH_2), 113.7 (CH_2), 32.1 (CH_2), 30.0 (CH_2), 29.8 (2×CH_2), 29.7 (CH_2), 29.5 (CH_2), 29.5 (CH_2), 28.6 (CH_2), 22.9 (CH_2), 22.2 (CH_3), 14.3 (CH_3) ppm; IR (thin film): ν_{max} = 2955, 2923, 2853, 1631, 1456 cm^{-1}; LRMS: (EI+): m/z (%): 234 ($[M]^{+\bullet}$, 8), 219 ($[M–CH_3]^{+\bullet}$, 8), 79 (100); HRMS (EI+): calculated for $C_{17}H_{30}$: 234.2348; found: 234.2356.

(*Z*)-2-Methyl-3-vinyltetradeca-1,3-diene (4k-*Z*)

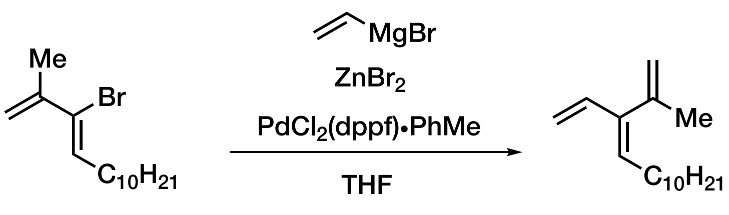

Following **Standard Procedure D**, the reaction mixture containing monobromodiene **3p** (110 mg, 0.383 mmol), 1,1'-bis(diphenylphosphino)ferrocene palladium(II) chloride•toluene (13 mg, 0.015 mmol) $ZnBr_2$ (3.1 mL, 1.22 M in THF, 3.83 mmol) and vinyl magnesium bromide (4.1 mL, 0.75 M in THF, 3.06 mmol) in THF (5.5 mL) was stirred at 23 °C for 16h. After work up, purification by flash column chromatography (6 g SiO_2, 100% PS 40–60) gave dendralene **4k-*Z*** as a colourless liquid (62 mg, 0.26 mmol, 69%, >95% inversion). R_f = 0.67 (100% PS 40–60); ^{1}H NMR (400 MHz, $CDCl_3$) δ 6.29 (dd, J = 17.2, 10.4 Hz, 1H), 5.42 (t, J = 7.5 Hz, 1H), 5.14 (s, 1H), 5.06 (d, J = 17.2 Hz, 1H), 4.95 (d, J = 10.4 Hz, 1H), 4.69 (s, 1H), 2.08 (q, J = 7.3 Hz, 2H), 1.85 (s, 3H), 1.26 (m, 16H), 0.88 (t, J = 6.8 Hz, 3H) ppm; ^{13}C NMR (100 MHz, $CDCl_3$) δ 143.3 (Cq), 141.9 (Cq), 138.9 (CH), 132.1 (CH), 115.0 (CH_2), 112.8 (CH_2), 32.1 (CH_2), 30.0 (CH_2), 29.8 (CH_2), 29.8 (CH_2), 29.7 (CH_2), 29.6 (CH_2), 29.5 (CH_2), 29.1 (CH_2), 23.3 (CH_3), 22.9 (CH_2), 14.3 (CH_3) ppm; IR (thin film): ν_{max} = 2956, 2922, 2853, 1804, 1624 cm^{-1}; LRMS: (EI+): m/z (%): 234 ([M]$^{+\bullet}$, 6), 219 ([M–CH_3]$^{+\bullet}$, 6), 79 (100); HRMS (EI+): calculated for $C_{17}H_{30}$: 234.2348; found: 234.2351.

(*E*)-1-Methoxy-4-(3-methyl-2-vinylbuta-1,3-dien-1-yl)benzene (4n-*E*)

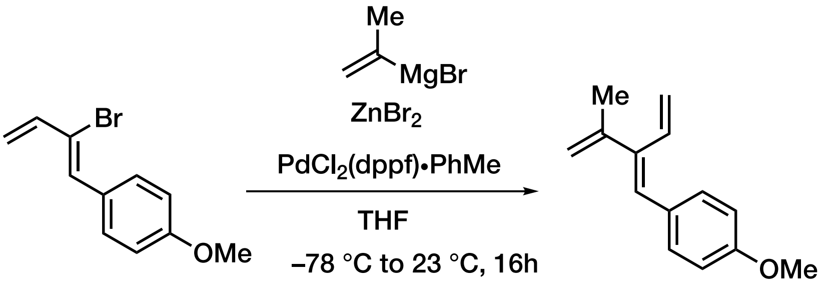

Following **Standard Procedure D**, isopropenyl magnesium bromide (12.8 mL, 0.56 M in THF, 7.19 mmol) was added to the reaction mixture containing monobromodiene **3a** (430 mg, 1.80 mmol), 1,1'-bis(diphenylphosphino)ferrocene palladium(II) chloride•toluene (74 mg, 0.09 mmol) and $ZnBr_2$ (7.9 mL, 1.14 M in THF, 8.99 mmol) at −78 °C. The reaction mixture was warmed to 23 °C and continued stirring for 16h. After work up, purification by flash column chromatography

(15 g SiO_2, 1% EtOAc in PS 40–60) gave dendralene **4n-*E*** as a colourless liquid (266 mg, 1.33 mmol, 74%, >95% inversion). R_f = 0.51 (5% EtOAc in PS 40–60); ^{1}H NMR (400 MHz, $CDCl_3$) δ 7.29 (d, J = 8.8 Hz, 2H), 6.87 (d, J = 8.8 Hz, 2H), 6.67 (dd, J = 18.4, 10.4 Hz, 1H), 6.46 (s, 1H), 5.34 (s, 1H), 5.32 – 5.28 (m, 1H), 5.09 (s, 1H), 5.07 (s, 1H), 3.82 (s, 3H), 1.98 (s, 3H) ppm; ^{13}C NMR (100 MHz, $CDCl_3$) δ 158.6 (Cq), 145.4 (Cq) 141.3 (Cq), 133.7 (CH), 131.1 (2×CH), 130.1 (Cq), 127.4 (CH), 118.8 (CH_2), 115.2 (CH_2), 113.6 (2×CH), 55.4 (CH_3), 22.4 (CH_3) ppm; IR (thin film): ν_{max} = 2952, 2835, 1605, 1508 cm^{-1}; LRMS: (EI+): m/z (%): 200 ($[M]^{+\bullet}$, 64), 199 ($[M–H]^{+\bullet}$, 25), 185 ($[M–CH_3]^{+\bullet}$, 100); HRMS (EI+): calculated for $C_{14}H_{16}O$: 200.1201; found: 200.1204.

(*Z*)-1-Methoxy-4-(3-methyl-2-vinylbuta-1,3-dien-1-yl)benzene (4n-*Z*)

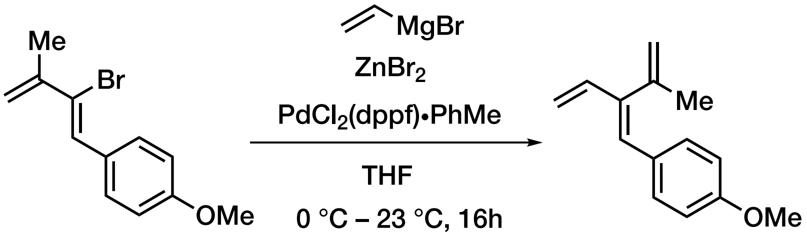

3b **4n-*Z***

Following **Standard Procedure D**, the reaction mixture containing monobromodiene **3b** (90 mg, 0.356 mmol), 1,1'-bis(diphenylphosphino)ferrocene palladium(II) chloride•toluene (15 mg, 0.018 mmol) $ZnBr_2$ (2.3 mL, 1.23 M in THF, 2.84 mmol) and vinyl magnesium bromide (2.5 mL, 0.86 M in THF, 2.13 mmol) in THF (4.5 mL) was stirred at 23 °C 16h. After work up, purification by flash column chromatography (3 g SiO_2, 1% EtOAc in PS 40–60) gave dendralene **4n-*Z*** as a colourless liquid (40 mg, 0.20 mmol, 56%, >95% inversion). R_f =0.56 (5% EtOAc in PS 40–60); ^{1}H NMR (400 MHz, $CDCl_3$) δ 7.47 (d, J = 8.8 Hz, 2H), 6.84 (d, J = 8.8 Hz, 2H), 6.46 (dd, J = 16.8, 10.4 Hz, 1H), 6.26 (s, 1H), 5.33 – 5.22 (m, 2H), 5.08 (d, J = 10.4 Hz, 1H), 4.91 (s, 1H), 3.81 (s, 3H), 1.90 (s, 3H) ppm; ^{13}C NMR (100 MHz, $CDCl_3$) δ 158.8 (Cq), 142.0 (Cq), 141.6 (Cq), 140.2 (CH), 130.4 (2×CH), 129.7 (Cq), 128.8 (CH), 116.5 (CH_2), 114.0 (CH_2), 113.8 (2×CH), 55.4 (CH_3), 22.6 (CH_3) ppm; IR (thin film): ν_{max} = 3078, 3002, 2961, 2835, 1595, 1507 cm^{-1}; LRMS: (EI+): m/z (%): 200 ($[M]^{+\bullet}$, 41), 185 ($[M–CH_3]^{+\bullet}$, 100), 170 ($[M–2×CH_3]^{+\bullet}$, 29), 154 ($[M–OCH_3CH_3]^{+\bullet}$, 21); HRMS (EI+): calculated for $C_{14}H_{16}O$: 200.1201; found: 200.1199.

One flask synthesis of the geometrical isomers

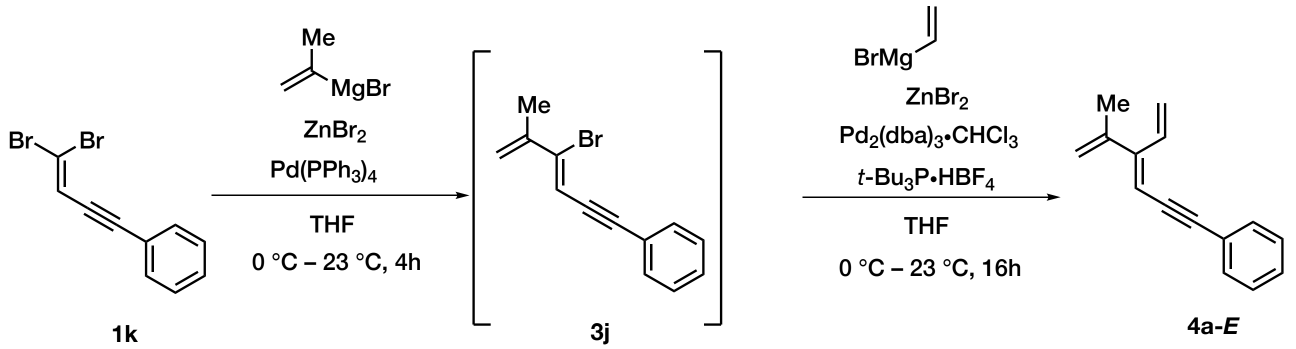

Following **Standard Procedure B**, the reaction mixture containing dibromide **1k** (154 mg, 0.539 mmol), $Pd(PPh_3)_4$ (19 mg, 0.016mmol) $ZnBr_2$ (0.67 mL, 1.23 M in THF, 0.808 mmol) and isopropenyl magnesium bromide (1.3 mL, 0.49 M in THF, 0.646 mmol) in THF (10 mL) was stirred at 23 °C for 4h. To the above reaction mixture, $Pd_2(dba)_3{\bullet}CHCl_3$ (22 mg, 0.022 mmol), *t*-$Bu_3P{\bullet}HBF_4$ (13 mg, 0.043 mmol) $ZnBr_2$ (1.7 mL, 1.23 M in THF, 2.15 mmol) and vinyl magnesium bromide (2.3 mL, 0.70 M in THF, 1.62 mmol) were added at 0 °C and the reaction mixture was stirred at 23 °C for 16h. After work up, purification by flash column chromatography (10 g SiO_2, 100% PS 40–60) gave dendralene **4a-*E*** as a pale yellow liquid (75 mg, 0.39 mmol, 72%, isomer ratio 95:5).

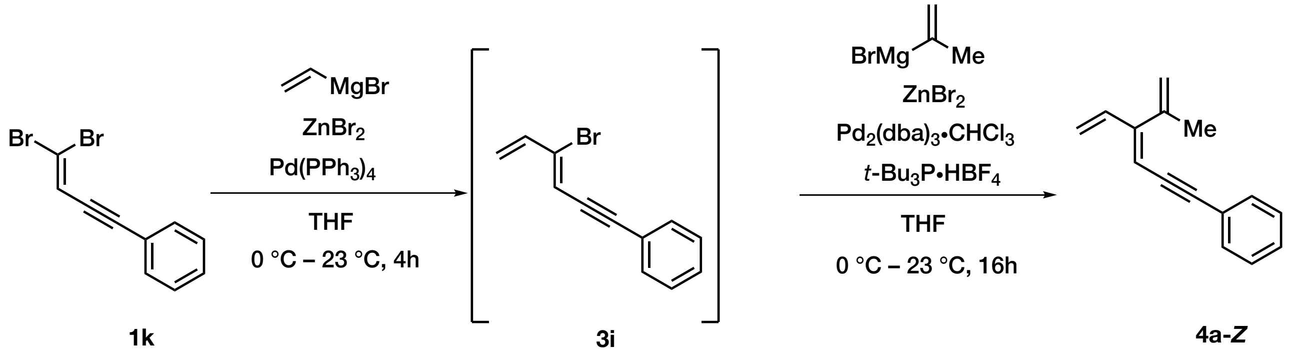

Following **Standard Procedure B**, the reaction mixture containing dibromide **1k** (110 mg, 0.385 mmol), $Pd(PPh_3)_4$ (13 mg, 0.012 mmol) $ZnBr_2$ (0.47 mL, 1.22 M in THF, 0.577 mmol) and vinyl magnesium bromide (0.63 mL, 0.73 M in THF, 0.462 mmol) in THF (10 mL) was stirred at 23 °C for 4h. To the above reaction mixture, $Pd_2(dba)_3{\bullet}CHCl_3$ (12 mg, 0.12 mmol), *t*-$Bu_3P{\bullet}HBF_4$ (8 mg, 0.027 mmol) $ZnBr_2$ (1.3 mL, 1.22 M in THF, 1.54 mmol) and isopropenyl magnesium bromide (2.3 mL, 0.51 M in THF, 1.15 mmol) were added at 0 °C and stirred the reaction mixture at 23 °C for 16h. After work up, purification by flash column chromatography (10 g SiO_2, 100% PS 40–60) gave monobromodiene **4a-*Z*** as a pale yellow liquid (50 mg, 0.257 mmol, 67%, isomer ratio 90:10).

((1*E*,3*Z*)-5-Methylene-4-(prop-1-en-2-yl)hepta-1,3,6-trien-1-yl)benzene (10a-*Z*)

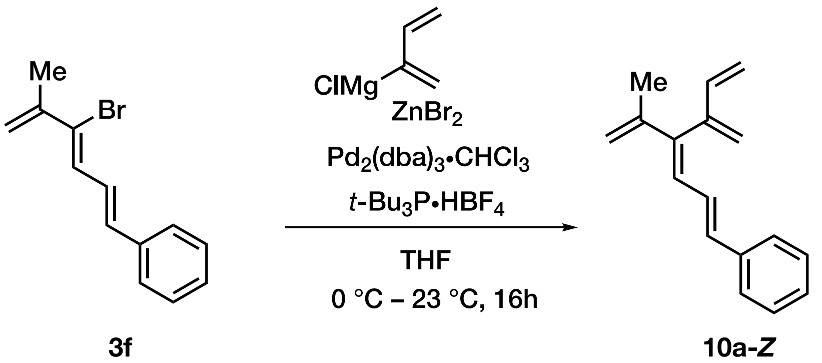

Following **Standard Procedure C**, the reaction mixture containing monobromodiene **3f** (80 mg, 0.321 mmol), $Pd_2(dba)_3$•$CHCl_3$ (13 mg, 0.013 mmol), *t*-Bu_3P•HBF_4 (8 mg, 0.026 mmol) $ZnBr_2$ (1.0 mL, 1.23 M in THF, 1.28 mmol) and chloroprene grignard reagent (1.3 mL, 0.75 M in THF, 0.963 mmol) in THF (4 mL) was stirred at 23 °C for 16h. After work up, purification by flash column chromatography (3 g SiO_2, 100% PS 40–60) gave dendralene **10a-*Z*** as a colourless liquid (53 mg, 0.24 mmol, 74%, >95% retention). R_f = 0.36 (100% PS 40–60);

^{1}H NMR (400 MHz, $(CD_3)_2CO$) δ 7.41 (d, J = 7.2 Hz, 2H), 7.33 (t, J = 7.6 Hz, 2H), 7.28 – 7.16 (m, 1H), 6.91 (dd, J = 15.6, 10.8 Hz, 1H), 6.74 (d, J = 15.6 Hz, 1H), 6.65 – 6.54 (m, 2H), 5.56 (s, 1H), 5.15 – 5.00 (m, 5H), 2.05 (s, 3H) ppm; ^{13}C NMR (100 MHz, $(CD_3)_2CO$) δ 146.7 (Cq), 142.9 (Cq), 142.6 (Cq), 138.9 (CH), 138.5 (Cq), 134.4 (CH), 129.5 (2×CH), 128.4 (CH), 128.3 (CH), 127.9 (CH), 127.2 (2×CH), 120.1 (CH_2), 117.0 (CH_2), 116.4 (CH_2), 20.4 (CH_3) ppm; IR (thin film): ν_{max} = 3083, 3033, 2980, 1944, 1795, 1591 cm^{-1}; LRMS: (EI+): m/z (%): 222 ($[M]^{+•}$, 100), 207 ($[M–CH_3]^{+•}$, 63), 192 ($[M–CH_3CH_2H]^{+•}$, 30); HRMS (EI+): calculated for $C_{17}H_{18}$: 222.1409; found: 222.1408.

(*Z*)-(5-Methylene-4-vinylhepta-3,6-dien-1-yn-1-yl)benzene (10b-*Z*)

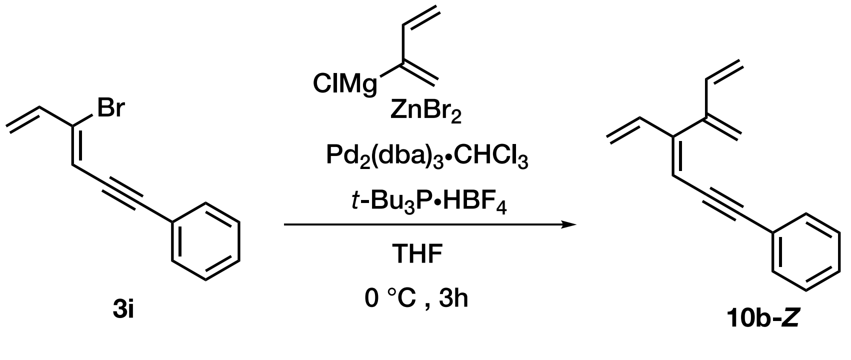

Following **Standard Procedure C**, the reaction mixture containing monobromodiene **3i** (55 mg, 0.236 mmol), $Pd_2(dba)_3$•$CHCl_3$ (10 mg, 0.0094 mmol), *t*-Bu_3P•HBF_4 (6 mg, 0.019 mmol) $ZnBr_2$

(0.77 mL, 1.23 M in THF, 0.94 mmol) and chloroprene grignard reagent (0.94 mL, 0.75 M in THF, 0.71 mmol) in THF (3 mL) was stirred at 0 °C for 3h. After work up, purification by flash column chromatography (3 g SiO_2, 100% PS 40–60) gave dendralene **10b-*Z*** as a colourless liquid (33 mg, 0.160 mmol, 68%, >95% retention). R_f = 0.58 (5% EtOAc in PS 40–60);

^{1}H NMR (400 MHz, $(CD_3)_2CO$) δ 7.39 – 7.30 (m, 5H), 6.62 (dd, J = 17.2, 10.4 Hz, 1H), 6.54 (dd, J = 16.8, 11.2 Hz, 1H), 6.03 (s, 1H), 5.54 (s, 1H), 5.26 (d, J = 17.2 Hz, 1H), 5.20 (d, J = 10.4 Hz, 1H), 5.12 (s, 1H), 5.11 – 5.07 (m, 2H) ppm; ^{13}C NMR (100 MHz, $(CD_3)_2CO$) δ 151.6 (Cq), 145.0 (Cq), 138.3 (CH), 137.5 (CH), 132.1 (2×CH), 129.4 (2×CH), 129.2 (CH), 124.5 (Cq), 119.6 (CH_2), 118.5 (CH_2), 116.6 (CH_2), 112.0 (CH), 96.8 (Cq), 88.7 (Cq) ppm; IR (thin film): ν_{max} = 3089, 3006, 2193, 1822, 1595 cm^{-1}; LRMS: (EI+): m/z (%): 206 ($[M]^{+\bullet}$, 50), 205 ($[M–H]^{+\bullet}$, 100), 191 ($[M–CH_3]^{+\bullet}$, 100), 179 ($[M–C_2H_3]^{+\bullet}$, 31), 165 ($[M–C_3H_5]^{+\bullet}$, 84), 152 ($[M–C_4H_6]^{+\bullet}$, 46); HRMS (EI+): calculated for $C_{16}H_{14}$: 206.1096; found: 206.1090.

(*Z*)-(5-Methylene-4-(prop-1-en-2-yl)hepta-3,6-dien-1-yn-1-yl)benzene (10c-*Z*)

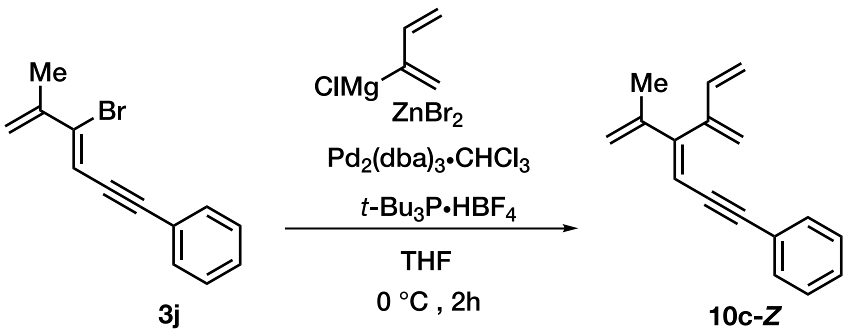

Following **Standard Procedure C**, the reaction mixture containing monobromodiene **3j** (55 mg, 0.223 mmol), $Pd_2(dba)_3$•$CHCl_3$ (9 mg, 0.009 mmol), *t*-Bu_3P•HBF_4 (5 mg, 0.018 mmol) $ZnBr_2$ (0.72 mL, 1.23 M in THF, 0.890 mmol) and chloroprene grignard reagent (0.89 mL, 0.75 M in THF, 0.668 mmol) in THF (3 mL) was stirred at 0 °C for 2h. After work up, purification by flash column chromatography (3 g SiO_2, 100% PS 40–60) gave dendralene **10c-*Z*** as a pale yellow liquid (38 mg, 0.17 mmol, 77%, >95% retention). R_f = 0.24 (100% PS 40–60);

^{1}H NMR (400 MHz, C_6D_6) δ 7.45 (dd, J = 8.0, 1.6 Hz, 2H), 7.02 – 6.90 (m, 3H), 6.43 (dd, J = 17.2, 10.4 Hz, 1H), 5.95 (s, 1H), 5.34 (s, 1H), 5.28 (s, 1H), 5.23 (d, J = 17.2 Hz, 1H), 5.15 (s, 1H), 5.06 (d, J = 10.4 Hz, 1H), 4.97 (s, 1H), 1.72 (s, 3H) ppm; ^{13}C NMR (100 MHz, C_6D_6) δ 152.2 (Cq), 146.3 (Cq), 141.5 (Cq), 137.5 (CH), 131.8 (2×CH), 128.6 (2×CH), 128.2 (CH), 124.6 (Cq), 119.2 (CH_2), 118.4 (CH_2), 116.3 (CH_2), 108.4 (CH), 96.5 (Cq), 89.1 (Cq), 19.8 (CH_3) ppm; IR (thin film): ν_{max} = 3088, 2971, 1813, 1593, 1488 cm^{-1}; LRMS: (EI+): m/z (%): 220 ($[M]^{+\bullet}$, 31), 219 ($[M–H]^{+\bullet}$,

100), 205 ([M–CH_3]$^{+•}$, 56), 191 ([M–C_2H_5]$^{+•}$, 47), 179 ([M–C_3H_5]$^{+•}$, 22), 165 ([M–C_4H_7]$^{+•}$, 36); HRMS (EI+): calculated for $C_{17}H_{16}$: 220.1252; found: 220.1252.

Triene-transmissive Twofold 6*π* -Electrocyclisation of [4]Dendralene

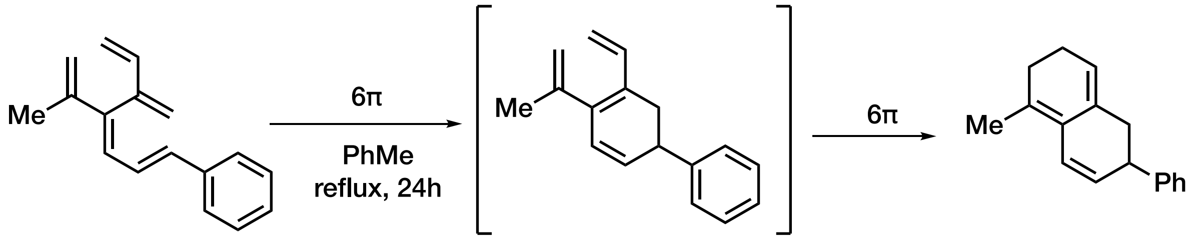

10a-*Z* **11 (not observed)** **12**

[4]dendralene **10a-*Z*** (16 mg, 0.072 mmol) was dissolved in 0.5 mL toluene-*d*[8] and heated under reflux for 24h. The reaction completion was confirmed by ^{1}H NMR spectroscopic analysis. The reaction mixture was concentrated under reduced pressure and purification by flash column chromatography (3 g SiO_2, 100% PS 40–60) gave the bicyclic [3]dendralene **12** as a colourless liquid (15 mg, 0.067 mmol, 94%). R_f =0.25 (100% PS 40–60); ^{1}H NMR (400 MHz, $CDCl_3$) δ 7.35 – 7.27 (m, 2H), 7.24 – 7.15 (m, 3H), 6.61 (d, J = 9.9 Hz, 1H), 5.82 (d, J = 9.8 Hz, 1H), 5.46 (s, 1H), 3.54 (s, 1H), 2.66 (dd, J = 13.7, 5.3 Hz, 1H), 2.41 – 2.28 (m, 1H), 2.27 – 2.04 (m, 4H), 1.94 (s, 3H) ppm; ^{13}C NMR (100 MHz, $CDCl_3$) δ 145.9 (Cq), 133.1 (Cq), 130.5 (Cq), 130.1 (CH), 128.5 (2×CH), 127.7 (2×CH), 126.3 (CH), 125.8 (Cq), 123.9 (CH), 119.0 (CH), 42.7 (CH), 40.1 (CH_2), 30.6 (CH_2), 22.3 (CH_2), 18.7 (CH_3) ppm; IR (thin film): ν_{max} = 3028, 2924, 2851, 1602 cm^{-1}; LRMS: (EI+): m/z (%): 222 ([M]$^{+•}$, 100), 207 ([M–CH_3]$^{+•}$, 63), 129 ([M–$C_6H_5CH_3$]$^{+•}$, 29); HRMS (EI+): calculated for $C_{17}H_{18}$: 222.1409; found: 222.1408.

(3a*R*,6*R*,6a*R*,9a*S*,10a*R*,10b*S*)-2,5,8-trimethyl-6-(phenylethynyl)-3a,4,6,6a,9a,10,10a,10b-octahydroisoindolo[5,6-*e*]isoindole-1,3,7,9(2*H*,8*H*)-tetraone (7)

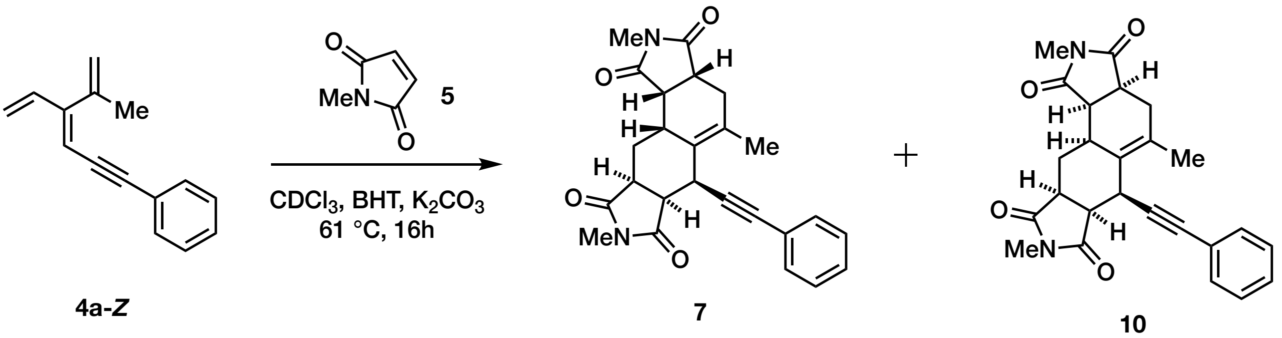

[3]dendralene **4a-*Z*** (14 mg, 0.07 mmol) and NMM **5** (20 mg, 0.18mmol) were dissolved in 0.5 mL $CDCl_3$ containing tiny crystals of BHT and K_2CO_3 and the reaction mixture was heated under reflux for 16h. The reaction completion was confirmed by ^{1}H NMR spectroscopic analysis. The solvent was distilled off to afford the crude material as a mixture of diastereomers (dr = 67:33).

The major adduct **7** was isolated using 40% EtOAc in PS 40–60 as a white solid (16 mg, 0.04 mmol, 53%). R_f = 0.23 (50% EtOAc in PS 40–60); m.p. 211 – 216 °C (recrystallized by slow diffusion method from CH_2Cl_2 and diethyl ether); ^{1}H NMR (400 MHz, CD_2Cl_2) δ 7.37 – 7.16 (m, 5H), 4.19 (d, J = 4.7 Hz, 1H), 3.63 (q, J = 9.7 Hz, 1H), 3.13 (p, J = 8.8 Hz, 2H), 2.94 (s, 3H), 2.88 (s, 3H), 2.78 (brs, 1H), 2.73 – 2.52 (m, 4H), 2.26 (dd, J = 14.7, 5.1 Hz, 1H), 1.84 (s, 3H) ppm; ^{13}C NMR (100 MHz, CD_2Cl_2) δ 181.1 (Cq), 179.4 (Cq), 179.4 (Cq), 178.0 (Cq), 131.9 (Cq), 131.6 (2×CH), 128.8 (3×CH), 128.2 (Cq), 122.8 (Cq), 86.6 (Cq), 84.2 (Cq), 46.3 (CH), 45.0 (CH), 40.9 (CH), 36.6 (CH), 33.8 (CH), 31.2 (CH_2), 29.7 (CH), 25.1 (CH_3), 24.7 (CH_3), 22.6 (CH_2), 19.4 (CH_3) ppm; IR (thin film): ν_{max} = 3055, 2942, 2851, 1774, 1688 cm^{-1}; LRMS: (ESI+): m/z (%): 417 ([M+H]$^{+•}$, 100); HRMS (ESI+): calculated for $C_{25}H_{25}O_4N_2$: 417.1809; found: 417.1813.

The minor adduct **10** was isolated using 10% CH_2Cl_2 in EtOAc as a pale yellow solid (4 mg, 0.01 mmol, 13%). R_f = 0.26 (100% EtOAc); m.p. 230 – 240 °C (decomposition, recrystallized by slow diffusion method from CH_2Cl_2 and diethyl ether). ^{1}H NMR (400 MHz, CD_2Cl_2) δ 7.47 – 7.06 (m, 5H), 4.26 (d, J = 5.3 Hz, 1H), 3.11 (brs, 2H), 3.06 – 2.76 (m, 6H), 2.76 – 2.51 (m, 4H), 2.53 – 2.26 (m, 2H), 2.20 (d, J = 13.7 Hz, 1H), 1.82 (s, 3H) ppm; ^{13}C NMR (100 MHz, CD_2Cl_2) δ 179.6 (Cq), 179.3 (Cq), 177.5 (Cq), 177.1 (Cq), 132.0 (Cq), 131.9 (2×CH), 128.7 (Cq), 128.6 (3×CH), 123.0 (Cq), 86.9 (Cq), 83.9 (Cq), 44.4 (CH), 44.3 (CH), 41.0 (CH), 39.2 (CH), 36.1 (CH), 31.0 (CH_2), 28.0 (CH), 24.9 (CH_3), 24.9 (CH_3), 23.7 (CH_2), 19.6 (CH_3) ppm; IR (thin film):

ν_{max} = 2919, 2849, 1773, 1695 cm^{-1}; LRMS: (ESI+): m/z (%): 439 ([M+Na]$^{+\bullet}$, 100), 417 ([M+H]$^{+\bullet}$, 60); HRMS (ESI+): calculated for $C_{25}H_{25}O_4N_2$: 417.1814; found: 417.1815; calculated for $C_{25}H_{24}O_4N_2Na$: 439.1634; found: 439.1631.

2,8,10a-Trimethyl-6-(phenylethynyl)-3a,4,6,6a,9a,10,10a,10b-octahydroisoindolo[5,6-*e*]isoindole-1,3,7,9(2*H*,8*H*)-tetraone (9)

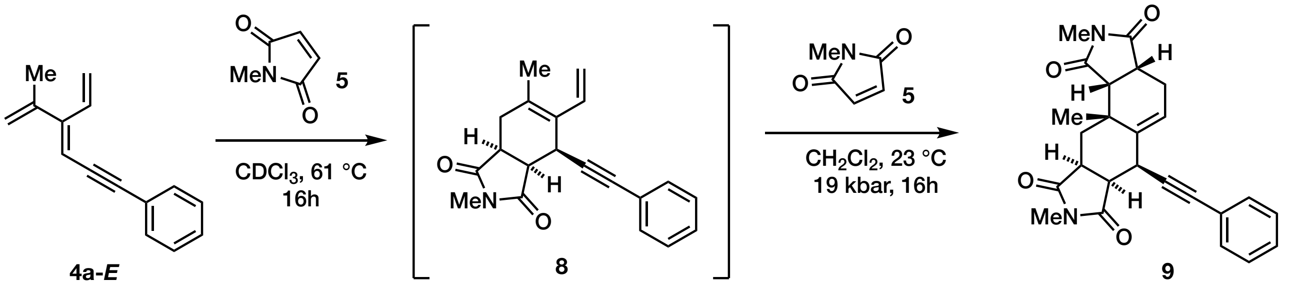

[3]dendralene **4a-*E*** (15 mg, 0.08 mmol) and NMM (10 mg, 0.09mmol) were dissolved in 0.5 mL $CDCl_3$ containing tiny crystals of BHT and K_2CO_3 and heated the reaction mixture under reflux for 16h. The formation of mono Diels–Alder adduct was confirmed by ^{1}H NMR spectroscopic analysis. The solvent was removed by distillation and 0.6 mL CH_2Cl_2 and NMM (26 mg, 0.23 mmol) were added. The reaction mixture was subjected to 19 kbar pressure at 23°C for 16h. Reaction completion was confirmed by ^{1}H NMR analysis. The solvent was distilled off to give the crude product as a single diastereomer. The crude reaction mixture was purified by flash column chromatography (5g SiO_2, 40% EtOAc in PS 40–60) to give the twofold adduct **9** as a white solid (22 mg, 0.053 mmol, 68%). R_f = 0.42 (50% EtOAc in PS 40–60); m.p. 209 – 213 °C (Recrystallized by slow diffusion method from CH_2Cl_2 and diethyl ether);

^{1}H NMR (400 MHz, C_6D_6) δ 7.31 (brs, 2H), 6.91 (brs, 3H), 5.05 (d, J = 6.5 Hz, 1H), 3.65 (d, J = 5.1 Hz, 1H), 3.37 (q, J = 9.7 Hz, 1H), 2.91 (dd, J = 14.8, 9.0 Hz, 1H), 2.75 (s, 3H), 2.61 (s, 3H), 2.56 – 2.44 (m, 1H), 2.44 – 2.18 (m, 3H), 1.95 (d, J = 8.7 Hz, 1H), 1.84 (dd, J = 16.5, 8.9 Hz, 1H), 0.91 (s, 3H) ppm; ^{13}C NMR (100 MHz, C_6D_6) δ 180.1 (Cq), 178.6 (Cq), 177.4 (Cq), 176.4 (Cq), 140.5 (Cq), 131.6 (2×CH), 128.8 (2×CH), 128.7 (CH), 122.9 (Cq), 122.5 (CH), 86.7 (Cq), 85.7 (Cq), 51.4 (CH), 45.1 (CH), 39.3 (CH), 37.3 (CH), 36.6 (Cq), 36.5 (CH), 32.4 (CH_2), 29.5 (CH_3), 24.7 (CH_3), 24.4 (CH_3), 24.0 (CH_2) ppm; IR (thin film): ν_{max} = 3056, 2942, 2864, 1773, 1696, 1489 cm^{-1}; LRMS: (ESI+): m/z (%): 439 ([M+Na]$^{+\bullet}$, 100), 417 ([M+H]$^{+\bullet}$, 20); HRMS (ESI+): calculated for $C_{25}H_{24}O_4N_2Na$: 439.1628; found: 439.1612.

X-ray Crystallography

Single crystal X-ray data for compounds were collected on a Supernova diffractometer using Cu *Kα* radiation, *λ* = 1.54184 Å and XCalibur with Mo *Kα* radiation, *λ* = 0.71073 Å radiation. Data reduction was performed using the CrysAlis PRO package.[21] Structure solutions for all compounds were determined by ShelXT,[22] and the structures refined using ShelXL in the OLEX2 program package.[23,24]

1. Compound 4l-*Z*

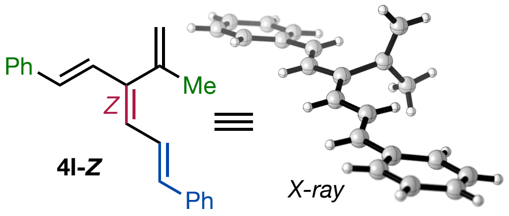

$C_{21}H_{20}$, M = 272.37, T = 150 K, monoclinic, space group $P2_1$, Z = 2, a = 8.5944(1), b = 5.9312(1), c = 16.3498(2) Å, β = 104.547(1)°, V = 806.71(2) Å^3, D_x = 1.121 Mg m^{-3}, 3077 unique data ($2\theta_{max}$ = 147.4°), 3018 with $I > 2\sigma(I)$; R = 0.045, Rw = 0.127, S = 1.07. CCDC 1902664.

2. Compound 4e-*Z*

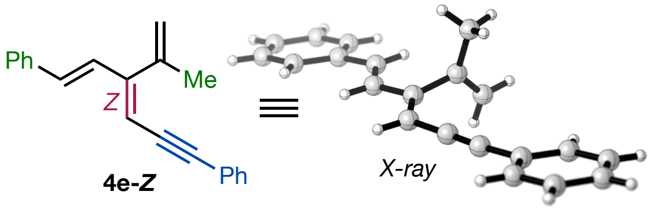

$C_{21}H_{18}$, M = 270.35, T = 294 K, orthorhombic, space group $Pna2_1$, Z = 4, a = 10.9002(3), b = 20.0267(4), c = 7.6356(1) Å, V = 1666.81(6) Å^3, D_x = 1.077 Mg m^{-3}, 3141 unique data ($2\theta_{max}$ = 145.4°), 2705 with $I > 2\sigma(I)$; R = 0.051, Rw = 0.161, S = 1.03. CCDC 1902662.

3. Compound 3g

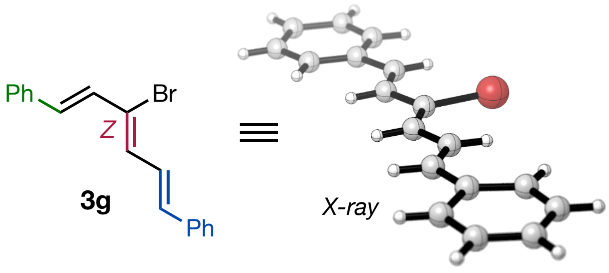

$C_{18}H_{15}Br$, M = 311.21, T = 150 K, triclinic, space group P-1 (No. 2), Z = 4, a = 9.6214(3), b = 10.8141(3), c = 15.9731(5) Å, α = 76.964(3), β = 79.006(3), γ = 64.023(3)°, V = 1447.50(7) Å^3, D_x = 1.428 Mg m^{-3}, 5613 unique data ($2\theta_{max}$ = 147.6°), 5389 with $I > 2\sigma(I)$; R = 0.092, Rw = 0.230, S = 1.09. CCDC 1902661.

4. Compound 3c

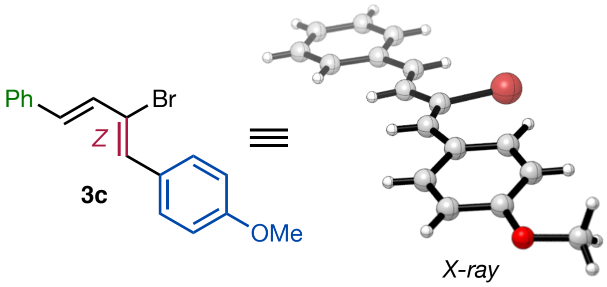

$C_{17}H_{15}BrO$, M = 315.20, T = 150 K, orthorhombic, space group $P2_12_12_1$, Z = 8, a = 5.79782(9), b = 7.59133(14), c = 63.3743(11) Å, V = 2789.30(8) Å^3, D_x = 1.501 Mg m^{-3}, 5656 unique data ($2\theta_{max}$ = 148.4°), 5612 with $I > 2\sigma(I)$; R = 0.060, Rw = 0.131, S = 1.31. CCDC 1902663.

5. Compound 7

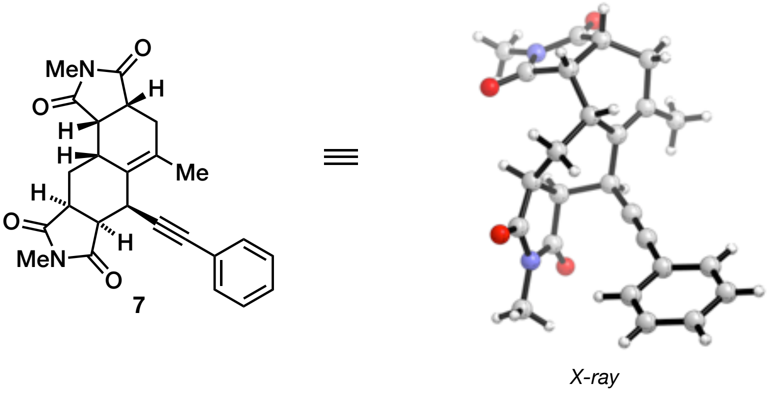

$C_{25}H_{24}N_2O_4$, M = 416.46, monoclinic, $C2/c$ (No. 15), a = 21.8655(7) Å, b = 6.7309(2) Å, c = 29.0129(8) Å, β = 100.540(3)°, $\alpha = \gamma$ = 90°, V = 4197.9(2) Å^3, T = 150.00(10) K, Z = 8, Z' = 1, $\mu(MoK_\alpha)$ = 0.090 mm^{-1}, 36549 reflections measured, 5529 unique (R_{int} = 0.0213) which were used in all calculations. The final wR_2 was 0.1174 (all data) and R_1 was 0.0446 ($I > 2\sigma(I)$). CCDC 1922944.

6. Compound 10

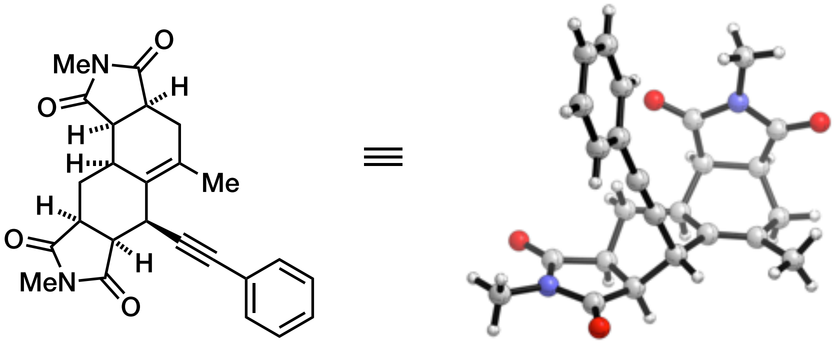

10

$C_{25}H_{24}N_2O_4$, M = 416.46, tetragonal, I-4 (No. 82), a = 18.1165(5) Å, b = 18.1165(5) Å, c = 12.8619(11) Å, $\alpha = \beta = \gamma$ = 90°, V = 4221.4(4) Å^3, T = 150.00(10) K, Z = 8, Z' = 1, μ(CuK_α) = 0.725 mm^{-1}, 12779 reflections measured, 3605 unique (R_{int} = 0.0488) which were used in all calculations. The final wR_2 was 0.0871 (all data) and R_1 was 0.0405 (I >2σ(I)). CCDC 1922943.

7. Compound 9

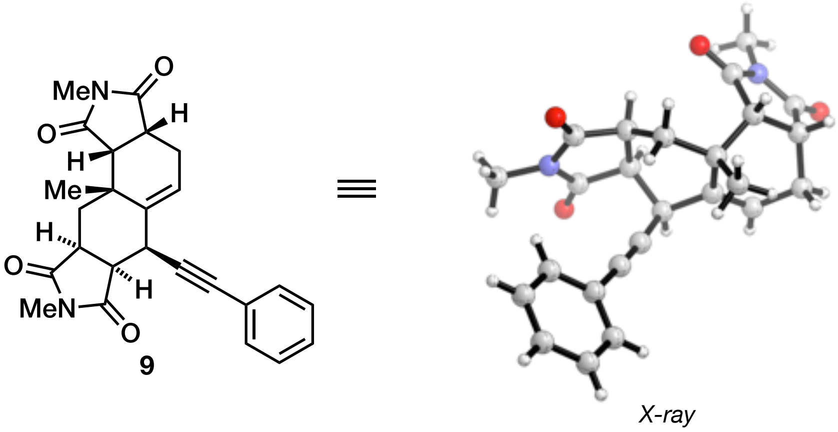

9

$C_{25}H_{24.34}N_2O_{4.17}$, M = 419.53, monoclinic, C2 (No. 5), a = 22.1402(8) Å, b = 6.8047(2) Å, c = 14.2360(5) Å, β = 100.710(4)°, α = γ = 90°, V = 2107.40(13) Å^3, T = 150.01(10) K, Z = 4, Z' = 1, μ(MoK_α) = 0.091 mm^{-1}, 18911 reflections measured, 5250 unique (R_{int} = 0.0222) which were used in all calculations. The final wR_2 was 0.0904 (all data) and R_1 was 0.0355 (I > 2σ(I)). CCDC 1922945.

References

[1] A. B. Pangborn, M. A. Giardello, R. H. Grubbs, R. K. Rosen, F. J. Timmers, *Organometallics* **1996**, *15*, 1518-1520.

[2] J. K. Sliwowski, E. Caspi, *Journal of Steroid Biochemistry* **1977**, *8*, 47-49.

[3] E. N. Ndunda, V. O. Madadi, B. Mizaikoff, *Environ. Sci.: Processes Impacts.* **2015**, *17*, 2101-2109.

[4] E. C. Juenge, S. E. Cook, *J. Am. Chem. Soc.* **1959**, *81*, 3578-3580.

[5] B. E. Love, E. G. Jones, *J. Org. Chem.* **1999**, *64*, 3755-3756.

[6] S. S. Zalesskiy, V. P. Ananikov, *Organometallics* **2012**, *31*, 2302-2309.

[7] L. Brandsma, Vasilevsky, S. F. and Verkruijsse, H. D., *Application of Transition Metal Catalysts in Organic Synthesis*, Springer-Verlag, Berlin, **1998, pp** 5.

[8] D. H. Huh, J. S. Jeong, H. B. Lee, H. Ryu, Y. G. Kim, *Tetrahedron* **2002**, *58*, 9925-9932.

[9] X. Y. Chen, L. Wang, M. Frings, C. Bolm, *Org. Lett.* **2014**, *16*, 3796-3799.

[10] (a) D. Nitsch, S. M. Huber, A. Pöthig, A. Narayanan, G. A. Olah, G. K. S. Prakash, T. Bach, *J. Am. Chem. Soc.* **2014**, *136*, 2851-2857; (b) A. Rosiak, W. Frey, J. Christoffers, *Eur. J. Org. Chem.* **2006**, 4044-4054.

[11] (a) A. K. Morri, Y. Thummala, V. R. Doddi, *Org. Lett.* **2015**, *17*, 4640-4643; (b) Y. B. Malysheva, S. Y. Buchvalova, E. V. Svirshchevskaya, V. V. Fokin, A. Y. Fedorov, *Synlett* **2013**, *24*, 1772-1776.

[12] P. Pawluć, G. Hreczycho, J. Walkowiak, B. Marciniec, *Synlett* **2007**, *13*, 2061-2064.

[13] N. B. Desai, N. McKelvie, F. Ramirez, *J. Am. Chem. Soc.* **1962**, *84*, 1745-1747.

[14] K. Miura, G. Inoue, H. Sasagawa, H. Kinoshita, J. Ichikawa, A. Hosomi, *Org. Lett.* **2009**, *11*, 5066-5069.

[15] M. Yoshida, H. Otaka, T. Doi, *Eur. J. Org. Chem.* **2014**, 6010-6016.

[16] I. N. Michaelides, B. Darses, D. J. Dixon, *Org. Lett.* **2011**, *13*, 664-667.

[17] (a) E. Métay, Q. Hu, E. Negishi, *Org. Lett.* **2006**, *8*, 5773-5776; (b) J. Uenishi, R. Kawahama, O. Yonemitsu, J. Tsuji, *J. Org. Chem.* **1996**, *61*, 5716-5717.

[18] (a) P. Singh, M. Kaur, W. Holzer, *Eur. J. Med. Chem.* **2010**, *45*, 4968-4982; (b) K. Tanaka, T. Kobayashi, H. Mori, S. Katsumura, *J. Org. Chem.* **2004**, *69*, 5906-5925.

[19] A. P. Dieskau, M. S. Holzwarth, B. Plietker, *J. Am. Chem. Soc.* **2012**, *134*, 5048-5051.

[20] K. Aikawa, Y. t. Hioki, N. Shimizu, K. Mikami, *J. Am. Chem. Soc.* **2011**, *133*, 20092-20095.

[21] Agilent CrysAlis PRO. Agilent Technologies Ltd, Yarnton, Oxfordshire, England, **2014**.

[22] G. M. Sheldrick, *Acta Cryst.* **2015**, *A71*, 3–8.

[23] G. M. Sheldrick, *Acta Cryst.* **2015**, *C71*, 3–8.

[24] O. V. Dolomanov, L. J. Bourhis, R. J. Gildea, J. A. K. Howard, H. Puschmann, J. Appl. Cryst. **2009**, *42*, 339-341.

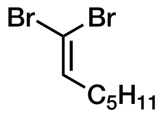

1f

400 MHz, $CDCl_3$

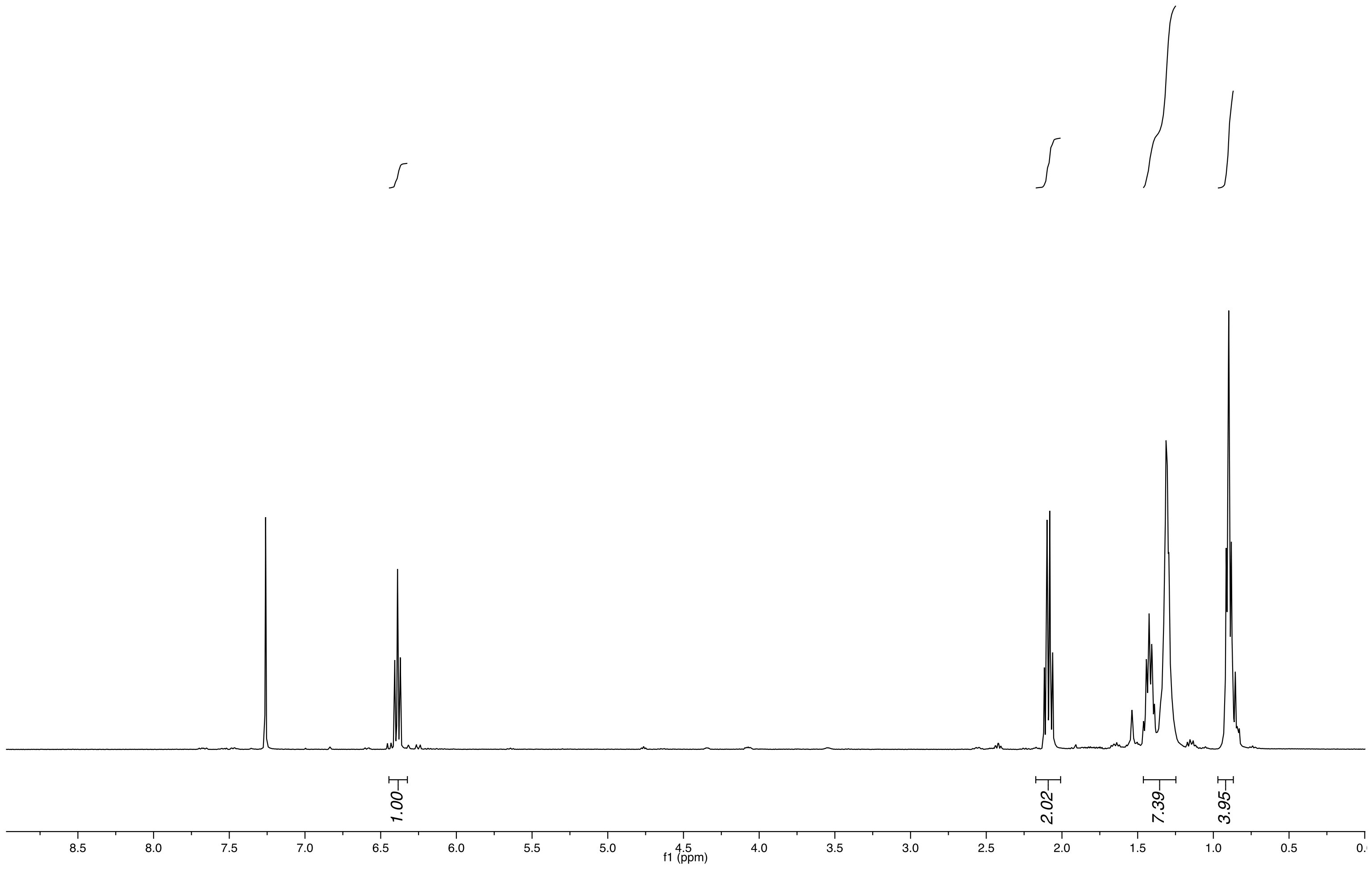

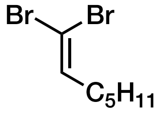

1f

100 MHz, $CDCl_3$

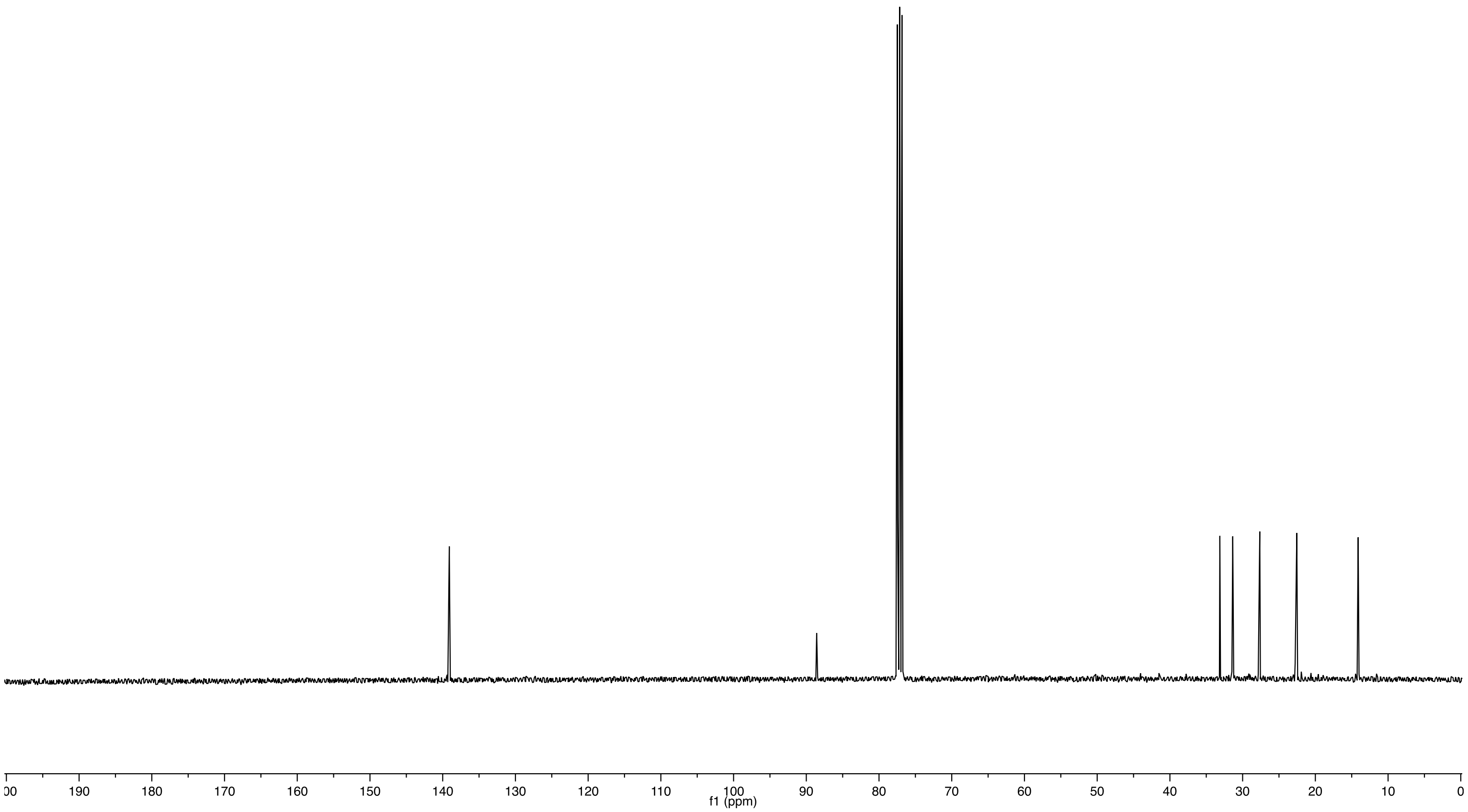

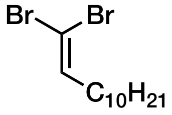

1g

400 MHz, $CDCl_3$

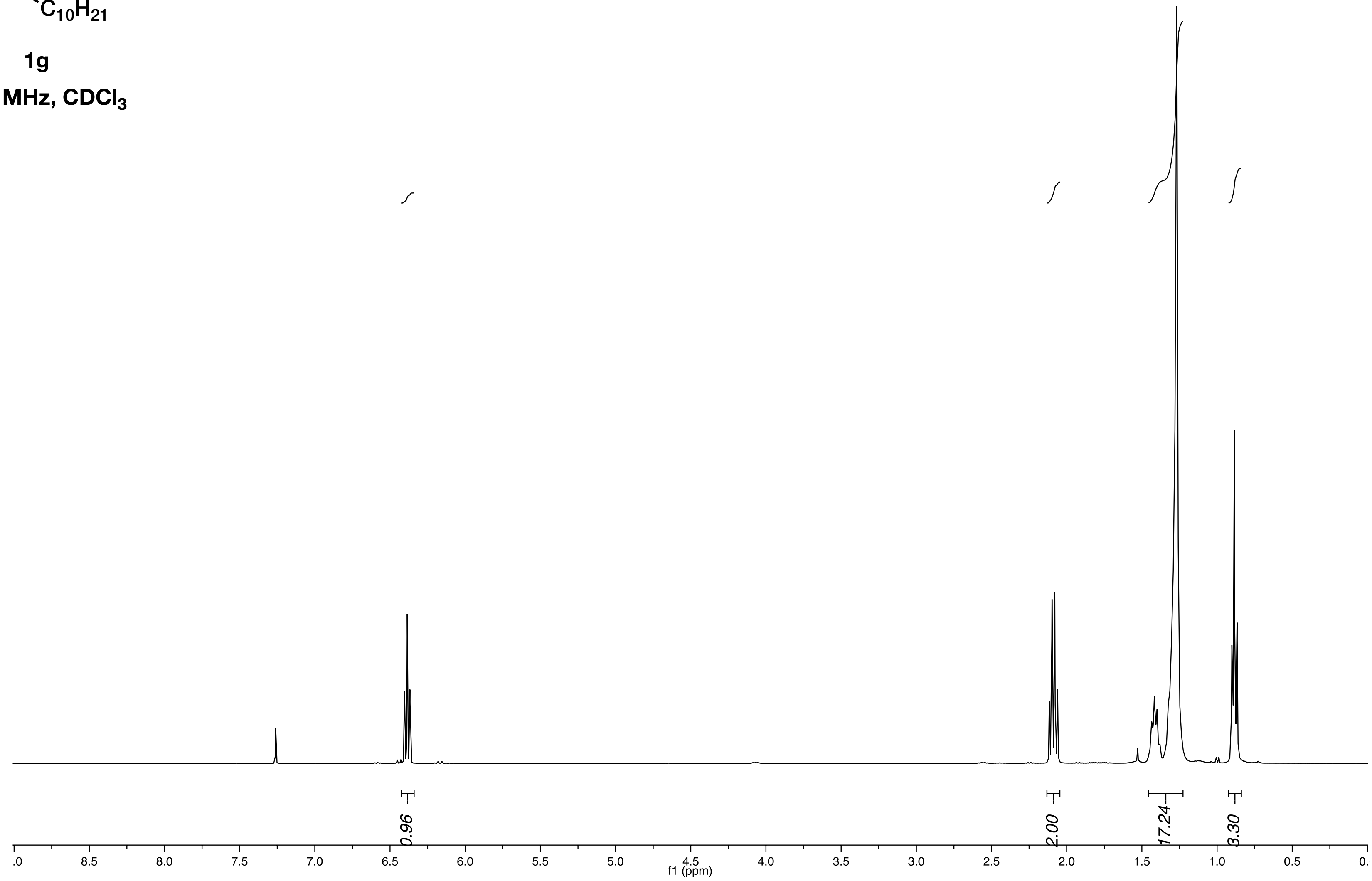

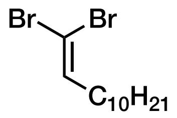

1g

100 MHz, $CDCl_3$

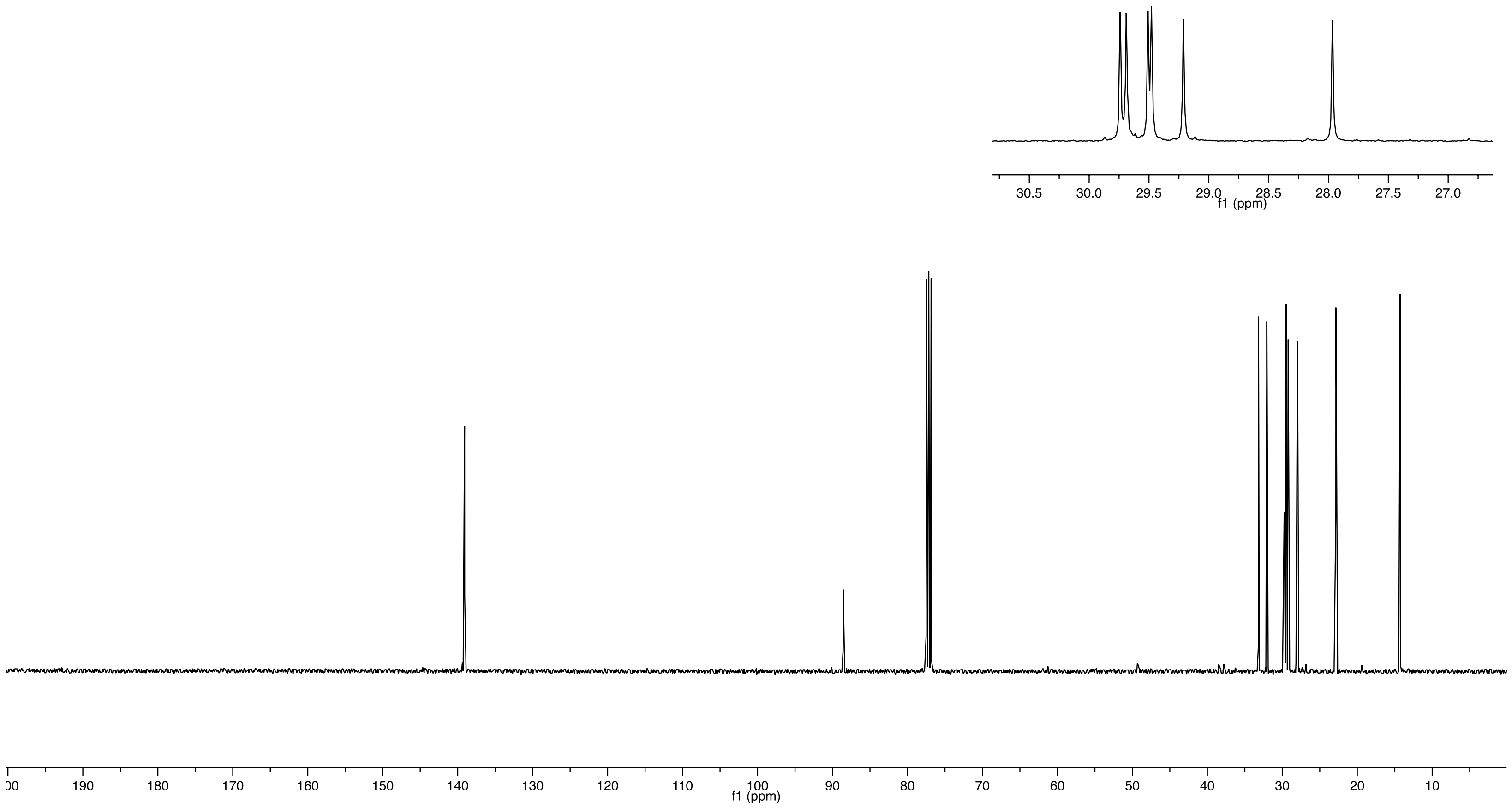

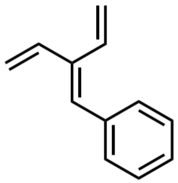

2a

400 MHz, $CDCl_3$

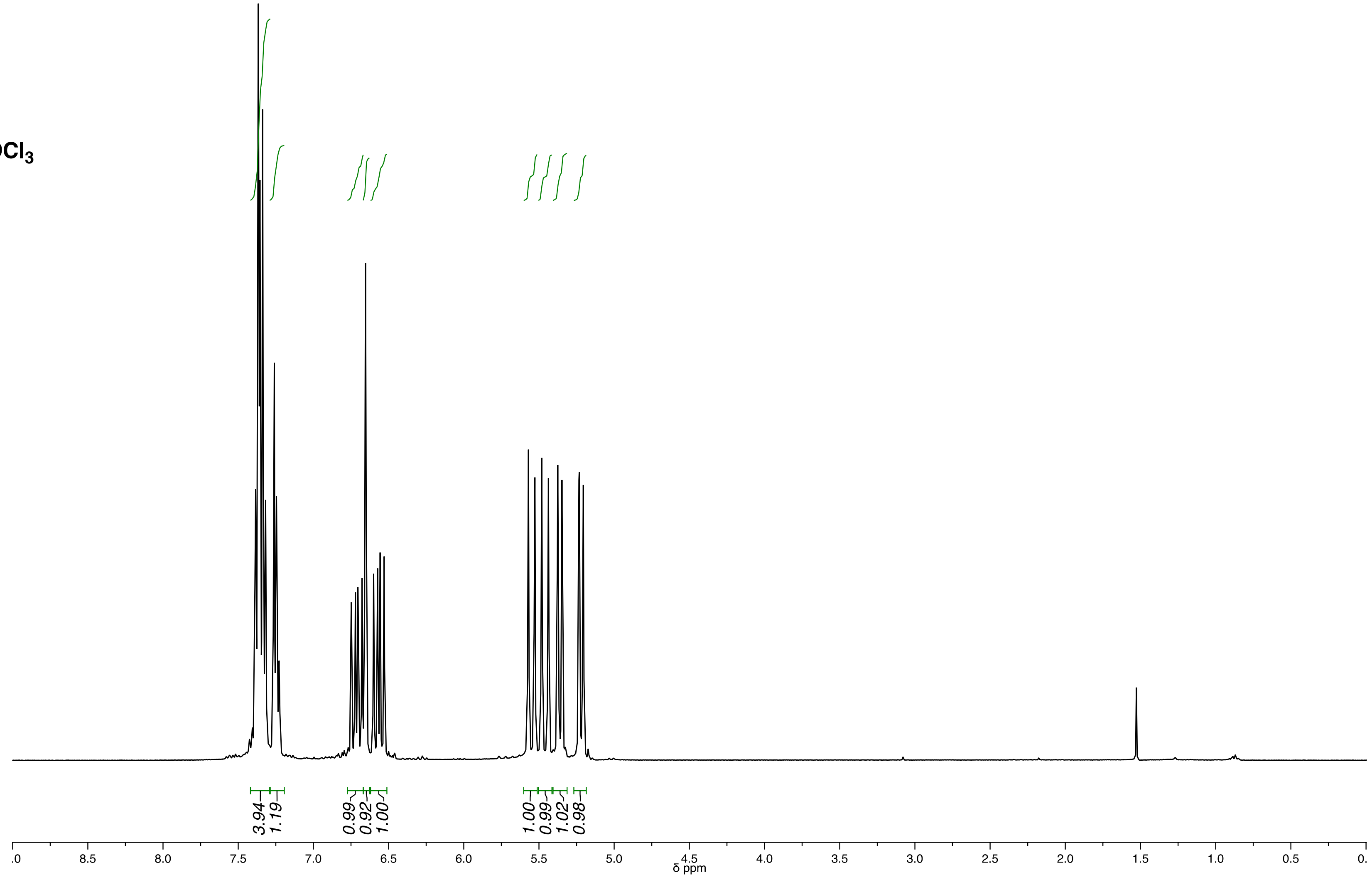

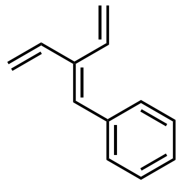

2a

100 MHz, $CDCl_3$

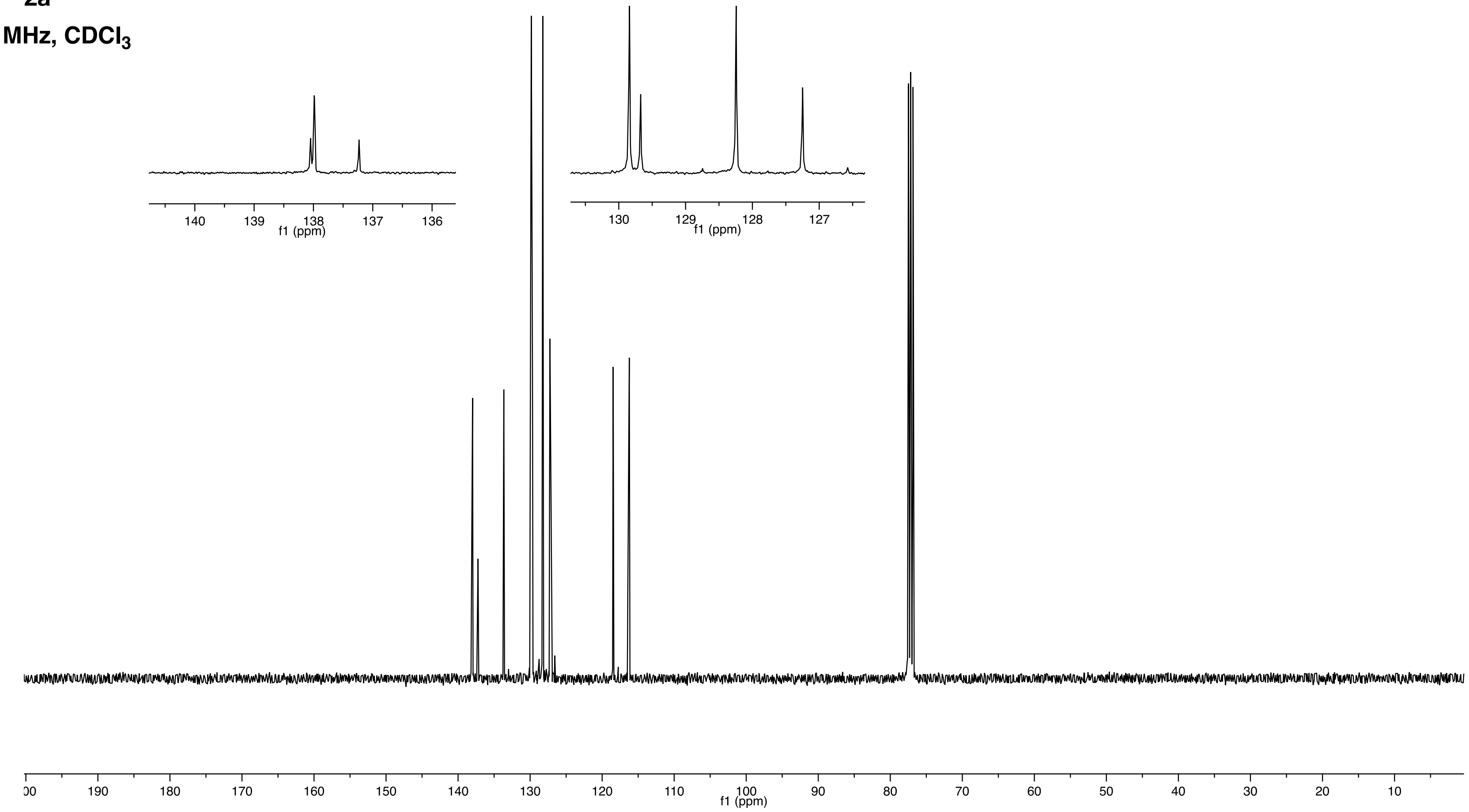

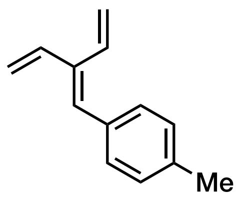

2b

400 MHz, $CDCl_3$

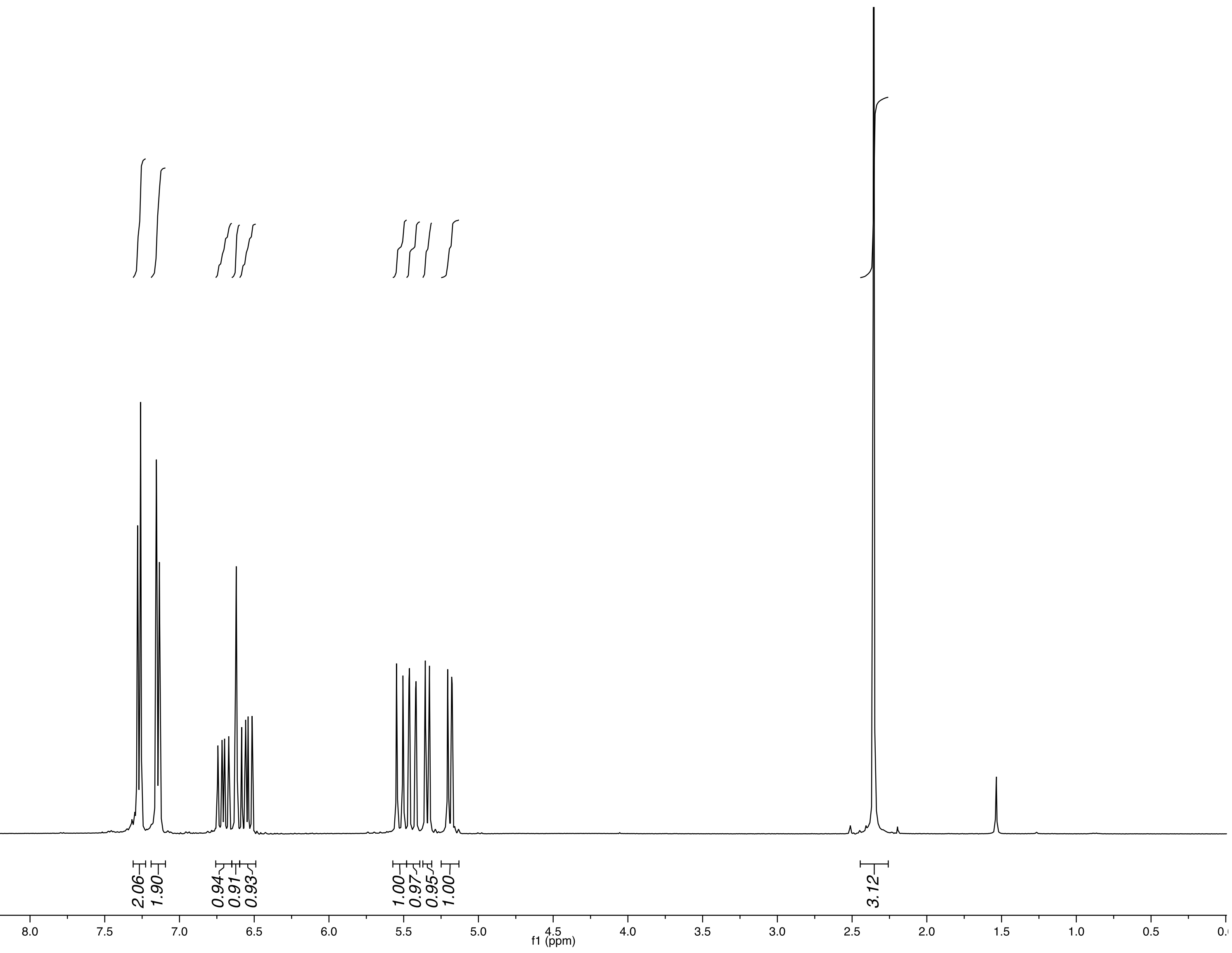

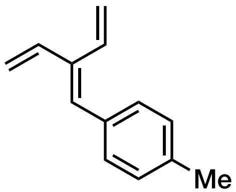

2b

100 MHz, $CDCl_3$

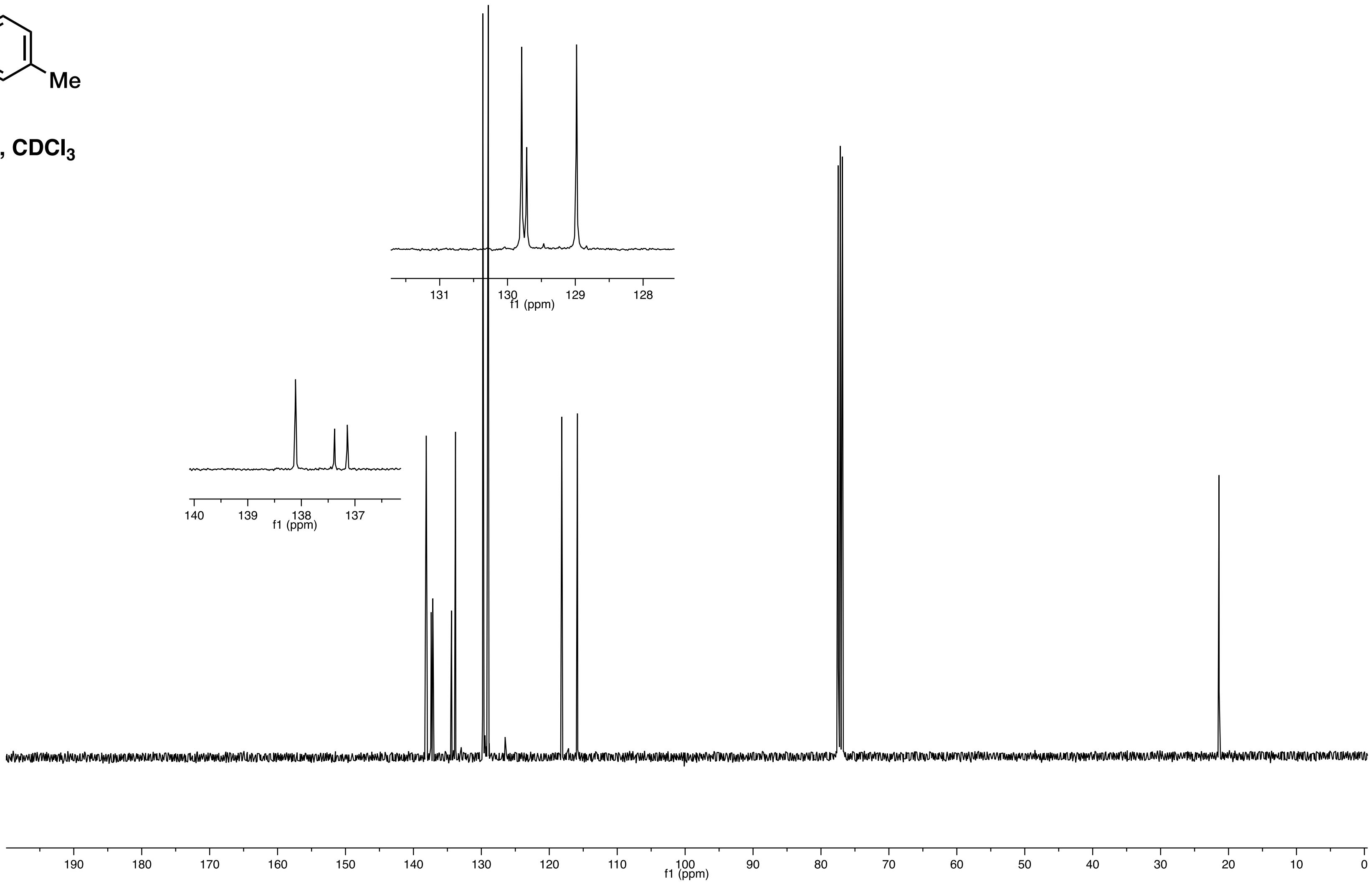

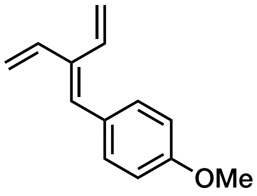

2c

400 MHz, $CDCl_3$

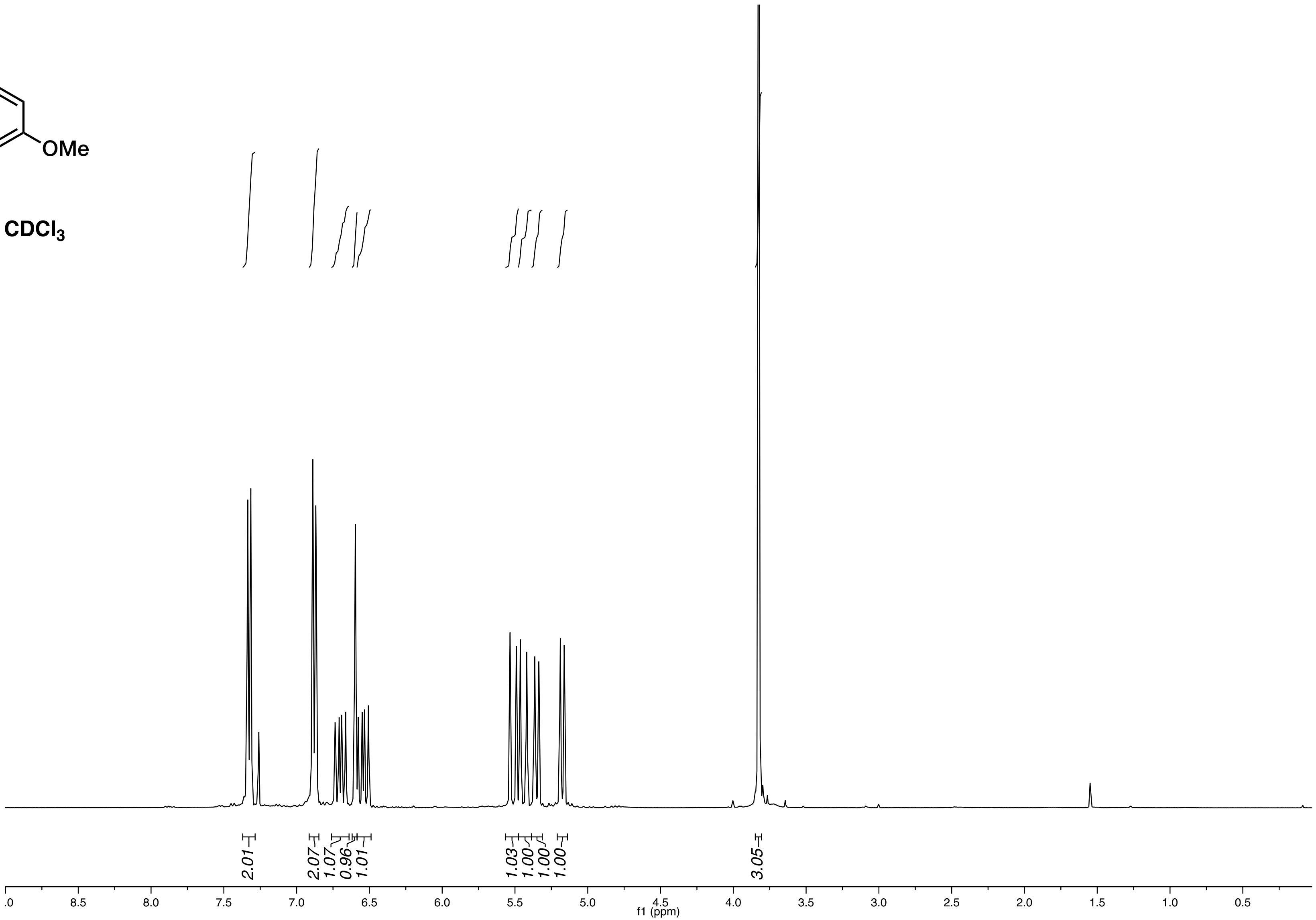

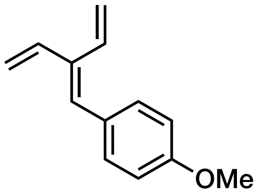

2c

100 MHz, $CDCl_3$

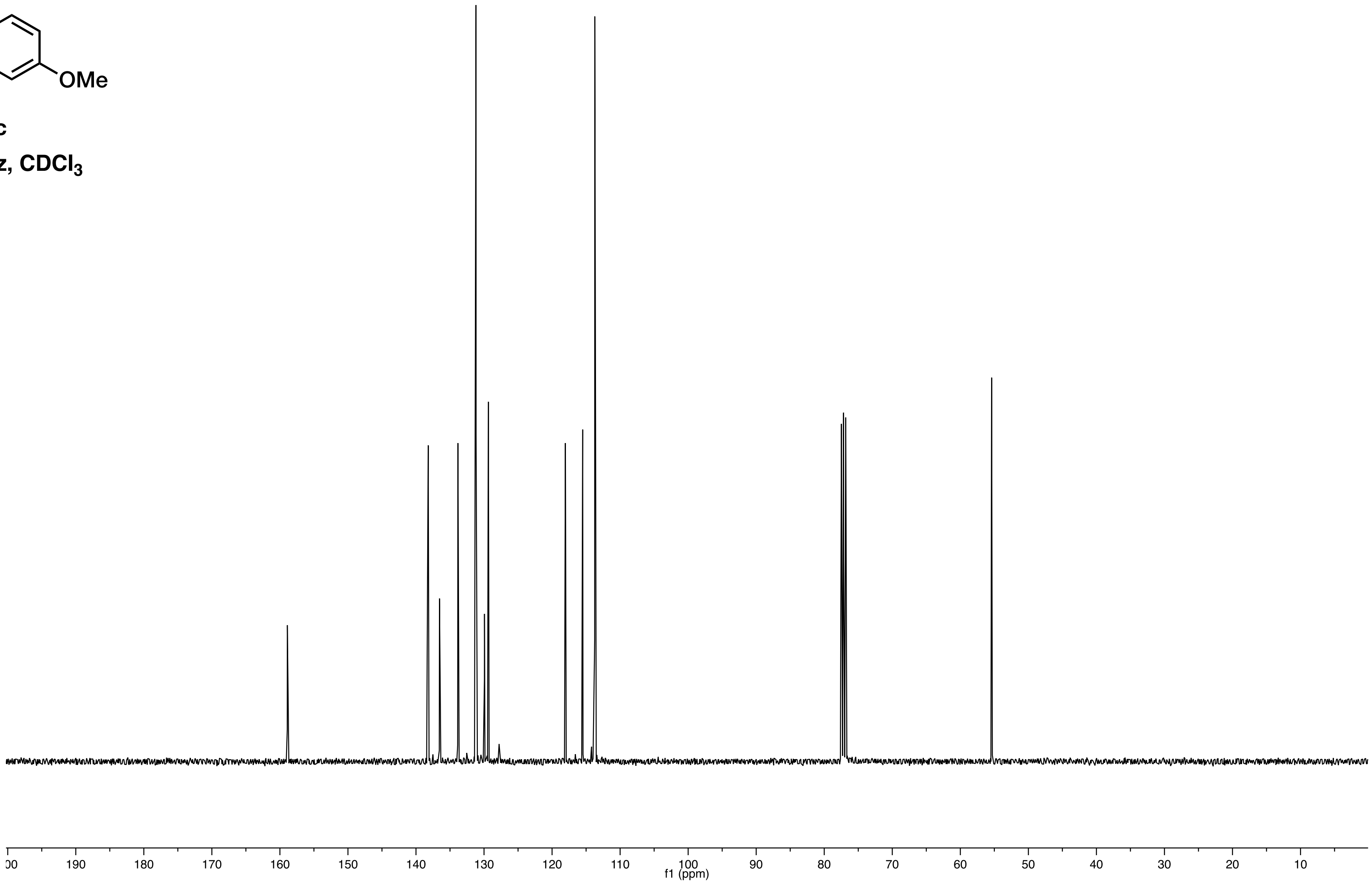

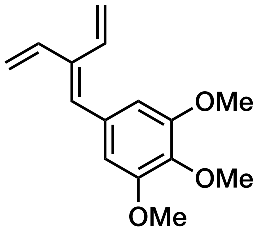

2d

400 MHz, $CDCl_3$

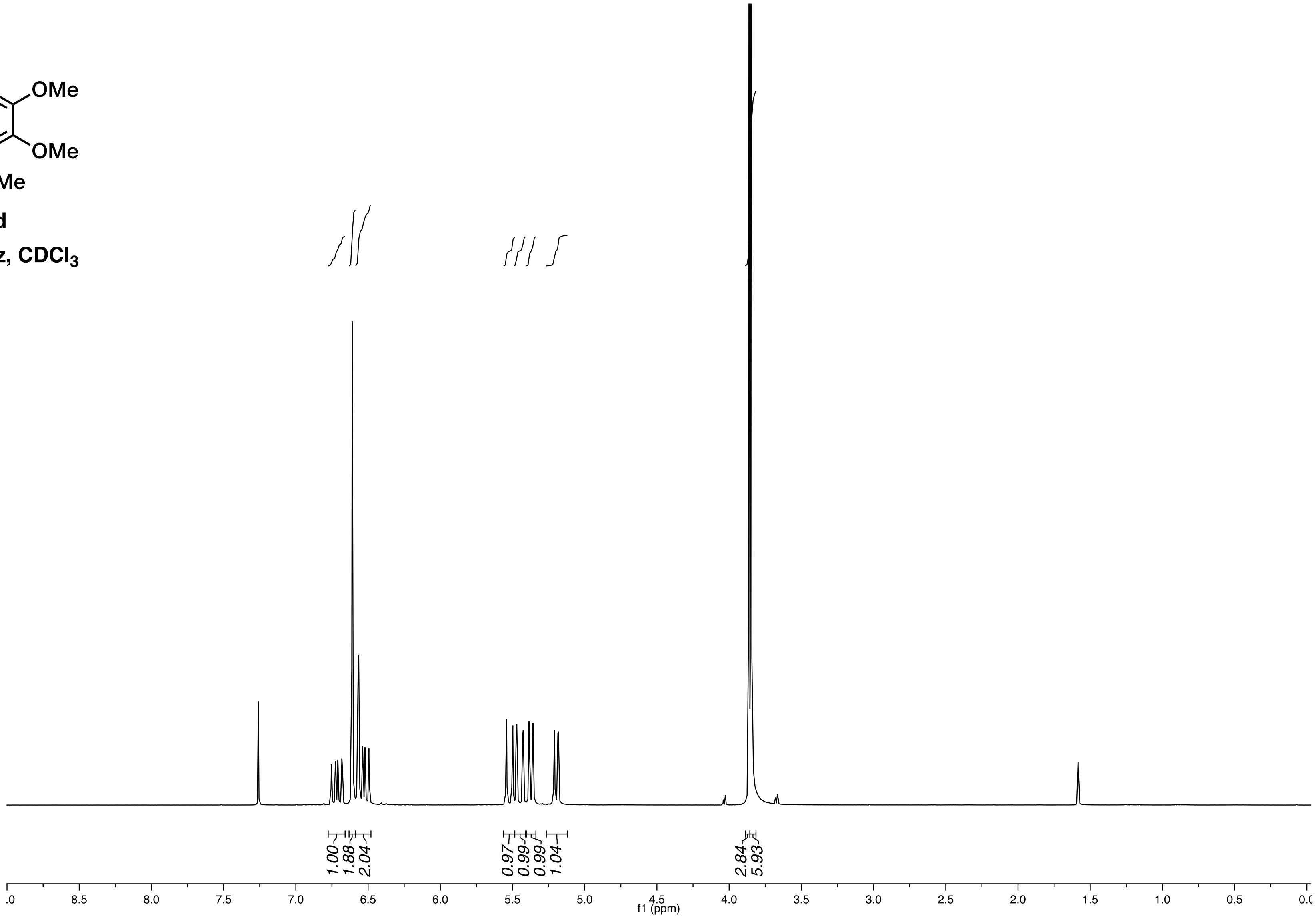

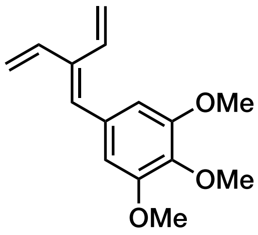

2d

100 MHz, CDCl$_3$

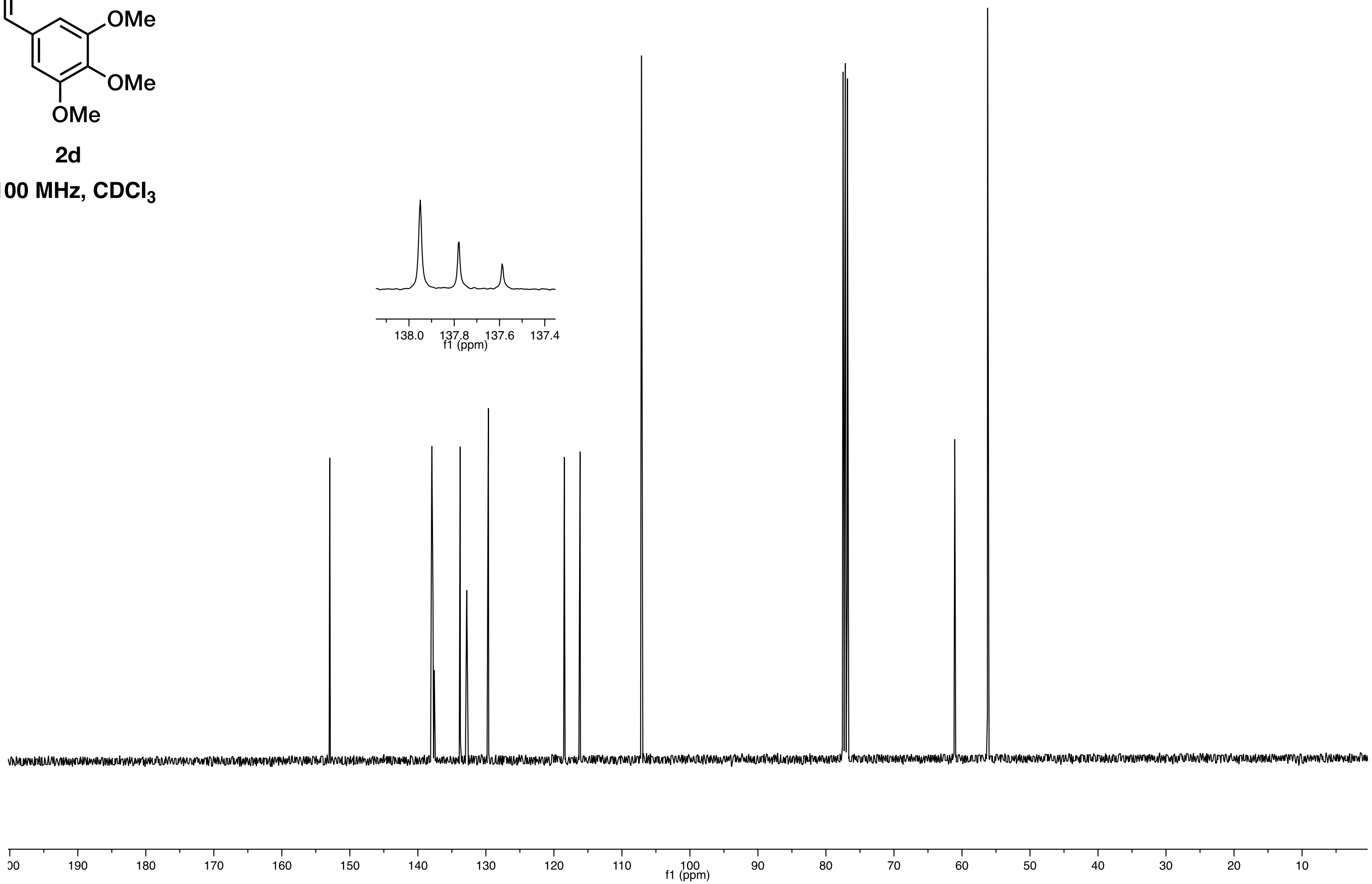

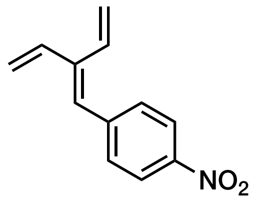

2e

400 MHz, C_6D_6

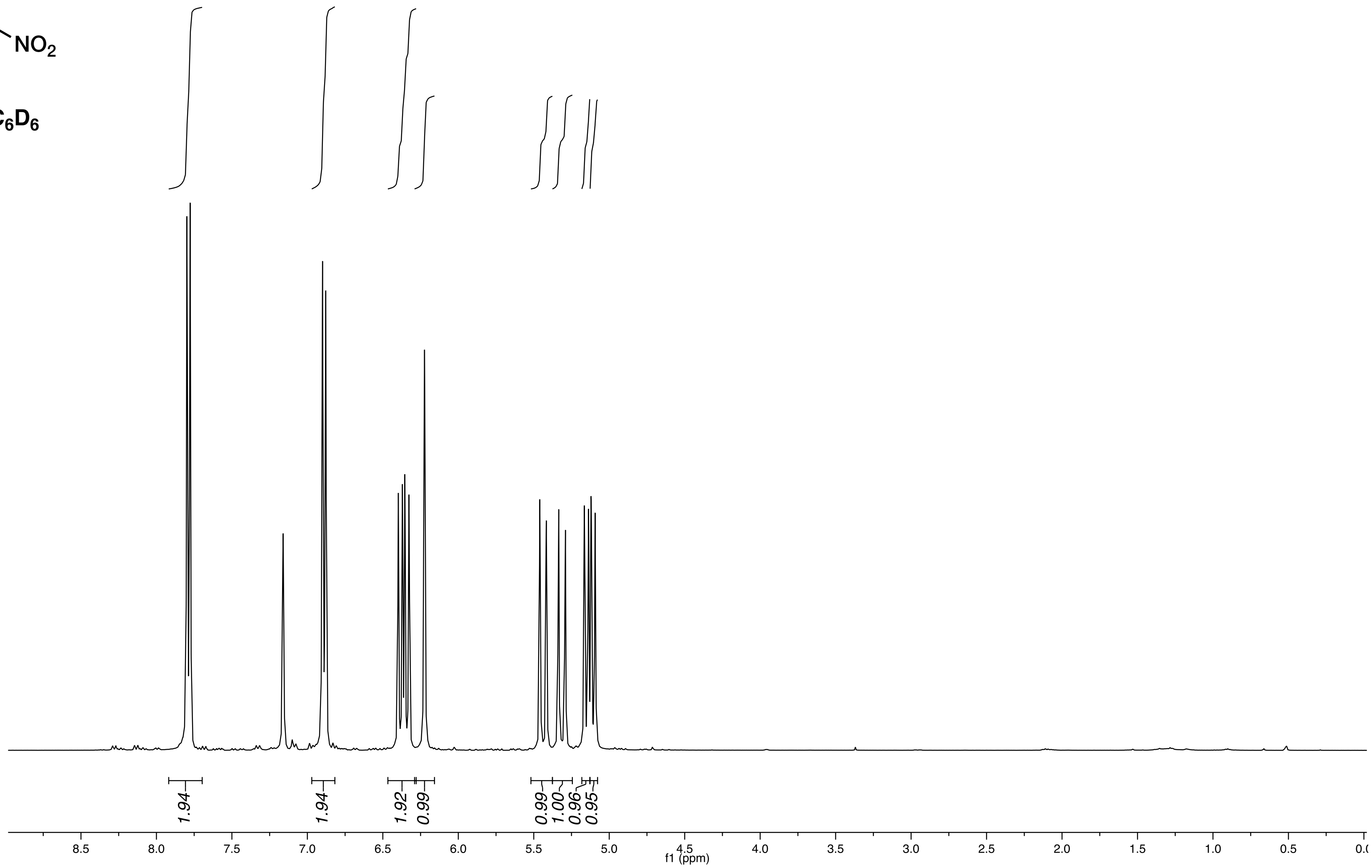

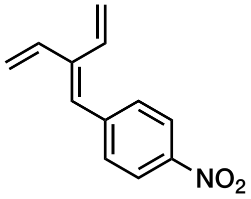

2e

100 MHz, C_6D_6

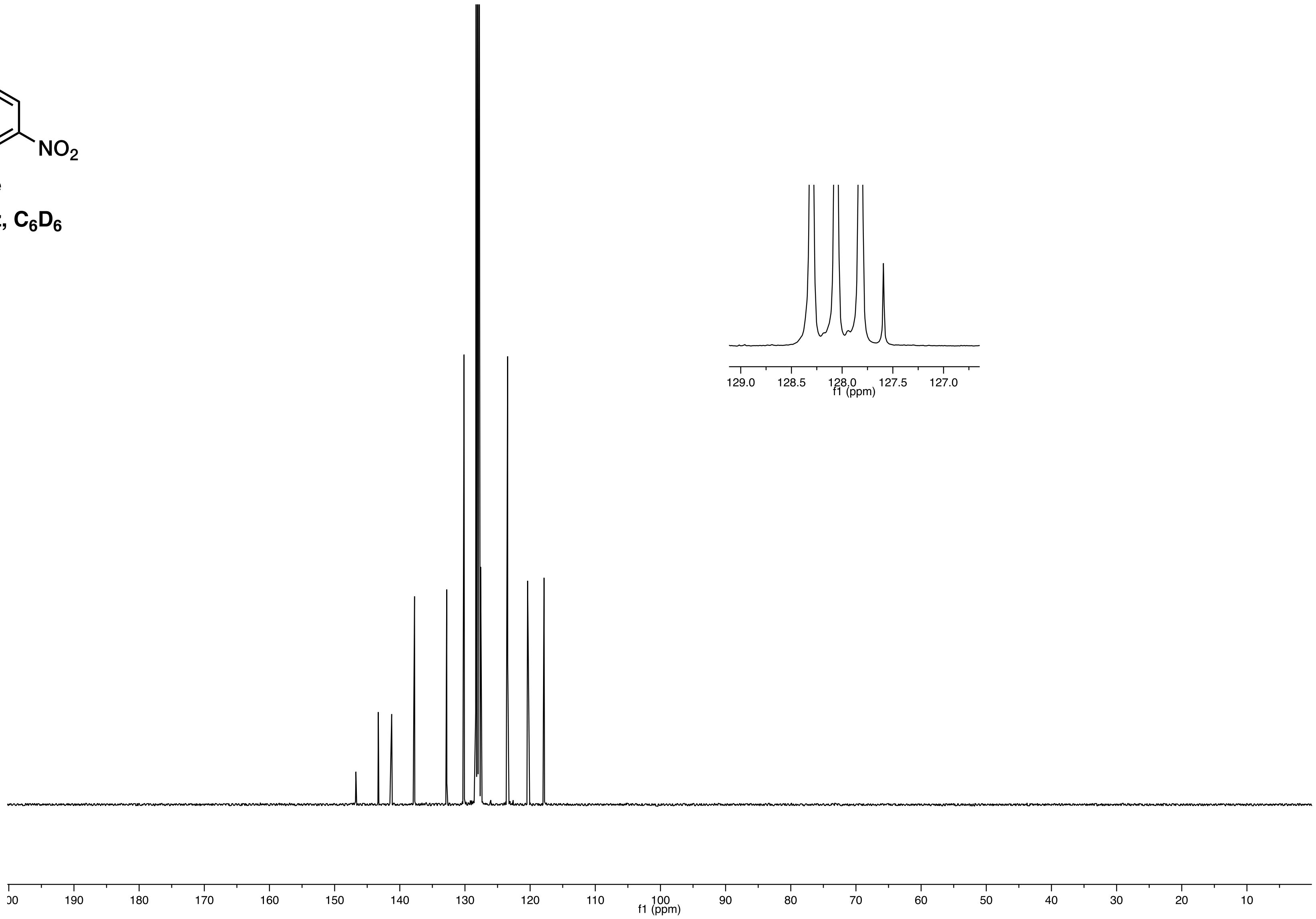

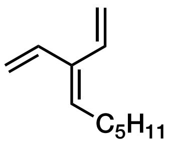

2f

400 MHz, $CDCl_3$

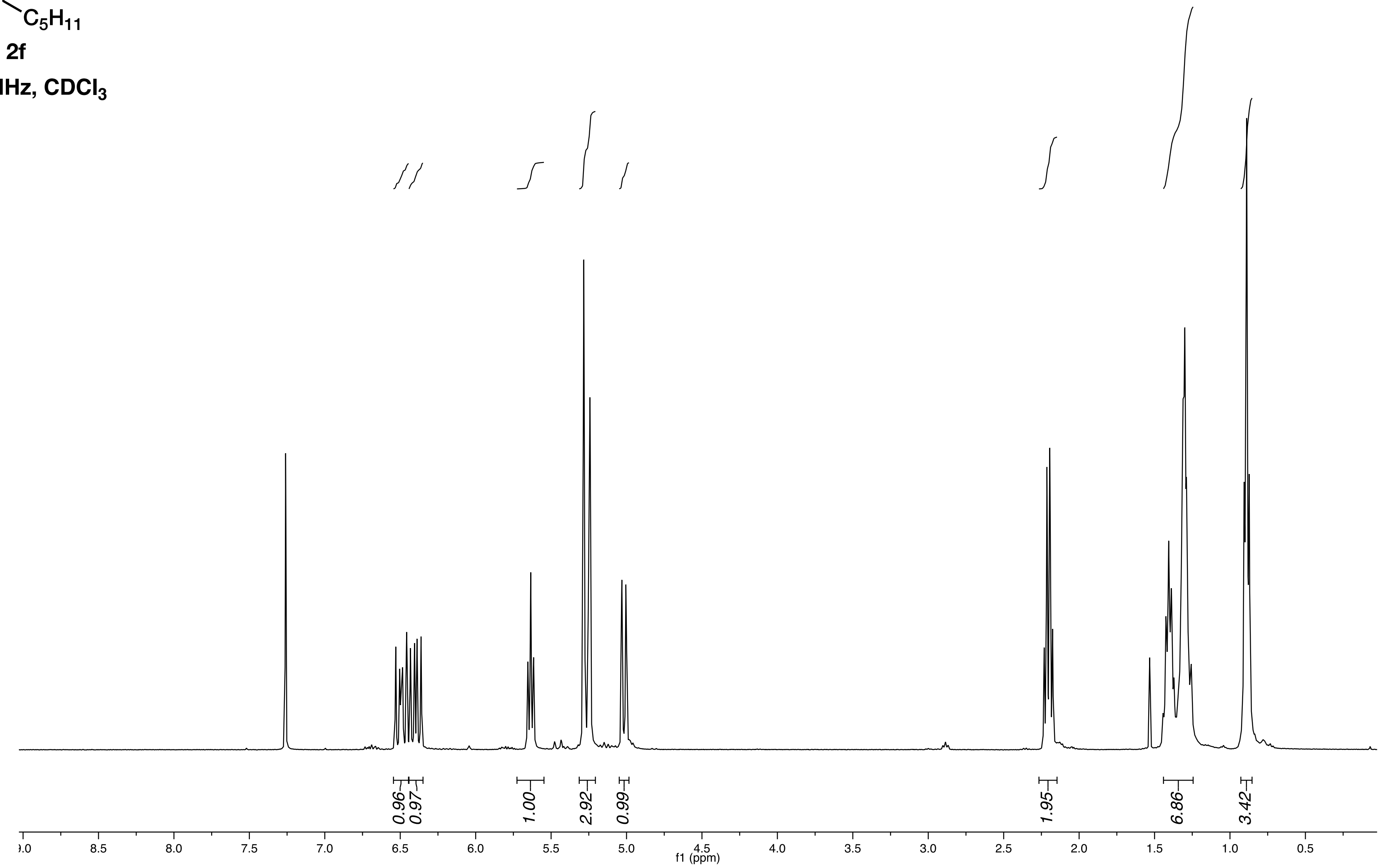

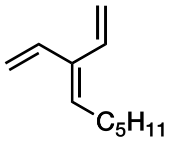

2f

100 MHz, $CDCl_3$

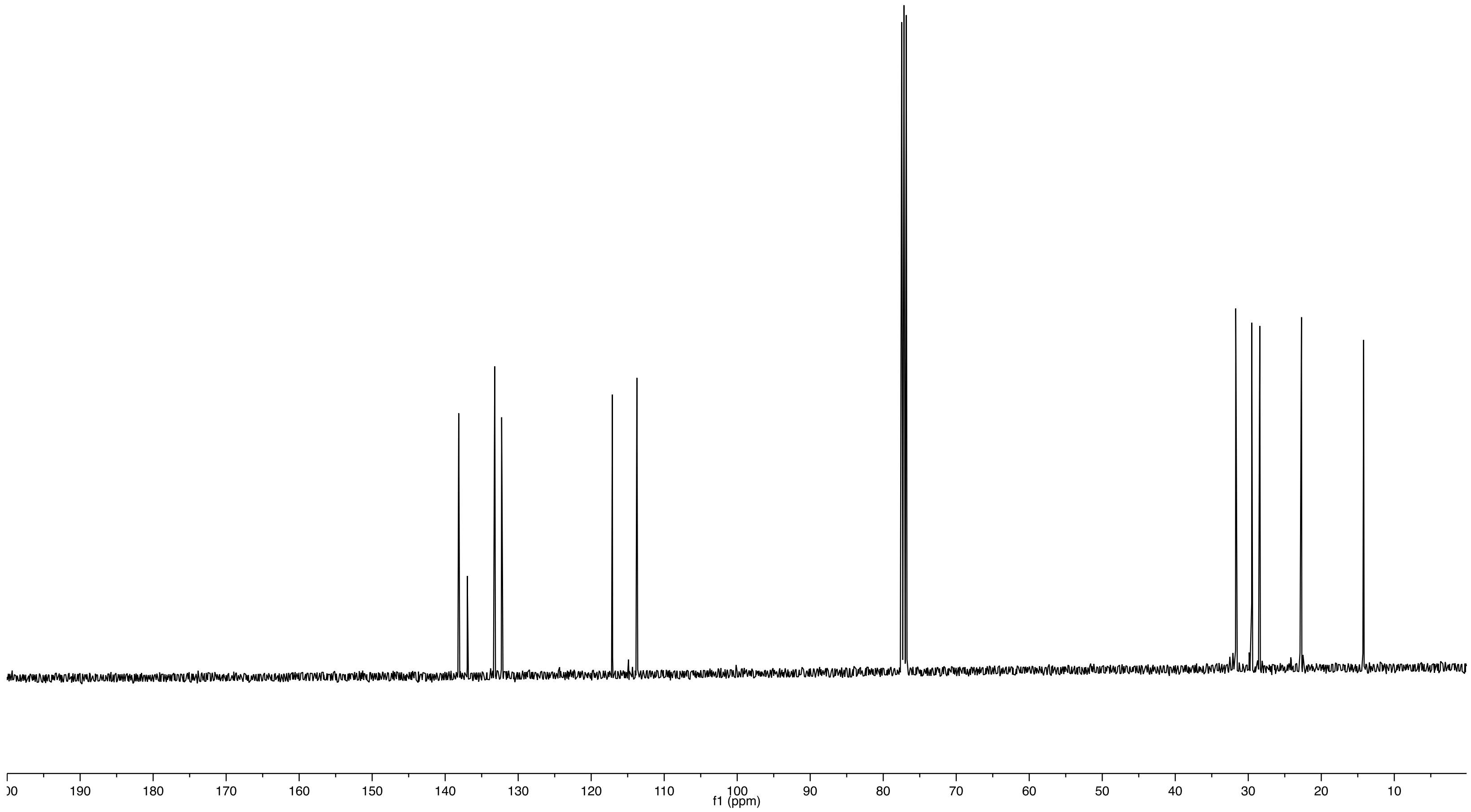

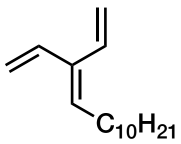

2g

400 MHz, $CDCl_3$

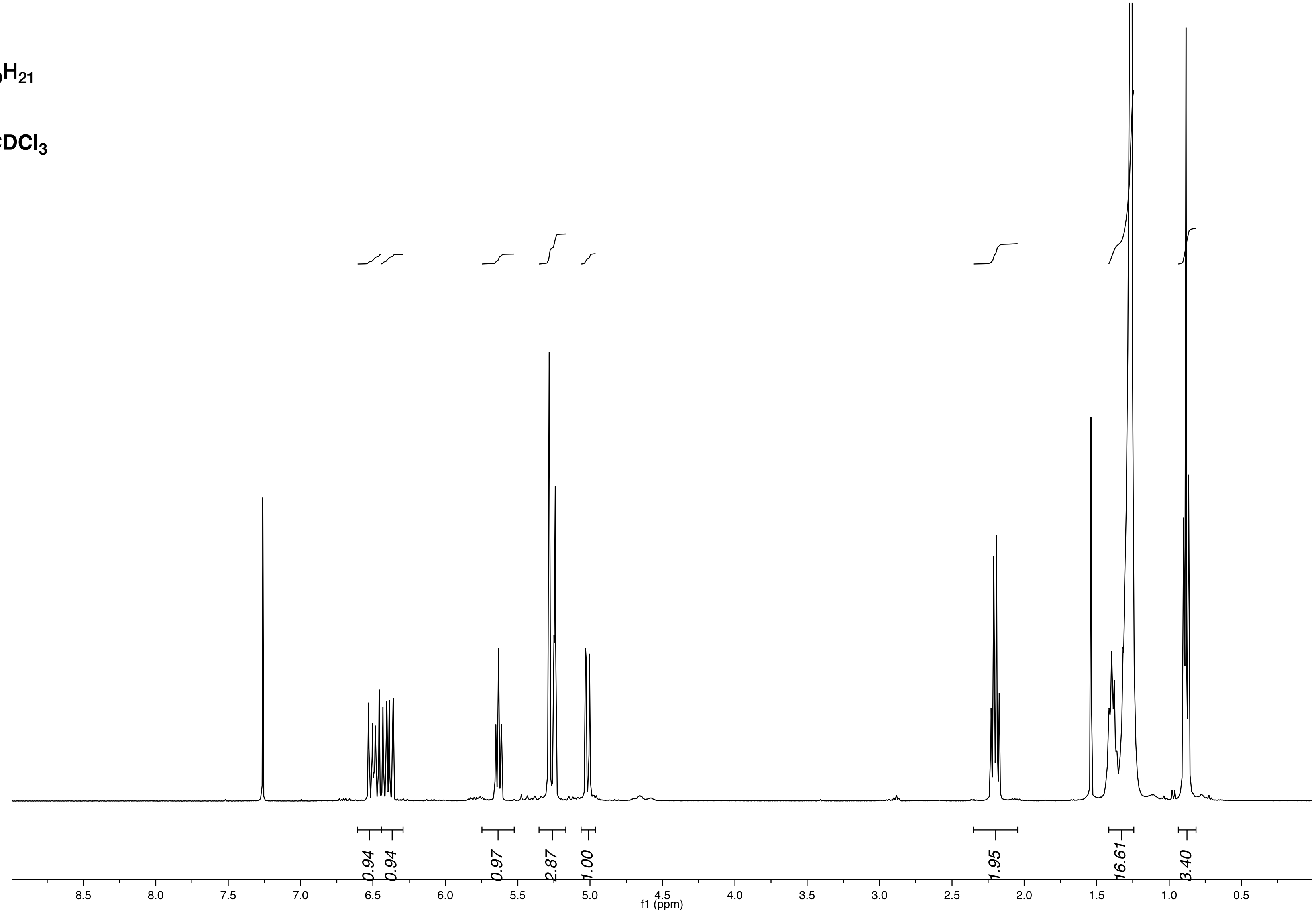

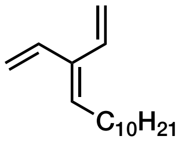

2g

100 MHz, $CDCl_3$

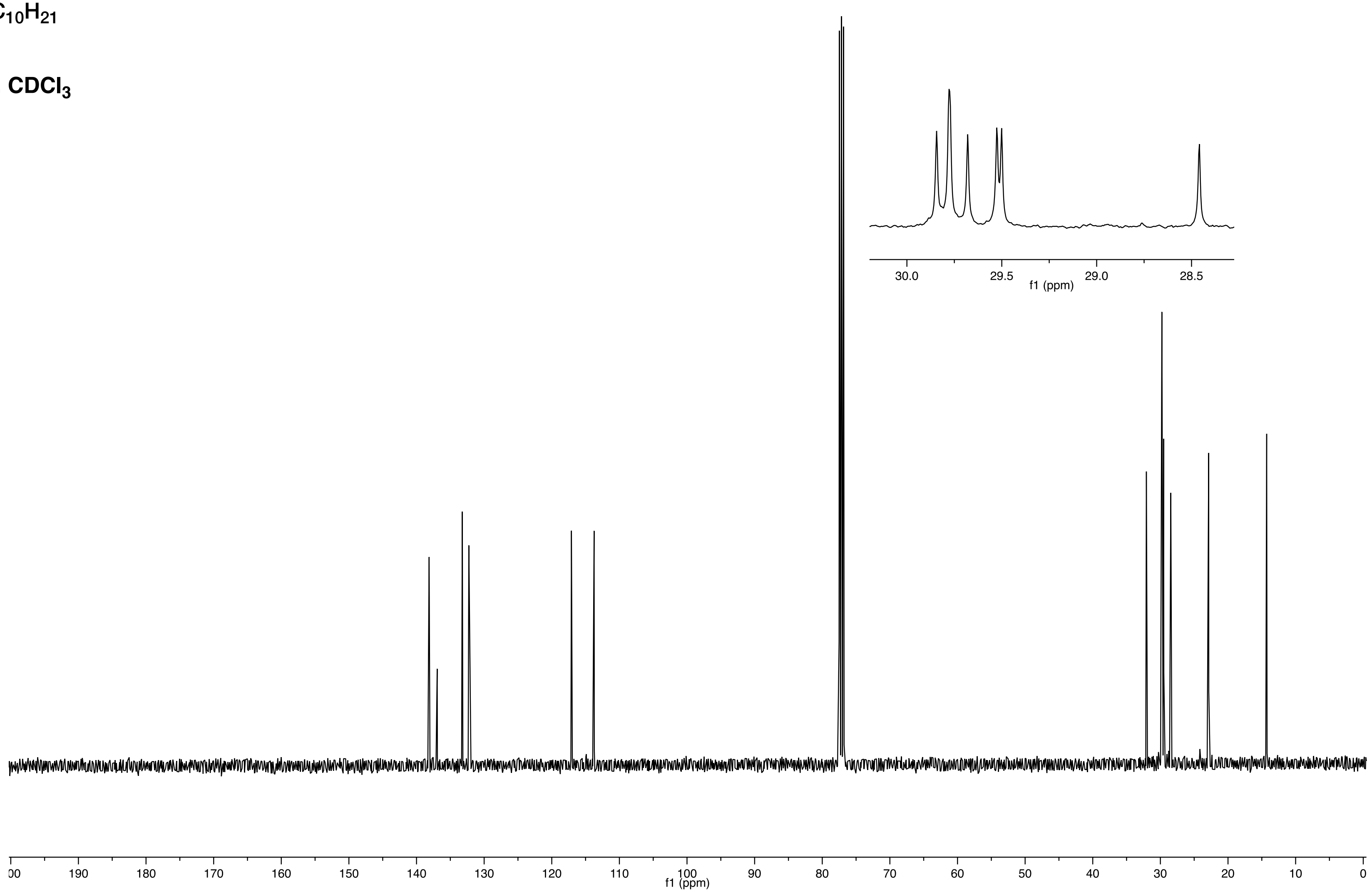

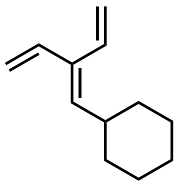

2h

400 MHz, $CDCl_3$

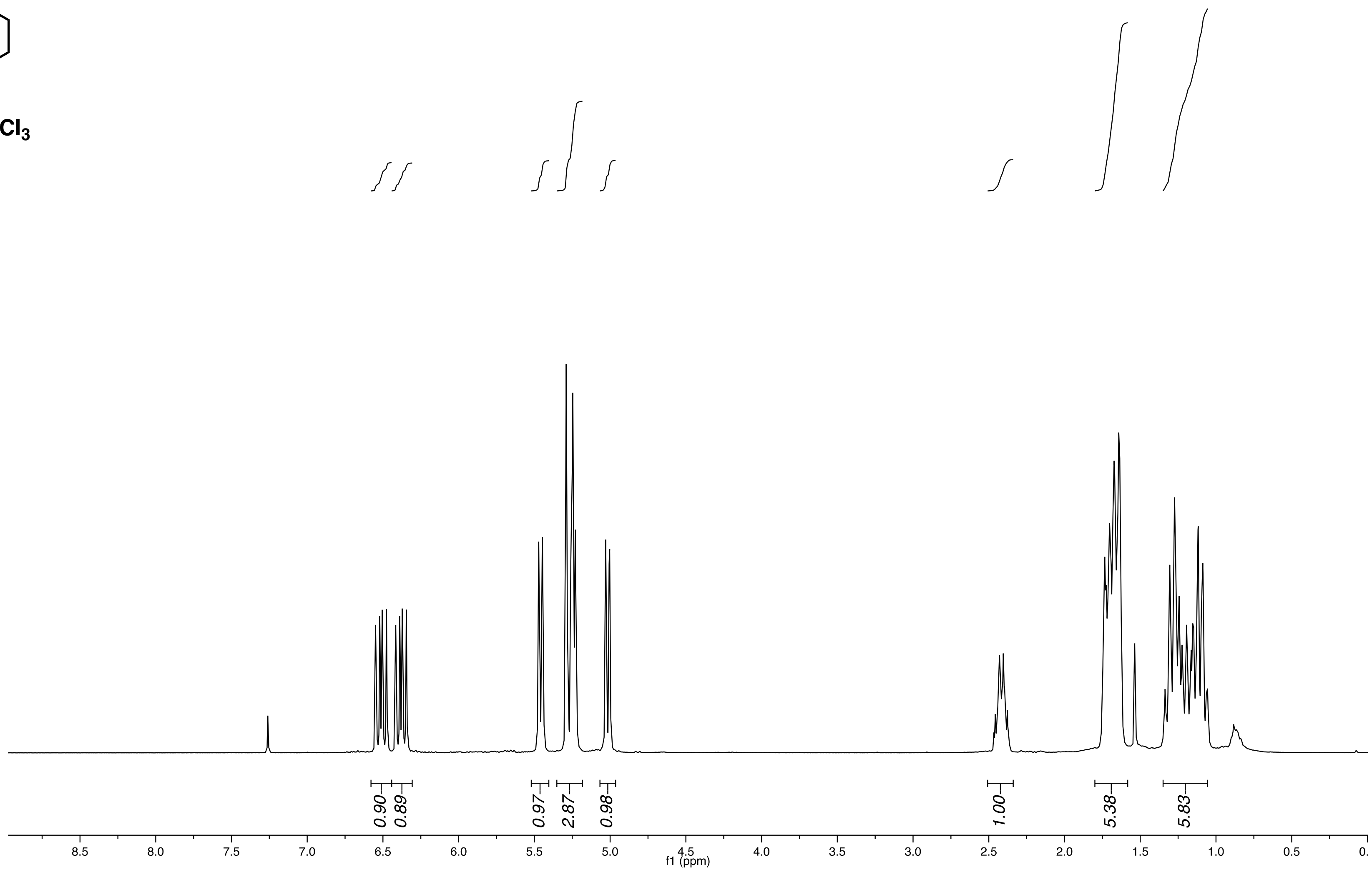

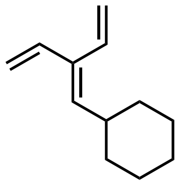

2h

100 MHz, $CDCl_3$

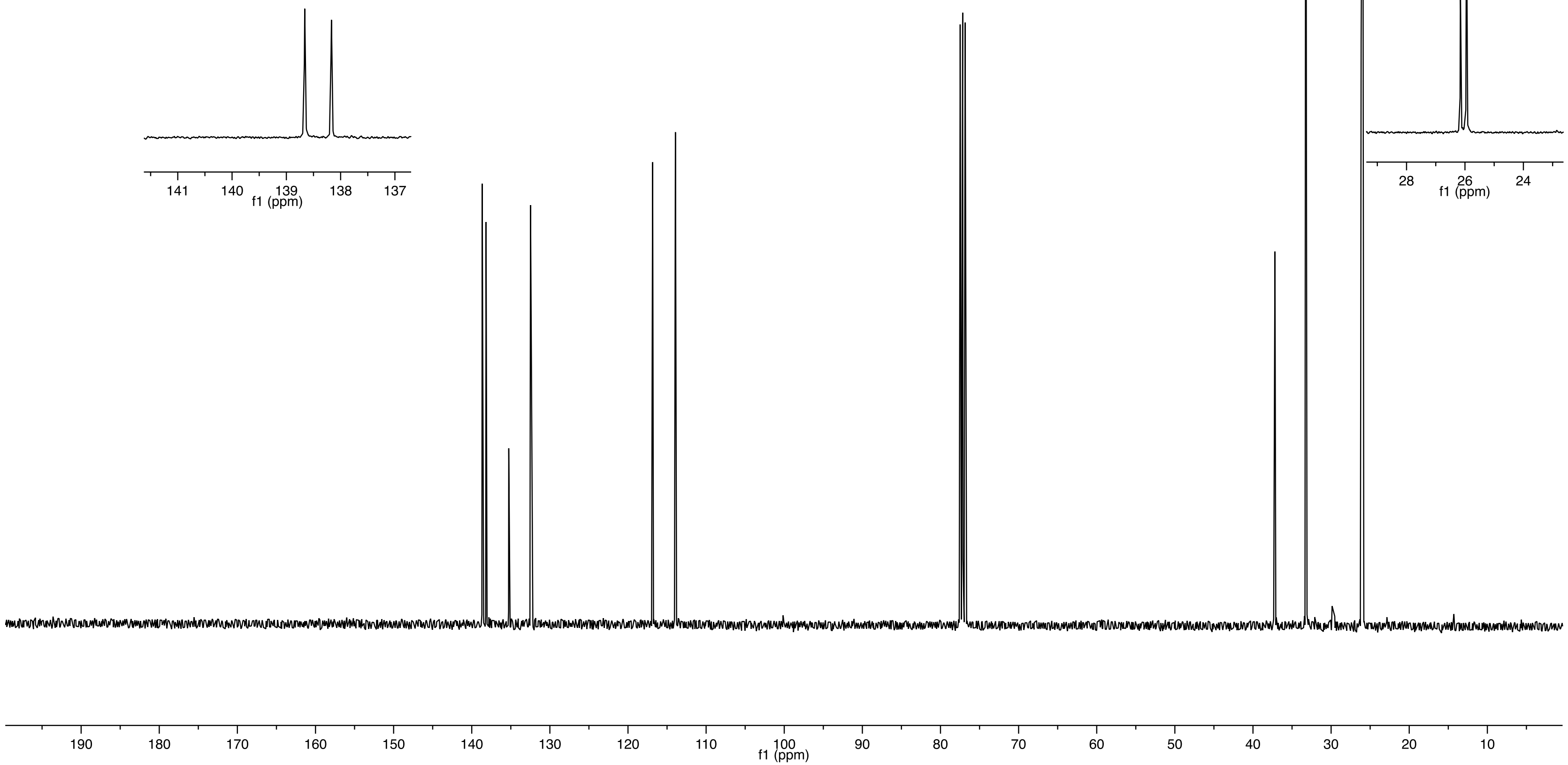

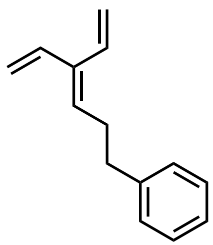

2i

400 MHz, $CDCl_3$

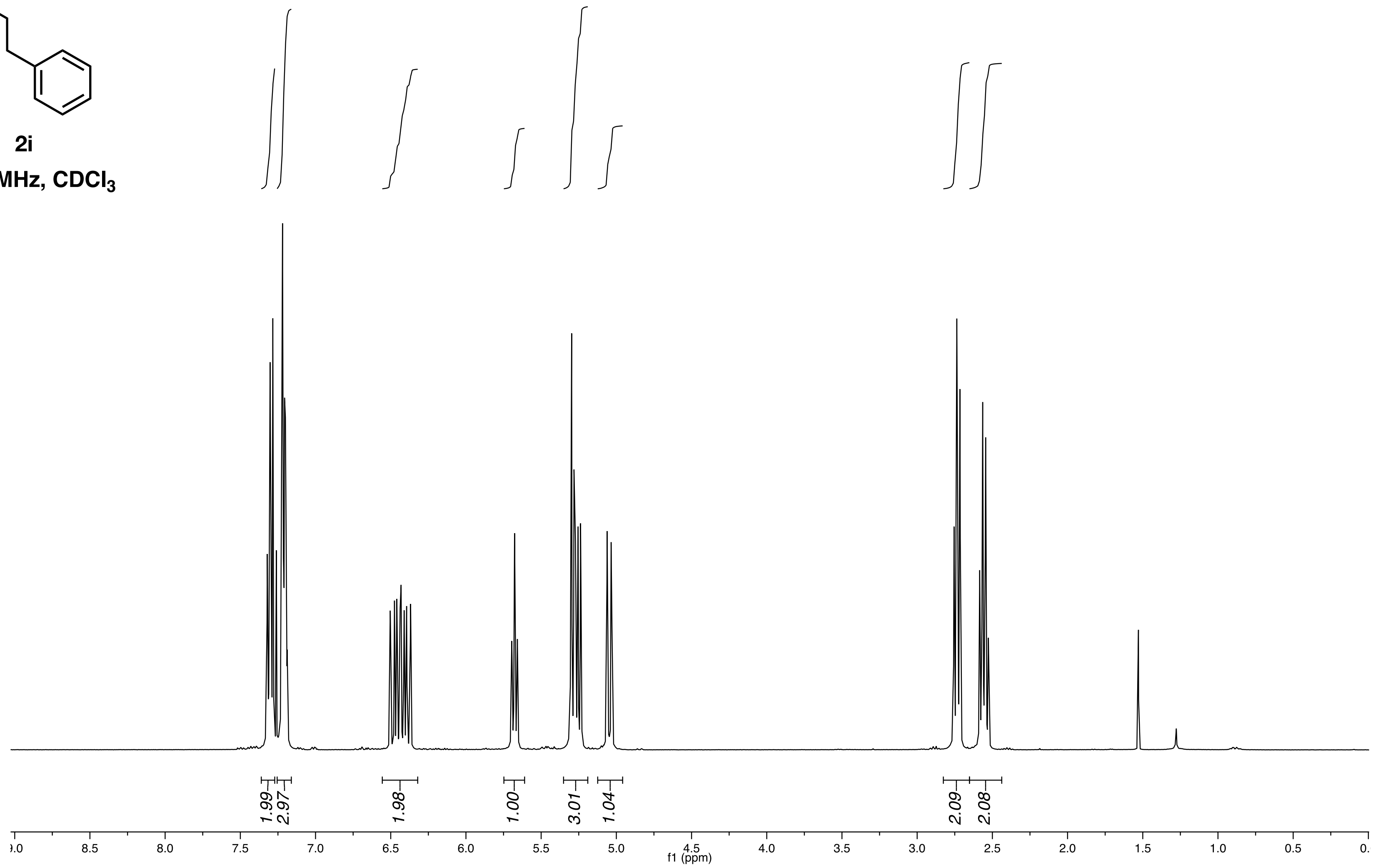

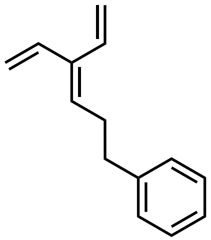

2i

100 MHz, $CDCl_3$

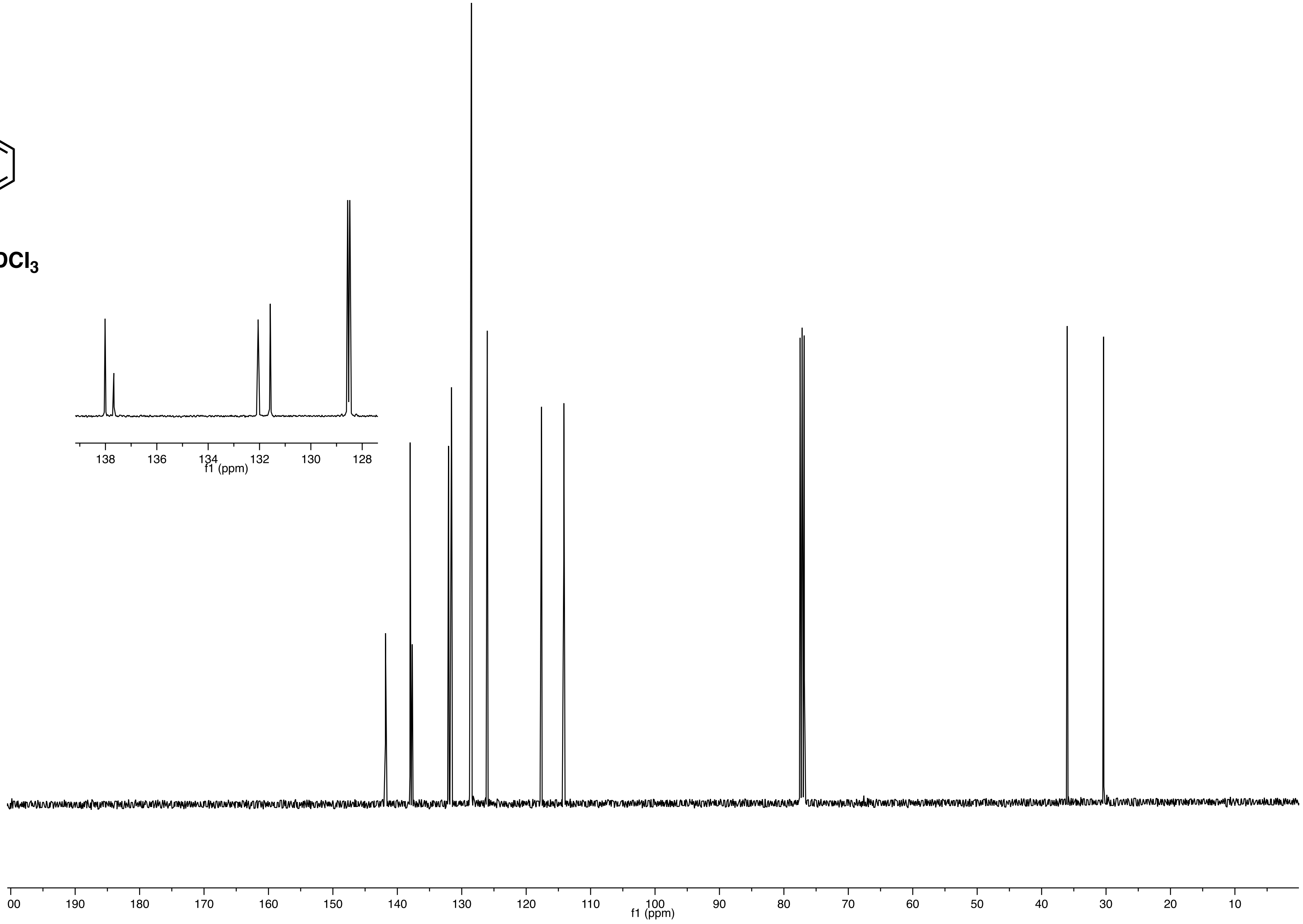

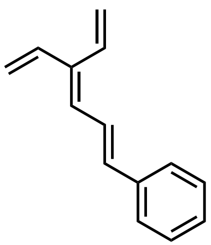

2j

400 MHz, $CDCl_3$

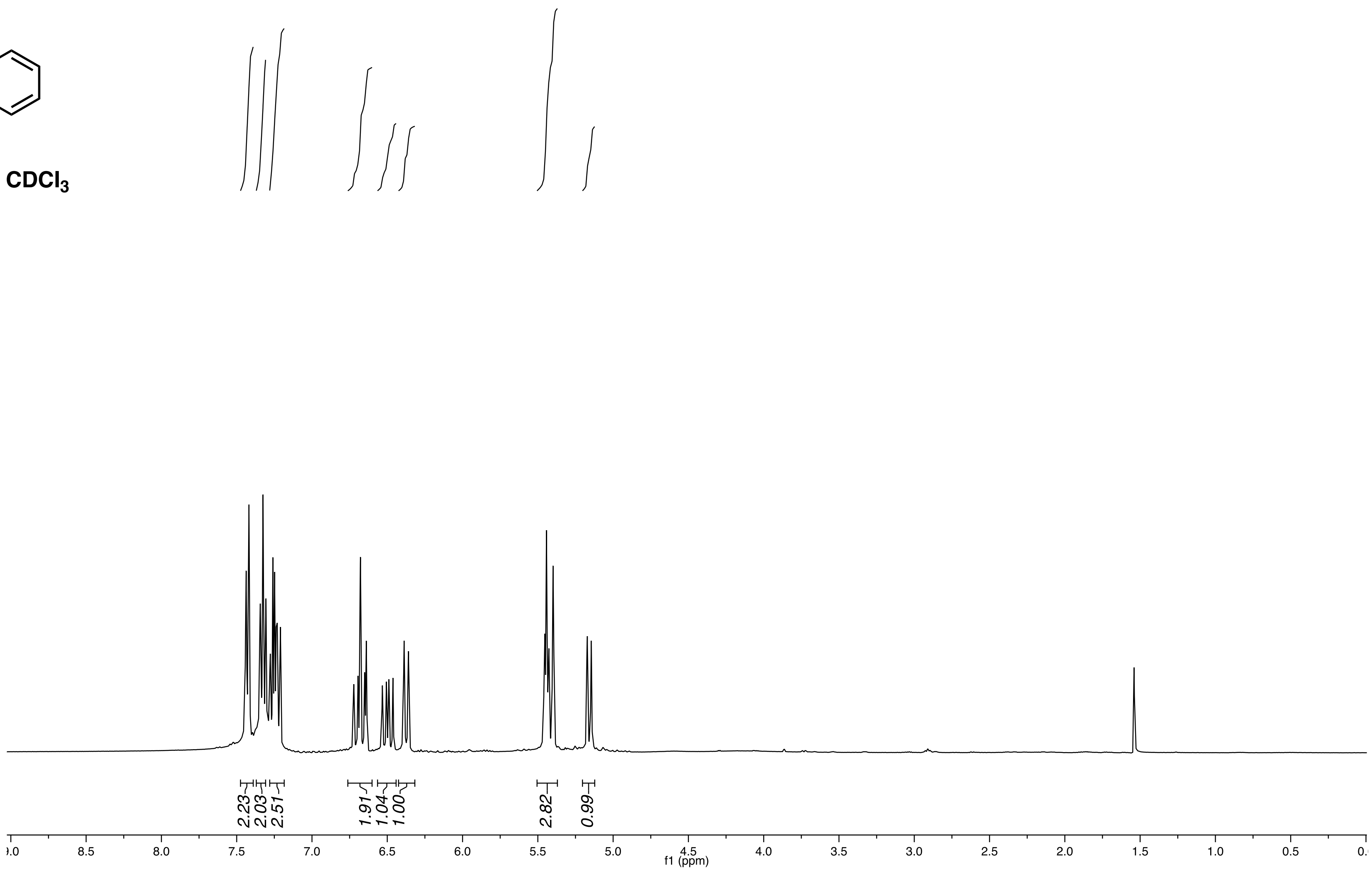

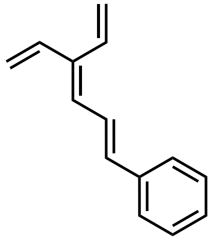

2j

100 MHz, $CDCl_3$

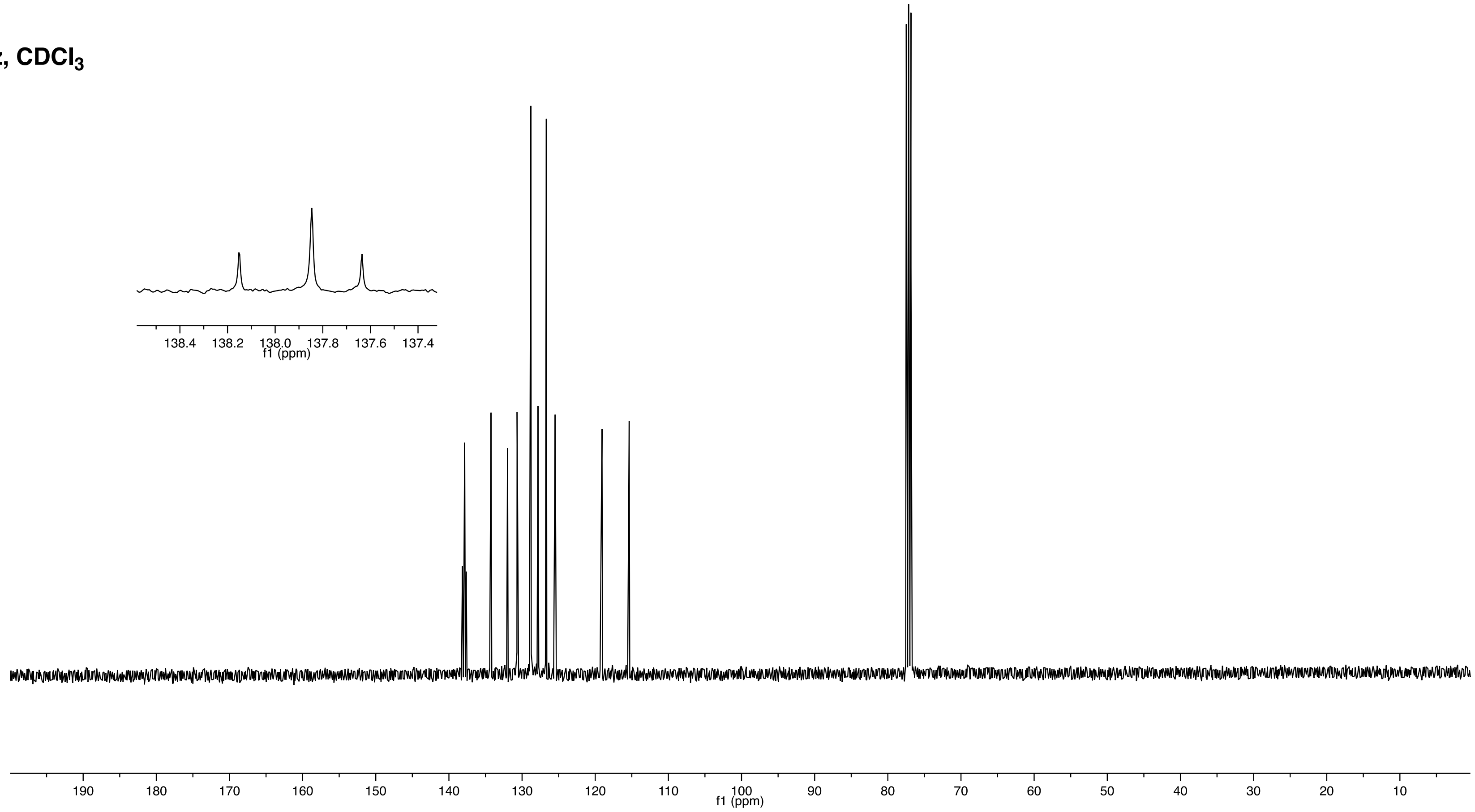

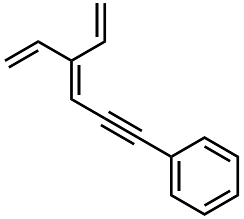

2k

400 MHz, $CDCl_3$

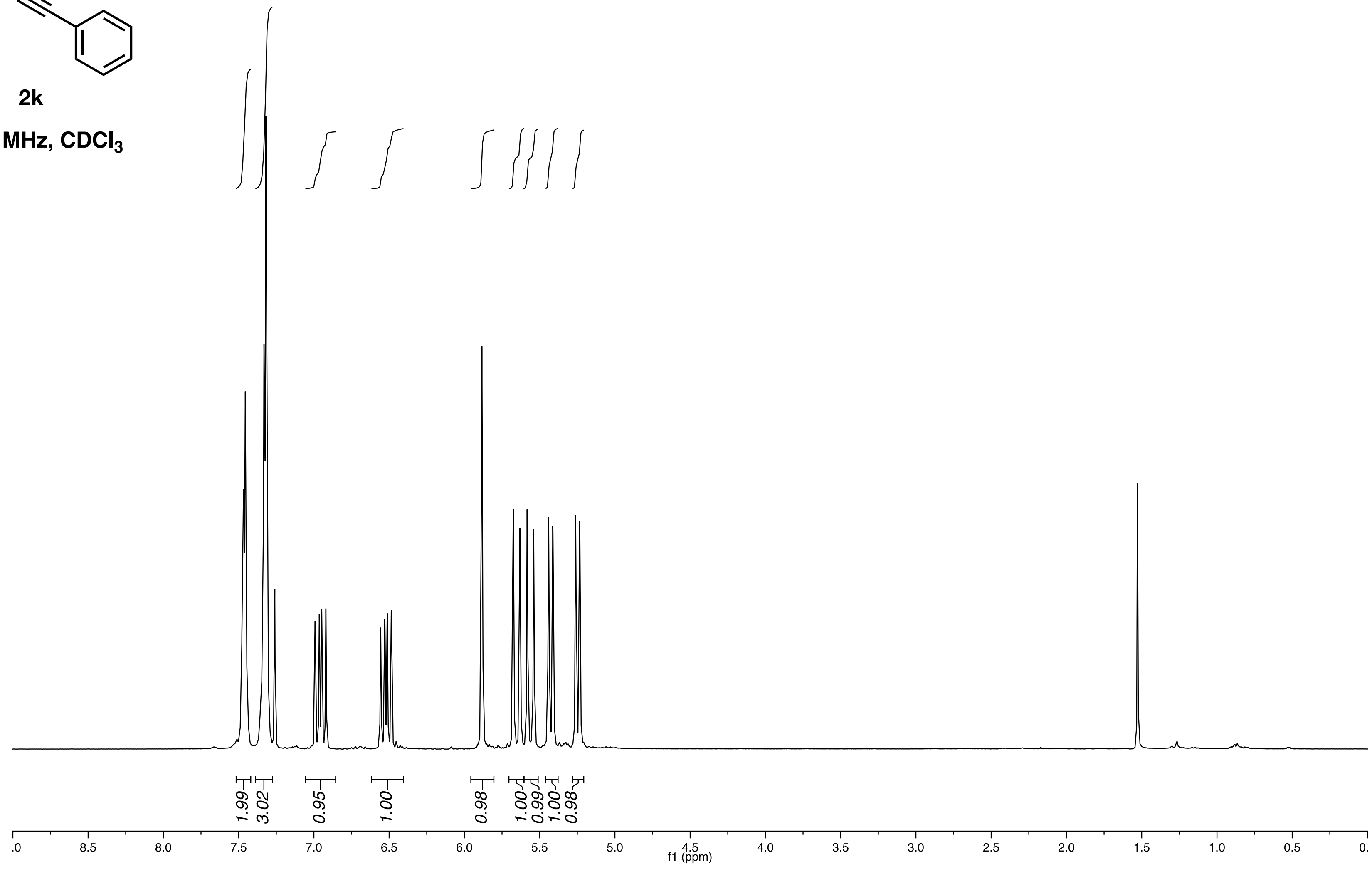

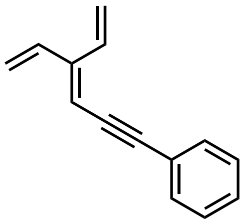

2k

100 MHz, $CDCl_3$

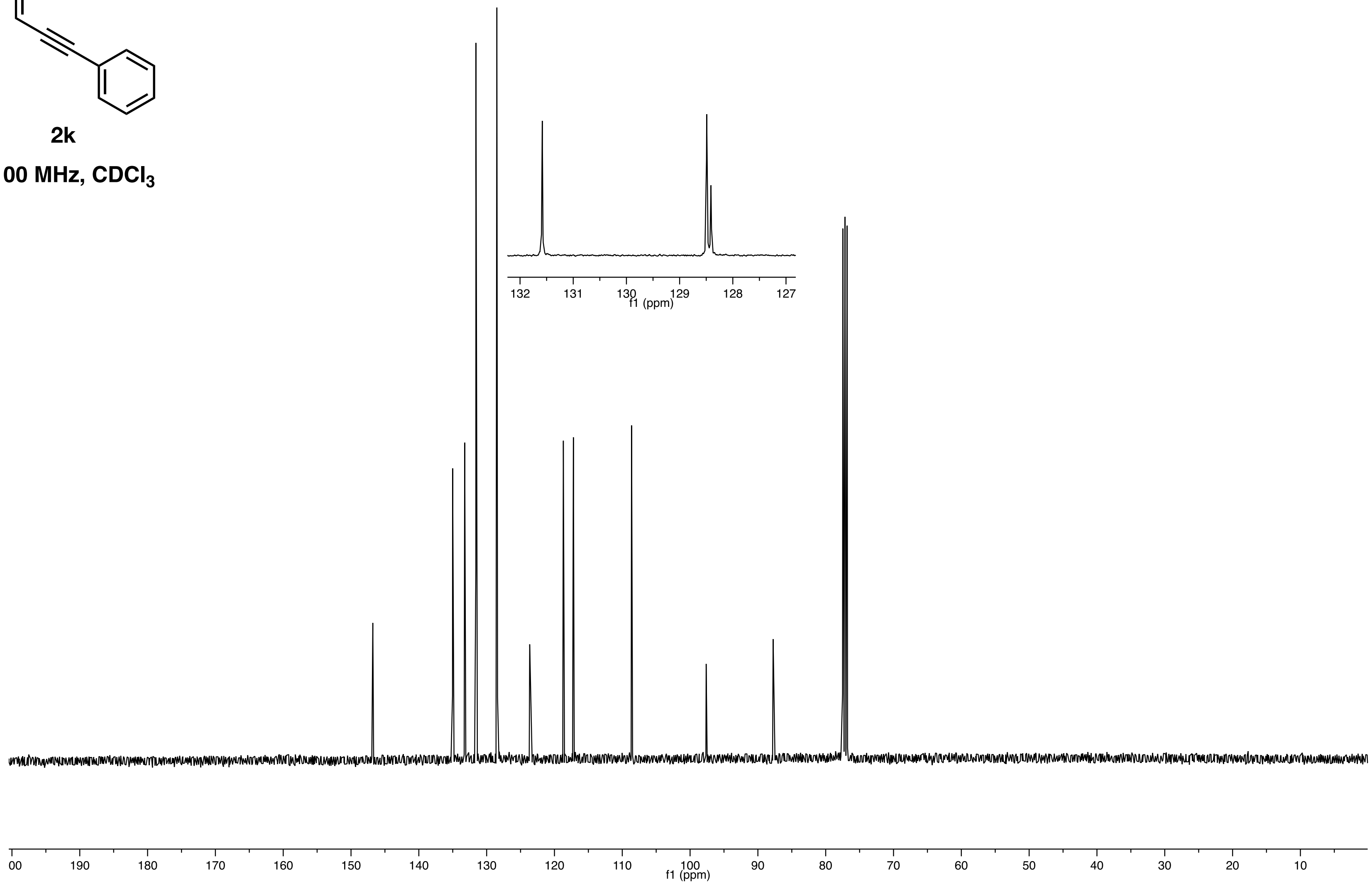

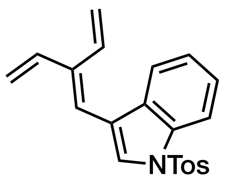

2l

400 MHz, $CDCl_3$

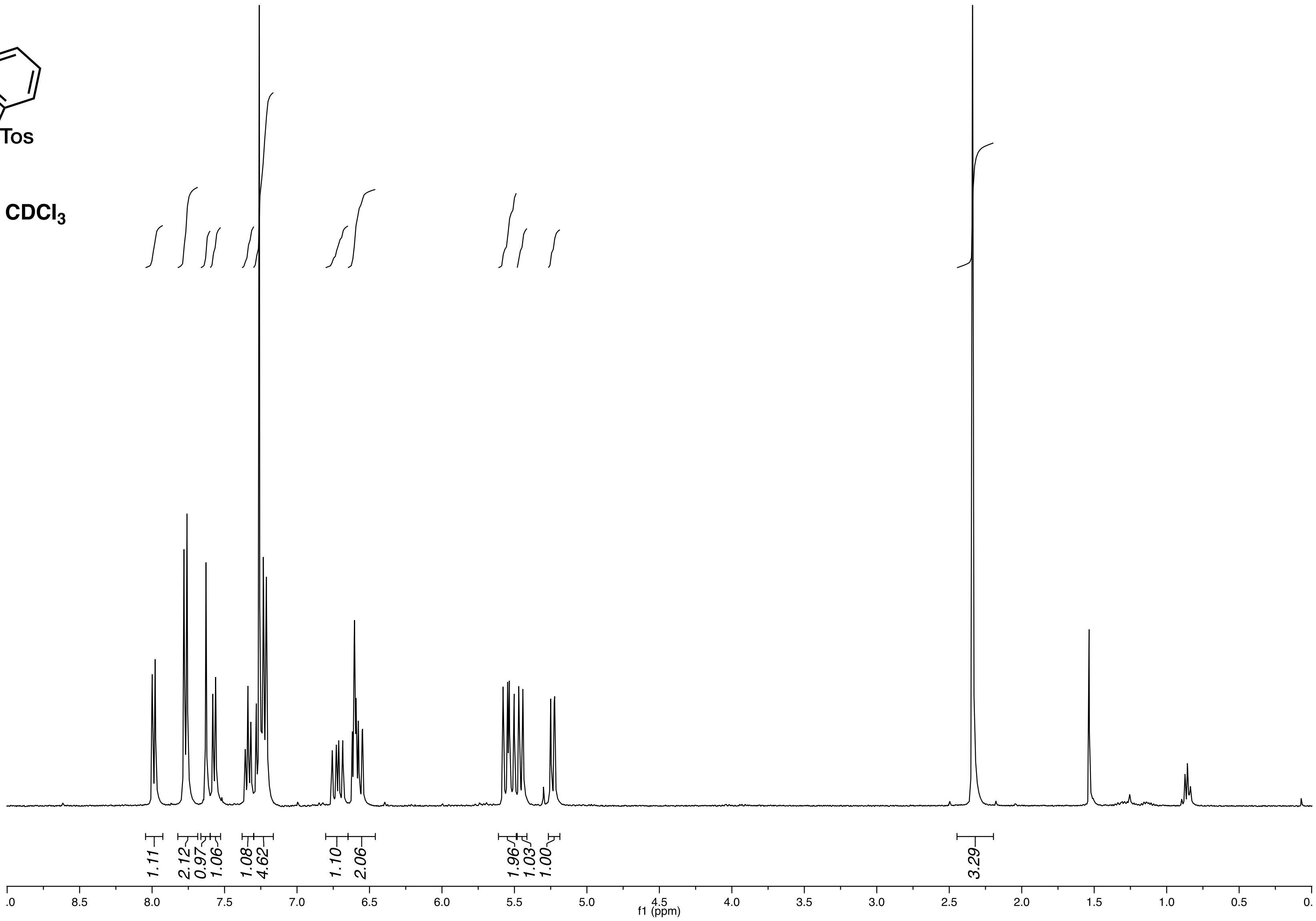

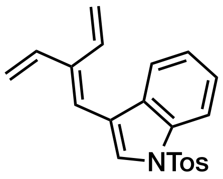

2l

100 MHz, $CDCl_3$

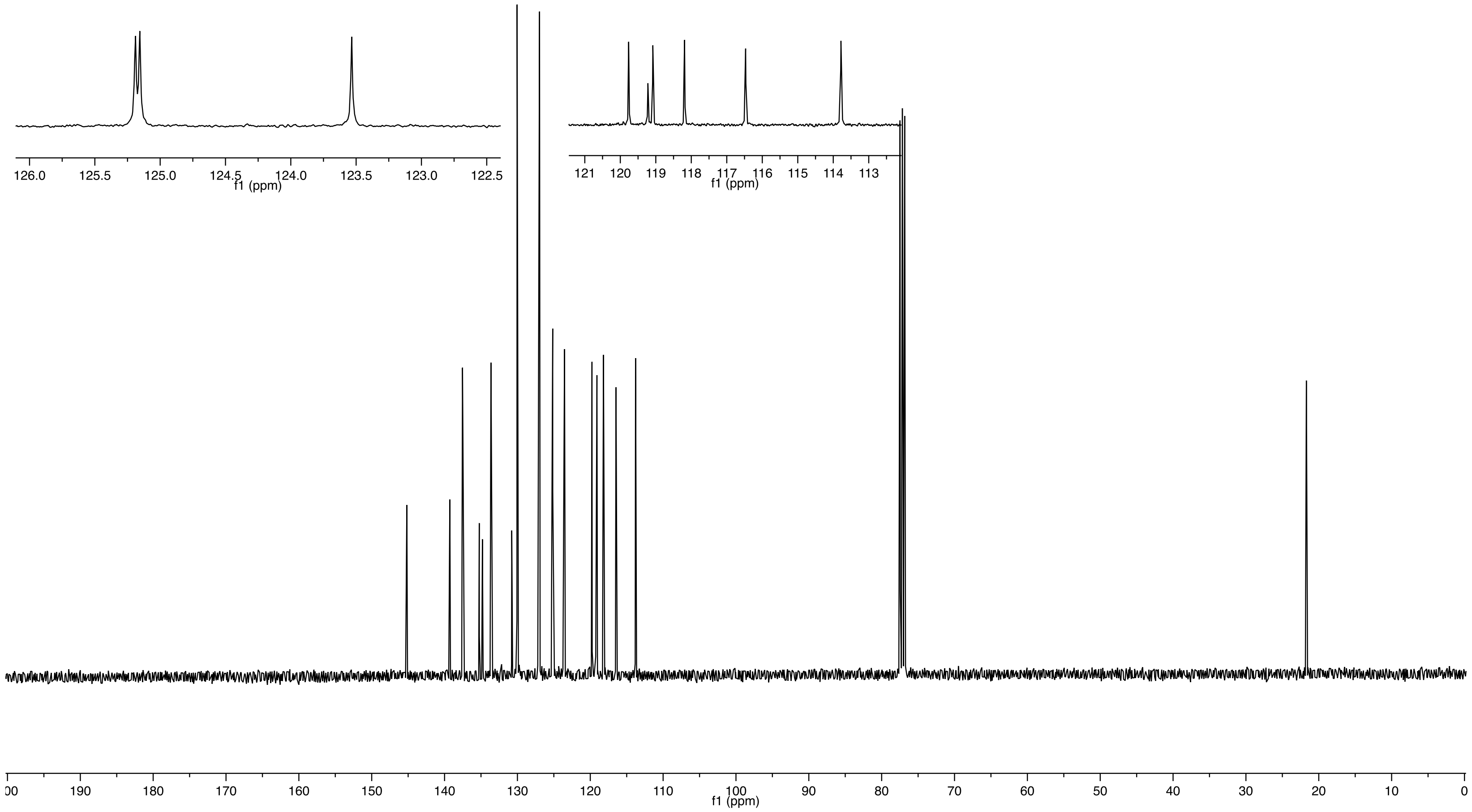

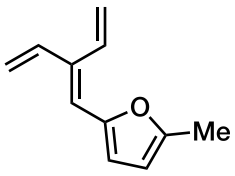

2m

400 MHz, $CDCl_3$

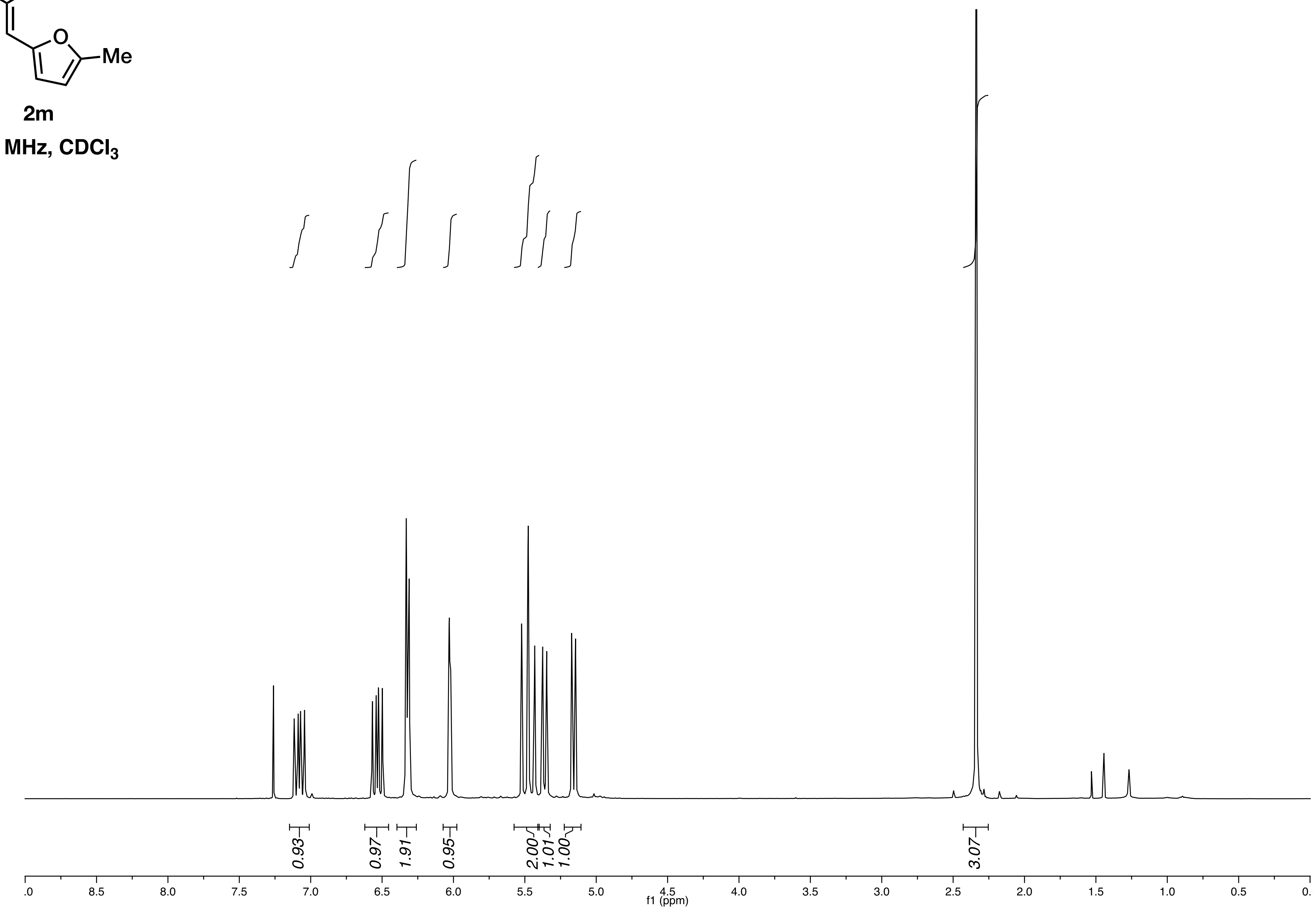

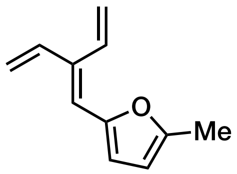

2m

100 MHz, $CDCl_3$

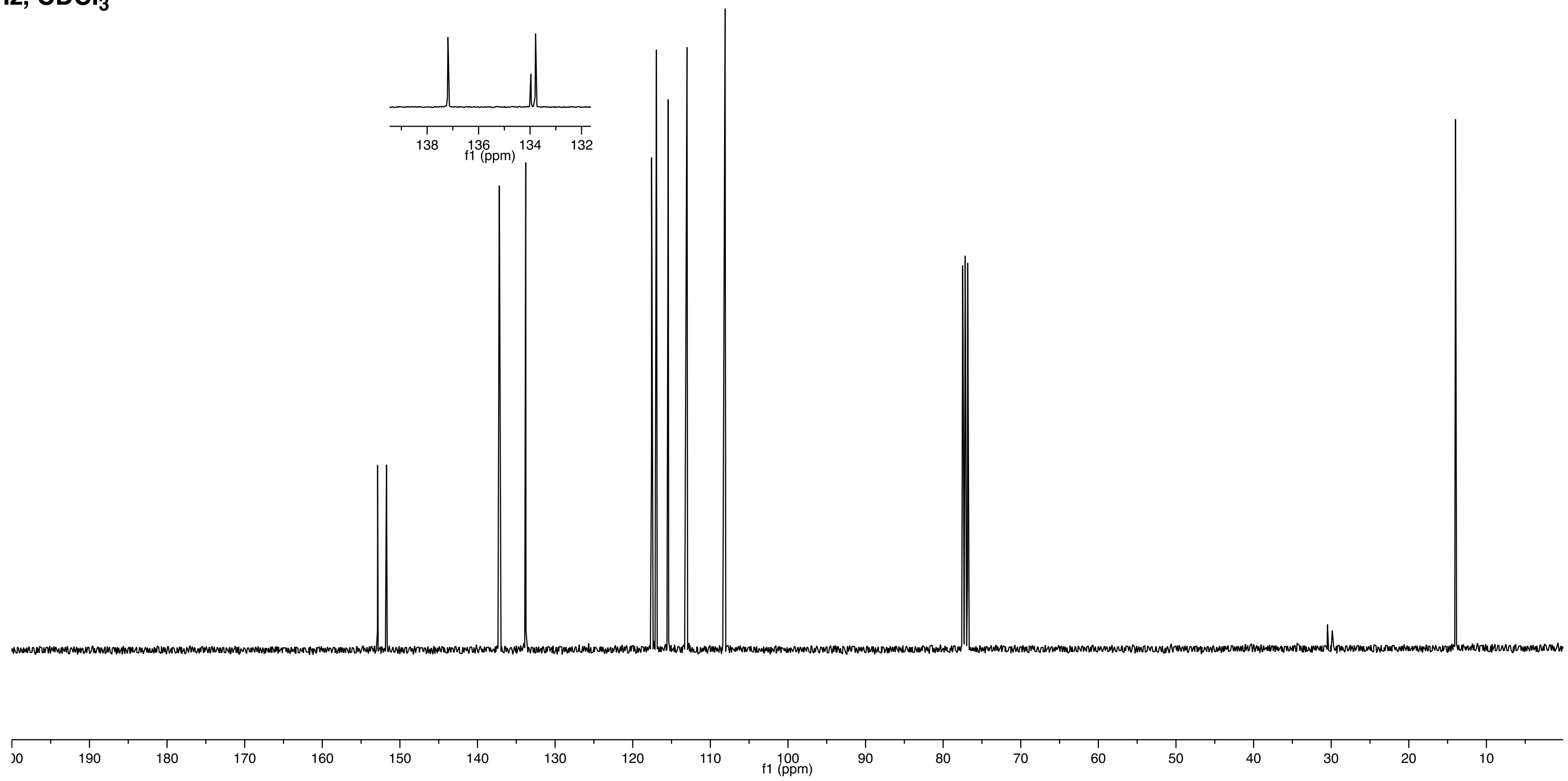

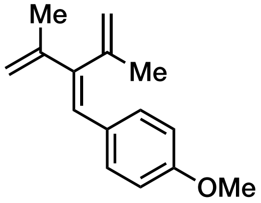

2n
400 MHz, $CDCl_3$

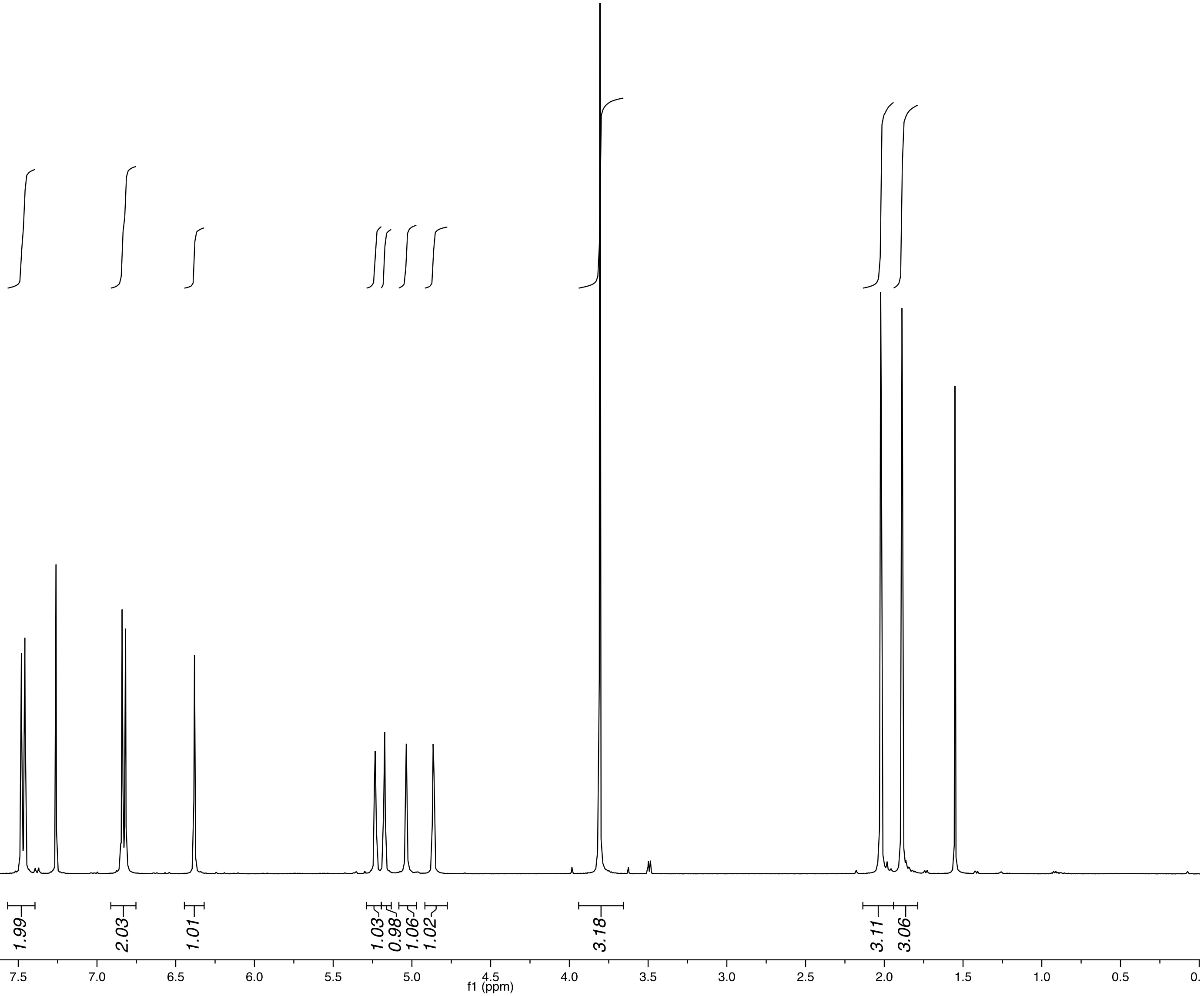

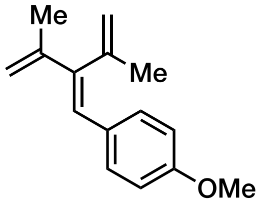

2n
100 MHz, $CDCl_3$

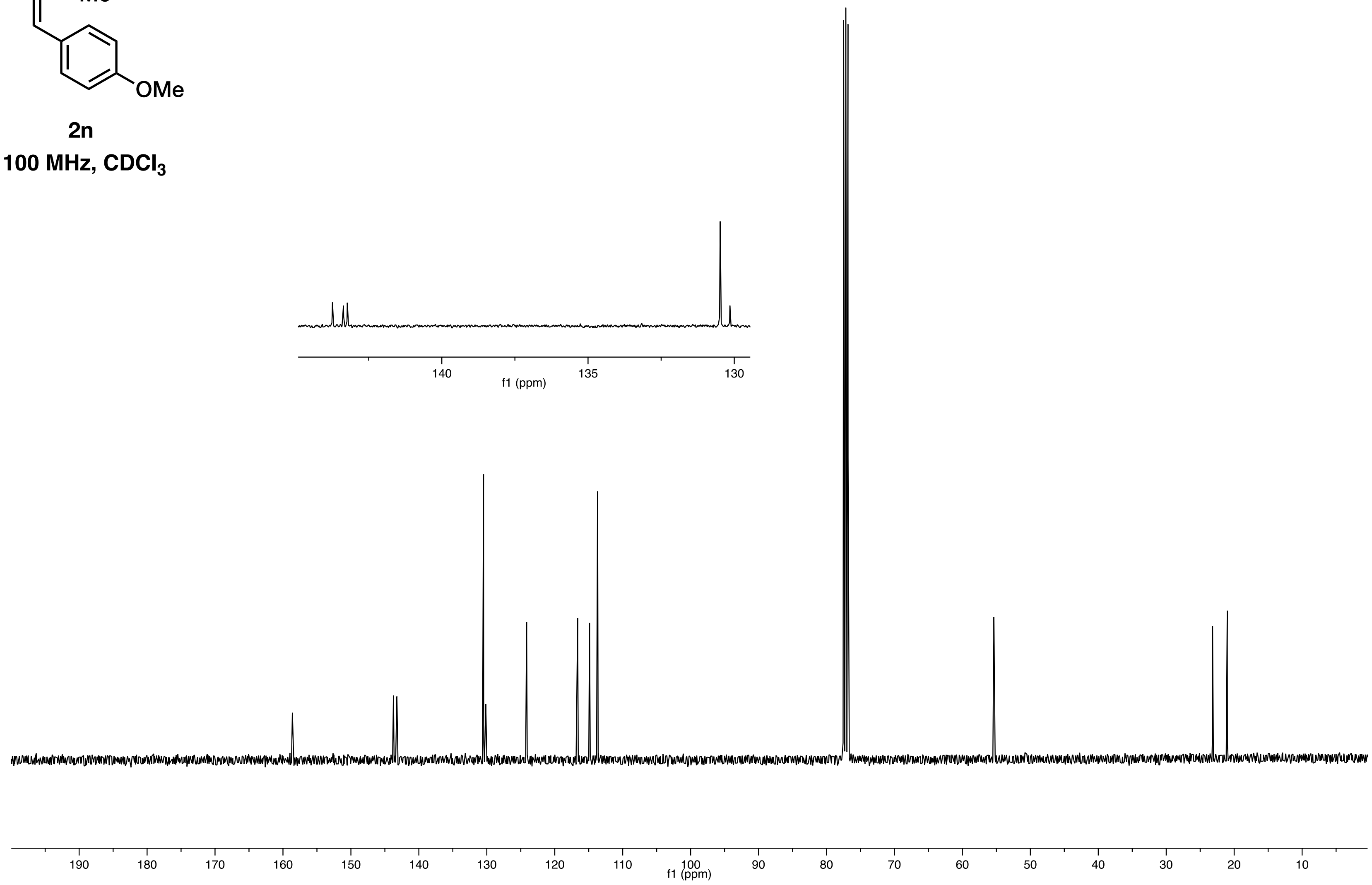

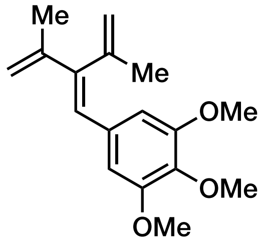

2o

400 MHz, $CDCl_3$

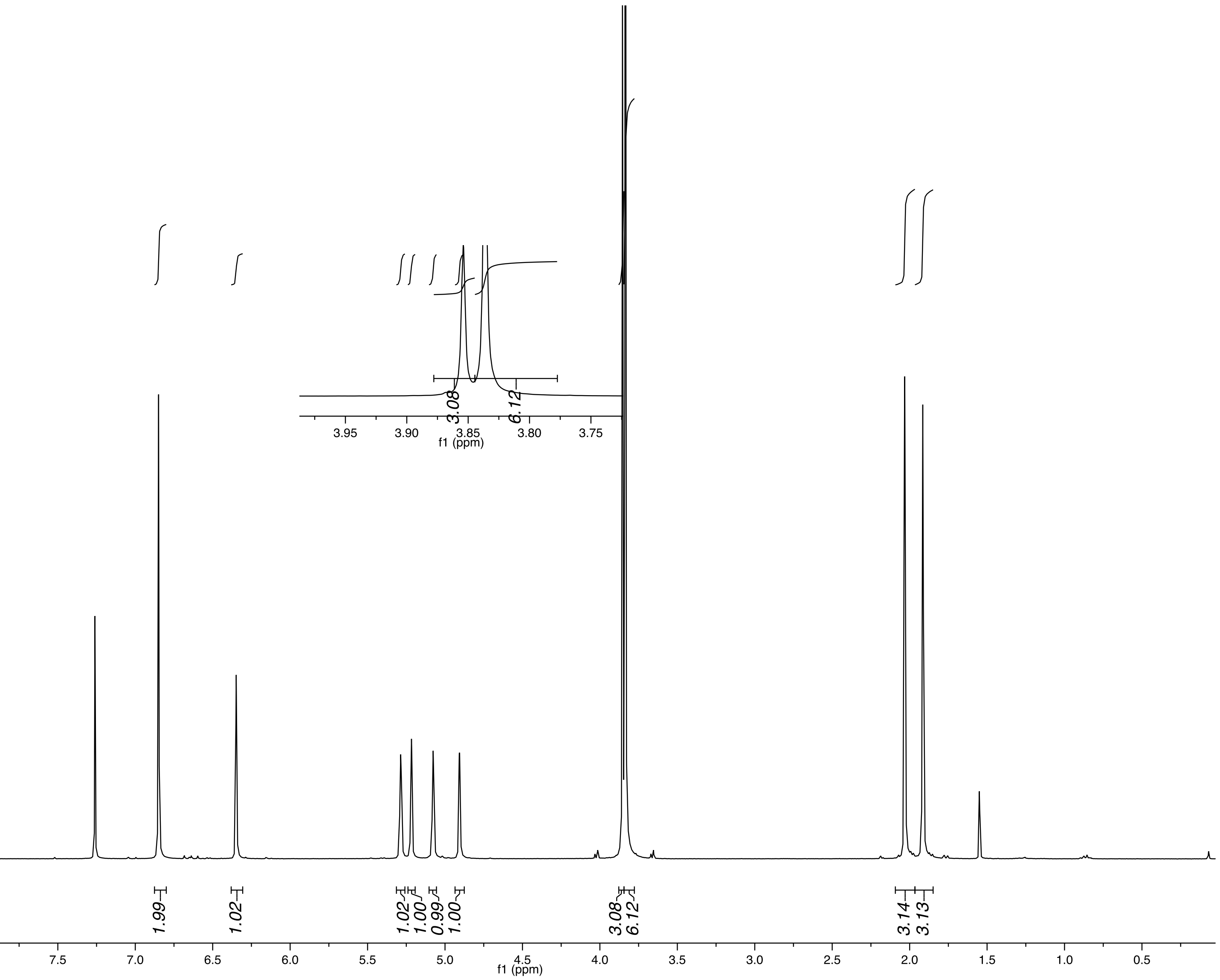

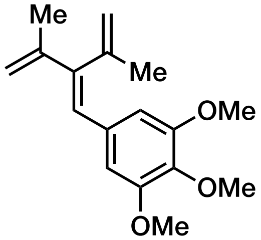

2o

100 MHz, $CDCl_3$

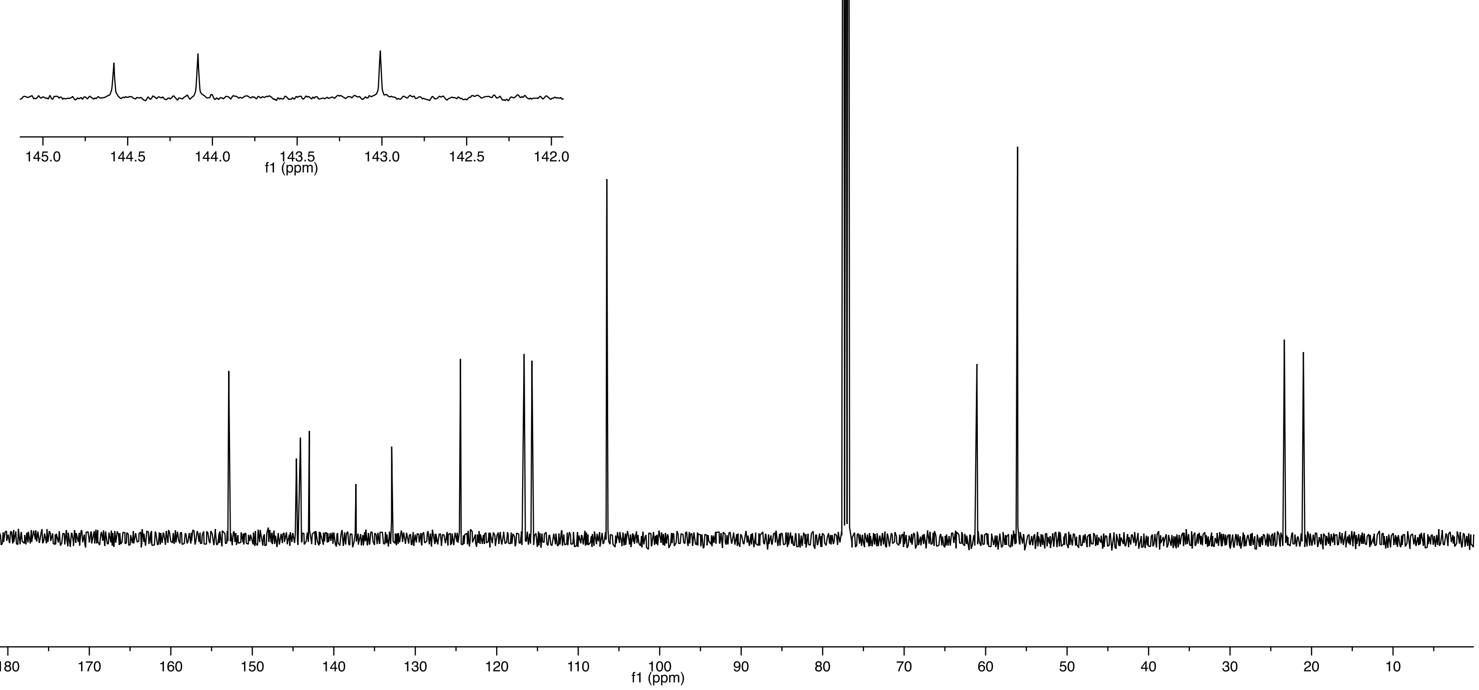

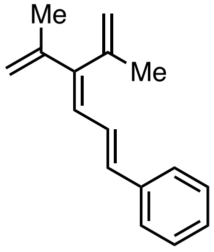

2p

400 MHz, $CDCl_3$

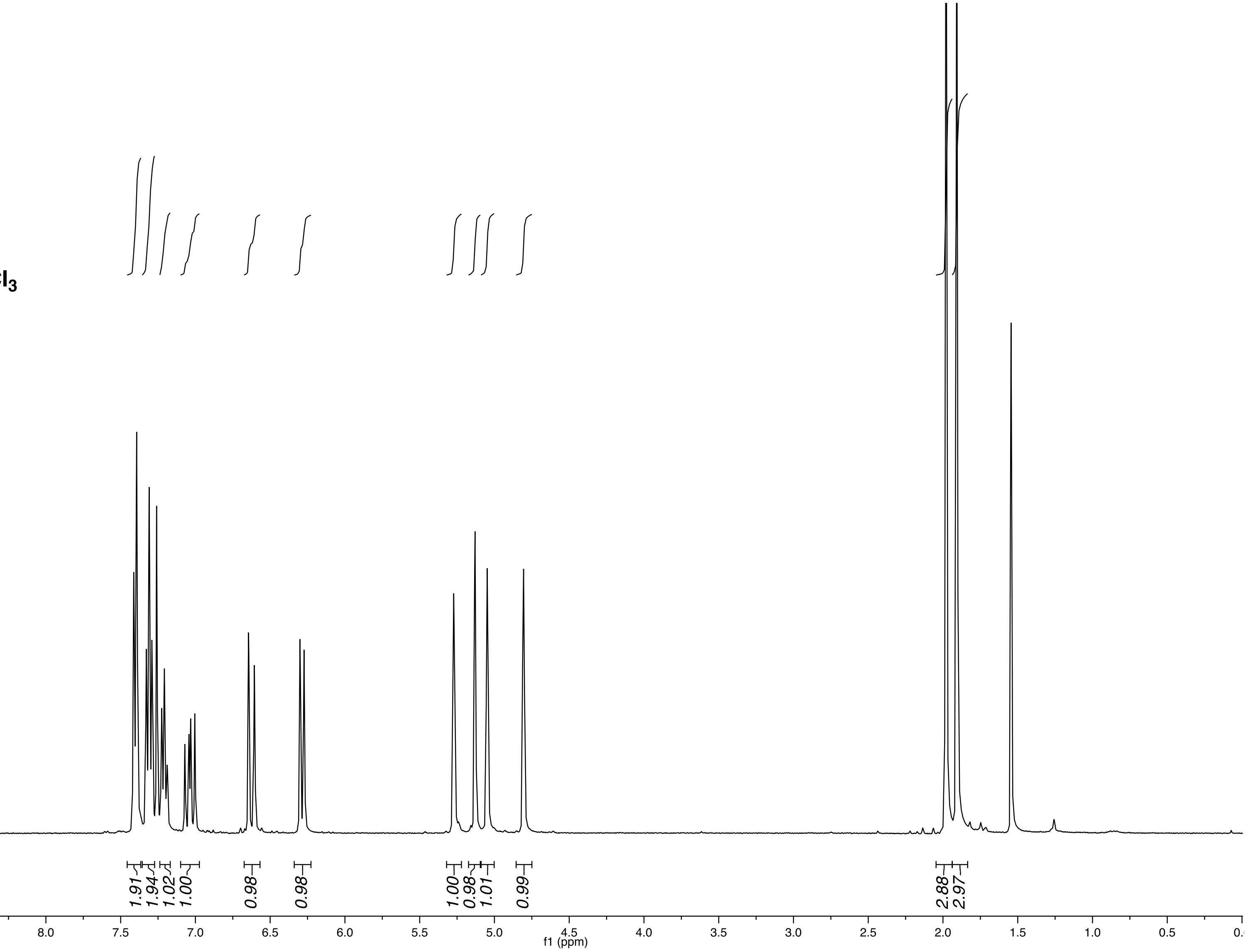

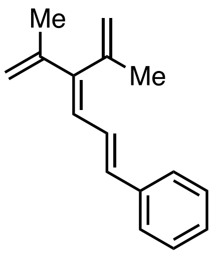

2p

100 MHz, $CDCl_3$

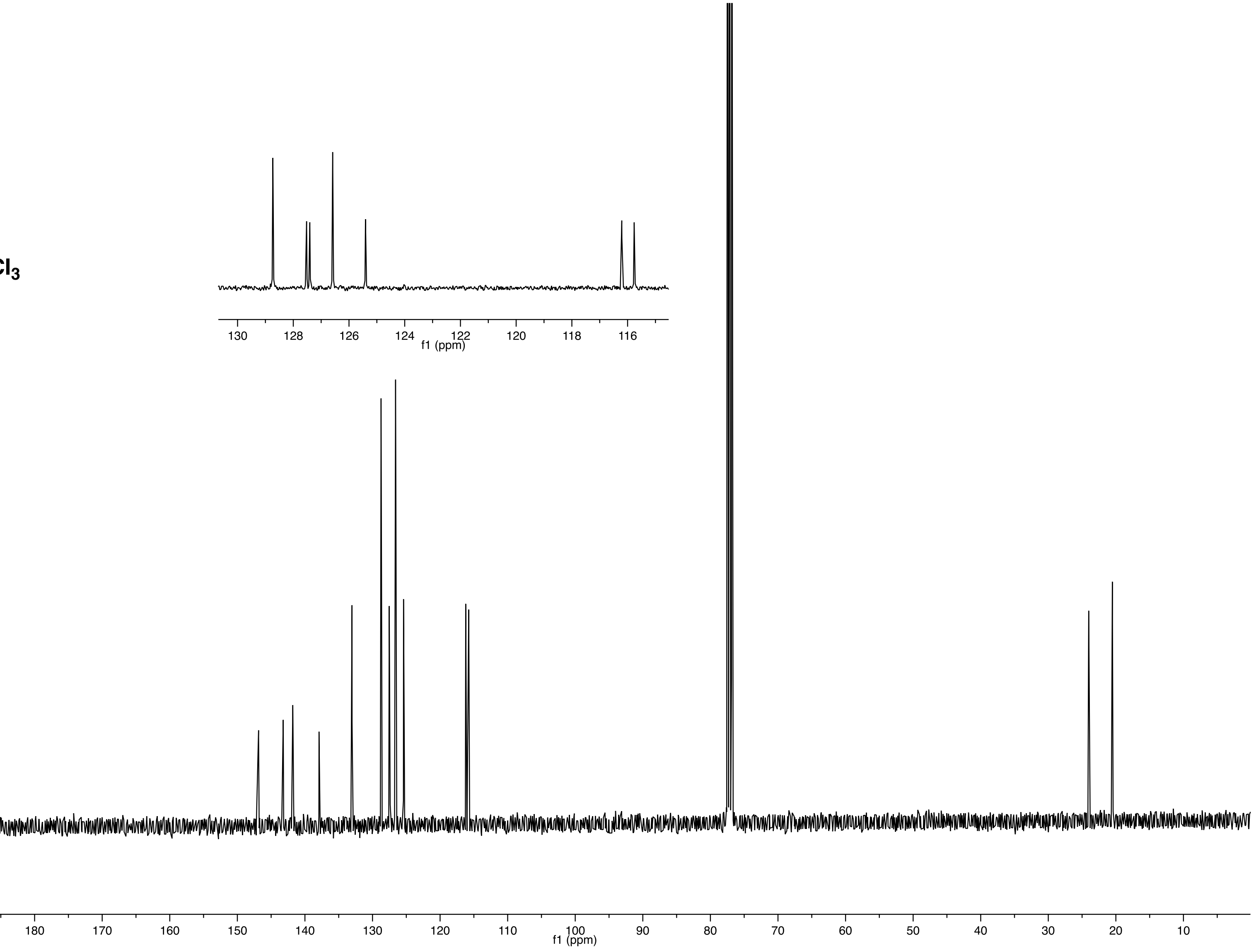

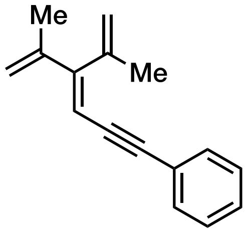

2q
400 MHz, $CDCl_3$

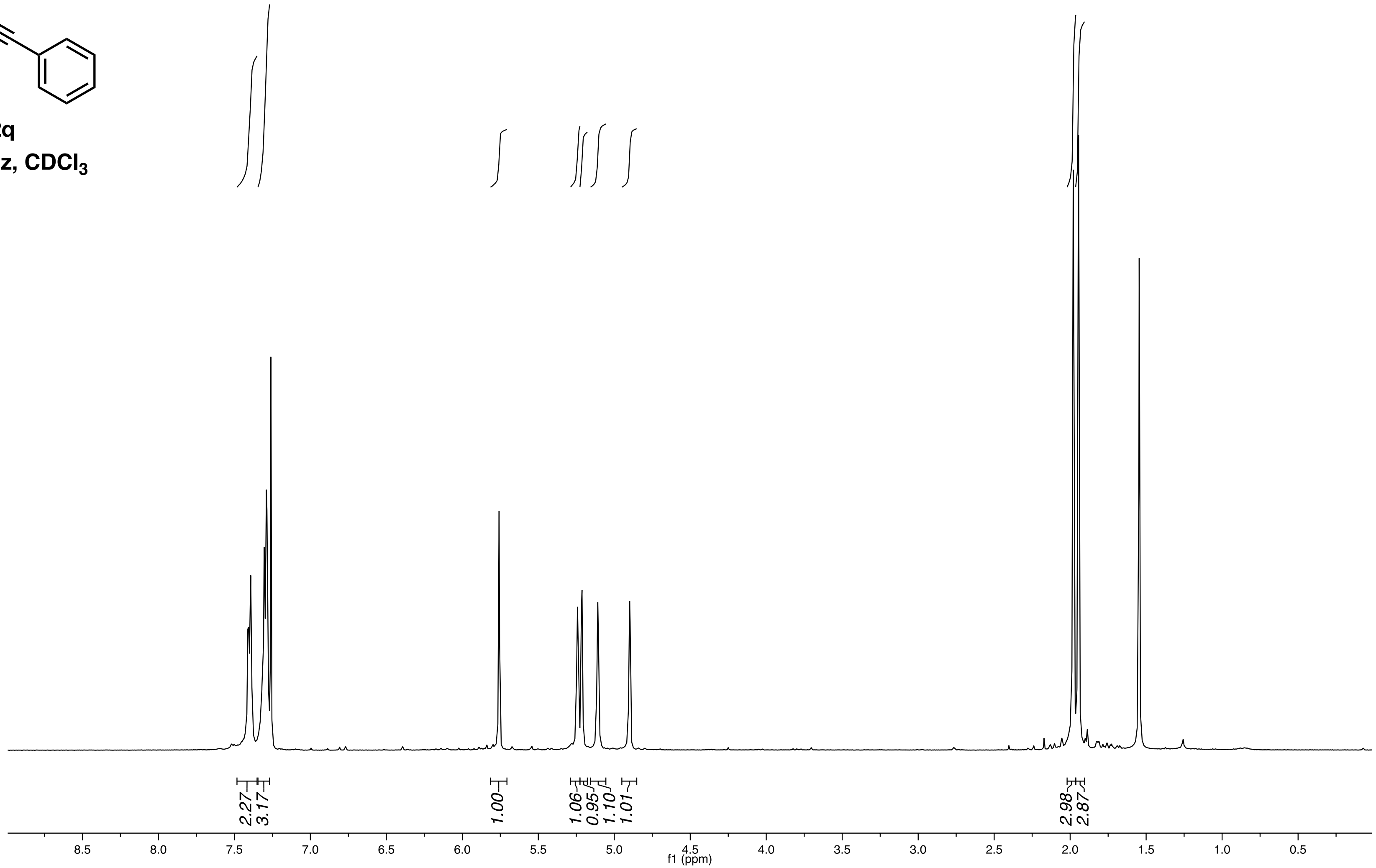

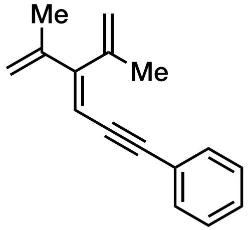

2q

100 MHz, CDCl$_3$

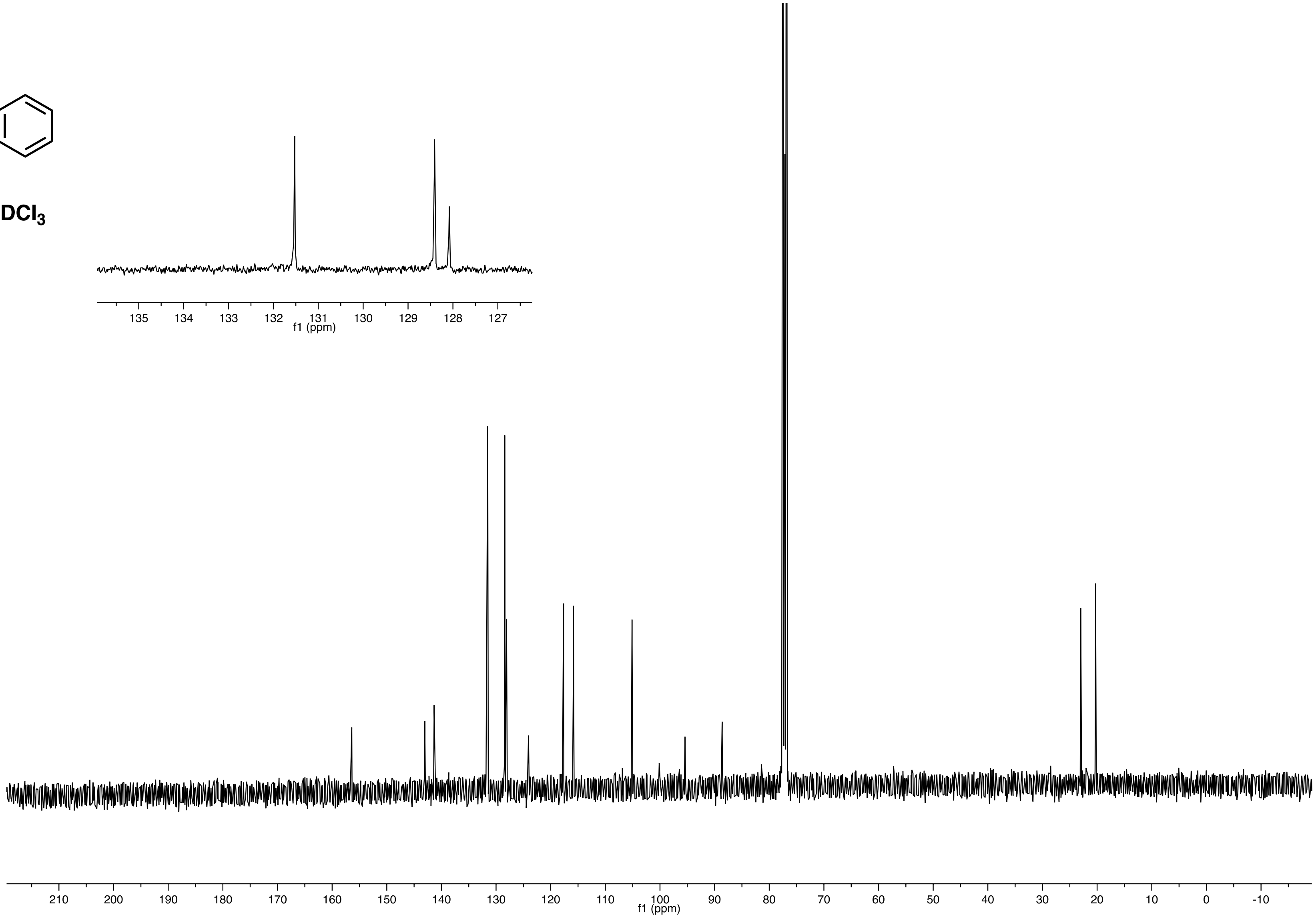

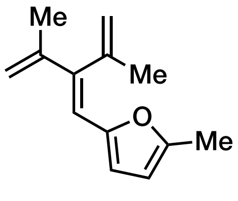

2r

400 MHz, $CDCl_3$

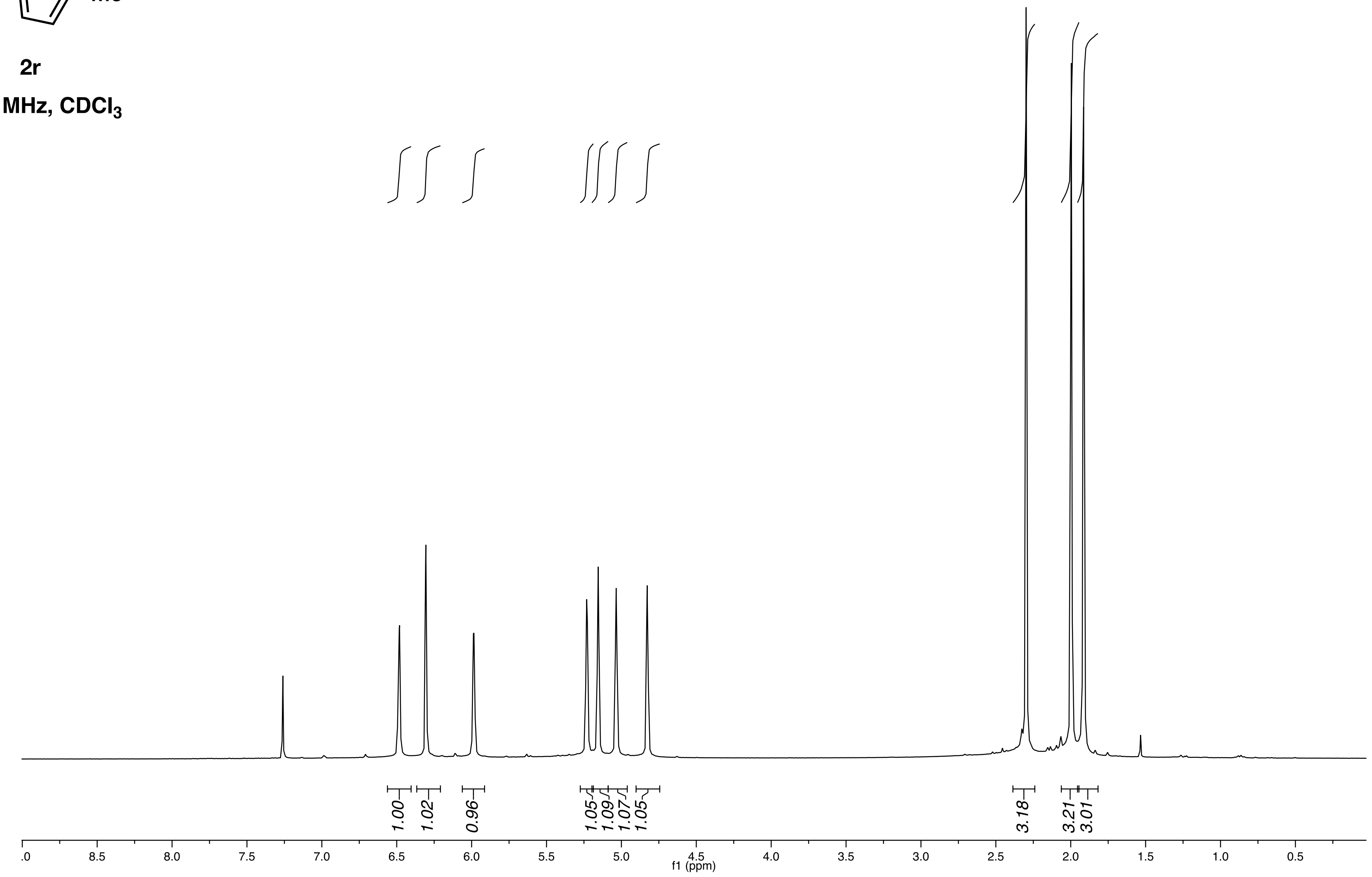

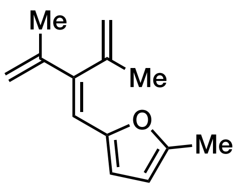

2r

100 MHz, $CDCl_3$

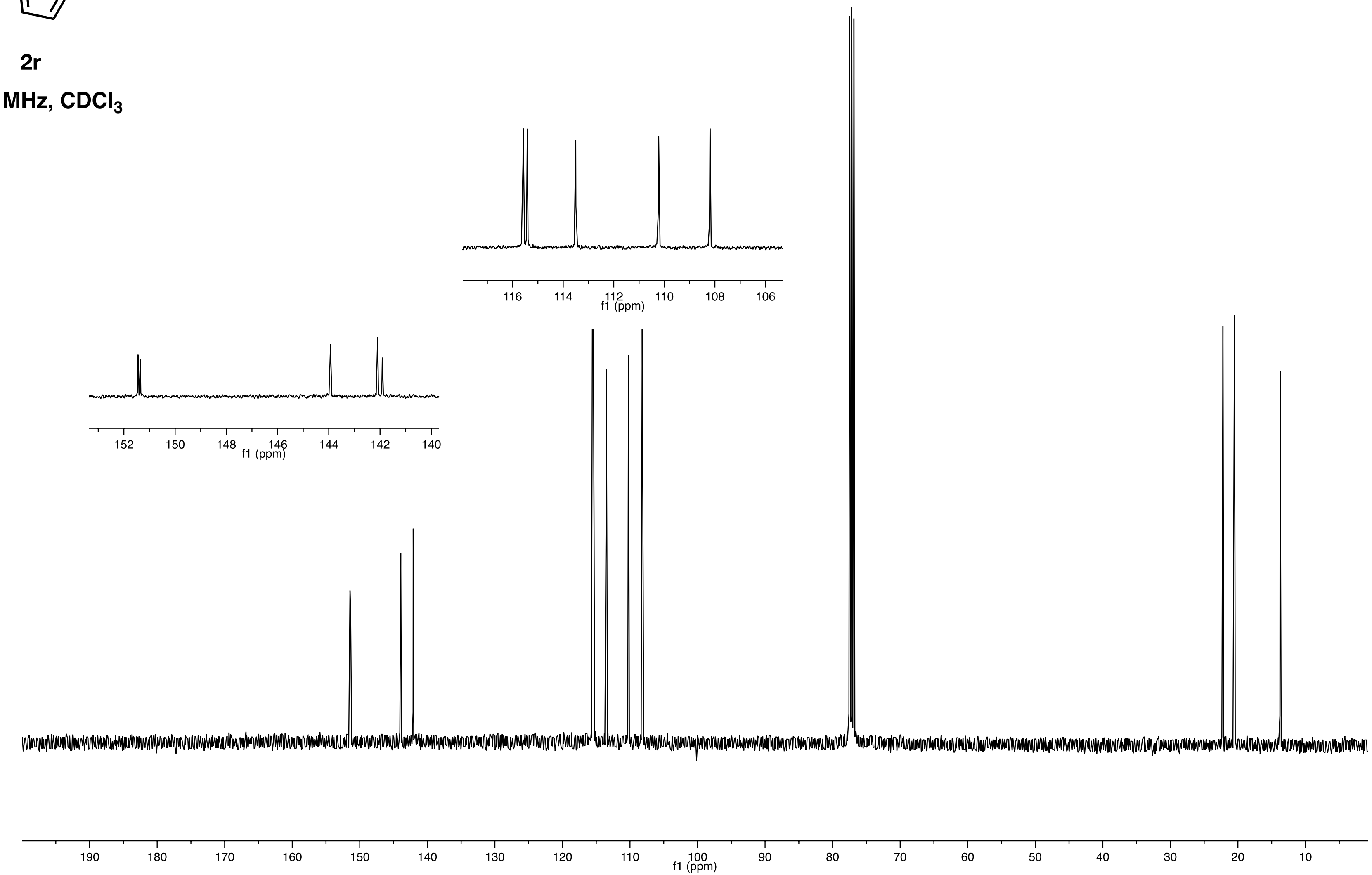

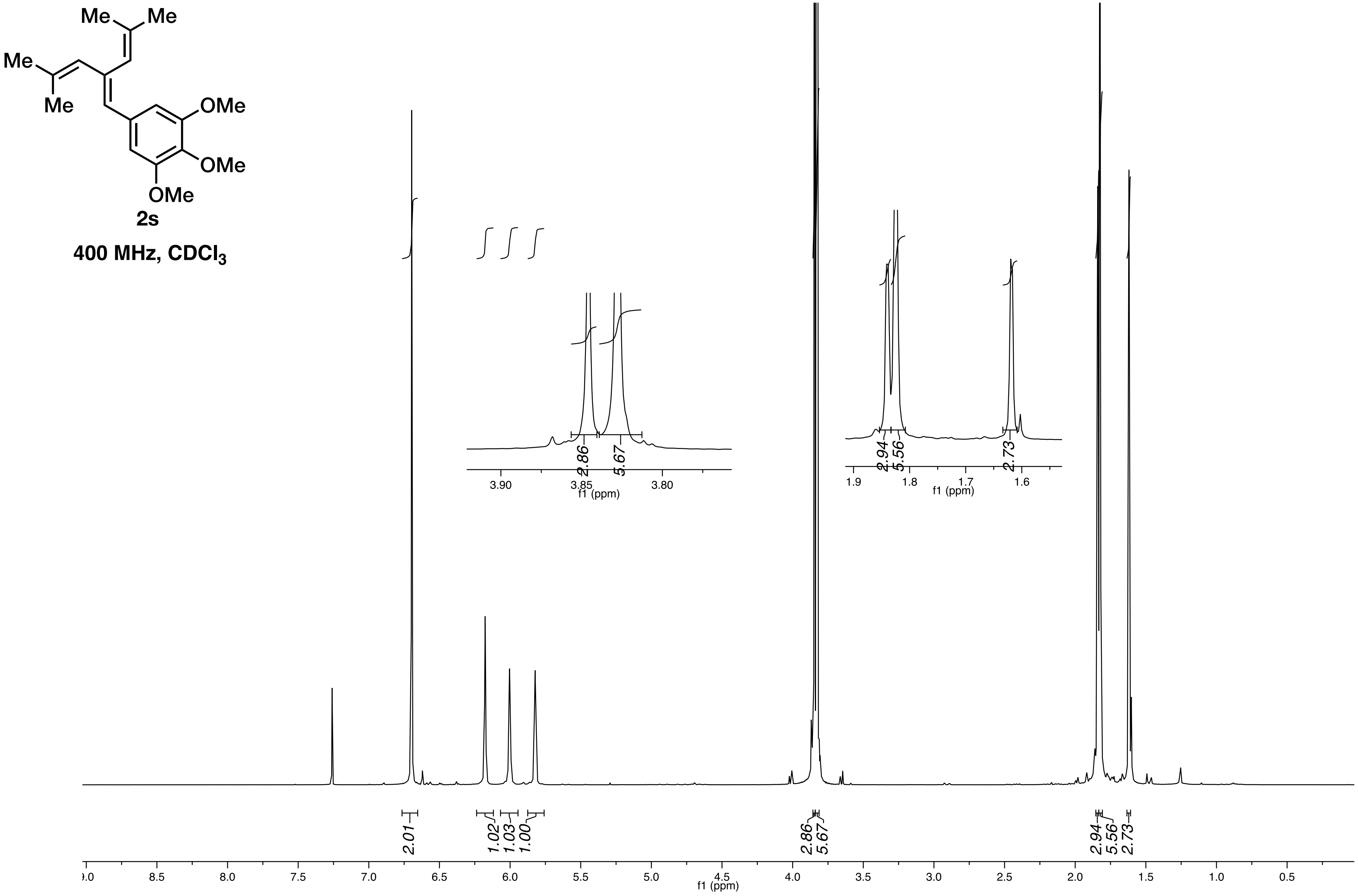
Me
Me
Me
Me
OMe
OMe
OMe
2s
400 MHz, CDCl3
2.01
1.02
1.03
1.00
2.86
5.67
2.94
5.56
2.73
f1 (ppm)

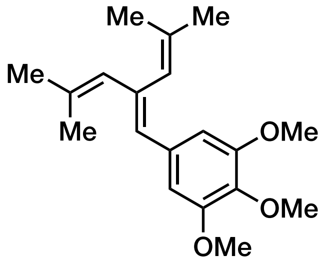

2s

100 MHz, $CDCl_3$

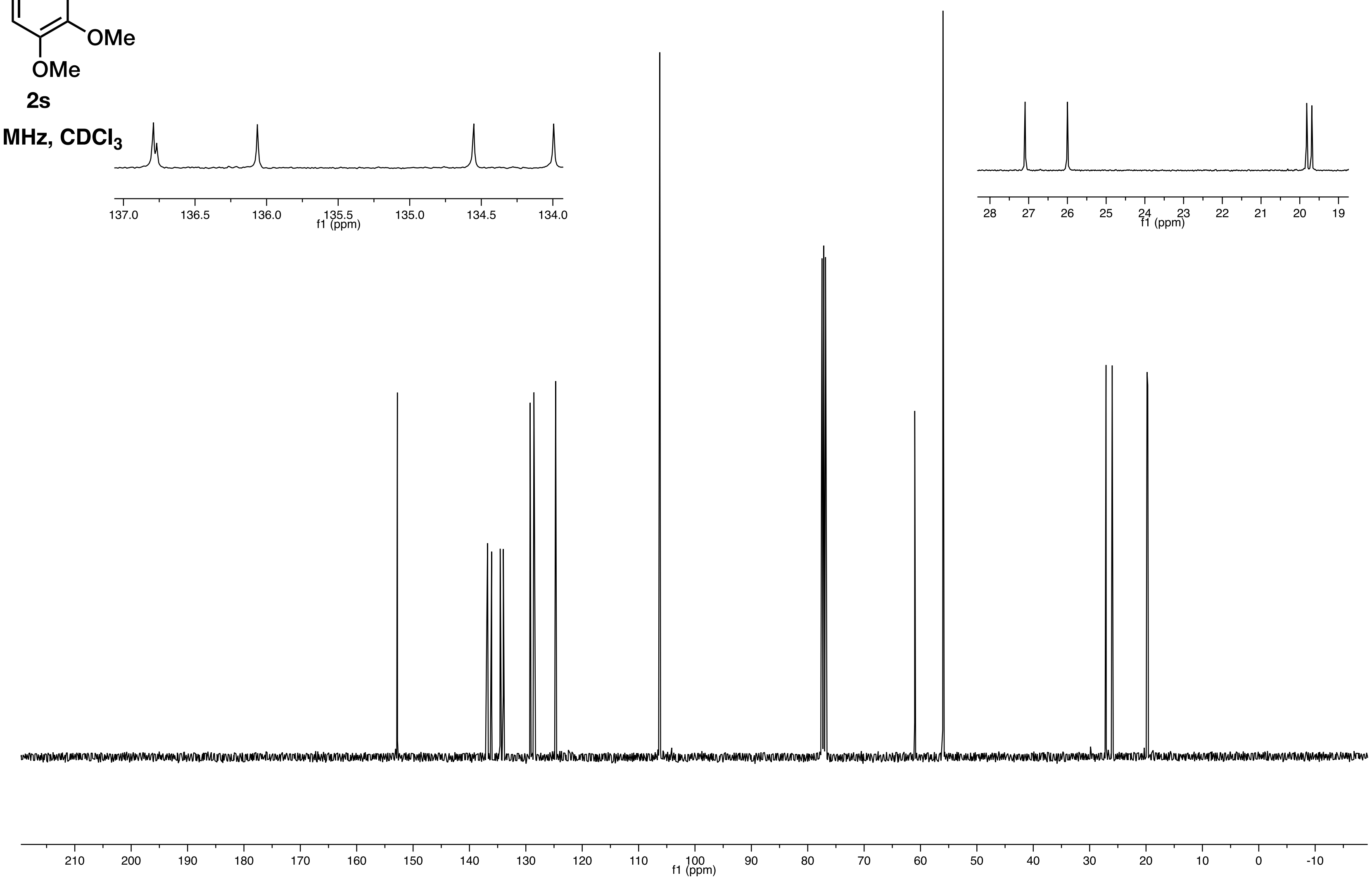

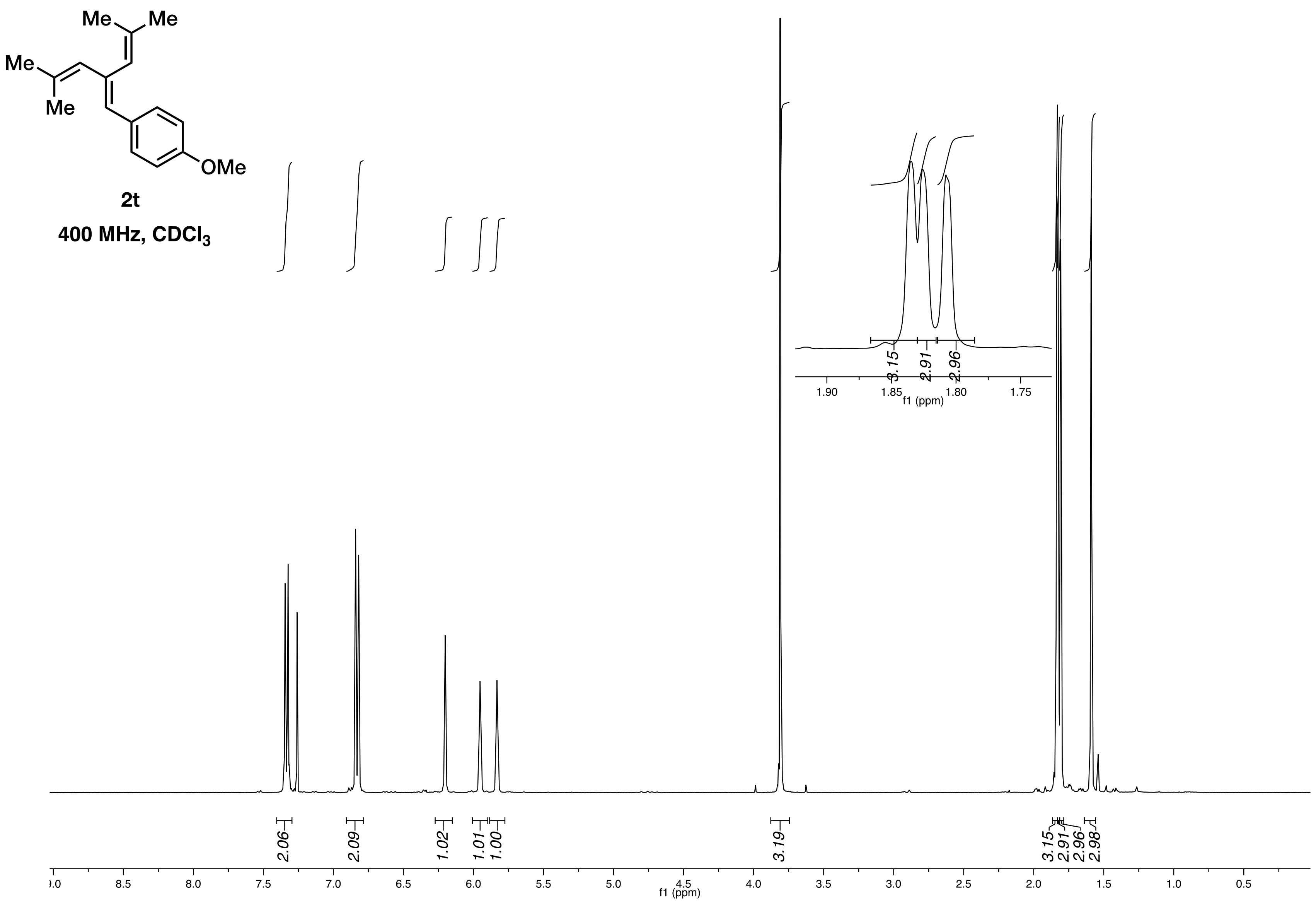

2t

400 MHz, $CDCl_3$

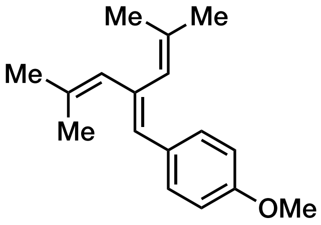

2t

100 MHz, $CDCl_3$

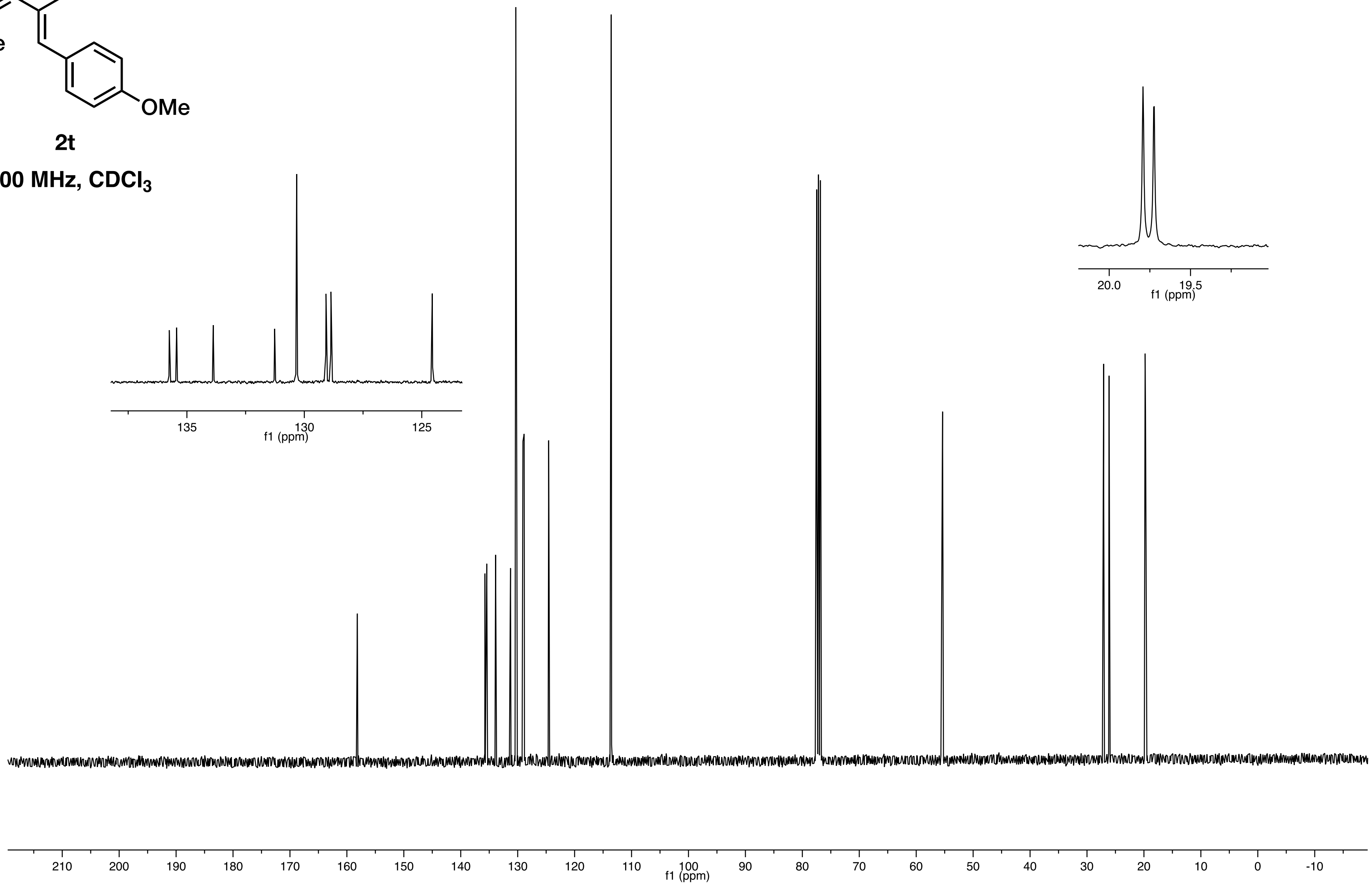

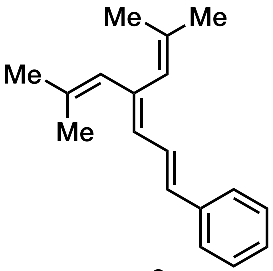

2u

400 MHz, $CDCl_3$

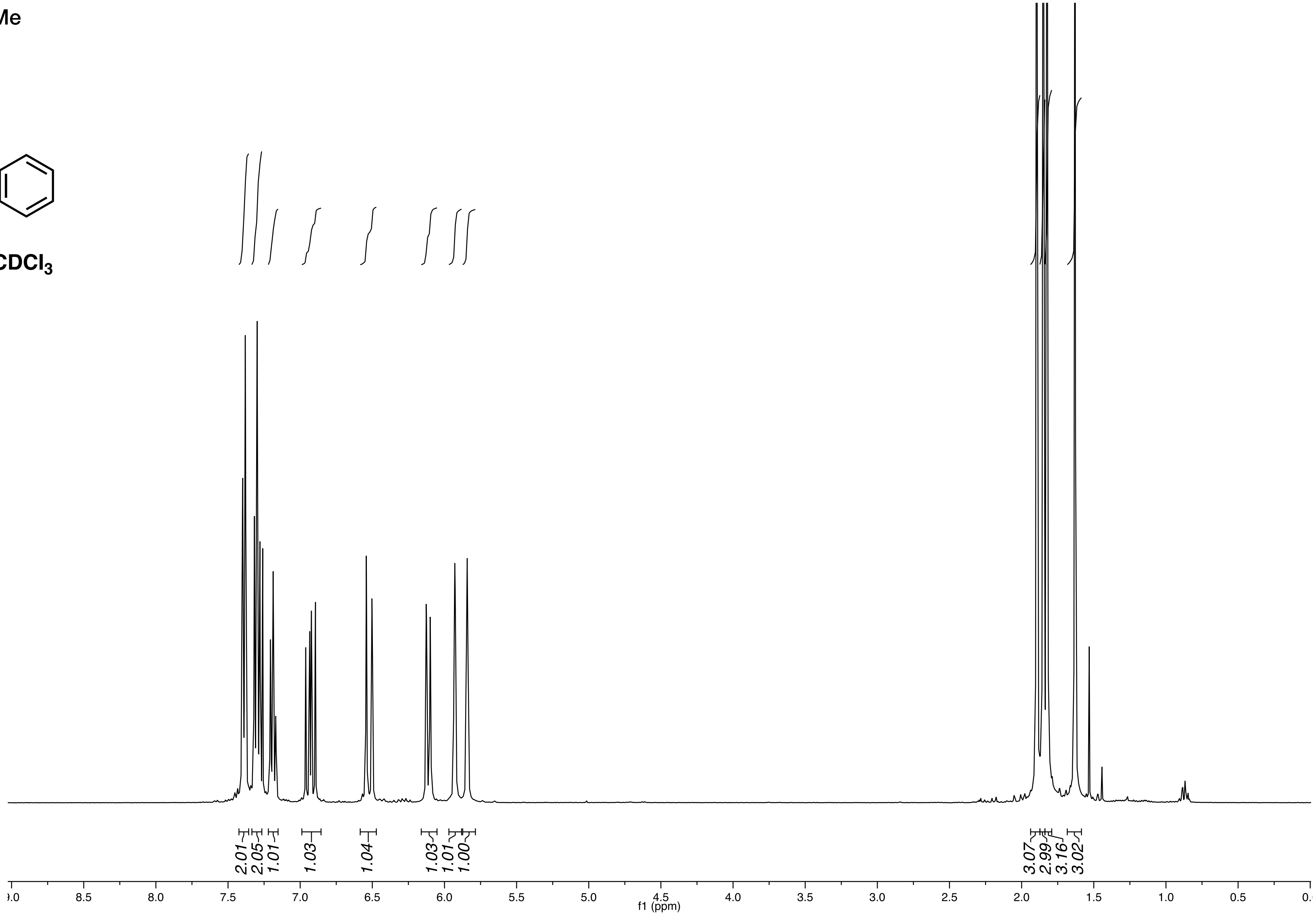

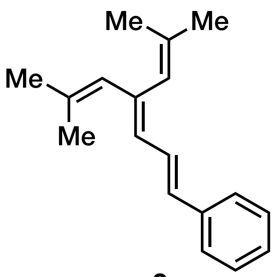

2u

100 MHz, CDCl$_3$

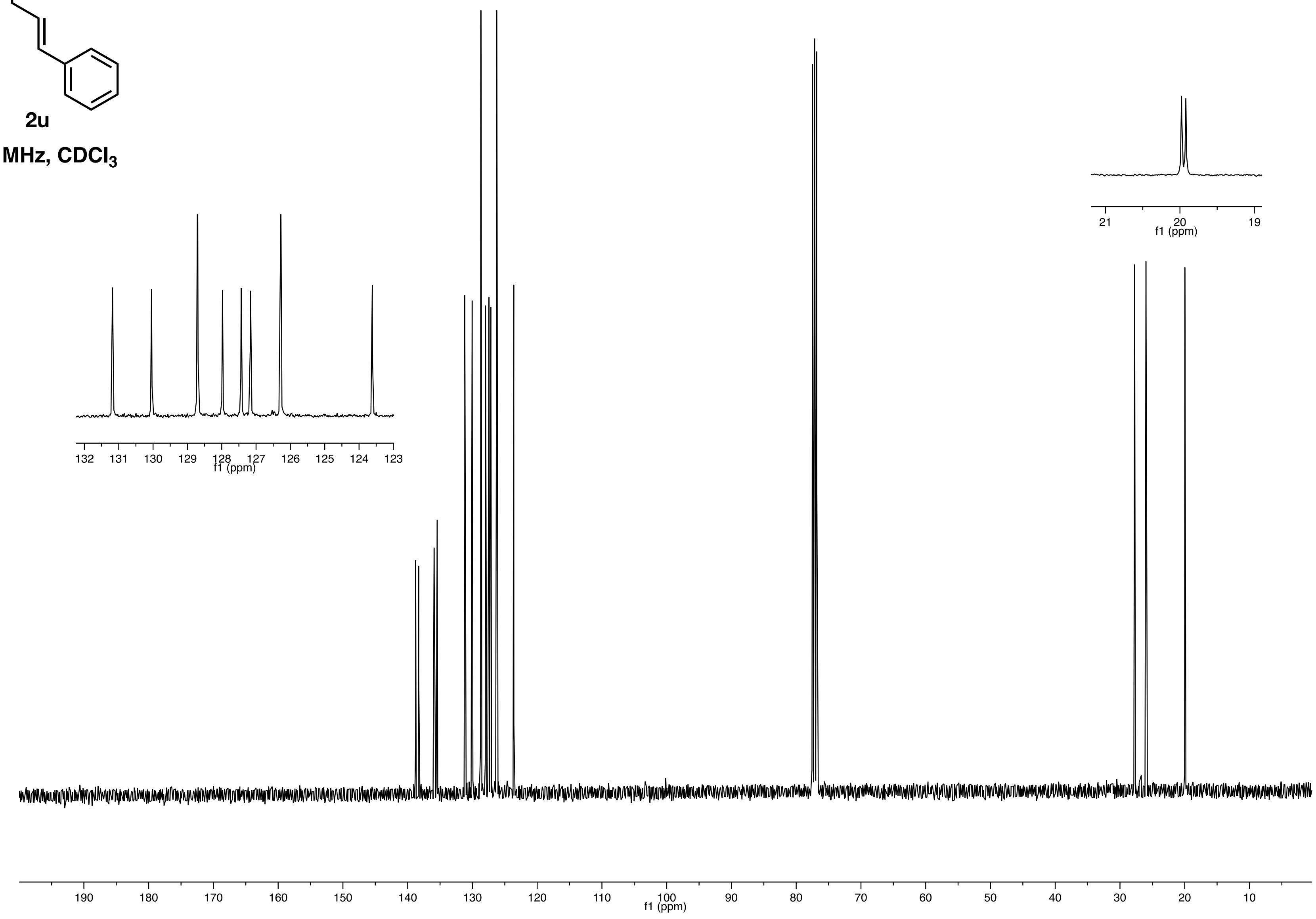

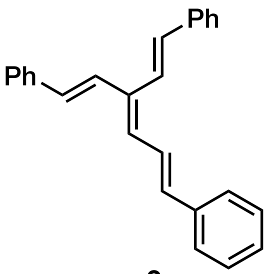

2v

700 MHz, CD_3CN

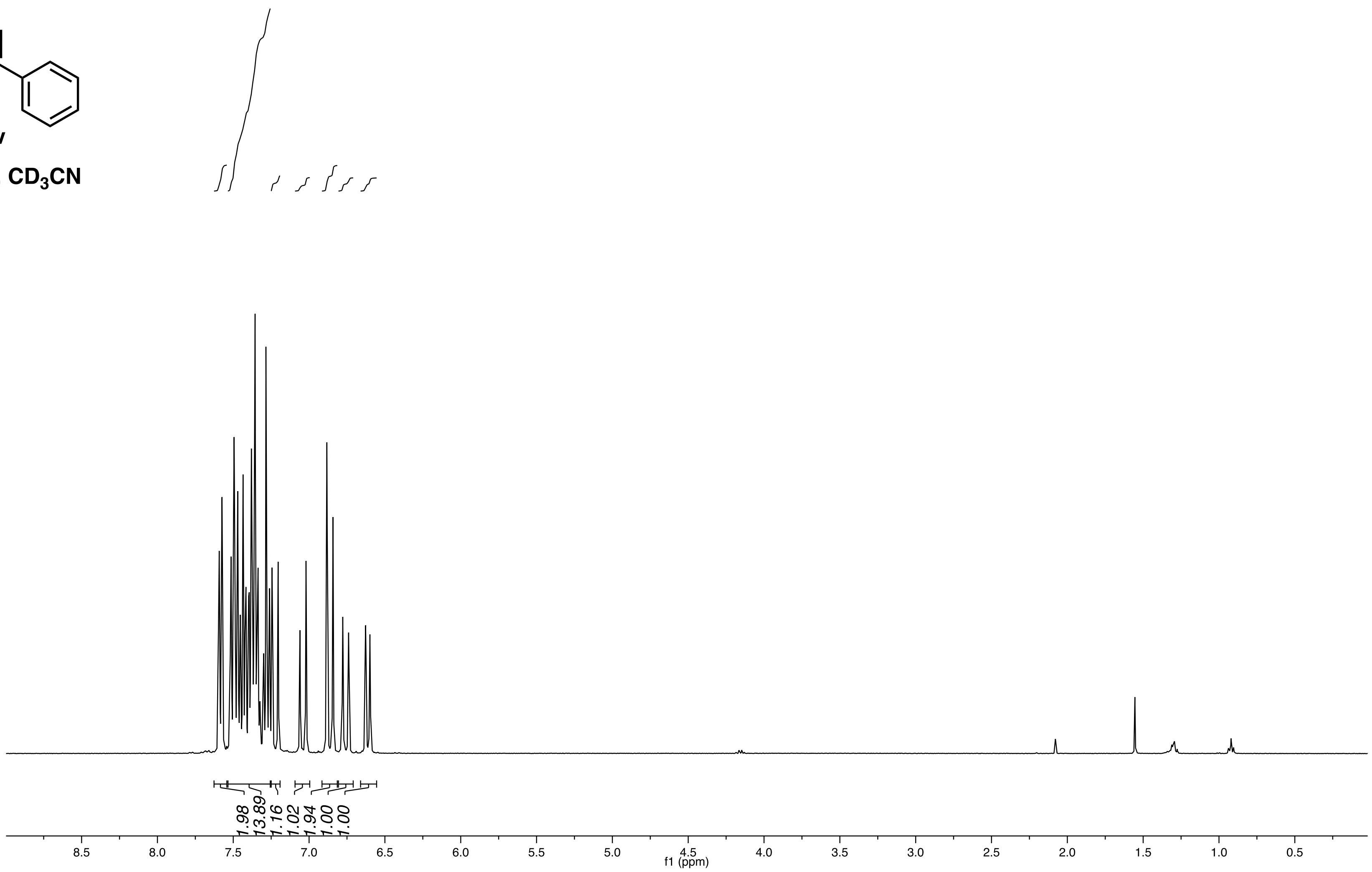

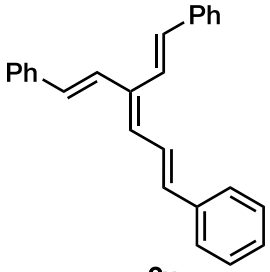

2v

176 MHz, CD_3CN

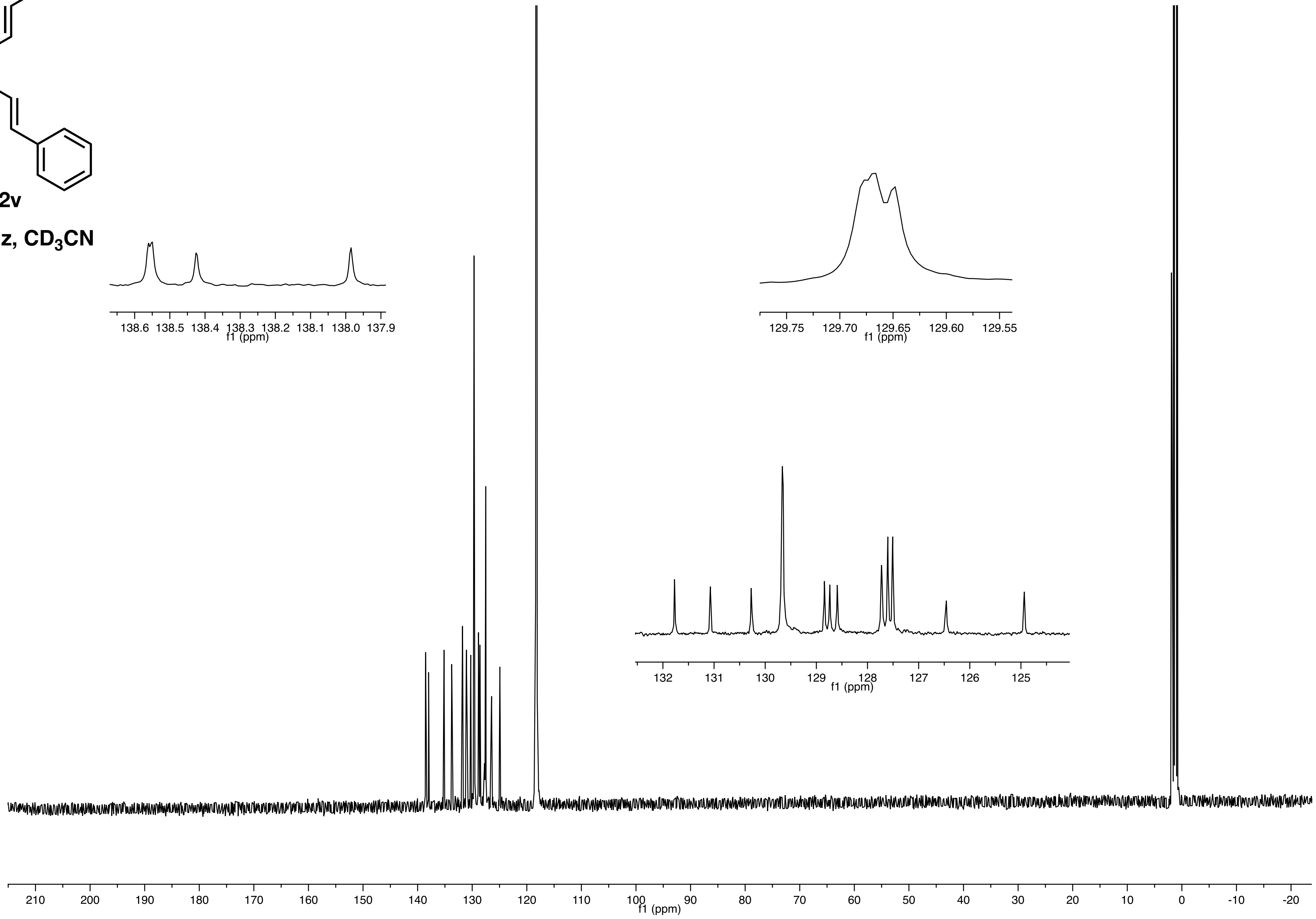

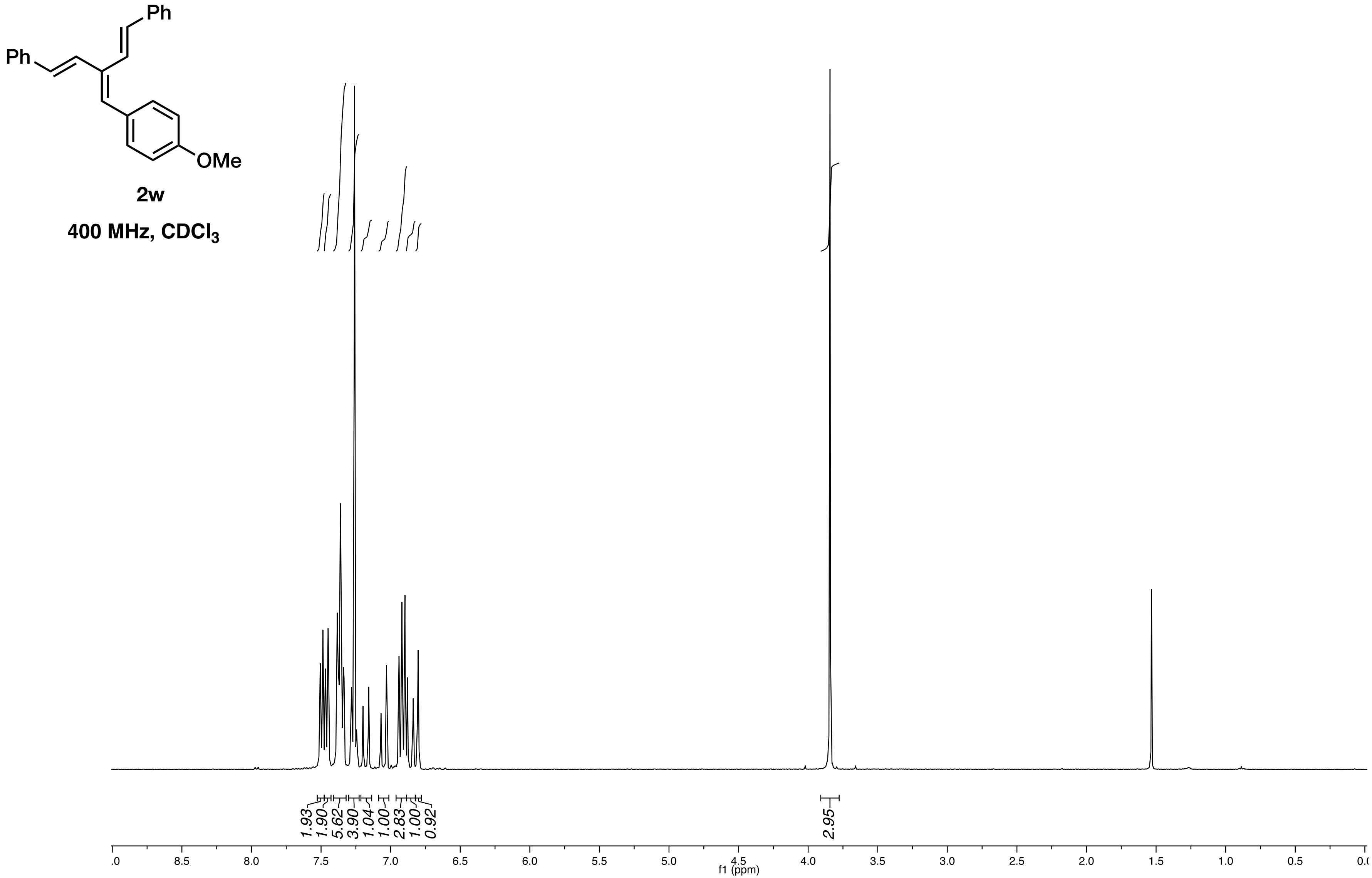
Ph
Ph
OMe
2w
400 MHz, CDCl3
1.93
1.90
5.62
3.90
1.04
1.00
2.83
1.00
0.92
2.95
f1 (ppm)

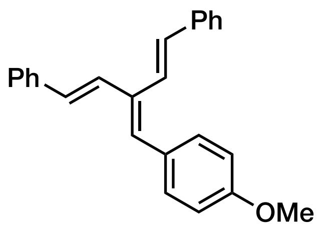

2w

100 MHz, $CDCl_3$

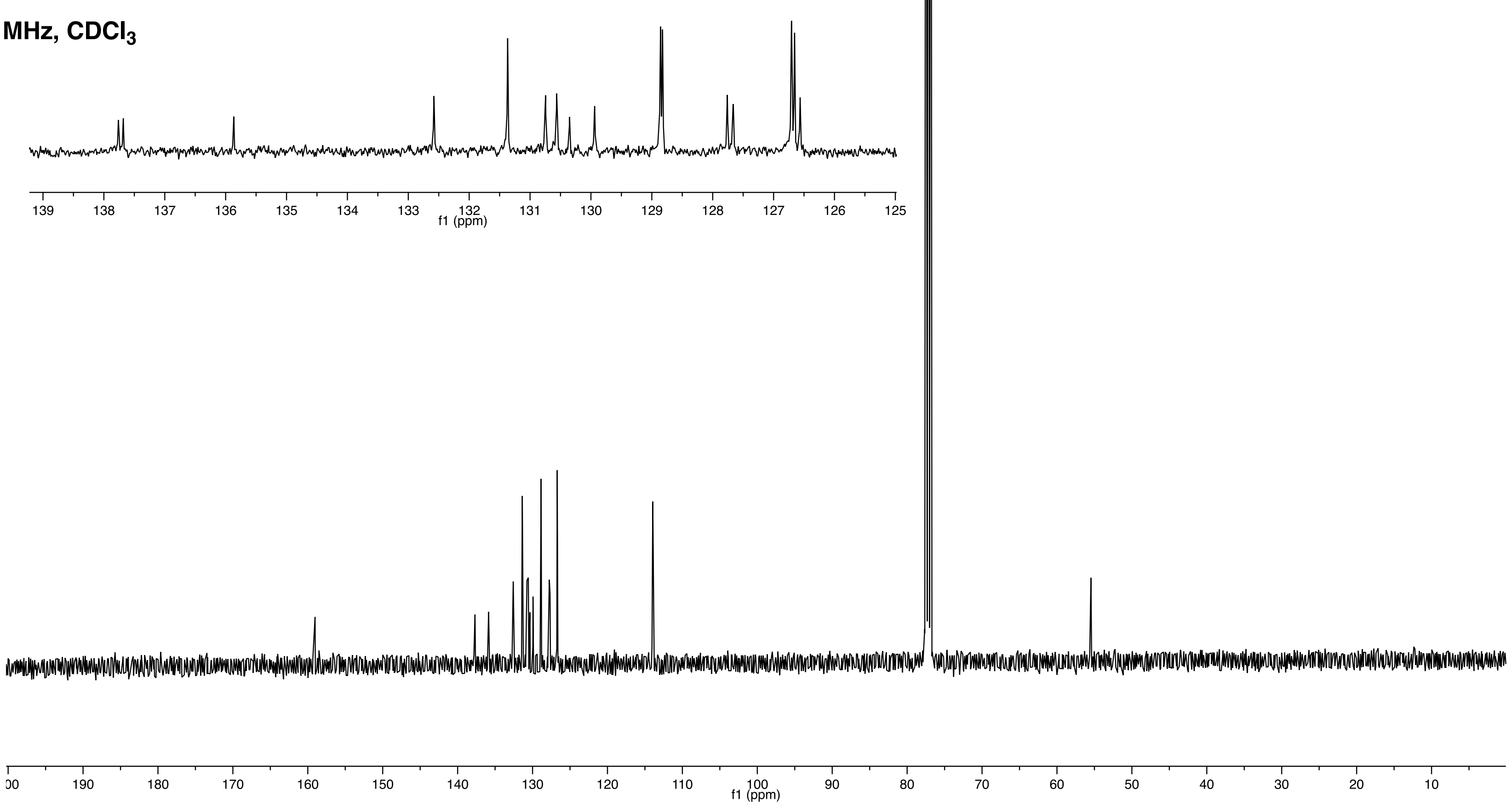

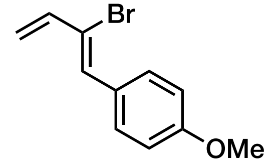

3a

400 MHz, $CDCl_3$

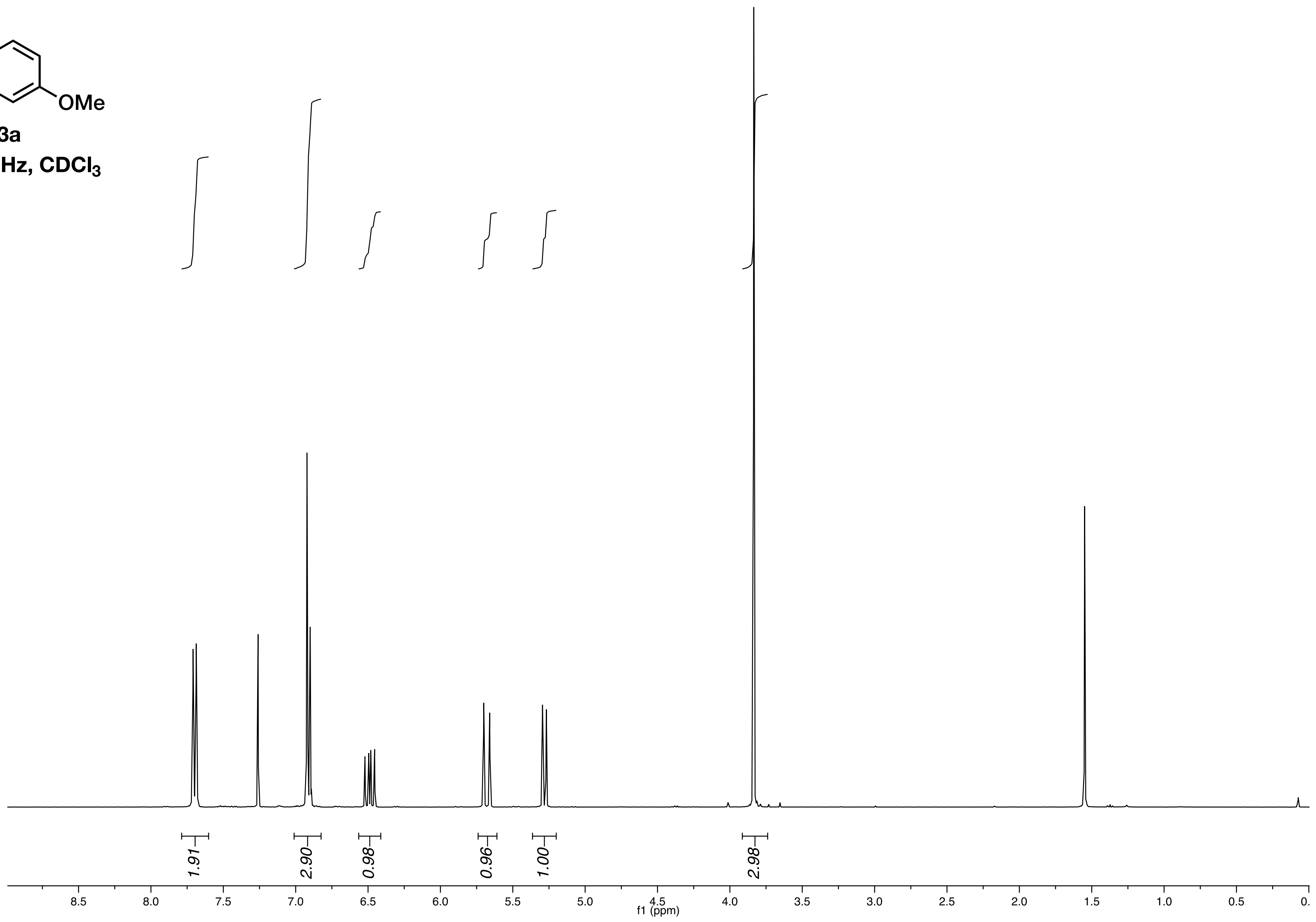

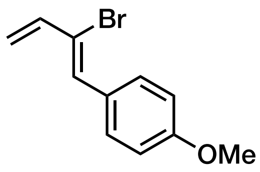

3a

100 MHz, $CDCl_3$

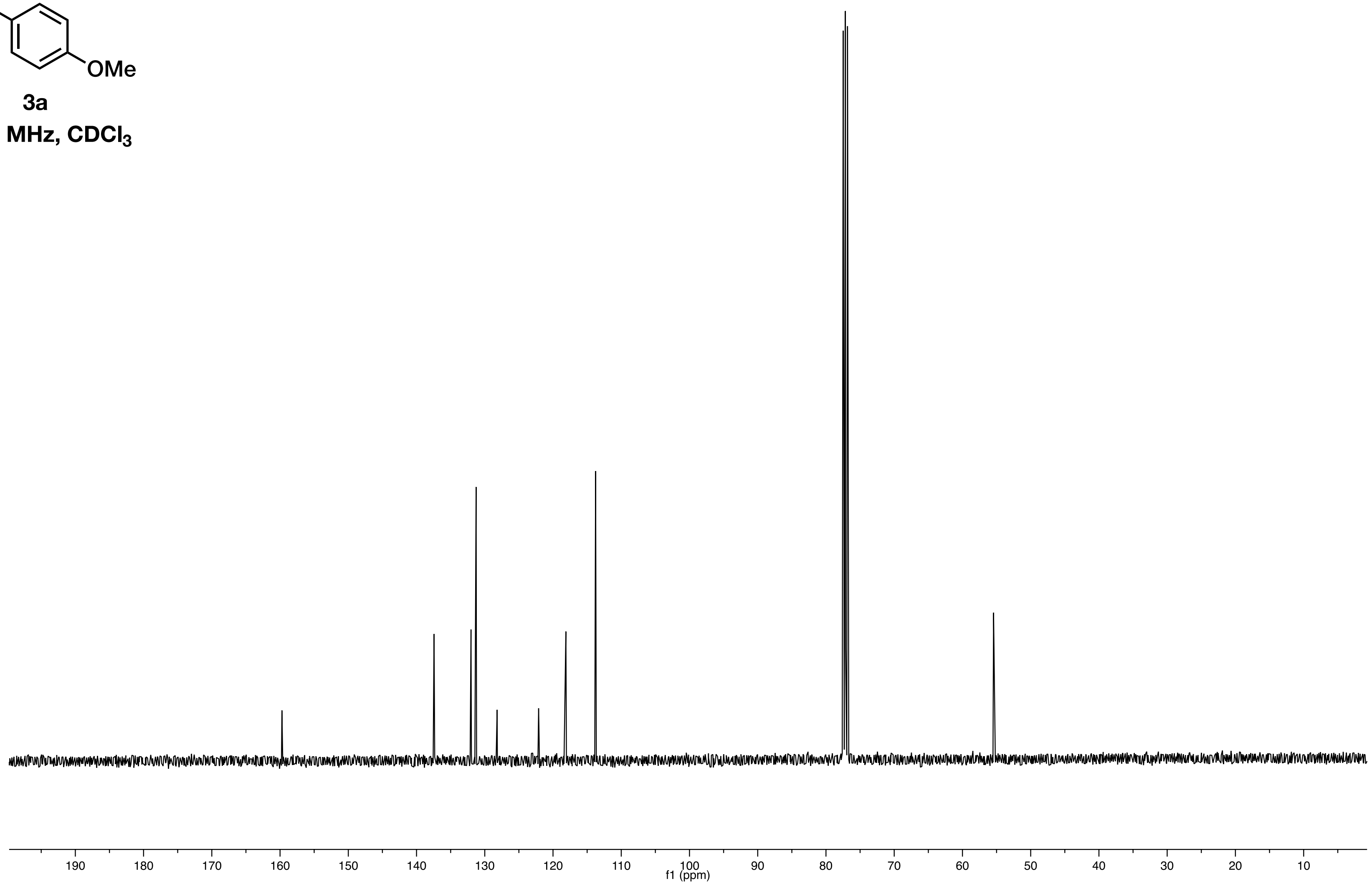

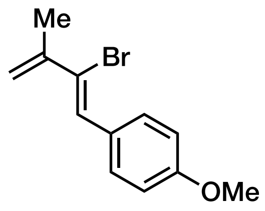

3b

400 MHz, $CDCl_3$

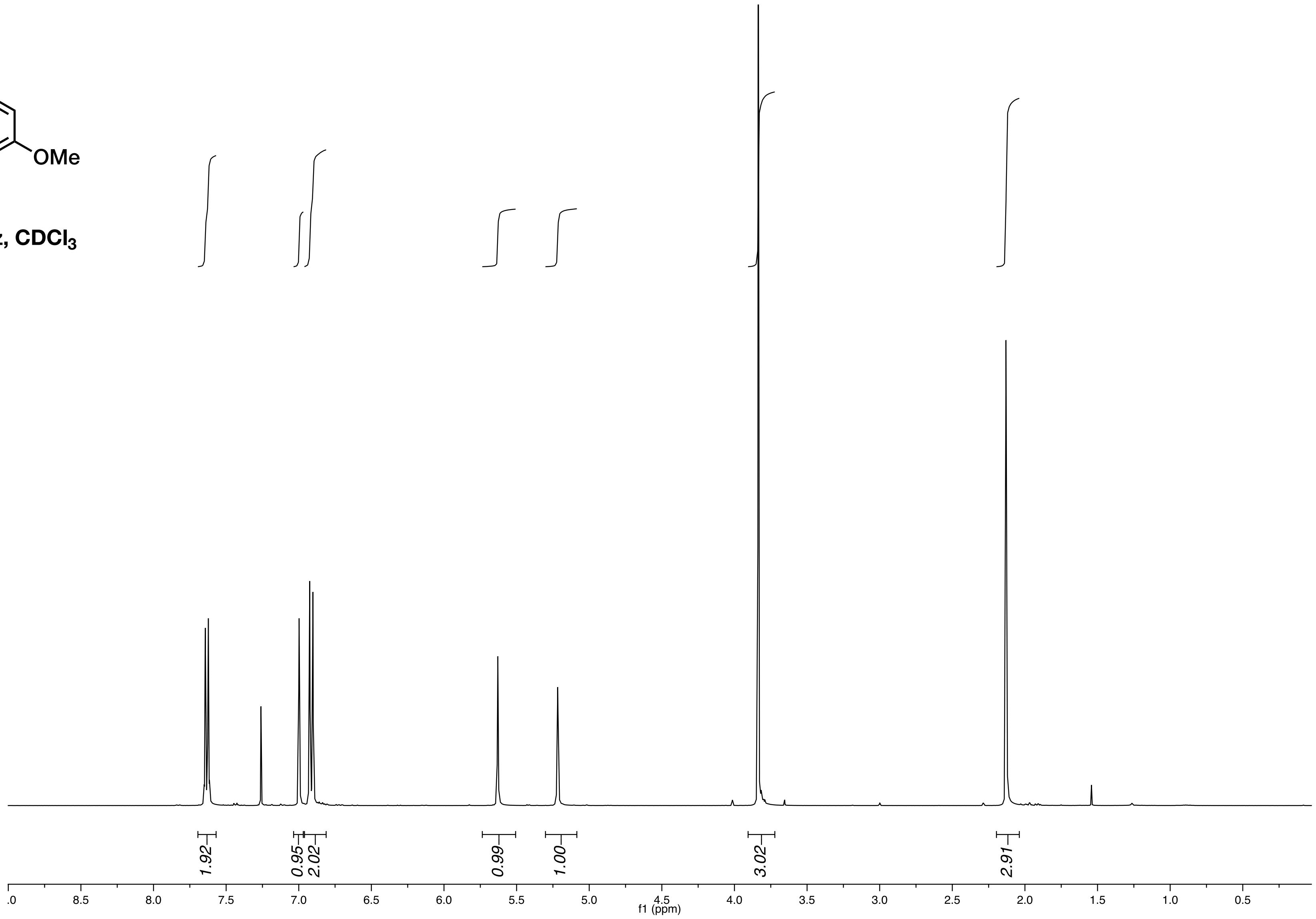

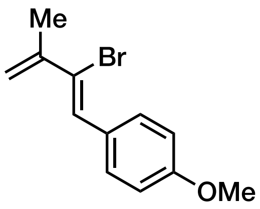

3b

100 MHz, $CDCl_3$

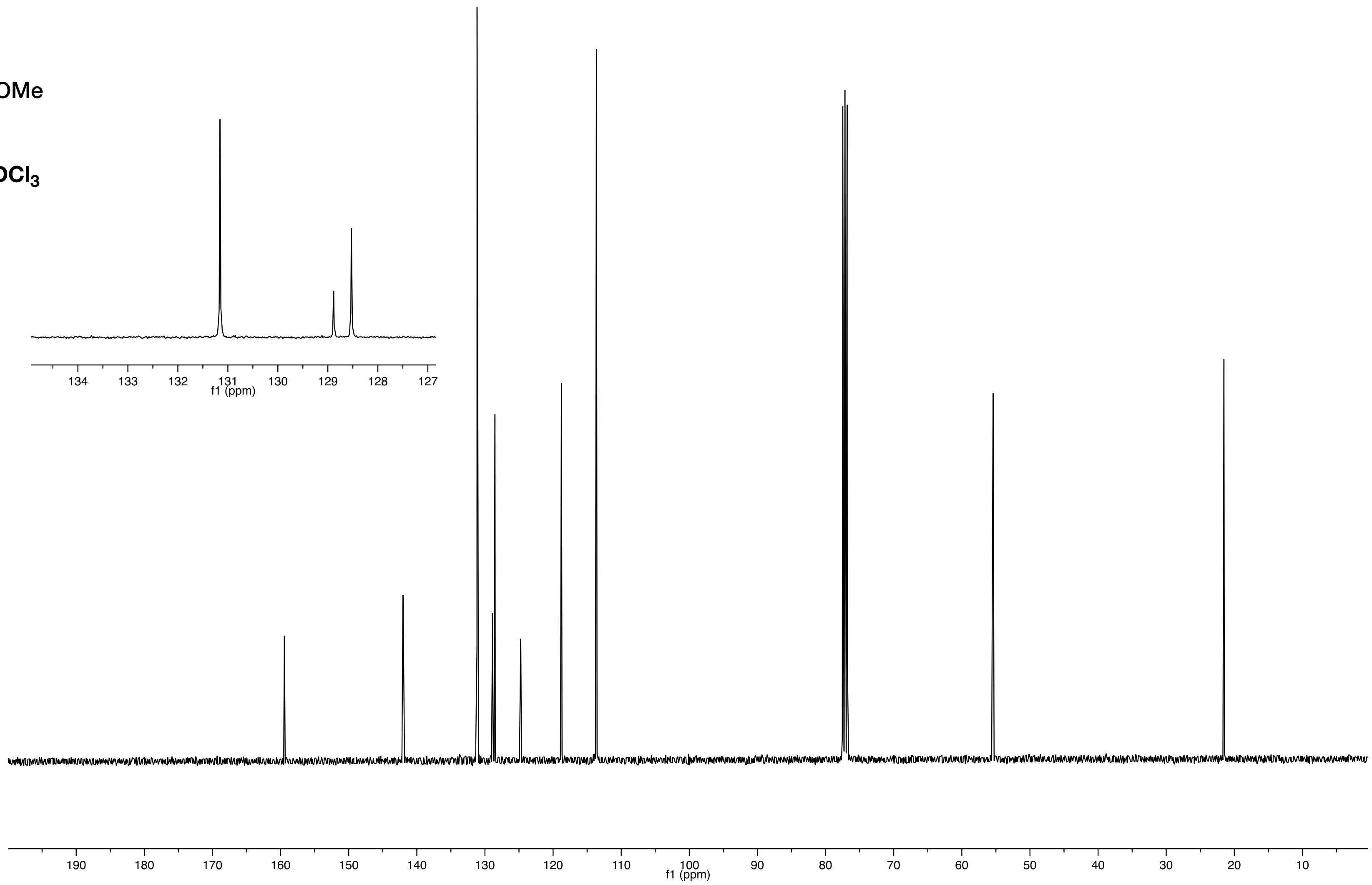

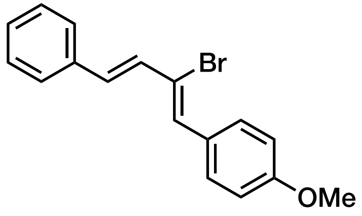

3c

400 MHz, $(CD_3)_2SO$

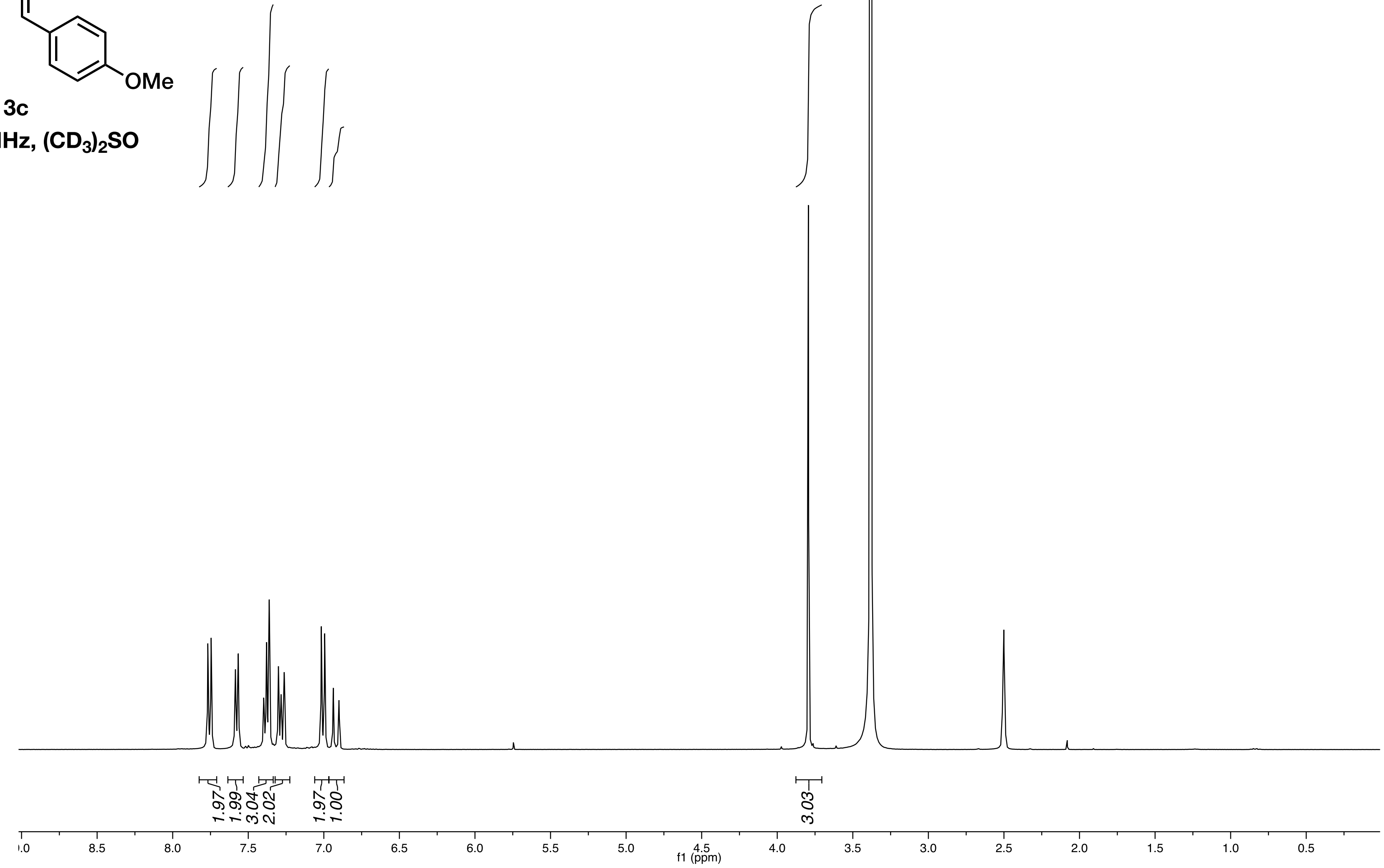

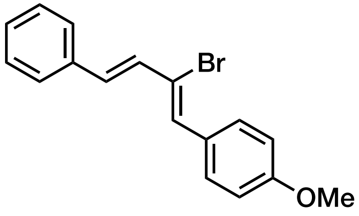

3c
100 MHz, $(CD_3)_2SO$

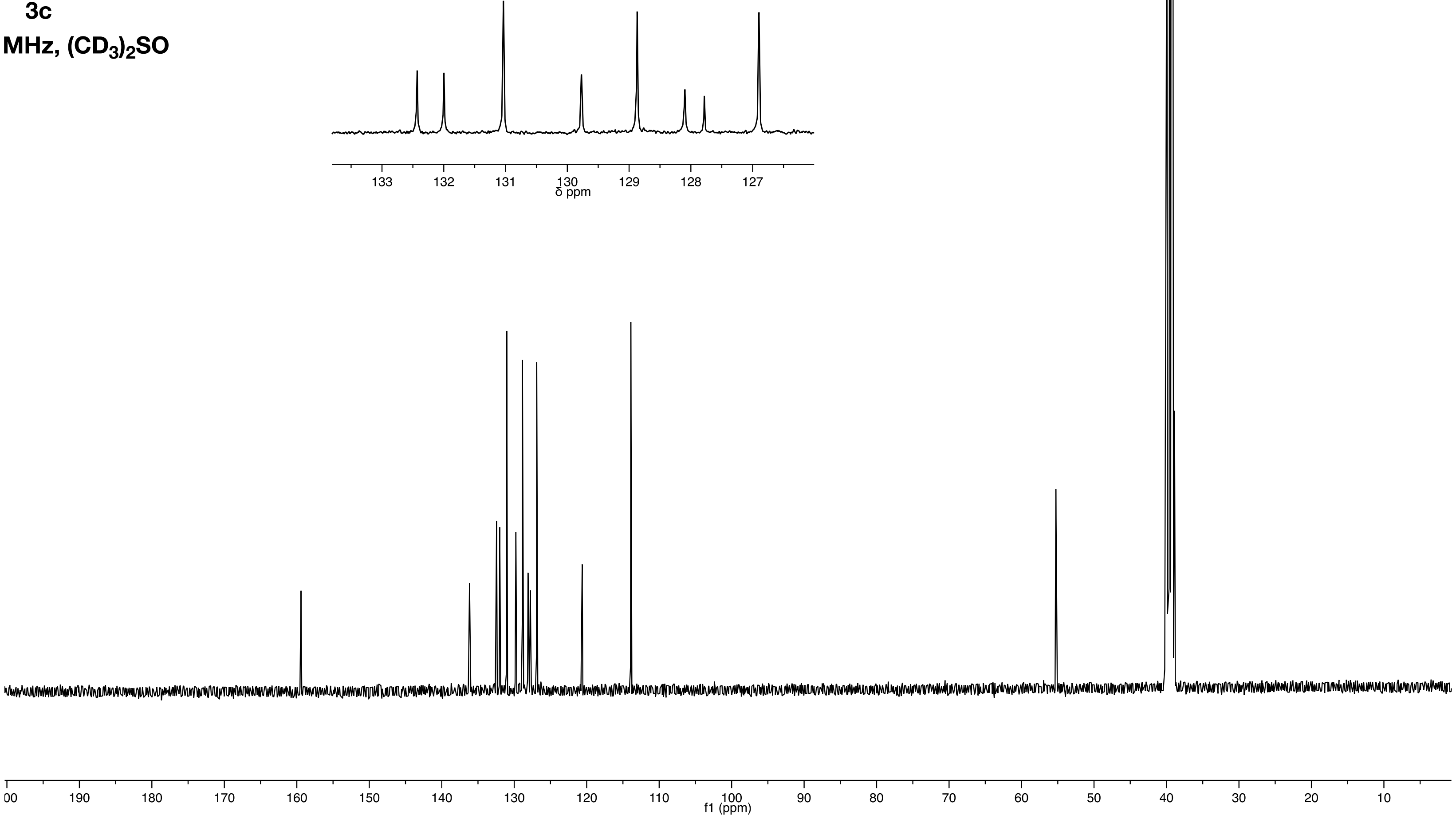

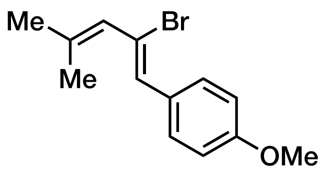

3d

400 MHz, $CDCl_3$

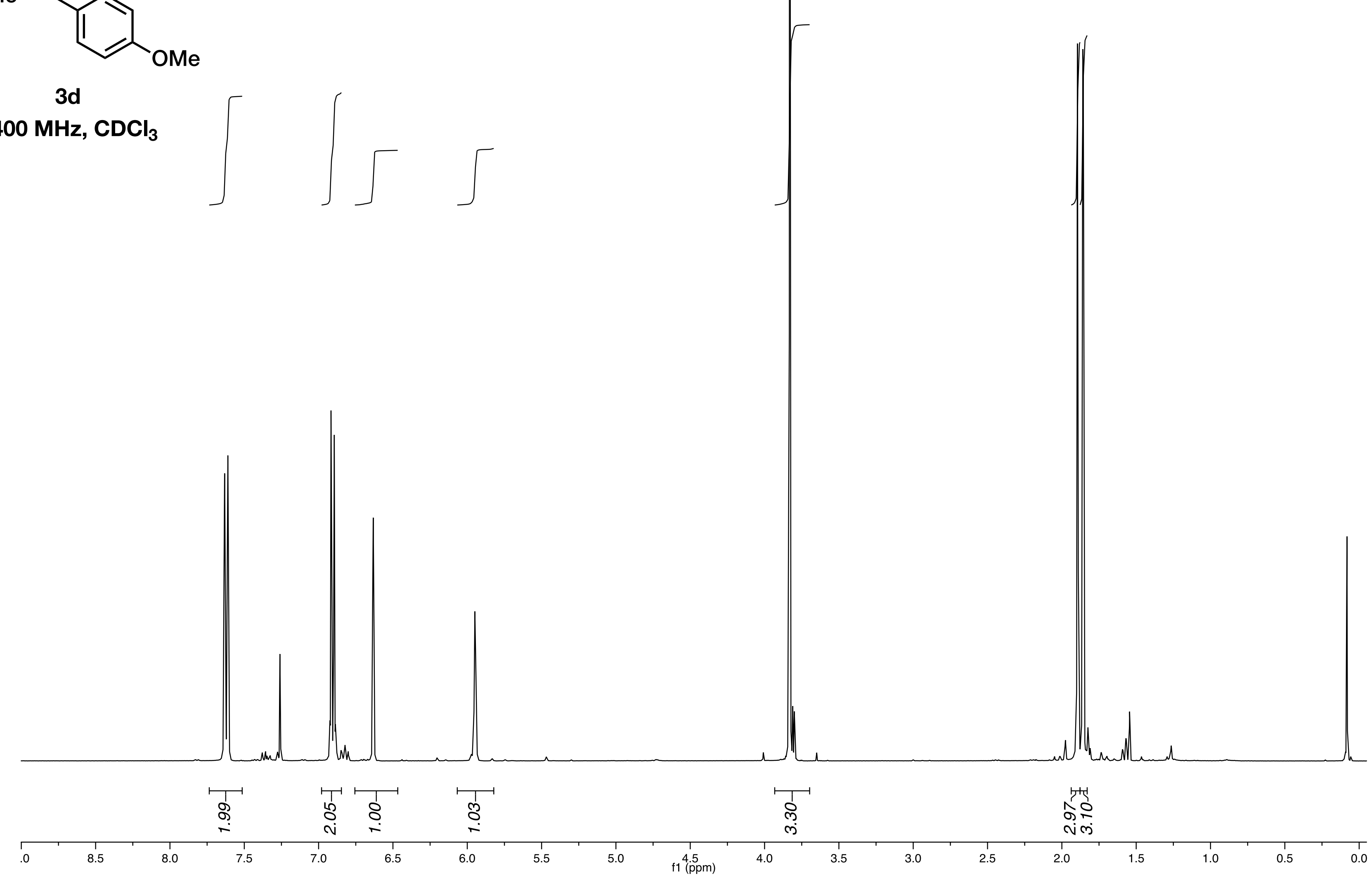

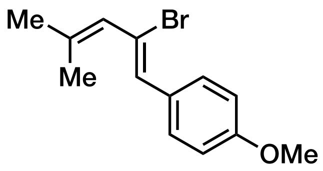

3d

100 MHz, $CDCl_3$

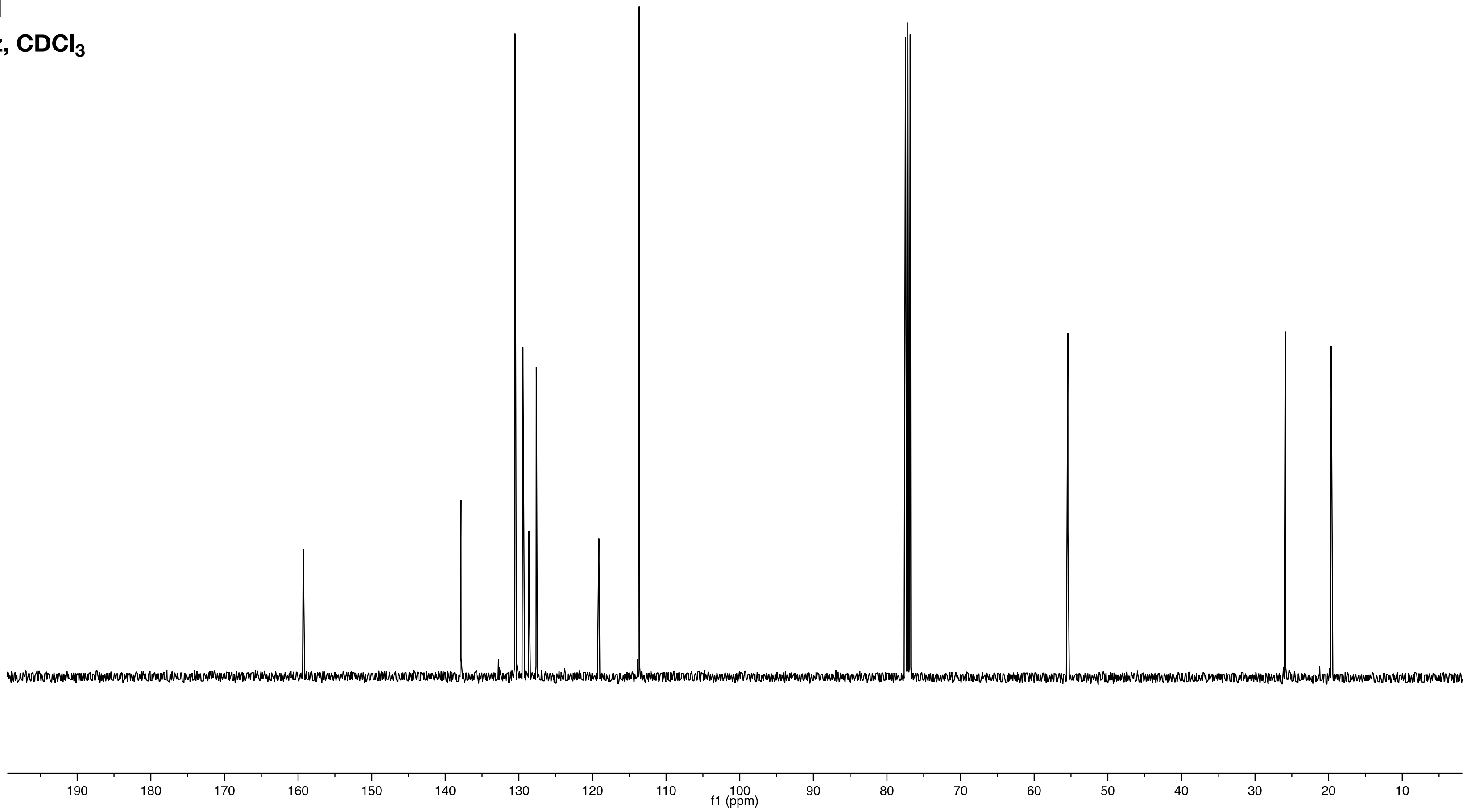

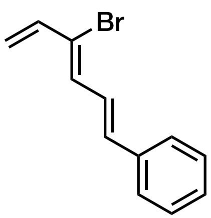

3e
400 MHz, $CDCl_3$

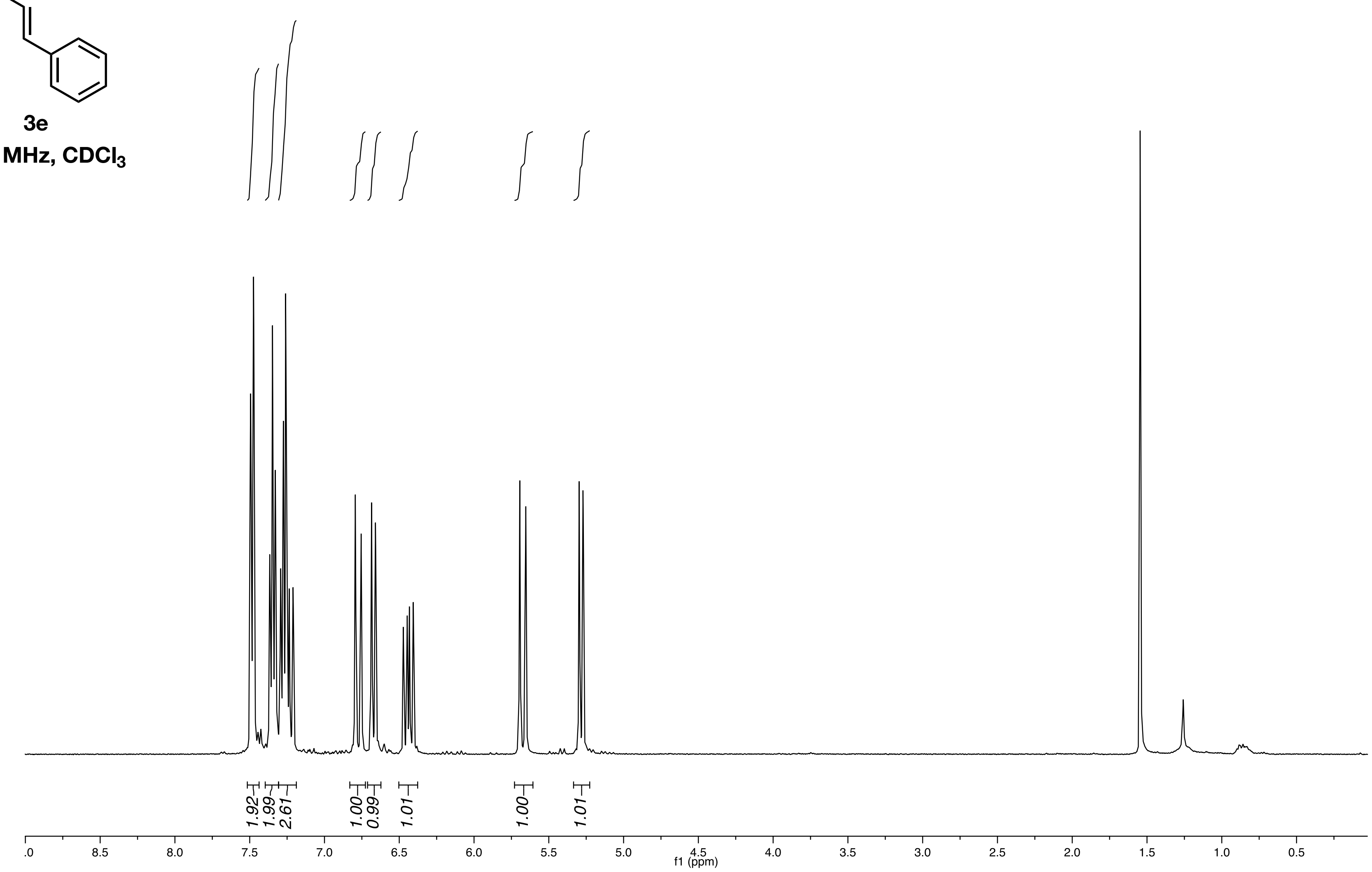

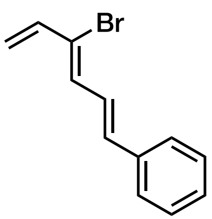

3e

100 MHz, $CDCl_3$

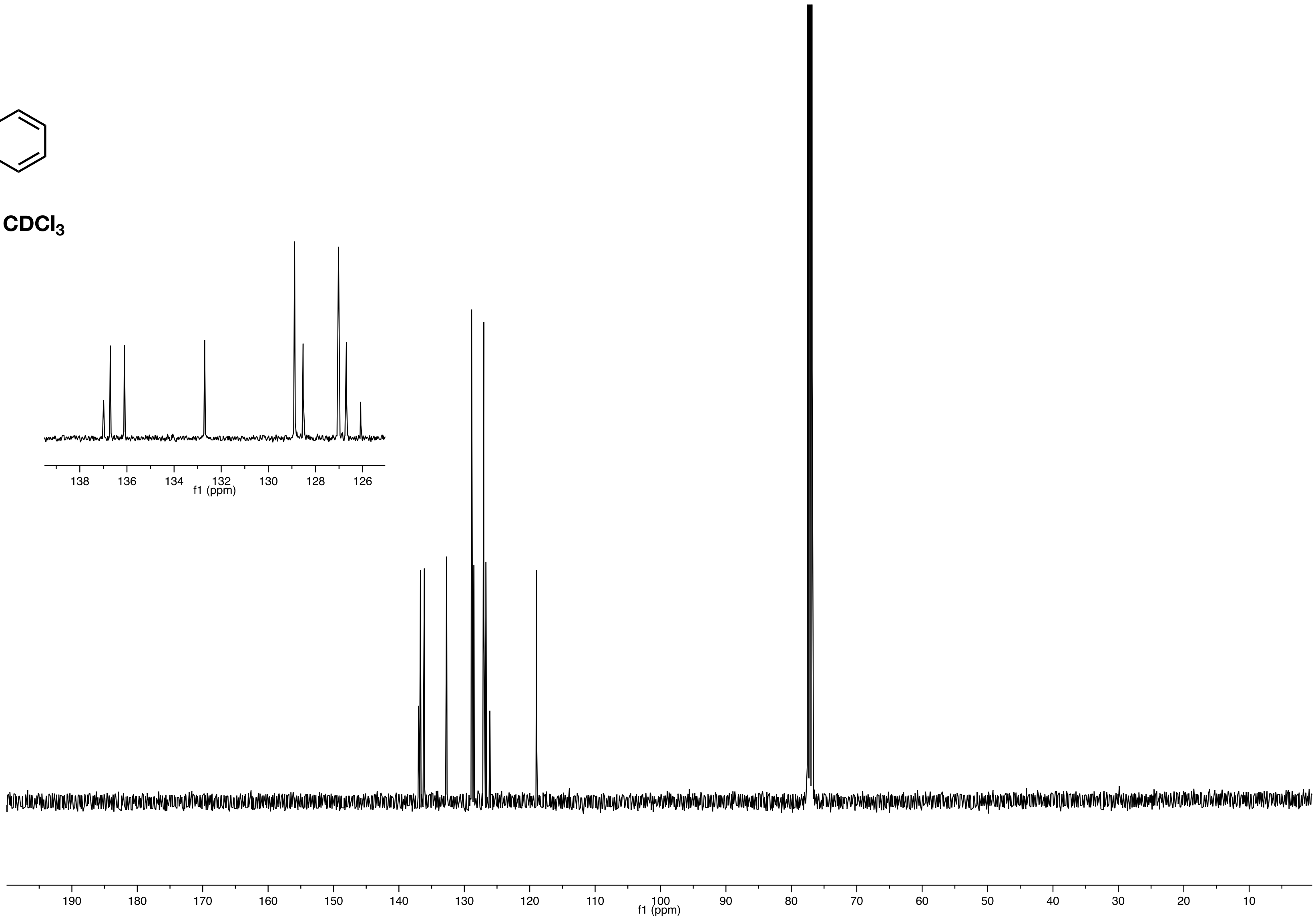

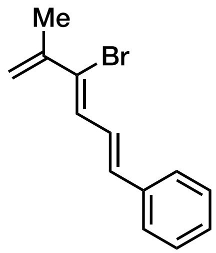

3f

400 MHz, $CDCl_3$

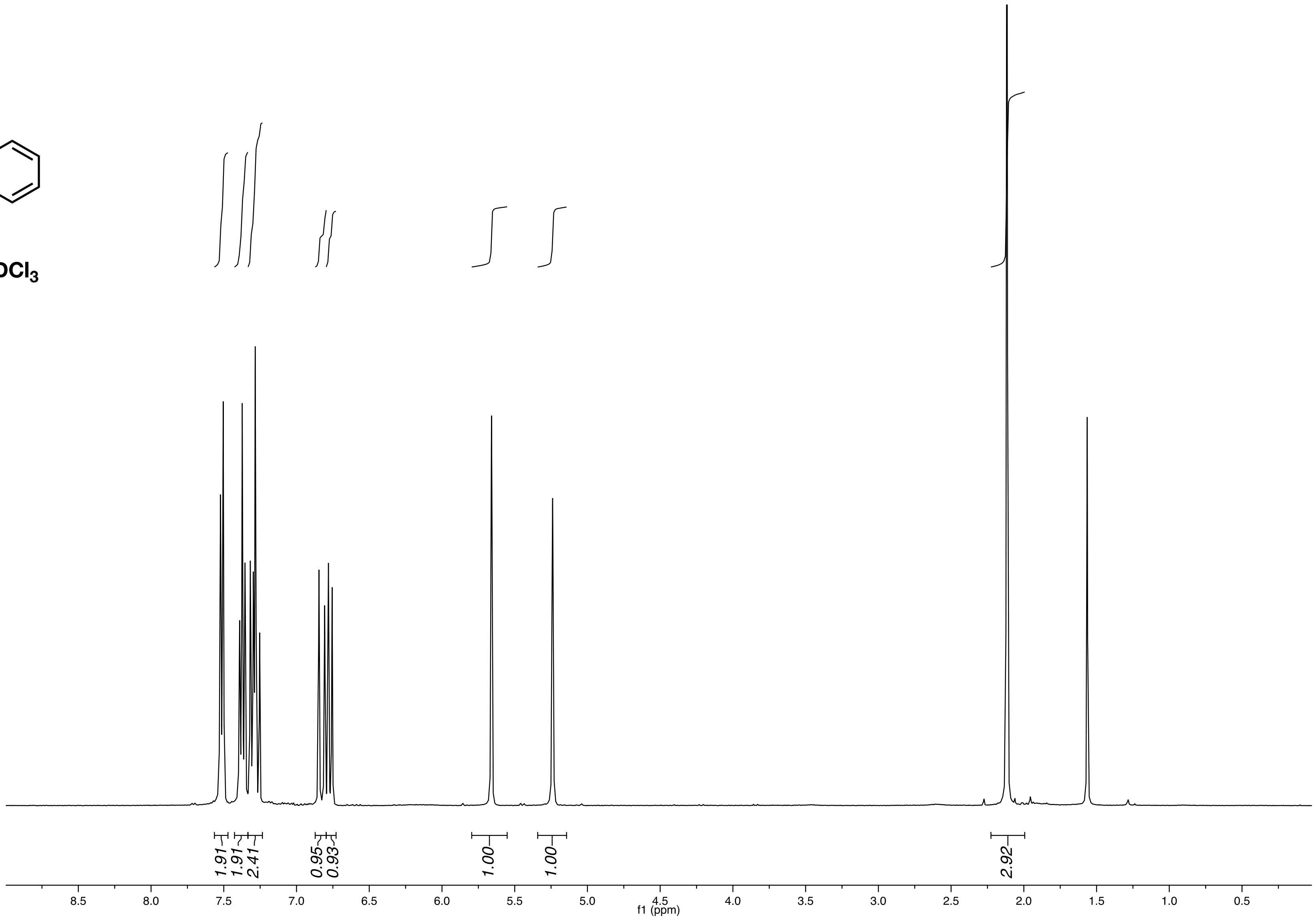

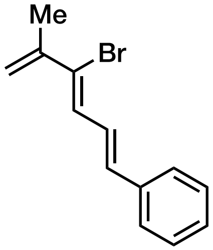

3f

100 MHz, $CDCl_3$

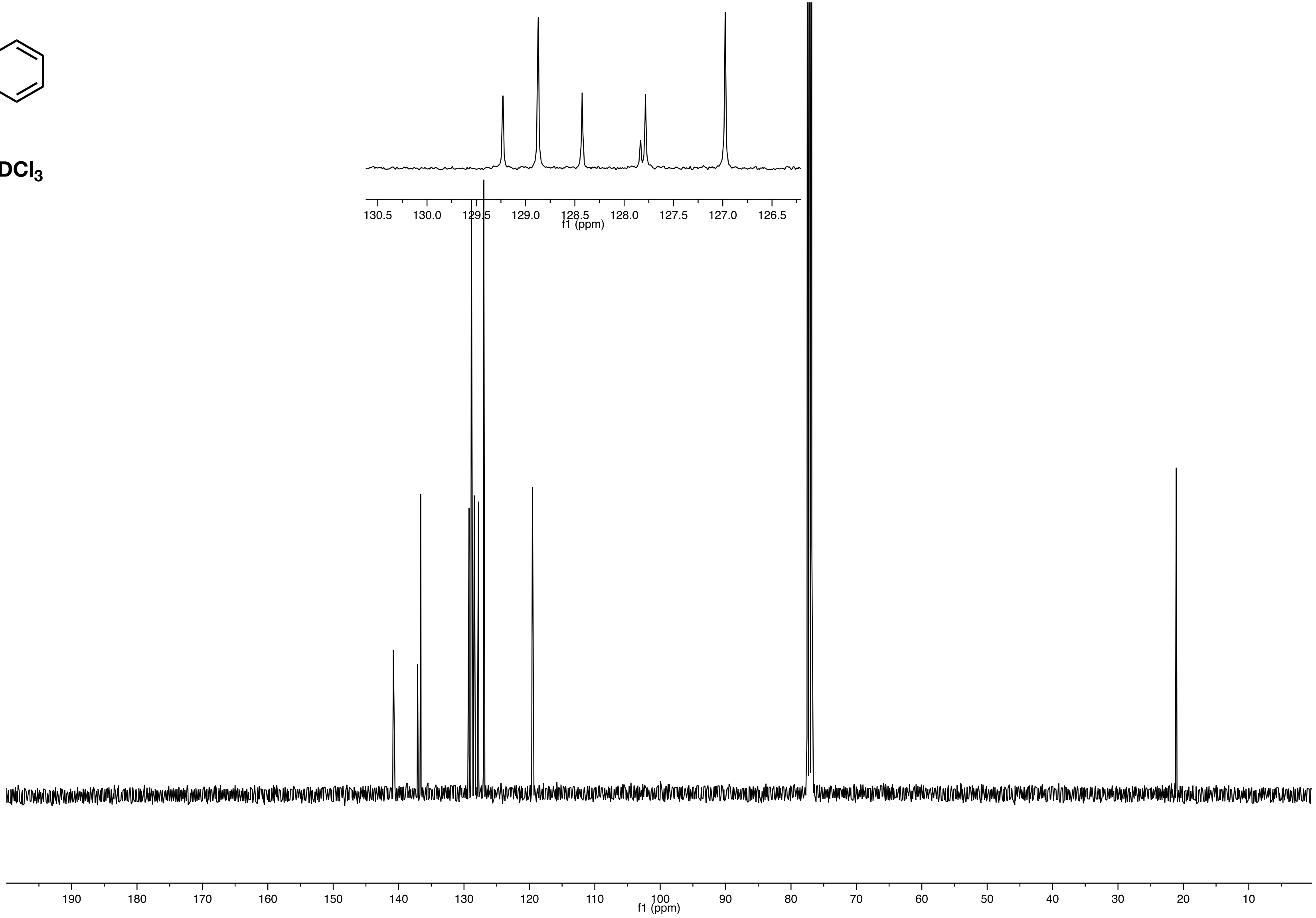

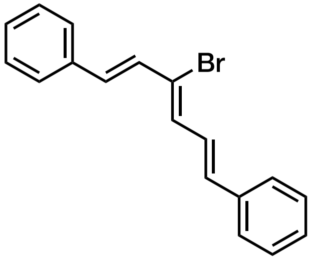

3g
400 MHz, $CDCl_3$

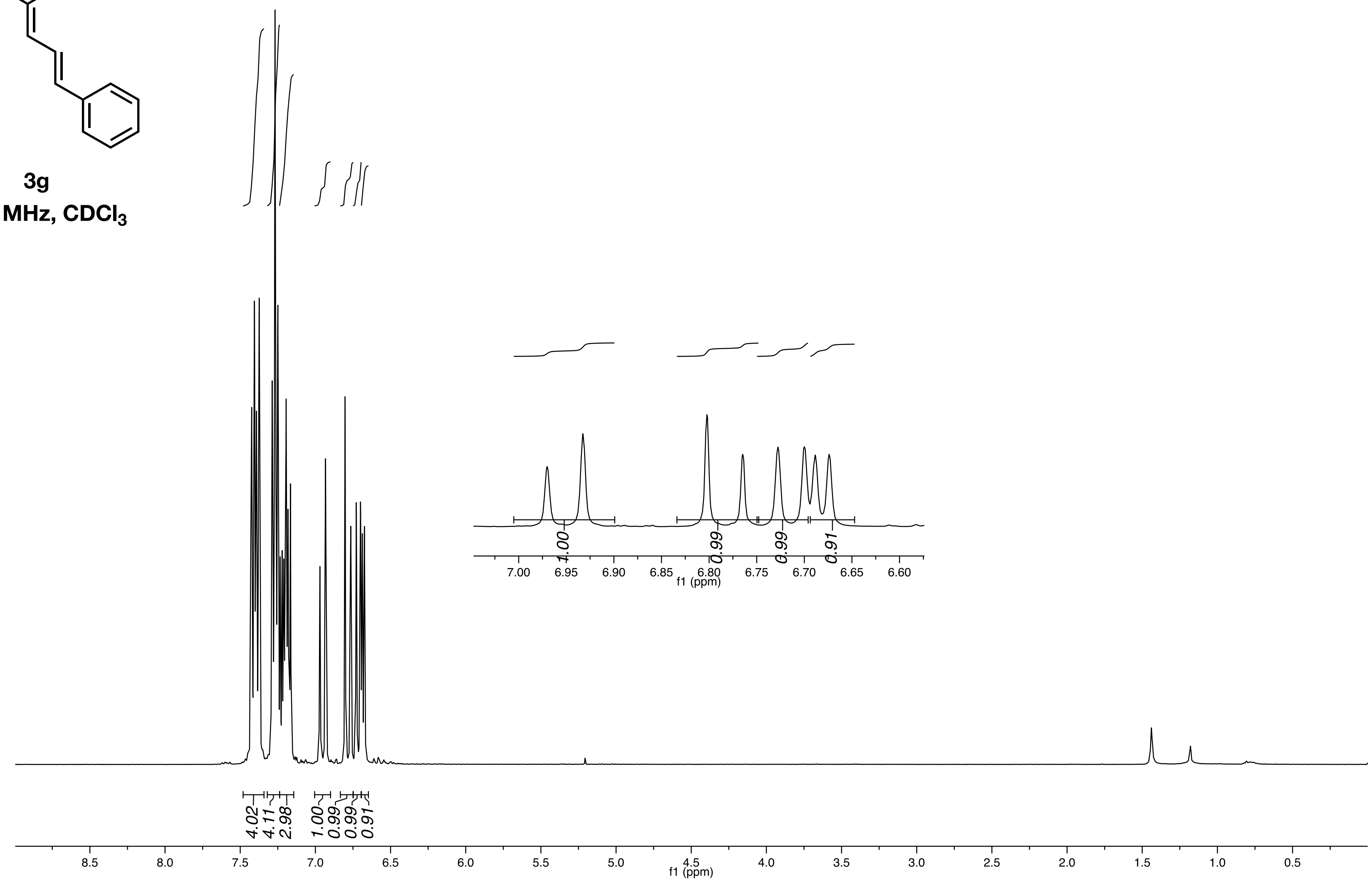

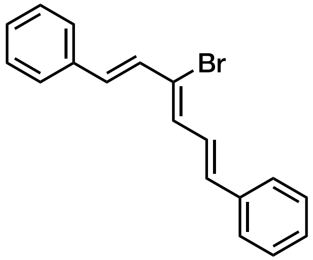

3g

100 MHz, $CDCl_3$

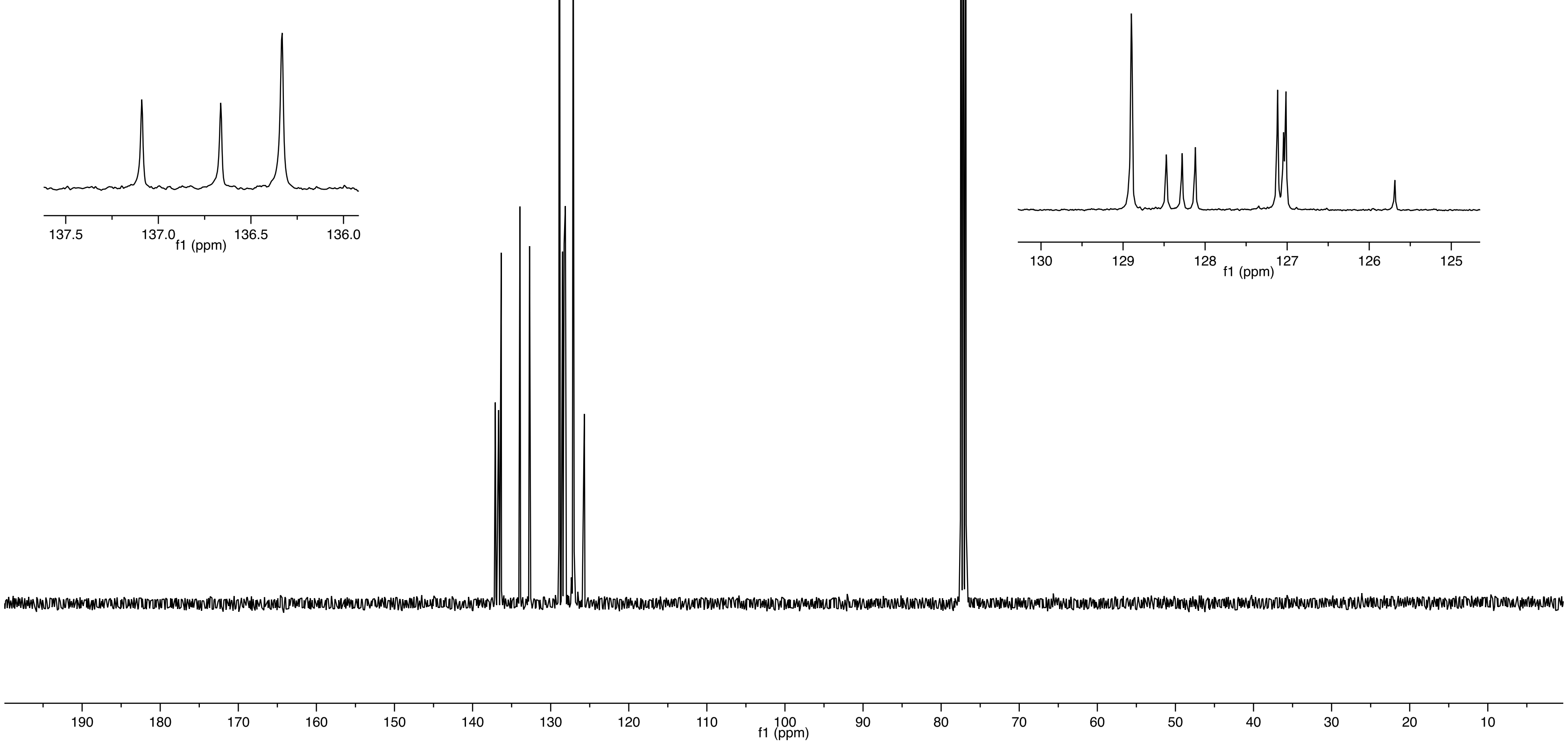

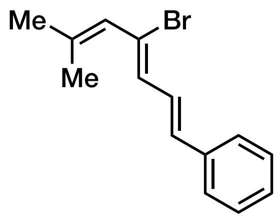

3h

400 MHz, $CDCl_3$

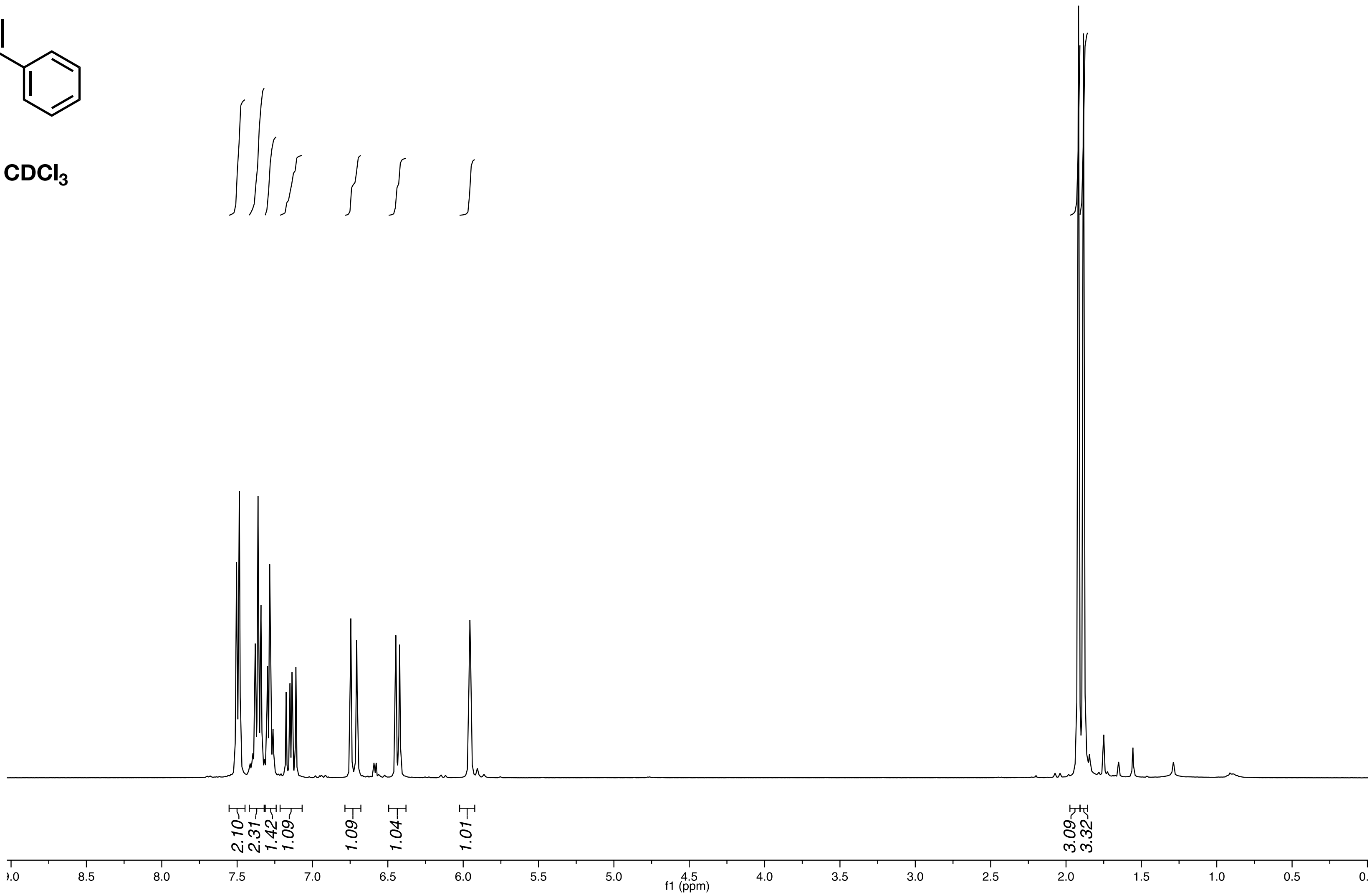

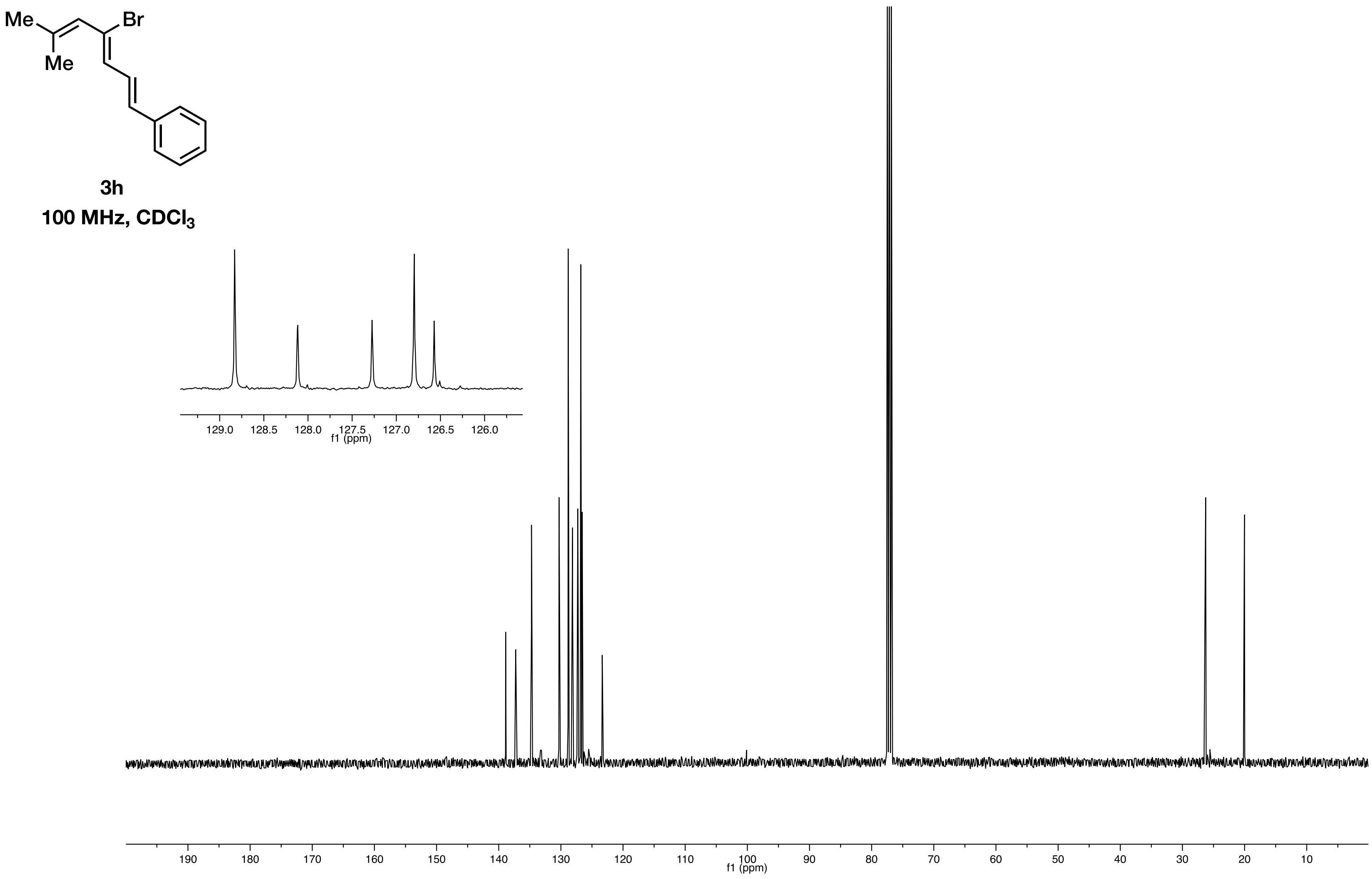
Me
Br
Me
3h
100 MHz, CDCl3
129.0
128.5
128.0
127.5
127.0
126.5
126.0
f1 (ppm)
190
180
170
160
150
140
130
120
110
100
90
80
70
60
50
40
30
20
10
f1 (ppm)

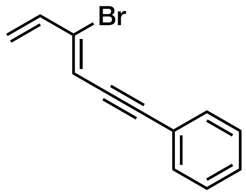

3i

400 MHz, $CDCl_3$

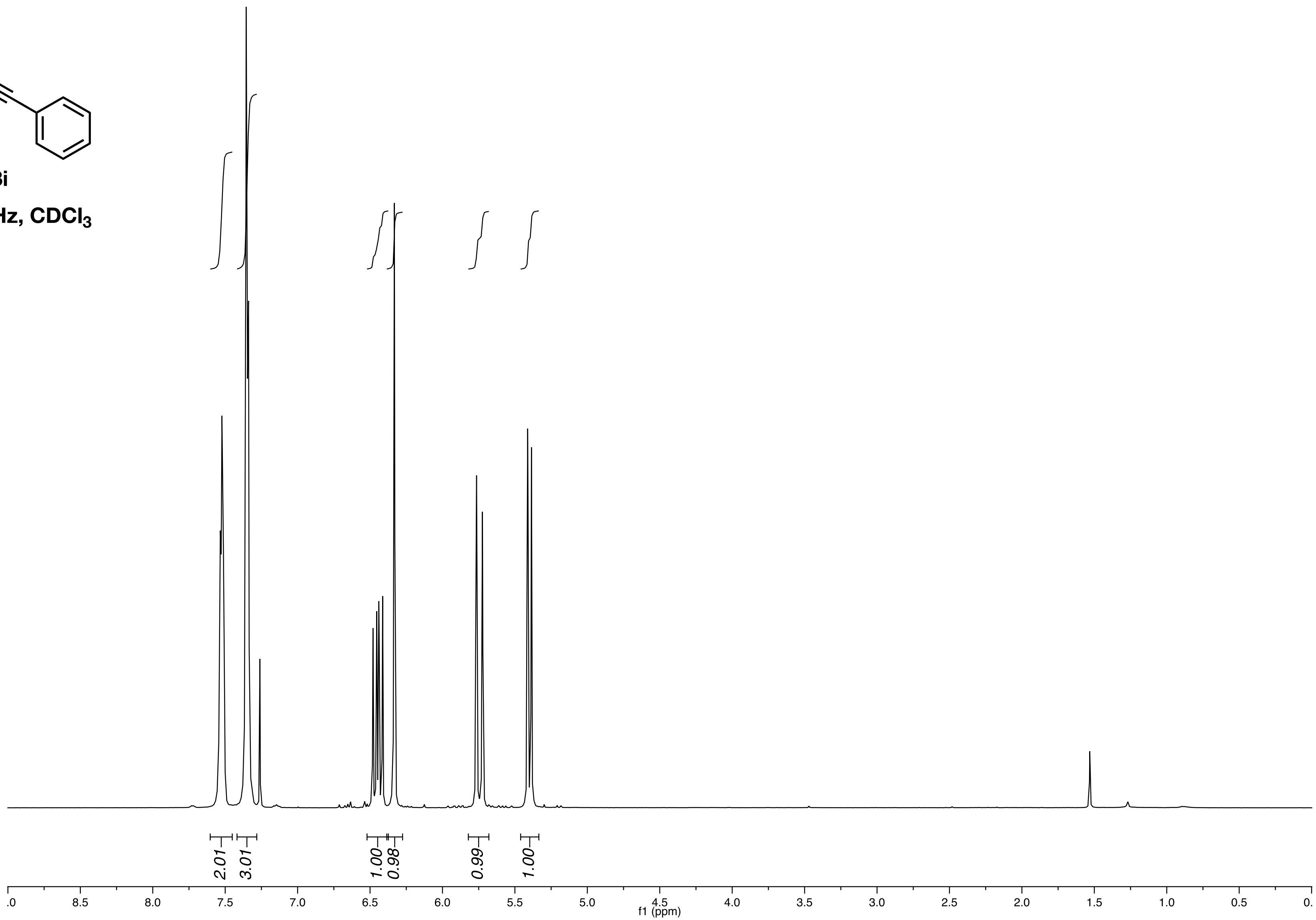

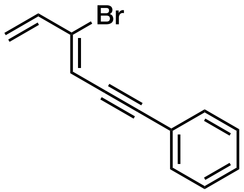

3i

100 MHz, $CDCl_3$

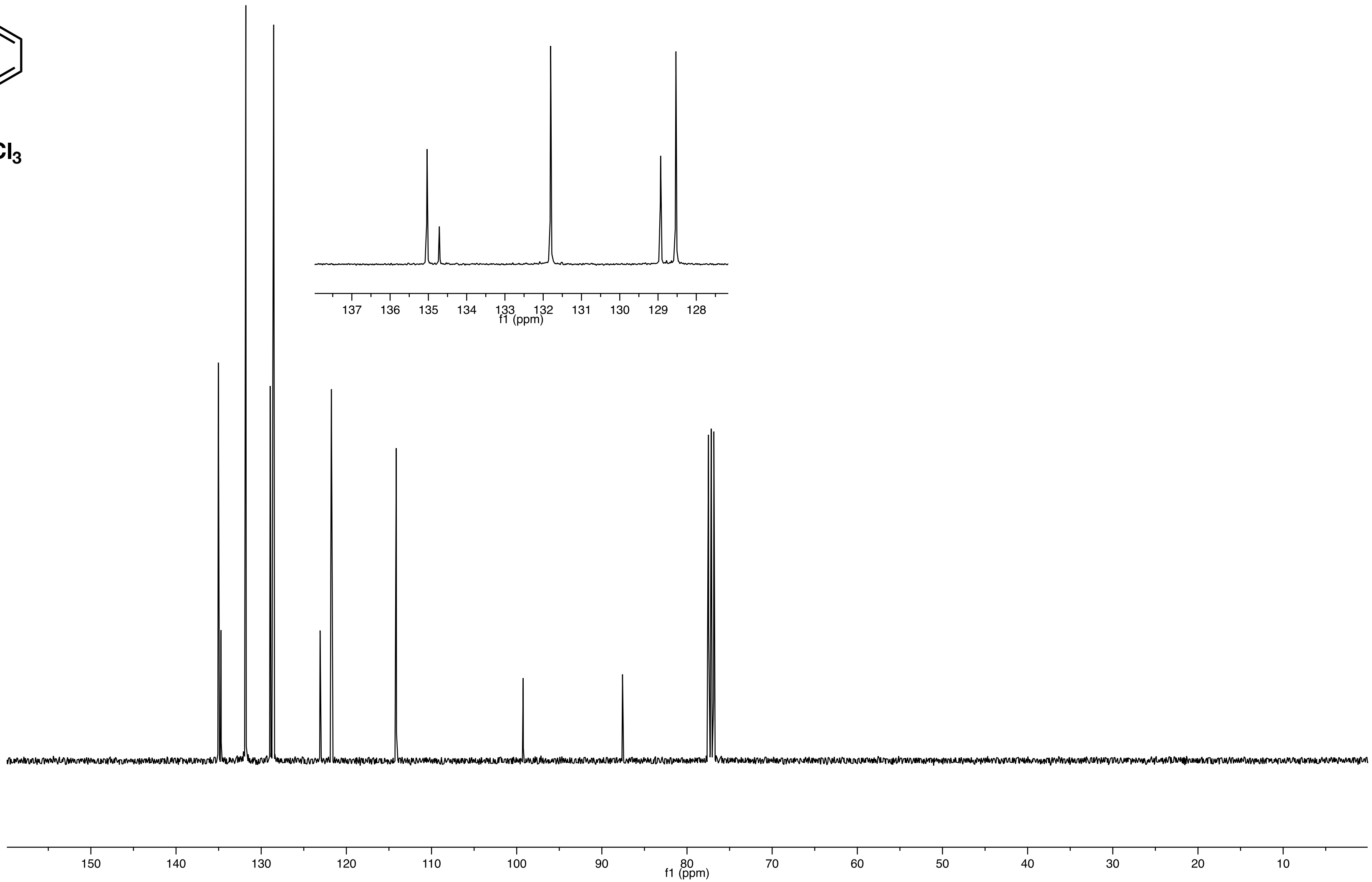

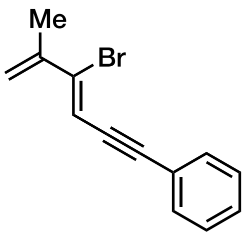

3j
400 MHz, $CDCl_3$

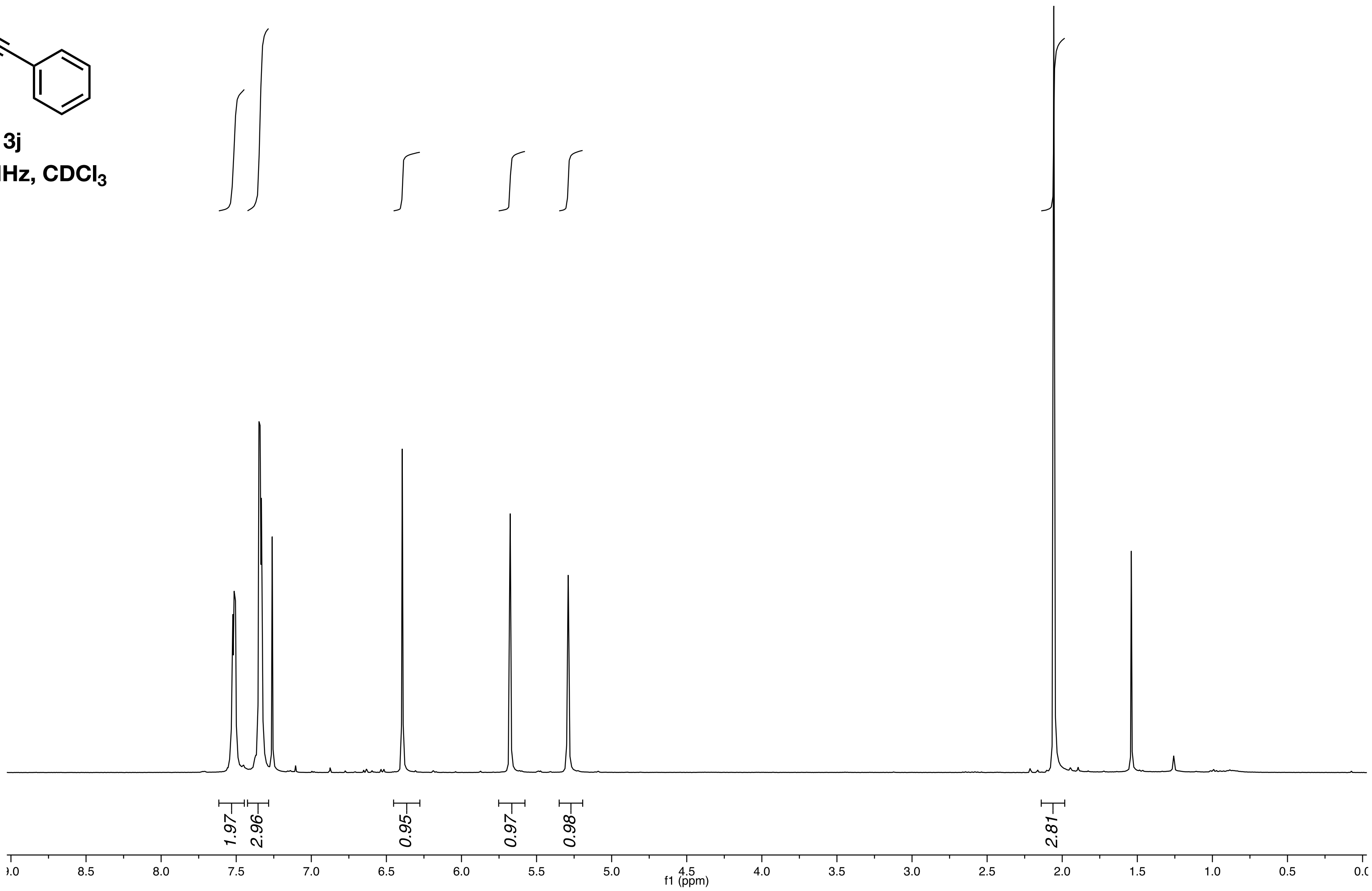

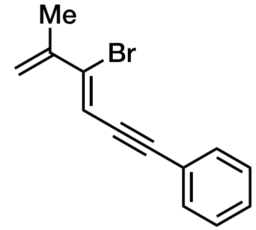

3j
100 MHz, $CDCl_3$

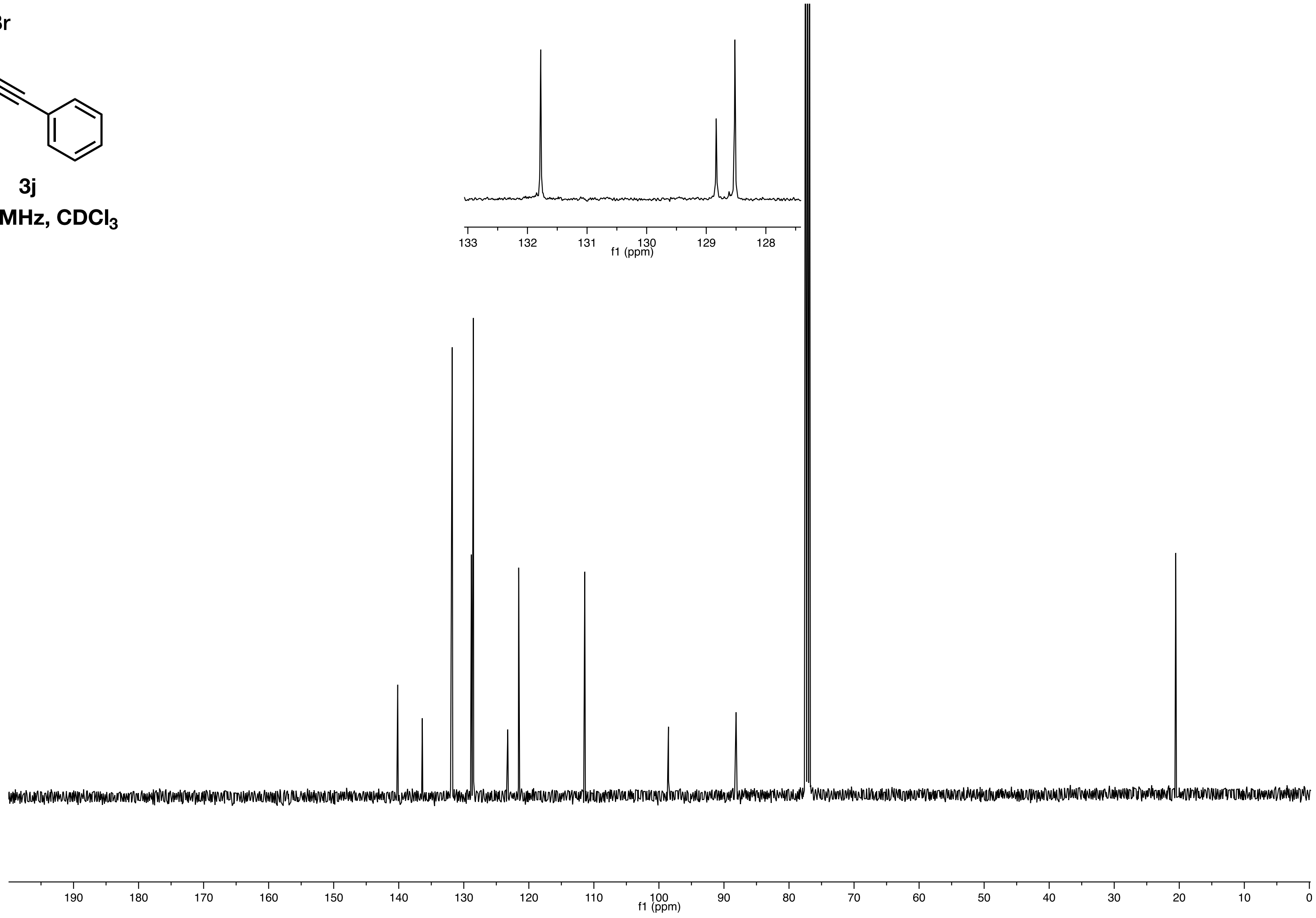

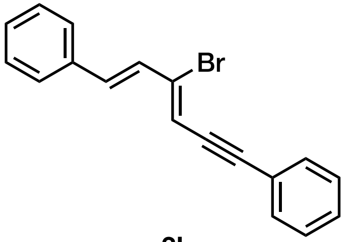

3k

400 MHz, $CDCl_3$

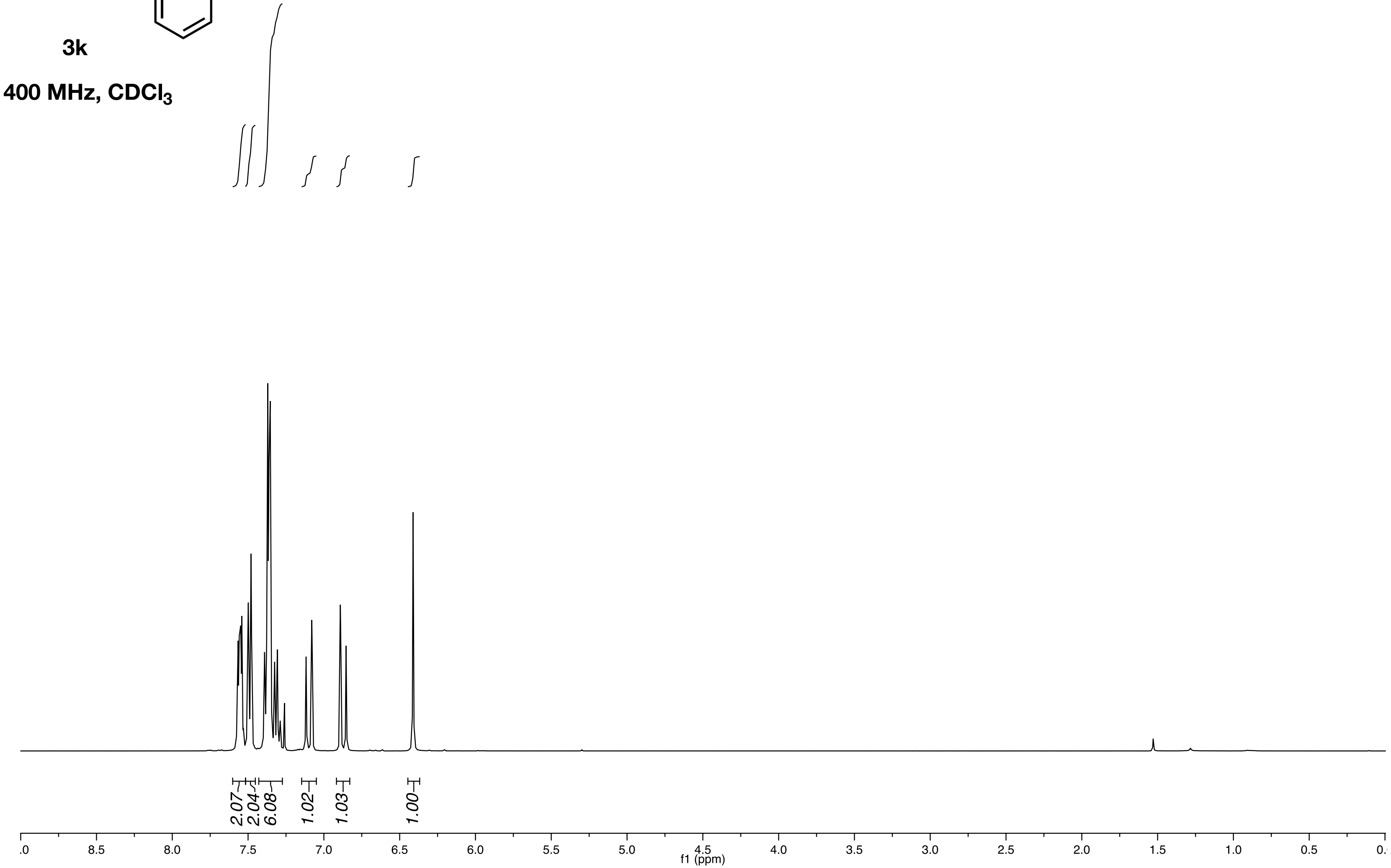

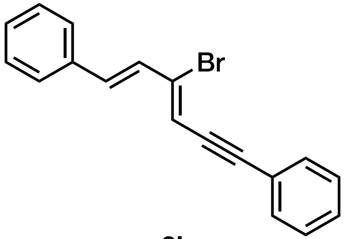

3k

100 MHz, $CDCl_3$

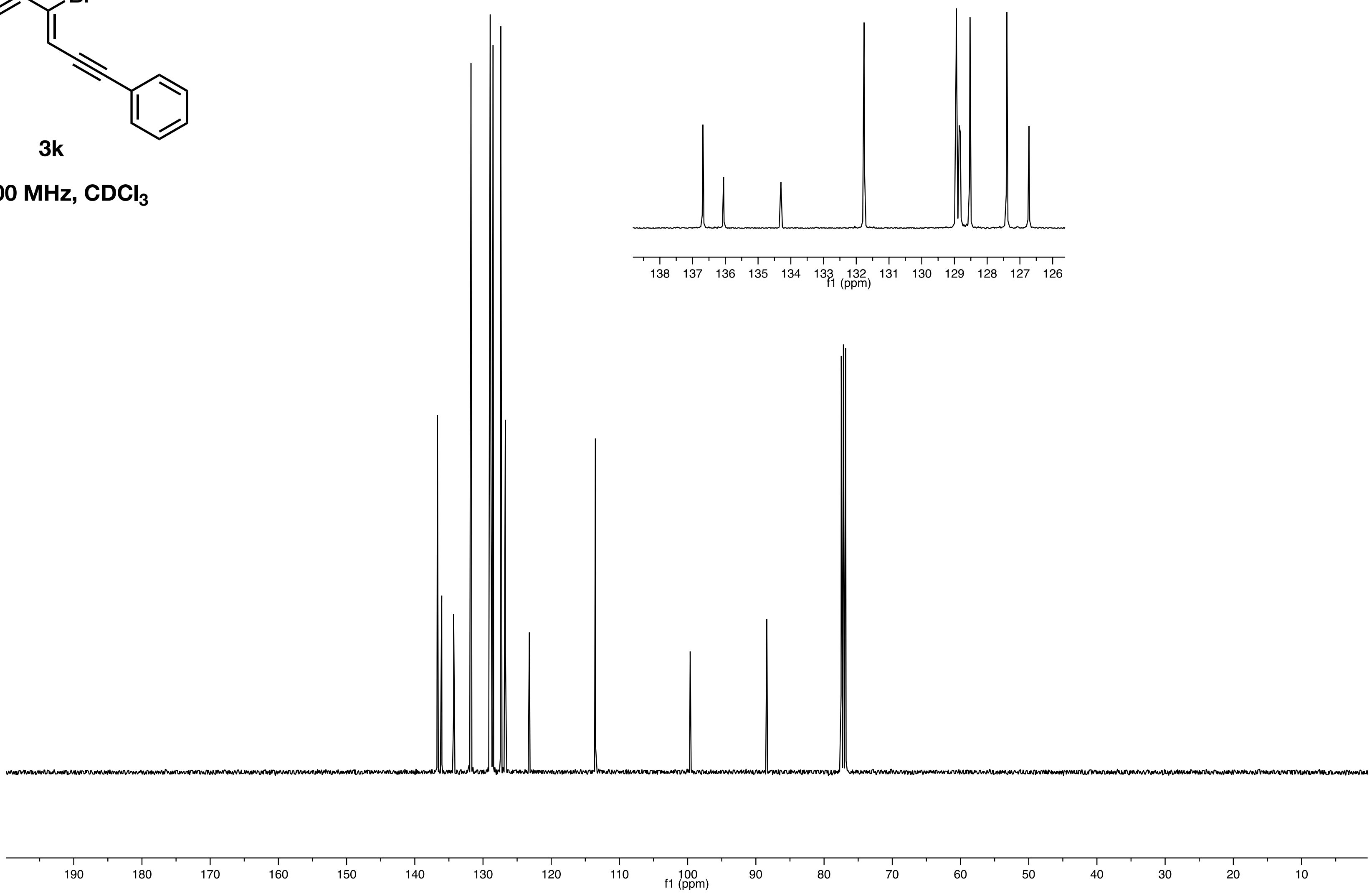

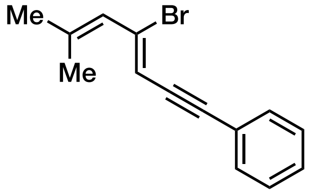

3l
400 MHz, $CDCl_3$

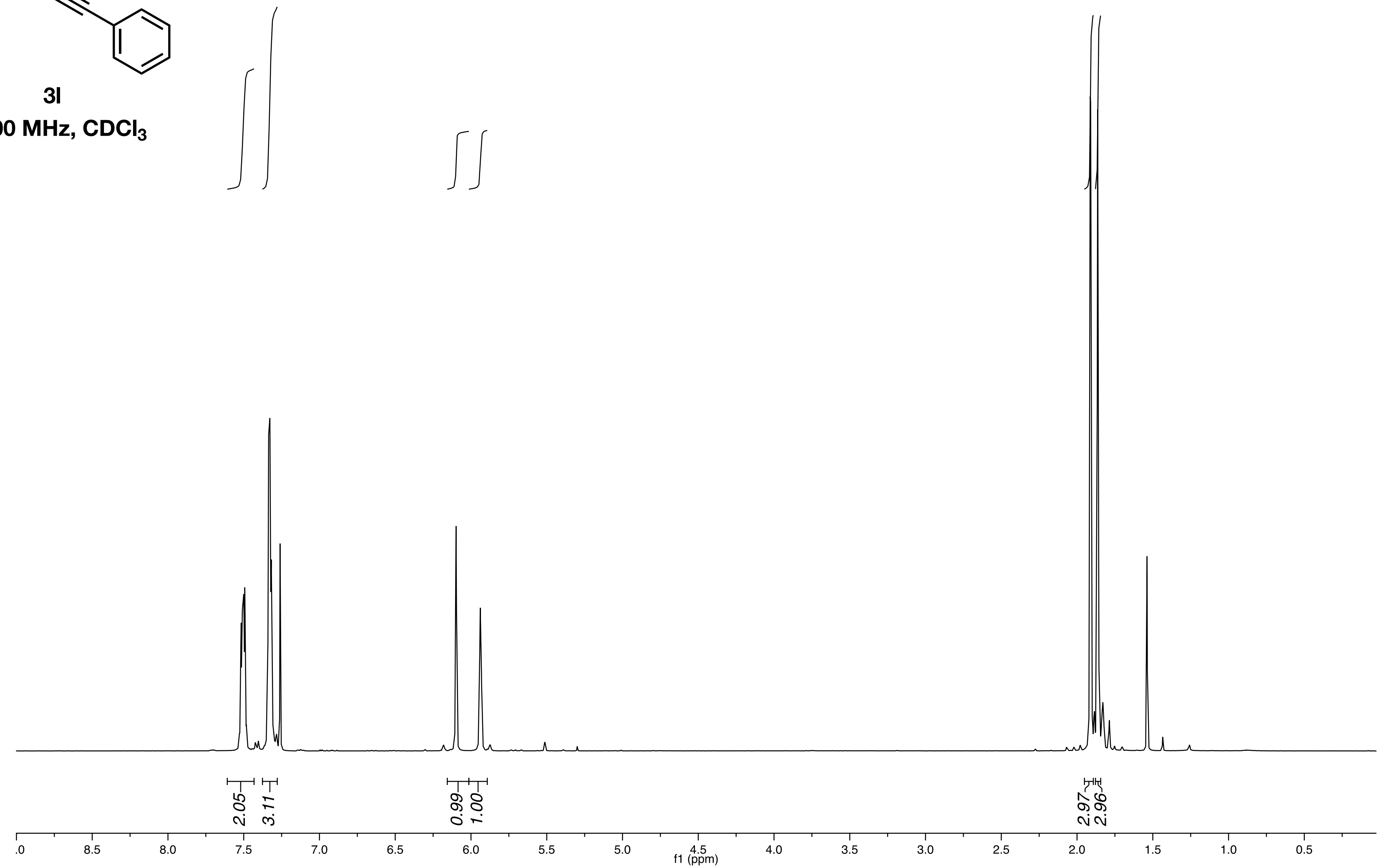

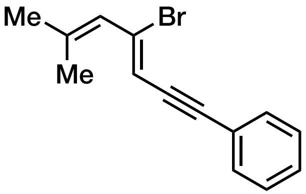

3l

100 MHz, $CDCl_3$

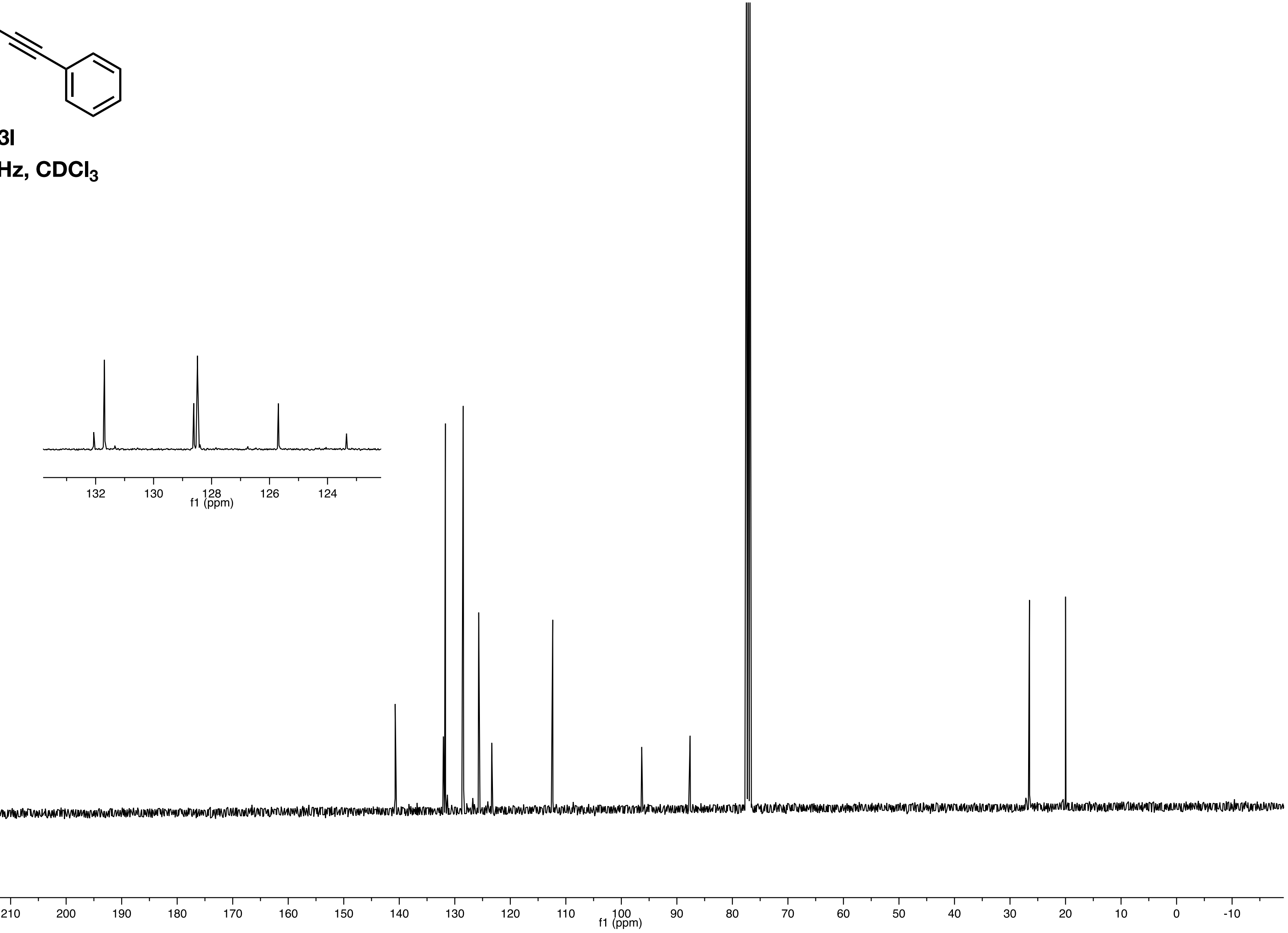

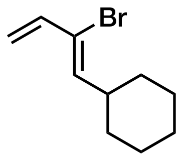

3m

400 MHz, $CDCl_3$

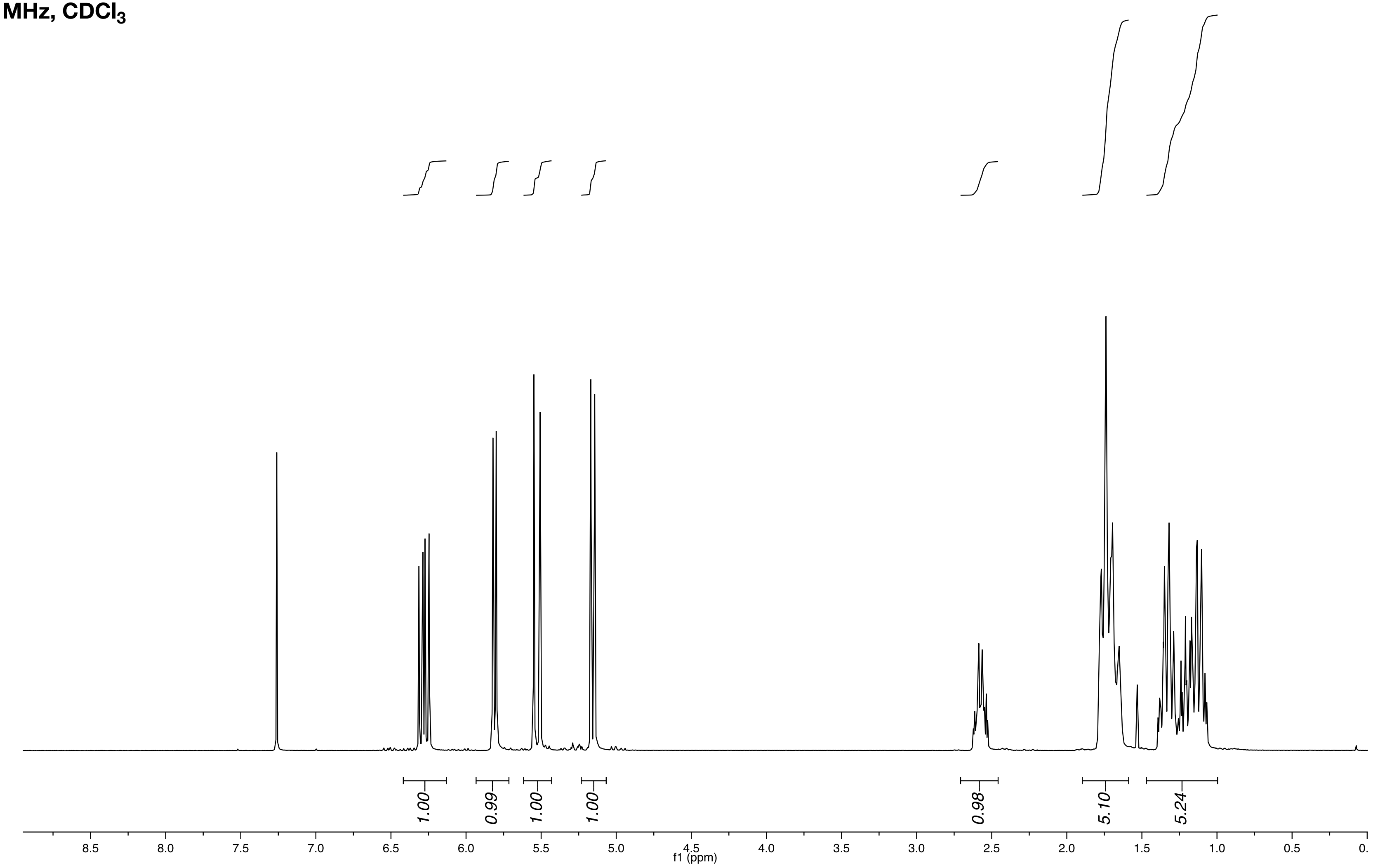

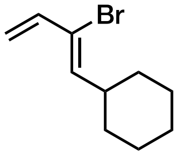

3m
100 MHz, $CDCl_3$

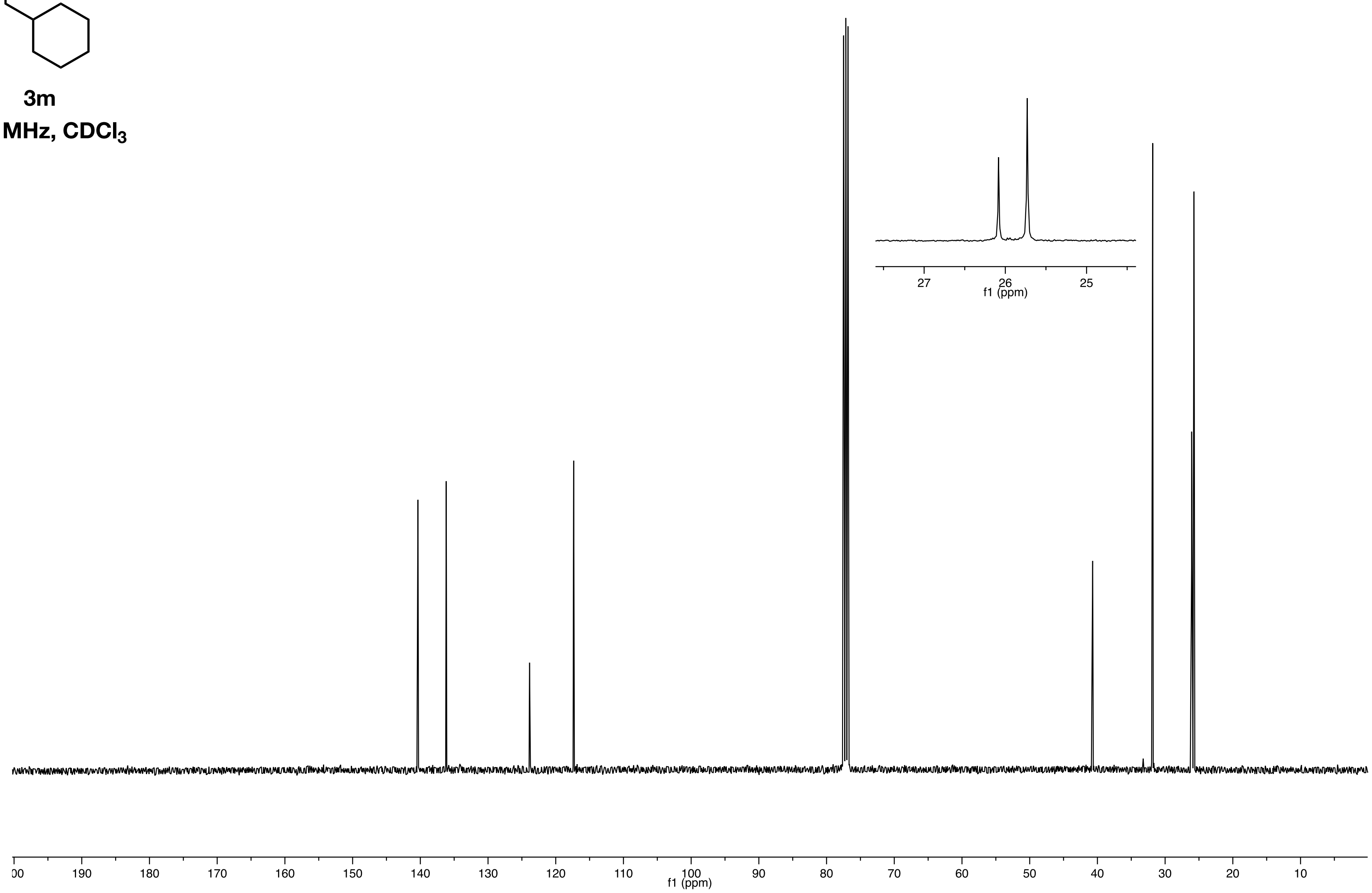

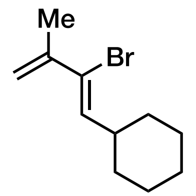

3n

400 MHz, $CDCl_3$

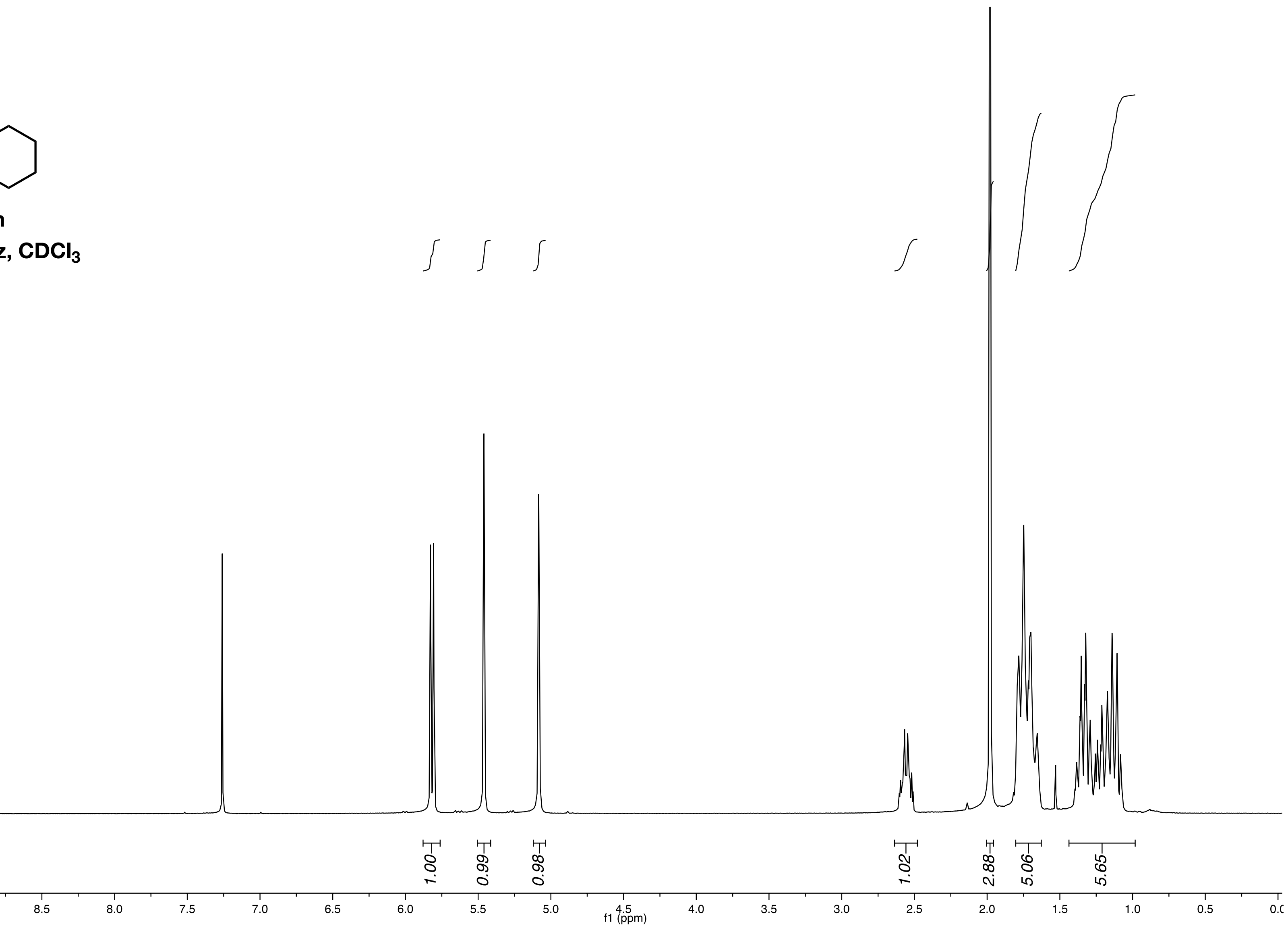

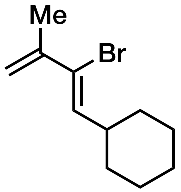

3n

100 MHz, $CDCl_3$

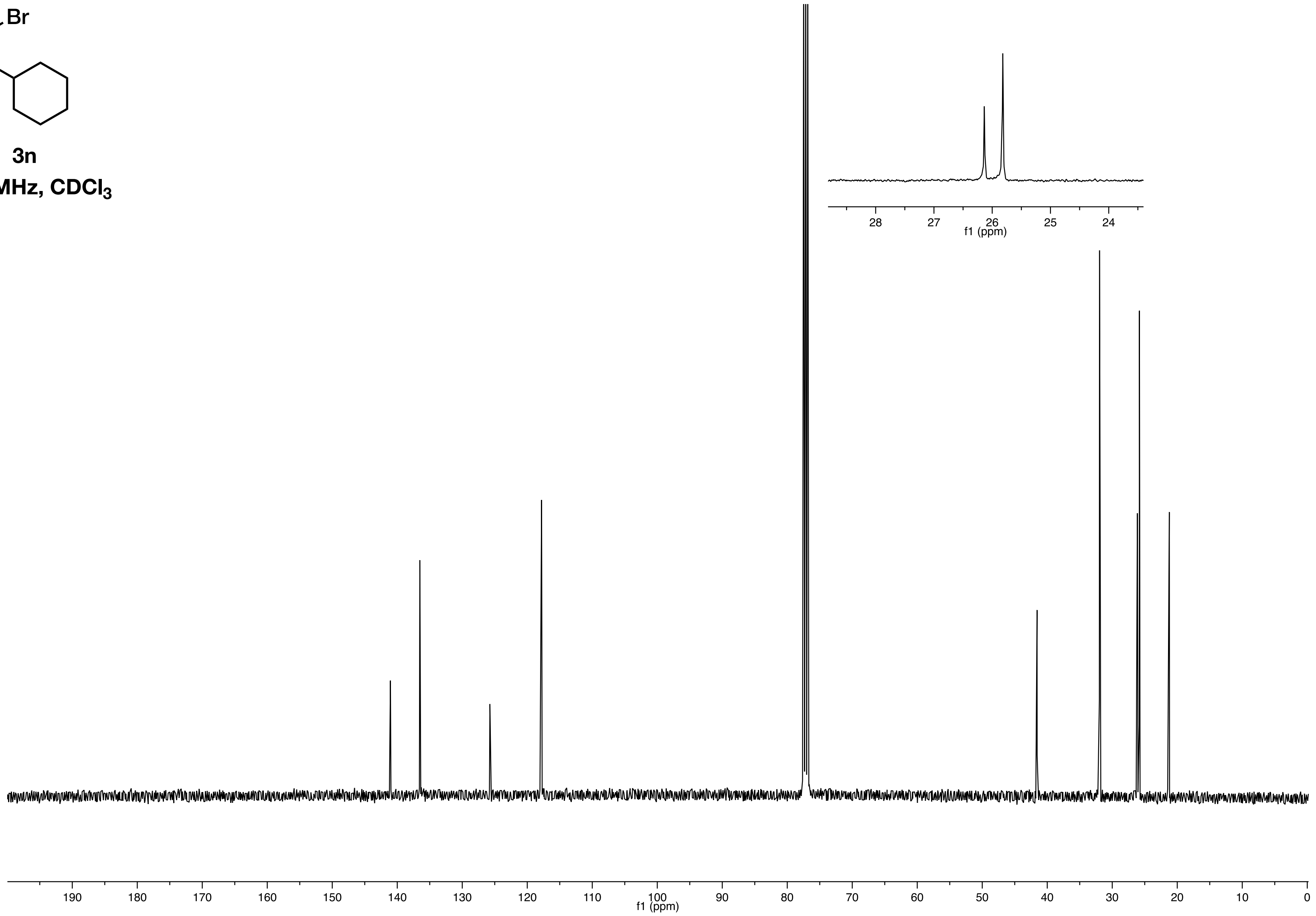

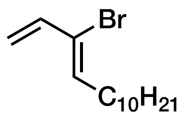

3o
400 MHz, $CDCl_3$

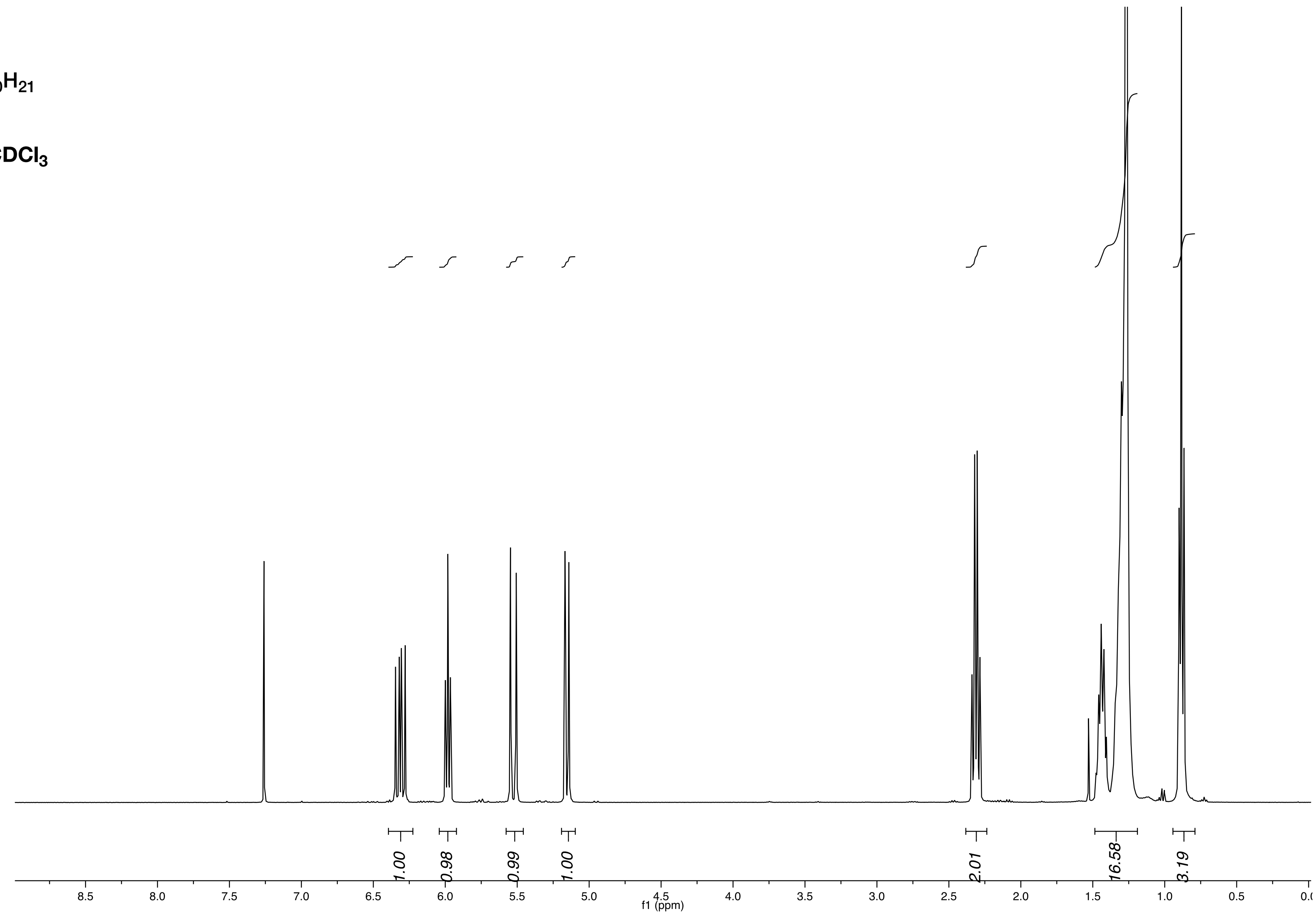

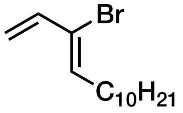

3o
100 MHz, CDCl$_3$

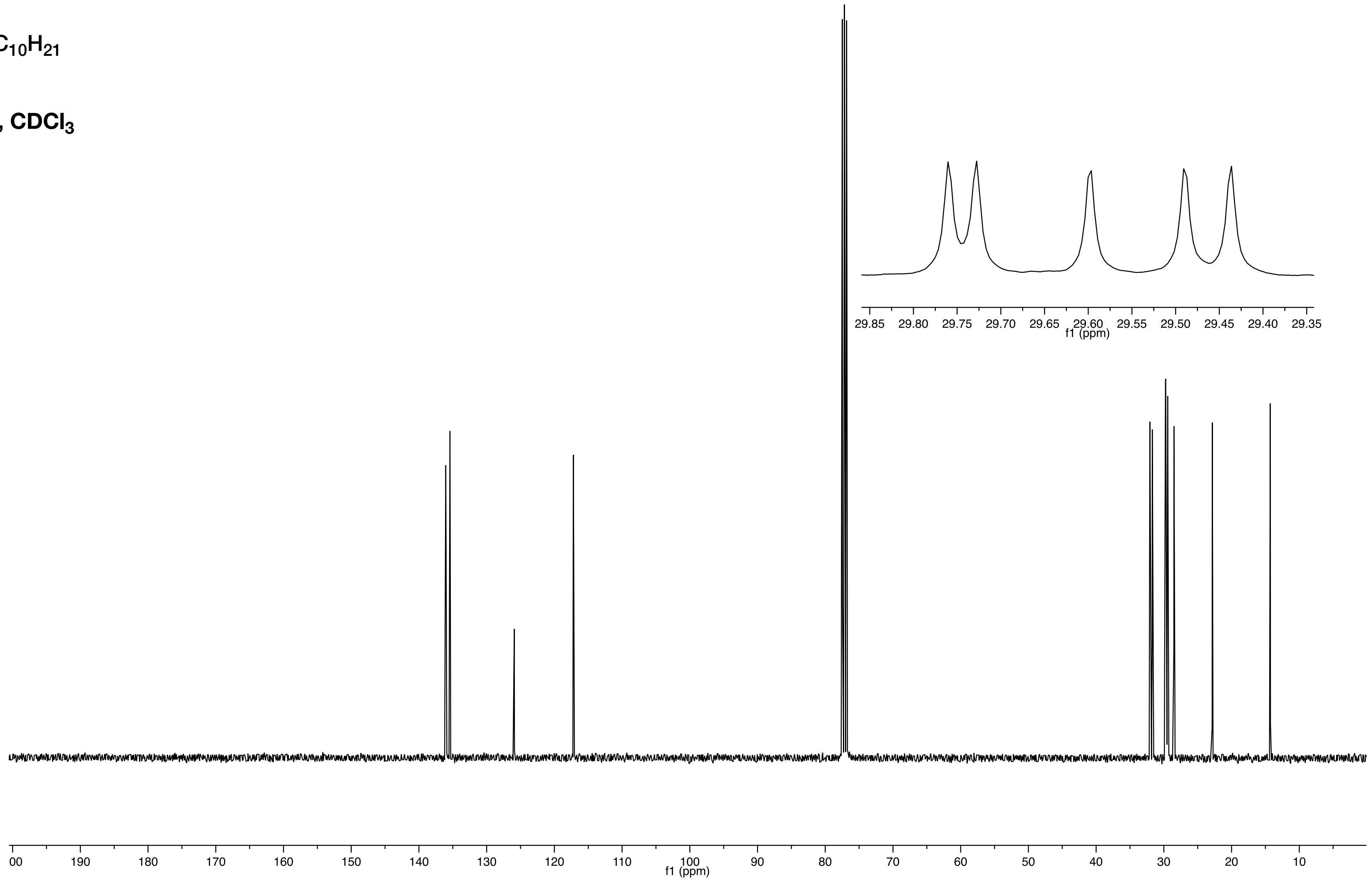

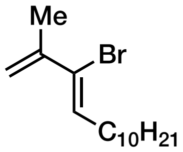

3p

400 MHz, $CDCl_3$

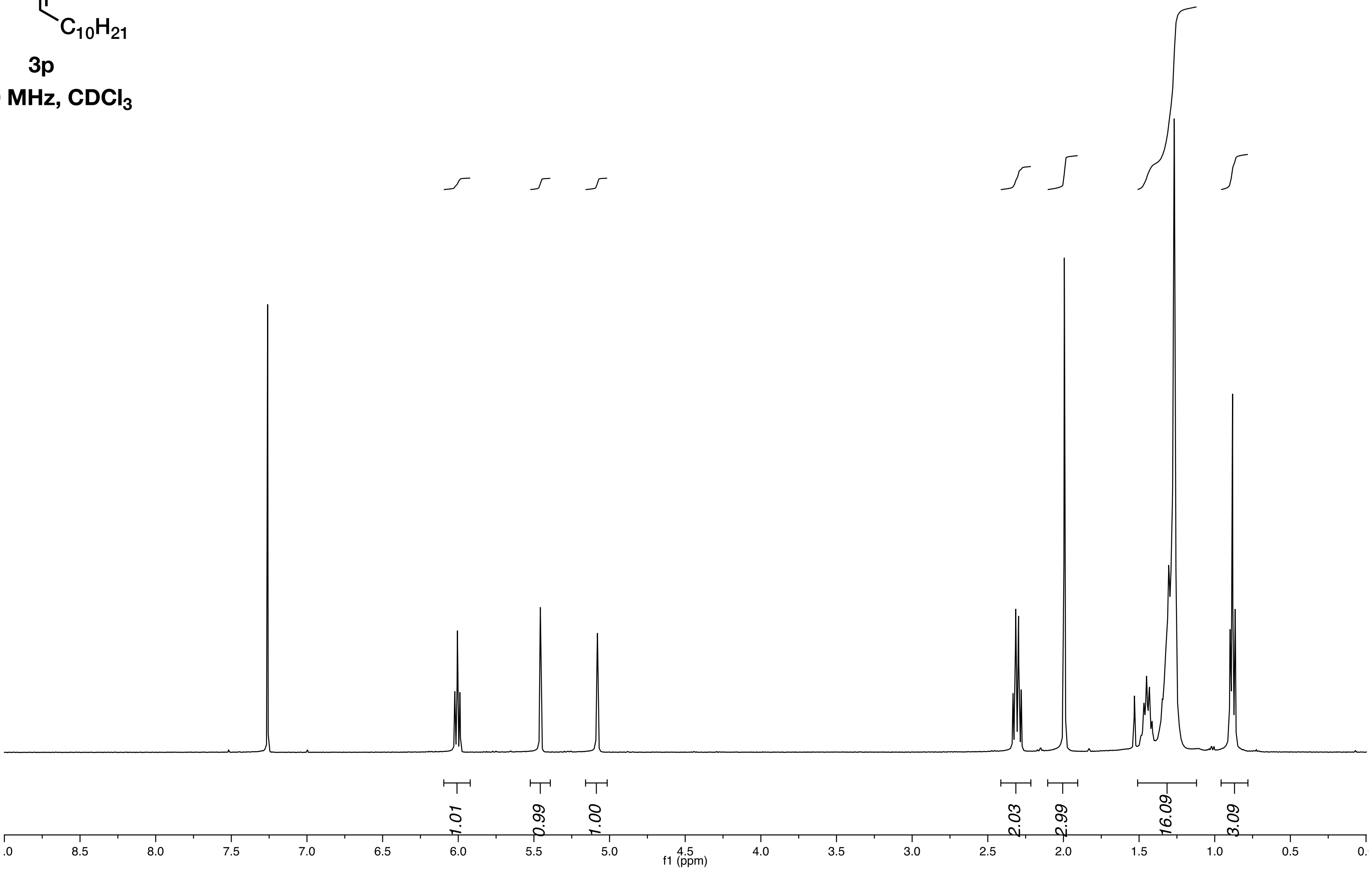

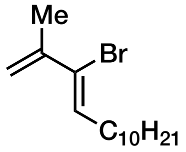

3p

100 MHz, $CDCl_3$

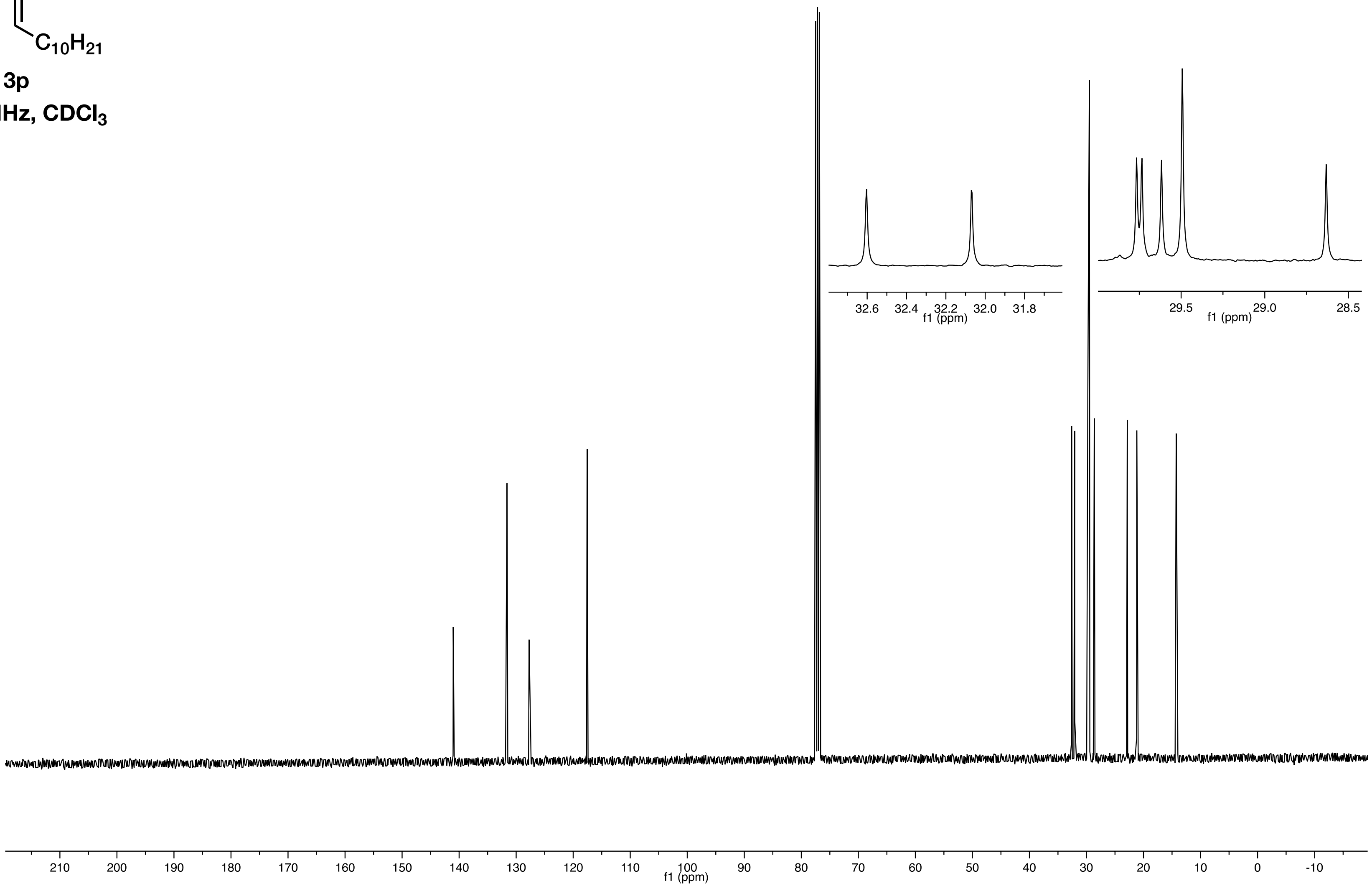

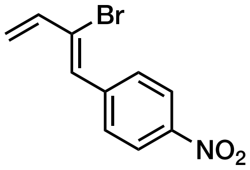

3q

400 MHz, $CDCl_3$

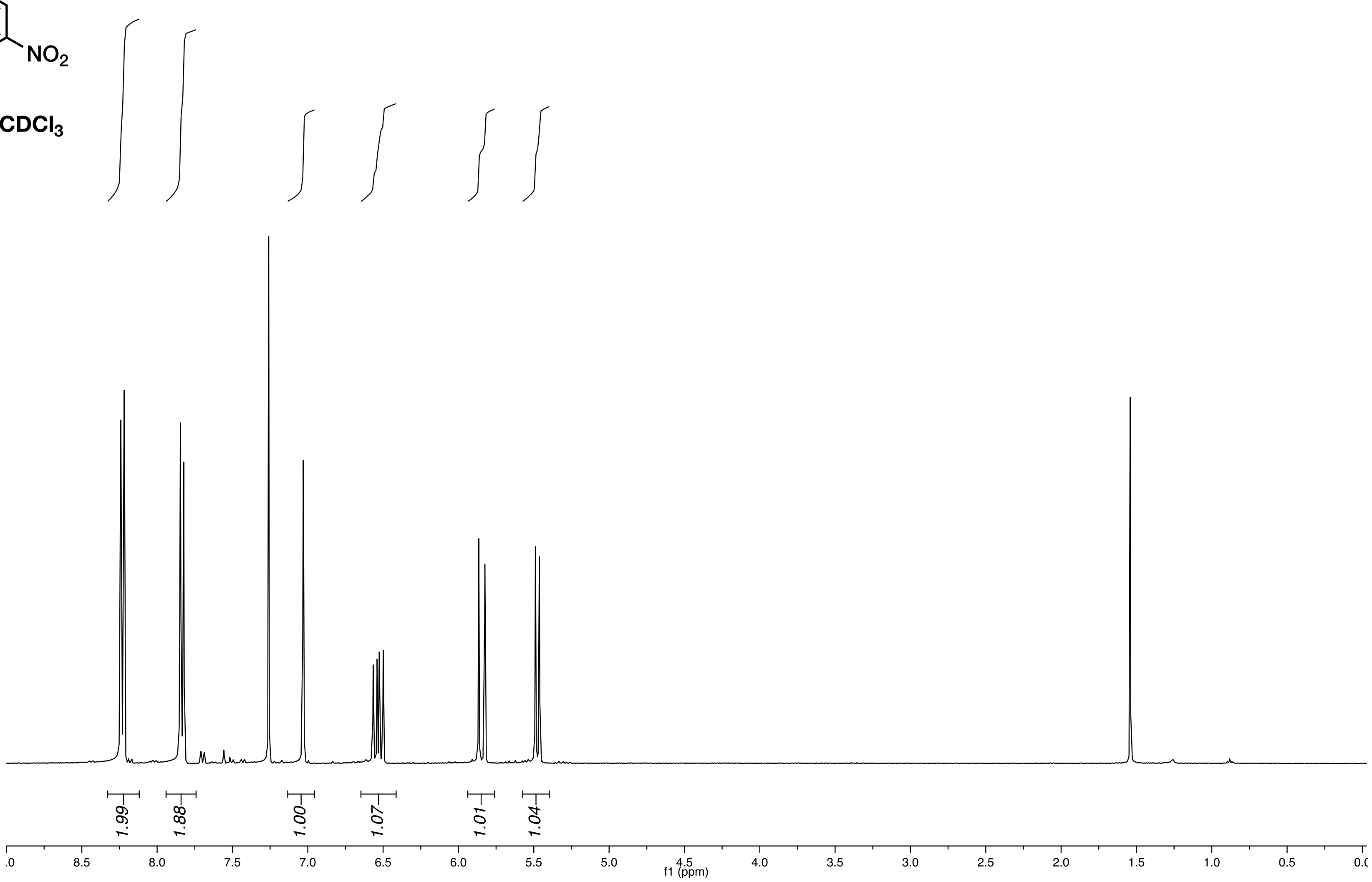

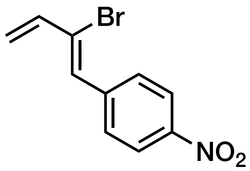

3q

100 MHz, CDCl$_3$

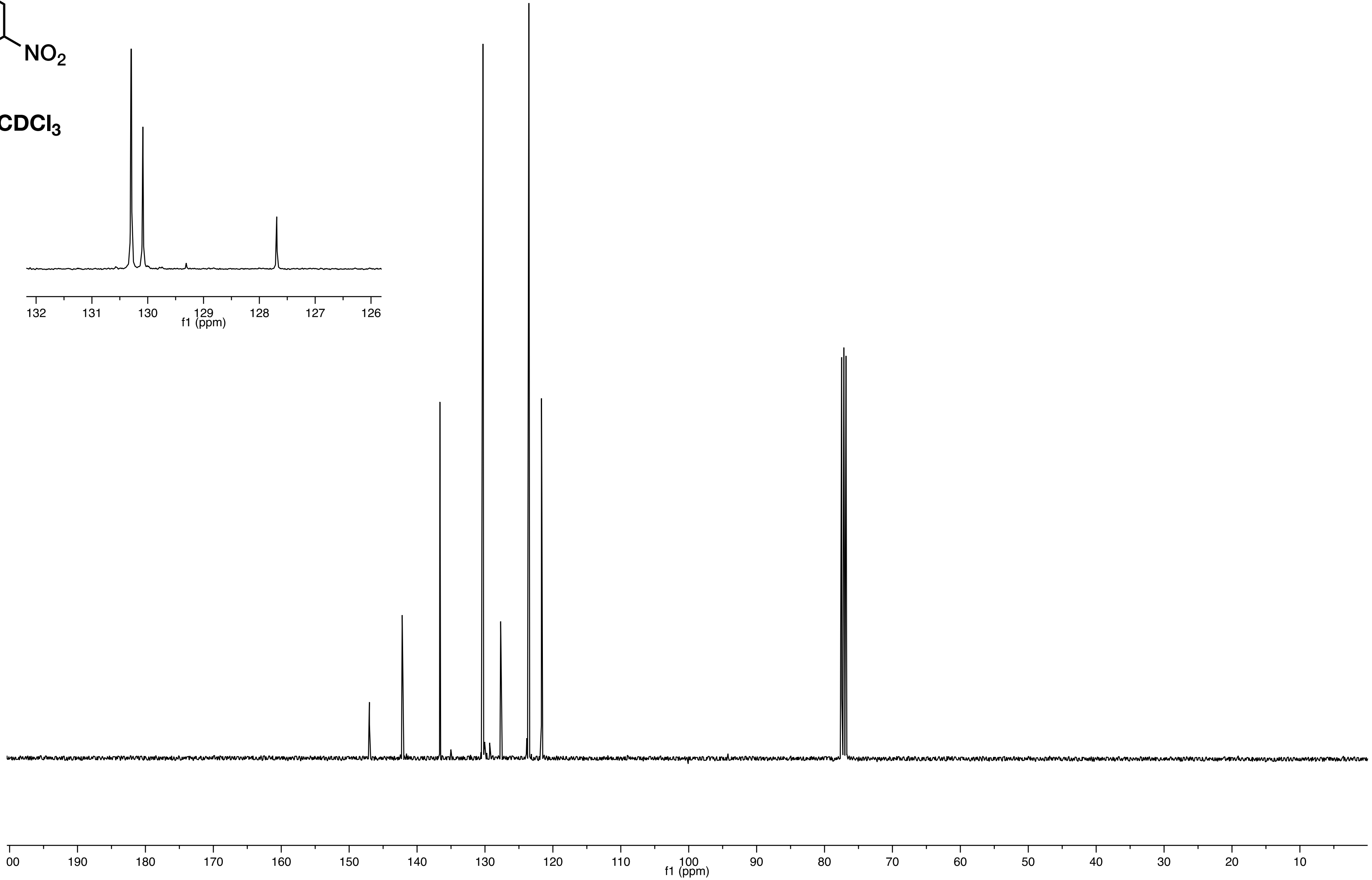

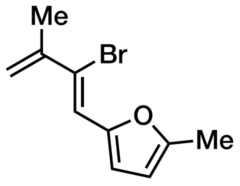

3r

400 MHz, $CDCl_3$

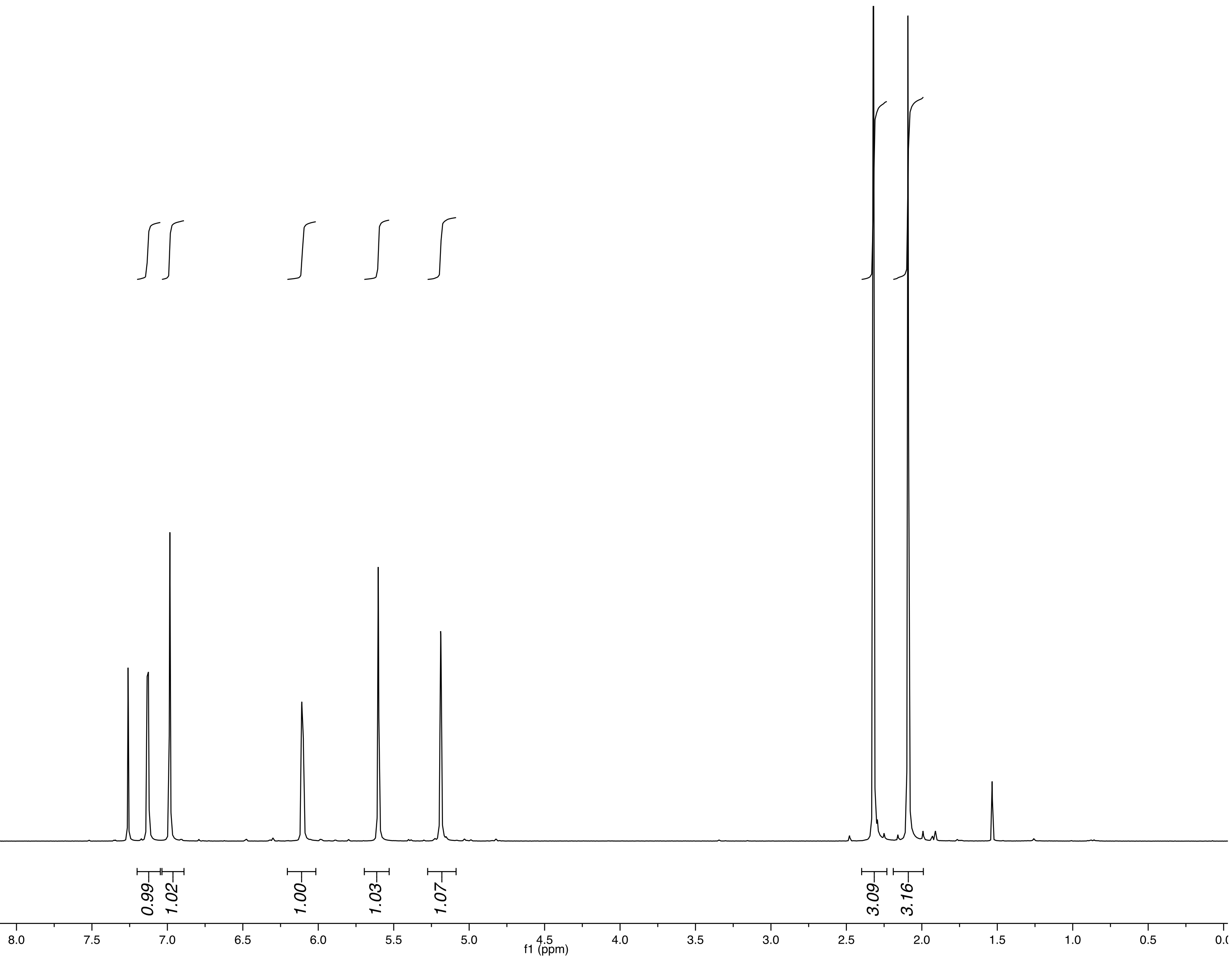

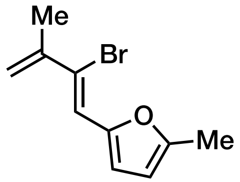

3r

100 MHz, $CDCl_3$

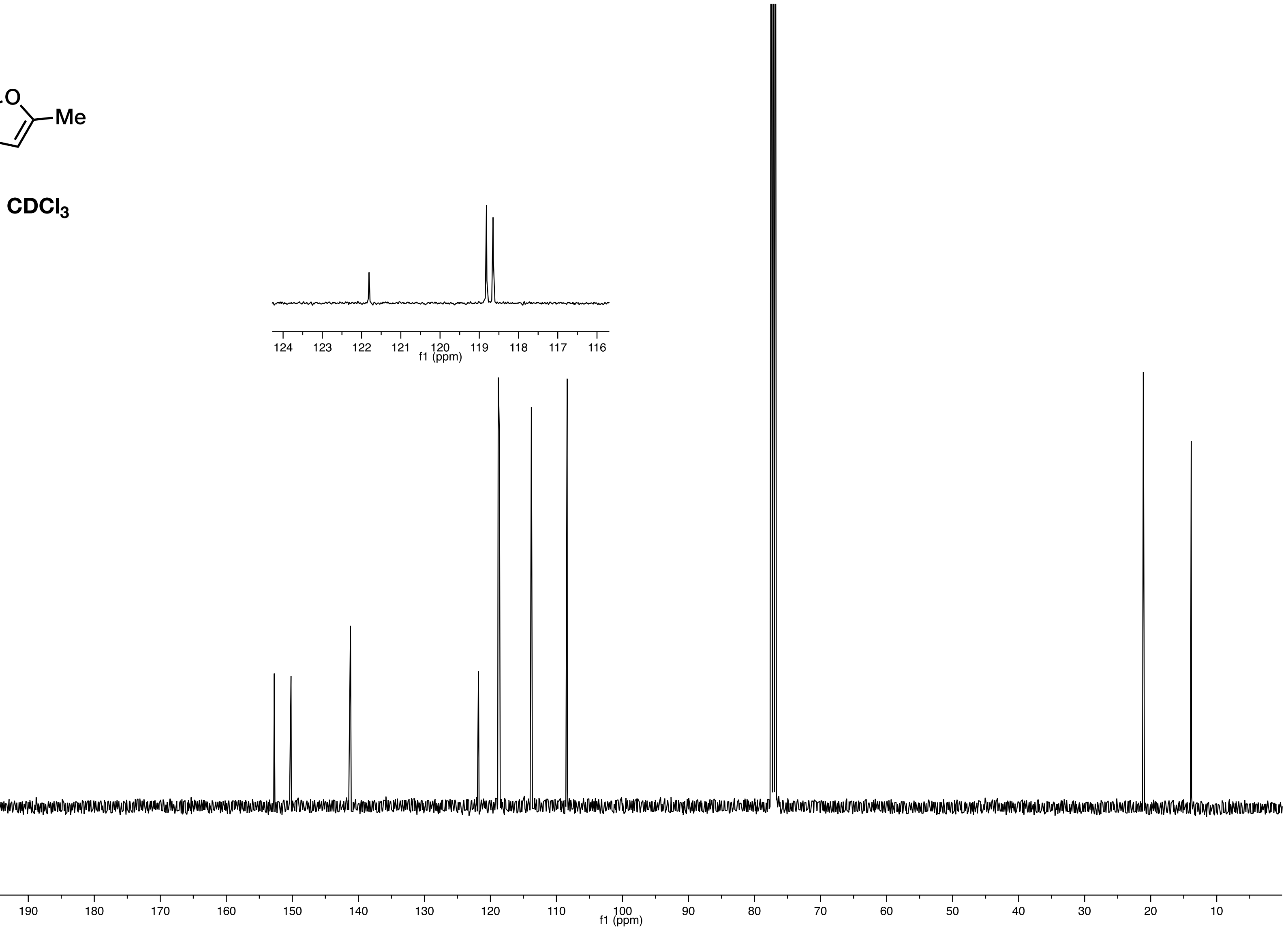

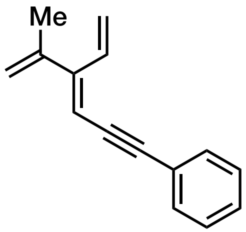

4a-*E*

400 MHz, $CDCl_3$

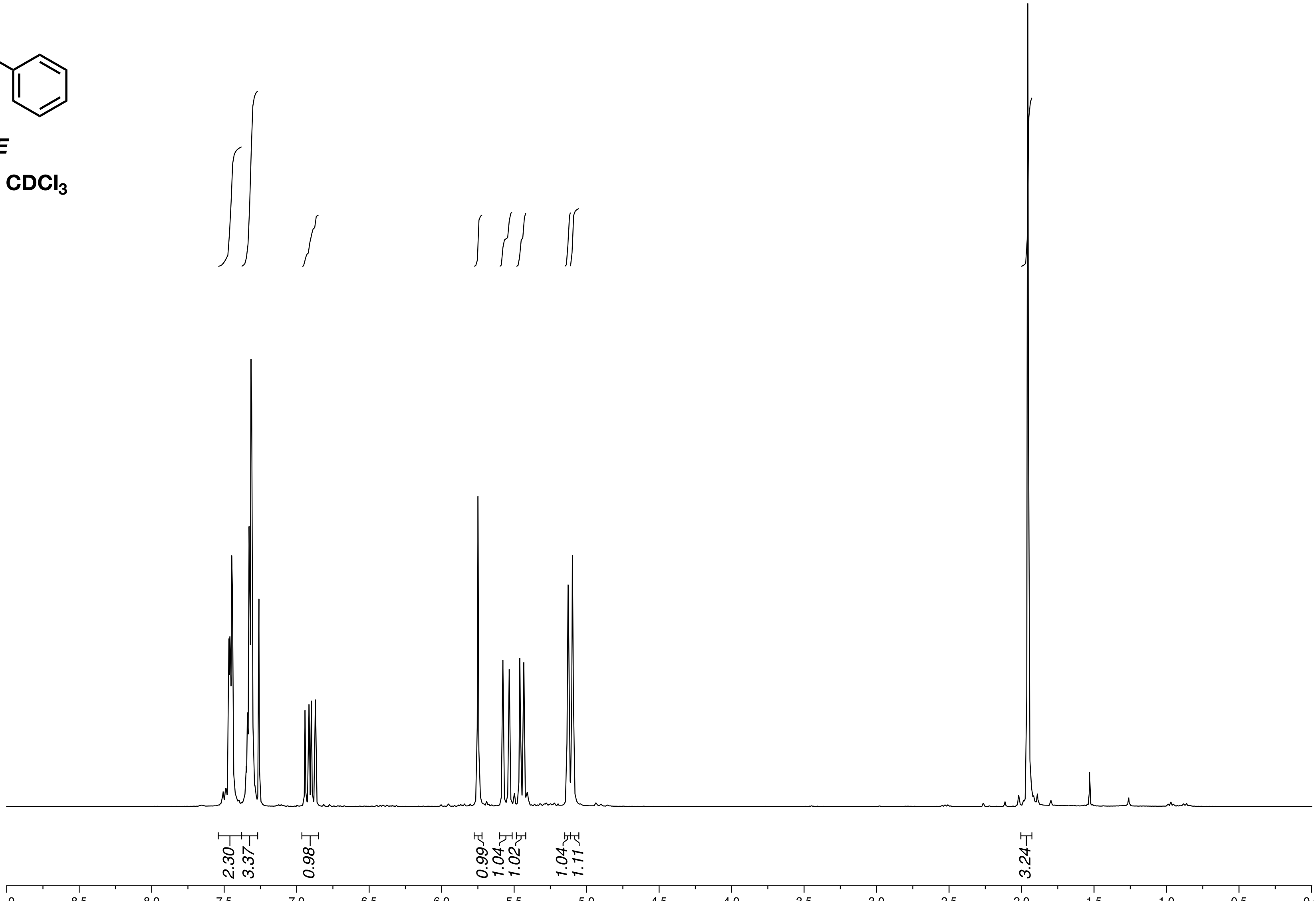

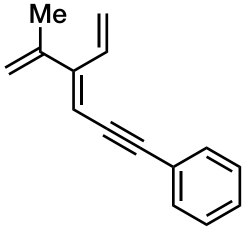

4a-*E*

100 MHz, $CDCl_3$

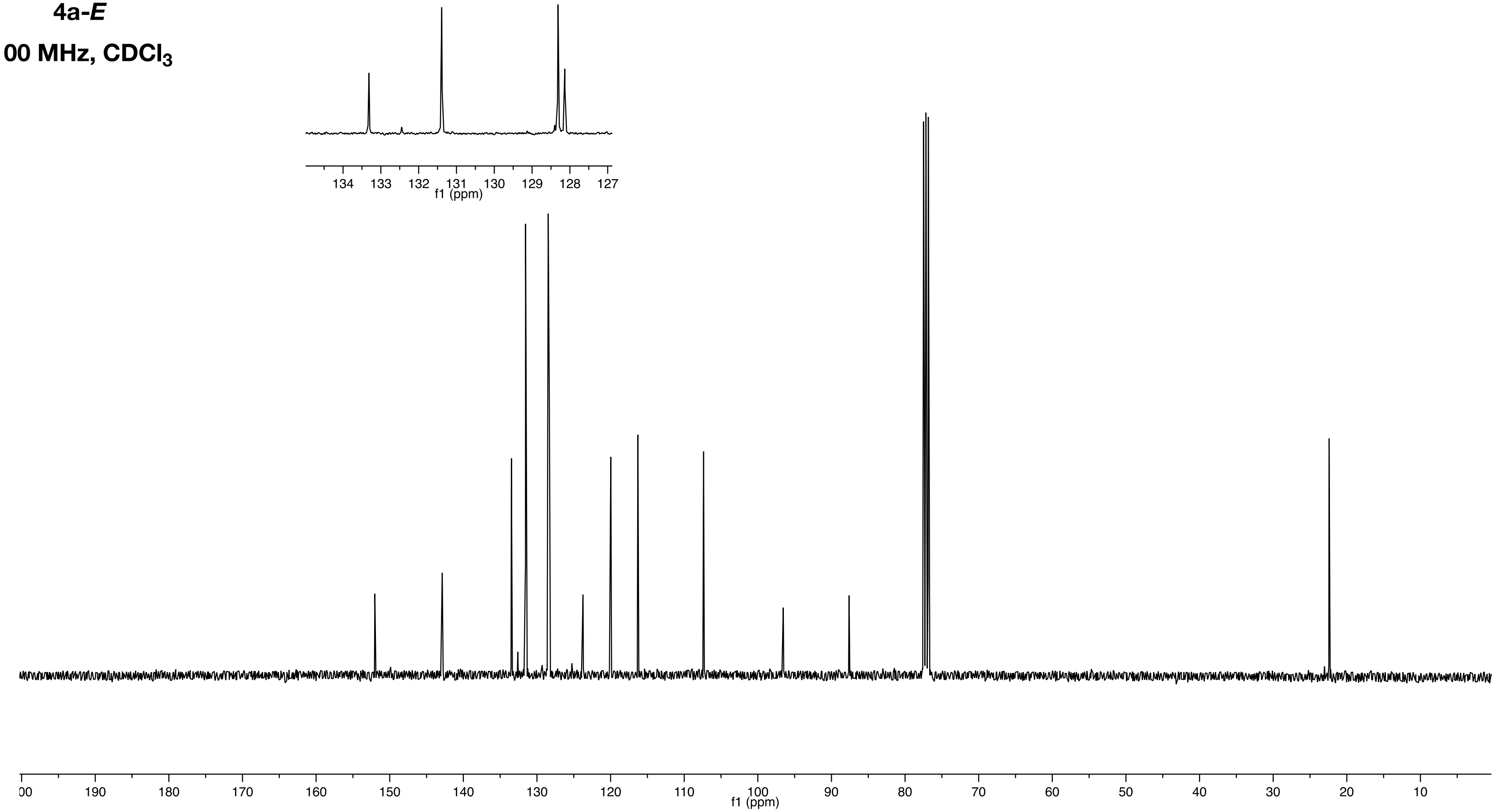

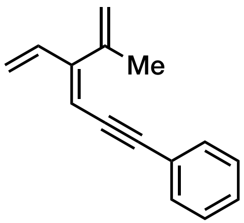

4a-*Z*

400 MHz, $CDCl_3$

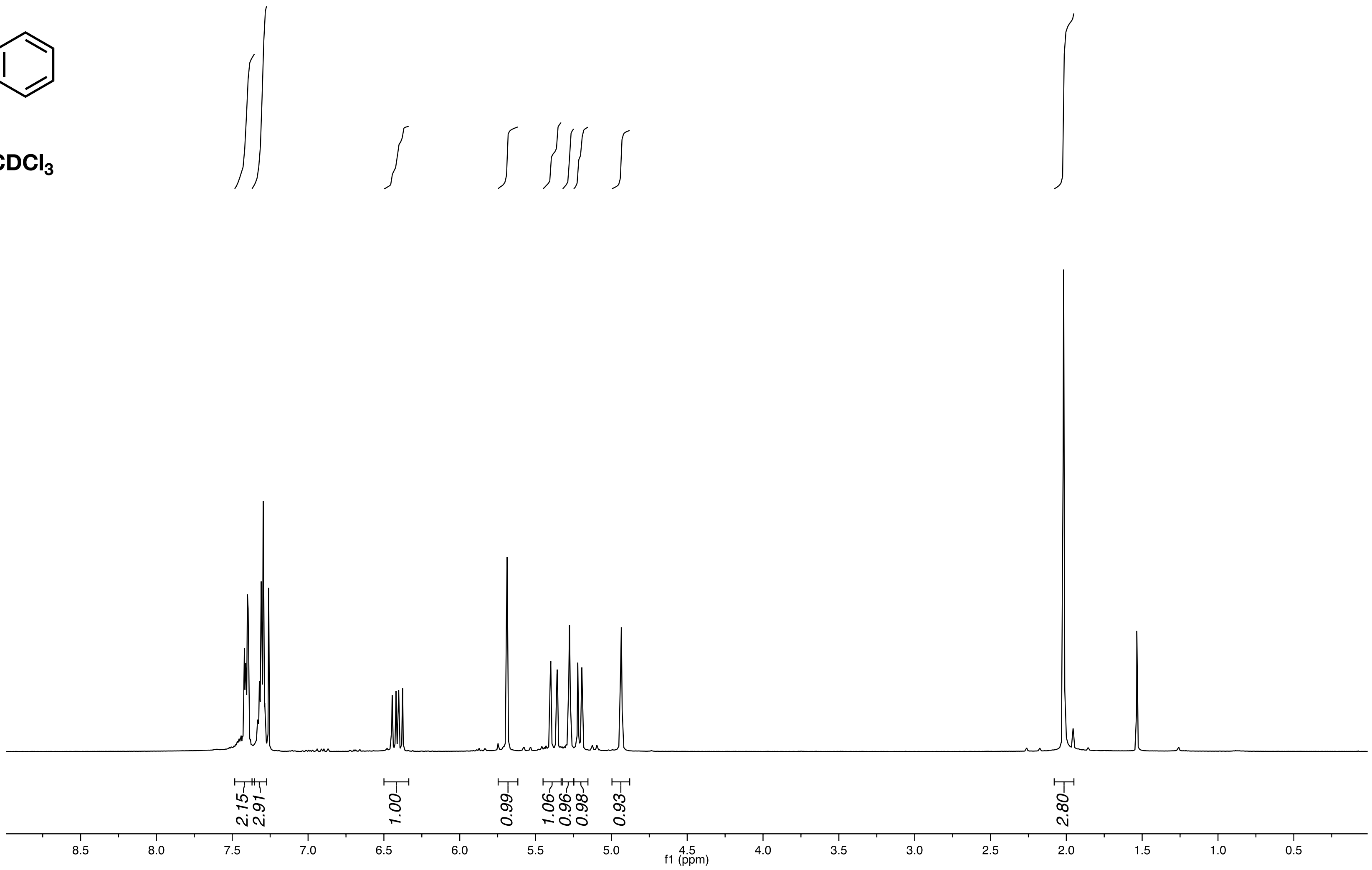

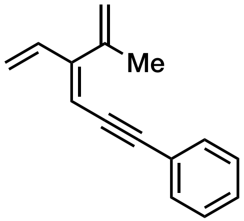

4a-*Z*

100 MHz, $CDCl_3$

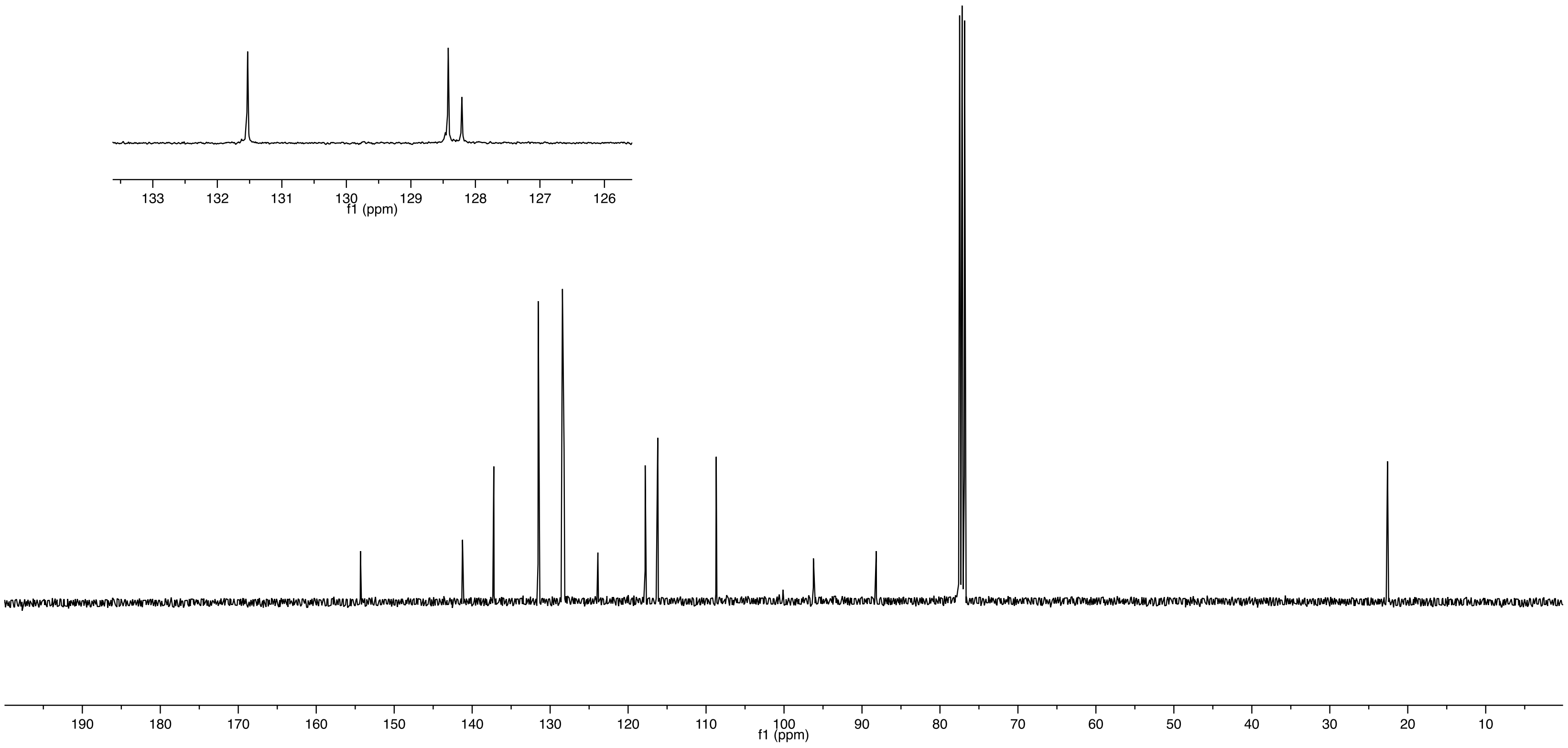

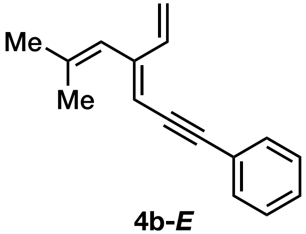

4b-*E*

400 MHz, $CDCl_3$

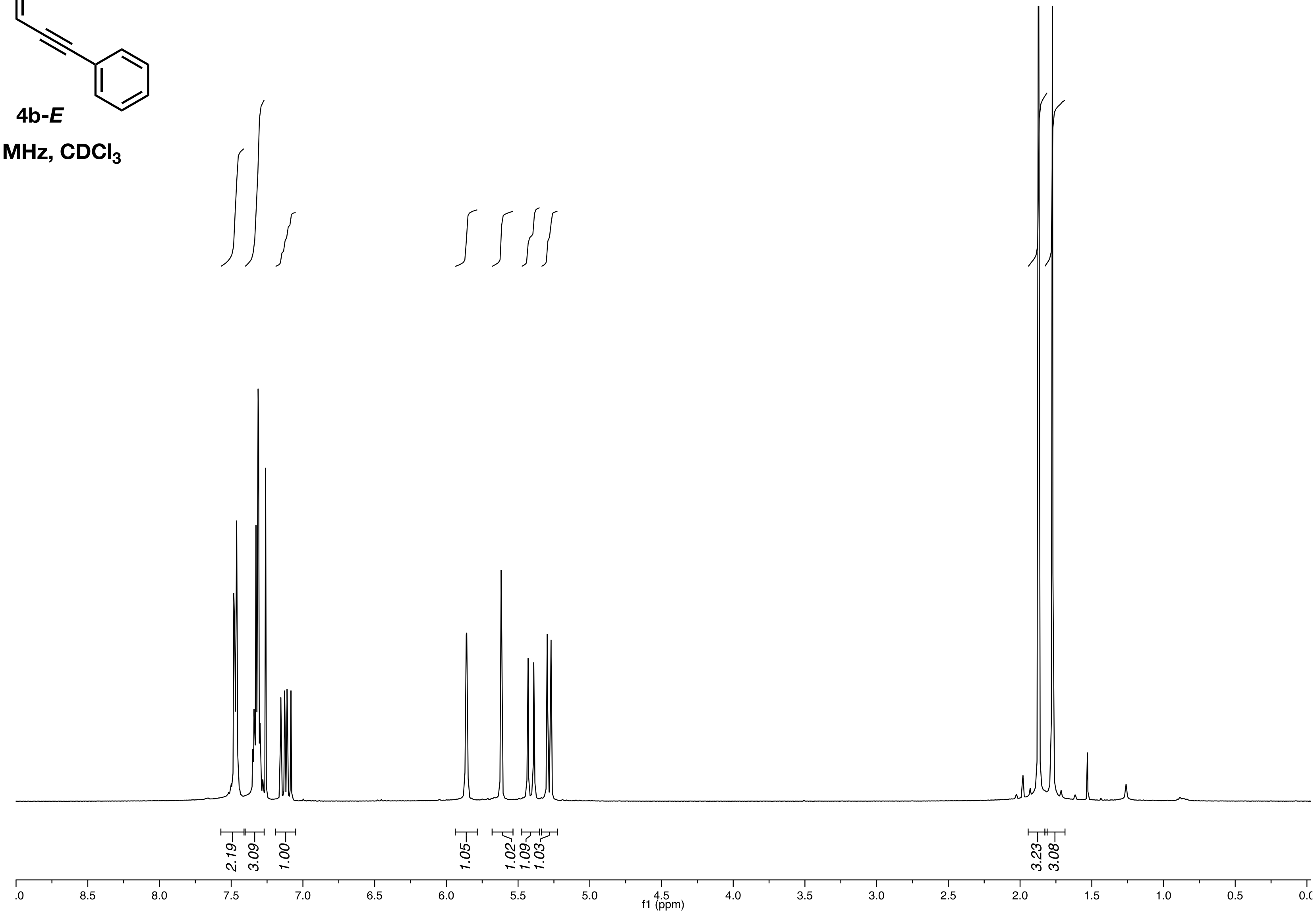

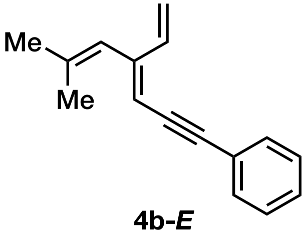

4b-*E*

100 MHz, $CDCl_3$

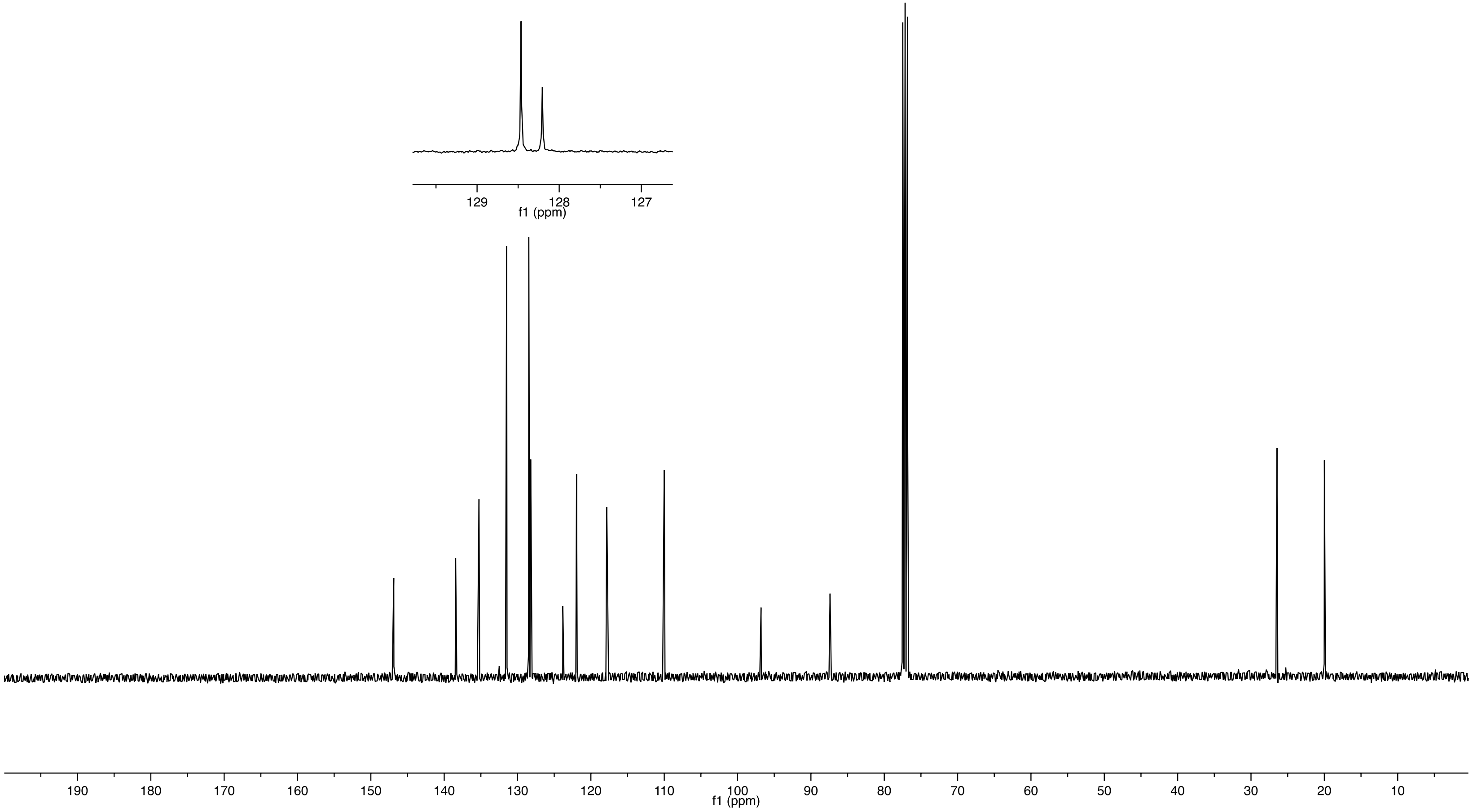

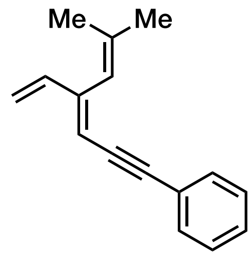

4b-*Z*

400 MHz, $CDCl_3$

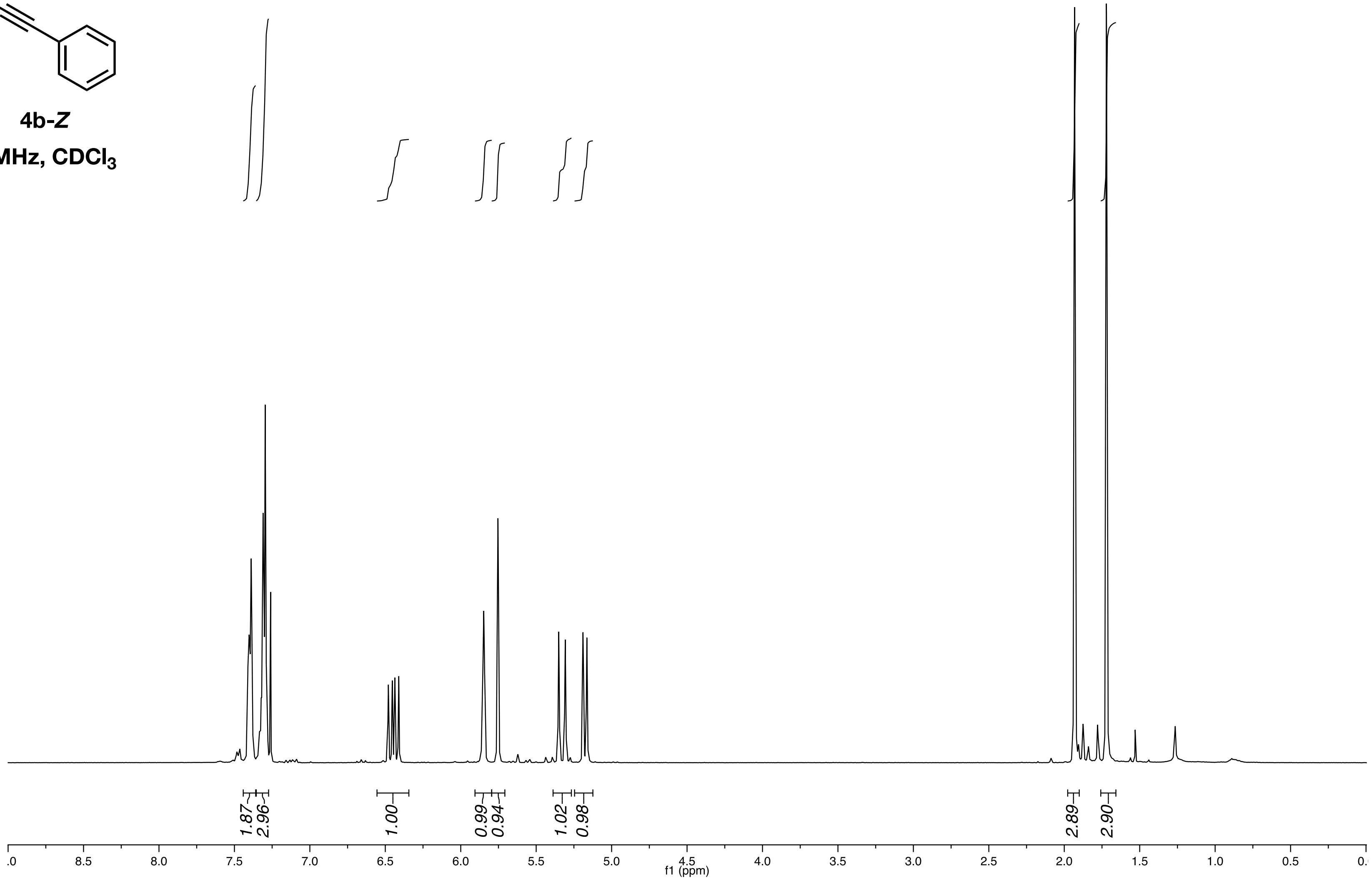

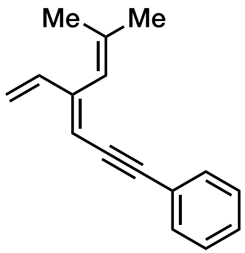

4b-*Z*

100 MHz, $CDCl_3$

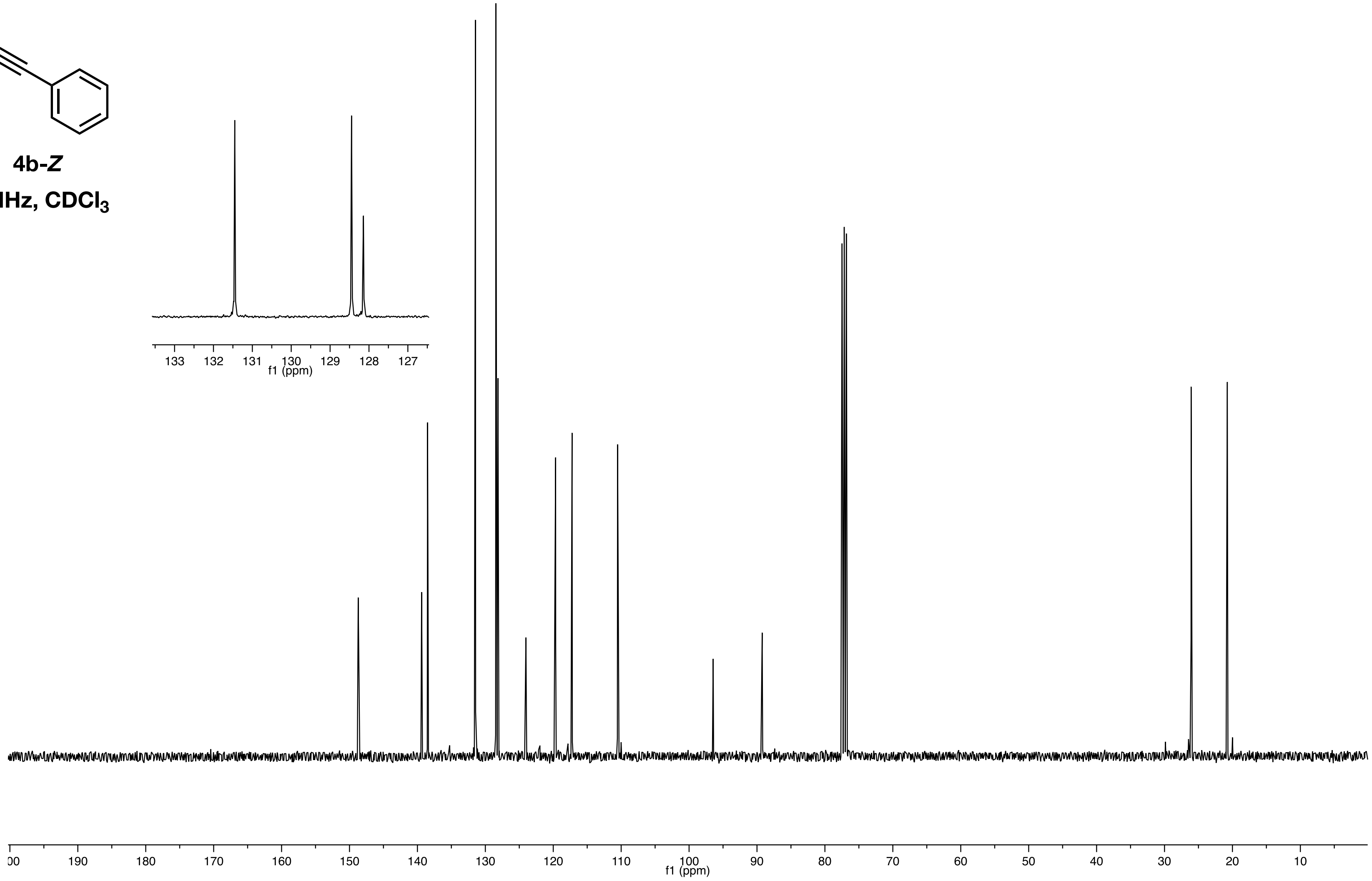

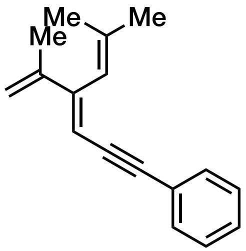

4c-*E*

400 MHz, $CDCl_3$

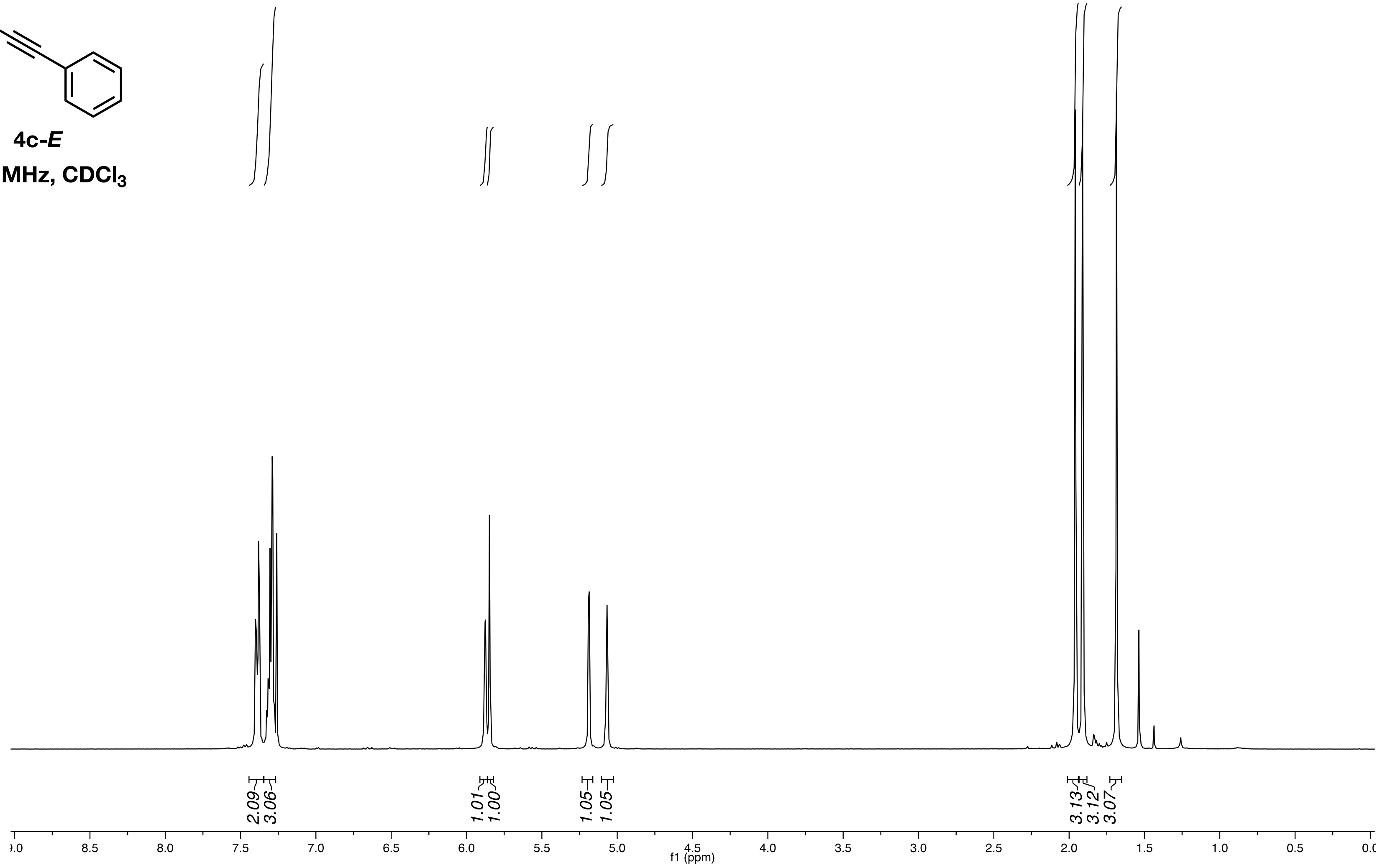

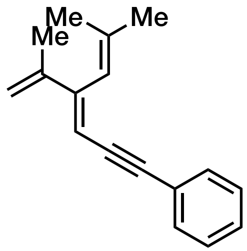

4c-*E*

100 MHz, $CDCl_3$

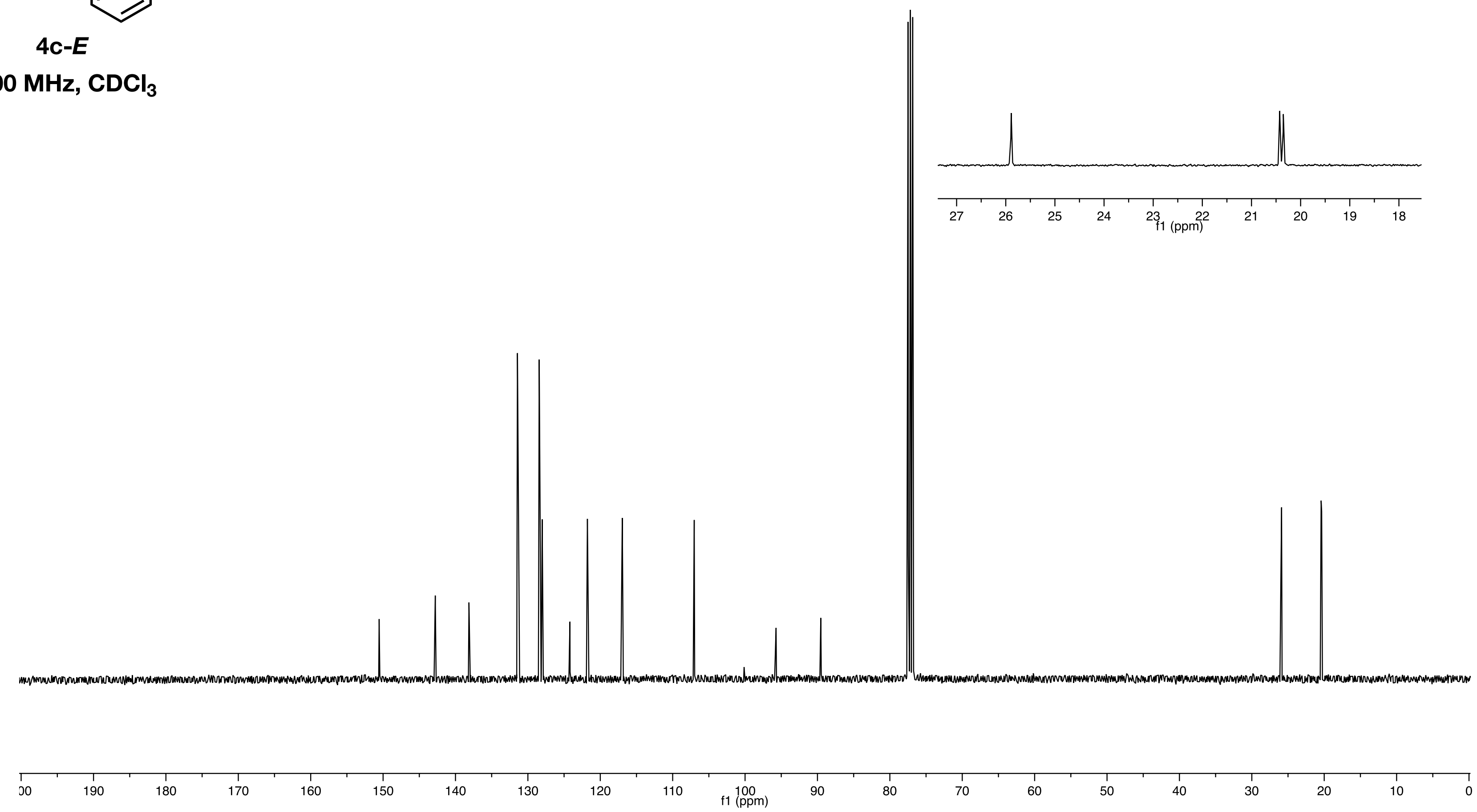

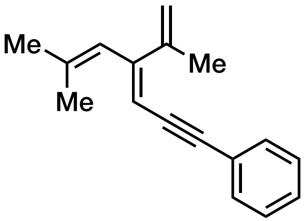

4c-*Z*
400 MHz, $CDCl_3$

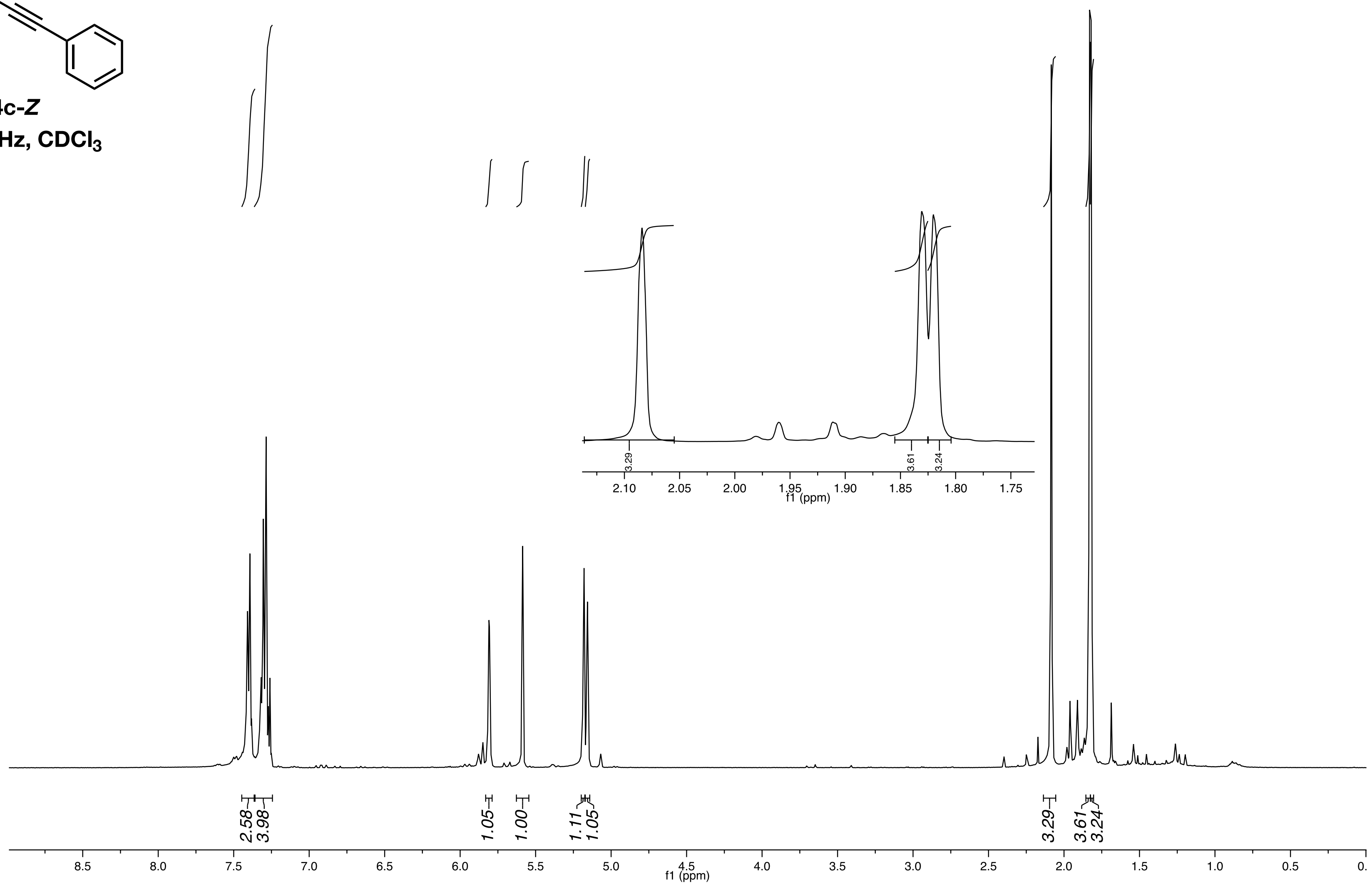

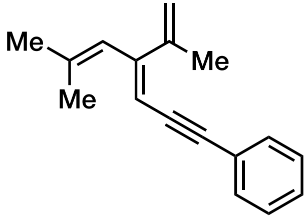

4c-*Z*
100 MHz, $CDCl_3$

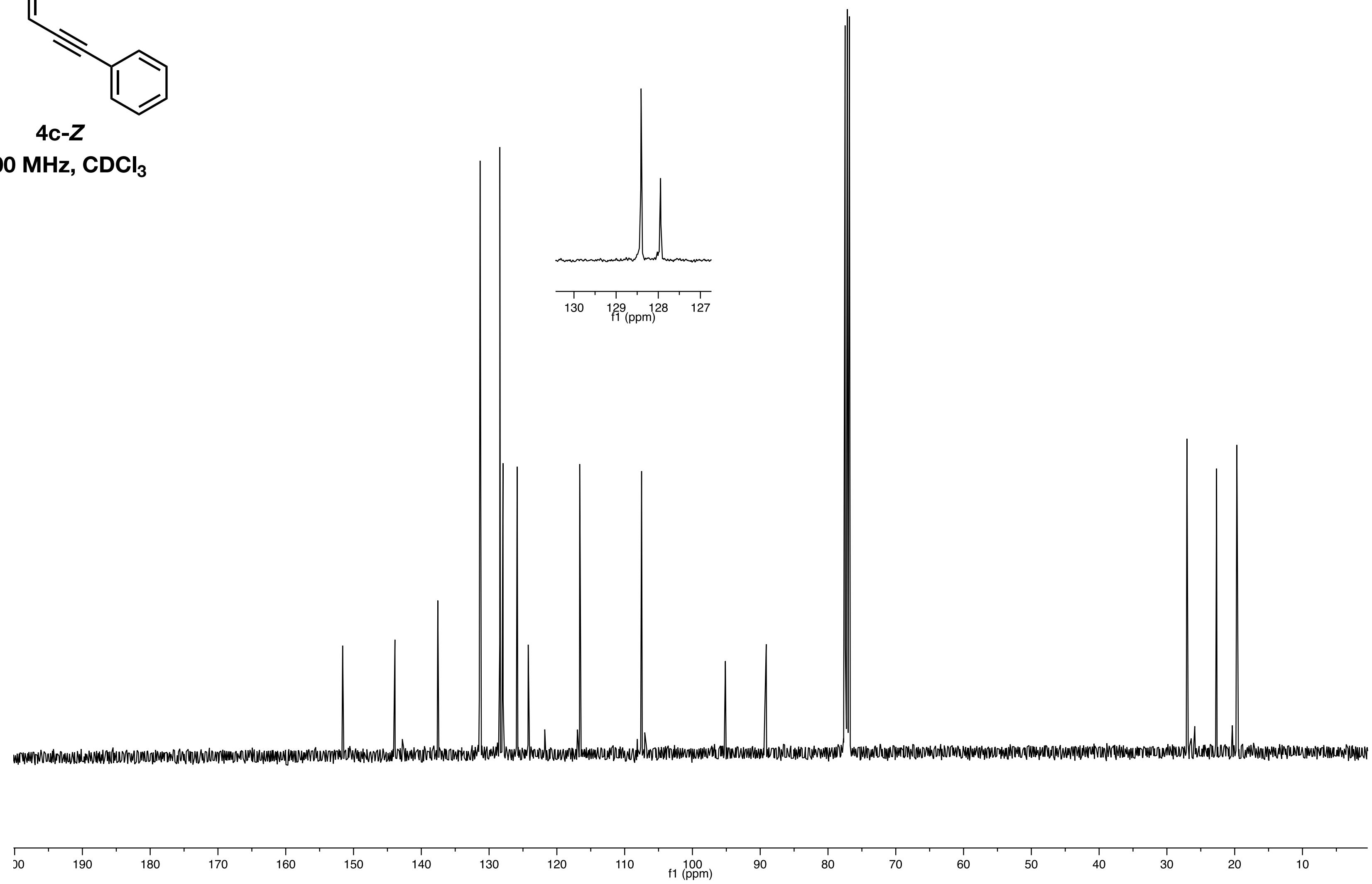

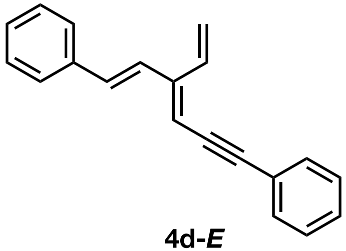

4d-*E*

400 MHz, $CDCl_3$

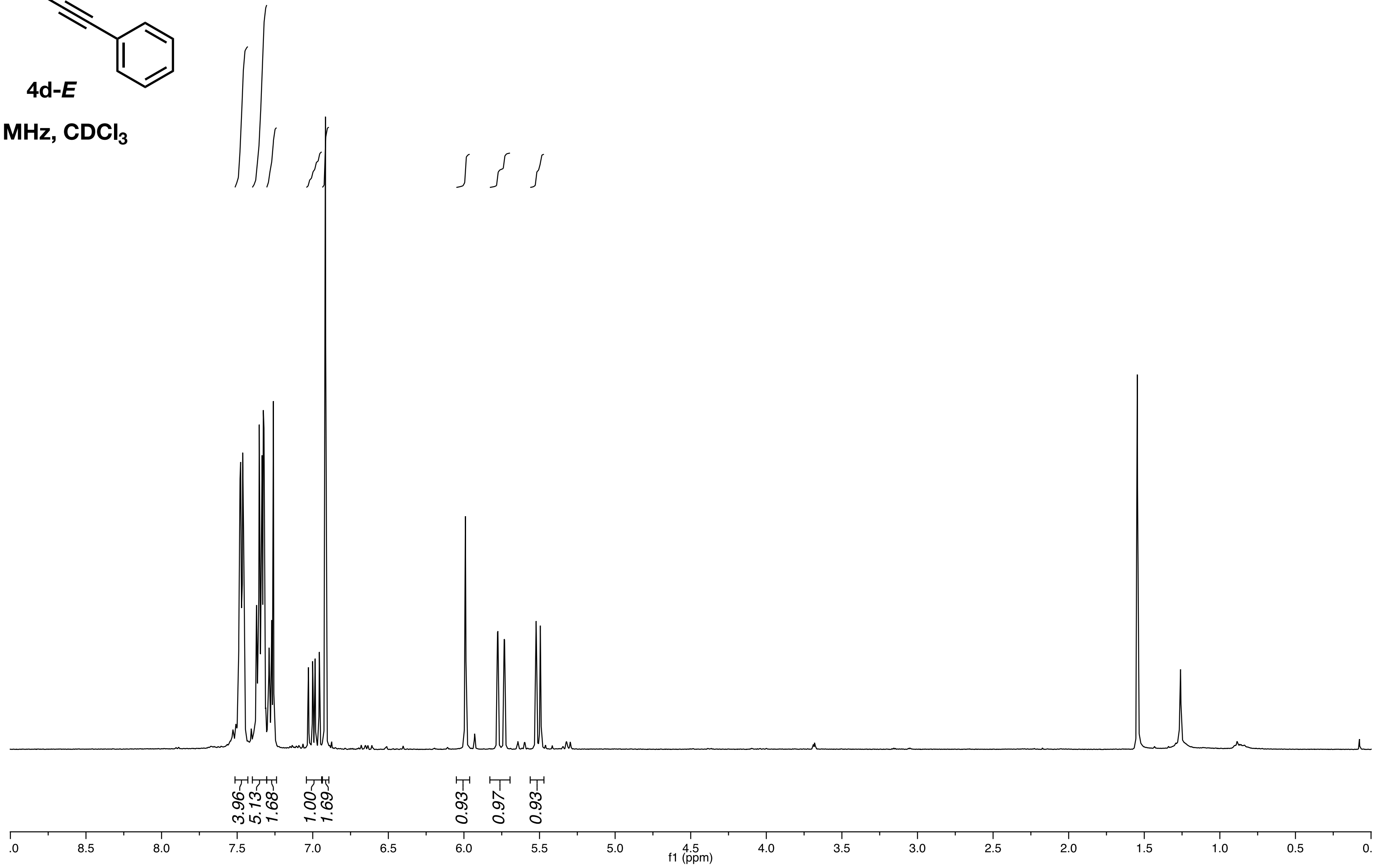

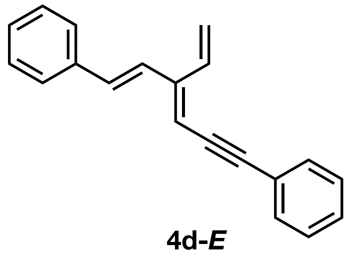

4d-*E*

100 MHz, $CDCl_3$

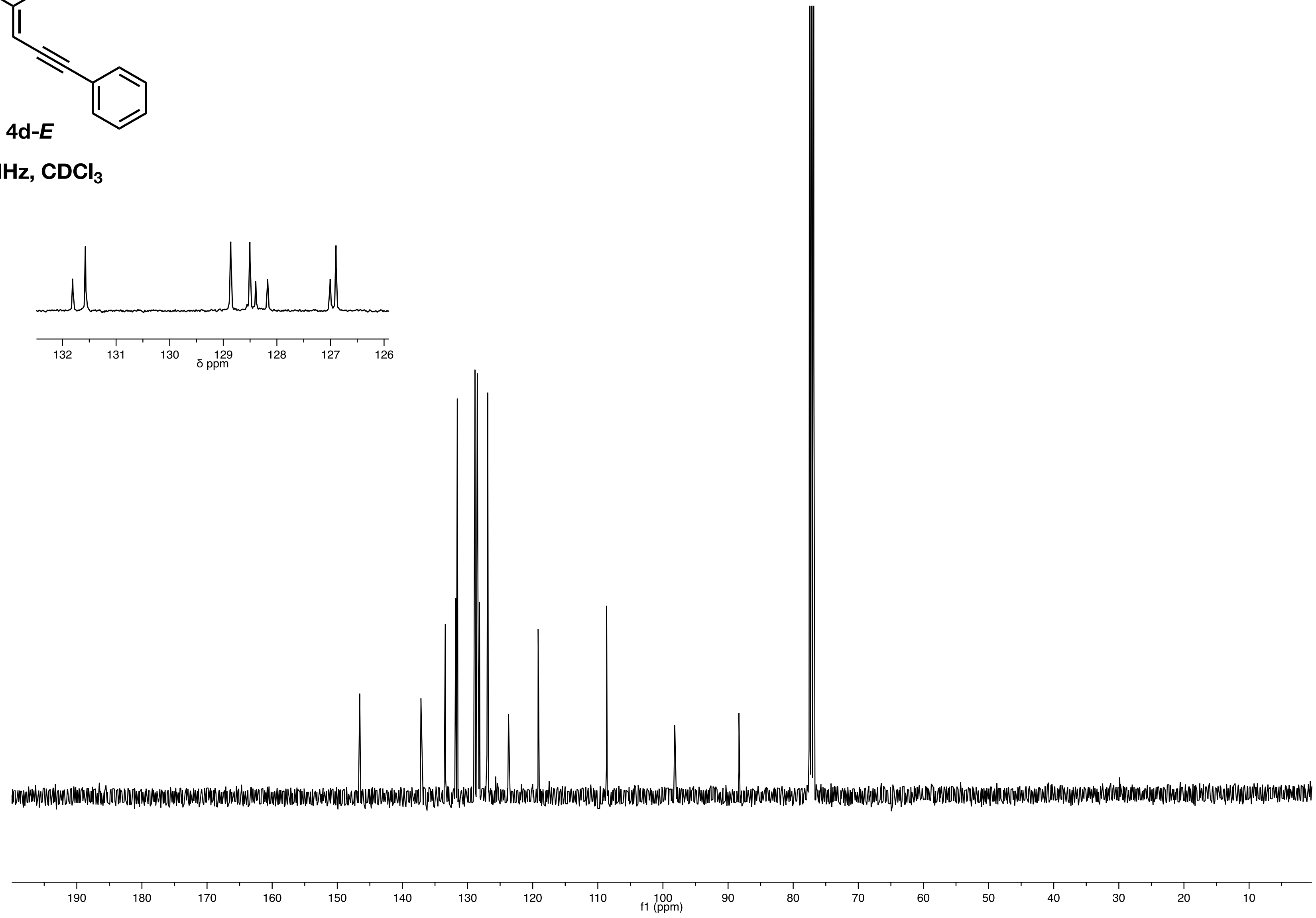

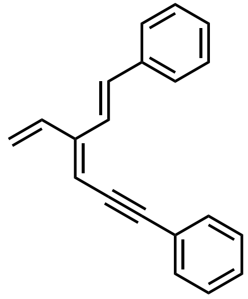

4d-*Z*
400 MHz, $CDCl_3$

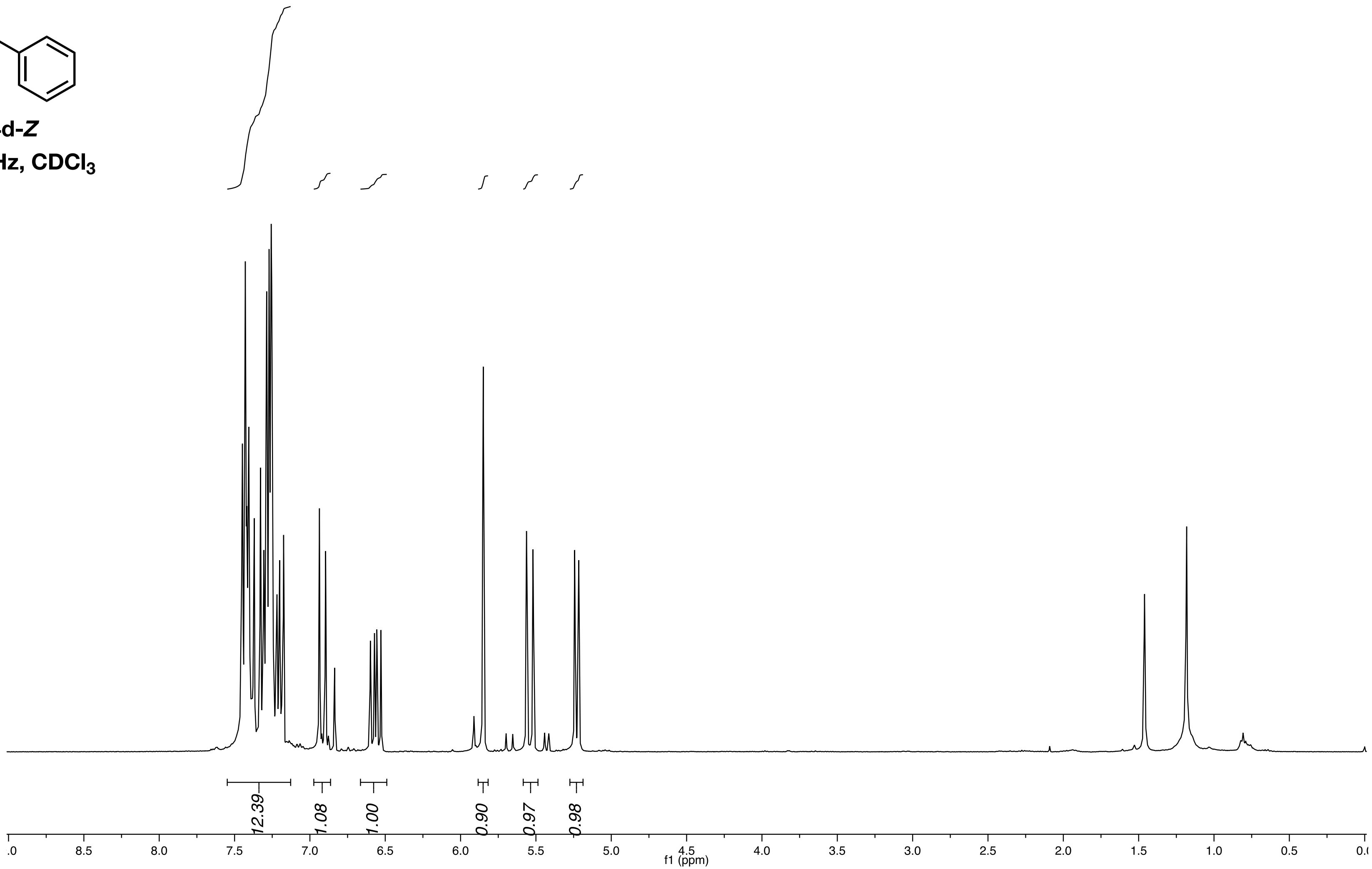

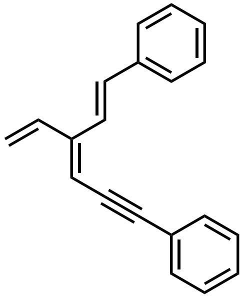

4d-*Z*
100 MHz, $CDCl_3$

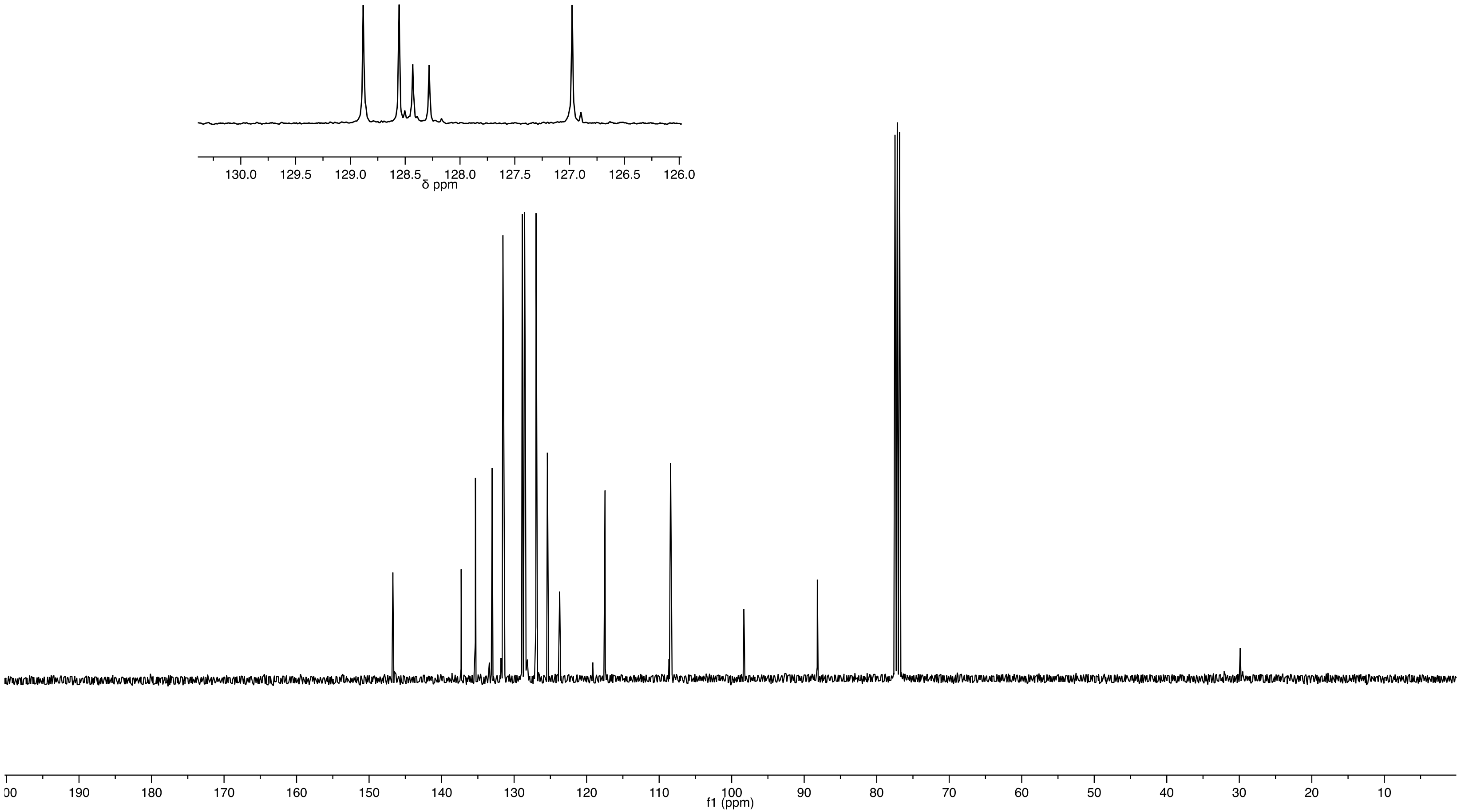

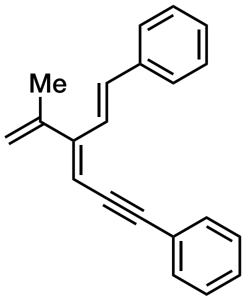

4e-*E*

400 MHz, $CDCl_3$

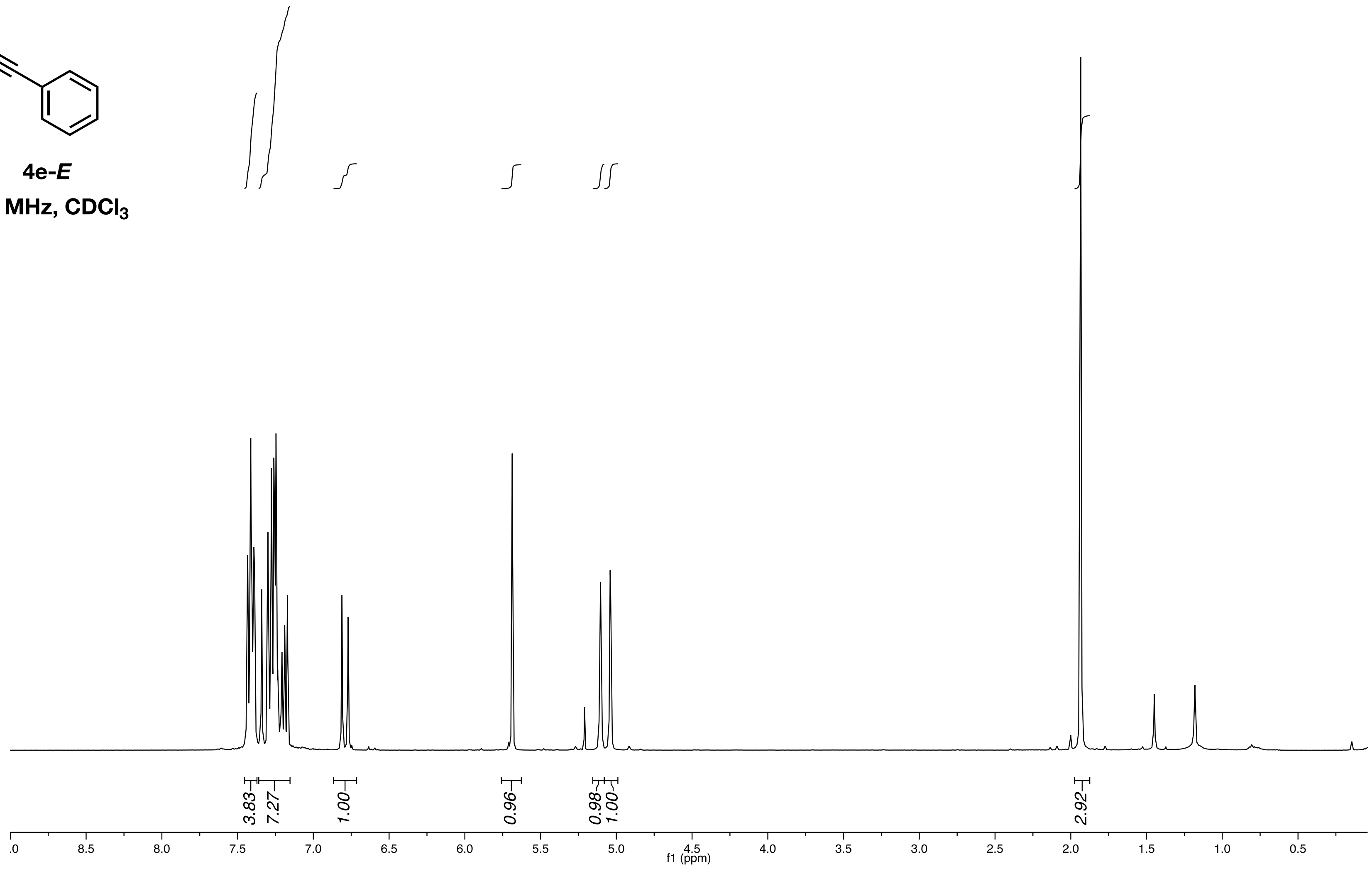

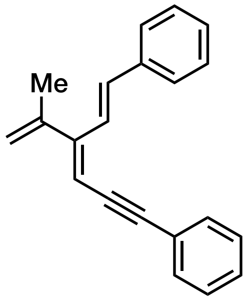

4e-*E*

100 MHz, $CDCl_3$

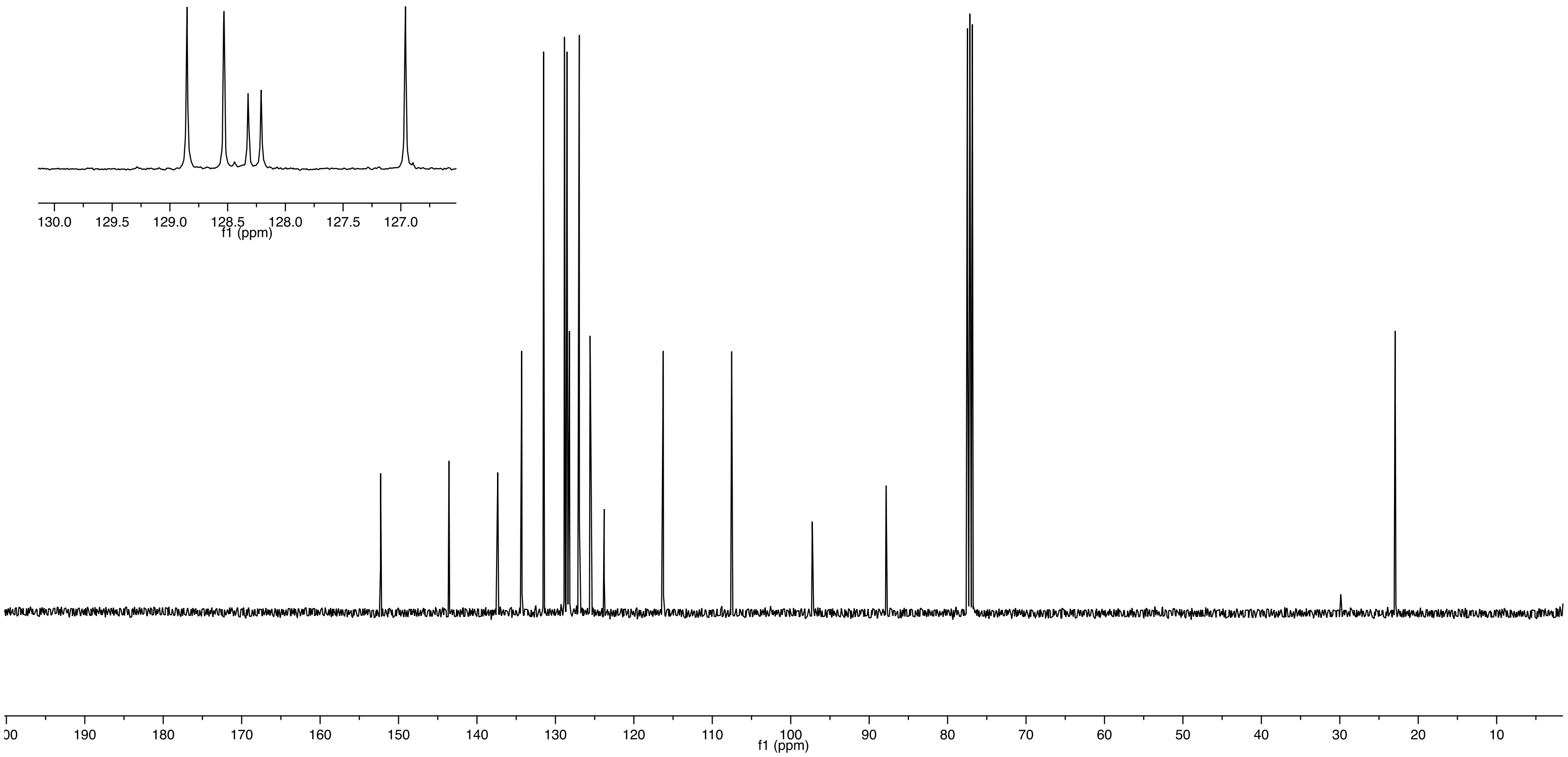

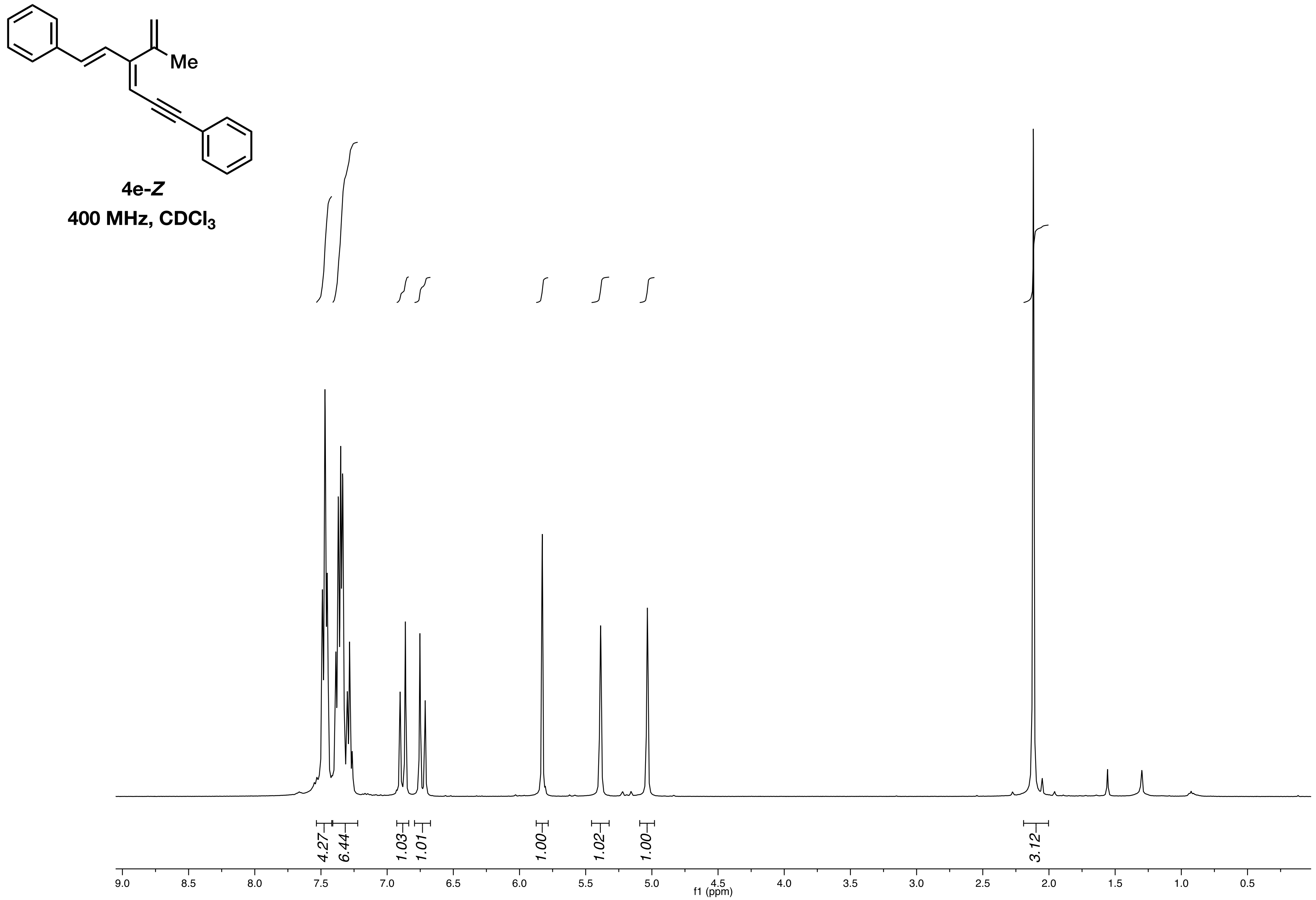
Me
4e-Z
400 MHz, CDCl3
4.27
6.44
1.03
1.01
1.00
1.02
1.00
3.12
9.0
8.5
8.0
7.5
7.0
6.5
6.0
5.5
5.0
4.5
4.0
3.5
3.0
2.5
2.0
1.5
1.0
0.5
f1 (ppm)

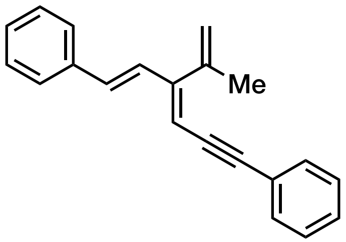

4e-*Z*

100 MHz, $CDCl_3$

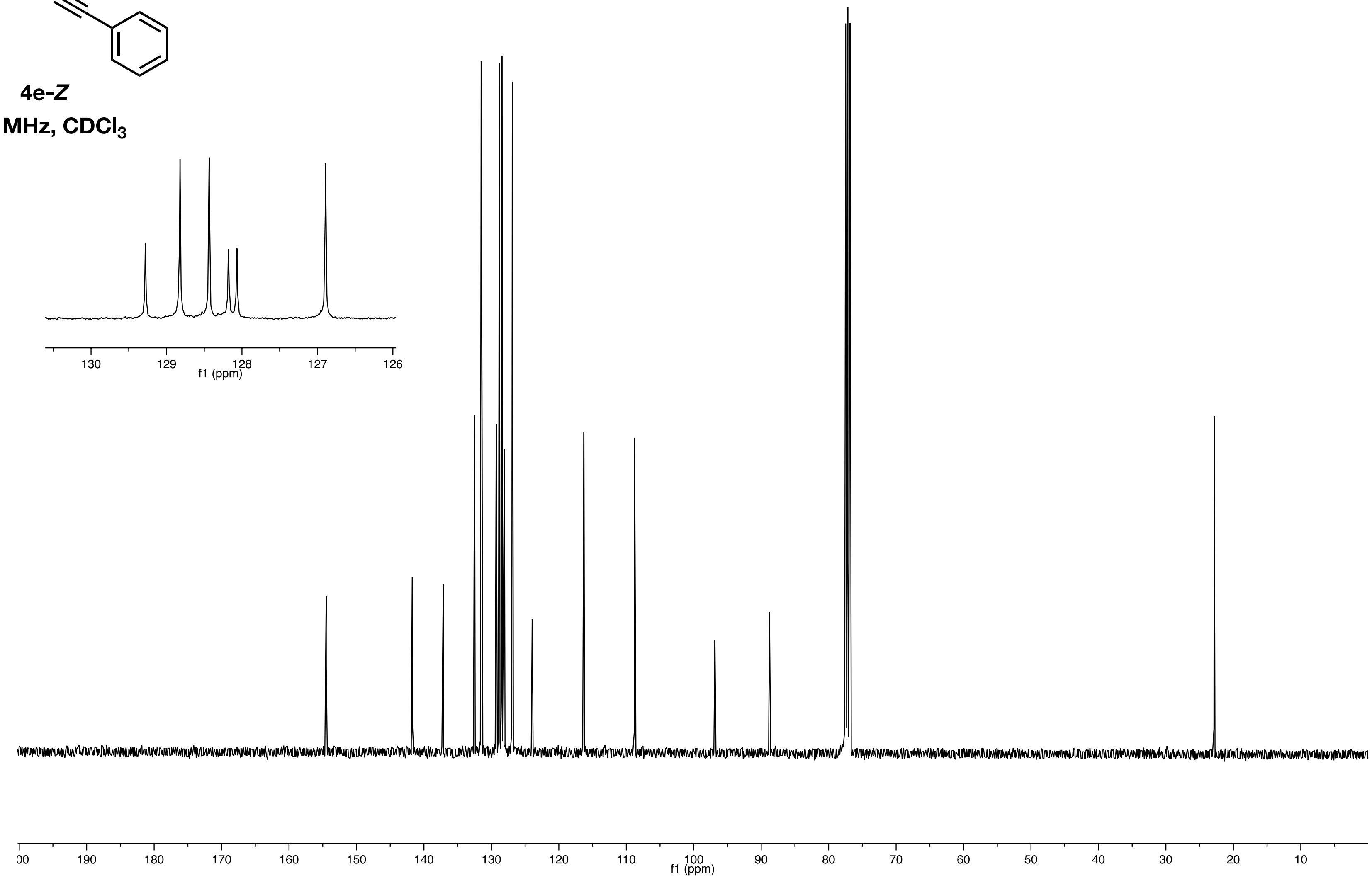

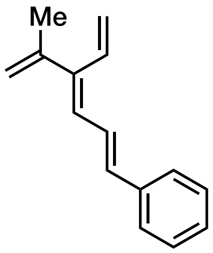

4f-*E*

400 MHz, $CDCl_3$

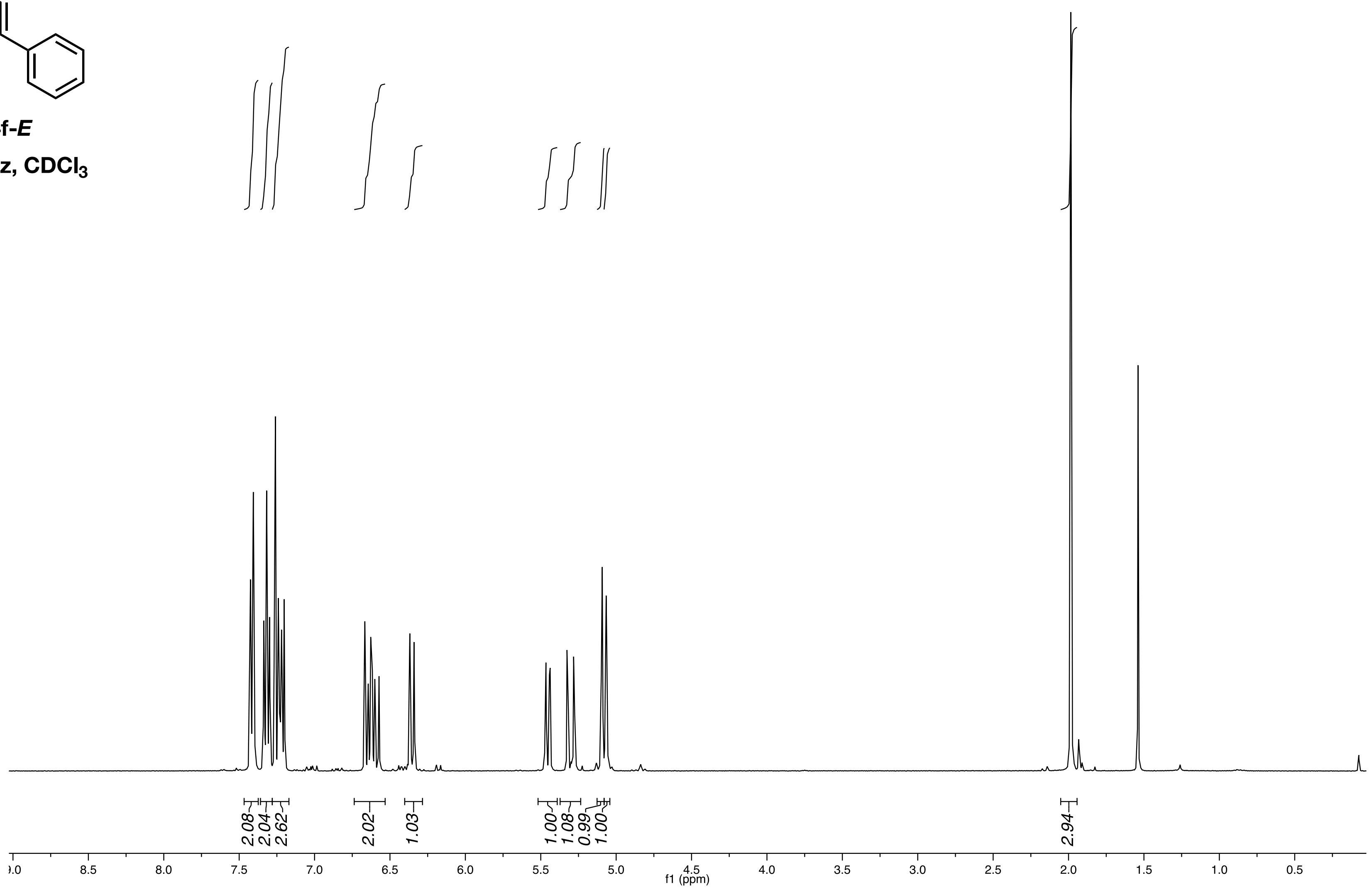

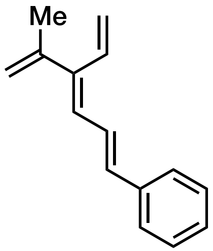

4f-*E*

100 MHz, $CDCl_3$

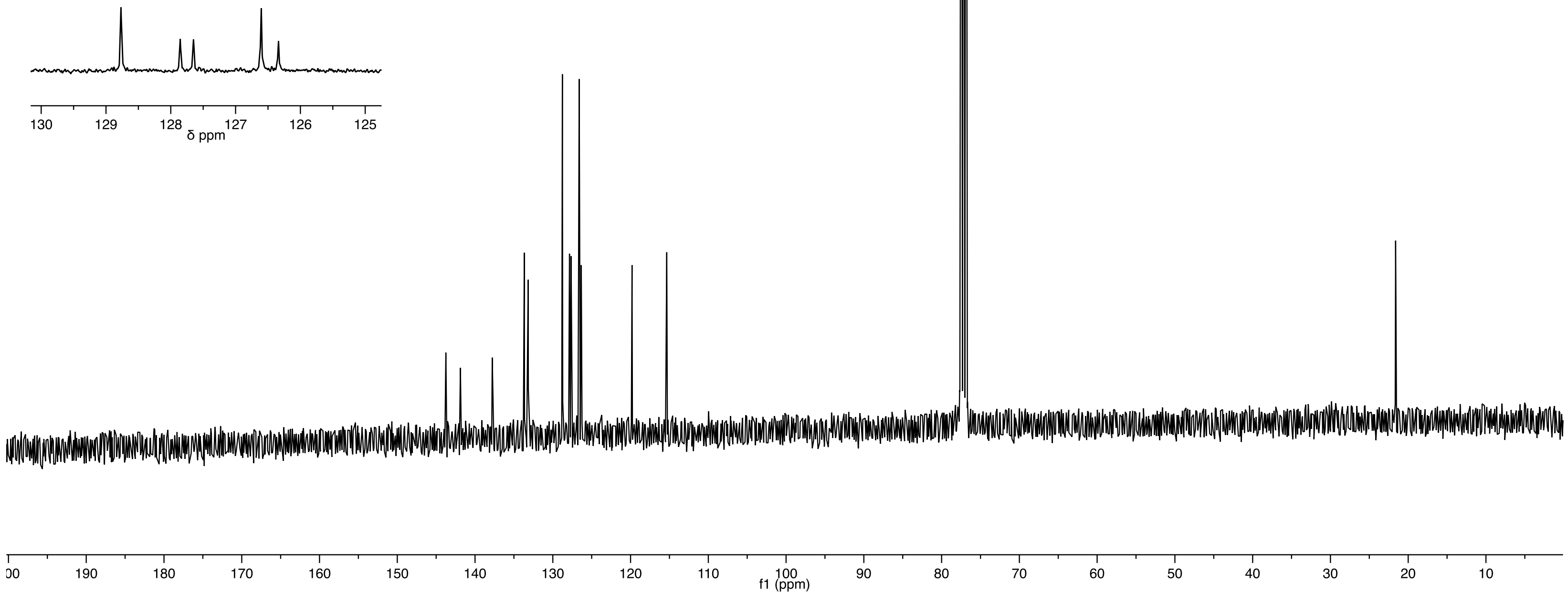

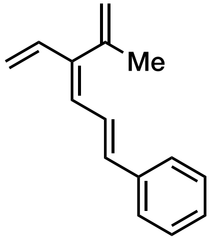

4f-*Z*

400 MHz, $CDCl_3$

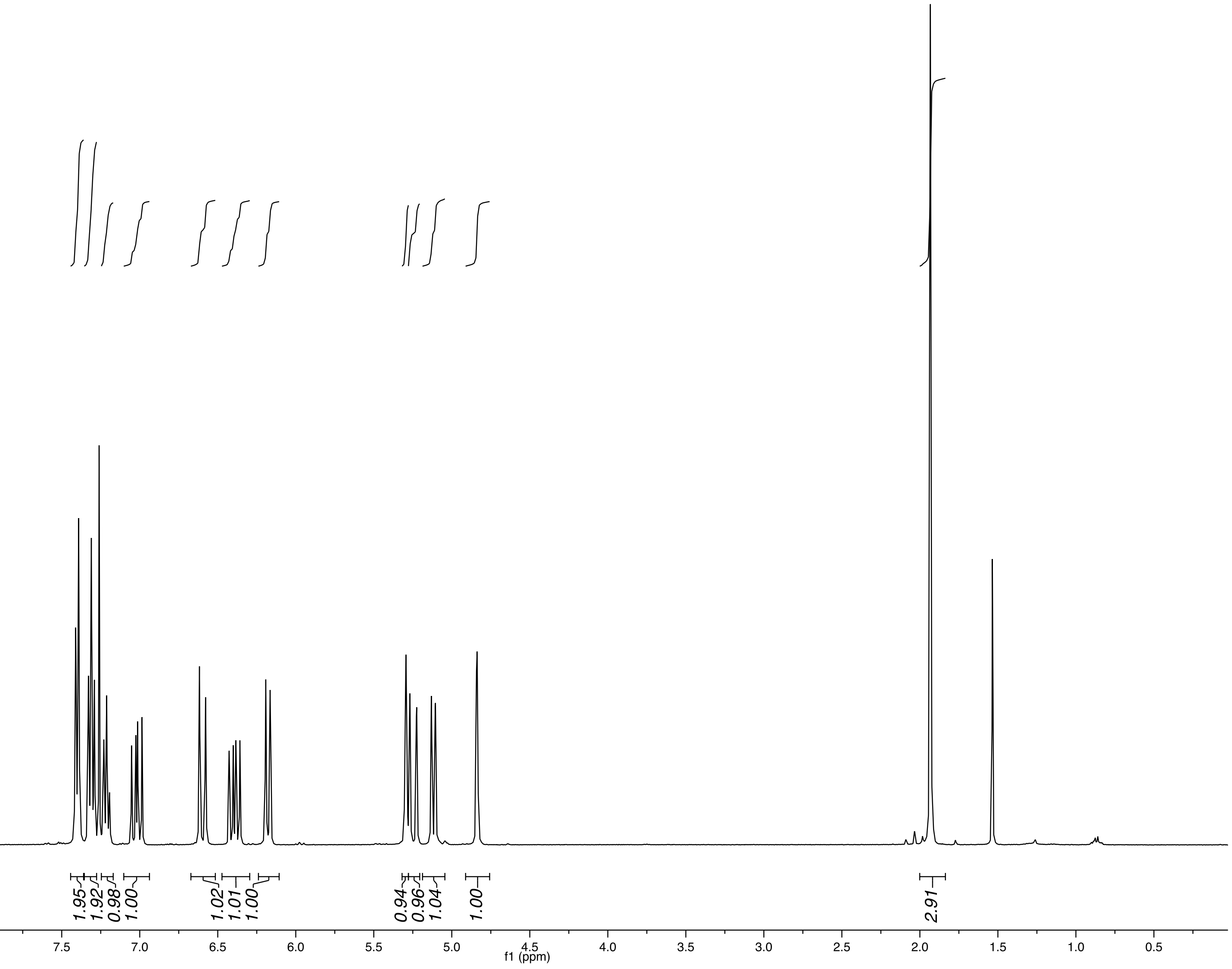

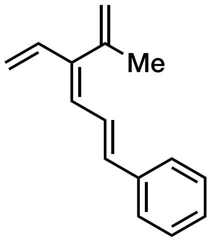

4f-*Z*

100 MHz, $CDCl_3$

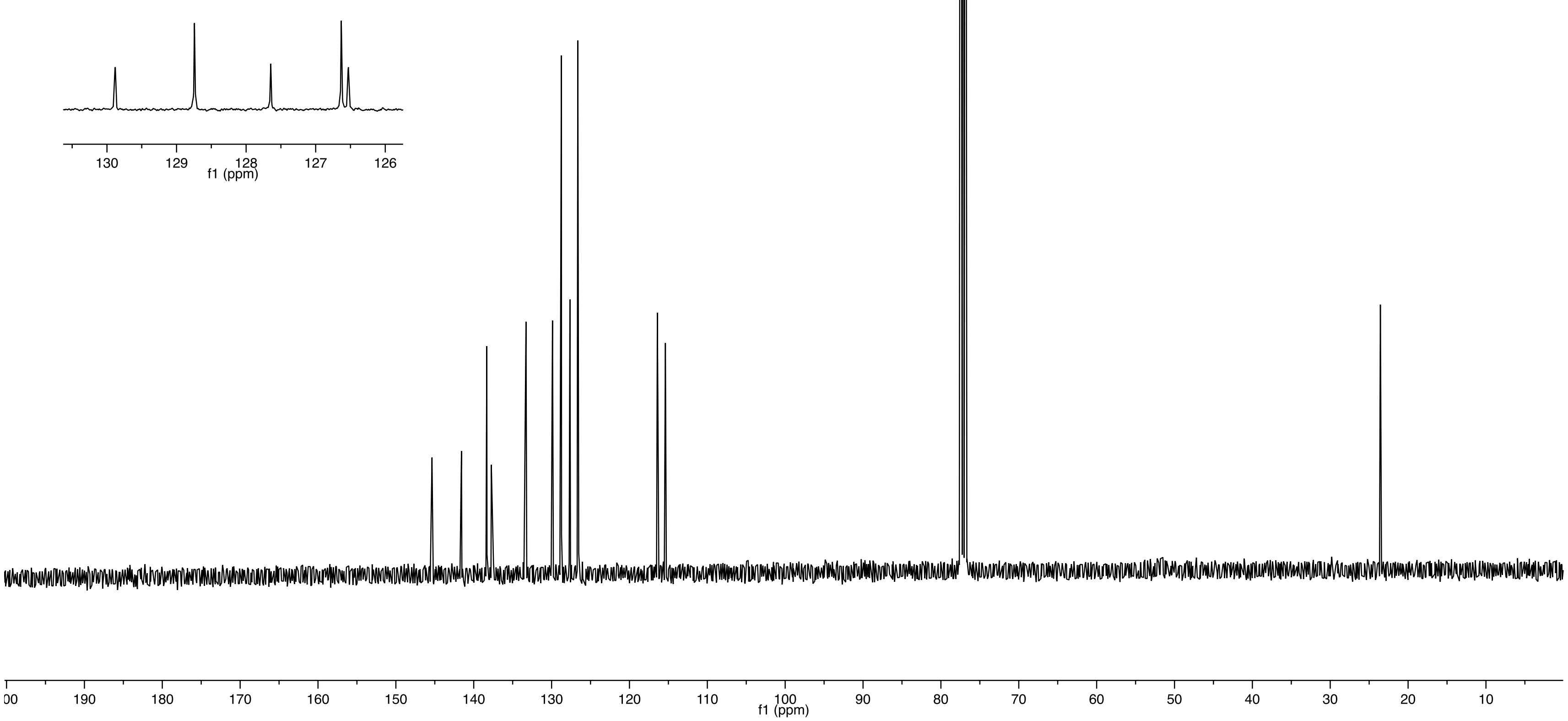

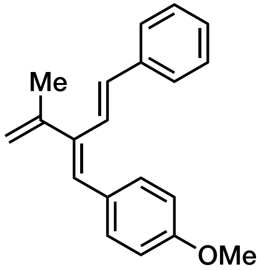

4g-*E*
400 MHz, $CDCl_3$

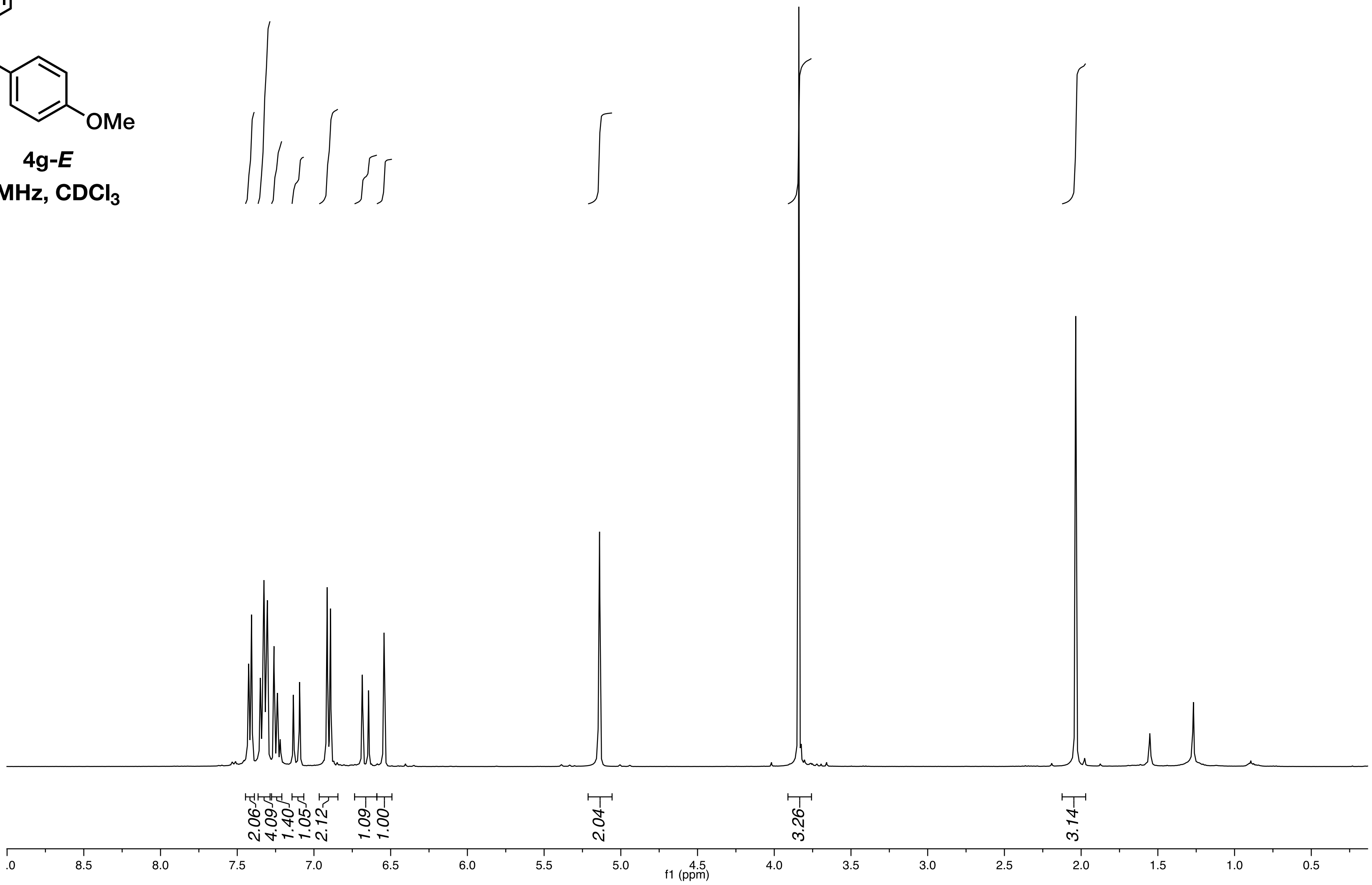

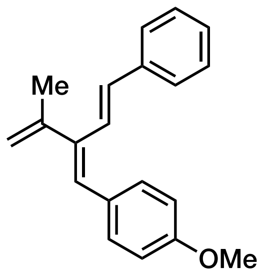

4g-*E*
100 MHz, $CDCl_3$

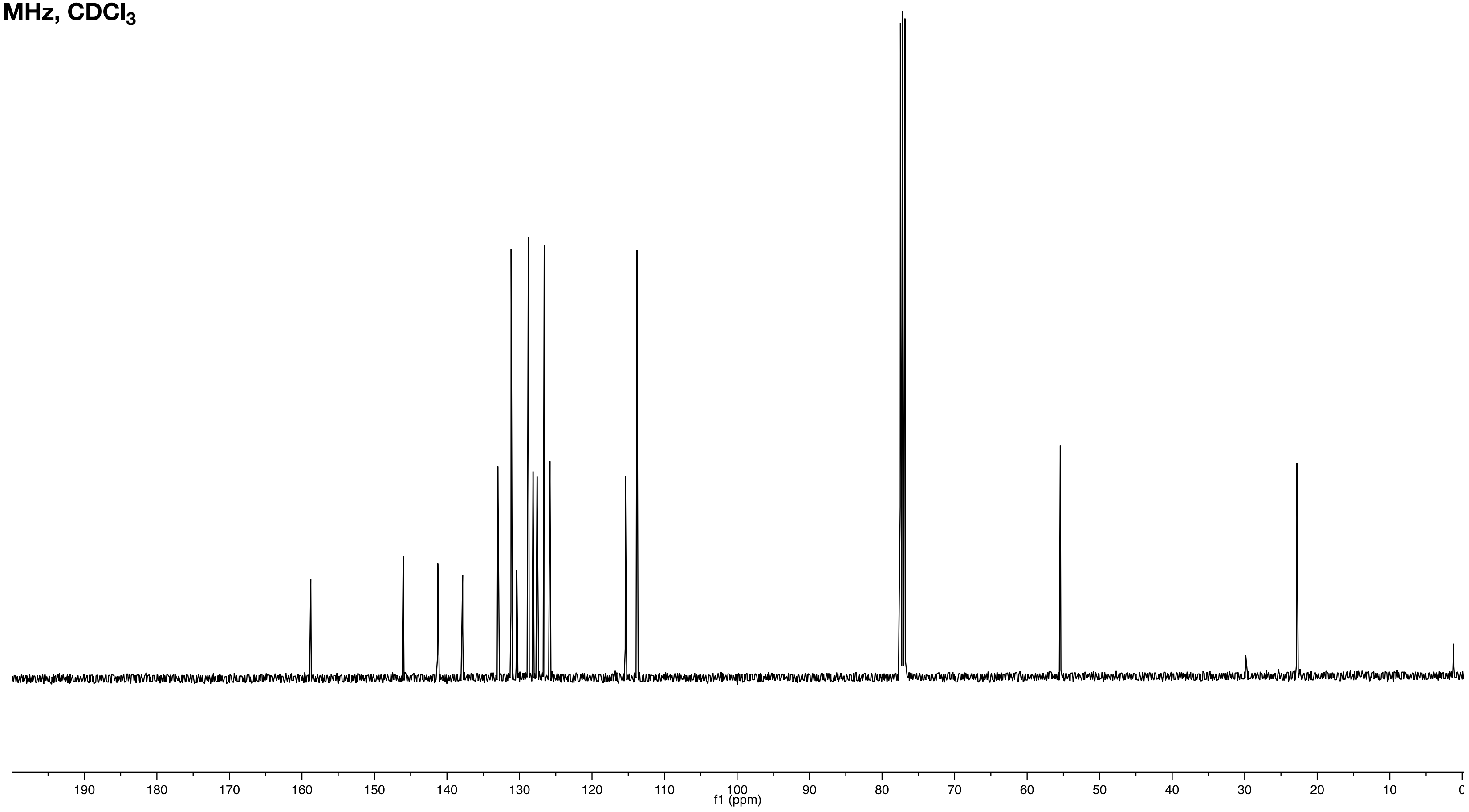

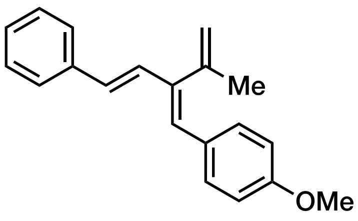

4g-*Z*

400 MHz, $CDCl_3$

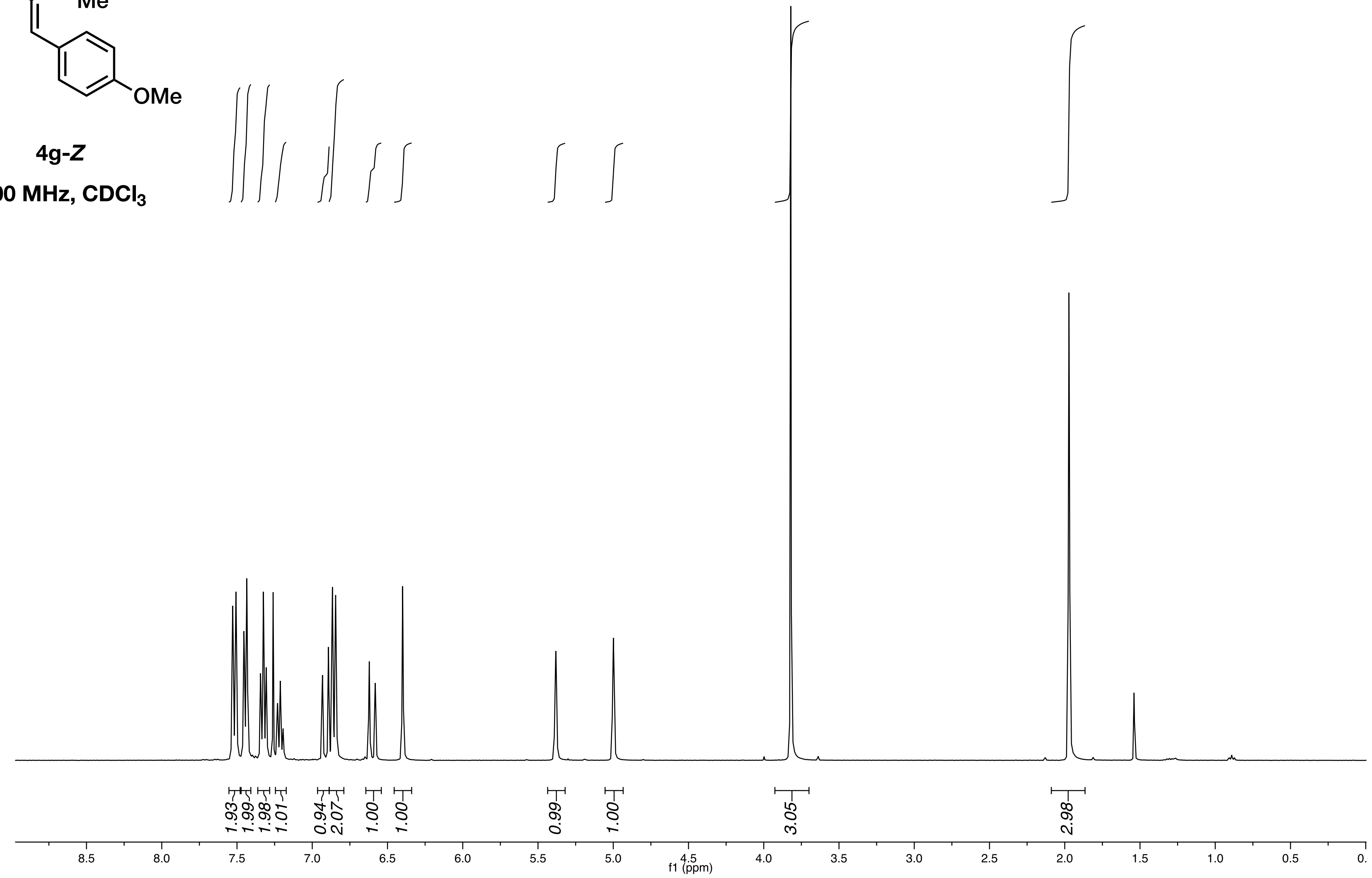

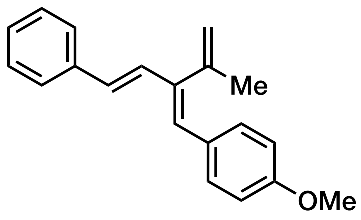

4g-*Z*

100 MHz, $CDCl_3$

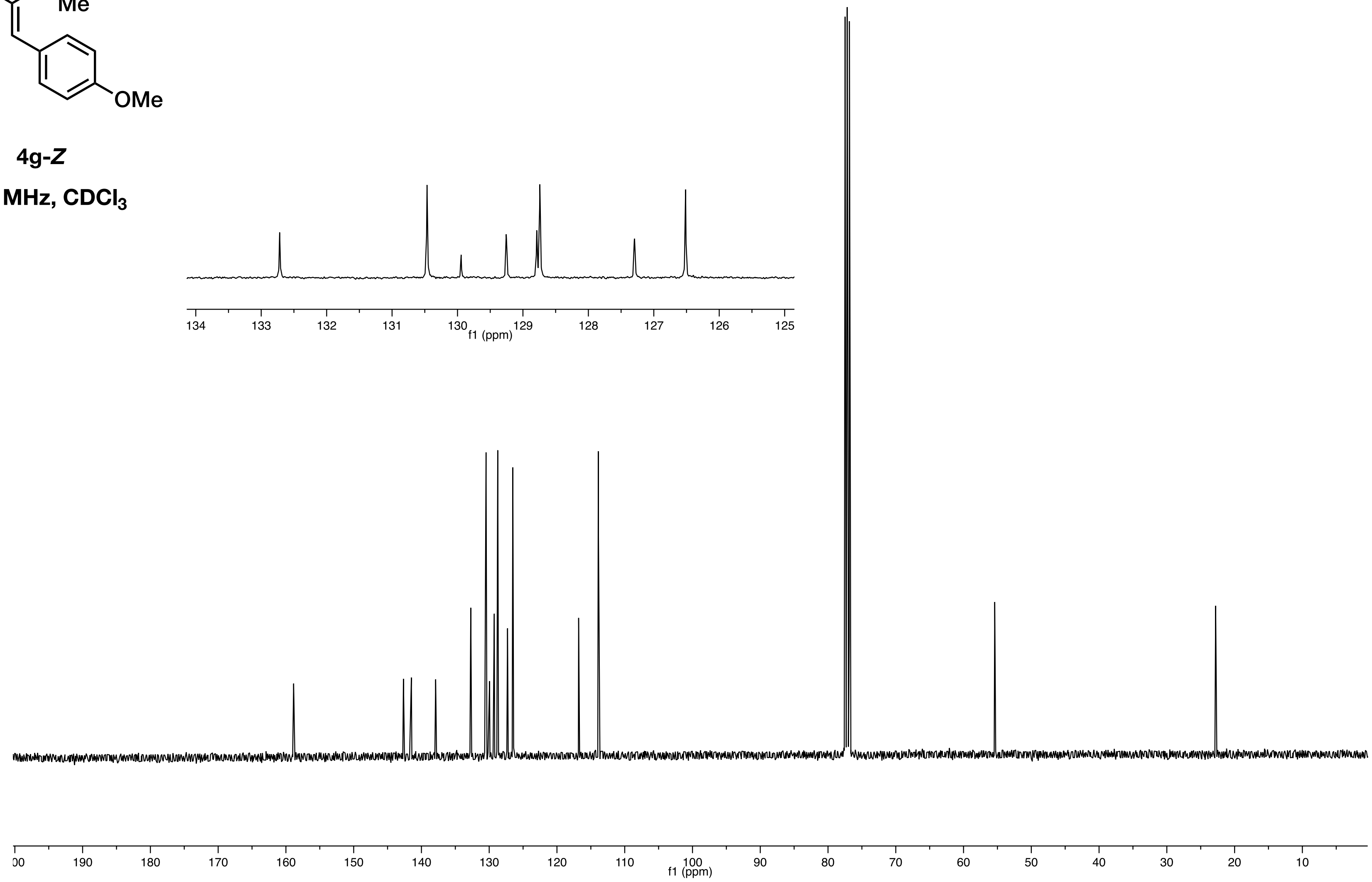

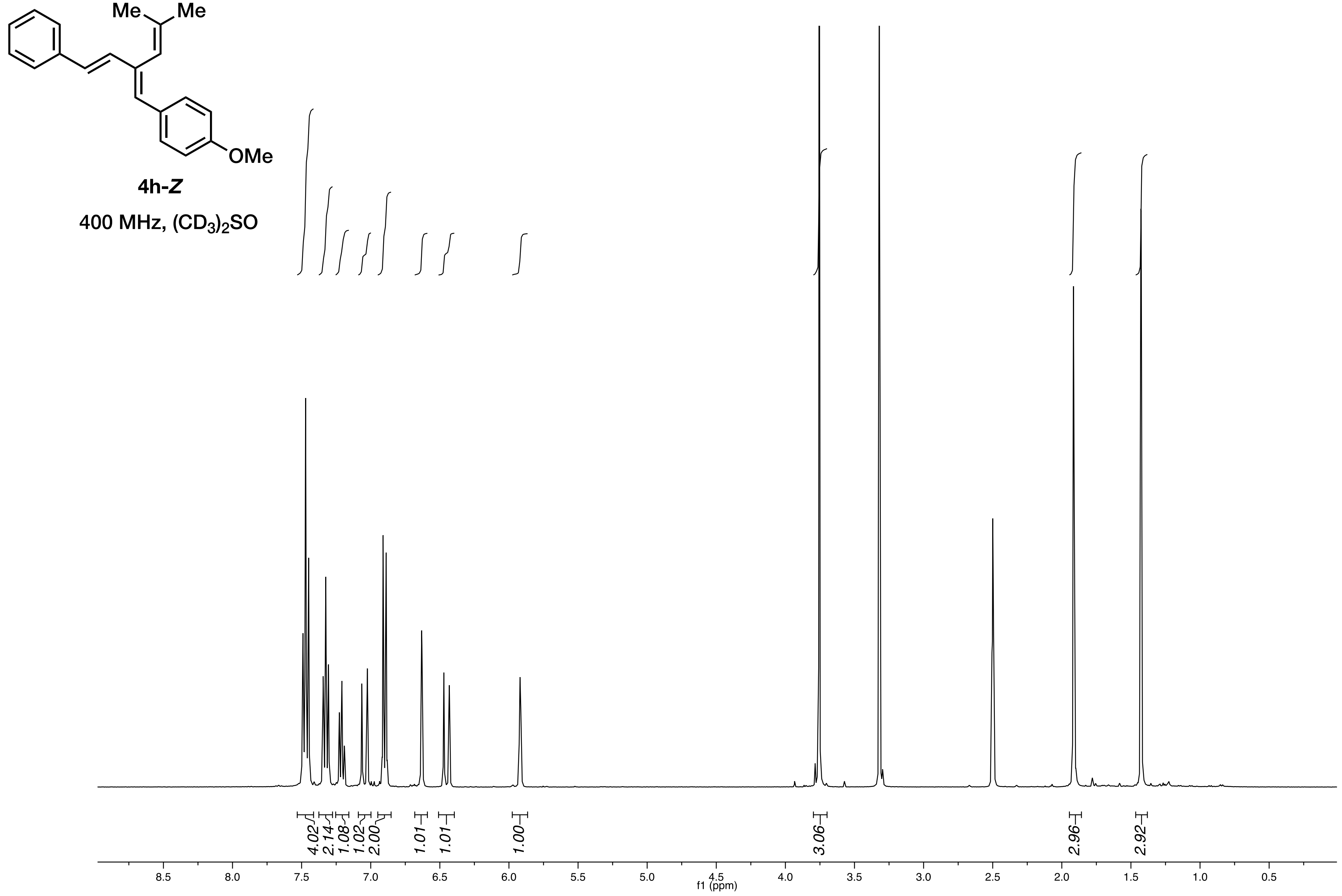

Me
Me
OMe
4h-*Z*
400 MHz, $(CD_3)_2SO$
4.02
2.14
1.08
1.02
2.00
1.01
1.01
1.00
3.06
2.96
2.92
8.5
8.0
7.5
7.0
6.5
6.0
5.5
5.0
4.5
4.0
3.5
3.0
2.5
2.0
1.5
1.0
0.5
f1 (ppm)

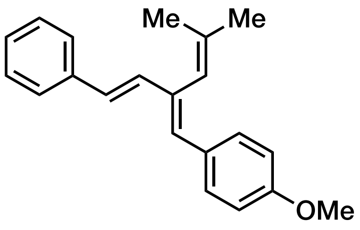

4h-*Z*

100 MHz, $(CD_3)_2SO$

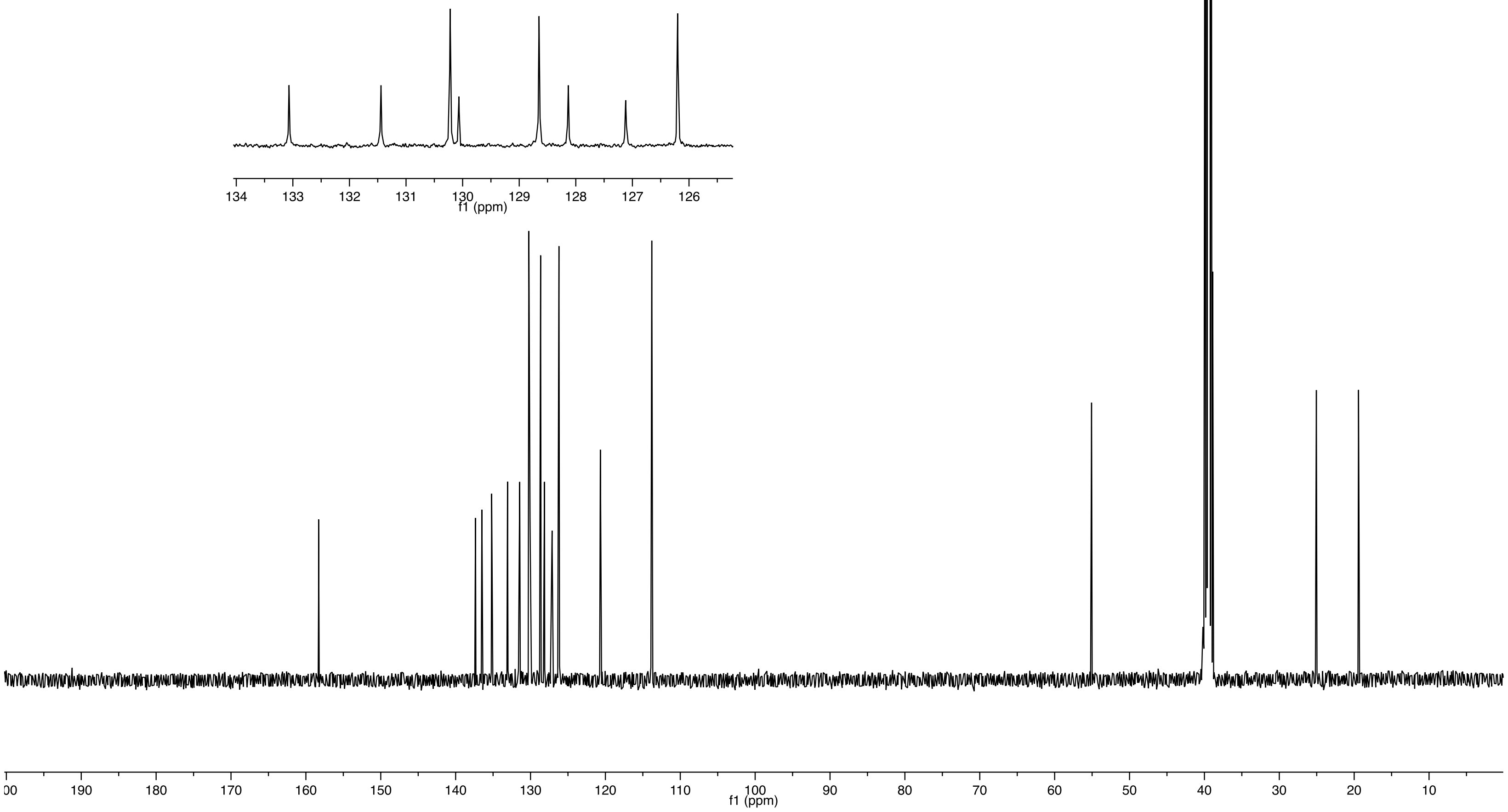

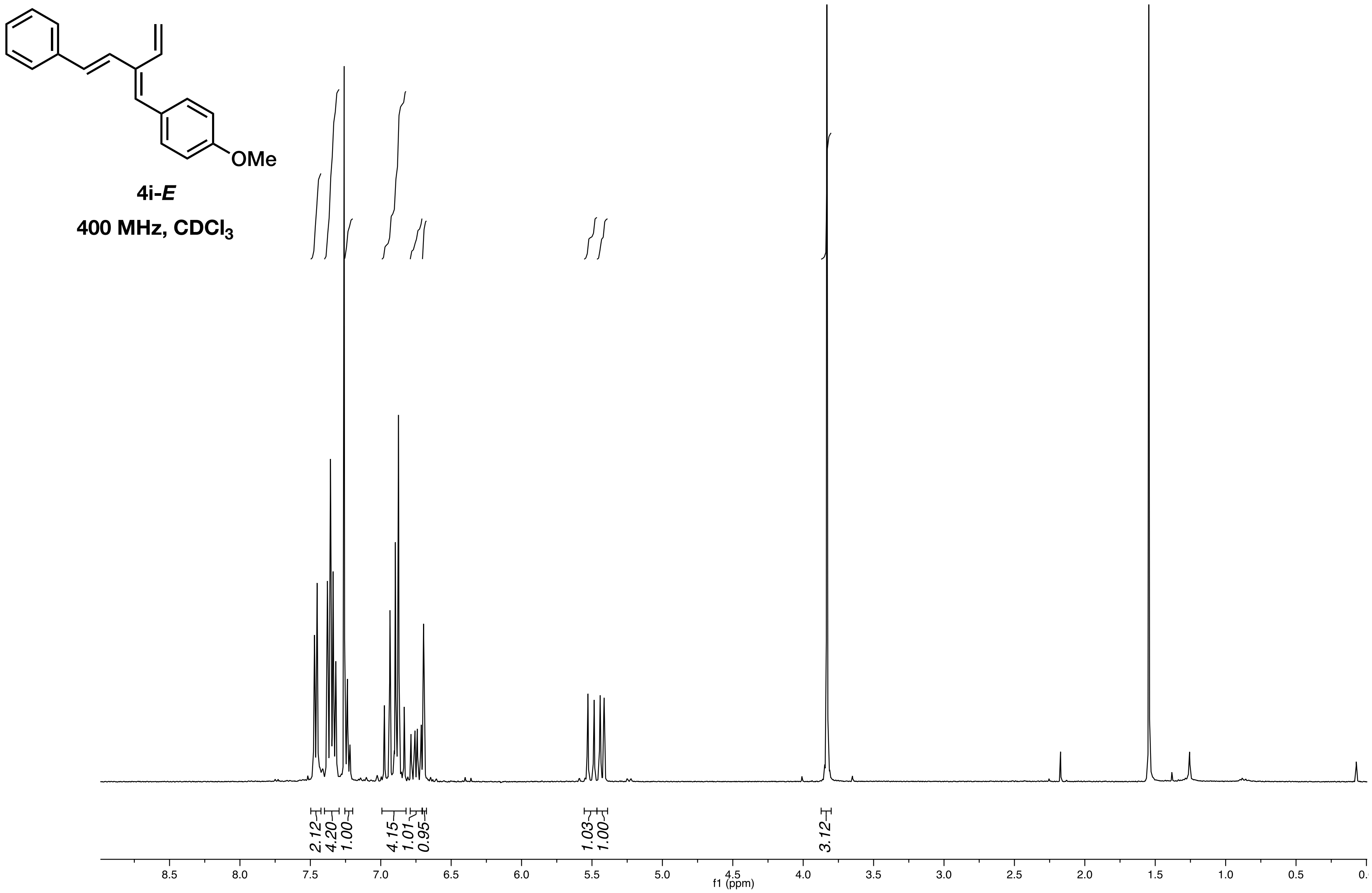

OMe
4i-*E*
400 MHz, CDCl3
2.12
4.20
1.00
4.15
1.01
0.95
1.03
1.00
3.12
8.5
8.0
7.5
7.0
6.5
6.0
5.5
5.0
4.5
4.0
3.5
3.0
2.5
2.0
1.5
1.0
0.5
f1 (ppm)

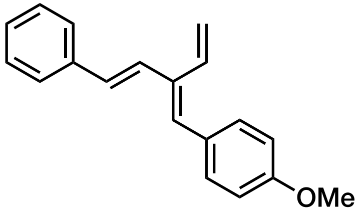

4i-*E*

100 MHz, $CDCl_3$

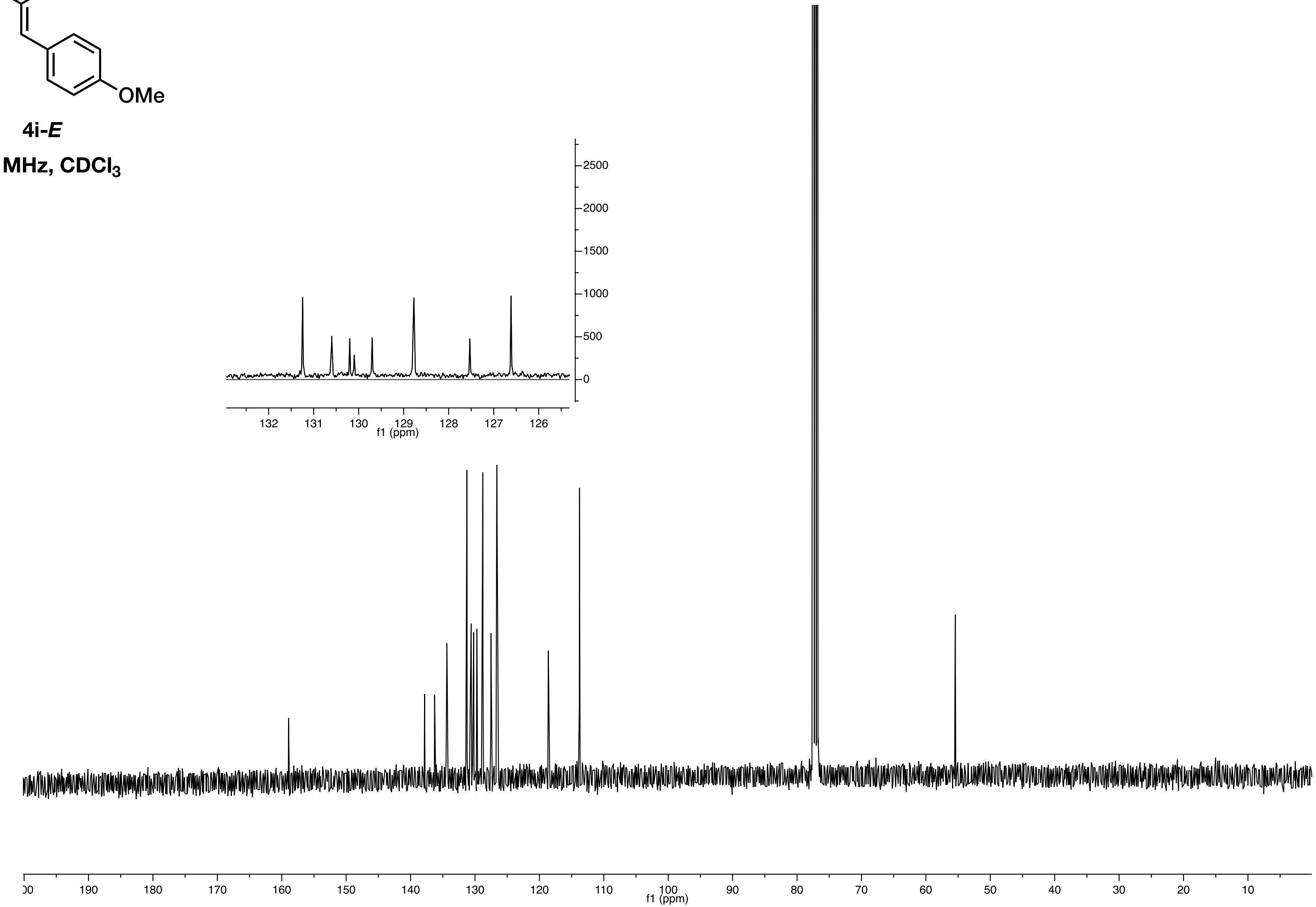

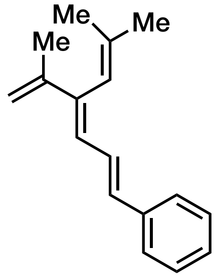

4j-*E*

400 MHz, $CDCl_3$

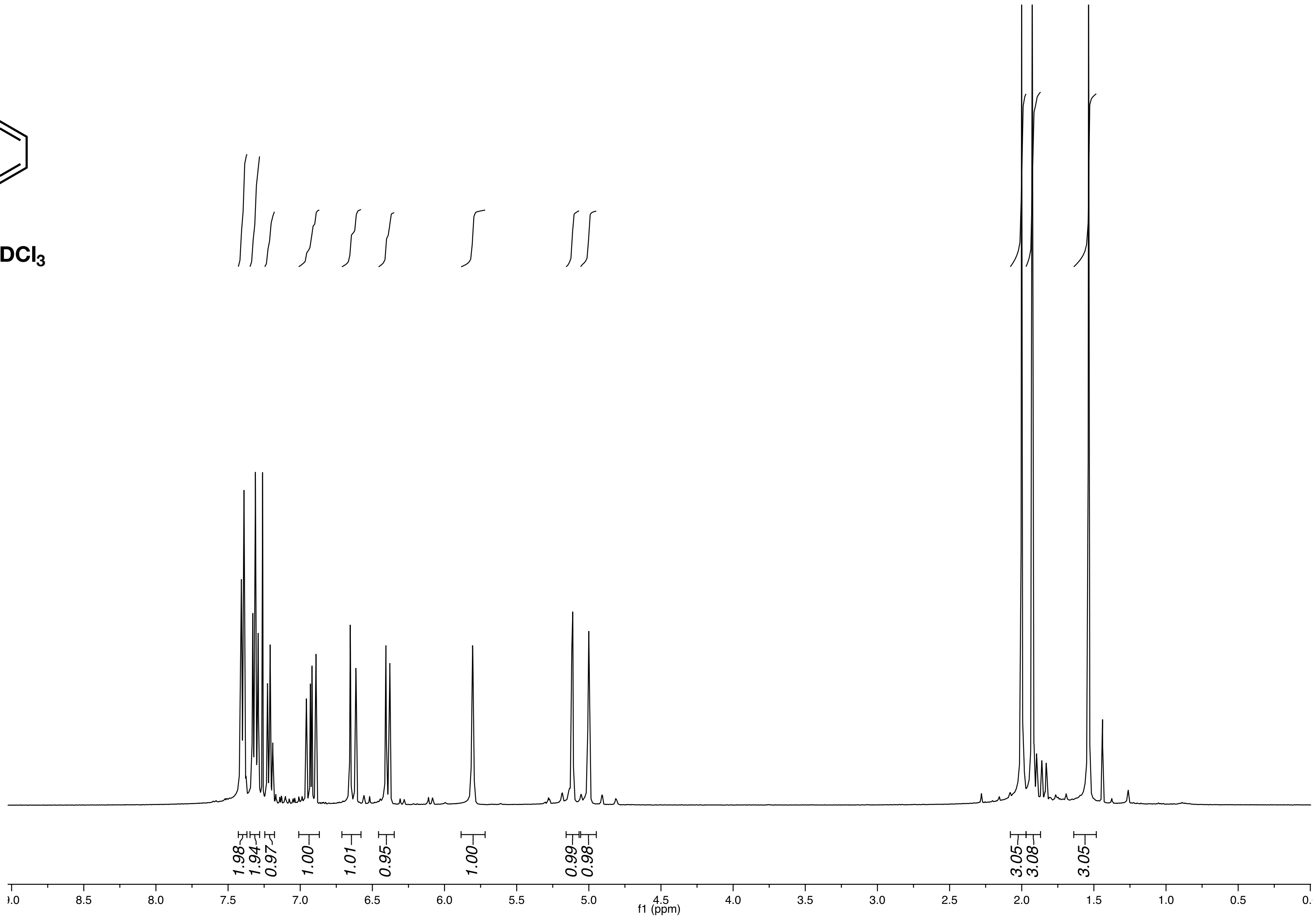

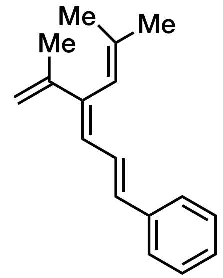

4j-*E*

100 MHz, $CDCl_3$

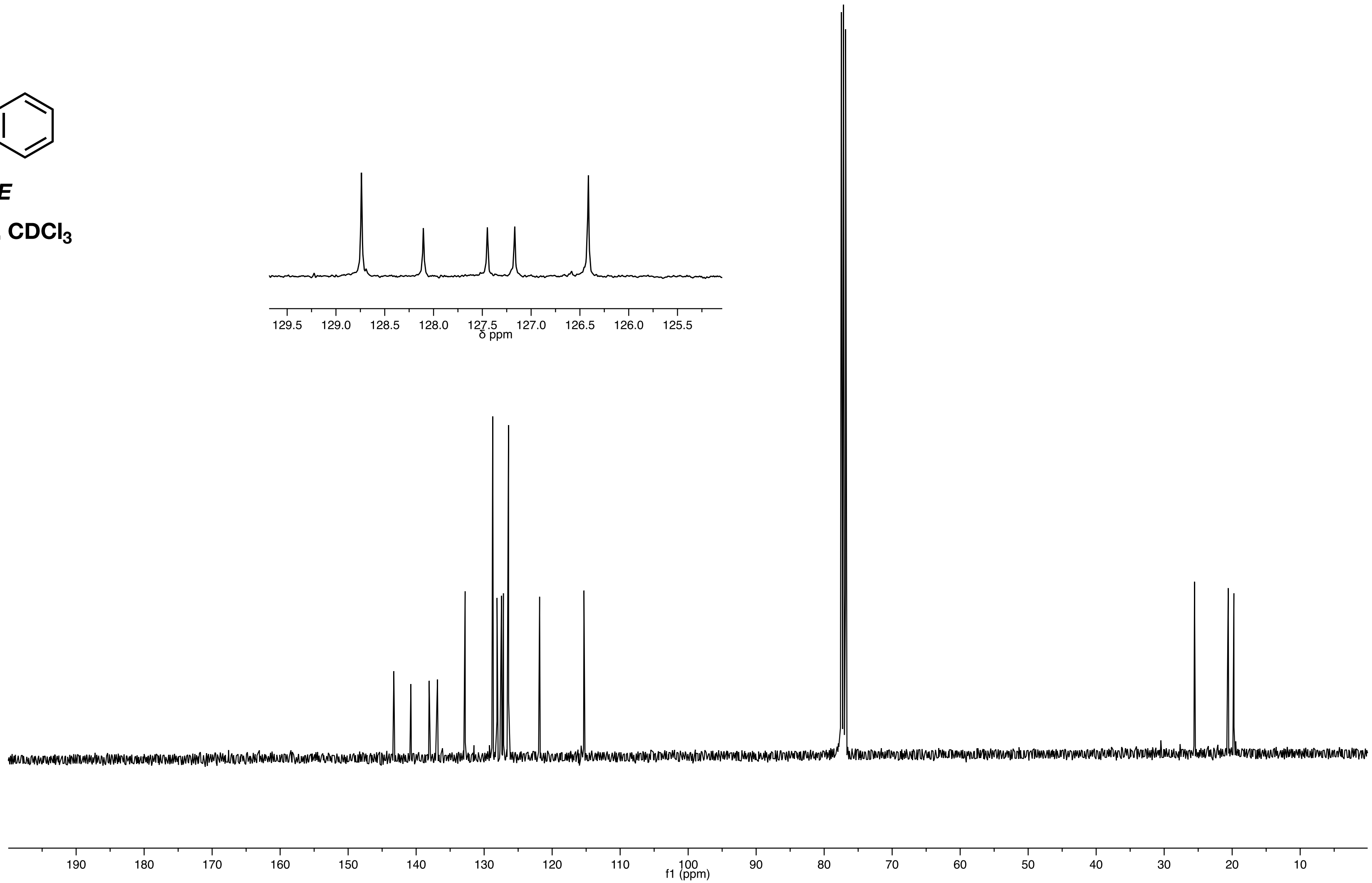

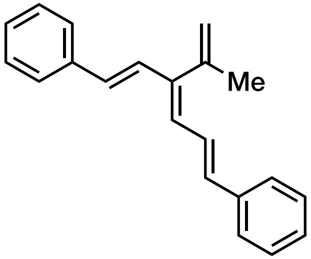

4l-*Z*

400 MHz, $CDCl_3$

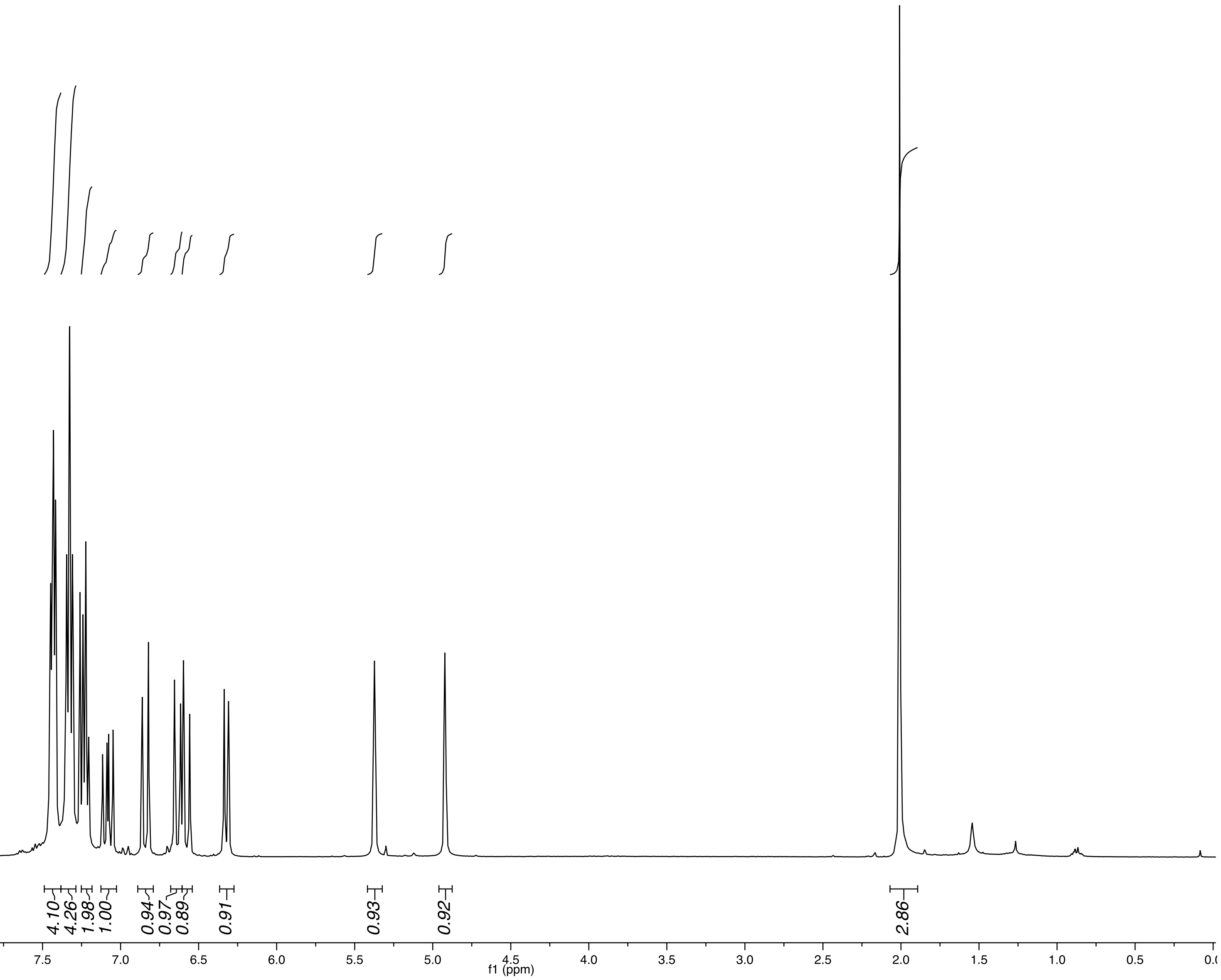

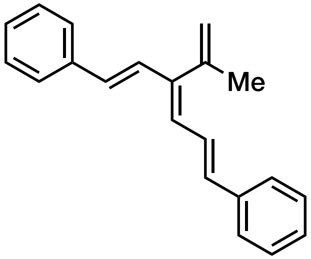

4l-*Z*

100 MHz, $CDCl_3$

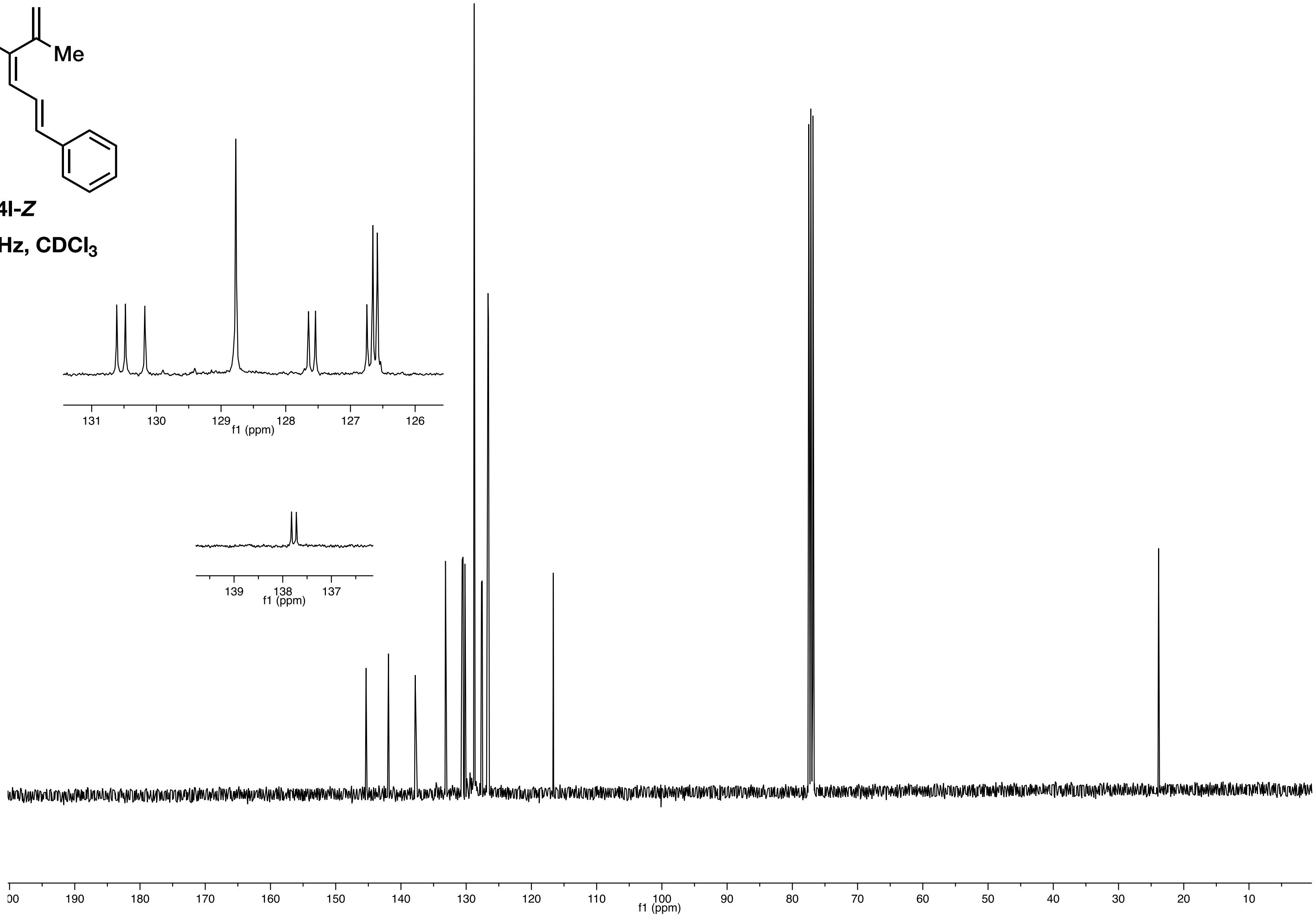

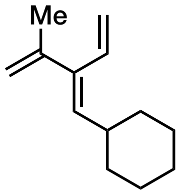

4m-*E*

400 MHz, $CDCl_3$

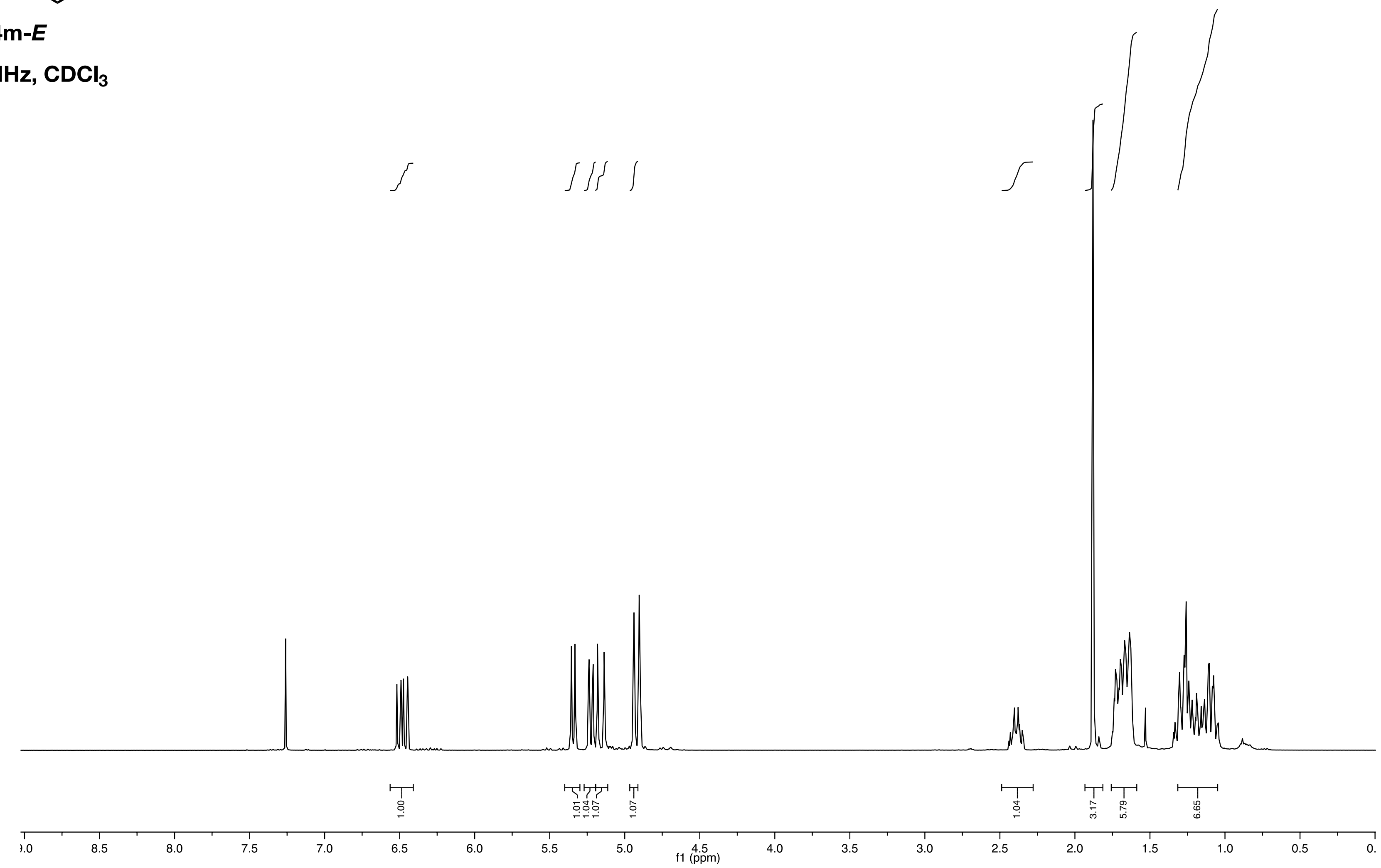

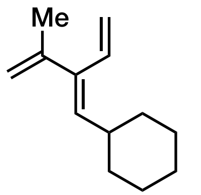

4m-*E*

100 MHz, $CDCl_3$

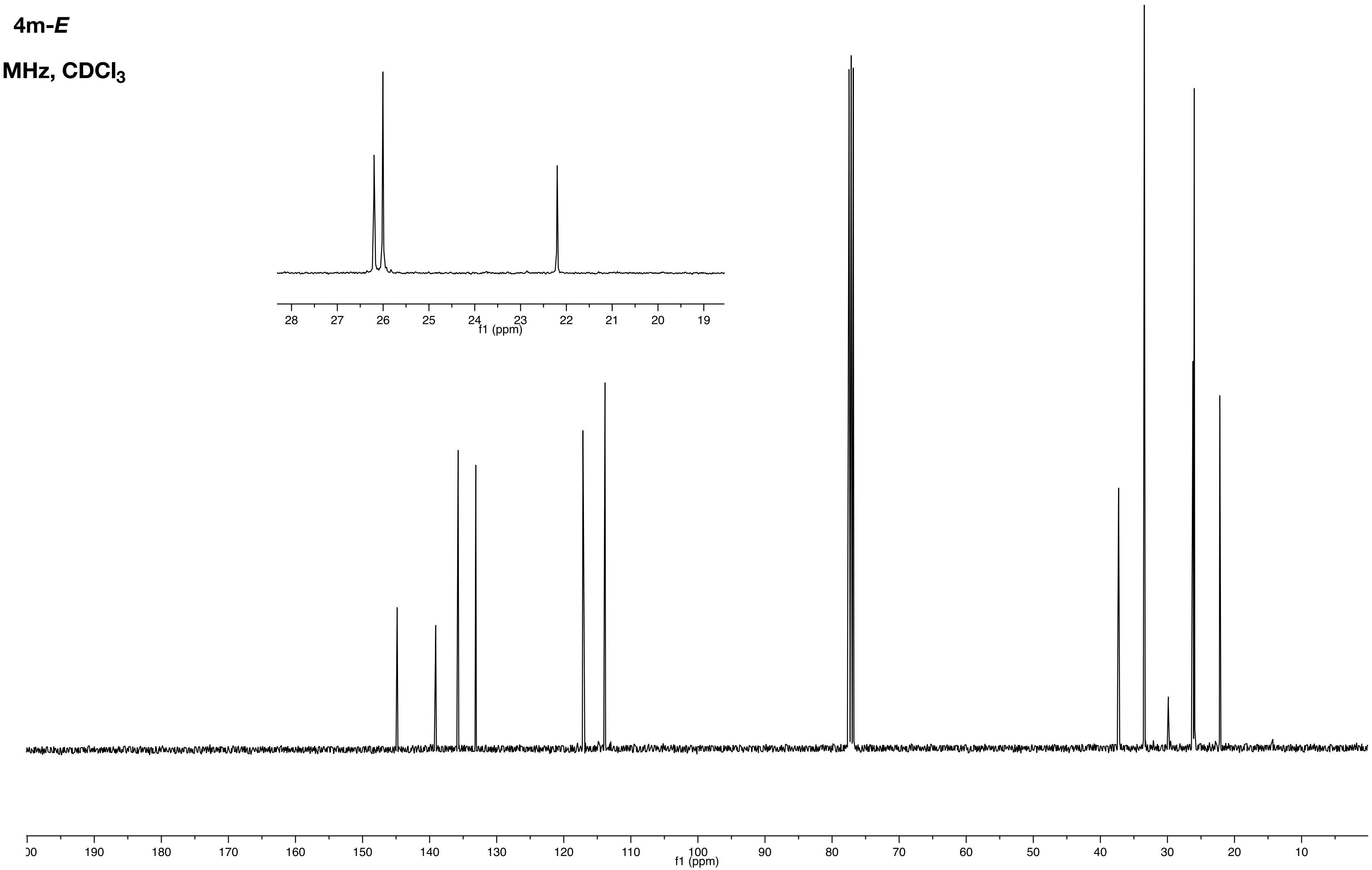

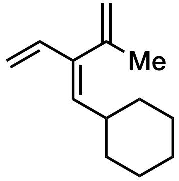

4m-*Z*

400 MHz, $CDCl_3$

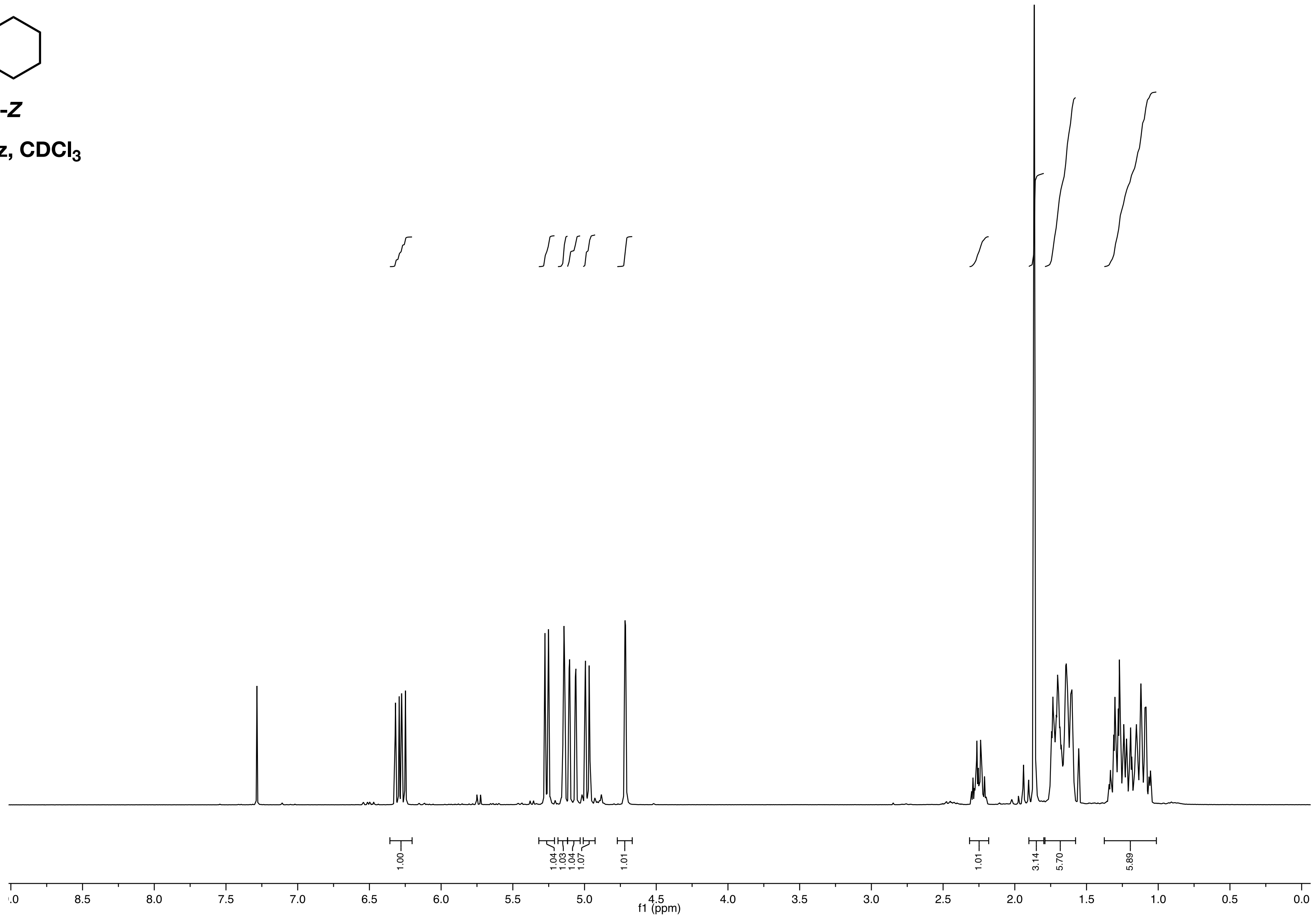

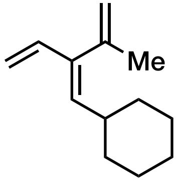

4m-*Z*

100 MHz, $CDCl_3$

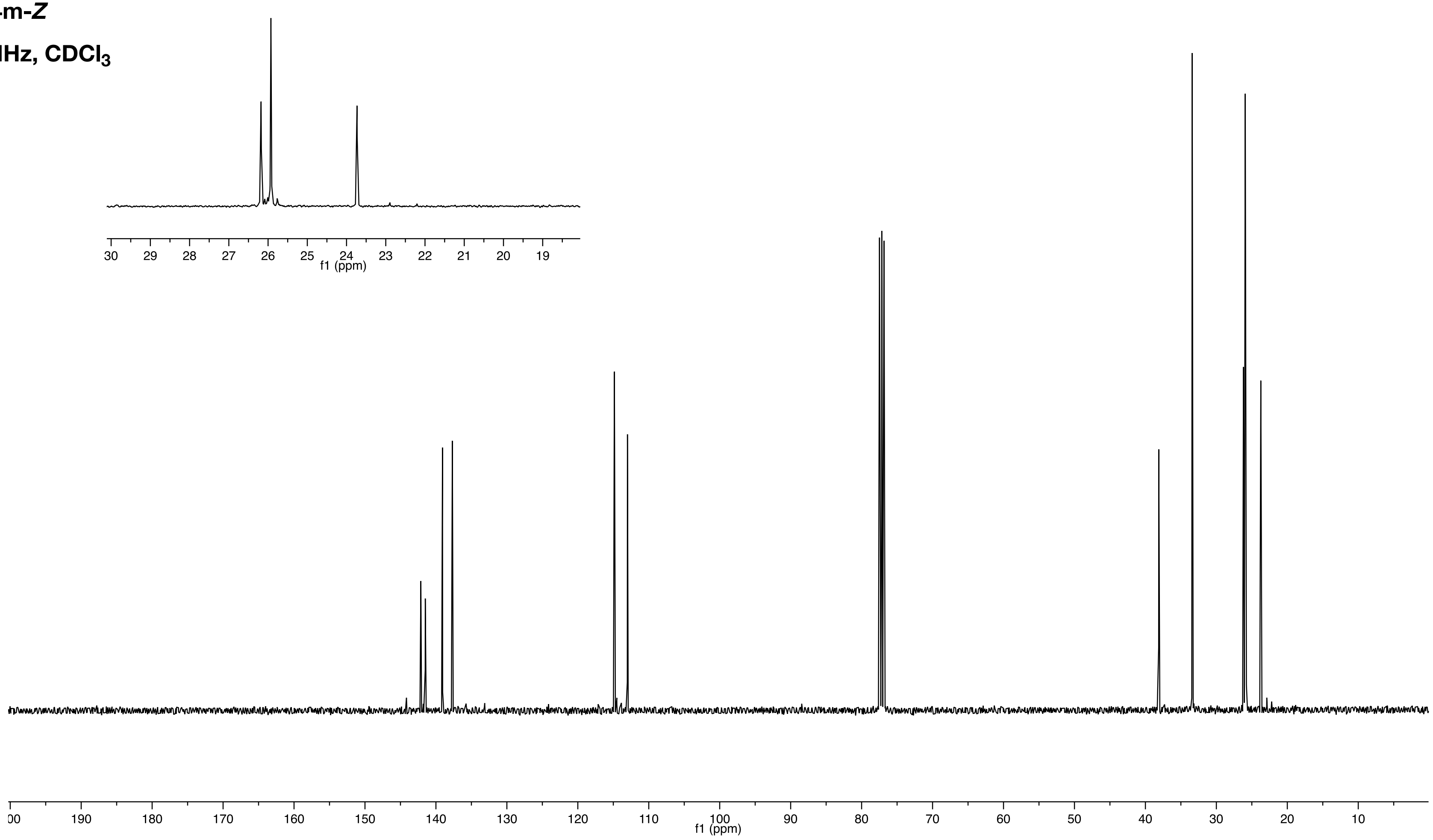

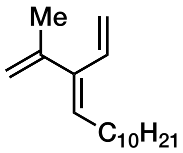

4k-*E*

400 MHz, $CDCl_3$

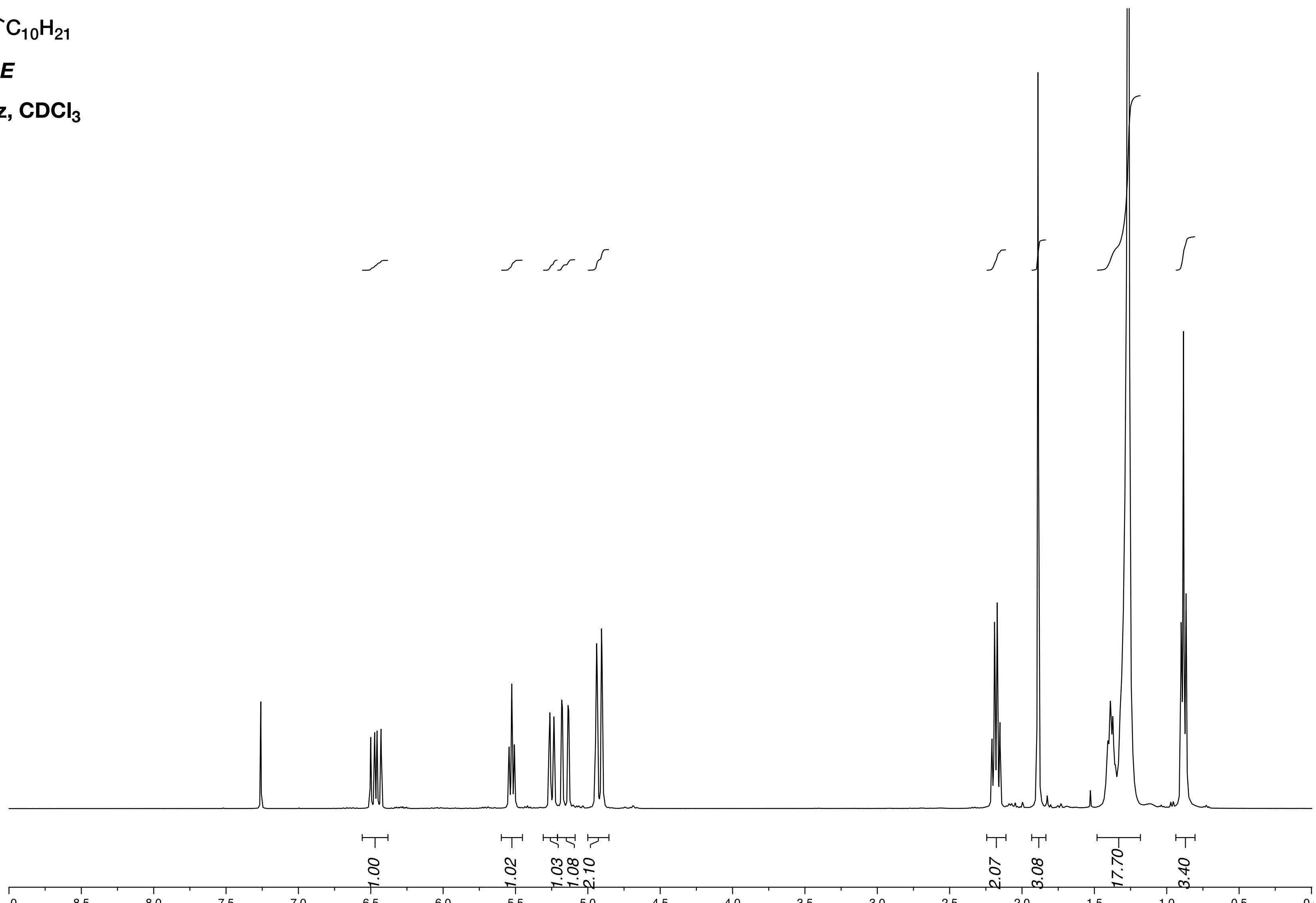

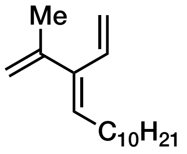

4k-*E*

100 MHz, $CDCl_3$

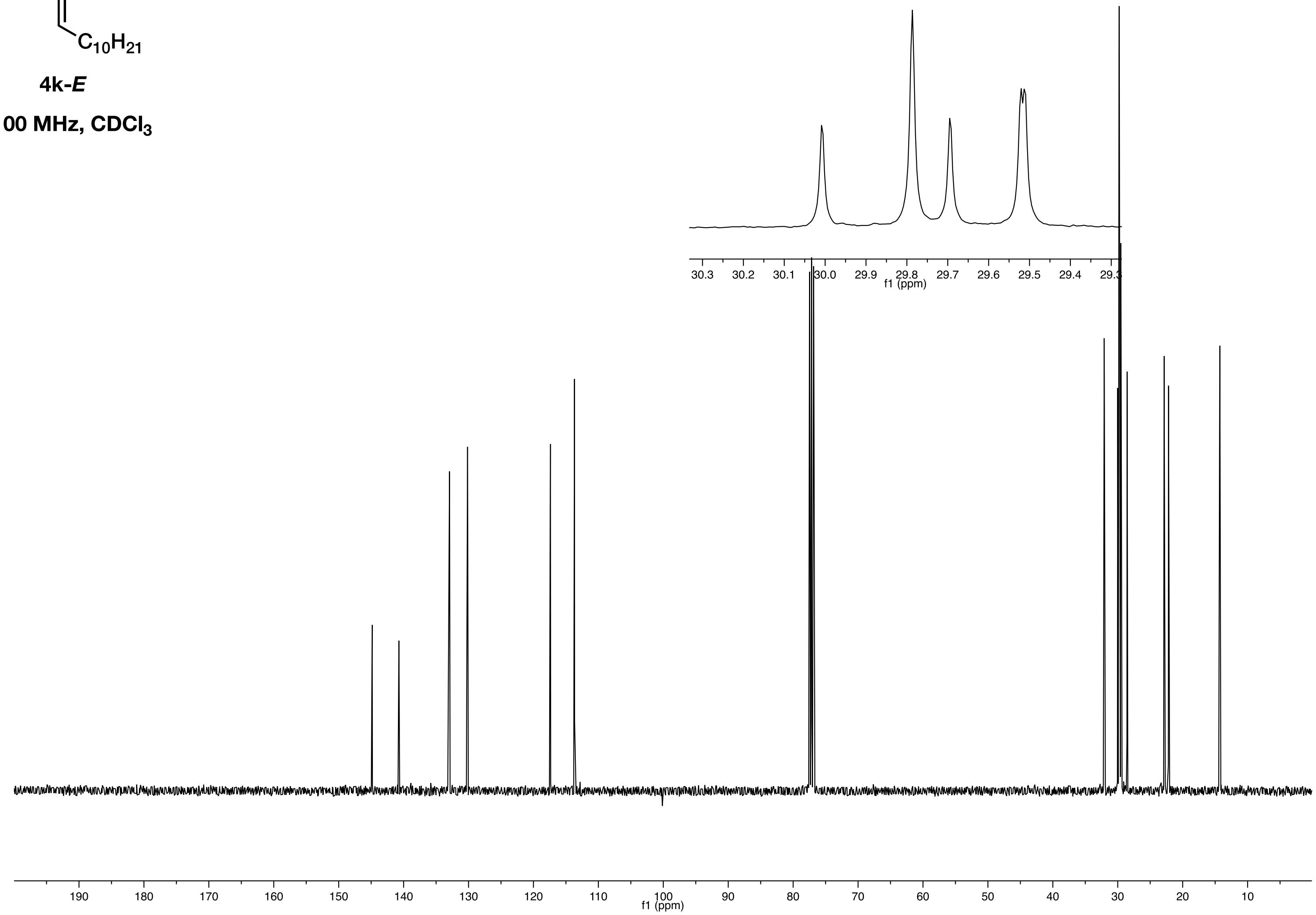

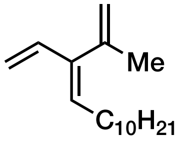

4k-*Z*

400 MHz, $CDCl_3$

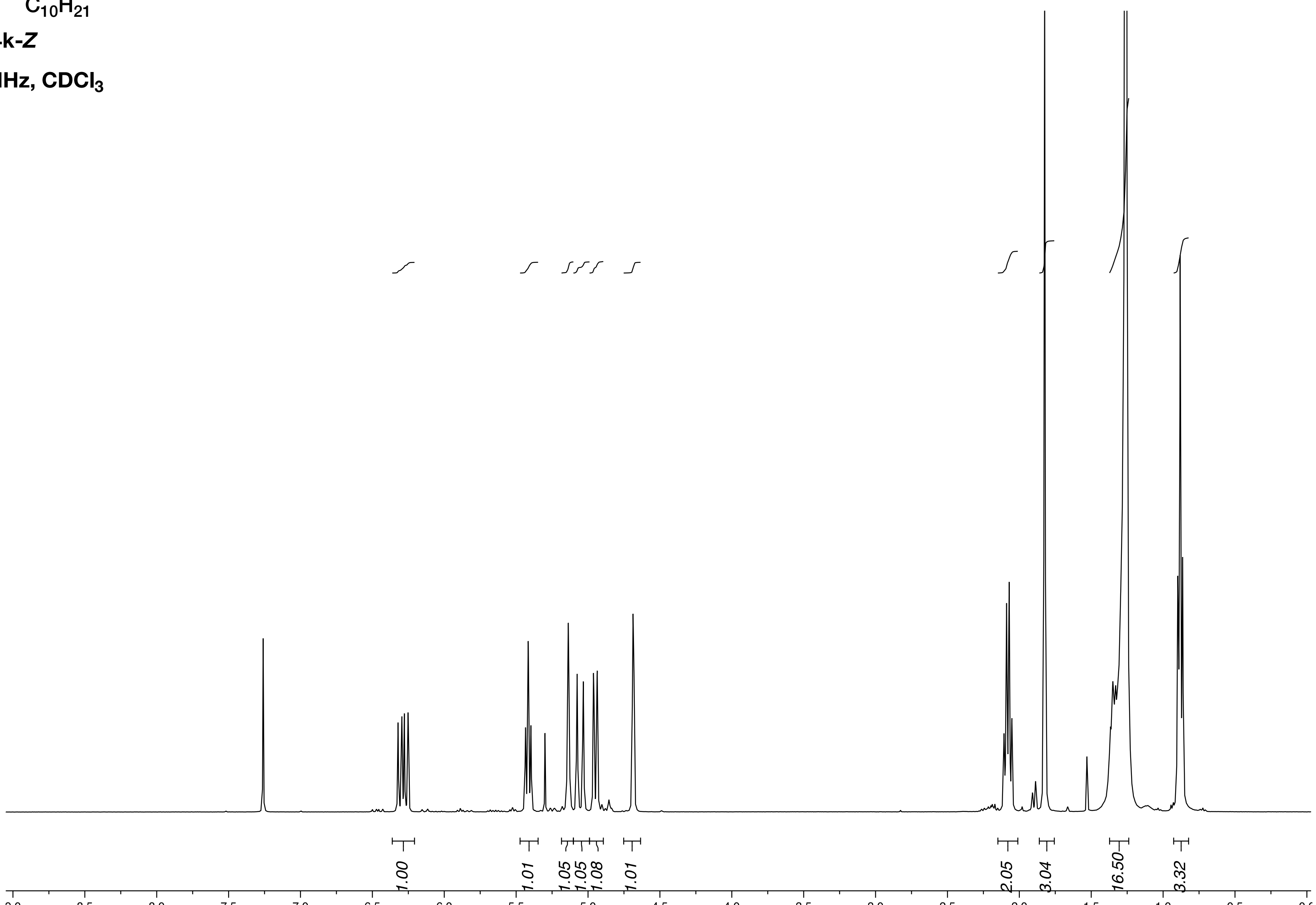

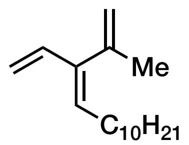

4k-*Z*

100 MHz, $CDCl_3$

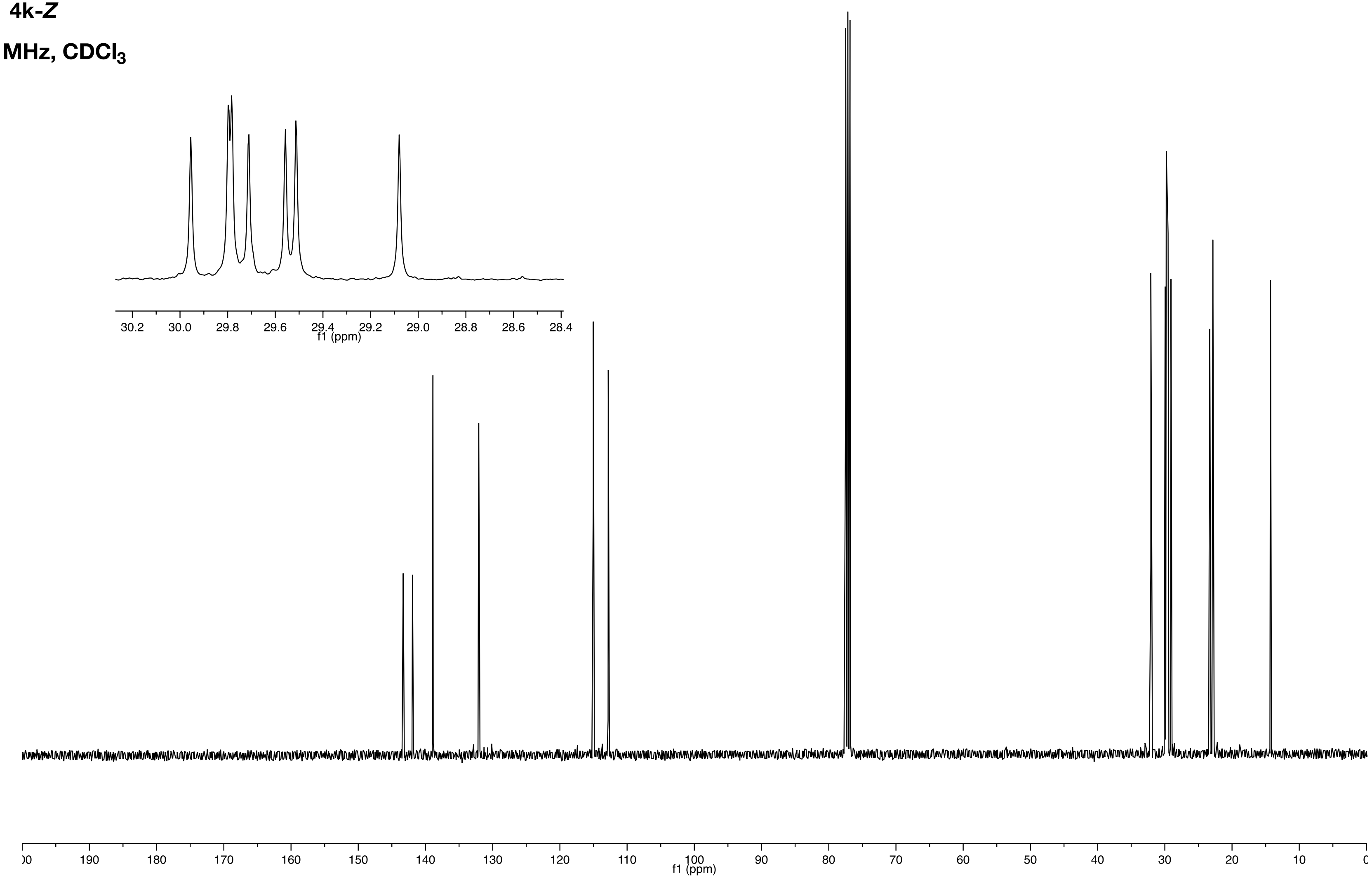

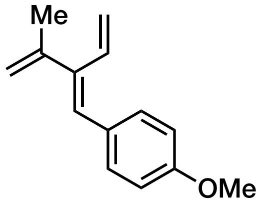

4n-*E*

400 MHz, $CDCl_3$

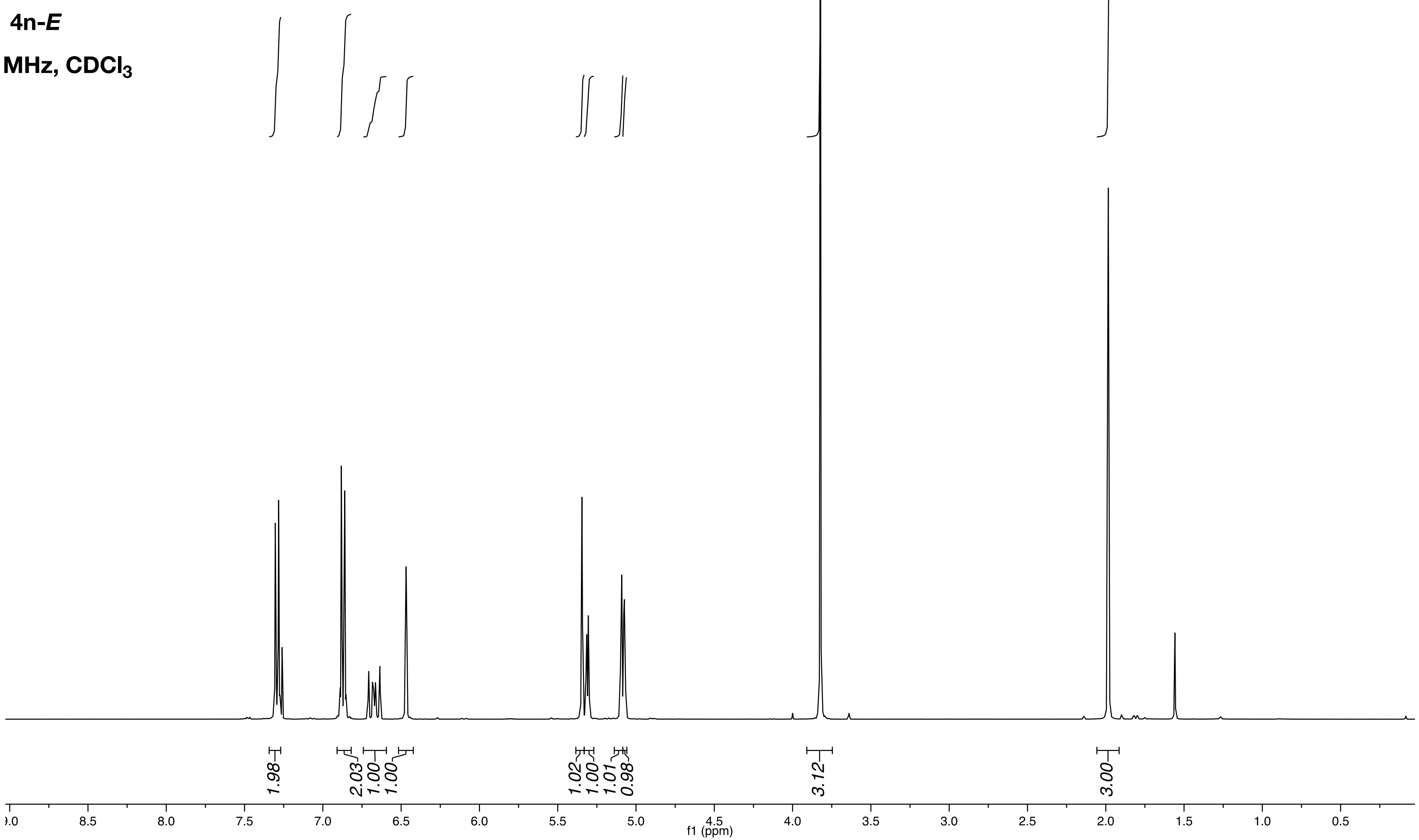

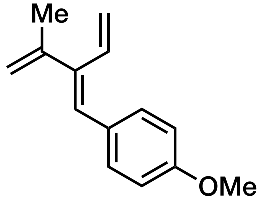

4n-*E*

100 MHz, $CDCl_3$

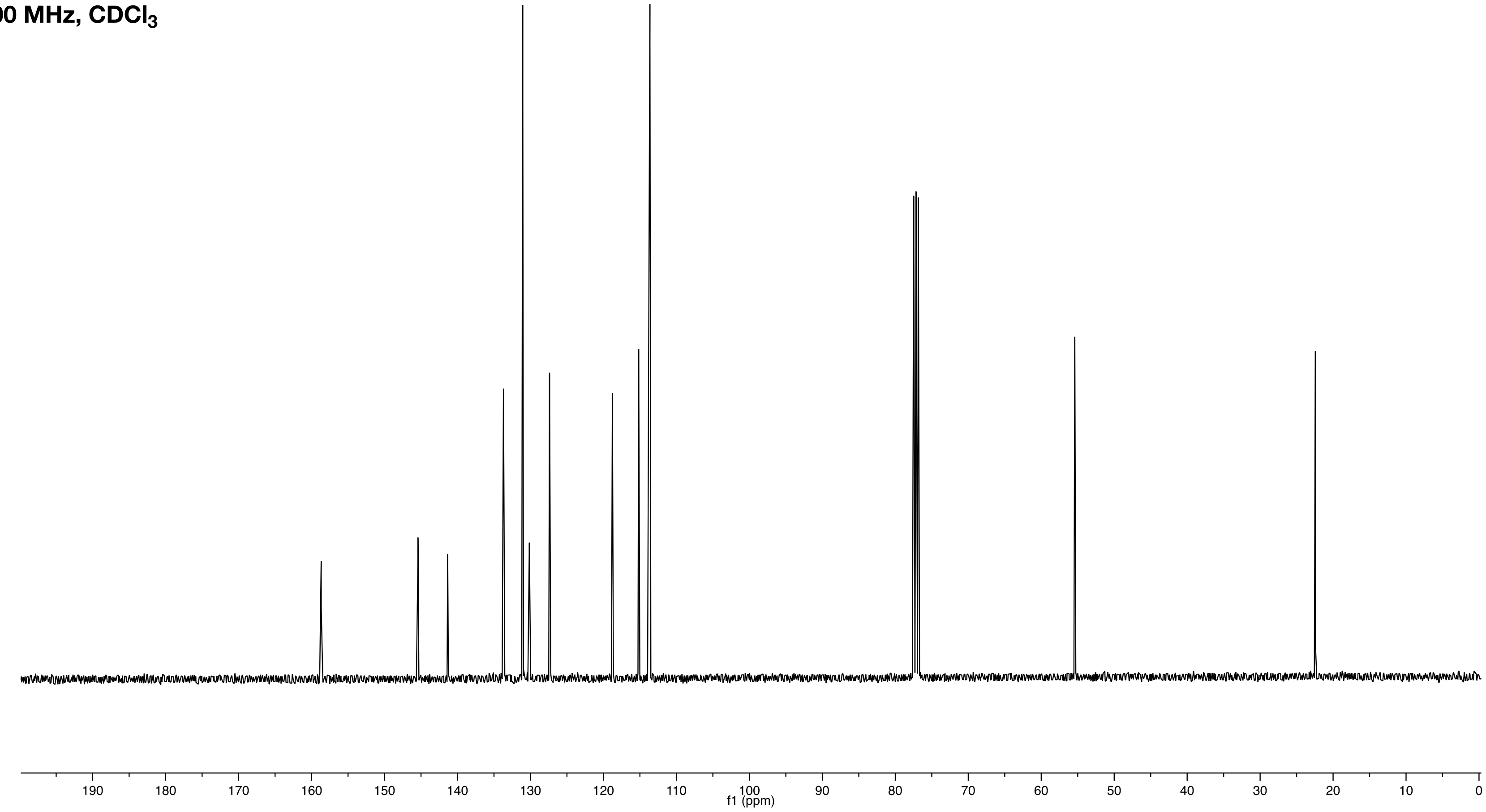

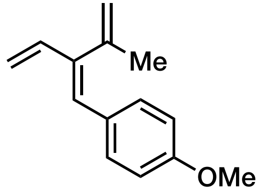

4n-*Z*

400 MHz, $CDCl_3$

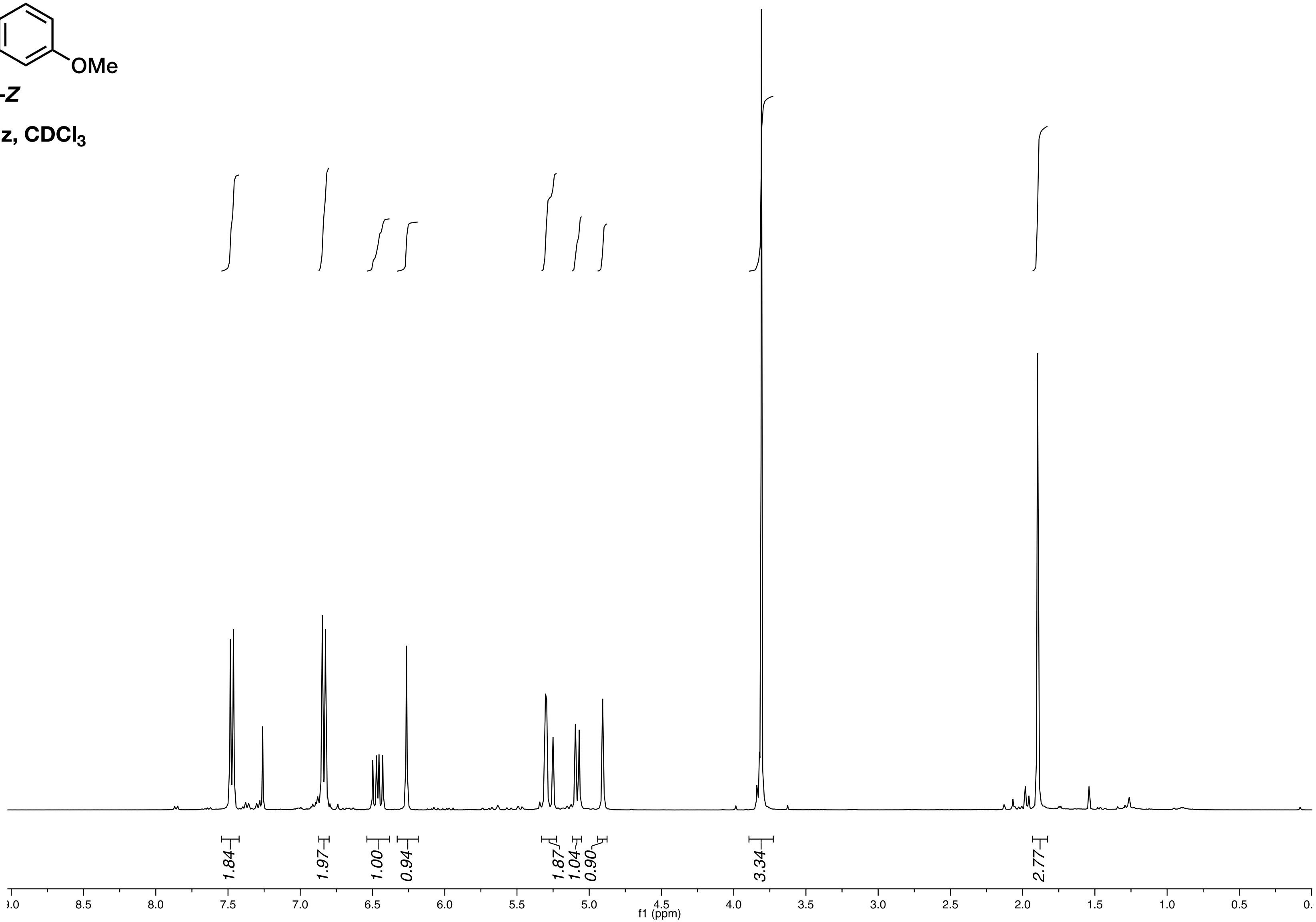

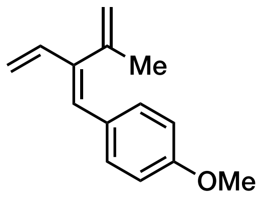

4n-*Z*

100 MHz, $CDCl_3$

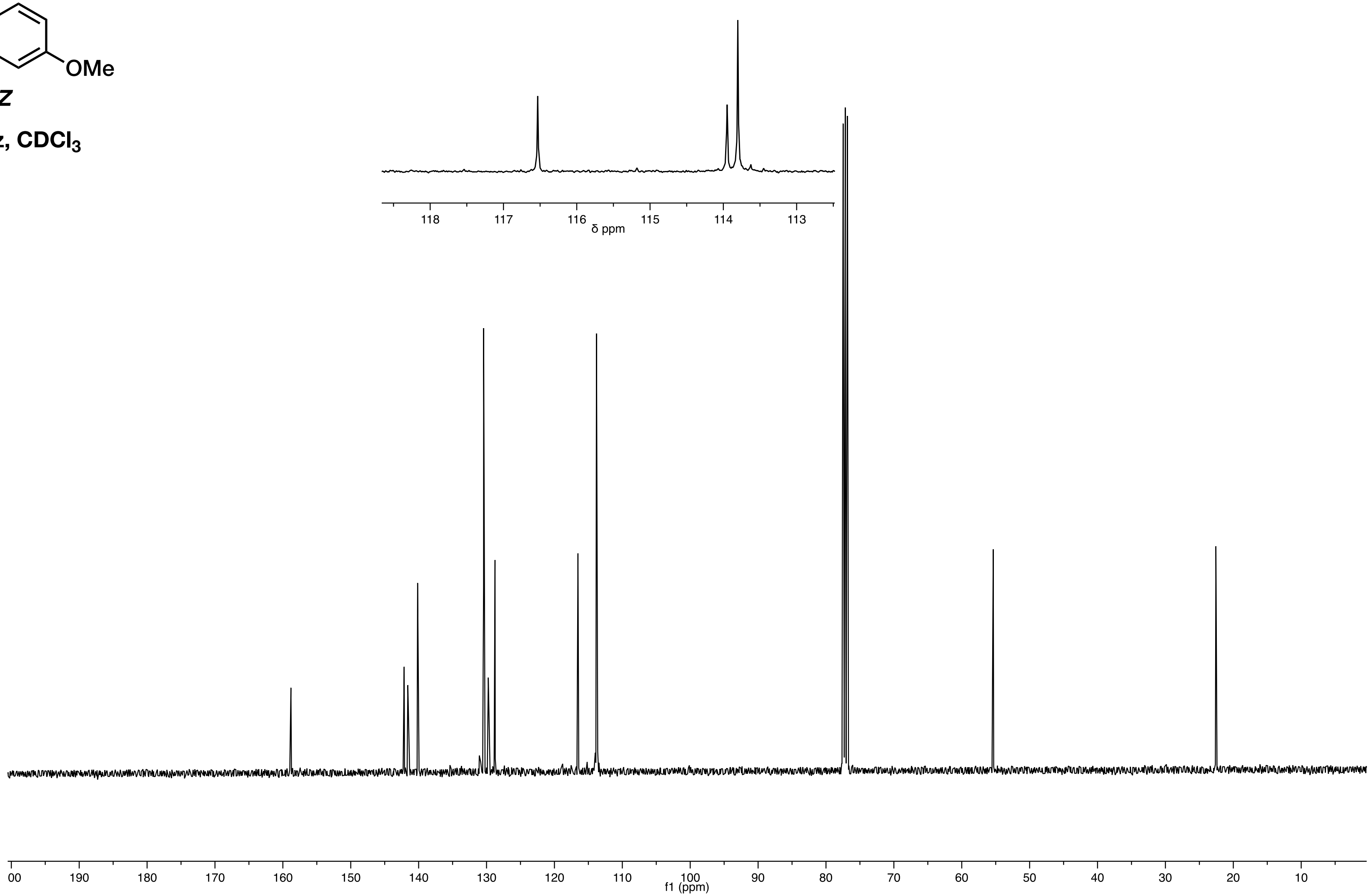

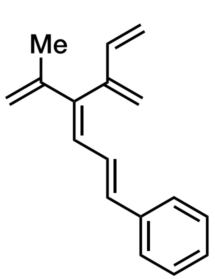

10a-*Z*

400 MHz, $(CD_3)_2CO$

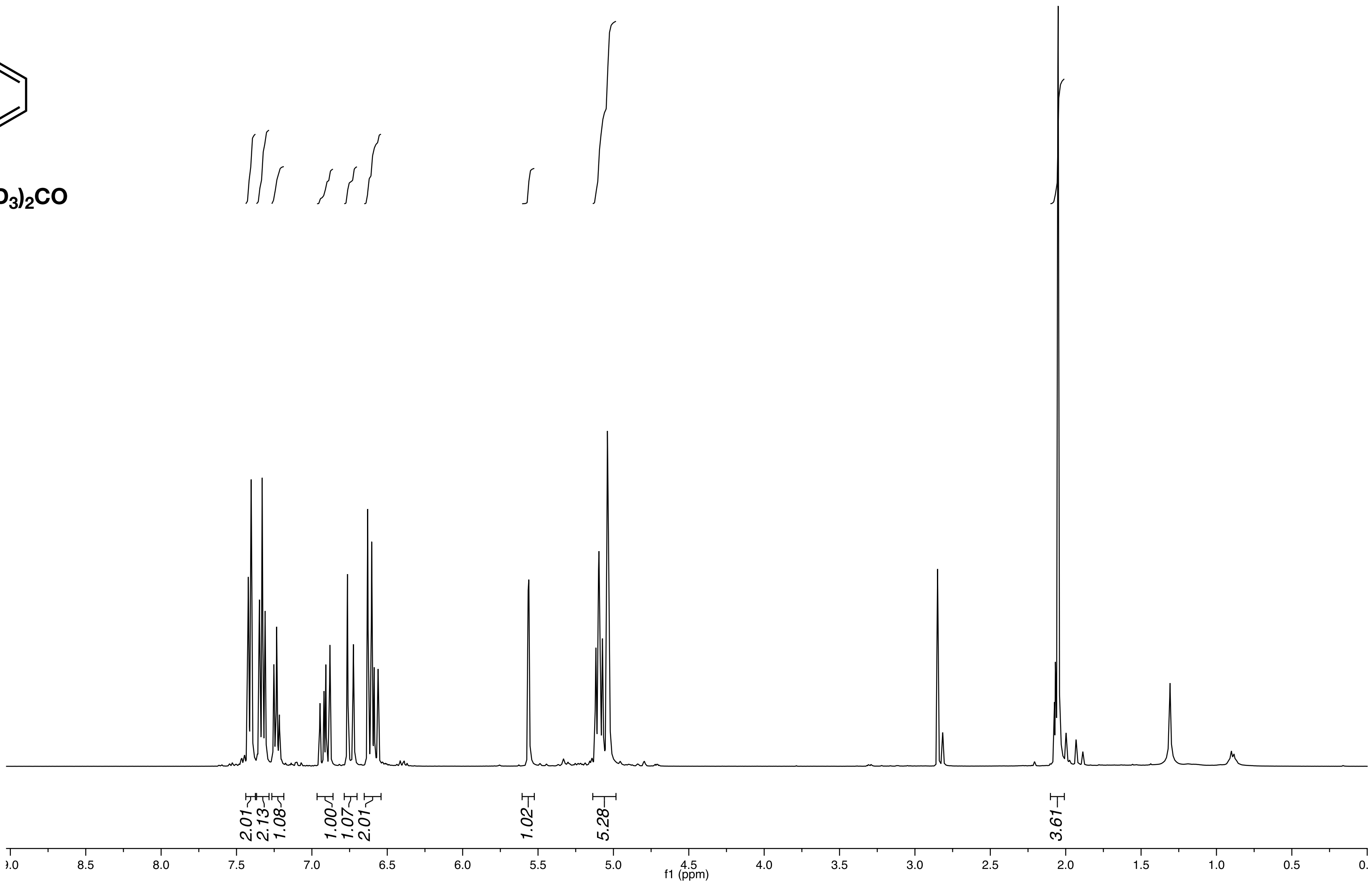

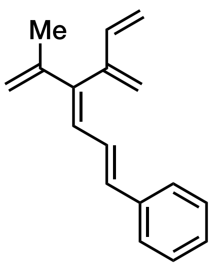

10a-*Z*

100 MHz, $(CD_3)_2CO$

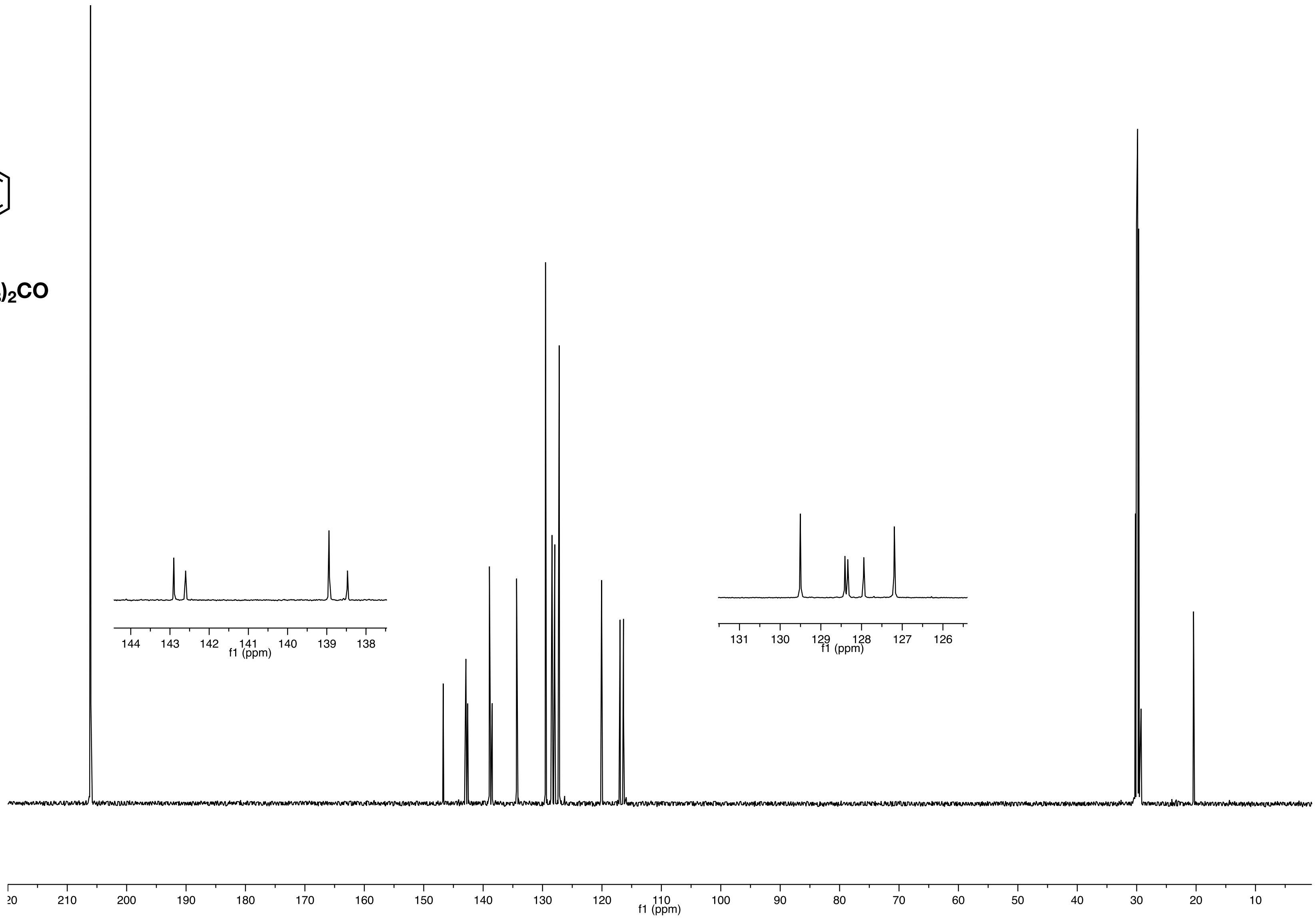

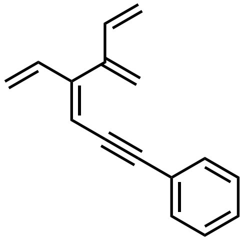

10b-*Z*

400 MHz, $(CD_3)_2CO$

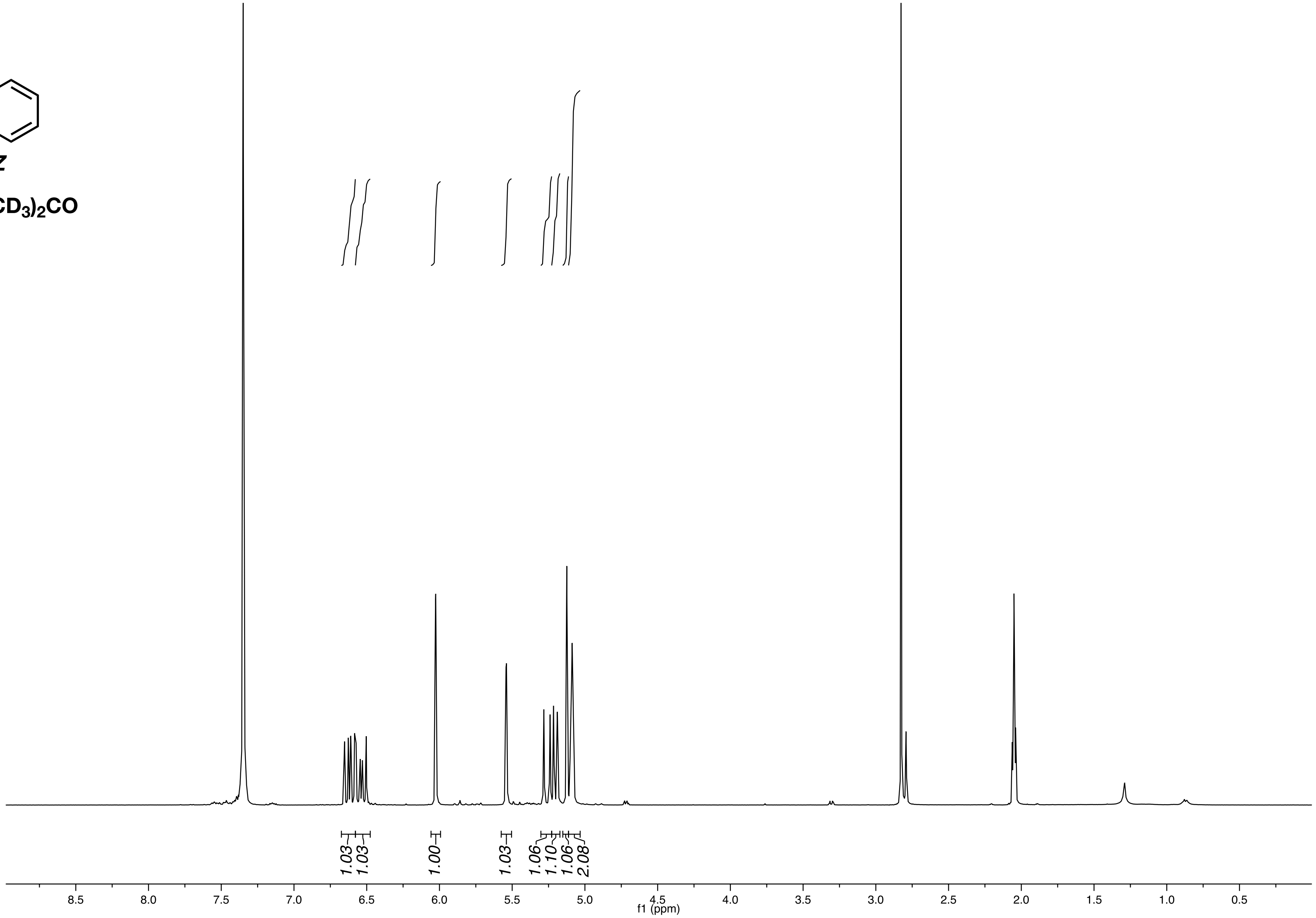

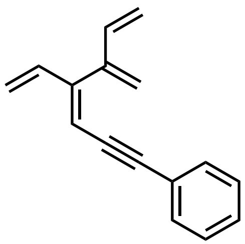

10b-*Z*

100 MHz, $(CD_3)_2CO$

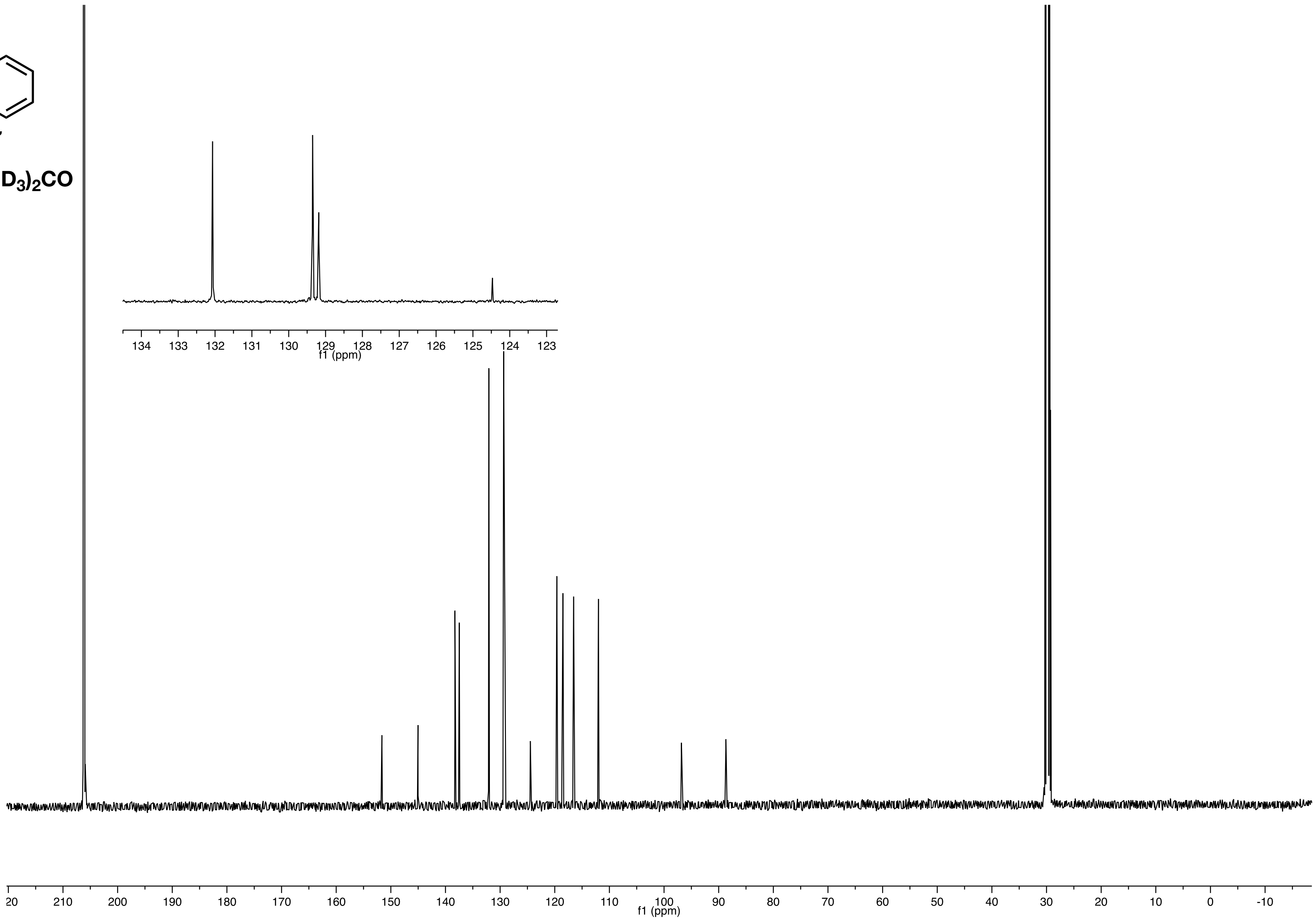

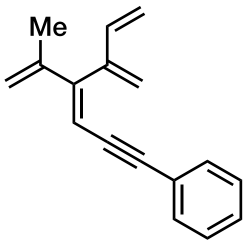

10c-*Z*

400 MHz, C_6D_6

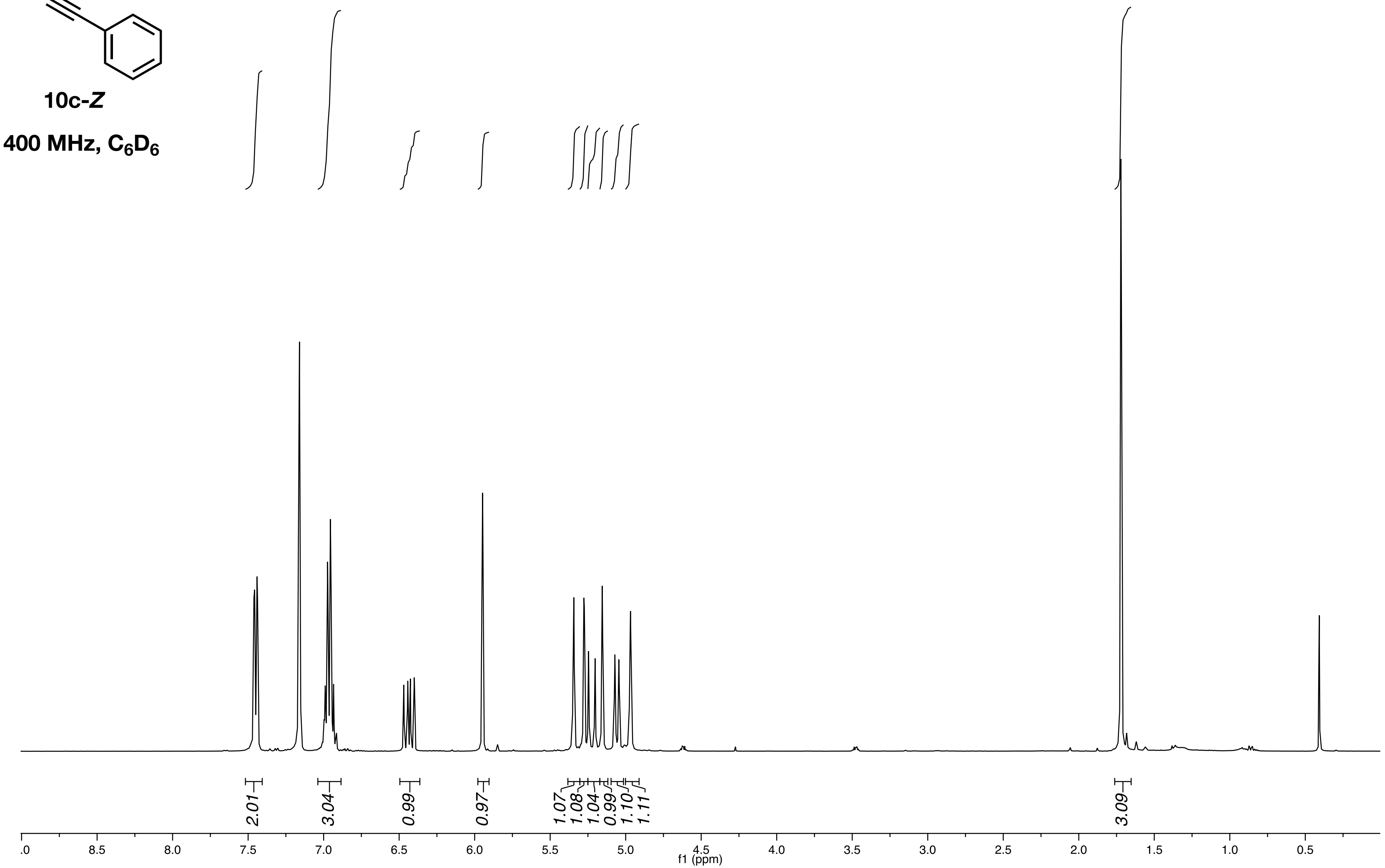

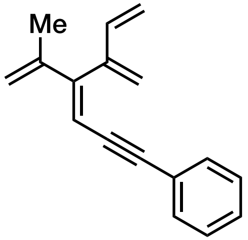

10c-*Z*

100 MHz, C_6D_6

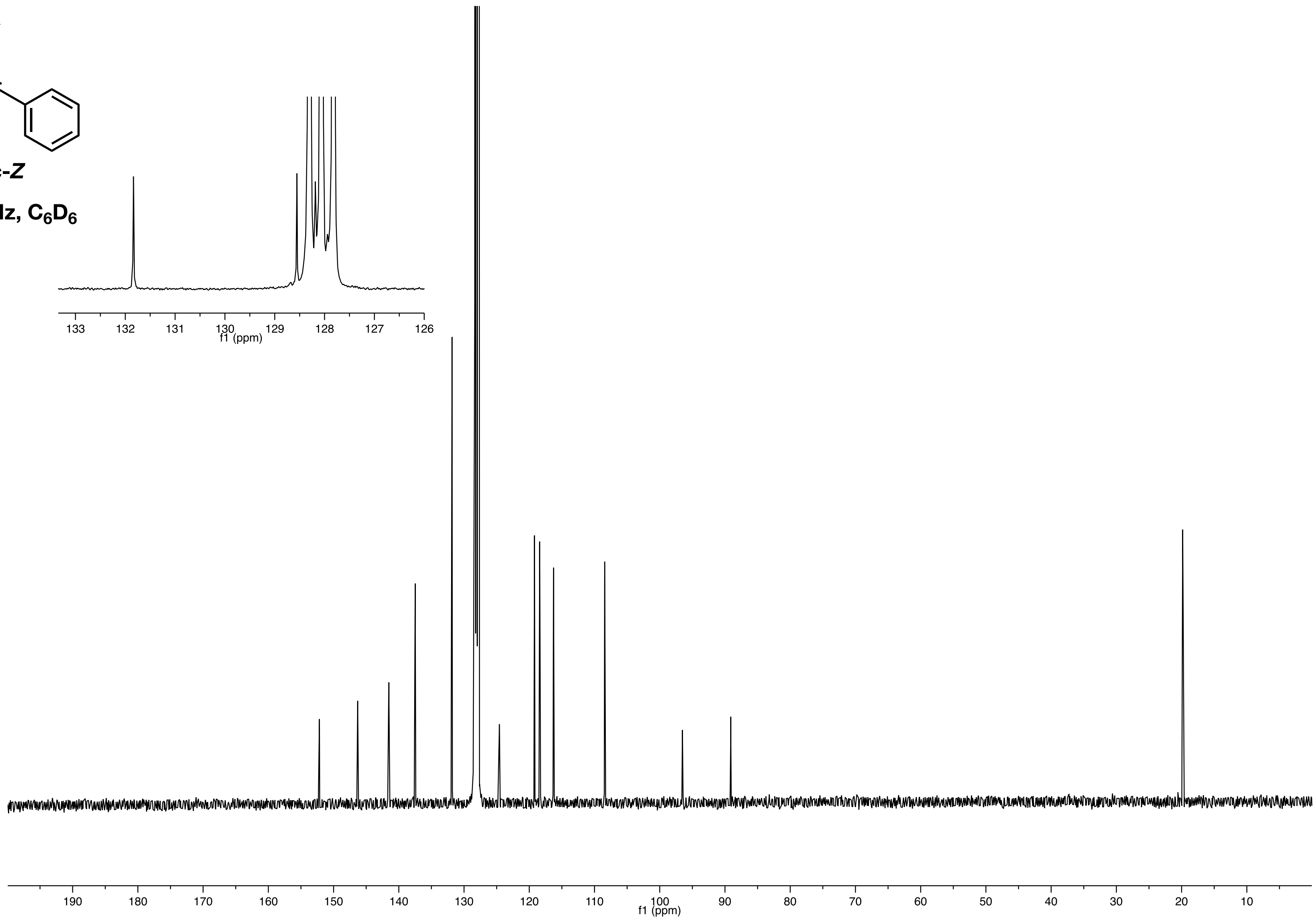

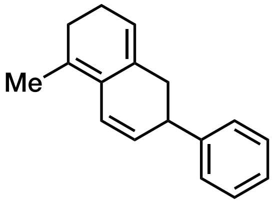

12

400 MHz, $CDCl_3$

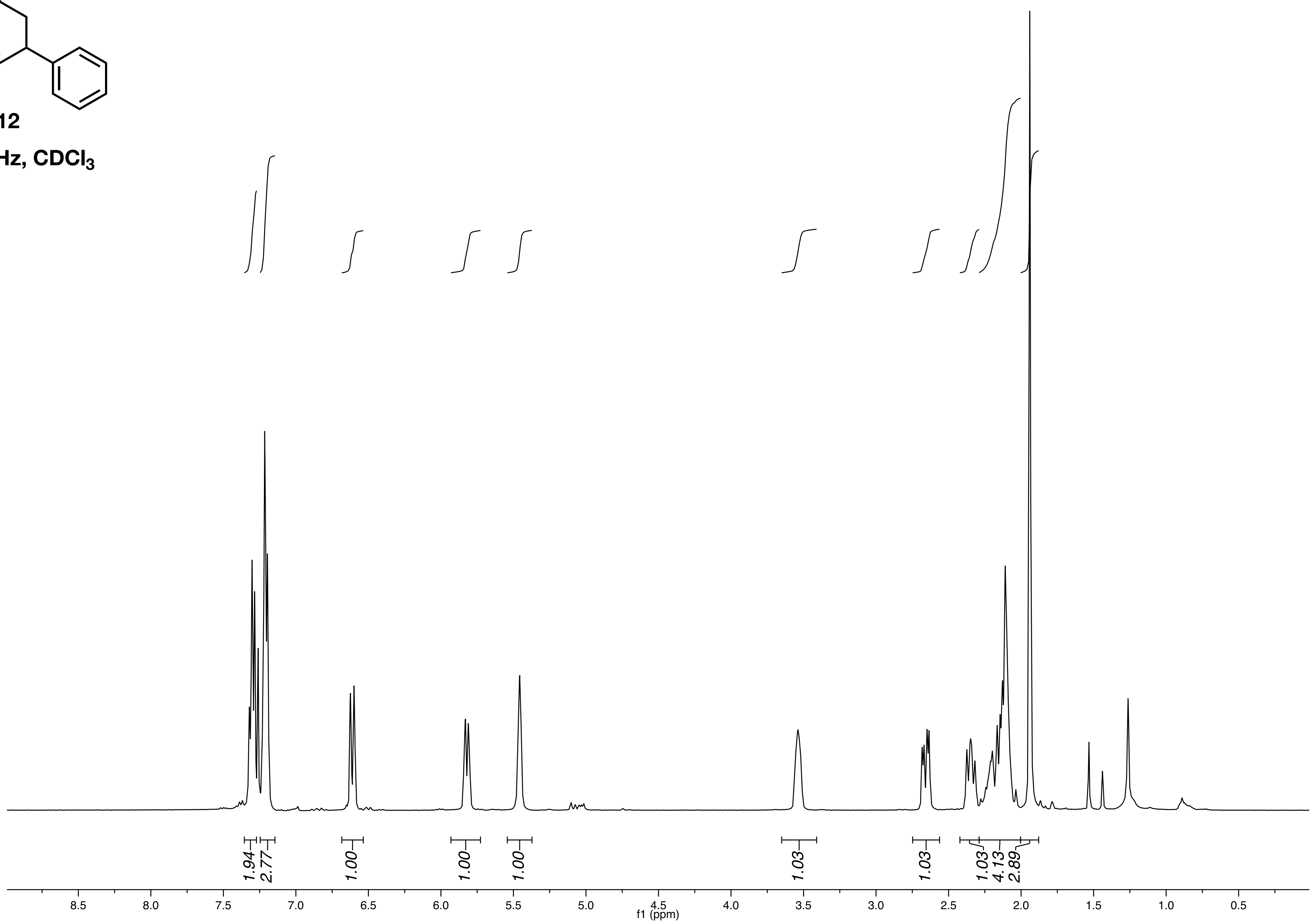

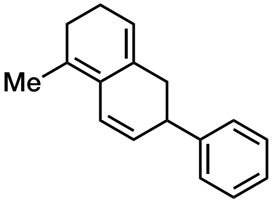

12

100 MHz, $CDCl_3$

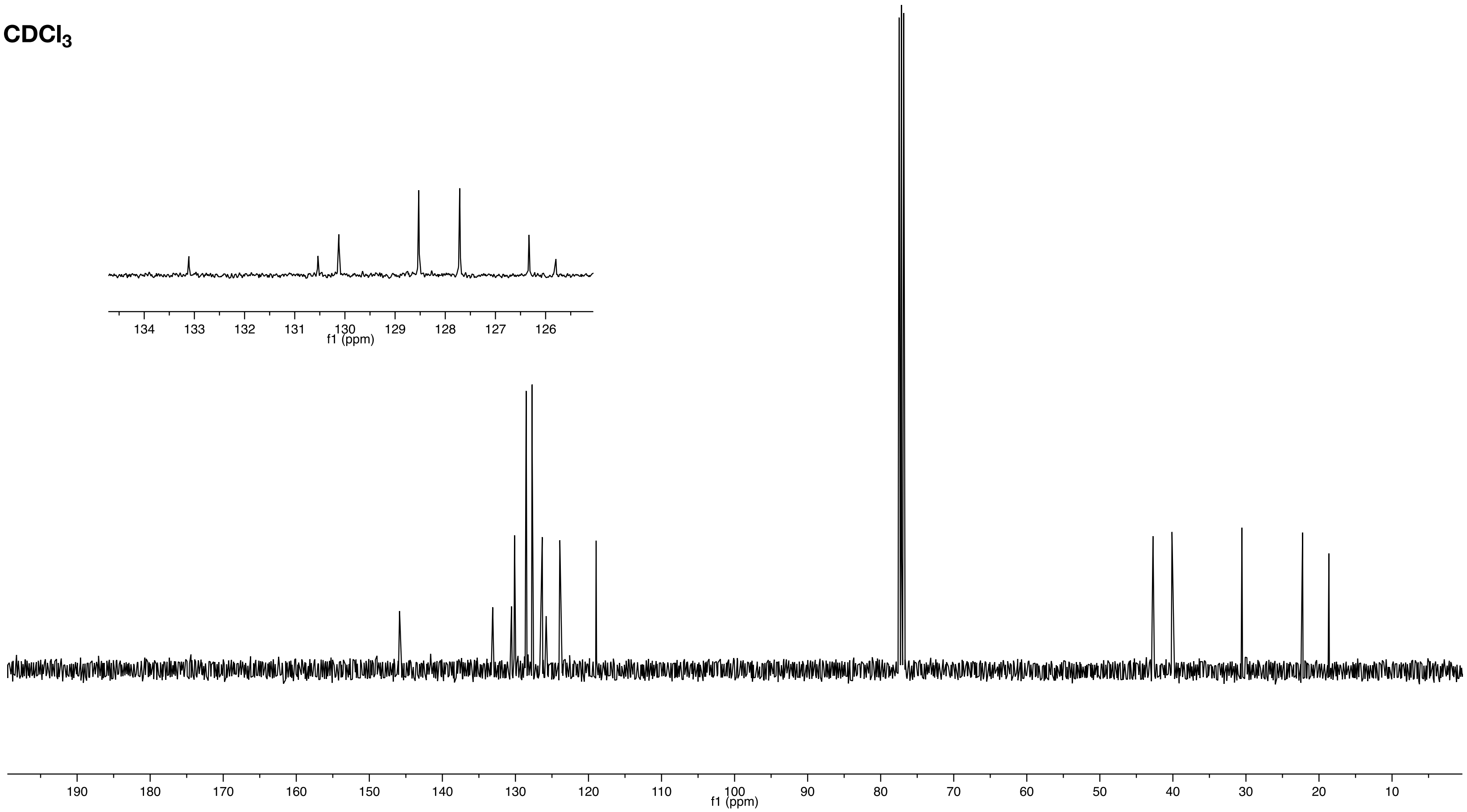

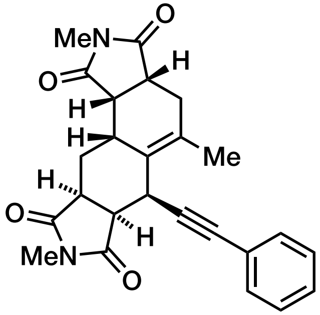

7
CD_2Cl_2, 400 MHz

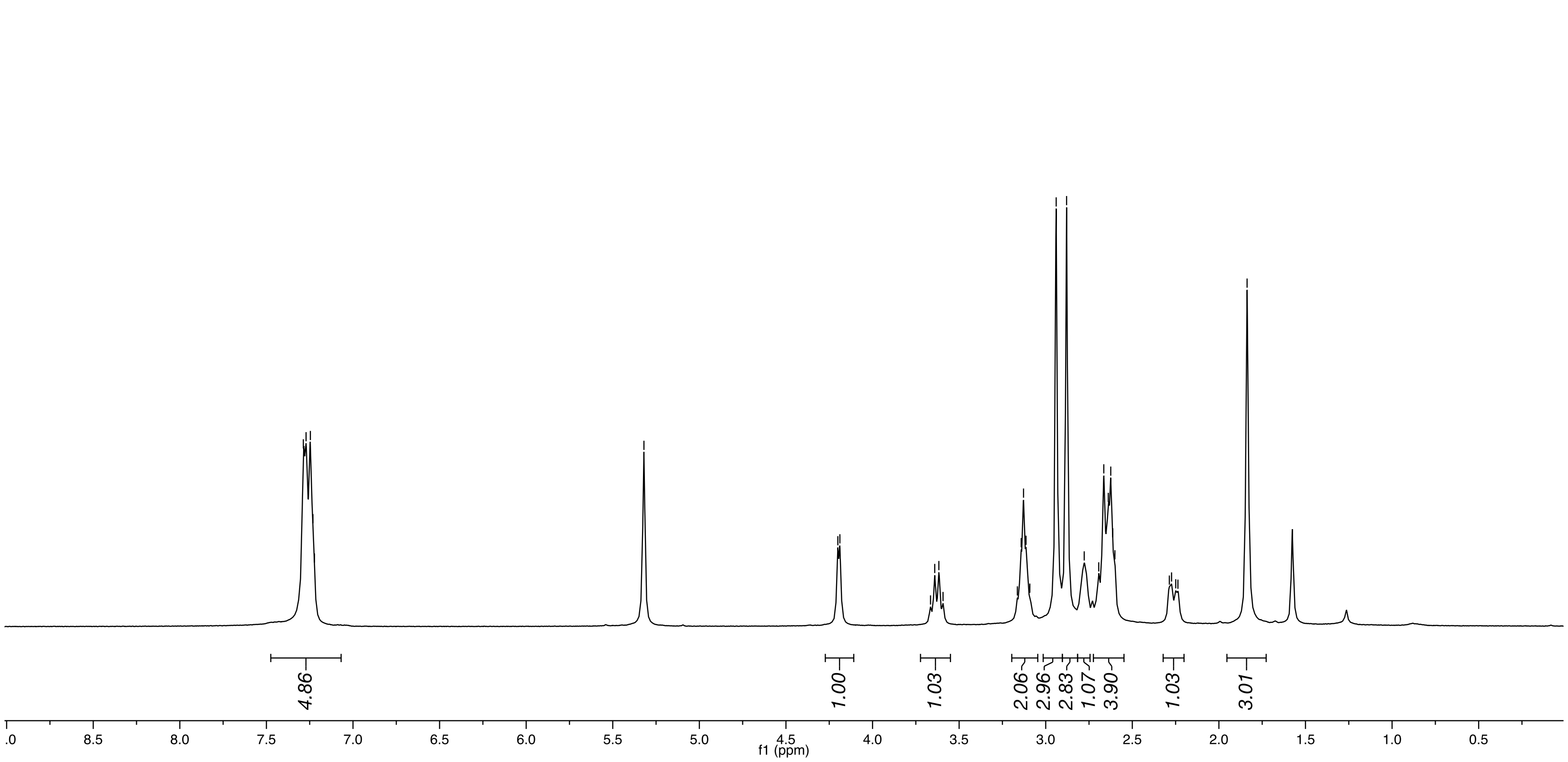

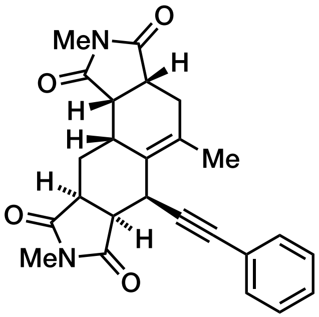

7
CD_2Cl_2, 100 MHz

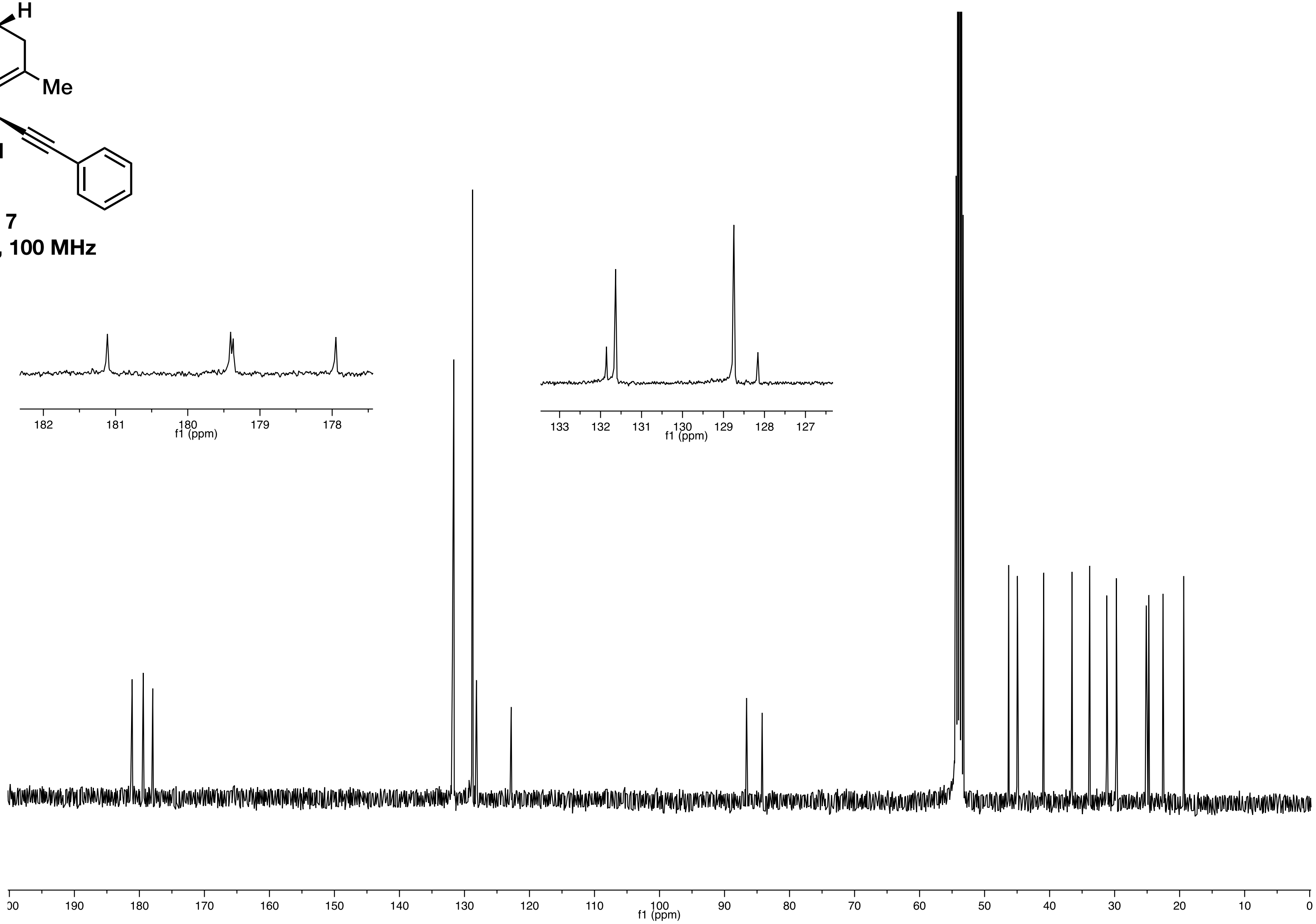

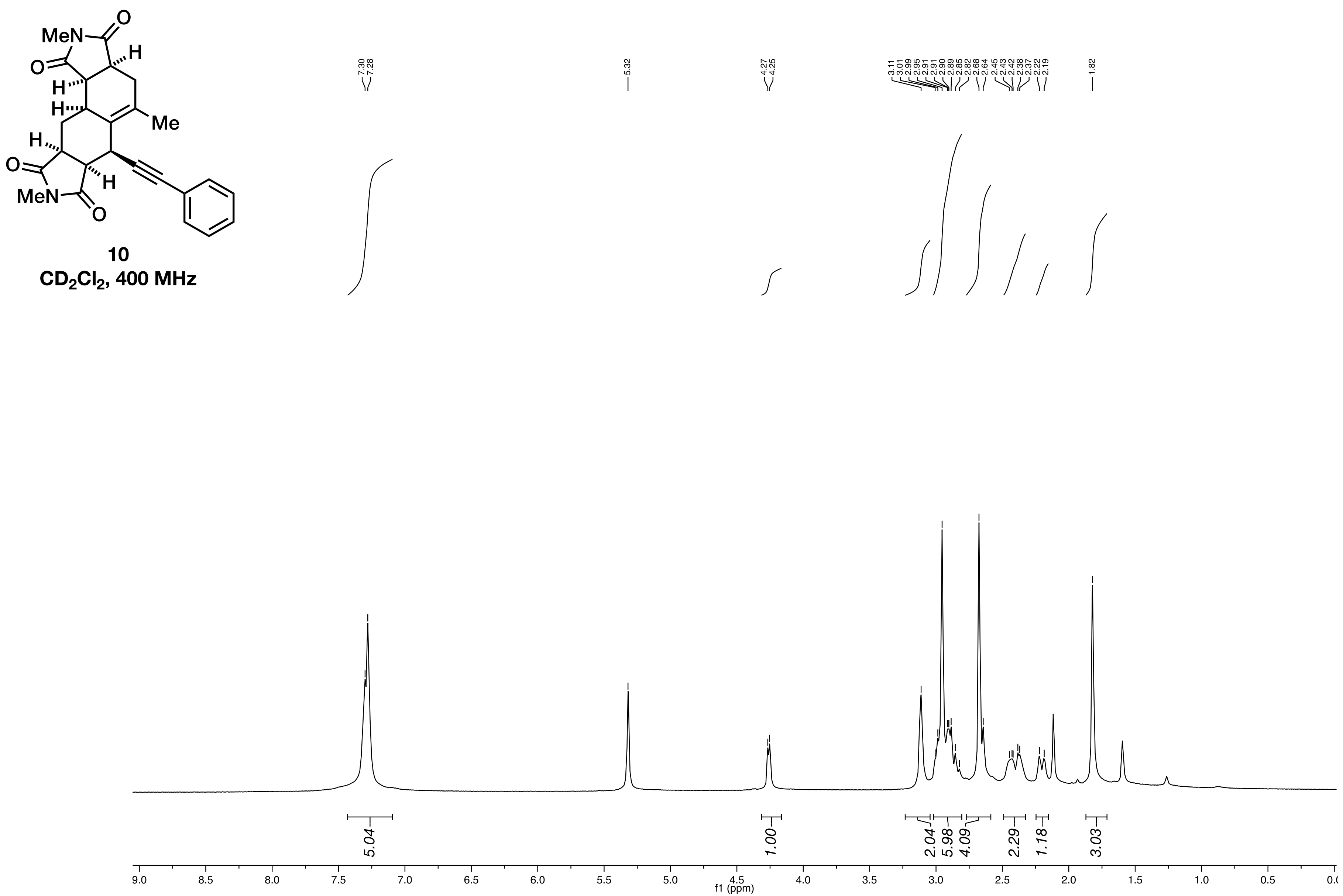
MeN
O
H
Me
MeN
10
CD2Cl2, 400 MHz
7.30
7.28
5.32
4.27
4.25
3.11
3.01
2.99
2.95
2.91
2.91
2.90
2.89
2.85
2.82
2.68
2.64
2.45
2.43
2.42
2.38
2.37
2.22
2.19
1.82
5.04
1.00
2.04
5.98
4.09
2.29
1.18
3.03
f1 (ppm)

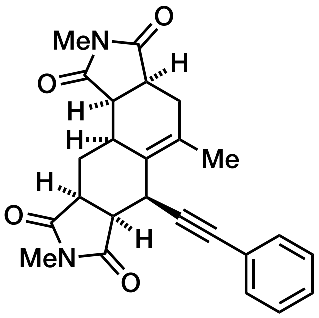

10
CD_2Cl_2, 100 MHz

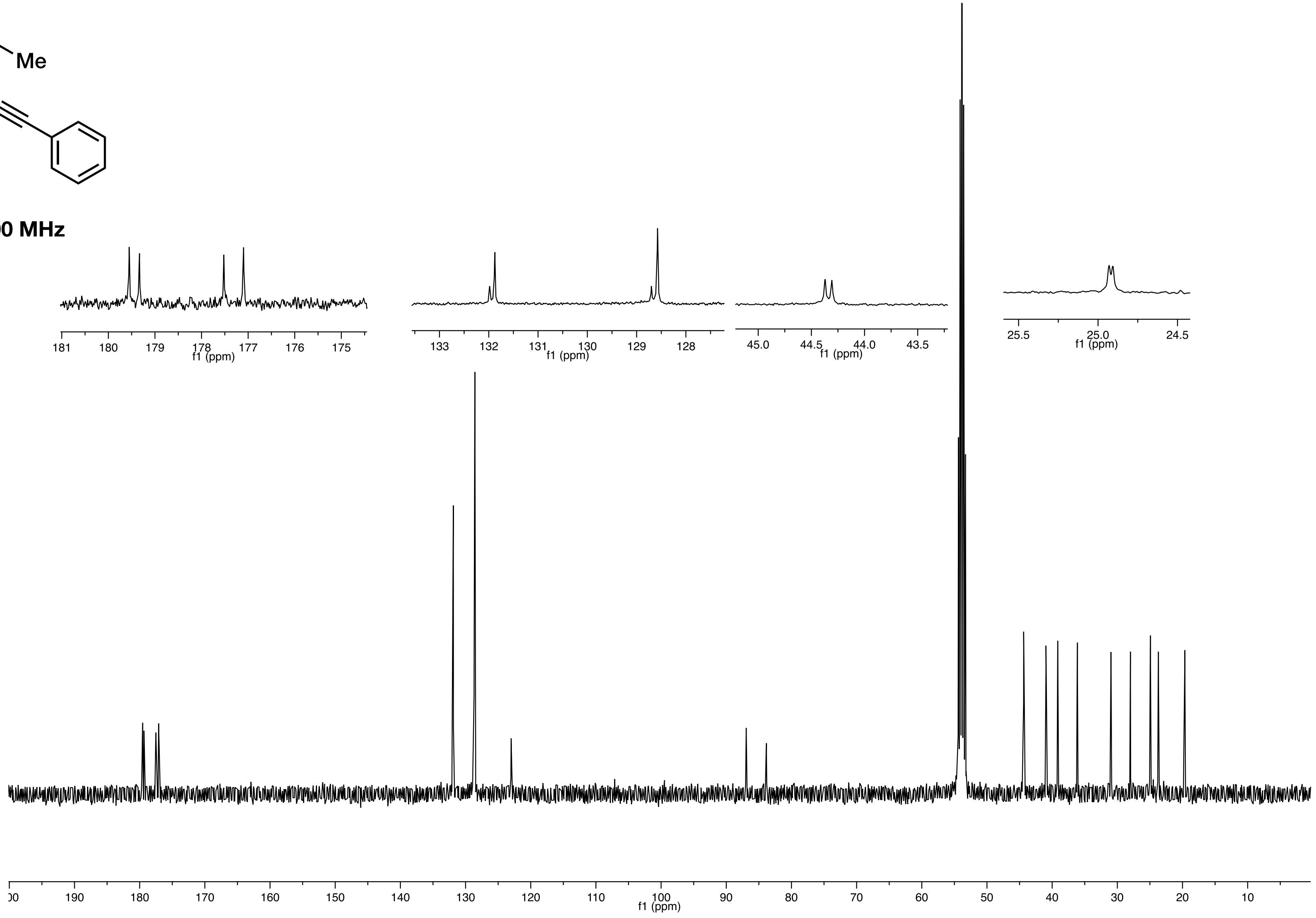

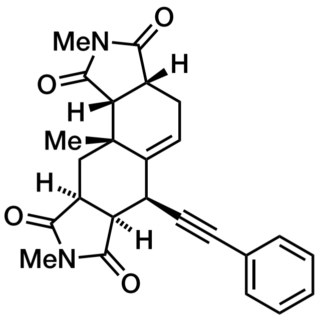

9

C_6D_6, 400 MHz

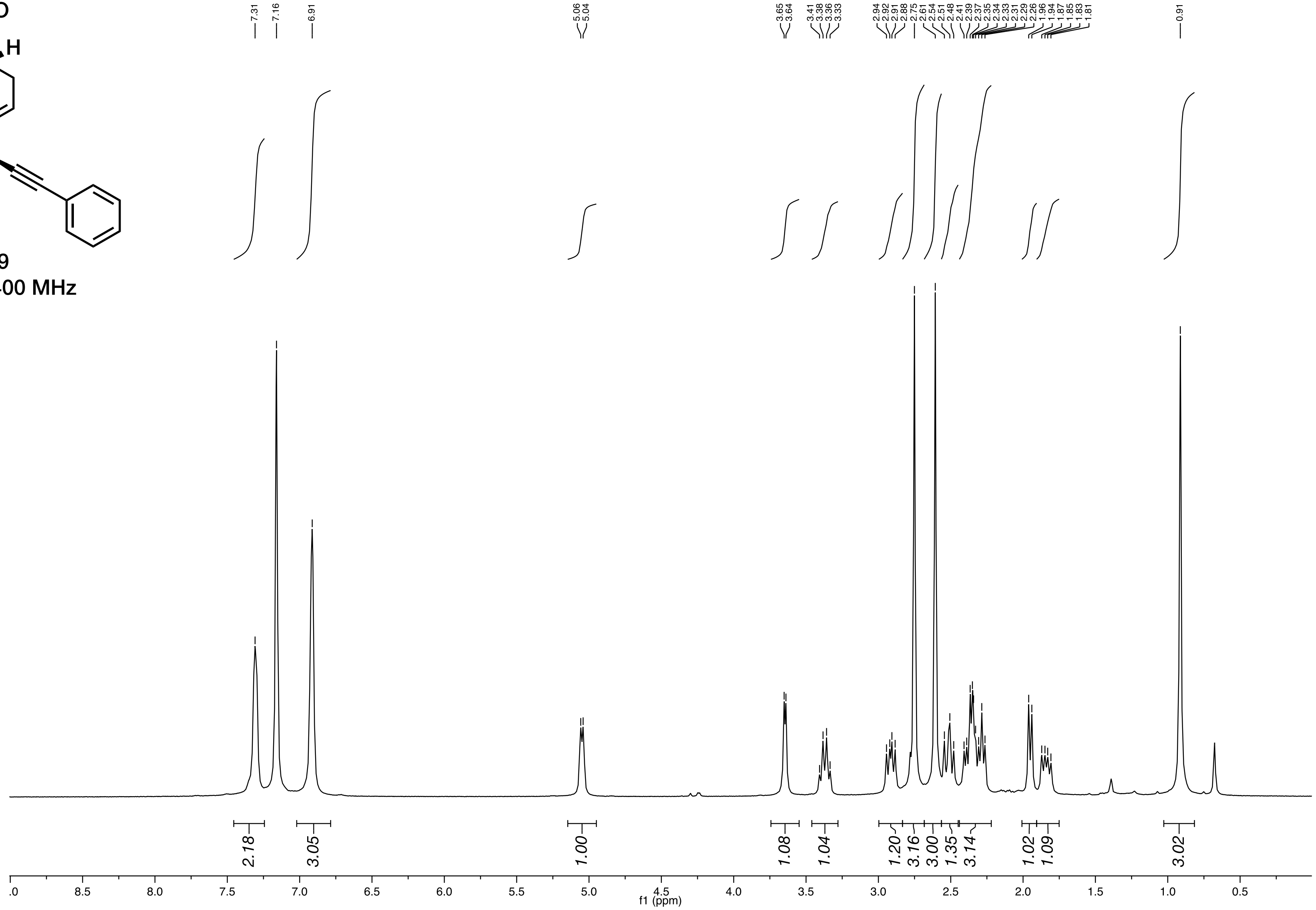

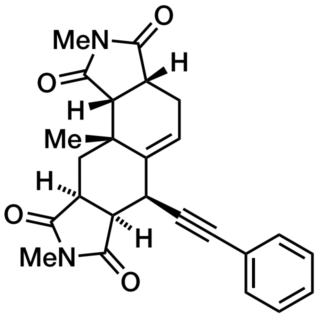

9
C_6D_6, 100 MHz

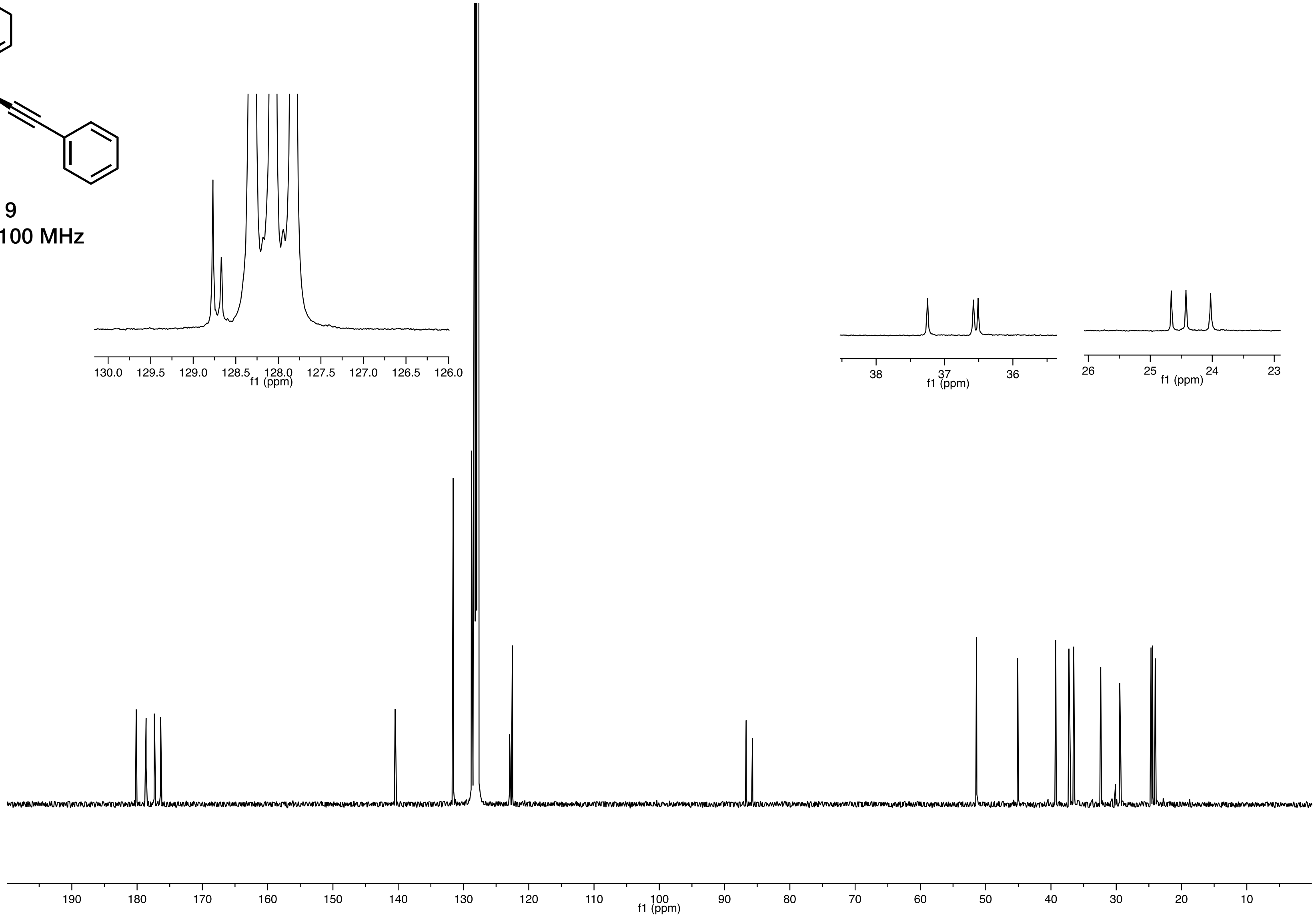

Supplement: Supplementary file 1 [file SC-010-C9SC03976G-s001.pdf]
